# Supplementary material for: Synthetic exploration of sulfinyl radicals using sulfinyl sulfones
Source: Nat Commun. 2021 Sep 2;12:5244. doi: 10.1038/s41467-021-25593-5 (PMC8413321; doi:10.1038/s41467-021-25593-5)
Supplement: Supplementary file 1 — Supplementary Information [file 41467_2021_25593_MOESM1_ESM.pdf]

Synthetic exploration of sulfinyl radicals using sulfinyl sulfones

Supplementary Information

Zikun Wang[1]†, Zhansong Zhang[1]†, Wanjun Zhao[1], Paramasivam Sivaguru[1], Giuseppe Zanoni[2], Yingying Wang[1], Edward A. Anderson[3] and Xihe Bi[1,4]*

[1] *Department of Chemistry, Northeast Normal University, Changchun 130024, China;*
[2] *Department of Chemistry, University of Pavia, Viale Taramelli 12, 27100, Pavia, Italy;*
[3] *Chemistry Research Laboratory, University of Oxford, 12 Mansfield Road, Oxford, OX1 3TA, U.K.;*
[4] *State Key Laboratory of Elemento-Organic Chemistry, Nankai University, Tianjin 300071, China;*
† These authors contributed equally to this work;
** Corresponding author: bixh507@nenu.edu.cn.*

Table of contents

Supplementary Methods 2
- I. General experimental details 2
- II. General synthetic procedures 2
- III. Study on the instability of sulfinyl sulfone **1** 6
- IV. Synthetic utility of **2** 6
- V. Mechanistic investigations 10
- VI. Analysis of EPR spectra 12
- VII. X-Ray crystallographic data 13
- VIII. Density functional theory calculations 19
- IX. Characterization data of the products 45
- X. Copies of NMR spectra 73

Supplementary References 235

Supplementary Methods

I. General experimental details

All reagents and solvents were purchased from commercial sources and used without purification unless otherwise stated. The products were purified by column chromatography over silica gel (200-400 size). ^{1}H, ^{13}C and ^{19}F Nuclear Magnetic Resonance (NMR) spectra were recorded at 25 °C on a Bruker 600 MHz, 150 MHz and 564 MHz, respectively and TMS was used as internal standard. Coupling constants are given in hertz. The following abbreviations are used: s, singlet; d, doublet; t, triplet; q, quadruplet, quint., quintet, m, multiplet. Mass spectra were recorded on TSQ 8000 Evo by using EI method. High resolution mass spectra (HRMS) were recorded on Bruck microTof by using ESI method.

II. General synthetic procedures

General procedure for sulfinylsulfonation of alkynes from 1 :

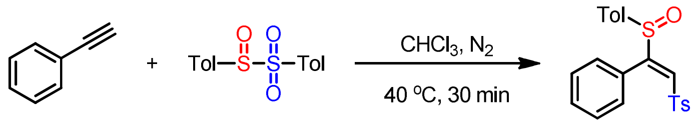

In the glove box, the solution of sulfinyl sulfone **1** (441 mg, 1.5 mmol) and phenylacetylene (102 mg, 1.0 mmol) in $CHCl_3$ (3 mL) was transferred into the Schlenk tube. The reaction mixture was stirred at 40 °C for 30 min. After the completion of reaction, CH_2Cl_2 (10 mL) was poured into the reaction mixture. The organic layers were extracted with water (3 × 20 mL). The combined organic layers were dried with anhydrous Na_2SO_4 and filtered and solvent was removed under reduced pressure. The resulting crude product was purified by column chromatography on silica gel using Petroleum ether/EtOAc/DCM (10/1/1) as eluent to afford **2** with 98% yield.

General procedure for sulfinylsulfonation of enynes:

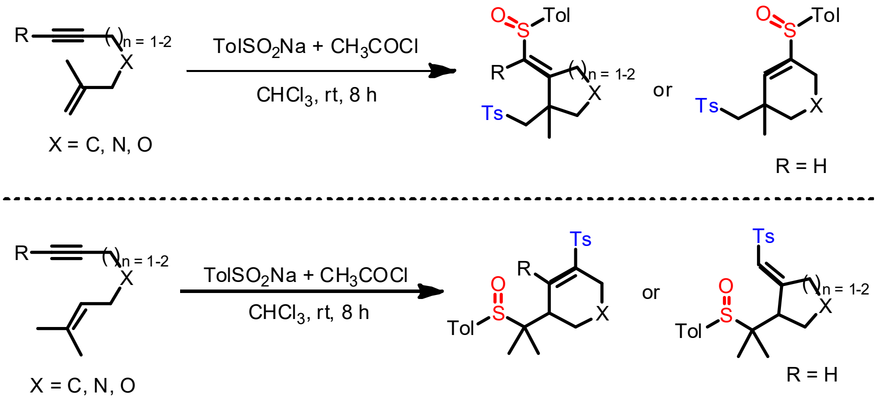

Oven dried Schlenk tube was repeatedly purged with N_2 and added *p*-toluene sulfinate (320 mg, 1.8 mmol). The solution of enyne (0.3 mmol) and CH_3COCl (94.2 mg, 1.2 mmol) in $CHCl_3$ (3 mL) was transferred into the tube. The reaction mixture was stirred at room temperature for 8 h. After the completion of reaction, CH_2Cl_2 (10 mL) was poured into the reaction mixture. The organic layers were extracted with water (3 × 20 mL). The combined organic layers were dried with anhydrous Na_2SO_4 and filtered and solvent was removed under reduced pressure. The resulting crude product was purified by column chromatography on silica gel using Petroleum ether/EtOAc/DCM (5/1/1) as eluent to afford the corresponding product.

The procedure for sulfinylsulfonation of ethylene/1,3-butadiene:

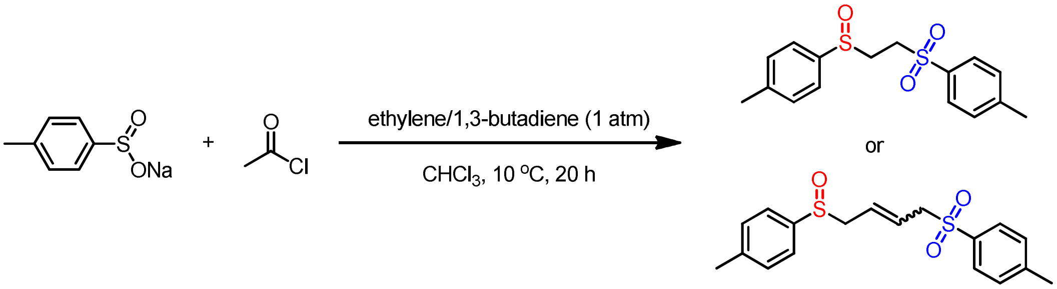

Put sodium sulfinate (320 mg, 1.8 mmol) into an oven dried Schlenk tube, replace it with nitrogen three times, and then connect the ethylene/1,3-butadiene gas ballon under vacuum. The solution CH_3COCl (94.2 mg, 1.2 mmol) in $CHCl_3$ (3 mL) was transferred into the tube. The reaction mixture was stirred at 10 °C for 20 h. After the completion of reaction, CH_2Cl_2 (10 mL) was poured into the reaction mixture. The organic layers were extracted with water (3 × 20 mL). The combined organic layers were dried with anhydrous Na_2SO_4 and filtered and solvent was removed under reduced pressure. The resulting crude product was purified by column chromatography on silica gel using Petroleum ether/EtOAc/DCM (5/1/1) as eluent to afford the corresponding product.

The procedure for the synthesis of 100,102,106:

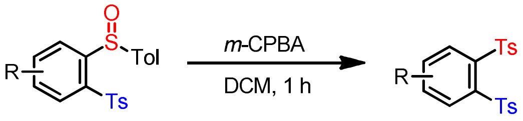

99, 101, 103 **100, 102, 104**

An oven dried 25 mL round-bottomed flask was charged with **99, 101, 103** (0.5 mmol), *m*-CPBA (0.75 mmol) and DCM (3 mL) , The reaction mixture was stirred at room temperature for 1 h. After completion of the reaction, 10 mL saturated Na_2SO_3 solution was added and the resulting mixture was extracted with DCM (3 × 10 mL) and the organic layer was combined and dried with anhydrous $NaSO_4$. The combined organic extracts were washed with water, brine, dried over anhydrus $NaSO_4$, and concentrated under reduced pressure. The resulting crude product was purified by column chromatography on silica gel using Petroleum ether/EtOAc/DCM (8:1:1) as eluent to afford the **100, 102, 104**.

The procedure for the synthesis of 124:

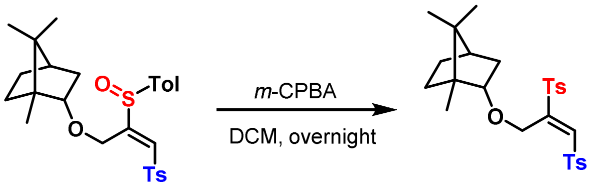

121 **124**, 86%, dr = 1:1

An oven dried 25 mL round-bottomed flask was charged with **121** (97.2mg, 0.2 mmol), *m*-CPBA (68.8 mg, 0.4 mmol) and DCM (3 mL).The reaction mixture was stirred at room temperature for overnight. After completion of the reaction, 10 mL saturated Na_2SO_3 solution was added and the resulting mixture was extracted with DCM (3 × 10 mL) and the organic layer was combined and dried with anhydrous $NaSO_4$. The combined organic extracts were washed with water, brine, dried over anhydrus $NaSO_4$, and concentrated under reduced pressure. The resulting crude product was purified by column chromatography on silica gel using Petroleum ether/EtOAc/DCM (8:1:1) as eluent to afford the **124** with 86% yield.

The procedure for the synthesis of 125:

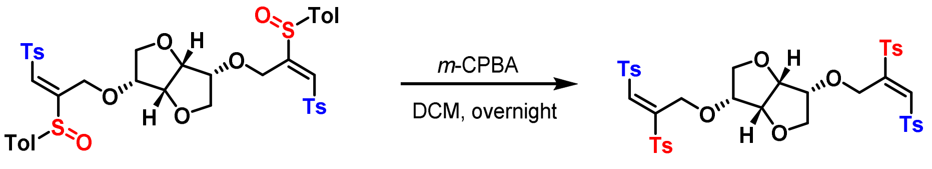

122 **125**, 84%

An oven dried 25 mL round-bottomed flask was charged with **122** (162.0mg, 0.2 mmol), *m*-CPBA (137.6 mg, 0.8 mmol) and DCM (5 mL).The reaction mixture was stirred at room temperature for overnight. After completion of the reaction, 10 mL saturated Na_2SO_3 solution was added and the resulting mixture was extracted with DCM (3 × 10 mL) and the organic layer was combined and dried with anhydrous $NaSO_4$. The combined organic extracts were washed with water, brine, dried over anhydrus $NaSO_4$, and concentrated under reduced pressure. The resulting crude product was purified by column chromatography on silica gel using Petroleum ether/EtOAc/DCM (8:3:1) as eluent to afford the **125** with 84% yield.

The procedure for the synthesis of 128:

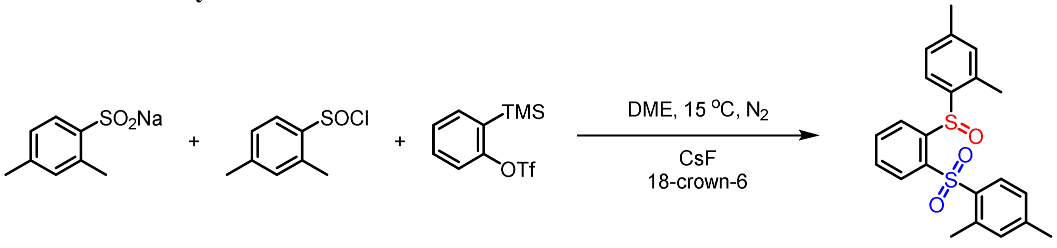

128, 52%

In the glove box, the solution of sodium 2,4-dimethylbenzenesulfinate (57.6 mg, 0.3 mmol), 2,4-dimethylbenzenesulfinic chloride (56.4 mg, 0.3 mmol), in DME (3 mL) was transferred into the Schlenk tube. The reaction mixture was stirred at 15 °C for 2 h. Then add 2-(trimethylsilyl)phenyl trifluoromethanesulfonate (89 mg, 0.3 mmol), CsF (136 mg, 0.9 mmol), 18-crown-6 (238 mg, 0.9 mmol), The reaction mixture was stirred at room temperature for 5 h, After the completion of reaction, CH_2Cl_2 (10 mL) was poured into the reaction mixture. The organic layers were extracted with water (3 × 20 mL). The combined organic layers were dried with anhydrous Na_2SO_4 and filtered and solvent was removed under reduced pressure. The resulting crude product was purified by column chromatography on silica gel using Petroleum ether/EtOAc/DCM (5/1/1) as eluent to afford the **128** with 52% yield.

The procedure for the synthesis of 129:

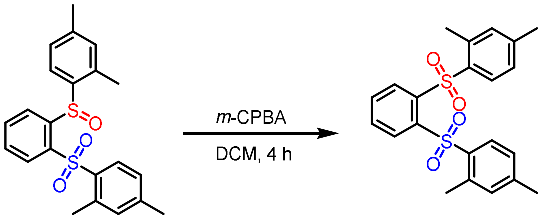

128 **129**, 91%

An oven dried 25 mL round-bottomed flask was charged with **128** (119 mg, 0.3 mmol), *m*-CPBA (0.9 mmol) and DCM (3 mL), The reaction mixture was stirred at room temperature for 4 h. After completion of the reaction, 10 mL saturated Na_2SO_3 solution was added and the resulting mixture was extracted with DCM (3 × 10 mL) and the organic layer was combined and dried with anhydrous $NaSO_4$. The combined organic extracts were washed with water, brine, dried over anhydrus $NaSO_4$, and concentrated under reduced pressure. The resulting crude product was purified by column chromatography on silica gel using Petroleum ether/EtOAc/DCM (8:1:1) as eluent to afford the **129** with 91% yield.

The procedure for the synthesis of 130:

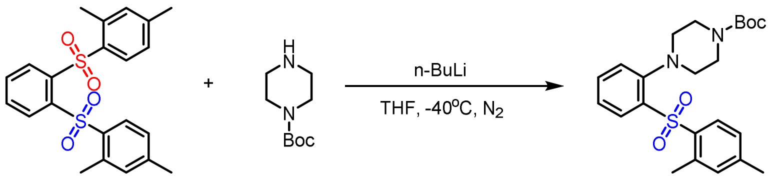

129 **130**, 84%

To a solution of tert-butyl piperazine-1-carboxylate (186 mg, 1 mmol) in THF (3 mL) was added slowly n-BuLi (0.6 mL, 1.6 mol/L in hexane) under -40 °C. After stirring for 30 min, **129** (82 mg, 0.2 mmol) was added to the mixture under the same temperature. The mixture was then stirred for 30 min. After completion of the reaction, methol was added and the resulting mixture was warmed to room temperature. Then the mixture was concentrated under reduced pressure. The resulting crude product was purified by column chromatography on silica gel using Petroleum ether/EtOAc (15:1) as eluent to afford the **130** with 84% yield.

The procedure for the synthesis of 131:

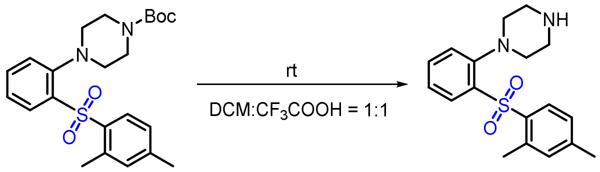

130 **131**, 93%

An oven dried 25 mL round-bottomed flask was charged with **130** (86 mg, 0.2 mmol), CF_3COOH (2 mL) and DCM (2 mL), The reaction mixture was stirred at room temperature for 1 h. After completion of the reaction, the solvent was removed, the residue in DCM (20 mL) and extracted with saturated $NaHCO_3$ solution (20 mL). The resulting mixture was extracted with DCM (3 × 10 mL) and the organic layer was combined and dried with anhydrous $NaSO_4$. The combined organic extracts were washed with water, brine, dried over anhydrus $NaSO_4$, and concentrated under reduced pressure to afford the **131** with 93% yield.

The procedure for the synthesis of 132:

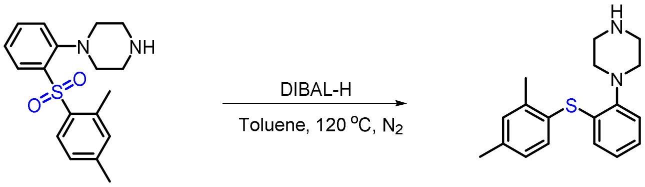

131 **132**, 42%

Oven dried Schlenk tube was repeatedly purged with N_2. The solution of **131** (66 mg, 0.2 mmol) in Toluene (1 mL) was transferred into the tube. DIBAL-H (2.7 mL, 1.5 mol/L in Toluene) was added to the mixture. The reaction mixture was stirred at 120 °C for 18 h. After the completion of reaction, the reaction mixture was cooled to room temperature and quenched by slow addition of water. The mixture was extracted with EtOAc and the combined organic layers were dried (Na_2SO_4), and concentrated. The resulting crude product was purified by column chromatography on silica gel using EtOAc/Et_3N (20:1) as eluent to afford the **132** with 42% yield.

III. Study on the instability of sulfinyl sulfone 1

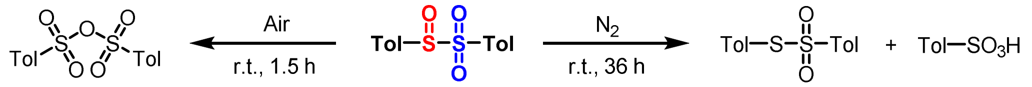

152, 98% **1** **153**, 52% **154**, 28%

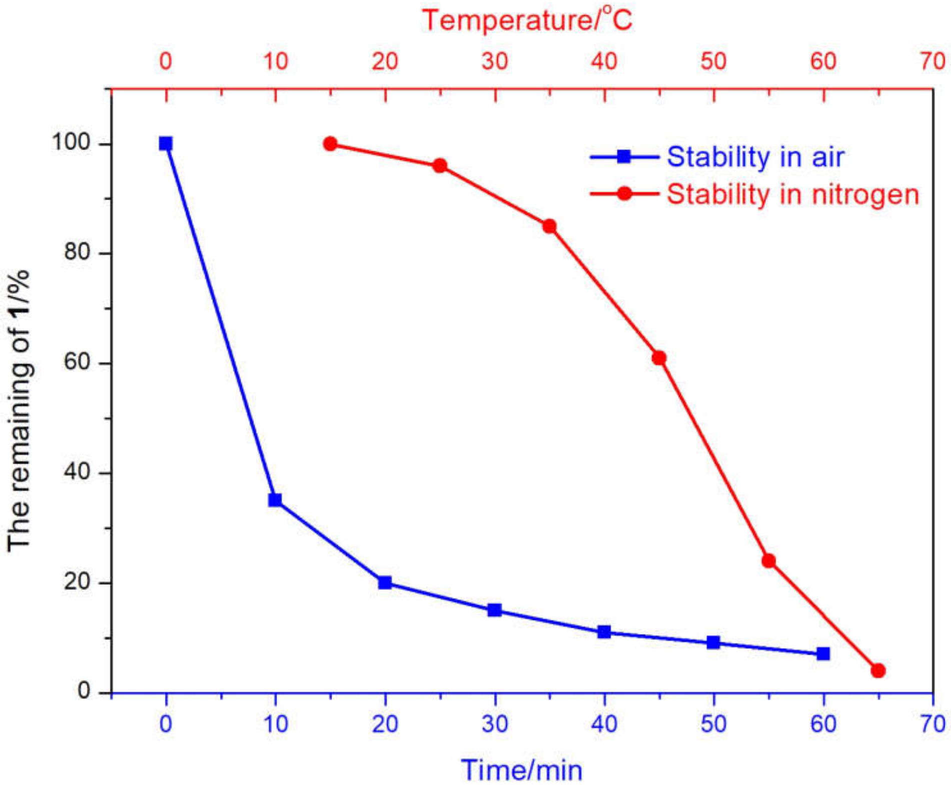

Supplementary Figure 1. The instability of sulfinyl sulfone **1**.

Study on the stability of sulfinyl sulfone 1 in air: In the glove box, the solution of sulfinyl sulfone **1** (441 mg, 1.5 mmol) and 1,3,5-trimethoxybenzene (56 mg, 0.5 mmol) in $CDCl_3$ (3 mL) was transferred into the Schlenk tube. Removd the reaction system from the glove box and stirred in the air. Every ten minutes, took samples from the reaction system for ^{1}H NMR detection. Then draw a curve based on the obtained data.

Study on the stability of sulfinyl sulfone 1 in nitrogen: In the glove box, the solution of sulfinyl sulfone **1** (441 mg, 6.0 mmol) and CH_2Br_2 (3.0 mmol) in $CDCl_3$ (18 mL) was divided into six equal parts and placed in six Schlenk tubes. The six reaction systems were placed at 15 °C, 25 °C, 35 °C, 45 °C, 55 °C, and 65 °C respectively, and stirred for 30 minutes. Then took samples from the reaction system for ^{1}H NMR detection and draw a curve based on the obtained data.

IV. Synthetic utility of 2

Multigram-scale synthesis of product 2:

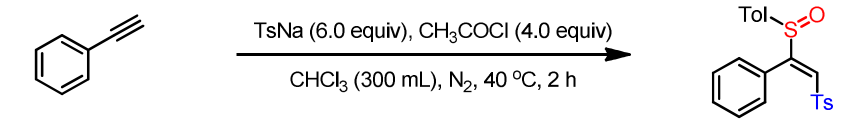

100 mmol, 10.24 g

2, 85%
85 mmol, 33.71g

Oven dried three-necked flask was repeatedly purged with N_2 and added sodium sulfinates (600 mmol, 106.8 g). The solution of phenylacetylene (100 mmol, 10.24 g) in $CHCl_3$ (200 mL) was transferred into the flask. The reaction mixture was stirred at 40 °C for 10 min. Then the solution of acetyl chloride (400 mmol, 31.4 g) in $CHCl_3$ (100 mL) was slowly dropped to the above reaction mixture. After the completion of reaction, the reaction mixture was filtered and the filtrate is collected. The solvent of the filtrate was removed under reduced pressure. The resulting crude product was washed by petroleum ether/EtOAc (20:1, 3 × 200 mL) to afford the product **2** in the yield of 85% as a white solid.

The procedure for the synthesis of 133:

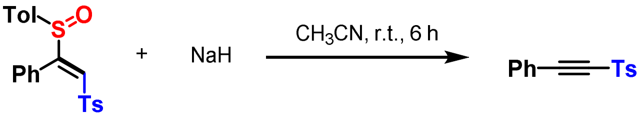

2 133

The solution of **2** (198 mg, 0.5 mmol) and NaH (0.75 mmol) in acetonitrile (3 mL) was transferred into the Schlenk tube. The reaction system was stirred at room temperature for 6 h. After the completion of reaction, saturated ammonium chloride solution (10 mL) was added to the reaction system to quench the reaction. Then CH_2Cl_2 (20 mL) was poured into the reaction mixture. The organic layers were extracted with water (3 × 20 mL). The combined organic layers were dried with anhydrous Na_2SO_4 and filtered and solvent was removed under reduced pressure. The resulting crude product was purified by column chromatography on silica gel using Petroleum ether/EtOAc (10/1) as eluent to afford the product **133** in the yield of 69% as a white solid.

The procedure for the synthesis of 134:

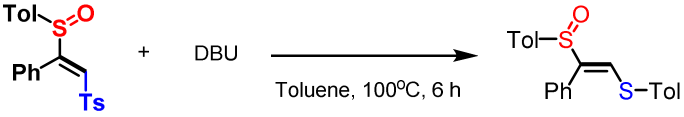

2 134

The solution of **2** (198 mg, 0.5 mmol) and DBU (5.0 mmol) in Toluene (3 mL) was transferred into the Schlenk tube. The reaction system was stirred at 100°C for 6 h. After the completion of reaction, saturated ammonium chloride solution (10 mL) was added to the reaction system to quench the reaction. Then CH_2Cl_2 (20 mL) was poured into the reaction mixture. The organic layers were extracted with water (3 × 20 mL). The combined organic layers were dried with anhydrous Na_2SO_4 and filtered and solvent was removed under reduced pressure. The resulting crude product was purified by column chromatography on silica gel using Petroleum ether/EtOAc (20/1) as eluent to afford the product **134** in the yield of 46% as a white solid.

The procedure for the synthesis of 135:[1]

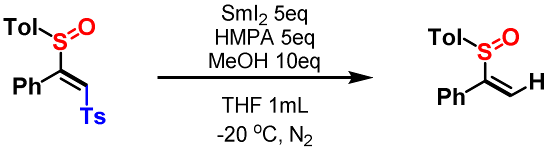

2 **135**, 74%

In the glove box, the solution of compound **2** (396 mg, 1.0 mmol), methanol (405 µL, 10.0 mmol), and hexamethylphosphoric triamide (HMPA, 896 mg, 5.0 mmol) in THF (1 mL) was transferred into the Schlenk tube. The six reaction systems were placed at -20 °C and stirred for 10 min. The solution of samarium iodide in THF (0.1 M, 50 mL) was slowly dropped into the above reaction system. Then, the reaction system was stirred at -20 °C for 2 h. After the completion of reaction, added hydrochloric acid (1 M) to the reaction system to quench the reaction. CH_2Cl_2 (20 mL) was poured into the reaction mixture. The organic layers were extracted

with water (3 × 20 mL). The combined organic layers were dried with anhydrous Na_2SO_4 and filtered and solvent was removed under reduced pressure. The resulting crude product was purified by column chromatography on silica gel using Petroleum ether/EtOAc (8/1) as eluent to afford the **135** with 74% yield.

The procedure for the synthesis of 136: [1]

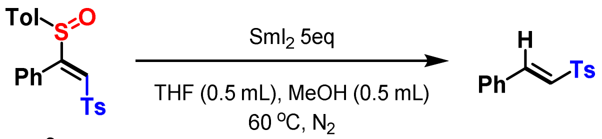

2 **136,** 72%

In the glove box, the solution of compound **2** (396 mg, 1.0 mmol), in methanol/THF (v/v = 1/1, 1 mL) was transferred into the Schlenk tube. The solution of samarium iodide in THF (0.1 M, 50 mL) was slowly dropped into the above reaction system at room temperature. Then, the reaction system was stirred at 60 °C for 3 h. After the completion of reaction, hydrochloric acid (1 M, 10 mL) was added to the reaction system to quench the reaction. CH_2Cl_2 (20 mL) was poured into the reaction mixture. The organic layers were extracted with water (3 × 20 mL). The combined organic layers were dried with anhydrous Na_2SO_4 and filtered and solvent was removed under reduced pressure. The resulting crude product was purified by column chromatography on silica gel using Petroleum ether/EtOAc (8/1) as eluent to afford the **136** with 72% yield.

General procedure for the synthesis of 137-139: [2]

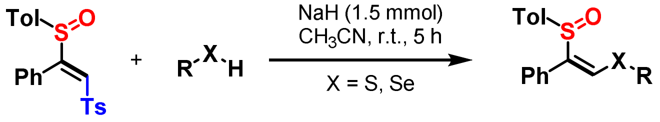

2, 1.0 mmol 1.5 mmol **137-139**

The solution of **2** (396 mg, 1.0 mmol), RXH (1.5 mmol) and NaH (1.5 mmol) in acetonitrile (3 mL) was transferred into the Schlenk tube. The reaction system was stirred at room temperature for 5 h. After the completion of reaction, saturated ammonium chloride solution (10 mL) was added to the reaction system to quench the reaction. Then CH_2Cl_2 (20 mL) was poured into the reaction mixture. The organic layers were extracted with water (3 × 20 mL). The combined organic layers were dried with anhydrous Na_2SO_4 and filtered and solvent was removed under reduced pressure. The resulting crude product was purified by column chromatography on silica gel using Petroleum ether/EtOAc (10/1) as eluent to afford the corresponding products.

General procedure for the synthesis of 140,141 and 143-145:

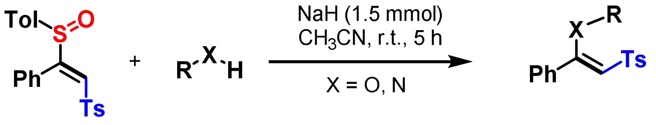

2, 1.0 mmol 1.5 mmol **140, 141, 143-145**

The solution of **2** (396 mg, 1.0 mmol), RXH (1.5 mmol) and NaH (1.5 mmol) in acetonitrile (3 mL) was transferred into the Schlenk tube. The reaction system was stirred at room temperature for 5 h. After the completion of reaction, saturated ammonium chloride solution (10 mL) was added to the reaction system to quench the reaction. Then CH_2Cl_2 (20 mL) was poured into the reaction mixture. The organic layers were extracted with water (3 × 20 mL). The combined organic layers were dried with anhydrous Na_2SO_4 and filtered and solvent was removed under reduced pressure. The resulting crude product was purified by column chromatography on silica gel using Petroleum ether/EtOAc (10/1) as eluent to afford the corresponding products.

The procedure for the synthesis of 142:

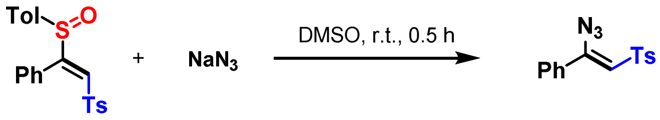

2, 1.0 mmol 10.0 mmol **142**, 62%

The solution of **2** (396 mg, 1.0 mmol) and NaN_3 (10.0 mmol) in DMSO (3 mL) was transferred into the Schlenk tube. The reaction system was stirred at room temperature for 0.5 h. After the completion of reaction, CH_2Cl_2 (20 mL) was poured into the reaction mixture. The organic layers were extracted with water (3 × 20 mL). The combined organic layers were dried with anhydrous Na_2SO_4 and filtered and solvent was removed under reduced pressure. The resulting crude product was purified by column chromatography on silica gel using Petroleum ether/EtOAc (15/1) as eluent to afford **142** with 62% yield.

The procedure for the synthesis of 146:

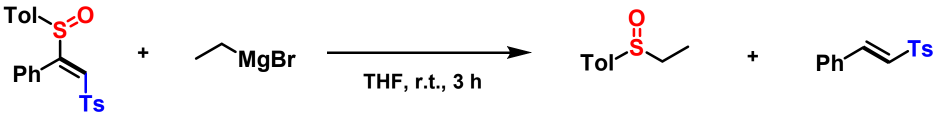

2 **146** **136**

In the glove box, the solution of compound **2** (198 mg, 0.5 mmol), in THF (3 mL) was transferred into the Schlenk tube. The solution of EtMgBr (1.0 mmol) was slowly dropped into the above reaction system at room temperature. Then, the reaction system was stirred at room temperature for 3 h. Saturated ammonium chloride solution (10 mL) was added to the reaction system to quench the reaction. CH_2Cl_2 (20 mL) was poured into the reaction mixture. The organic layers were extracted with water (3 × 20 mL). The combined organic layers were dried with anhydrous Na_2SO_4 and filtered and solvent was removed under reduced pressure. The resulting crude product was purified by column chromatography on silica gel using Petroleum ether/EtOAc (8/1) as eluent to afford the **146** with 87% yield and **136** with 89% yield.

The procedure for the synthesis of 147:

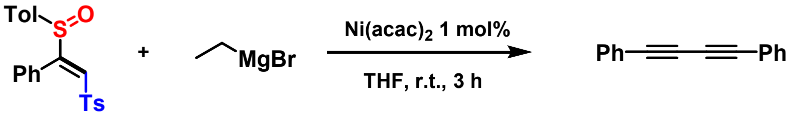

2 **147**

In the glove box, the solution of compound **2** (198 mg, 0.5 mmol) and $Ni(acac)_2$ (1 mol%, 0.005mmol) in THF (3 mL) was transferred into the Schlenk tube. The solution of EtMgBr (1.0 mmol) was slowly dropped into the above reaction system at room temperature. Then, the reaction system was stirred at room temperature for 3 h. Saturated ammonium chloride solution (10 mL) was added to the reaction system to quench the reaction. CH_2Cl_2 (20 mL) was poured into the reaction mixture. The organic layers were extracted with water (3 × 20 mL). The combined organic layers were dried with anhydrous Na_2SO_4 and filtered and solvent was removed under reduced pressure. The resulting crude product was purified by column chromatography on silica gel using Petroleum ether/EtOAc (8/1) as eluent to afford the **147** with 73% yield.

The procedure for the synthesis of 148: [3]

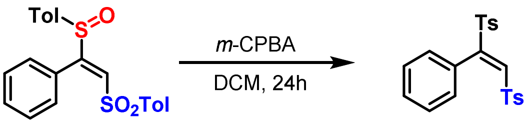

2 **148,** 85%

An oven dried 25 mL round-bottomed flask was charged with **2** (198.2 mg, 0.5 mmol), *m*-CPBA (129.4 mg, 0.75 mmol) and DCM (10 mL).The reaction mixture was stirred at room temperature for 24 h. After completion of the reaction, 20 mL saturated Na_2SO_3 solution was added and the resulting mixture was

extracted with DCM (3 × 20 mL) and the organic layer was combined and dried with anhydrous $NaSO_4$. The combined organic extracts were washed with water, brine, dried over anhydrus $NaSO_4$, and concentrated under reduced pressure. The resulting crude product was purified by column chromatography on silica gel using Petroleum ether/EtOAc/DCM (8:1:1) as eluent to afford the **148** with 85% yield.

The procedure for the synthesis of 149: [4]

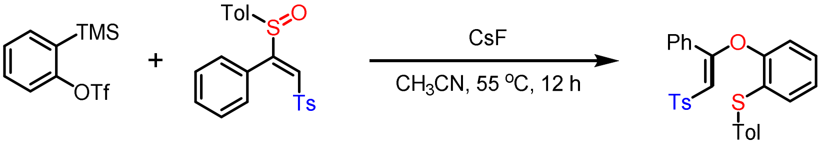

2 **149**

A screw capped reaction vial was charged with **2** (198.3 mg, 0.5 mmol), CsF (455.7 mg, 3 mmol), 2-(trimethylsilyl)phenyl trifluoromethanesulfonate (447.5 mg, 1.5 mmol) acetonitrile (5 mL) in the glove box. The reaction mixture was stirred for 12 h at 55 °C. After completion of the reaction, water was added and the resulting mixture was extracted with EtOAc (2 × 20 mL). The combined organic extracts were washed with water, brine, dried over anhydrus $NaSO_4$, and concentrated under reduced pressure. The resulting crude product was purified by column chromatography on silica gel using Petroleum ether/EtOAc (10:1) as eluent to afford the **149** with 61% yield.

The procedure for the synthesis of 150: [5]

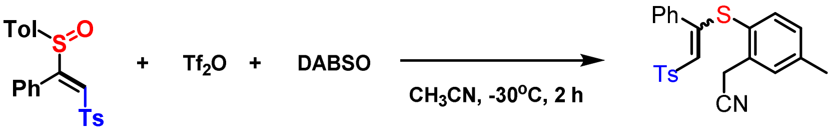

2 **150**

To a solution of **2** (198.9 mg, 0.5 mmol) in MeCN (3 mL) was added Tf_2O (167 μL, 1.0 mmol) under -30°C. After stirring for 10 min, DABSO (300 mg, 2.5 mmol) was added to the mixture under the same temperature. The mixture was then stirred for 2 h. After completion of the reaction, water was added and the resulting mixture was extracted with EtOAc (2 × 20 mL). The combined organic extracts were washed with water, brine, dried over anhydrus $NaSO_4$, and concentrated under reduced pressure. The resulting crude product was purified by column chromatography on silica gel using Petroleum ether/EtOAc (10:1) as eluent to afford the **150** with 90% yield.

V. Mechanistic investigations

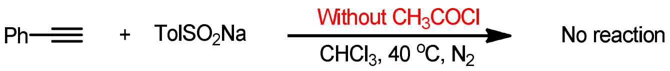

Oven dried Schlenk tube was repeatedly purged with N_2 and added sodium *p*-toluene sulfinate (320 mg, 1.8 mmol). The solution of phenylacetylene (32 mg, 0.3 mmol) in $CHCl_3$ (3 mL) was transferred into the tube. The reaction mixture was stirred at 40 °C for 30 min. Then, CH_2Cl_2 (10 mL) was poured into the reaction mixture. The organic layers were extracted with water (3 × 20 mL). The combined organic layers were dried with anhydrous Na_2SO_4 and filtered and solvent was removed under reduced pressure. The resulting crude product was analyzed by ^{1}H-NMR.

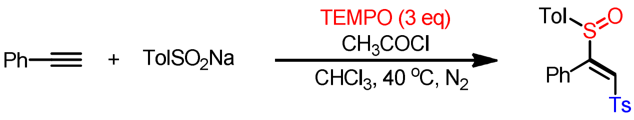

2, 0%

Oven dried Schlenk tube was repeatedly purged with N_2 and added sodium *p*-toluene sulfinate (320 mg, 1.8 mmol) and TEMPO (141 mg, 0.9 mmol). The solution of phenylacetylene (32 mg, 0.3 mmol), CH_3COCl (94.2 mg, 1.2 mmol) in $CHCl_3$ (3 mL) was transferred into the tube. The reaction mixture was stirred at 40 °C for 30 min. Then, CH_2Cl_2 (10 mL) was poured into the reaction mixture. The organic layers were extracted with water (3 × 20 mL). The combined organic layers were dried with anhydrous Na_2SO_4 and filtered and solvent was removed under reduced pressure. The resulting crude product was analyzed by ^{1}H-NMR.

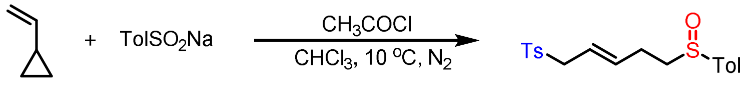

151, 52%

Oven dried Schlenk tube was repeatedly purged with N_2 and added sodium *p*-toluene sulfinate (320 mg, 1.8 mmol). The solution of cyclopropylethylene (0.3 mmol), CH_3COCl (94.2 mg, 1.2 mmol) in $CHCl_3$ (3 mL) was transferred into the tube. The reaction mixture was stirred at 10 °C for 10 h. After the completion of reaction, CH_2Cl_2 (10 mL) was poured into the reaction mixture. The organic layers were extracted with water (3 × 20 mL). The combined organic layers were dried with anhydrous Na_2SO_4 and filtered and solvent was removed under reduced pressure. The resulting crude product was purified by column chromatography on silica gel using petroleum ether/EtOAc/DCM (8/1/1) as eluent to afford **151** with 52% yield.

VI. Analysis of EPR spectra

EPR measurements: EPR spectra were recorded at room temperature on a Bruker EMX nano spectrometer: Mod. Amplitude = 1 G; Time Constan = 120 sec; Sweep time = 120 sec; Power = 0.3 mw. DMPO (5,5-dimethyl-1-pyrroline N-oxide) was employed as the radical trap. EPR spectra (X band, 9.64 GHz, RT) of conditions: In glove box, **1** (0.5 mmol), phenylacetylene (0.5 mmol), and DMPO (1 mmol) in $CHCl_3$ (5 mL) was stirring at room temperature for 5 min. 0.02 mL of this reaction solution was taken out into a small tube and sealed it under N_2. Then, this mixture was used for EPR measurement.

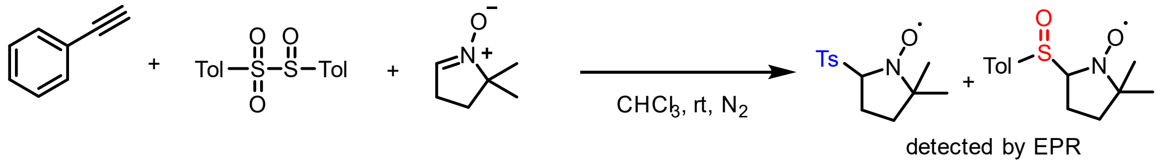

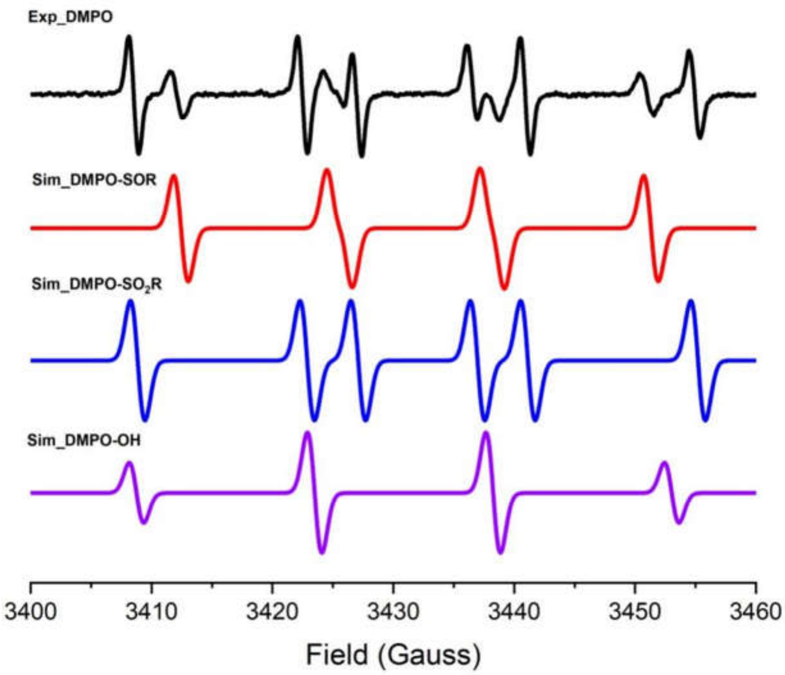

Supplementary Figure 2. EPR spectra of experiments (Exp) and simulation (Sim). The hyperfine parameters of the DMPO adduct radical are: DMPO-OH, A_N = 14.8 G, $A_{H\beta}$ = 14.8 G; DMPO-SOR, A_N = 13.7 G, $A_{H\beta}$ = 12.65 G; DMPO-SO_2R, A_N = 14.1 G, $A_{H\beta}$ = 18.3 G.

Note: Supplementary Figure 2 shows that both the signals of adducts of DMPO with sulfinyl radical and sulfonyl radical could be observed in this reaction system.

VII. X-Ray crystallographic data

Supplementary Table 1. Crystal structure of **1**

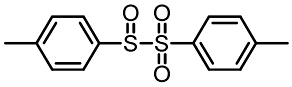

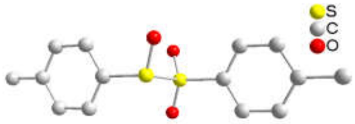

(CCDC No. 1994377)

Empirical formula	$C_{14}H_{14}O_3S_2$
Temperature	173 K
Wavelength	1.54178 Å
Unit cell dimensions	a = 7.1257(4) Å<br>b = 7.3028(4) Å<br>c = 7.5619(4) Å<br>alpha = 82.433(3) deg.<br>beta = 67.200(3) deg.<br>gamma = 71.699(3) deg.
Volume	344.39(3) $Å^3$
Z	1
Calculated density	1.419 g/cm^3
Absorption coefficient	3.519 mm-1
F(000)	154.0
Crystal size	0.21 x 0.20 x 0.15 mm
Theta range for data collection	6.349 to 62.565 deg.
Reflections collected / unique	4525 / 1081 [R(int) = 0.0374]
Data / restraints / parameters	1081/427/150
Goodness-of-fit on F^2	1.060
Final R indices [I>2sigma(I)]	R1 = 0.0532, wR2 = 0.1536
Rindices (all data)	R1 = 0.0565, wR2 = 0.1565

Supplementary Table 2. Crystal structure of **2**

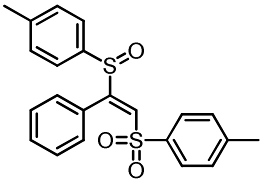

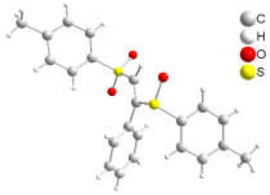

(CCDC No. 1864547)

Empirical formula	$C_{22}H_{20}O_3S_2$
Temperature	298(2)
Wavelength	1.40700 Å
Unit cell dimensions	a = 10.1993(15) Å<br>b = 8.4606(13) Å<br>c = 22.825(3) Å<br>alpha = 90 deg.<br>beta = 96.224(3) deg.<br>gamma = 90 deg.
Volume	1958.0(5) Å ^{3}
Z	4
Calculated density	1.658 Mg/m^3
Absorption coefficient	0.942 mm^{-1}
F(000)	992.0
Crystal size	0.21 x 0.30 x 0.15 mm
Theta range for data collection	3.59 to 52.788 deg.
Reflections collected / unique	10770 / 4003 [R(int) = 0.0662]
Data / restraints / parameters	4003 / 0 / 244
Goodness-of-fit on F^2	1.059
Final R indices [I>2sigma(I)]	R1 = 0.0590, wR2 = 0.1099
Rindices (all data)	R1 = 0.1144, wR2 = 0.1306

Supplementary Table 3. Crystal structure of **49**

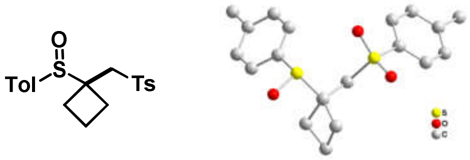

(CCDC No. 2003800)

Empirical formula	$C_{19}H_{22}O_3S_2$
Temperature	293 K
Wavelength	0.71073 Å
Unit cell dimensions	a = 7.6658(7) Å<br>b = 11.2719(8) Å<br>c = 21.6538(16) Å<br>alpha = 90 deg.<br>beta = 90 deg.<br>gamma = 90 deg.
Volume	1871.1(3) $Å^3$
Z	4
Calculated density	1.287 g/cm3
Absorption coefficient	0.298 mm-1
F(000)	768.0
Crystal size	0.20 x 0.14 x 0.15 mm
Theta range for data collection	3.349 to 29.291deg.
Reflections collected / unique	8891 / 4029 [R(int) = 0.0395]
Data / restraints / parameters	4003 / 0 / 219
Goodness-of-fit on F^2	1.011
Final R indices [I>2sigma(I)]	R1 = 0.0620, wR2 = 0.1411
Rindices (all data)	R1 = 0.1115, wR2 = 0.1735

Supplementary Table 4. Crystal structure of **79**

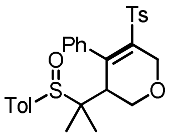 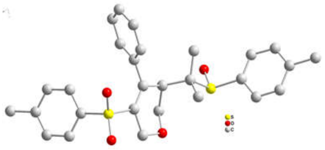 (CCDC No. 2003798)	
Empirical formula	$C_{28}H_{30}O_4S_2$
Temperature	293 K
Wavelength	0.71073 Å
Unit cell dimensions	a = 9.8456(9) Å<br>b = 10.6822(8) Å<br>c = 13.2774(11) Å<br>alpha = 84.982(7) deg.<br>beta = 70.901(8) deg.<br>gamma = 74.808(7) deg.
Volume	1273.4(2) $Å^3$
Z	2
Calculated density	1.290 g/cm3
Absorption coefficient	0.241 mm-1
F(000)	524.0
Crystal size	0.15 x 0.13 x 0.15 mm
Theta range for data collection	3.351 to 29.221 deg.
Reflections collected / unique	9360 / 5813 [R(int) = 0.0236]
Data / restraints / parameters	5813 / 0 / 311
Goodness-of-fit on F^2	1.029
Final R indices [I>2sigma(I)]	R1 = 0.0596, wR2 = 0.1354
Rindices (all data)	R1 = 0.1000, wR2 = 0.1636

Supplementary Table 5. Crystal structure of **91**

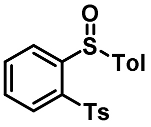

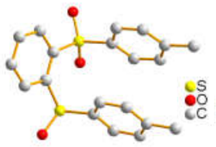

(CCDC No. 2058384)

Empirical formula	$C_{20}H_{18}O_3S_2$
Temperature	293 K
Wavelength	0.71073 Å
Unit cell dimensions	a = 11.655(3) Å<br>b = 14.655(3) Å<br>c = 20.785(5) Å<br>alpha = 90 deg.<br>beta = 90 deg.<br>gamma = 90 deg.
Volume	3550.1(14) $Å^3$
Z	8
Calculated density	1.319 g/cm3
Absorption coefficient	0.314 mm-1
F(000)	1409.0
Crystal size	0.18 x 0.21 x 0.15 mm
Theta range for data collection	6.804 to 59.348 deg.
Reflections collected / unique	11969 / 4275 [R_{int}= 0.0615, R_{sigma}= 0.0843]
Data / restraints / parameters	4275/0/228
Goodness-of-fit on F^2	1.038
Final R indices [I>2sigma(I)]	R_1= 0.0784, wR_2= 0.2150
Rindices (all data)	R_1= 0.1556, wR_2= 0.2653

Supplementary Table 6. Crystal structure of **119**

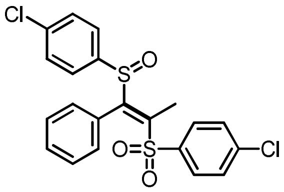

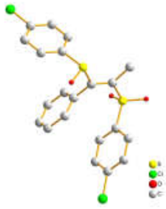

(**CCDC No. 1907354**)

Empirical formula	$C_{21}H_{16}Cl_2O_3S_2$
Temperature	273.15 K
Wavelength	1.54178 Å
Unit cell dimensions	a = 10.0347(6) Å<br>b = 9.9087(6) Å<br>c = 21.0829(12) Å<br>alpha = 90 deg.<br>beta = 98.721(3) deg.<br>gamma = 90 deg.
Volume	2072.1(2) Å ^{3}
Z	4
Calculated density	1.447 g/cm3
Absorption coefficient	4.871 mm-1
F(000)	928.0
Crystal size	0.15 × 0.12 × 0.1 mm
Theta range for data collection	8.486 to 130.29 deg.
Reflections collected / unique	18069 / 3499 [R_{int} = 0.0416, R_{sigma} = 0.0305]
Data / restraints / parameters	3499/0/254
Goodness-of-fit on F^2	1.045
Final R indices [I>2sigma(I)]	R_1 = 0.0482, wR_2 = 0.1083
Rindices (all data)	R_1 = 0.0585, wR_2 = 0.1146

VIII. Density functional theory calculations

Computational methods: All of the density functional theory (DFT) calculations were implemented with the Gaussian 09[6] package. Geometry optimizations and frequencies were calculated with the B3LYP[7,8] density functional and 6-31+G(d,p) basis set for all atoms in conjunction with the SMD implicit solvation model to account for the solvation effects of chloroform. Frequency calculations were examined for all intermediates and transition structures to determine minima (zero imaginary frequencies) or transition structures (only one imaginary frequency), respectively. Intrinsic reaction coordinate (IRC)[9,10] was used to confirmed whether transition states connected the reactant and product. Dispersion effects were also considered by performing geometry optimization and frequency calculations by using the B3LYP-D3 functional[11]. The key 3D structures were prepared using the CYLview visualization program[12].

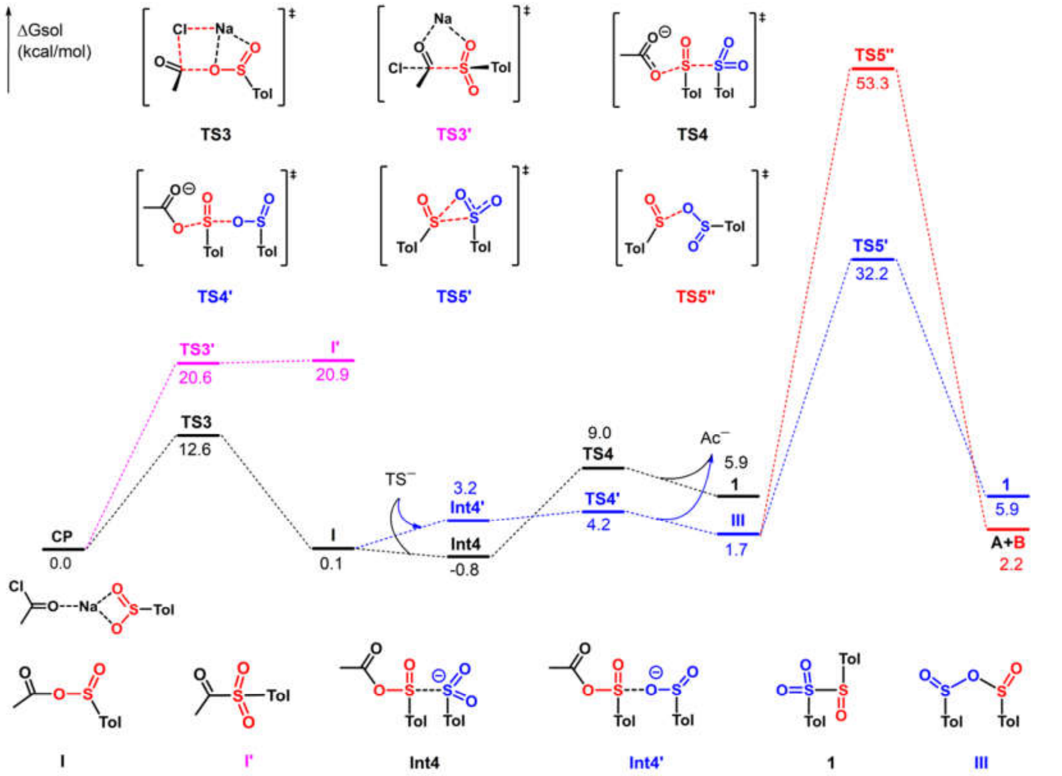

Supplementary Figure 3. Free energy profiles for the conversion from $TolSO_2Na$ and CH_3COCl into sulfinyl sulfones (**1**). The energies are in kcal/mol and represent the relative free energies calculated with the SMD-B3LYP/6-31+G(d,p) level in chloroform.

As shown in Supplementary Figure 3, the reaction from the complex $TolSO_2Na$ and CH_3COCl (**CP**) into sulfinyl sulfones (**1**) undergoes transition states (**TS3** and **TS4**), including anion exchange, nucleophilic attack of Ts− (negative charge on sulfur atom), for which the energy barriers are 12.6, 9.8 kcal/mol, suggesting the conversion from **CP** to **1** proceeds fast and smoothly under the standard condition. In the anion exchang step, the energy barrier of the Ts- tautomeric (negative charge on the sulfur atom) nucleophilic attack on CH_3COCl is 8 kcal/mol higher than that of **TS3** (Pink line)**.** Futhermore, the path of nucleophilic attack of Ts− tautomers (O=S-O−, negative charge on oxygen atom) for the reaction from **I** to **1** was also calculated, and the free energy profile is drawn in blue line. The reaction from **I** easily undergoes nucleophilic attack of Ts− (negative charge on oxygen atom) *via* **TS4′** to form sulfinic anhydride (**III**). Subsequently, the homolysis of sulfinic anhydride (**III**) or isomerizes to sulfinyl sulfone (**1**) through **TS5″** (red line) and **TS5′** (blue line) were taken into account and are excluded due to the high energy barriers ($\Delta\Delta G$ = 51.6 and 30.5 kcal.mol^{-1}). Calculation results show that nucleophilic attack of Ts− tautomers (O=S-O−, negative charge on oxygen atom) is impossible.

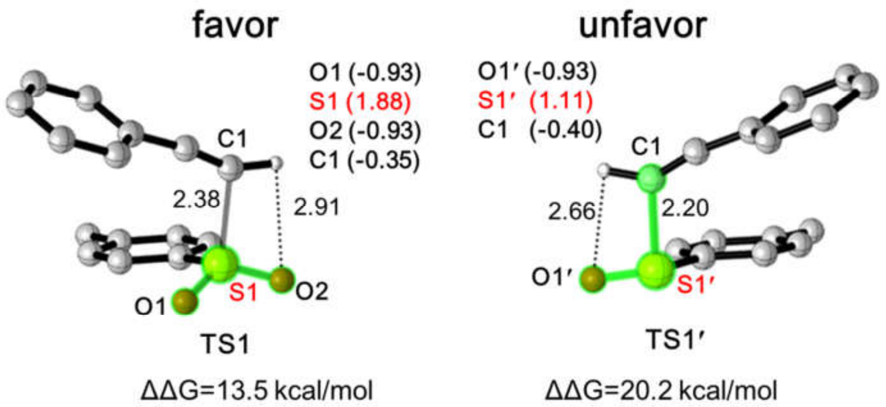

Supplementary Figure 4. Optimized geometries and Gibbs energy barriers for radical addition transition state **TS1** and **TS1′**. The values in parentheses are corresponding NPA charge of certain atoms.

We have performed the Natural Population Analysis (NPA) of the key transition states to study the electronic effect (Supplementary Figure 4). NPA charge shows that the charge on S1 in **TS1** is 1.88 e whereas the charge on S1′ is 1.11 e in **TS1′**, confirming the relatively higher electrophilicity of sulfur S1 in the transition state **TS1**. NPA charge demonstrates that the strong electrophilicity of sulfur S1 in forming S-C bond of transition state **TS1** might result in a lower energy barrier.

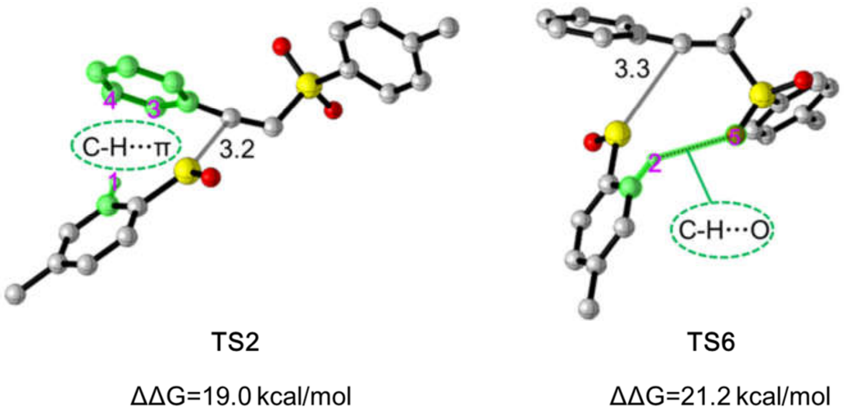

Supplementary Figure 5. Optimized structures of transition state **TS2** and **TS6**. The selected bond lengths are given in angstroms. Some hydrogen atoms are hidden for clarity.

Supplementary Figure 5 shows the structures of two key transition states, **TS2** and **TS6**. In **TS2**, we identified C−H…π interaction, where the π system comes from the benzene ring in the phenylacetylene and the C-H unit comes from the sulfinyl radical **B**. Meanwhile, in **TS6**, we identified one C−H…O interaction between the benzene ring and the sulfonyl radical **A**. These interactions stabilize transition states **TS2** and **TS6**, and shorten the reaction site. However, in **TS2**, the C-S bond distance of the sulfinyl radical **B** to the alkenyl radical was 3.2 Å, which was 0.1 Å shorter than that in **TS6**. Therefore, the elecrostaction interaction in **TS2** is stronger, resulting in lower energy barrier.

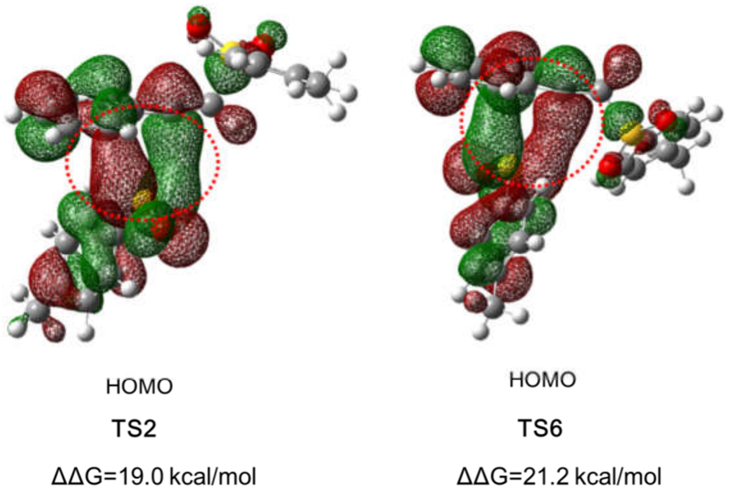

Supplementary Figure 6. Diagrams of the HOMOs of **TS2** and **TS6**. The values are calculated energy barriers (in kcal/mol).

This fact can be understood by analyzing the natures of the transition states involved in these different processes. Supplementary Figure 6 compares the calculated patterns of the higest occupied molecular orbitals (HOMOs) of transition states **TS2** and **TS6**. The bonding interaction between the S and C atom in the **TS2** is larger than that of **TS6**, confirming the former expected to be the suitable transition state.

Supplementary Figure 7. DFT calculations and graphical reprentation of the proposed radical mechanistic pathway. The energies are in kcal/mol and represent the relative free energies calculated at the SMD-B3LYP-D3/6-31+G(d,p) level in chloroform.

Cartesian coordinates of the SMD-B3LYP/6-31+G(d,p) computed structures

CP Charge = 0, Multiplicity = 1
Zero-point correction= 0.174365 (Hartree/Particle)
Thermal correction to Energy= 0.192197
Thermal co rrection to Enthalpy= 0.193141
Thermal correction to Gibbs Free Energy= 0.123577
Sum of electronic and zero-point Energies= -1595.278279
Sum of electronic and thermal Energies= -1595.260447
Sum of electronic and thermal Enthalpies= -1595.259502
Sum of electronic and thermal Free Energies= -1595.329066

C	-3.99253800	0.74250600	-1.17025900
C	-2.94151100	-0.17690700	-1.20709300
C	-2.39867900	-0.65827100	-0.01202100
C	-2.91764300	-0.23215100	1.21311900
C	-3.96951800	0.68827300	1.23821900
C	-4.52464400	1.18995600	0.05045200
H	-4.40176700	1.12266400	-2.10365600
H	-2.53240400	-0.50485200	-2.15810000
H	-2.49087700	-0.60282700	2.14046600
H	-4.36036000	1.02580300	2.19540500
C	-5.68276800	2.15862400	0.08069700
H	-5.73196000	2.69519200	1.03328500
H	-5.60970500	2.89766000	-0.72426300
H	-6.63746700	1.63218000	-0.04890000
S	-1.07189500	-1.92786300	-0.05341500
O	-0.24856300	-1.50683700	-1.30666700
O	-0.22543200	-1.56345700	1.20208600
Na	1.44560500	-0.65702400	-0.04722500
C	4.89909300	2.46081500	-0.12857500
H	4.03737300	3.12734200	-0.19106100
H	5.51534700	2.70850100	0.74124000
H	5.52785300	2.56740500	-1.01915400
C	4.42861800	1.05067400	-0.02622900
O	3.29422700	0.65686100	-0.05357700
Cl	5.76284200	-0.15468500	0.14966200

TS3 Charge = 0, Multiplicity = 1
Zero-point correction= 0.174095 (Hartree/Particle)
Thermal correction to Energy= 0.190963
Thermal correction to Enthalpy= 0.191908
Thermal correction to Gibbs Free Energy= 0.127260
Sum of electronic and zero-point Energies= -1595.262210
Sum of electronic and thermal Energies= -1595.245341
Sum of electronic and thermal Enthalpies= -1595.244397
Sum of electronic and thermal Free Energies= -1595.309044

C	-3.71522300	-1.00528000	-0.08843400
C	-2.38204600	-1.23041700	0.27247000
C	-1.52070000	-0.14432500	0.41534600
C	-1.98342400	1.16241600	0.22345700

C	-3.31424600	1.37131300	-0.13350900
C	-4.20324900	0.29260600	-0.29417400
H	-4.38466700	-1.85359200	-0.20727600
H	-2.01209200	-2.23760200	0.43682200
H	-1.31245800	2.00949300	0.33543000
H	-3.66991100	2.38686000	-0.29064500
C	-5.64459100	0.53737600	-0.67048600
H	-5.71993500	1.11139900	-1.60157200
H	-6.18619900	-0.40282900	-0.80795600
H	-6.16271400	1.11404400	0.10538000
S	0.19909800	-0.41091300	0.95926300
O	0.41035200	-1.92894800	0.79460800
O	0.97523500	0.28604600	-0.24999700
Na	1.81628200	-1.77891700	-0.99583200
C	2.85730800	1.58565400	1.32550700
H	2.78254100	0.66981800	1.90519700
H	3.84891700	2.02743800	1.46755000
H	2.08972300	2.30397200	1.63095400
C	2.69399500	1.35189500	-0.13134100
O	2.63837200	2.01357000	-1.09423700
Cl	4.03785400	-0.43994600	-0.49905600

I Charge = 0, Multiplicity = 1
Zero-point correction= 0.173563 (Hartree/Particle)
Thermal correction to Energy= 0.187109
Thermal correction to Enthalpy= 0.188053
Thermal correction to Gibbs Free Energy= 0.131587
Sum of electronic and zero-point Energies= -972.686152
Sum of electronic and thermal Energies= -972.672607
Sum of electronic and thermal Enthalpies= -972.671663
Sum of electronic and thermal Free Energies= -972.728129

C	-2.72655100	0.70581600	0.84513000
C	-1.38771500	1.05651600	0.66438500
C	-0.63432200	0.36275300	-0.28173100
C	-1.19613500	-0.65547200	-1.06024900
C	-2.53351600	-0.99149700	-0.86425600
C	-3.31862600	-0.31885300	0.09003800
H	-3.32050100	1.23846400	1.58272600
H	-0.93474300	1.85526700	1.24303400
H	-0.60270500	-1.18273100	-1.80278900
H	-2.97746800	-1.78365800	-1.46152000
C	-4.76497000	-0.69663100	0.28850600
H	-5.31971300	-0.64240100	-0.65521900
H	-4.85335900	-1.72620400	0.65569100
H	-5.25508800	-0.03777800	1.01032900
S	1.07738800	0.82489900	-0.58051100
O	1.33816900	2.08547500	0.18237100
O	1.68811100	-0.52484500	0.39990900
C	3.97382500	-0.07213200	-0.44681100
H	3.98891400	0.96807200	-0.10557900
H	4.97220100	-0.49828200	-0.34602000
H	3.68713800	-0.07912000	-1.50402500

C	3.01493100	-0.88726300	0.37739800
O	3.32567100	-1.85180900	1.04235700

Int4 Charge = -1, Multiplicity = 1
Zero-point correction= 0.298553 (Hartree/Particle)
Thermal correction to Energy= 0.324105
Thermal correction to Enthalpy= 0.325049
Thermal correction to Gibbs Free Energy= 0.236367
Sum of electronic and zero-point Energies= -1792.287089
Sum of electronic and thermal Energies= -1792.261537
Sum of electronic and thermal Enthalpies= -1792.260592
Sum of electronic and thermal Free Energies= -1792.349275

C	1.27289700	2.85861100	1.27522100
C	1.66666500	1.53002500	1.12514300
C	1.43299800	0.88835800	-0.09420600
C	0.79967200	1.54427100	-1.14836700
C	0.40921000	2.87356400	-0.97961600
C	0.64026900	3.55090300	0.22832800
H	1.44973200	3.36242300	2.22195200
H	2.13896800	1.00474400	1.95098300
H	0.61072700	1.01850400	-2.07902200
H	-0.08550200	3.39110300	-1.79755000
C	0.18806600	4.97820000	0.41536200
H	0.91173500	5.55318800	1.00266000
H	0.04668100	5.48232800	-0.54543700
H	-0.76822800	5.01581500	0.95286200
O	1.40495600	-1.31237200	-1.58292800
S	1.94089400	-0.83603600	-0.27188100
O	3.65495200	-0.29614800	-0.62880100
C	4.59381000	-1.26781300	-0.56365900
C	5.96584300	-0.72510900	-0.87375900
H	6.20643900	0.09984900	-0.19610300
H	6.70628900	-1.51989400	-0.77388000
H	5.98180800	-0.33049100	-1.89507900
O	4.34436600	-2.42942300	-0.29450600
S	-0.98472000	-1.47195700	1.48398300
O	-1.22729600	-2.97147200	1.72713500
O	-1.15674300	-0.62352400	2.75605100
C	-2.47469000	-0.98836700	0.49084900
C	-3.19952900	0.16095300	0.80306900
C	-2.83838500	-1.75308200	-0.62332600
C	-4.29692200	0.53314900	0.01616900
H	-2.91081100	0.74825100	1.67024600
C	-3.93007200	-1.37456600	-1.40640200
H	-2.27786300	-2.65098900	-0.86909600
C	-4.67950300	-0.22473300	-1.09955800
H	-4.86377000	1.42522600	0.27557300
H	-4.20865700	-1.98145000	-2.26609000
C	-5.86253600	0.17343600	-1.95228800
H	-6.62945500	-0.61099300	-1.96275900
H	-6.32950500	1.09137800	-1.58187700
H	-5.56600200	0.34558700	-2.99438000

TS4 Charge = -1, Multiplicity = 1
Zero-point correction= 0.298839 (Hartree/Particle)
Thermal correction to Energy= 0.323574
Thermal correction to Enthalpy= 0.324518
Thermal correction to Gibbs Free Energy= 0.239823
Sum of electronic and zero-point Energies= -1792.274638
Sum of electronic and thermal Energies= -1792.249903
Sum of electronic and thermal Enthalpies= -1792.248959
Sum of electronic and thermal Free Energies= -1792.333654

C	1.49067300	2.93929500	0.98877100
C	1.61289000	1.55659000	0.88808400
C	1.00637000	0.89607400	-0.18786900
C	0.30321400	1.60632800	-1.16207800
C	0.20201200	2.99502500	-1.05206300
C	0.78907300	3.68205100	0.02118800
H	1.96016600	3.45435400	1.82328400
H	2.18650100	1.00042500	1.62110400
H	-0.14869300	1.07599300	-1.99433600
H	-0.33916000	3.55163800	-1.81305800
C	0.69769600	5.18431600	0.13061900
H	0.02625600	5.60103900	-0.62584300
H	0.33173300	5.48993900	1.11771400
H	1.68316300	5.64779300	-0.00353200
O	0.46672400	-1.27244600	-1.60326900
S	1.16489800	-0.89438100	-0.32175700
O	3.74103300	-1.90172800	-1.48793500
C	4.40635900	-1.24693100	-0.63643200
C	5.85248800	-1.70965300	-0.38213700
H	6.43237000	-1.62724700	-1.30931600
H	6.33828600	-1.11649000	0.39748100
H	5.85744300	-2.76641300	-0.09041800
O	3.98483300	-0.24807100	0.02021000
S	-0.60149200	-1.37527200	1.34637600
O	-0.55435300	-2.86011500	1.50382600
O	-0.45930700	-0.52378000	2.56548000
C	-2.17667300	-0.96645700	0.56929900
C	-2.78563100	0.26151800	0.84605500
C	-2.73936400	-1.86225300	-0.34430700
C	-3.98970600	0.57573400	0.21686200
H	-2.33125500	0.94908600	1.55136000
C	-3.94210000	-1.52691000	-0.96752600
H	-2.25185500	-2.80754400	-0.55645300
C	-4.58700200	-0.30964700	-0.69660300
H	-4.47320200	1.52394500	0.43806300
H	-4.38389900	-2.22247700	-1.67615400
C	-5.90306700	0.03264400	-1.35084300
H	-6.04263100	-0.52344400	-2.28258600
H	-6.74327500	-0.21565900	-0.68940100
H	-5.97126900	1.10227500	-1.57443700

1 Charge = 0, Multiplicity = 1

Zero-point correction= 0.249747 (Hartree/Particle)
Thermal correction to Energy= 0.269048
Thermal correction to Enthalpy= 0.269992
Thermal correction to Gibbs Free Energy= 0.199270
Sum of electronic and zero-point Energies= -1563.702344
Sum of electronic and thermal Energies= -1563.683043
Sum of electronic and thermal Enthalpies= -1563.682099
Sum of electronic and thermal Free Energies= -1563.752821

C	4.20801000	-1.09185500	-0.55327800
C	2.88712600	-0.92875600	-0.97073600
C	2.22234500	0.26413700	-0.66252400
C	2.86281200	1.29281400	0.03732300
C	4.18406800	1.11262200	0.43662500
C	4.87592000	-0.07981300	0.15482300
H	4.72920100	-2.01550100	-0.78886400
H	2.39016900	-1.71899900	-1.52650200
H	2.33194700	2.21395600	0.25462500
H	4.68900700	1.90858400	0.97770100
C	6.29683200	-0.26628900	0.62247100
H	6.89138200	0.63866900	0.45882100
H	6.32308500	-0.48081700	1.69853900
H	6.78422800	-1.09800900	0.10611600
O	0.18646200	1.94605000	-1.11527700
S	0.53712400	0.47918100	-1.23646200
S	-0.45117400	-0.53464800	0.66944500
O	-0.11686100	-1.97497000	0.55680900
O	-0.04709800	0.24135100	1.86461700
C	-2.20506600	-0.31218800	0.40021700
C	-2.83698100	0.82325400	0.91898600
C	-2.89885600	-1.24773400	-0.37332600
C	-4.19602500	1.00333500	0.67146400
H	-2.28050600	1.54290500	1.50921500
C	-4.25868400	-1.04586700	-0.60667000
H	-2.39356600	-2.12115800	-0.77187900
C	-4.92793100	0.07597800	-0.09028400
H	-4.69635200	1.87884300	1.07650900
H	-4.80795500	-1.77336700	-1.19779500
C	-6.40374900	0.27343200	-0.32531900
H	-6.76040500	-0.33160700	-1.16353800
H	-6.97807600	-0.01700600	0.56373300
H	-6.63631000	1.32311100	-0.53222800

A Charge = 0, Multiplicity = 2
Zero-point correction= 0.125274 (Hartree/Particle)
Thermal correction to Energy= 0.133987
Thermal correction to Enthalpy= 0.134931
Thermal correction to Gibbs Free Energy= 0.090057
Sum of electronic and zero-point Energies= -819.434372
Sum of electronic and thermal Energies= -819.425659
Sum of electronic and thermal Enthalpies= -819.424714
Sum of electronic and thermal Free Energies= -819.469589

S	-2.14136700	-0.00175100	-0.22812000
O	-2.63349300	1.26442300	0.27482100
O	-2.62959800	-1.26760500	0.27958300
C	-0.36650400	0.00099000	-0.09543600
C	0.30897800	-1.21134000	-0.07926500
C	0.30417700	1.21329800	-0.07689800
C	1.69109400	-1.19668200	-0.01807500
H	-0.24649300	-2.13935000	-0.09552800
C	1.68894200	1.20331400	-0.01535200
H	-0.25360300	2.13996000	-0.09167700
C	2.39909400	0.00545300	0.01459300
H	2.23347600	-2.13423600	0.00686000
H	2.22700800	2.14280700	0.01144700
C	3.89858900	-0.00254000	0.10206500
H	4.31406900	0.97770500	-0.12495400
H	4.21955600	-0.27979800	1.10823700
H	4.32637100	-0.72856900	-0.58948600

B Charge = 0, Multiplicity = 2
Zero-point correction= 0.122445 (Hartree/Particle)
Thermal correction to Energy= 0.131098
Thermal correction to Enthalpy= 0.132042
Thermal correction to Gibbs Free Energy= 0.086137
Sum of electronic and zero-point Energies= -744.196673
Sum of electronic and thermal Energies= -744.188020
Sum of electronic and thermal Enthalpies= -744.187076
Sum of electronic and thermal Free Energies= -744.232981

C	-1.53511100	1.18440100	-0.00897600
C	-0.16040300	1.33847600	-0.00274800
C	0.65617300	0.20953700	0.00013000
C	0.09928700	-1.06472300	-0.00490400
C	-1.27814000	-1.19918300	-0.01136900
C	-2.11569700	-0.08384300	-0.01088700
H	-2.17164500	2.06114700	-0.01498600
H	0.27994200	2.32923800	-0.00361200
H	0.75433400	-1.92636500	-0.00728500
H	-1.71640400	-2.19021200	-0.01927200
C	-3.60929000	-0.24286800	0.01477300
H	-3.91362700	-1.18222500	-0.44524000
H	-3.97668800	-0.24348300	1.04355200
H	-4.10208700	0.57479000	-0.51040200
O	3.02326200	-0.91065000	0.00477800
S	2.39494700	0.43822000	0.00393100

Int1 Charge = 0, Multiplicity = 2
Zero-point correction= 0.235570 (Hartree/Particle)
Thermal correction to Energy= 0.253875
Thermal correction to Enthalpy= 0.254819
Thermal correction to Gibbs Free Energy= 0.182477
Sum of electronic and zero-point Energies= -1127.753204
Sum of electronic and thermal Energies= -1127.734899
Sum of electronic and thermal Enthalpies= -1127.733955

Sum of electronic and thermal Free Energies= -1127.806297

C	-4.14226700	0.80403900	-0.79578000
C	-3.65380700	-0.46152600	-0.47200900
C	-2.40301600	-0.55363200	0.14325800
C	-1.66096400	0.58087600	0.48646300
C	-2.17204600	1.83303000	0.15270900
C	-3.41438800	1.96673600	-0.49289600
H	-5.10502000	0.88781500	-1.29207000
H	-4.22296400	-1.35319900	-0.71143900
H	-0.70074400	0.48904700	0.98208500
H	-1.59541400	2.72100800	0.39697800
C	-3.93738800	3.32949200	-0.86845200
H	-3.75285300	4.05918300	-0.07334000
H	-3.43306200	3.69874300	-1.77067800
H	-5.01119400	3.30507400	-1.07432200
S	-1.78337200	-2.18224700	0.62315200
O	-2.41648200	-3.20702400	-0.26234900
O	-0.29528600	-2.10429400	0.74223200
C	3.49093800	-1.09451000	-1.25856100
C	2.94045300	-0.56896800	-0.08949900
C	3.51279900	0.57099000	0.51037000
C	4.64348200	1.16828700	-0.08279700
C	5.18641700	0.63404700	-1.25125900
C	4.61335800	-0.49739200	-1.84205100
H	3.04185800	-1.97318600	-1.71261000
H	2.06989100	-1.03448700	0.36161300
H	5.08761900	2.04544500	0.37761400
H	6.05849100	1.10093300	-1.70028300
H	5.03926900	-0.91134300	-2.75164100
C	2.95537800	1.11427500	1.71313700
C	2.48668500	1.57391600	2.73416600
H	2.07840400	1.97349200	3.63780500

TS1 Charge = 0, Multiplicity = 2
Zero-point correction= 0.235613 (Hartree/Particle)
Thermal correction to Energy= 0.252558
Thermal correction to Enthalpy= 0.253502
Thermal correction to Gibbs Free Energy= 0.187048
Sum of electronic and zero-point Energies= -1127.745594
Sum of electronic and thermal Energies= -1127.728649
Sum of electronic and thermal Enthalpies= -1127.727705
Sum of electronic and thermal Free Energies= -1127.794159

C	-2.42326200	1.72763200	0.86139600
C	-1.57338700	0.62411100	0.95557700
C	-1.87160100	-0.52096900	0.21382600
C	-2.98082000	-0.56861600	-0.63647700
C	-3.81687300	0.54404400	-0.71443900
C	-3.55325500	1.70809700	0.02871500
H	-2.20277500	2.61674500	1.44611700
H	-0.70643000	0.64931300	1.60737700
H	-3.19667400	-1.46362200	-1.21040000

H	-4.68909100	0.50795900	-1.36235100
C	-4.48008800	2.89487600	-0.05482000
H	-5.42914000	2.68365800	0.45389200
H	-4.04073300	3.77970000	0.41412000
H	-4.71987000	3.14006200	-1.09524600
S	-0.74169400	-1.92952600	0.27664700
O	-0.06925300	-1.90764600	1.60813100
O	-1.50566600	-3.14464600	-0.14022500
C	4.81712000	0.38963100	0.70868400
C	3.85001800	-0.47906000	0.21305100
C	2.79966700	0.01832800	-0.59770900
C	2.75249900	1.40244600	-0.89836100
C	3.72825700	2.25742700	-0.39601100
C	4.76131800	1.75671400	0.40733000
H	5.61689400	0.00241800	1.33296500
H	3.88605200	-1.53843500	0.44547800
H	1.95074100	1.78469200	-1.52213100
H	3.68621600	3.31710400	-0.62998000
H	5.51986600	2.42939400	0.79676700
C	1.82291900	-0.85909700	-1.11431600
C	0.89360200	-1.61776600	-1.42817500
H	0.43472100	-2.28502000	-2.13582000

II Charge = 0, Multiplicity = 2
Zero-point correction= 0.237528 (Hartree/Particle)
Thermal correction to Energy= 0.254310
Thermal correction to Enthalpy= 0.255254
Thermal correction to Gibbs Free Energy= 0.188731
Sum of electronic and zero-point Energies= -1127.757006
Sum of electronic and thermal Energies= -1127.740224
Sum of electronic and thermal Enthalpies= -1127.739280
Sum of electronic and thermal Free Energies= -1127.805803

C	-2.99106100	1.62356200	0.69957900
C	-1.89687200	0.76963400	0.83321400
C	-1.91893000	-0.46642400	0.18080700
C	-3.00957900	-0.85157400	-0.60390000
C	-4.09328300	0.01824100	-0.72714200
C	-4.10508200	1.26353300	-0.07769400
H	-2.98112200	2.58178400	1.21211200
H	-1.04561700	1.05267700	1.44343600
H	-3.01793600	-1.81726300	-1.09839500
H	-4.94589600	-0.27890600	-1.33196200
C	-5.30048400	2.17670400	-0.18408900
H	-6.02597600	1.95392900	0.60895200
H	-5.01305500	3.22720800	-0.07882700
H	-5.81535800	2.05225300	-1.14165600
S	-0.50437700	-1.56212400	0.32646000
O	0.20419600	-1.24356600	1.58379600
O	-0.96872500	-2.94545000	0.07006300
C	5.21106000	0.04409400	0.18049800
C	4.04801200	-0.61501800	-0.18821900
C	2.87108600	0.13260700	-0.51148400

C	2.92995800	1.56129600	-0.44848700
C	4.10662900	2.19391400	-0.07641600
C	5.25307700	1.44668400	0.23968600
H	6.09644400	-0.53495400	0.42713900
H	4.01365400	-1.69872200	-0.23168600
H	2.04382500	2.13805900	-0.69323100
H	4.13717800	3.27875800	-0.02866600
H	6.16929700	1.95217100	0.52932600
C	1.71367300	-0.50854400	-0.90360900
C	0.58332400	-1.11448400	-1.08545700
H	0.16164300	-1.44624000	-2.03450100

Int2 Charge = 0, Multiplicity = 3
Zero-point correction= 0.359376 (Hartree/Particle)
Thermal correction to Energy= 0.387111
Thermal correction to Enthalpy= 0.388055
Thermal correction to Gibbs Free Energy= 0.291455
Sum of electronic and zero-point Energies= -1872.008959
Sum of electronic and thermal Energies= -1871.981223
Sum of electronic and thermal Enthalpies= -1871.980279
Sum of electronic and thermal Free Energies= -1872.076879

C	4.55655400	-2.24131200	1.47495100
C	4.18951100	-1.20675700	0.61564900
C	3.39261300	-1.49949700	-0.49529000
C	2.95943700	-2.80272300	-0.75175400
C	3.33815400	-3.82438100	0.11999200
C	4.13834500	-3.56297900	1.24374400
H	5.17812600	-2.01845700	2.33822900
H	4.52307800	-0.19085400	0.80019200
H	2.34594500	-3.01603700	-1.62068700
H	3.00592000	-4.84007400	-0.07722900
C	4.56145200	-4.67939700	2.16587100
H	5.52015400	-5.10608200	1.84395200
H	4.69113600	-4.32277800	3.19233000
H	3.82738400	-5.49080200	2.17203200
S	2.87643900	-0.16672600	-1.58280500
O	3.90570100	0.89433300	-1.54469200
O	2.48441800	-0.76201300	-2.88171700
C	1.18934100	5.37240900	-0.12519000
C	1.10446600	4.08953600	-0.64490300
C	1.39651600	2.95812500	0.18213600
C	1.76940400	3.18733900	1.54547200
C	1.84686400	4.48126100	2.03825100
C	1.55855800	5.58132700	1.21387400
H	0.96851600	6.22238300	-0.76458300
H	0.82194600	3.92703900	-1.67993100
H	1.99090700	2.33689300	2.18212900
H	2.13467800	4.64134000	3.07347500
H	1.62233200	6.59021200	1.60994800
C	1.29377400	1.67478700	-0.31269100
C	1.34001600	0.48113300	-0.81312800
H	0.52179200	-0.23992900	-0.85779100

C	-6.04157400	-2.06148300	0.01287100
C	-4.71423900	-1.65006100	-0.05256100
C	-4.41539100	-0.28191800	0.03929000
C	-5.44294300	0.66195700	0.19751700
C	-6.76660500	0.22903100	0.26040400
C	-7.09063200	-1.13442700	0.16820700
H	-6.27349800	-3.12131300	-0.05916300
H	-3.91140300	-2.37032800	-0.17472500
H	-5.21579700	1.72301400	0.26989900
H	-7.56027200	0.96133000	0.38106700
C	-8.52202900	-1.60299100	0.22873000
H	-8.67070900	-2.31395600	1.05021200
H	-9.21067200	-0.76637900	0.37579900
H	-8.80724100	-2.11802700	-0.69662400
S	-2.74867900	0.32250200	-0.04327400
O	-1.81462200	-0.88150600	-0.22482900

TS2 Charge = 0, Multiplicity = 1
Zero-point correction= 0.360514 (Hartree/Particle)
Thermal correction to Energy= 0.386762
Thermal correction to Enthalpy= 0.387707
Thermal correction to Gibbs Free Energy= 0.299404
Sum of electronic and zero-point Energies= -1871.986315
Sum of electronic and thermal Energies= -1871.960066
Sum of electronic and thermal Enthalpies= -1871.959122
Sum of electronic and thermal Free Energies= -1872.047425

C	5.01063100	-1.01666100	1.48330600
C	4.22661900	0.01343000	0.96411300
C	4.10217900	0.13378400	-0.42249100
C	4.74107200	-0.75833000	-1.28942000
C	5.51894800	-1.78147200	-0.74965700
C	5.66819200	-1.92725100	0.64015100
H	5.11487800	-1.11241000	2.56063300
H	3.72965000	0.71604400	1.62471700
H	4.64198700	-0.64734200	-2.36402800
H	6.02145100	-2.47476100	-1.41896200
C	6.53686300	-3.02111600	1.20815700
H	6.31514100	-3.20018800	2.26404000
H	6.40245500	-3.96090900	0.66270800
H	7.59801400	-2.75114600	1.13271100
S	3.07190100	1.43376000	-1.10228100
O	2.99152000	2.53567400	-0.12264600
O	3.51860200	1.70452000	-2.48702400
C	-2.13617700	3.28477200	0.90720700
C	-1.20803300	2.77882300	0.03019600
C	-0.45382800	1.59823200	0.37987700
C	-0.73246900	0.95042000	1.64141200
C	-1.67092500	1.51226500	2.51585900
C	-2.37629200	2.65369100	2.15697200
H	-2.68981300	4.18195300	0.64621700
H	-1.01421600	3.25817500	-0.92364200
H	-0.10339400	0.12985700	1.96405400

H	-1.84588800	1.03937700	3.47743900
H	-3.10620700	3.07798500	2.83958900
C	0.42671000	1.04975700	-0.49946600
C	1.40637500	0.66841600	-1.25543900
H	1.37470500	-0.07841200	-2.04646800
C	-5.69342200	-2.21139200	-0.35176400
C	-4.32567700	-2.30419400	-0.11473400
C	-3.53325200	-1.14550600	-0.16416000
C	-4.12477400	0.09843700	-0.44394400
C	-5.49709300	0.17033500	-0.68269100
C	-6.30580000	-0.97696100	-0.64138900
H	-6.30200500	-3.11192400	-0.31342500
H	-3.85905600	-3.25893000	0.10649800
H	-3.52362300	1.00288300	-0.47418100
H	-5.94833900	1.13539400	-0.89865700
C	-7.78866300	-0.90082300	-0.90405400
H	-8.05407000	-1.43790000	-1.82330200
H	-8.36006300	-1.36035500	-0.08904300
H	-8.12382300	0.13461800	-1.01237500
S	-1.77803600	-1.23724100	0.12775100
O	-1.47471300	-2.69497600	0.51338100

2 Charge = 0, Multiplicity = 1
Zero-point correction= 0.363955 (Hartree/Particle)
Thermal correction to Energy= 0.389847
Thermal correction to Enthalpy= 0.390791
Thermal correction to Gibbs Free Energy= 0.303152
Sum of electronic and zero-point Energies= -1872.052378
Sum of electronic and thermal Energies= -1872.026487
Sum of electronic and thermal Enthalpies= -1872.025543
Sum of electronic and thermal Free Energies= -1872.113182

C	3.77300200	1.37646800	-1.67253600
C	3.00498900	0.25752700	-1.34635600
C	3.09237700	-0.27036100	-0.05642800
C	3.93471000	0.29994300	0.90462700
C	4.69230500	1.41698300	0.55952400
C	4.62481700	1.97325600	-0.73019100
H	3.71312500	1.78664500	-2.67686900
H	2.35993200	-0.20550800	-2.08541900
H	4.00552500	-0.12810000	1.89913200
H	5.35148100	1.86124600	1.30082400
C	5.46642800	3.17158100	-1.08957200
H	6.52966500	2.90379600	-1.12498900
H	5.18990600	3.57587200	-2.06709900
H	5.36056000	3.96825100	-0.34490100
S	2.09894800	-1.69921200	0.37988400
O	1.70522600	-2.39337600	-0.86018800
O	2.81143700	-2.44672100	1.43998200
C	-2.61890800	-3.56896900	-1.20923300
C	-1.96777200	-2.96396400	-0.13176600
C	-1.26442500	-1.76178600	-0.31739200
C	-1.24720900	-1.16058900	-1.58677800

C	-1.90362400	-1.76726500	-2.65788900
C	-2.58908200	-2.97243800	-2.47283700
H	-3.14651600	-4.50647000	-1.05934500
H	-1.98425900	-3.43565600	0.84653600
H	-0.71910300	-0.22341600	-1.73052300
H	-1.87978500	-1.29811800	-3.63728600
H	-3.09656400	-3.44398400	-3.30953600
C	-0.61025600	-1.10648000	0.83518400
C	0.66775700	-0.98180100	1.19007100
H	0.93674900	-0.46044900	2.10841900
C	-2.27867200	3.15357700	0.11405700
C	-1.72600800	2.12014300	0.86623800
C	-2.46799500	0.95171200	1.06406800
C	-3.75453500	0.82034100	0.54244200
C	-4.29234200	1.86600500	-0.21543600
C	-3.56664500	3.04309800	-0.44374600
H	-1.70477700	4.06389300	-0.04185400
H	-0.73697000	2.22107000	1.30304000
H	-4.33611400	-0.08035000	0.71844900
H	-5.29394900	1.76413500	-0.62443000
C	-4.14065000	4.17089000	-1.26575200
H	-4.10775100	5.11853000	-0.71608900
H	-3.56642000	4.31328800	-2.18954100
H	-5.18009300	3.97479600	-1.54314900
S	-1.80105600	-0.39044700	2.09434700
O	-0.94547500	0.24732700	3.18334700

Int1′ Charge = 0, Multiplicity = 2
Zero-point correction= 0.230862 (Hartree/Particle)
Thermal correction to Energy= 0.248465
Thermal correction to Enthalpy= 0.249410
Thermal correction to Gibbs Free Energy= 0.177295
Sum of electronic and zero-point Energies= -1052.570765
Sum of electronic and thermal Energies= -1052.553162
Sum of electronic and thermal Enthalpies= -1052.552218
Sum of electronic and thermal Free Energies= -1052.624333

C	3.17266200	-0.96223500	-1.06859800
C	2.19497400	-0.00561100	-0.81485900
C	2.42450200	0.96383600	0.17357900
C	3.62734100	0.97024900	0.89774200
C	4.59484400	0.00332400	0.62758000
C	4.38819500	-0.97655900	-0.35732900
H	2.99497700	-1.71332400	-1.83438000
H	1.26169100	-0.00035700	-1.36878000
H	3.80923100	1.71975500	1.66428400
H	5.52541200	0.01116400	1.18852100
C	5.43635900	-2.01810300	-0.65673300
H	5.76608800	-1.95575900	-1.70082200
H	6.31506400	-1.89690000	-0.01717200
H	5.04273100	-3.03038200	-0.50551000
S	1.22786800	2.22129300	0.54671700
O	0.02591700	2.02142100	-0.38461300

C	-4.02581900	1.18794300	-0.62825600
C	-3.03550100	0.30854500	-0.18934800
C	-3.38706000	-0.97990800	0.26174100
C	-4.74326700	-1.36479300	0.26302000
C	-5.72465300	-0.47726600	-0.17794900
C	-5.36991500	0.80030500	-0.62467500
H	-3.74430200	2.17902200	-0.97239200
H	-1.99398800	0.61584100	-0.19296600
H	-5.01651200	-2.35655900	0.60998700
H	-6.76694500	-0.78332400	-0.17245500
H	-6.13654500	1.48955700	-0.96736000
C	-2.37878800	-1.89077300	0.71632800
C	-1.52908000	-2.66629800	1.10328500
H	-0.77888700	-3.34673400	1.44531500

TS1′ Charge = 0, Multiplicity = 2
Zero-point correction= 0.230916 (Hartree/Particle)
Thermal correction to Energy= 0.246905
Thermal correction to Enthalpy= 0.247849
Thermal correction to Gibbs Free Energy= 0.183742
Sum of electronic and zero-point Energies= -1052.547367
Sum of electronic and thermal Energies= -1052.531378
Sum of electronic and thermal Enthalpies= -1052.530434
Sum of electronic and thermal Free Energies= -1052.594542

C	-4.02796200	0.07171700	0.55258600
C	-3.06099400	-0.91471200	0.34519200
C	-1.89857300	-0.59395100	-0.35900400
C	-1.70367900	0.69951100	-0.86300100
C	-2.67844300	1.67141800	-0.64576500
C	-3.85560600	1.37681000	0.06628700
H	-4.93158500	-0.17820900	1.10286200
H	-3.20353100	-1.92458700	0.71752200
H	-0.80328800	0.94834500	-1.41964400
H	-2.52636800	2.67446100	-1.03704700
C	-4.91202300	2.43387500	0.27609700
H	-5.63873600	2.12608600	1.03348300
H	-4.46834100	3.38387900	0.59359200
H	-5.46238700	2.62905400	-0.65320100
S	-0.63536900	-1.84414000	-0.65076400
O	-1.23168200	-3.18259700	-0.21552700
C	3.99572100	2.10689700	-0.01601400
C	2.84795700	1.43938600	0.39716600
C	2.83542200	0.02057200	0.47729300
C	4.01569700	-0.69440300	0.13705600
C	5.15455200	-0.00972800	-0.27262900
C	5.15271600	1.38987900	-0.35197100
H	3.99275600	3.19153300	-0.07556800
H	1.95288400	1.99311100	0.66280800
H	4.01582600	-1.77797700	0.20115200
H	6.05021300	-0.56727000	-0.53137700
H	6.04634400	1.91790000	-0.67140800
C	1.68863500	-0.66308000	0.91183600

C	0.60910000	-1.28690100	1.07031100
H	0.02045700	-1.73305100	1.86063600

II′ Charge = 0, Multiplicity = 2

Zero-point correction= 0.232007 (Hartree/Particle)
Thermal correction to Energy= 0.248169
Thermal correction to Enthalpy= 0.249113
Thermal correction to Gibbs Free Energy= 0.184565
Sum of electronic and zero-point Energies= -1052.550151
Sum of electronic and thermal Energies= -1052.533989
Sum of electronic and thermal Enthalpies= -1052.533045
Sum of electronic and thermal Free Energies= -1052.597593

C	-4.04285500	-0.04140100	0.59909100
C	-3.00238800	-0.95167000	0.39897900
C	-1.87828800	-0.54982300	-0.32243400
C	-1.79352400	0.73654000	-0.86290500
C	-2.84129800	1.63395300	-0.65268500
C	-3.98066400	1.26219600	0.08111600
H	-4.91770600	-0.34996600	1.16606500
H	-3.06040900	-1.96311100	0.78991300
H	-0.92353500	1.04143800	-1.43950400
H	-2.77494500	2.63585700	-1.06934800
C	-5.12283600	2.22872300	0.27639900
H	-4.76802200	3.26299700	0.32526900
H	-5.83473100	2.16759800	-0.55707800
H	-5.67756400	2.01173200	1.19451300
S	-0.51333200	-1.71857600	-0.60584900
O	-1.04907800	-3.10977300	-0.26943600
C	4.05369900	2.10722900	-0.00042200
C	2.86993800	1.46311900	0.32881400
C	2.82418400	0.03508400	0.41206300
C	4.02546800	-0.69871600	0.15484900
C	5.19618300	-0.02977400	-0.17238700
C	5.22327100	1.37199900	-0.25325600
H	4.07212300	3.19179700	-0.06139300
H	1.96753100	2.03202400	0.52927200
H	4.00581400	-1.78200100	0.21887300
H	6.09973600	-0.60050900	-0.36778900
H	6.14538500	1.88572400	-0.50774200
C	1.65559000	-0.61515200	0.76711300
C	0.51927300	-1.21994500	0.88293000
H	0.03773300	-1.55236000	1.80441800

Int2′ Charge = 0, Multiplicity = 3

Zero-point correction= 0.359233 (Hartree/Particle)
Thermal correction to Energy= 0.387015
Thermal correction to Enthalpy= 0.387959
Thermal correction to Gibbs Free Energy= 0.290953
Sum of electronic and zero-point Energies= -1872.009890
Sum of electronic and thermal Energies= -1871.982108
Sum of electronic and thermal Enthalpies= -1871.981164
Sum of electronic and thermal Free Energies= -1872.078171

C	-3.47101200	-1.87116900	1.40410600
C	-2.63639500	-1.09040600	0.60487900
C	-3.12091700	-0.61256700	-0.61593500
C	-4.42147500	-0.89964500	-1.04063700
C	-5.24032100	-1.68211600	-0.22674600
C	-4.78191700	-2.17999600	1.00437300
H	-3.09599700	-2.24892000	2.35149300
H	-1.62322900	-0.86584200	0.92122400
H	-4.78138000	-0.52929300	-1.99469800
H	-6.25031100	-1.91171100	-0.55603100
C	-5.66858600	-3.04871000	1.86084200
H	-5.63774500	-4.09039200	1.51649300
H	-5.34936600	-3.03803300	2.90704600
H	-6.71231400	-2.72191500	1.81414300
S	-2.07119100	0.42523000	-1.63641500
O	-0.66047800	0.07028400	-1.37158900
O	-2.59170600	0.37902600	-3.02272200
C	1.80774300	4.60145600	-0.11920600
C	0.67827700	4.04587900	-0.70110500
C	-0.28047800	3.35617000	0.10727500
C	-0.04809700	3.26576800	1.51633700
C	1.09014100	3.83096400	2.07167000
C	2.02441800	4.50058700	1.26477100
H	2.53119400	5.11676900	-0.74455600
H	0.51039200	4.12053700	-1.77052700
H	-0.77085100	2.74916400	2.13950700
H	1.25838800	3.75090300	3.14181200
H	2.91247500	4.93855200	1.71016100
C	-1.41843400	2.81686000	-0.45887200
C	-2.36162100	2.13528300	-1.02852900
H	-3.37522500	2.47135000	-1.24756700
C	5.04578200	-2.39171500	-0.27949600
C	4.29998800	-2.07757500	0.85243800
C	3.05325800	-1.45155000	0.69975500
C	2.56109000	-1.14238100	-0.57890200
C	3.32563300	-1.46604100	-1.69909400
C	4.57523200	-2.09465000	-1.57326500
H	6.01174100	-2.87685800	-0.16144200
H	4.66963800	-2.30964200	1.84630900
H	1.59487600	-0.65971300	-0.70570400
H	2.94294700	-1.22553800	-2.68722900
C	5.39094800	-2.45892800	-2.78796000
H	5.42663800	-3.54727300	-2.92409000
H	4.96886000	-2.02282800	-3.69773400
H	6.42644900	-2.11325400	-2.69128100
S	2.04609800	-1.02203500	2.09688400
O	2.79277900	-1.44309700	3.36993400

TS6 Charge = 0, Multiplicity = 1

Zero-point correction= 0.360469 (Hartree/Particle)

Thermal correction to Energy= 0.386605

Thermal correction to Enthalpy= 0.387549

Thermal correction to Gibbs Free Energy= 0.300801
Sum of electronic and zero-point Energies= -1871.982670
Sum of electronic and thermal Energies= -1871.956533
Sum of electronic and thermal Enthalpies= -1871.955589
Sum of electronic and thermal Free Energies= -1872.042338

C	3.62443300	-1.28086200	-1.90730700
C	2.64207900	-1.15438500	-0.92784000
C	3.02761800	-1.13523100	0.41720300
C	4.37021000	-1.23338100	0.78827000
C	5.33875000	-1.35919800	-0.20978000
C	4.98534600	-1.38425000	-1.56746700
H	3.32856500	-1.30311400	-2.95296800
H	1.59490600	-1.08527100	-1.20241700
H	4.65438100	-1.22598700	1.83521200
H	6.38415600	-1.44341500	0.07445100
C	6.03247700	-1.53049500	-2.64306900
H	5.94709900	-0.73285000	-3.38984400
H	7.04236700	-1.49939600	-2.22505800
H	5.91634600	-2.48237700	-3.17543700
S	1.77433700	-0.95072300	1.68706200
O	0.50267900	-1.48485400	1.16475200
O	2.32127000	-1.46754600	2.96225000
C	-1.80127600	4.10258400	0.49252500
C	-1.13507000	3.04220400	1.11054800
C	0.21367200	2.71609800	0.71368400
C	0.81320900	3.46573500	-0.36353800
C	0.11466500	4.48544000	-0.96419200
C	-1.19998300	4.80908400	-0.54510700
H	-2.80531000	4.35948100	0.81673100
H	-1.57388300	2.52809600	1.95750800
H	1.81761500	3.20912400	-0.68320200
H	0.57356700	5.04709200	-1.77245500
H	-1.73368500	5.61997700	-1.03116800
C	0.88299300	1.67534700	1.28492800
C	1.63311000	0.86490200	1.96030700
H	2.28465200	1.15677200	2.78676300
C	-4.85276600	-1.72630200	-1.46852700
C	-3.82400900	-0.78866300	-1.53875000
C	-3.05675900	-0.51702200	-0.39635700
C	-3.32773200	-1.18991800	0.81032700
C	-4.36172400	-2.11909900	0.86272300
C	-5.14161600	-2.40749000	-0.27327300
H	-5.44084100	-1.93759000	-2.35815100
H	-3.60456800	-0.26700100	-2.46520400
H	-2.72809100	-0.99608500	1.69616400
H	-4.56182800	-2.64053200	1.79547300
C	-6.26837300	-3.40658100	-0.19806200
H	-6.59167200	-3.72101000	-1.19468900
H	-5.97334300	-4.29905600	0.36462200
H	-7.13974300	-2.97710800	0.31357500
S	-1.72910700	0.66419300	-0.42627300
O	-1.62018400	1.16535600	-1.87525300

2-1 Charge = 0, Multiplicity = 1
Zero-point correction= 0.364172 (Hartree/Particle)
Thermal correction to Energy= 0.389846
Thermal correction to Enthalpy= 0.390790
Thermal correction to Gibbs Free Energy= 0.304454
Sum of electronic and zero-point Energies= -1872.048601
Sum of electronic and thermal Energies= -1872.022927
Sum of electronic and thermal Enthalpies= -1872.021983
Sum of electronic and thermal Free Energies= -1872.108320

C	4.76014100	-0.60197900	-1.39160900
C	3.49819000	-0.95239600	-0.91756900
C	3.19609100	-0.72570900	0.42966700
C	4.13235600	-0.15840300	1.29760400
C	5.38976300	0.18834200	0.80108600
C	5.72392700	-0.02541300	-0.54505600
H	5.00371200	-0.78437500	-2.43502200
H	2.76755600	-1.40809100	-1.57740200
H	3.89122500	-0.00363400	2.34407400
H	6.12298000	0.62473500	1.47353000
C	7.08418500	0.34584700	-1.07934600
H	7.72995000	0.74118400	-0.29044800
H	7.58221100	-0.52369300	-1.52369000
H	7.00384700	1.10669800	-1.86493700
S	1.57493300	-1.15830900	1.05927600
O	0.97666400	-2.17364000	0.16614300
O	1.70202200	-1.44149200	2.50553900
C	-2.52497100	3.34698200	1.66667300
C	-1.82894800	2.15821700	1.44325700
C	-1.06545200	1.98985700	0.27265000
C	-1.01373600	3.03145400	-0.67088600
C	-1.71042800	4.21941300	-0.43773000
C	-2.46655200	4.38092500	0.72704500
H	-3.11156300	3.46363200	2.57347400
H	-1.87984700	1.35144800	2.16838900
H	-0.42554700	2.91252200	-1.57216900
H	-1.65790600	5.02093600	-1.16907800
H	-3.00702000	5.30697100	0.90210400
C	-0.31288700	0.72589100	0.08544500
C	0.63172700	0.36508900	0.96563200
H	0.89511900	1.02122900	1.79230600
C	-4.73305100	-0.90838100	-0.89206800
C	-3.50479900	-0.38452700	-1.30766200
C	-2.32880200	-0.94480100	-0.81448600
C	-2.35840800	-2.03418900	0.06285900
C	-3.59274100	-2.53848600	0.47012200
C	-4.79876600	-1.98605600	0.00238100
H	-5.65218400	-0.47147400	-1.27392000
H	-3.45624200	0.44333900	-2.00808300
H	-1.43616200	-2.47935100	0.42302600
H	-3.61919900	-3.38216600	1.15549900
C	-6.12504200	-2.53733400	0.46663700

H	-6.95816700	-2.08507000	-0.07888300
H	-6.17607900	-3.62318600	0.32756900
H	-6.27785000	-2.34181900	1.53528100
S	-0.73147800	-0.30205300	-1.42994000
O	-1.07179700	0.70887000	-2.51904100

Int4′ Charge = -1, Multiplicity = 1
Zero-point correction= 0.298717 (Hartree/Particle)
Thermal correction to Energy= 0.323022
Thermal correction to Enthalpy= 0.323966
Thermal correction to Gibbs Free Energy= 0.240813
Sum of electronic and zero-point Energies= -1792.286901
Sum of electronic and thermal Energies= -1792.262596
Sum of electronic and thermal Enthalpies= -1792.261652
Sum of electronic and thermal Free Energies= -1792.344805

C	0.10597600	2.49993900	-0.69424100
C	-0.72880700	1.39152300	-0.82840100
C	-1.27501600	0.80939600	0.31708700
C	-0.99095700	1.30914900	1.58500800
C	-0.15197200	2.41929600	1.70355000
C	0.40994100	3.02947200	0.57158800
H	0.53704700	2.95387300	-1.58317900
H	-0.94088000	0.98364400	-1.81302300
H	-1.41100500	0.82778200	2.46247900
H	0.07590400	2.81155300	2.69164400
C	1.35011400	4.20220700	0.70837300
H	1.21604900	4.92169100	-0.10635100
H	1.20095600	4.72628900	1.65749300
H	2.39485600	3.86658500	0.67805200
O	-2.65587500	-1.11391700	1.51301200
S	-2.34234600	-0.64425600	0.13218200
O	-3.75786300	0.50154300	-0.29159400
C	-4.84333000	-0.09811100	-0.80690800
C	-5.91972200	0.90309600	-1.15556700
H	-5.53420700	1.63087600	-1.87697900
H	-6.78342800	0.38577800	-1.57605000
H	-6.21872500	1.45406100	-0.25802600
O	-4.94592700	-1.30459500	-0.97110300
S	1.08304700	-2.75003600	0.24701500
O	-0.16563200	-2.08430100	-0.38941200
O	1.04807800	-2.72149800	1.78214200
C	2.42159100	-1.51962800	-0.13278400
C	3.24898500	-1.03582300	0.87917400
C	2.66296200	-1.14522300	-1.45960100
C	4.30304100	-0.16717000	0.56938200
H	3.05279400	-1.33481000	1.90537500
C	3.71750100	-0.28228900	-1.76337900
H	2.02115200	-1.51739600	-2.25478200
C	4.55822200	0.22012900	-0.75359000
H	4.93748400	0.21152100	1.36845600
H	3.89336400	0.00679700	-2.79810600
C	5.71092800	1.13623600	-1.09598900

H	5.37714700	1.99888800	-1.68507000
H	6.20047900	1.51521100	-0.19332600
H	6.47079300	0.61526400	-1.69233200

TS4′ Charge = -1, Multiplicity = 1
Zero-point correction= 0.297303 (Hartree/Particle)
Thermal correction to Energy= 0.322310
Thermal correction to Enthalpy= 0.323254
Thermal correction to Gibbs Free Energy= 0.237399
Sum of electronic and zero-point Energies= -1792.283211
Sum of electronic and thermal Energies= -1792.258204
Sum of electronic and thermal Enthalpies= -1792.257260
Sum of electronic and thermal Free Energies= -1792.343115

C	-0.34659700	2.43394200	0.38092500
C	0.40564200	1.27928900	0.59015900
C	0.99872900	0.64446800	-0.50420700
C	0.84905900	1.14889200	-1.79447000
C	0.08949900	2.30312700	-1.98928000
C	-0.52076200	2.96195900	-0.90982100
H	-0.80476000	2.93276300	1.23128700
H	0.53491300	0.88806600	1.59557700
H	1.32026800	0.64163700	-2.63027300
H	-0.03127200	2.69742500	-2.99525800
C	-1.36604000	4.19281700	-1.13071500
H	-1.25220400	4.91007400	-0.31096200
H	-1.10273200	4.69621200	-2.06609500
H	-2.43042800	3.92951900	-1.18653100
O	2.64127200	-1.16737200	-1.50765100
S	2.01195900	-0.82071400	-0.20319400
O	3.54665700	0.65267000	0.58788600
C	4.50560600	0.07329600	1.24325300
C	5.57787000	1.03636900	1.75110200
H	5.13210700	1.96244400	2.12542000
H	6.17458200	0.56272400	2.53427700
H	6.23936500	1.29955400	0.91699600
O	4.60818600	-1.14718500	1.45119900
S	-1.03674000	-2.56217200	-0.92078200
O	0.34800500	-2.30675900	-0.19643900
O	-1.08310200	-2.01252900	-2.34042400
C	-2.17522700	-1.45198000	0.00723700
C	-3.04720700	-0.61005000	-0.68003300
C	-2.24637400	-1.54724100	1.40159700
C	-3.97535500	0.15867900	0.03158100
H	-2.98368100	-0.54957800	-1.76246000
C	-3.17749700	-0.77978200	2.10211800
H	-1.56876900	-2.20562800	1.93898900
C	-4.05902200	0.08534100	1.42911600
H	-4.64495300	0.82355200	-0.50974500
H	-3.22203100	-0.85115700	3.18696700
C	-5.07950500	0.89292300	2.19668900
H	-4.61868800	1.42767800	3.03534100
H	-5.56958900	1.63055400	1.55391100

H	-5.86069700	0.24743900	2.61809900

III Charge = 0, Multiplicity = 1
Zero-point correction= 0.248920 (Hartree/Particle)
Thermal correction to Energy= 0.268183
Thermal correction to Enthalpy= 0.269128
Thermal correction to Gibbs Free Energy= 0.197842
Sum of electronic and zero-point Energies= -1563.707546
Sum of electronic and thermal Energies= -1563.688283
Sum of electronic and thermal Enthalpies= -1563.687338
Sum of electronic and thermal Free Energies= -1563.758624

C	3.29619200	1.61691600	1.05977600
C	2.52925100	0.46009300	1.21026400
C	2.44749500	-0.44067700	0.14620300
C	3.13582800	-0.21805100	-1.04842800
C	3.89459100	0.94300100	-1.18114300
C	3.98651000	1.87811000	-0.13432900
H	3.36285800	2.32275600	1.88320700
H	2.00525200	0.27175400	2.14402000
H	3.07591000	-0.94412200	-1.85307900
H	4.42863100	1.12562000	-2.11024200
C	4.80490300	3.13386600	-0.30223700
H	5.82357700	2.90168200	-0.63253300
H	4.36124400	3.78977100	-1.06136700
H	4.86978900	3.69734600	0.63271000
O	1.86719300	-2.87689300	-0.74592100
S	1.48319800	-1.94926100	0.36453200
S	-1.46391500	-1.86665400	0.57776500
O	-0.01087100	-1.16349200	-0.08685100
O	-1.95650500	-2.92068000	-0.36457000
C	-2.42728000	-0.37656600	0.24664600
C	-2.32892900	0.70144400	1.13041500
C	-3.30280800	-0.34713100	-0.83969900
C	-3.10648500	1.83642300	0.90039400
H	-1.65694500	0.66537400	1.98386800
C	-4.07082400	0.79680800	-1.05494500
H	-3.37958100	-1.20677800	-1.49799300
C	-3.98484400	1.90422300	-0.19380200
H	-3.03528100	2.67835900	1.58375500
H	-4.75332500	0.82757800	-1.90034300
C	-4.80265800	3.14459700	-0.45315200
H	-4.92884200	3.73885400	0.45660200
H	-4.31158900	3.78232200	-1.19949500
H	-5.79442000	2.89429900	-0.84290900

TS5′ Charge = 0, Multiplicity = 1
Zero-point correction= 0.247366 (Hartree/Particle)
Thermal correction to Energy= 0.266570
Thermal correction to Enthalpy= 0.267514
Thermal correction to Gibbs Free Energy= 0.196085
Sum of electronic and zero-point Energies= -1563.658840
Sum of electronic and thermal Energies= -1563.639637

Sum of electronic and thermal Enthalpies= -1563.638692
Sum of electronic and thermal Free Energies= -1563.710121

C	4.03140100	0.51593600	-0.67591200
C	3.26995400	-0.58350000	-0.32574700
C	2.16136800	-0.39679100	0.52655200
C	1.81979300	0.88387300	1.01353400
C	2.59997600	1.97193100	0.64754500
C	3.71177000	1.81080200	-0.20035600
H	4.88758400	0.38842800	-1.33212700
H	3.50655400	-1.57533300	-0.69657800
H	0.96099500	1.01969600	1.66565600
H	2.34926700	2.96080200	1.01862900
C	4.55259900	2.98713600	-0.60373700
H	5.60414300	2.82114700	-0.34062500
H	4.51905400	3.12984000	-1.69101800
H	4.21498100	3.90913400	-0.12471800
S	1.18198300	-1.71741000	1.08104700
O	1.82783500	-3.00640300	0.70696700
C	-2.43398900	1.91693000	-0.81964000
C	-1.77063000	0.69361600	-0.96328800
C	-2.16502800	-0.39233000	-0.18312000
C	-3.20397700	-0.26305200	0.74388500
C	-3.85279200	0.96389300	0.88131800
C	-3.48058800	2.07355900	0.10097000
H	-2.13295700	2.76114100	-1.43552100
H	-0.96069800	0.57645500	-1.67659800
H	-3.51213200	-1.11122100	1.35055300
H	-4.66298500	1.06162000	1.60021400
C	-4.19364900	3.39494800	0.25921700
H	-3.78910800	4.15103900	-0.41990600
H	-5.26521500	3.29349000	0.04959700
H	-4.10165500	3.77600100	1.28336900
S	-1.35400900	-2.02839500	-0.36511800
O	-2.36183600	-2.87473500	-1.12000600
O	-0.10883400	-1.70524400	-1.22610100

TS5′′ Charge = 0, Multiplicity = 1
Zero-point correction= 0.245852 (Hartree/Particle)
Thermal correction to Energy= 0.265375
Thermal correction to Enthalpy= 0.266320
Thermal correction to Gibbs Free Energy= 0.193728
Sum of electronic and zero-point Energies= -1563.624272
Sum of electronic and thermal Energies= -1563.604749
Sum of electronic and thermal Enthalpies= -1563.603805
Sum of electronic and thermal Free Energies= -1563.676397

C	-3.56596600	-1.22805700	0.52794000
C	-2.38253800	-0.71445100	-0.00140100
C	-2.26426800	0.67130400	-0.16625100
C	-3.30291000	1.53833400	0.20533500
C	-4.47335200	0.99827700	0.73001300
C	-4.62899900	-0.38955500	0.90000600

H	-3.66018100	-2.30300400	0.65565900
H	-1.57371900	-1.38327700	-0.27480500
H	-3.19505700	2.61018500	0.08086600
H	-5.28193400	1.66710400	1.01362200
C	-5.91275500	-0.95639300	1.44912600
H	-6.69417400	-0.96312400	0.67813100
H	-5.77970700	-1.98504400	1.79590400
H	-6.28818400	-0.35542800	2.28407600
O	-0.93078300	2.89730000	-0.89536000
S	-0.75331400	1.37619500	-0.82928100
S	1.19661900	-1.51115800	-1.46019800
O	0.06281600	0.06897300	-1.70339100
O	0.45036700	-2.51519900	-0.61244400
C	2.43898000	-0.76045500	-0.40900700
C	3.36139200	0.11812100	-0.98918300
C	2.50860400	-1.08214200	0.95081200
C	4.35255400	0.68700700	-0.18947100
H	3.30718900	0.36533200	-2.04626700
C	3.50548500	-0.50328100	1.73322200
H	1.79312700	-1.77628700	1.38025600
C	4.44199200	0.38883100	1.18008200
H	5.06726300	1.37289400	-0.63587400
H	3.56043800	-0.74872600	2.79097300
C	5.51982000	0.99770800	2.04181100
H	6.19299400	0.22571500	2.43404400
H	6.12311900	1.71668000	1.48059800
H	5.08851400	1.51633000	2.90584400

TS3′ Charge = 0, Multiplicity = 1
Zero-point correction= 0.174779 (Hartree/Particle)
Thermal correction to Energy= 0.191277
Thermal correction to Enthalpy= 0.192221
Thermal correction to Gibbs Free Energy= 0.128621
Sum of electronic and zero-point Energies= -1595.250096
Sum of electronic and thermal Energies= -1595.233598
Sum of electronic and thermal Enthalpies= -1595.232653
Sum of electronic and thermal Free Energies= -1595.296254

C	3.21926400	0.13396900	1.07995700
C	1.87126900	-0.15387700	1.30909100
C	1.04656600	-0.46956900	0.22771000
C	1.55365500	-0.49031500	-1.07539900
C	2.90131600	-0.20278600	-1.28631700
C	3.75521600	0.11718500	-0.21610500
H	3.86249100	0.37131800	1.92338800
H	1.47252300	-0.15050900	2.31837200
H	0.90617000	-0.73980400	-1.90893800
H	3.29793100	-0.22697900	-2.29848700
C	5.21269400	0.41919300	-0.46486100
H	5.73838100	-0.46673600	-0.84174700
H	5.33147700	1.20832100	-1.21623800
H	5.71599200	0.74400700	0.45029500
S	-0.71145000	-0.78628800	0.53470300

O	-1.14480800	-1.70515600	-0.60597800
O	-0.79571900	-1.38931700	1.91040400
Na	-3.19207100	-1.46230100	-1.37966700
C	-2.10276300	1.62900300	1.67038900
H	-2.40926300	0.95483700	2.47490800
H	-1.08654900	1.98255400	1.84494100
H	-2.78268600	2.48830000	1.64743400
C	-2.23664900	0.90275300	0.35754900
O	-3.24734100	0.31169000	-0.01501300
Cl	-1.37476100	1.96762000	-1.04119000

I′ Charge = 0, Multiplicity = 1
Zero-point correction= 0.174071 (Hartree/Particle)
Thermal correction to Energy= 0.187443
Thermal correction to Enthalpy= 0.188388
Thermal correction to Gibbs Free Energy= 0.132980
Sum of electronic and zero-point Energies= -972.654318
Sum of electronic and thermal Energies= -972.640946
Sum of electronic and thermal Enthalpies= -972.640001
Sum of electronic and thermal Free Energies= -972.695409

C	2.35147300	1.22659200	0.08494200
C	0.98659800	1.19744200	0.37582000
C	0.32237000	-0.03179300	0.37276000
C	0.99593900	-1.22295400	0.07536000
C	2.35648300	-1.17025200	-0.21648400
C	3.05629600	0.05005000	-0.21378000
H	2.87604600	2.17794100	0.09635400
H	0.45227000	2.11031600	0.61725400
H	0.46851900	-2.17095100	0.08453700
H	2.88700200	-2.09160300	-0.44199500
C	4.53413200	0.08183000	-0.51006700
H	5.10149900	-0.40373900	0.29346000
H	4.76442700	-0.45594400	-1.43647600
H	4.90105300	1.10715600	-0.60776600
S	-1.43192300	-0.08803000	0.73466900
O	-1.74536000	-1.39290500	1.35669600
O	-1.81829800	1.17704500	1.40585600
C	-2.73324600	1.23041500	-1.50394600
H	-3.43489100	1.66791200	-0.78595500
H	-1.88047800	1.91445200	-1.57777200
H	-3.20750400	1.11211000	-2.48026400
C	-2.27962800	-0.10300200	-1.01270200
O	-2.38130200	-1.17278100	-1.54098800

IX. Characterization data of the products

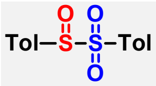

***p*-Toluenesulfinyl *p*-tolyl sulfone (1):** White Solid; **^{1}H-NMR** (500 MHz, $CDCl_3$) δ 7.52 (d, *J* = 8.5 Hz, 2H), 7.48 (d, *J* = 8.0 Hz, 2H), 7.32–7.28 (m, 4H), 2.46 (s, 3H), 2.44 (s, 3H); **^{13}C-NMR** (125 MHz, $CDCl_3$) δ 146.9, 144. 6, 134.8, 130.3, 129.9, 129.81, 129.77, 125.8, 21.9, 21.8; **HRMS** (ESI) *m/z* calculated $C_{14}H_{14}NaO_3S_2$ $[M+Na]^+$ 317.0277, found 317.0258.

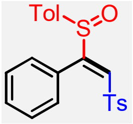

(*E*)-1-Methyl-4-((2-phenyl-2-(*p*-tolylsulfinyl)vinyl)sulfonyl)benzene (2) White Solid; mp: 167-168 °C; **^{1}H-NMR** (600 MHz, $CDCl_3$) δ 7.55 (d, *J* = 7.8 Hz, 2H), 7.35 (t, *J* = 7.8 Hz, 1H), 7.33 (s, 1H), 7.24 (t, *J* = 7.8 Hz, 2H), 7.21 (d, *J* = 8.4 Hz, 2H), 7.12 – 7.08 (m, 4H), 6.91 (d, *J* = 7.2 Hz, 2H), 2.40 (s, 3H), 2.33 (s, 3H); **^{13}C-NMR** (150 MHz, $CDCl_3$) δ 160.7, 144.8, 142.8, 137.5, 136.9, 130.1, 129.9, 129.7, 129.5, 129.3, 128.2, 128.1, 127.9, 125.5, 21.6, 21.5; **HRMS** (ESI) *m/z* calculated $C_{22}H_{20}NaO_3S_2$ $[M+Na]^+$ 419.0746, found 419.0751.

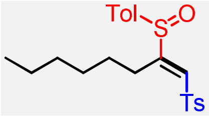

(*E*)-1-Methyl-4-((2-(*p*-tolylsulfinyl)oct-1-en-1-yl)sulfonyl)benzene (3): White Solid; mp: 108-109 °C; **^{1}H-NMR** (600 MHz, $CDCl_3$) δ 7.82 (d, *J* = 8.4 Hz, 2H), 7.48 (d, *J* = 8.4 Hz, 2H), 7.37 (d, *J* = 8.4 Hz, 2H), 7.30 (d, *J* = 7.8 Hz, 2H), 7.13 (s, 1H), 2.99 – 2.93 (m, 1H), 2.47 (s, 3H), 2.42 (s, 3H), 2.05 – 1.98 (m, 1H), 1.56 – 1.49 (m, 1H), 1.28 – 1.17 (m, 7H), 0.86 (t, *J* = 7.2 Hz, 3H); **^{13}C-NMR** (150 MHz, $CDCl_3$) δ 162.2, 145.0, 143.5, 137.9, 137.7, 130.4, 130.0, 127.7, 127.5, 126.5, 31.2, 29.3, 29.2, 26.7, 22.4, 21.7, 21.6, 14.0; **HRMS** (ESI) *m/z* calculated $C_{22}H_{28}NaO_3S_2$ $[M+Na]^+$ 427.1372, found 427.1385.

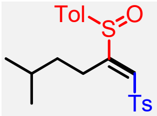

(*E*)-1-Methyl-4-((5-methyl-1-tosylhex-1-en-2-yl)sulfinyl)benzene (4): White Solid; mp: 104-105 °C; **^{1}H-NMR** (600 MHz, $CDCl_3$) δ 7.82 (d, *J* = 8.4 Hz, 2H), 7.48 (d, *J* = 8.4 Hz, 2H), 7.37 (d, *J* = 8.4 Hz, 2H), 7.30 (d, *J* = 8.4 Hz, 2H), 7.12 (s, 1H), 2.98 – 2.91 (m, 1H), 2.47 (s, 3H), 2.42 (s, 3H), 2.05 – 1.98 (m, 1H), 1.55 – 1.47 (m, 1H), 1.45 – 1.38 (m, 1H), 1.16 – 1.07 (m, 1H), 0.86 – 0.82 (m, 6H); **^{13}C-NMR** (150 MHz, $CDCl_3$) δ 162.5, 145.0, 143.5, 137.9, 137.7, 130.4, 130.0, 127.6, 127.4, 126.5, 38.1, 28.3, 24.8, 22.04, 22.01, 21.7, 21.5; **HRMS** (ESI) *m/z* calculated $C_{21}H_{26}NaO_3S_2$ $[M+Na]^+$ 413.1216, found 413.1214.

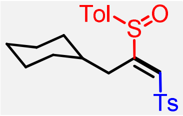

(*E*)-1-((3-Cyclohexyl-1-tosylprop-1-en-2-yl)sulfinyl)-4-methylbenzene (5): White Solid; mp: 130-131 °C; **^{1}H-NMR** (600 MHz, $CDCl_3$) δ 7.81 (d, *J* = 8.4 Hz, 2H), 7.43 (d, *J* = 8.4 Hz, 2H), 7.37 (d, *J* = 8.4 Hz, 2H), 7.28 (d, *J* = 8.4 Hz, 2H), 7.21 (s, 1H), 3.25 (dd, *J* = 14.4, 7.8 Hz, 1H), 2.47 (s, 3H), 2.41 (s, 3H), 1.74 – 1.63 (m, 6H), 1.62 – 1.56 (m, 1H), 1.24 – 1.10 (m, 3H), 1.02 – 0.93 (m, 2H); **^{13}C-NMR** (150 MHz, $CDCl_3$) δ 161.2, 144.9, 143.4, 137.9, 130.4, 130.0, 127.9, 127.7, 126.4, 37.9, 33.8, 33.5, 32.7, 26.1, 26.0, 21.6, 21.5; **HRMS** (ESI) *m/z* calculated $C_{23}H_{28}NaO_3S_2$ $[M+Na]^+$ 439.1372, found 439.1381.

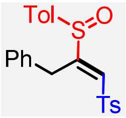

(*E*)-1-Methyl-4-((3-phenyl-1-tosylprop-1-en-2-yl)sulfinyl)benzene (6): White Solid; mp: 142-143 °C; **^{1}H-NMR** (400 MHz, $CDCl_3$) δ 7.73 (d, *J* = 8.4 Hz, 2H), 7.42 (d, *J* = 8.4 Hz, 2H), 7.35 – 7.31 (m, 3H), 7.30 – 7.22 (m, 5H), 7.11 – 7.07 (m, 2H), 4.83 (d, *J* = 16.0 Hz, 1H), 3.12 (d, *J* = 16.0 Hz, 1H), 2.46 (s, 3H), 2.41 (s, 3H); **^{13}C-NMR** (150 MHz, $CDCl_3$) δ 160.4, 145.2, 143.6, 137.5, 137.4, 135.2, 130.4, 130.0, 128.9, 128.74, 128.72, 127.8, 127.1, 126.7, 31.8, 21.7, 21.6; **HRMS** (ESI) *m/z* calculated $C_{23}H_{22}NaO_3S_2$ $[M+Na]^+$ 433.0903, found 433.0913.

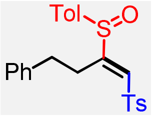

(*E*)-1-Methyl-4-((4-phenyl-1-tosylbut-1-en-2-yl)sulfinyl)benzene (7): White Solid; mp: 125-126 °C; **^{1}H-NMR** (400 MHz, DMSO) δ 7.84 (d, *J* = 8.4 Hz, 2H), 7.57 (d, *J* = 8.0 Hz, 2H), 7.50 (d, *J* = 8.0 Hz, 2H), 7.41 (d, *J* = 8.0 Hz, 2H), 7.29 (t, *J* = 7.2 Hz, 2H), 7.21 (t, *J* = 7.2 Hz, 1H), 7.15 (s, 1H), 7.10 (d, *J* = 7.2 Hz, 2H), 3.01 – 2.92 (m, 1H), 2.75 – 2.67 (m, 1H), 2.46 – 2.27 (m, 8H); **^{13}C-NMR** (150 MHz, DMSO) δ 162.5, 145.7, 143.6, 140.2, 138.0, 137.7, 130.9, 130.8, 129.0, 128.6, 128.1, 127.8, 126.9, 126.5, 35.2, 28.3, 21.6, 21.5; **HRMS** (ESI) *m/z* calculated $C_{24}H_{24}NaO_3S_2$ $[M+Na]^+$ 447.1059, found 447.1055.

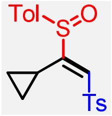

(*E*)-1-((2-cyclopropyl-2-(p-tolylsulfinyl)vinyl)sulfonyl)-4-methylbenzene (8): White Solid; mp: 101-102 °C; **^{1}H-NMR** (600 MHz, $CDCl_3$) δ 7.85 (d, *J* = 8.4 Hz, 2H), 7.51 (d, *J* = 7.8 Hz, 2H), 7.37 (d, *J* = 7.8 Hz, 2H), 7.31 – 7.27 (m, 3H), 2.46 (s, 3H), 2.41 (s, 3H), 1.99 – 1.94 (m, 1H), 1.11 – 1.07 (m, 1H), 0.83 – 0.78 (m, 2H), 0.73 – 0.69 (m, 1H); **^{13}C-NMR** (150 MHz, $CDCl_3$) δ 161.9, 145.0, 143.4, 138.4, 138.1, 130.5, 130.4, 130.0, 127.7, 126.7, 21.7, 21.6, 9.3, 8.7, 5.9; **HRMS** (ESI) *m/z* calculated $C_{19}H_{20}NaO_3S_2$ $[M+Na]^+$ 383.0746, found 383.0755.

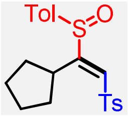

(*E*)-1-((2-Cyclopentyl-2-(*p*-tolylsulfinyl)vinyl)sulfonyl)-4-methylbenzene (9): White Solid; mp: 117-118 °C; **^{1}H-NMR** (600 MHz, $CDCl_3$) δ 7.85 (d, *J* = 8.4 Hz, 2H), 7.50 (d, *J* = 8.4 Hz, 2H), 7.38 (d, *J* = 8.4 Hz, 2H), 7.29 (d, *J* = 8.4 Hz, 2H), 7.23 (s, 1H), 3.53 – 3.45 (m, 1H), 2.47 (s, 3H), 2.41 (s, 3H), 1.78 – 1.73 (m, 2H), 1.70 – 1.64 (m, 1H), 1.63 – 1.58 (m, 1H), 1.56 – 1.50 (m, 2H), 1.48 – 1.43 (m, 1H), 1.24 – 1.16 (m, 1H); **^{13}C-NMR** (150 MHz, $CDCl_3$) δ 163.8, 144.9, 143.5, 138.8, 138.0, 130.4, 130.0, 128.2, 127.7, 127.3, 39.3, 32.0, 31.9, 25.5, 25.1, 21.7, 21.6; **HRMS** (ESI) *m/z* calculated $C_{21}H_{24}NaO_3S_2$ $[M+Na]^+$ 411.1059, found 411.1067.

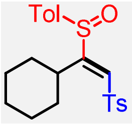

(*E*)-1-((2-Cyclohexyl-2-(*p*-tolylsulfinyl)vinyl)sulfonyl)-4-methylbenzene (10): White Solid; mp: 138-139 °C; **^{1}H-NMR** (600 MHz, $CDCl_3$) δ 7.85 (d, *J* = 8.4 Hz, 2H), 7.51 (d, *J* = 8.4 Hz, 2H), 7.38 (d, *J* = 7.8 Hz, 2H), 7.29 (d, *J* = 7.8 Hz, 2H), 7.17 (s, 1H), 3.20 (tt, *J* = 12.6, 3.2 Hz, 1H), 2.46 (s, 3H), 2.42 (s, 3H), 1.73 – 1.70 (m, *J* = 13.8 Hz, 1H), 1.66 – 1.58 (m, 3H), 1.51 – 1.48 (m, 1H), 1.41 – 1.38 (m, 1H), 1.24 – 1.13 (m, 2H), 1.07 – 0.93 (m, 2H); **^{13}C-NMR** (150 MHz, $CDCl_3$) δ 165.0, 144.9, 143.6, 138.6, 138.0, 130.3, 130.0, 127.7, 127.6, 127.3, 39.0, 31.3, 30.6, 26.3, 26.2, 25.2, 21.63, 21.57; **HRMS** (ESI) *m/z* calculated $C_{22}H_{26}NaO_3S_2$ $[M+Na]^+$ 425.1216, found 425.1224.

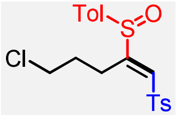

(*E*)-1-((5-Chloro-1-tosylpent-1-en-2-yl)sulfinyl)-4-methylbenzene (11) White Solid; mp: 122-123 °C; **^{1}H-NMR** (600 MHz, $CDCl_3$) δ 7.81 (d, *J* = 8.4 Hz, 2H), 7.50 (d, *J* = 8.4 Hz, 2H), 7.38 (d, *J* = 8.4 Hz, 2H), 7.31 (d, *J* = 8.4 Hz, 2H), 7.17 (s, 1H), 3.56 – 3.46 (m, 2H), 2.98 – 2.91 (m, 1H), 2.47 (s, 3H), 2.42 (s, 3H), 2.39 – 2.33 (m, 1H), 2.06 – 1.97 (m, 1H), 1.70 – 1.62 (m, 1H); **^{13}C-NMR** (150 MHz, $CDCl_3$) δ 160.5, 145.3, 143.7, 137.5, 137.2, 130.5, 130.1, 128.6, 127.7, 126.5, 44.3, 32.0, 24.2, 21.7, 21.6; **HRMS** (ESI) *m/z* calculated $C_{19}H_{21}ClNaO_3S_2$ $[M+Na]^+$ 419.0513, found 419.0521.

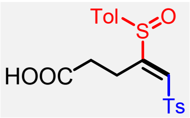

(*E*)-4-(*p*-tolylsulfinyl)-5-tosylpent-4-enoic acid (12) White Solid; mp: 90-91 °C; **^{1}H-NMR** (600 MHz, $CDCl_3$) δ 7.82 (d, *J* = 8.4 Hz, 2H), 7.70 (br., 1H), 7.50 (d, *J* = 7.8 Hz, 2H), 7.38 (d, *J* = 7.8 Hz, 2H), 7.31 (d, *J* = 7.8 Hz, 2H), 7.19 (s, 1H), 3.03 – 2.97 (m, 1H), 2.67 – 2.60 (m, 1H), 2.58 – 2.53 (m, 1H), 2.47 (s, 3H), 2.41 (s, 3H), 2.24 – 2.16 (m, 1H); **^{13}C-NMR** (150 MHz, $CDCl_3$) δ 176.3, 159.3, 145.4, 144.0, 137.2, 136.7, 130.6, 130.2, 129.3, 127.7, 126.5, 32.9, 21.7, 21.6, 21.5; **HRMS** (ESI) *m/z* calculated $C_{19}H_{20}NaO_5S_2$ $[M+Na]^+$ 415.0644, found 415.0648.

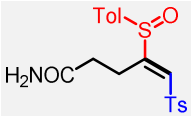

(*E*)-4-(*p*-tolylsulfinyl)-5-tosylpent-4-enamide (13) White Solid; mp: 189-190 °C; **^{1}H-NMR** (600 MHz, DMSO) δ 7.86 (d, *J* = 8.4 Hz, 2H), 7.54 – 7.49 (m, 4H), 7.39 (d, *J* = 7.8 Hz, 2H), 7.35 (s, 1H), 7.11 (s, 1H), 6.87 (s, 1H), 2.98 – 2.90 (m, 1H), 2.44 (s, 3H), 2.37 (s, 3H), 2.36 – 2.30 (m, 1H), 2.29 – 2.23 (m, 1H), 2.12 – 2.06 (m, 1H); **^{13}C-NMR** (150 MHz, DMSO) δ 172.4, 163.3, 145.7, 143.5, 138.2, 137.7, 130.9, 130.8, 127.9, 127.8, 126.4, 34.4, 21.9, 21.6, 21.5; **HRMS** (ESI) *m/z* calculated $C_{19}H_{22}NO_4S_2$ $[M+H]^+$ 392.0985, found 392.0974.

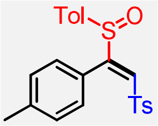

(*E*)-1-Methyl-4-((2-(*p*-tolyl)-2-(*p*-tolylsulfinyl)vinyl)sulfonyl)benzene (14) White Solid; mp: 166-167 °C; **^{1}H-NMR** (600 MHz, $CDCl_3$) δ 7.57 (d, *J* = 8.0 Hz, 2H), 7.28 (s, 1H), 7.22 (d, *J* = 8.0 Hz, 2H), 7.13 – 7.10 (m, 4H), 7.07 (d, *J* = 7.8 Hz, 2H), 6.85 (d, *J* = 7.8 Hz, 2H), 2.40 (s, 3H), 2.34 (s, 3H), 2.33 (s, 3H); **^{13}C-NMR** (150 MHz, $CDCl_3$) δ 160.6, 144.7, 142.6, 140.4, 137.6, 137.1, 129.8, 129.7, 129.1, 128.9, 128.8, 127.9, 125.4, 125.1, 21.6, 21.44, 21.41; **HRMS** (ESI) *m/z* calculated $C_{23}H_{22}NaO_3S_2$ $[M+Na]^+$ 433.0903, found 433.0912.

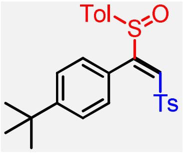

(*E*)-1-(*tert*-Butyl)-4-(1-(*p*-tolylsulfinyl)-2-tosylvinyl)benzene (15) White Solid; mp: 157-158 °C; **^{1}H-NMR** (600 MHz, $CDCl_3$) δ 7.52 (d, *J* = 7.8 Hz, 2H), 7.32 (s, 1H), 7.24 (d, *J* = 7.8 Hz, 2H), 7.17 (d, *J* = 8.4 Hz, 2H), 7.11 – 7.06 (m, 4H), 6.84 (d, *J* = 8.4 Hz, 2H), 2.39 (s, 3H), 2.33 (s, 3H), 1.30 (s, 9H); **^{13}C-NMR** (150 MHz, $CDCl_3$) δ 160.8, 153.5, 144.5, 142.5, 137.5, 137.2, 129.7, 129.5, 129.3, 129.0, 127.9, 125.3, 125.1, 125.0, 34.8, 31.2, 21.6, 21.4; **HRMS** (ESI) *m/z* calculated $C_{26}H_{28}NaO_3S_2$ $[M+Na]^+$ 475.1372, found 475.1381.

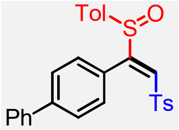

(*E*)-4-(1-(*p*-Tolylsulfinyl)-2-tosylvinyl)-1,1'-biphenyl (16) White Solid; mp: 162-163 °C; **^{1}H-NMR** (600 MHz, $CDCl_3$) δ 7.61 – 7.57 (m, 4H), 7.49 (d, *J* = 7.8 Hz, 2H), 7.46 (t, *J* = 7.8 Hz, 2H), 7.38 (t, *J* = 7.2 Hz, 1H), 7.35 (s, 1H), 7.22 (d, *J* = 7.8 Hz, 2H), 7.15 (d, *J* = 7.8 Hz, 2H), 7.12 (d, *J* = 8.4 Hz, 2H), 7.01 (d, *J* = 8.4 Hz, 2H), 2.40 (s, 3H), 2.34 (s, 3H); **^{13}C-NMR** (150 MHz, $CDCl_3$) δ 160.3, 144.8, 142.7, 139.7, 137.5, 137.0, 129.9, 129.7, 129.5, 128.9, 128.0, 127.9, 127.03, 127.01, 126.6, 125.5, 21.6, 21.4; **HRMS** (ESI) *m/z* calculated $C_{28}H_{24}NaO_3S_2$ $[M+Na]^+$ 495.1059, found 495.1047.

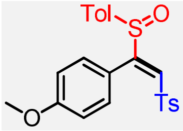

(*E*)-1-Methoxy-4-(1-(*p*-tolylsulfinyl)-2-tosylvinyl)benzene (17) White Solid; mp: 150-151 °C; **^{1}H-NMR** (600 MHz, $CDCl_3$) δ 7.58 (d, *J* = 8.4 Hz, 2H), 7.27 (s, 1H), 7.23 (d, *J* = 8.4 Hz, 2H), 7.12 (s, 4H), 6.93 (d, *J* = 8.4 Hz, 2H), 6.79 (d, *J* = 8.4 Hz, 2H), 3.81 (s, 3H), 2.41 (s, 3H), 2.33 (s, 3H); **^{13}C-NMR** (150 MHz, $CDCl_3$) δ 161.2, 160.5, 144.8, 142.6, 137.7, 137.4, 130.9, 129.9, 129.7, 128.7, 127.9, 125.4, 120.2, 113.7, 55.3, 21.7, 21.5; **HRMS** (ESI) *m/z* calculated $C_{23}H_{22}NaO_4S_2$ $[M+Na]^+$ 449.0852, found 449.0853.

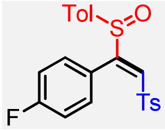

(*E*)-1-Fluoro-4-(1-(*p*-tolylsulfinyl)-2-tosylvinyl)benzene (18) White Solid; mp: 148-149 °C; **^{1}H-NMR** (600 MHz, $CDCl_3$) δ 7.57 (d, *J* = 8.4 Hz, 2H), 7.34 (s, 1H), 7.24 (d, *J* = 7.8 Hz, 2H), 7.15 – 7.10 (m, 4H), 6.95 (t, *J* = 8.4 Hz, 2H), 6.93 – 6.89 (m, 2H), 2.41 (s, 3H), 2.34 (s, 3H); **^{13}C-NMR** (150 MHz, $CDCl_3$) δ 163.6 (d, *J* = 251.5 Hz), 159.5, 145.0, 142.9, 137.3, 136.7, 131.2 (d, *J* = 9.0 Hz), 129.88, 129.87, 129.7, 127.8, 125.4, 124.1 (d, *J* = 3.2 Hz), 115.4 (d, *J* = 21.0 Hz), 21.5, 21.4; **HRMS** (ESI) *m/z* calculated $C_{22}H_{19}FNaO_3S_2$ $[M+Na]^+$ 437.0652, found 437.0637.

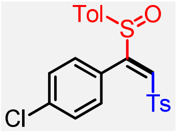

(*E*)-1-Chloro-4-(1-(*p*-tolylsulfinyl)-2-tosylvinyl)benzene (19) White Solid; mp: 185-186 °C; **^{1}H-NMR** (600 MHz, $CDCl_3$) δ 7.58 (d, *J* = 8.4 Hz, 2H), 7.33 (s, 1H), 7.26 (d, *J* = 8.4 Hz, 2H), 7.24 – 7.22 (m, 2H), 7.16 – 7.12 (m, 4H), 6.86 (d, *J* = 8.4 Hz, 2H), 2.42 (s, 3H), 2.35 (s, 3H); **^{13}C-NMR** (150 MHz, $CDCl_3$) δ 159.4, 145.1, 143.1, 137.3, 136.7, 136.5, 130.5, 130.1, 130.0, 129.9, 128.5, 127.9, 126.7, 125.5, 21.7, 21.5; **HRMS** (ESI) *m/z* calculated $C_{22}H_{19}ClNaO_3S_2$ $[M+Na]^+$ 453.0356, found 453.0339.

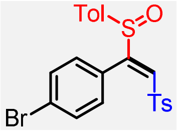

(*E*)-1-Bromo-4-(1-(*p*-tolylsulfinyl)-2-tosylvinyl)benzene (20) White Solid; mp: 189-190 °C; **^{1}H-NMR** (600 MHz, $CDCl_3$) δ 7.58 (d, *J* = 8.4 Hz, 2H), 7.39 (d, *J* = 8.4 Hz, 2H), 7.33 (s, 1H), 7.26 (d, *J* = 8.4 Hz, 2H), 7.17 – 7.12 (m, 4H), 6.79 (d, *J* = 8.4 Hz, 2H), 2.43 (s, 3H), 2.36 (s, 3H); **^{13}C-NMR** (150 MHz, $CDCl_3$) δ 159.3, 145.1, 143.1, 137.2, 136.6, 131.4, 130.7, 130.0, 129.8, 127.9, 127.2, 125.5, 124.8, 21.7, 21.5; **HRMS** (ESI) *m/z* calculated $C_{22}H_{19}BrNaO_3S_2$ $[M+Na]^+$ 496.9851, found 496.9841.

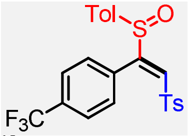

(*E*)-1-Methyl-4-((2-(*p*-tolylsulfinyl)-2-(4-(trifluoromethyl)phenyl)vinyl)sulfonyl)benzene (21) White Solid; mp: 174-175 °C; **^{1}H-NMR** (600 MHz, $CDCl_3$) δ 7.56 (d, *J* = 8.4 Hz, 2H), 7.49 (d, *J* = 8.4 Hz, 2H), 7.38 (s, 1H), 7.24 (d, *J* = 8.4 Hz, 2H), 7.16 (d, *J* = 8.4 Hz, 2H), 7.13 (d, *J* = 8.4 Hz, 2H), 7.00 (d, *J* = 8.4 Hz, 2H), 2.42 (s, 3H), 2.36 (s, 3H); **^{13}C-NMR** (125 MHz, $CDCl_3$) δ 158.8, 145.2, 143.3, 136.9, 136.2, 132.0, 131.8 (q, *J* = 34.5 Hz), 130.7, 130.1, 129.9, 129.6, 127.9, 125.5, 124.9 (q, *J* = 3.0 Hz), 123.5 (q, *J* = 271.5 Hz), 21.6, 21.5; **HRMS** (ESI) *m/z* calculated $C_{23}H_{19}F_3NaO_3S_2$ $[M+Na]^+$ 487.0620, found 487.0628.

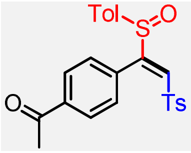

(*E*)-1-(4-(1-(*p*-Tolylsulfinyl)-2-tosylvinyl)phenyl)ethanone (22) White Solid; mp: 145-146 °C; **^{1}H-NMR** (600 MHz, $CDCl_3$) δ 7.84 (d, *J* = 8.4 Hz, 2H), 7.60 (d, *J* = 8.4 Hz, 2H), 7.34 (s, 1H), 7.27 (d, *J* = 8.4 Hz, 2H), 7.15 – 7.11 (m, 4H), 7.04 (d, *J* = 8.4 Hz, 2H), 2.60 (s, 3H), 2.43 (s, 3H), 2.35 (s, 3H); **^{13}C-NMR** (125 MHz, $CDCl_3$) δ 197.2, 159.4, 145.2, 143.2, 137.8, 137.2, 136.4, 133.0, 130.1, 130.0, 129.9, 129.5, 127.9, 127.8, 125.5, 26.6, 21.6, 21.5; **HRMS** (ESI) *m/z* calculated $C_{24}H_{22}NaO_4S_2$ $[M+Na]^+$ 461.0852, found 461.0863.

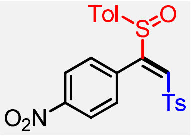

(*E*)-1-Methyl-4-((2-(4-nitrophenyl)-2-(*p*-tolylsulfinyl)vinyl)sulfonyl)benzene (23) White Solid; mp: 180-181 °C; **^{1}H-NMR** (600 MHz, $CDCl_3$) δ 8.11 (d, *J* = 8.4 Hz, 2H), 7.61 (d, *J* = 8.4 Hz, 2H), 7.38 (s, 1H), 7.30 (d, *J* = 7.8 Hz, 2H), 7.19 – 7.13 (m, 4H), 7.09 (d, *J* = 8.4 Hz, 2H), 2.44 (s, 3H), 2.36 (s, 3H); **^{13}C-NMR** (150 MHz, $CDCl_3$) δ 158.1, 148.4, 145.5, 143.6, 136.8, 136.0, 135.0, 131.0, 130.18, 130.16, 130.0, 127.9, 125.5, 123.1, 21.6, 21.5; **HRMS** (ESI) *m/z* calculated $C_{22}H_{19}NNaO_5S_2$ $[M+Na]^+$ 464.0597, found 464.0586.

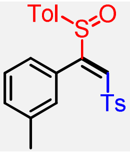

(*E*)-1-Methyl-3-(1-(*p*-tolylsulfinyl)-2-tosylvinyl)benzene (24) White Solid; mp: 157-158 °C; **^{1}H-NMR** (600 MHz, $CDCl_3$) δ 7.54 (d, *J* = 8.4 Hz, 2H), 7.31 (s, 1H), 7.20 (d, *J* = 8.4 Hz, 2H), 7.15 – 7.08 (m, 6H), 6.68 (d, *J* = 7.2 Hz, 1H), 6.64 (s, 1H), 2.39 (s, 3H), 2.33 (s, 3H), 2.23 (s, 3H); **^{13}C-NMR** (150 MHz, $CDCl_3$) δ 160.8, 144.6, 142.6, 137.7, 137.5, 136.9, 130.7, 129.7, 129.5, 129.4, 129.3, 127.93, 127.86, 126.4, 125.4, 21.5, 21.4, 21.1; **HRMS** (ESI) *m/z* calculated $C_{23}H_{22}NaO_3S_2$ $[M+Na]^+$ 433.0903, found 433.0898.

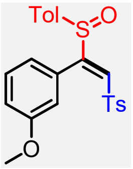

(*E*)-1-Methoxy-3-(1-(*p*-tolylsulfinyl)-2-tosylvinyl)benzene (25) White Solid; mp: 153-154 °C; **^{1}H-NMR** (600 MHz, $CDCl_3$) δ 7.56 (d, *J* = 7.8 Hz, 2H), 7.32 (s, 1H), 7.22 (d, *J* = 7.8 Hz, 2H), 7.15 (t, *J* = 7.8 Hz, 1H), 7.13 – 7.11 (m, 4H), 6.88 (dd, *J* = 8.4, 2.4 Hz, 1H), 6.50 (d, *J* = 7.2 Hz, 1H), 6.37 – 6.35 (m, 1H), 3.66 (s, 3H), 2.40 (s, 3H), 2.34 (s, 3H); **^{13}C-NMR** (150 MHz, $CDCl_3$) δ 160.4, 158.9, 144.8, 142.7, 137.4, 137.0, 129.8, 129.63, 129.59, 129.3, 129.1, 127.9, 125.5, 121.5, 116.3, 114.2, 55.2, 21.6, 21.4; **HRMS** (ESI) *m/z* calculated $C_{23}H_{22}NaO_4S_2$ $[M+Na]^+$ 449.0852, found 449.0851.

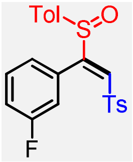

(*E*)-1-Fluoro-3-(1-(*p*-tolylsulfinyl)-2-tosylvinyl)benzene (26) White Solid; mp: 166-167 °C; **^{1}H NMR** (600 MHz, $CDCl_3$) δ 7.59 (d, *J* = 8.4 Hz, 2H), 7.34 (s, 1H), 7.26 (d, *J* = 7.8 Hz, 2H), 7.23 (dd, *J* = 7.8, 5.4 Hz, 1H), 7.16 – 7.12 (m, 4H), 7.07 – 7.03 (m, 1H), 6.73 – 6.70 (m, 1H), 6.61 – 6.57 (m, 1H), 2.42 (s, 3H), 2.35 (s, 3H); **^{13}C-NMR** (150 MHz, $CDCl_3$) δ 161.9 (d, *J* = 250.0 Hz), 159.0 (d, *J* = 3.0 Hz), 145.1, 143.1, 137.3, 136.6, 130.3, 130.2 (d, *J* = 9.0 Hz) , 130.0, 129.82, 129.76, 127.9, 125.5, 125.2 (d, *J* = 3.0 Hz), 117.0 (d, *J* = 21.0 Hz), 116.2 (d, *J* = 22.5 Hz), 21.6, 21.5; **HRMS** (ESI) *m/z* calculated $C_{22}H_{19}FNaO_3S_2$ $[M+Na]^+$ 437.0652, found 437.0657.

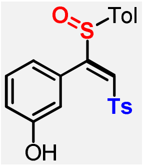

(*E*)-3-(1-(*p*-tolylsulfinyl)-2-tosylvinyl)phenol (27) White Solid; mp: 165-166 °C; **^{1}H-NMR** (600 MHz, $CDCl_3$) δ 7.58 (d, *J* = 8.4 Hz, 2H), 7.29 (s, 1H), 7.22 (d, *J* = 7.8 Hz, 2H), 7.14 – 7.08 (m, 4H), 7.04 (t, *J* = 7.8 Hz, 1H), 6.79 – 6.76 (m, 1H), 6.48 (s, 1H), 6.42 – 6.40 (m, 1H), 6.39 – 6.38 (m, 1H), 2.39 (s, 3H), 2.31 (s, 3H); **^{13}C-NMR** (150 MHz, $CDCl_3$) δ 160.1, 155.8, 145.0, 142.9, 137.1, 136.4, 129.9, 129.8, 129.4, 129.2, 128.9, 127.9, 125.5, 121.1, 117.5, 116.0, 21.6, 21.4; **HRMS** (ESI) *m/z* calculated $C_{22}H_{20}NaO_4S_2$ $[M+Na]^+$ 435.0695, found 435.0670.

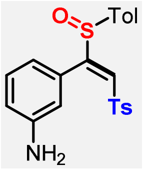

(*E*)-3-(1-(*p*-tolylsulfinyl)-2-tosylvinyl)aniline (28) White Solid; mp: 162-163 °C; **^{1}H-NMR** (600 MHz, $CDCl_3$) δ 7.59 (d, *J* = 8.4 Hz, 2H), 7.26 (s, 1H), 7.23 (d, *J* = 8.4 Hz, 2H), 7.14 – 7.10 (m, 4H), 7.01 (t, *J* = 7.8 Hz, 1H), 6.66 – 6.63 (m, 1H), 6.30 (d, *J* = 7.8 Hz, 1H), 6.25 – 6.23 (m, 1H), 3.65 (br., 2H), 2.41 (s, 3H), 2.34 (s, 3H); **^{13}C-NMR** (150 MHz, $CDCl_3$) δ 160.7, 146.1, 144.7, 142.5, 137.5, 137.1, 129.7, 129.6, 128.98, 128.96, 128.0, 125.4, 119.2, 116.7, 115.5, 21.6, 21.5; **HRMS** (ESI) *m/z* calculated $C_{22}H_{21}NNaO_3S_2$ $[M+Na]^+$ 434.0855, found 434.0833.

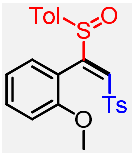

(*E*)-1-Methoxy-2-(1-(*p*-tolylsulfinyl)-2-tosylvinyl)benzene (29) White Solid; mp: 138-139 °C; **^{1}H-NMR** (400 MHz, $CDCl_3$) δ 7.52 (d, *J* = 8.4 Hz, 2H), 7.40 (s, 1H), 7.32 – 7.27 (m, 1H), 7.18 (d, *J* = 8.4 Hz, 2H), 7.14 – 7.08 (m, 4H), 6.78 (t, *J* = 7.2 Hz, 1H), 6.67 (d, *J* = 8.4 Hz, 1H), 6.57 (s, 1H), 3.45 (s, 3H), 2.39 (s, 3H), 2.34 (s, 3H); **^{13}C-NMR** (150 MHz, $CDCl_3$) δ 156.2, 144.3, 142.3, 137.3, 131.6, 131.0, 129.7, 129.34, 129.27, 127.9, 125.6, 119.8, 116.7, 110.2, 54.9, 21.5, 21.4; **HRMS** (ESI) *m/z* calculated $C_{23}H_{22}NaO_4S_2$ $[M+Na]^+$ 449.0852, found 449.0852.

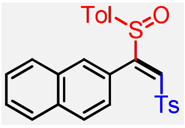

(*E*)-2-(1-(*p*-Tolylsulfinyl)-2-tosylvinyl)naphthalene (30) White Solid; mp: 168-169 °C; **^{1}H-NMR** (600 MHz, $CDCl_3$) δ 7.80 (d, *J* = 7.8 Hz, 1H), 7.72 (d, *J* = 7.8 Hz, 1H), 7.66 (d, *J* = 8.4 Hz, 1H), 7.55 – 7.49 (m, 4H), 7.42 – 7.40 (m, 2H), 7.10 (d, *J* = 8.4 Hz, 4H), 7.06 (d, *J* = 8.4 Hz, 2H), 6.93 (dd, *J* = 8.4, 1.8 Hz, 1H), 2.32 (s, 3H), 2.30 (s, 3H); **^{13}C-NMR** (150 MHz, $CDCl_3$) δ 160.4, 144.8, 142.7, 137.3, 136.8, 133.5, 132.1, 129.83, 129.81, 129.5, 128.8, 128.3, 127.9, 127.74, 127.73, 127.4, 126.7, 126.4, 125.7, 125.5, 21.5, 21.4; **HRMS** (ESI) *m/z* calculated $C_{26}H_{22}NaO_3S_2$ $[M+Na]^+$ 469.0903, found 469.0895.

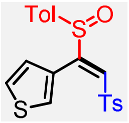

(*E*)-3-(1-(*p*-Tolylsulfinyl)-2-tosylvinyl)thiophene (31) White Solid; mp: 163-164 °C; **^{1}H-NMR** (600 MHz, $CDCl_3$) δ 7.58 (d, *J* = 8.4 Hz, 2H), 7.32 (s, 1H), 7.26 – 7.21 (m, 4H), 7.18 – 7.10 (m, 4H), 6.84 (d, *J* = 4.8 Hz, 1H), 2.40 (s, 3H), 2.33 (s, 3H); **^{13}C-NMR** (150 MHz, $CDCl_3$) δ 155.9, 144.8, 142.6, 137.44, 137.35, 129.9, 129.6, 129.1, 128.4, 127.9, 127.8, 127.7, 126.0, 125.2, 21.6, 21.4; **HRMS** (ESI) *m/z* calculated $C_{20}H_{18}NaO_3S_3$ $[M+Na]^+$ 425.0310, found 425.0316.

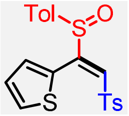

(*E*)-2-(1-(*p*-Tolylsulfinyl)-2-tosylvinyl)thiophene (32) White Solid; mp: 156-157 °C; **^{1}H-NMR** (600 MHz, $CDCl_3$) δ 7.66 (d, *J* = 8.4 Hz, 2H), 7.48 (dd, *J* = 5.4, 1.2 Hz, 1H), 7.33 (s, 1H), 7.26 (d, *J* = 8.4 Hz, 2H), 7.21 (d, *J* = 8.4 Hz, 2H), 7.16 – 7.13 (m, 3H), 7.03 (dd, *J* = 5.4, 4.2 Hz, 1H), 2.42 (s, 3H), 2.34 (s, 3H); **^{13}C-NMR** (150 MHz, $CDCl_3$) δ 153.8, 144.9, 142.8, 137.8, 137.3, 132.1, 130.5, 130.0, 129.8, 129.4, 127.9, 127.7, 127.5, 125.3, 21.6, 21.5; **HRMS** (ESI) *m/z* calculated $C_{20}H_{18}NaO_3S_3$ $[M+Na]^+$ 425.0310, found 425.0306.

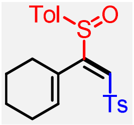

(*E*)-1-((2-(cyclohex-1-en-1-yl)-2-(*p*-tolylsulfinyl)vinyl)sulfonyl)-4-methylbenzene (33) White Solid; mp: 141-142 °C; **^{1}H-NMR** (600 MHz, $CDCl_3$) δ 7.77 (d, *J* = 7.8 Hz, 2H), 7.44 (d, *J* = 7.8 Hz, 2H), 7.33 (d, *J* = 7.8 Hz, 2H), 7.26 (d, *J* = 7.8 Hz, 2H), 6.95 (s, 1H), 5.59 – 5.56 (m, 1H), 2.44 (s, 3H), 2.40 (s, 3H), 2.33 – 2.26 (m, 1H), 2.11 – 2.00 (m, 2H), 1.58 – 1.49 (m, 2H), 1.42 – 1.40 (m, 1H), 1.32 – 1.29 (m, 1H), 1.03 – 1.00 (m, 1H); **^{13}C-NMR** (150 MHz, $CDCl_3$) δ 162.0, 144.8, 142.9, 138.0, 137.7, 132.8, 129.91, 129.86, 128.6, 127.9, 126.9, 126.1, 29.4, 25.4, 22.0, 21.7, 21.6, 21.1; **HRMS** (ESI) *m/z* calculated $C_{22}H_{24}NaO_3S_2$ $[M+Na]^+$ 423.1059, found 423.1043.

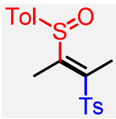

(*E*)-1-methyl-4-((3-(*p*-tolylsulfinyl)but-2-en-2-yl)sulfonyl)benzene (34) White Solid; mp: 81-82 °C; **^{1}H-NMR** (600 MHz, $CDCl_3$) δ 7.73 (d, *J* = 7.2 Hz, 2H), 7.38 – 7.33 (m, 4H), 7.29 (d, *J* = 7.8 Hz, 2H), 2.46 (s, 3H), 2.42 (s, 3H), 2.41 (s, 3H), 2.20 (s, 3H); **^{13}C-NMR** (150 MHz, $CDCl_3$) δ 153.5, 145.2, 141.9, 140.0, 138.4, 136.5, 130.2, 130.1, 127.7, 123.9, 21.7, 21.4, 16.7, 8.7; **HRMS** (ESI) *m/z* calculated $C_{18}H_{20}NaO_3S_2$ $[M+Na]^+$ 371.0746, found 371.0737.

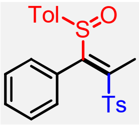

(*E*)-1-Methyl-4-((1-phenyl-1-(*p*-tolylsulfinyl)prop-1-en-2-yl)sulfonyl)benzene (35) White Solid; mp: 143-144 °C; **^{1}H-NMR** (600 MHz, $CDCl_3$) δ 7.29 – 7.24 (m, 3H), 7.18 – 7.12 (m, 3H), 7.11 (d, *J* = 8.4 Hz, 2H), 7.03 – 7.69 (m, 3H), 6.76 (d, *J* = 6.6 Hz, 1H), 6.21 (d, *J* = 6.6 Hz, 1H), 2.72 (s, 3H), 2.38 (s, 3H), 2.37 (s, 3H); **^{13}C-NMR** (150 MHz, $CDCl_3$) δ 153.6, 144.5, 144.0, 142.0, 137.8, 136.5, 131.0, 129.6, 129.4, 128.8, 128.0, 126.7, 126.5, 124.3, 21.6, 21.4, 15.7; **HRMS** (ESI) *m/z* calculated $C_{23}H_{22}NaO_3S_2$ $[M+Na]^+$ 433.0903, found 433.0896.

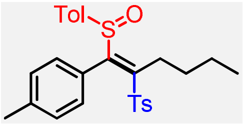

(*E*)-1-Methyl-4-((1-(*p*-tolyl)-1-(*p*-tolylsulfinyl)hex-1-en-2-yl)sulfonyl)be nzene (36) White Solid; mp: 140-141 °C; **^{1}H-NMR** (600 MHz, $CDCl_3$) δ 7.13 (d, *J* = 8.0 Hz, 4H), 7.00 (d, *J* = 8.0 Hz, 4H), 6.81 (d, *J* = 5.4 Hz, 1H), 6.72 (d, *J* = 5.4 Hz, 1H), 6.52 (d, *J* = 6.0 Hz, 1H), 6.04 (d, *J* = 6.0 Hz, 1H), 3.26 – 3.19 (m, 1H), 3.17 – 3.11 (m, 1H), 2.36 (s, 3H), 2.35 (s, 3H), 2.27 (s, 3H), 2.05 – 1.96 (m, 1H), 1.84 – 1.76 (m, 1H), 1.63 – 1.54 (m, 2H), 1.05 (t, *J* = 7.2 Hz, 3H); **^{13}C-NMR** (150 MHz, $CDCl_3$) δ 153.2, 150.1, 143.9, 141.8, 138.6, 137.7, 137.3, 131.0, 130.8, 129.5, 129.0, 127.8, 127.2, 127.1, 124.6, 123.6, 33.2, 29.2, 23.0, 21.5, 21.41, 21.36, 13.7; **HRMS** (ESI) *m/z* calculated $C_{27}H_{30}NaO_3S_2$ $[M+Na]^+$ 489.1529 found 489.1526.

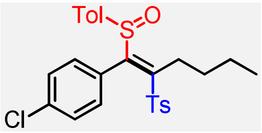

(*E*)-1-Chloro-4-(1-(*p*-tolylsulfinyl)-2-tosylhex-1-en-1-yl)benzene (37) White Solid; mp: 132-133 °C; **^{1}H-NMR** (600 MHz, $CDCl_3$) δ 7.15 (d, *J* = 7.8 Hz, 4H), 7.06 (d, *J* = 7.8 Hz, 2H), 6.99 (d, *J* = 7.8 Hz, 2H), 6.96 (d, *J* = 7.8 Hz, 1H), 6.89 (d, *J* = 7.8 Hz, 1H), 6.53 (d, *J* = 7.2 Hz, 1H), 6.10 (d, *J* = 7.2 Hz, 1H), 3.30 – 3.20 (m, 1H), 3.17 – 3.10 (m, 1H), 2.37 (s, 3H), 2.36 (s, 3H), 2.08 – 1.96 (m, 1H), 1.85 – 1.75 (m, 1H), 1.63 – 1.57 (m, 2H), 1.06 (t,

J = 7.2 Hz, 3H); **^{13}C-NMR** (150 MHz, $CDCl_3$) δ 151.6, 151.0, 144.4, 142.1, 137.2, 137.0, 135.1, 132.5, 131.9, 129.7, 129.2, 127.7, 126.7, 126.6, 125.0, 124.4, 33.2, 29.3, 23.0, 21.6, 21.4, 13.7; **HRMS** (ESI) *m/z* calculated $C_{26}H_{27}ClNaO_3S_2$ $[M+Na]^+$ 509.0982, found 509.0981.

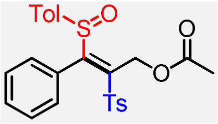

(*E*)-3-Phenyl-3-(*p*-tolylsulfinyl)-2-tosylallyl acetate (38) White Solid; mp: 134-135 °C; **^{1}H-NMR** (600 MHz, $CDCl_3$) δ 7.28 (t, J = 7.8 Hz, 1H), 7.22 (d, J = 8.4 Hz, 2H), 7.16 (d, J = 7.8 Hz, 2H), 7.14 – 7.10 (m, 1H), 7.09 (d, J = 7.8 Hz, 2H), 7.06 (d, J = 8.4 Hz, 2H), 7.03 – 6.97 (m, 1H), 6.70 (s, 1H), 6.21 (s, 1H), 5.66 (d, J =12.6 Hz, 1H), 5.57 (d, J = 12.6 Hz, 1H), 2.38 (s, 3H), 2.37 (s, 3H), 2.20 (s, 3H); **^{13}C-NMR** (150 MHz, $CDCl_3$) δ 169.9, 160.5, 144.7, 142.3, 142.1, 136.74, 136.66, 130.4, 129.7, 129.4, 129.2, 128.1, 126.5, 125.7, 124.8, 58.1, 21.6, 21.4, 20.9; **HRMS** (ESI) *m/z* calculated $C_{25}H_{24}NaO_5S_2$ $[M+Na]^+$ 491.0957, found 491.0948.

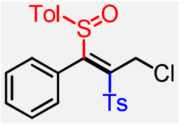

(*E*)-1-((3-Chloro-1-phenyl-1-(*p*-tolylsulfinyl)prop-1-en-2-yl)sulfonyl)-4-methylbenzene (39) White Solid; mp: 109-110 °C; **^{1}H-NMR** (600 MHz, $CDCl_3$) δ 7.25 (t, J = 7.8 Hz, 1H), 7.16 (d, J = 8.4 Hz, 2H), 7.13 (d, J = 8.4 Hz, 2H), 7.11 – 7.05 (m, 3H), 7.02 (d, J = 7.8 Hz, 2H), 6.93 (t, J = 7.8 Hz, 1H), 6.65 (d, J = 6.0 Hz, 1H), 6.12 (d, J = 6.0 Hz, 1H), 5.28 (d, J = 12.0 Hz, 1H), 5.25 (d, J = 12.0 Hz, 1H), 2.35 (s, 6H); **^{13}C-NMR** (150 MHz, $CDCl_3$) δ 158.4, 144.8, 144.6, 142.4, 136.6, 136.2, 130.8, 130.4, 129.7, 129.3, 129.2, 128.1, 126.6, 126.5, 125.6, 125.0, 36.4, 21.6, 21.5; **HRMS** (ESI) *m/z* calculated $C_{23}H_{21}ClNaO_3S_2$ $[M+Na]^+$ 467.0513, found 467.0507.

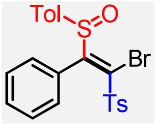

(*Z*)-1-((2-Bromo-1-phenyl-2-tosylvinyl)sulfinyl)-4-methylbenzene (40) White Solid; mp: 174-175 °C; **^{1}H-NMR** (600 MHz, $CDCl_3$) δ 7.46 (d, J = 8.4 Hz, 2H), 7.28 – 7.18 (m, 8H), 7.10 (d, J = 5.4 Hz, 1H), 6.83 (d, J = 5.4 Hz, 1H), 6.21 (d, J = 5.4 Hz, 1H), 2.44 (s, 3H), 2.39 (s, 3H); **^{13}C-NMR** (150 MHz, $CDCl_3$) δ 156.7, 145.9, 142.8, 137.7, 136.2, 134.5, 131.8, 131.5, 129.9, 129.7, 128.9, 128.3, 127.3, 127.1, 125.2, 124.2, 21.7, 21.5; **HRMS** (ESI) *m/z* calculated $C_{22}H_{19}BrNaO_3S_2$ $[M+Na]^+$ 496.9851, found 496.9852.

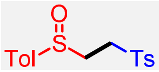

1-methyl-4-((2-(*p*-tolylsulfinyl)ethyl)sulfonyl)benzene (41) White Solid; **^{1}H-NMR** (600 MHz, $CDCl_3$) δ 7.73 (d, J = 7.8 Hz, 2H), 7.41 (d, J = 7.8 Hz, 2H), 7.37 – 7.31 (m, 4H), 3.53 – 3.47 (m, 1H), 3.30 – 2.25 (m, 1H), 3.05 (td, J = 13.2, 3.6 Hz, 1H), 2.97 – 2.92 (m, 1H), 2.46 (s, 3H), 2.43 (s, 3H); **^{13}C-NMR** (150 MHz, $CDCl_3$) δ 145.4, 142.2, 138.5, 135.5, 130.3, 130.2, 128.1, 123.9, 48.2, 47.9, 21.7, 21.4; **HRMS** (ESI) *m/z* calculated $C_{16}H_{18}NaO_3S_2$ $[M+Na]^+$ 345.0590, found 345.0595.

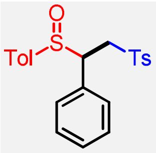

dr = 1 : 0.6

1-methyl-4-(((2*R*)-2-phenyl-2-(*p*-tolylsulfinyl)ethyl)sulfonyl)benzene (42) White Solid; dr = 1 : 0.6. **^{1}H-NMR** (500 MHz, $CDCl_3$) δ 7.55 (d, J = 8.0 Hz, major, 2H), 7.45 (d, J = 8.0 Hz, minor, 2H), 7.24 – 7.04 (m, major, 7H, minor, 9H), 6.90 (d, J = 7.5 Hz, minor, 2H), 6.84 (d, J = 8.0 Hz, major, 2H), 6.69 (d, J = 7.5 Hz, major, 2H), 4.38 (dd, J = 10.5, 3.5 Hz, major, 1H), 4.13 – 4.01 (m, major, 1H, minor, 1H), 3.93 (dd, J = 14.5, 3.0 Hz, minor, 1H), 3.84 (dd, J = 14.5, 11.5 Hz, minor, 1H), 3.58 (dd, J = 14.5, 10.5 Hz, major, 1H), 2.39 – 2.34 (m, major, 6H, minor, 6H). **^{13}C-NMR** (125 MHz, $CDCl_3$) δ 144.8, 144.6, 142.3, 142.2, 137.7, 136.4, 136.2, 135.5, 131.5, 129.73, 129.70, 129.6, 129.22, 129.19, 128.7, 128.62, 128.56, 128.1, 128.0, 127.9, 125.0, 124.9, 66.8, 63.7, 54.9, 52.9, 21.59, 21.56, 21.50, 21.45; **HRMS** (ESI) *m/z* calculated $C_{22}H_{23}O_3S_2$ $[M+H]^+$ 399.1083, found 399.1093.

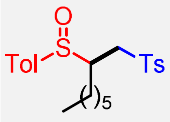

dr = 1 : 0.75

1-methyl-4-(((2*R*)-2-(*p*-tolylsulfinyl)octyl)sulfonyl)benzene (43): White Solid; dr = 1 : 0.75. **^{1}H-NMR** (500 MHz, $CDCl_3$) δ 7.77 (d, J = 8.5 Hz, minor, 2H), 7.49 (d, J = 8.0 Hz, major, 2H), 7.38 –7.36 (m, minor, 4H), 7.31 – 7.22 (m, major, 6H, minor, 2H), 3.76 (dd, J = 14.5, 3.0 Hz, minor, 1H), 3.24 – 3.10 (m, major, 2H, minor, 1H), 2.86 – 2.84 (m, major, 1H), 2.72 (dd, J = 14.0, 9.0 Hz, minor, 1H), 2.46 – 2.42 (m, major, 6H, minor, 6H), 2.07 – 2.02 (m, major, 2H), 1.93 – 1.90 (m, minor, 1H), 1.73 – 1.19 (m, major, 8H, minor, 9H), 0.92 –

0.86 (m, major, 3H, minor, 3H). **^{13}C-NMR** (125 MHz, $CDCl_3$) δ145.2, 144.8, 142.3, 141.6, 137.9, 136.2, 135.7, 130.1, 130.0, 129.9, 128.1, 127.7, 127.6, 125.2, 124.2, 59.5, 57.8, 52.61, 52.56, 31.5, 31.4, 29.5, 28.90, 28.86, 27.2, 26.8, 26.4, 22.6, 22.5, 21.70, 21.66, 21.47, 21.46, 14.1, 14.0; **HRMS** (ESI) *m/z* calculated $C_{22}H_{30}NaO_3S_2$ $[M+Na]^+$ 429.1529, found 429.1556.

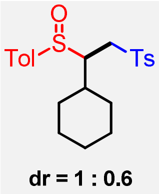

1-(((2*R*)-2-cyclohexyl-2-(*p*-tolylsulfinyl)ethyl)sulfonyl)-4-methylbenzene (44) White Solid; dr = 1 : 0.6. **^{1}H-NMR** (600 MHz, $CDCl_3$) δ 7.68 (d, J = 7.8 Hz, minor, 2H), 7.45–7.43 (m, major, 2H, minor, 2H), 7.32 (d, J = 7.8 Hz, minor, 2H), 7.29–7.22 (m, major, 4H, minor, 2H), 7.20 (d, J = 7.8 Hz, major, 2H), 3.54 (dd, J = 14.4, 3.6 Hz, minor, 1H), 3.33–3.24 (m, major, 2H), 3.17–3.15 (m, minor, 1H), 3.11 (dd, J = 14.4, 6.6 Hz, minor, 1H), 2.87–2.85 (m, major, 1H), 2.46–2.40 (m, major, 6H, minor, 6H), 2.16–2.13 (m, major, 1H), 2.01–1.98 (m, major, 1H), 1.90–1.77 (m, major, 2H, minor, 2H), 1.76–1.60 (m, major, 3H, minor, 2H), 1.48–1.44 (m, major, 1H), 1.40–1.35 (m, major, 1H, minor, 1H), 1.28–1.20 (m, major, 2H, minor, 6H). **^{13}C-NMR** (150 MHz, $CDCl_3$) δ 145.0, 144.6, 142.3, 141.2, 139.0, 137.4, 136.5, 136.3, 130.0, 129.92, 129.85, 129.77, 128.1, 127.7, 125.5, 124.0, 65.5, 62.7, 51.0, 49.3, 39.4, 36.8, 32.1, 31.5, 29.5, 29.0, 26.40, 26.35, 26.28, 26.12, 26.11, 25.9, 21.7, 21.6, 21.51, 21.46; **HRMS** (ESI) *m/z* calculated $C_{22}H_{28}NaO_3S_2$ $[M+Na]^+$ 427.1372, found 427.1373.

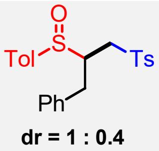

1-methyl-4-(((2*R*)-3-phenyl-2-(*p*-tolylsulfinyl)propyl)sulfonyl)benzene (45) White Solid; dr = 1 : 0.4. **^{1}H-NMR** (600 MHz, $CDCl_3$) δ 7.67 (d, J = 7.8 Hz, minor, 2H), 7.51 (d, J = 7.8 Hz, major, 2H), 7.43 (d, J = 7.2 Hz, minor, 2H), 7.38 – 7.34 (m, major, 3H, minor, 2H), 7.32 – 7.28 (m, major, 2H, minor, 2H), 7.28 – 7.25 (m, minor, 2H), 7.25 – 7.21 (m, major, 2H, minor, 1H), 7.18 (d, J = 7.8 Hz, major, 2H), 7.13 (d, J = 7.8 Hz, major, 2H), 7.08 (d, J = 7.2 Hz, minor, 2H), 3.63 (dd, J = 15.0, 5.4 Hz, minor, 1H), 3.51 (dd, J = 14.4, 3.6 Hz, major, 1H), 3.43 – 3.41 (m, minor, 1H), 3.26 – 3.18 (m, major, 3H, minor, 1H), 3.09 – 3.06 (m, major, 1H), 2.99 (dd, J = 15.0, 6.2 Hz, minor, 1H), 2.74 (dd, J = 15.0, 6.6 Hz, minor, 1H), 2.44 – 2.39 (m, major, 6H, minor, 6H). **^{13}C-NMR** (150 MHz, $CDCl_3$) δ 145.2, 144.9, 142.2, 141.5, 137.5, 137.1, 136.6, 135.9, 130.1, 130.00, 129.96, 129.6, 129.3, 128.9, 128.8, 128.0, 127.7, 127.2, 127.1, 125.1, 124.1, 61.3, 59.2, 52.4, 35.4, 31.0, 21.70, 21.67, 21.48, 21.45; **HRMS** (ESI) *m/z* calculated $C_{23}H_{24}NaO_3S_2$ $[M+Na]^+$ 435.1059, found 435.1056.

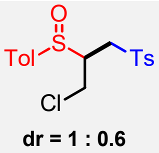

1-(((2*S*)-3-chloro-2-(*p*-tolylsulfinyl)propyl)sulfonyl)-4-methylbenzene (46) White Solid; dr = 1 : 0.6. **^{1}H-NMR** (500 MHz, $CDCl_3$) δ 7.65 (d, J = 8.0 Hz, minor, 2H), 7.60 (d, J = 8.0 Hz, major, 2H), 7.44 (d, J = 8.5 Hz, minor, 2H), 7.39 (d, J = 8.5 Hz, major, 2H), 7.33 – 7.26 (m, major, 4H, minor, 4H), 4.25 (dd, J = 12.0, 4.5 Hz, minor, 1H), 4.16 – 4.07 (m, major, 1H), 3.94 (dd, J = 12.5, 3.5 Hz, major, 1H), 3.65 (dd, J = 12.5, 6.5 Hz, minor, 1H), 3.48 – 3.36 (m, major, 2H, minor, 2H), 3.27 – 3.13 (m, major, 1H, minor, 1H), 2.46 – 2.42 (m, major, 6H, minor, 6H). **^{13}C-NMR** (125 MHz, $CDCl_3$) δ 145.5, 145.3, 143.2, 142.5, 137.2, 135.5, 135.4, 135.3, 130.3, 130.24, 130.19, 130.1, 128.0, 127.8, 125.4, 124.5, 62.1, 60.0, 50.7, 50.2, 41.4, 40.6, 21.72, 21.71, 21.6, 21.5; **HRMS** (ESI) *m/z* calculated $C_{17}H_{19}ClNaO_3S_2$ $[M+Na]^+$ 393.0356, found 393.0360.

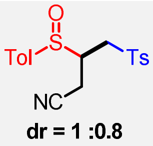

(3*R*)-3-(*p*-tolylsulfinyl)-4-tosylbutanenitrile (47) White Solid; dr = 1 : 0.8. **^{1}H-NMR** (600 MHz, $CDCl_3$) δ 7.68 (d, J = 7.8 Hz, minor, 2H), 7.55 (d, J = 7.8 Hz, major, 2H), 7.40 (d, J = 7.8 Hz, minor, 2H), 7.34 – 7.30 (m, major, 2H, minor, 2H), 7.28 (d, J = 7.8 Hz, minor, 2H), 7.25 (d, J = 7.8 Hz, major, 4H), 3.56 (dd, J = 14.4, 3.6 Hz, minor, 1H), 3.36 – 3.34 (m, minor, 1H), 3.29 – 3.22 (m, major, 1H), 3.19 – 2.98 (m, major, 4H, minor, 2H), 2.58 (dd, J = 17.4, 7.2 Hz, minor, 1H), 2.43 – 2.34 (m, major, 6H, minor, 6H). **^{13}C-NMR** (150 MHz, $CDCl_3$) δ 145.8, 145.6, 143.5, 143.1, 136.1, 135.3, 135.22, 135.18, 130.45, 130.42, 130.3, 130.2, 128.0, 127.8, 124.9, 124.5, 115.8, 115.6, 55.2, 53.7, 52.4, 51.6, 21.67, 21.65, 21.5, 17.4, 15.3; **HRMS** (ESI) *m/z* calculated $C_{18}H_{19}NNaO_3S_2$ $[M+Na]^+$ 384.0699, found 384.0693.

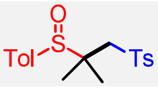

1-methyl-4-((2-methyl-1-tosylpropan-2-yl)sulfinyl)benzene (48) White Solid; **^{1}H-NMR** (500 MHz, $CDCl_3$) δ 7.75 (d, J = 8.5 Hz, 2H), 7.41 (d, J = 8.0 Hz, 2H), 7.35 (d, J = 8.5 Hz, 2H), 7.30 (d, J = 8.0 Hz, 2H), 3.53 (d, J = 14.0 Hz, 1H), 2.96 (d, J = 14.0 Hz, 1H), 2.45 (s,

3H), 2.42 (s, 3H), 1.62 (s, 3H), 1.36 (s, 3H). ^{13}**C-NMR** (125 MHz, $CDCl_3$) δ 144.9, 142.7, 138.4, 134.8, 130.0, 129.6, 127.6, 126.4, 58.9, 58.1, 21.7, 21.5, 21.4, 20.0; **HRMS** (ESI) *m/z* calculated $C_{18}H_{22}NaO_3S_2$ $[M+Na]^+$ 373.0903, found 373.0913.

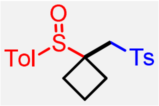

1-methyl-4-(((1-(*p*-tolylsulfinyl)cyclobutyl)methyl)sulfonyl)benzene (49) White Solid; ^{1}**H-NMR** (500 MHz, $CDCl_3$) δ 7.78 (d, *J* = 8.5 Hz, 2H), 7.55 (d, *J* = 8.5 Hz, 2H), 7.36 (d, *J* = 8.5 Hz, 2H), 7.30 (d, *J* = 8.0 Hz, 2H), 3.62 (d, *J* = 14.5 Hz, 1H), 2.92 (d, *J* = 14.5 Hz, 1H), 2.78 – 2.71 (m, 1H), 2.63 – 2.57 (m, 1H), 2.50 – 2.48 (m, 1H), 2.45 (s, 3H), 2.40 (s, 3H), 2.39 – 2.33 (m, 1H), 2.08 – 2.02 (m, 1H), 1.95 – 1.87 (m, 1H). ^{13}**C-NMR** (125 MHz, $CDCl_3$) δ 145.1, 142.3, 137.9, 135.8, 130.1, 129.7, 127.8, 125.9, 61.3, 56.7, 25.5, 25.3, 21.7, 21.5, 15.2; **HRMS** (ESI) *m/z* calculated $C_{19}H_{22}NaO_3S_2$ $[M+Na]^+$ 385.0903, found 385.0908.

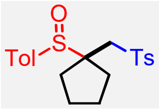

1-methyl-4-(((1-(*p*-tolylsulfinyl)cyclopentyl)methyl)sulfonyl)benzene (50) White Solid; ^{1}**H-NMR** (500 MHz, $CDCl_3$) δ 7.73 (d, *J* = 8.0 Hz, 2H), 7.47 (d, *J* = 8.0 Hz, 2H), 7.34 (d, *J* = 8.0 Hz, 2H), 7.29 (d, *J* = 8.0 Hz, 2H), 3.70 (d, *J* = 14.0 Hz, 1H), 2.96 (d, *J* = 14.0 Hz, 1H), 2.50 – 2.43 (m, 4H), 2.41 (s, 3H), 2.36 – 2.32 (m, 1H), 2.30 – 2.26 (m, 1H), 1.93 – 1.86 (m, 3H), 1.75 – 1.67 (m, 2H); ^{13}**C-NMR** (125 MHz, $CDCl_3$) δ 144.9, 142.5, 138.5, 135.7, 130.0, 129.7, 127.5, 126.4, 68.6, 56.1, 31.4, 31.2, 25.6, 25.2, 21.7, 21.5; **HRMS** (ESI) *m/z* calculated $C_{20}H_{24}NaO_3S_2$ $[M+Na]^+$ 399.1059, found 399.1051.

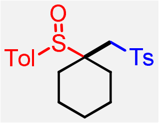

1-methyl-4-(((1-(*p*-tolylsulfinyl)cyclohexyl)methyl)sulfonyl)benzene (51) White Solid; ^{1}**H-NMR** (500 MHz, $CDCl_3$) δ 7.72 (d, *J* = 8.5 Hz, 2H), 7.40 (d, *J* = 8.0 Hz, 2H), 7.33 (d, *J* = 8.0 Hz, 2H), 7.29 (d, *J* = 8.0 Hz, 2H), 3.69 (d, *J* = 14.5 Hz, 1H), 2.62 (d, *J* = 14.5 Hz, 1H), 2.47 – 2.40 (m, 7H), 2.31 – 2.26 (m, 1H), 2.20 – 2.17 (m, 1H), 1.98 – 1.93 (m, 1H), 1.79 – 1.45 (m, 6H); ^{13}**C-NMR** (125 MHz, $CDCl_3$) δ 144.7, 142.2, 138.5, 134.7, 129.9, 129.7, 127.5, 126.3, 63.8, 56.5, 29.8, 28.3, 25.2, 22.0, 21.6, 21.52, 21.45; **HRMS** (ESI) *m/z* calculated $C_{21}H_{26}NaO_3S_2$ $[M+Na]^+$ 413.1216, found 413.1235.

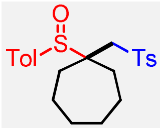

1-(p-tolylsulfinyl)-1-(tosylmethyl)cycloheptane (52) White Solid; ^{1}**H-NMR** (500 MHz, $CDCl_3$) δ 7.71 (d, *J* = 8.5 Hz, 2H), 7.41 (d, *J* = 8.0 Hz, 2H), 7.33 (d, *J* = 8.0 Hz, 2H), 7.27 (d, *J* = 8.0 Hz, 2H), 3.54 (d, *J* = 14.0 Hz, 1H), 2.73 (d, *J* = 14.0 Hz, 1H), 2.65 – 2.60 (m, 1H), 2.44 (s, 3H), 2.41 (s, 3H), 2.33 – 2.28 (m, 1H), 2.01 – 1.96 (m, 1H), 1.86 – 1.79 (m, 2H), 1.77 – 1.66 (m, 5H), 1.57 – 1.54 (m, 1H), 1.49 – 1.44 (m, 1H); ^{13}**C-NMR** (125 MHz, $CDCl_3$) δ 144.7, 142.4, 138.6, 135.0, 129.9, 129.6, 127.4, 126.6, 65.9, 58.7, 33.2, 31.7, 31.2, 30.5, 23.9, 23.2, 21.6, 21.4; **HRMS** (ESI) *m/z* calculated $C_{22}H_{28}NaO_3S_2$ $[M+Na]^+$ 427.1372, found 427.1378.

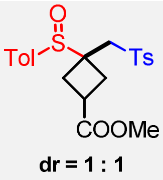

methyl 3-(*p*-tolylsulfinyl)-3-(tosylmethyl)cyclobutanecarboxylate (53): white solid; dr = 1:1. ^{1}**H-NMR** (500 MHz, $CDCl_3$) δ 7.82 (d, *J* = 8.0 Hz, 2H), 7.77 (d, *J* = 8.0 Hz, 2H), 7.67 (d, *J* = 8.0 Hz, 2H), 7.51 (d, *J* = 8.0 Hz, 2H), 7.39 – 7.36 (m, 4H), 7.34 (d, *J* = 8.0 Hz, 2H), 7.30 (d, *J* = 8.0 Hz, 2H), 3.87 (d, *J* = 15.0 Hz, 1H), 3.71 (s, 3H), 3.66 (s, 3H), 3.58 (d, *J* = 14.5 Hz, 1H), 3.39 (p, *J* = 8.5 Hz, 1H), 3.07 – 3.03 (m, 2H), 2.95 – 2.90 (m, 3H), 2.85 – 2.77 (m, 4H), 2.71 – 2.66 (m, 1H), 2.46 (s, 6H), 2.42 – 2.40 (m, 6H), 2.33 – 2.30 (m, 1H). ^{13}**C-NMR** (125 MHz, $CDCl_3$) δ 175.0, 174.0, 145.4, 145.3, 142.62, 142.61, 137.7, 137.5, 135.4, 135.2, 130.20, 130.16, 129.88, 129.87, 128.0, 127.7, 126.1, 125.7, 58.5, 58.4, 57.1, 55.7, 52.11, 52.10, 32.3, 31.2, 29.7, 29.4, 29.1, 28.10, 28.07, 21.7, 21.5; **HRMS** (ESI) *m/z* calculated $C_{21}H_{24}NaO_5S_2$ $[M+Na]^+$ 443.0957, found 443.0948.

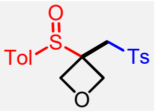

3-(*p*-tolylsulfinyl)-3-(tosylmethyl)oxetane (54) White Solid; **^{1}H-NMR** (500 MHz, $CDCl_3$) δ 7.82 (d, *J* = 8.0 Hz, 2H), 7.64 (d, *J* = 8.0 Hz, 2H), 7.40 (d, *J* = 8.0 Hz, 2H), 7.34 (d, *J* = 8.0 Hz, 2H), 5.05 (d, *J* = 7.5 Hz, 1H), 4.91 (d, *J* = 8.0 Hz, 1H), 4.85 (d, *J* = 8.0 Hz, 1H), 4.56 (d, *J* = 7.5 Hz, 1H), 3.90 (d, *J* = 15.0 Hz, 1H), 3.19 (d, *J* = 15.0 Hz, 1H), 2.47 (s, 3H), 2.42 (s, 3H); **^{13}C-NMR** (125 MHz, $CDCl_3$) δ 145.8, 143.0, 137.0, 135.0, 130.3, 130.1, 127.9, 125.6, 73.0, 72.4, 60.6, 56.2, 21.7, 21.5; **HRMS** (ESI) *m/z* calculated $C_{18}H_{20}NaO_4S_2$ $[M+Na]^+$ 387.0695, found 387.0698.

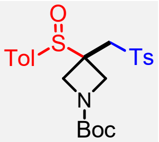

tert-butyl 3-(*p*-tolylsulfinyl)-3-(tosylmethyl)azetidine-1-carboxylate (55) White Solid; **^{1}H-NMR** (600 MHz, $CDCl_3$) δ 7.81 (d, *J* = 7.8 Hz, 2H), 7.63 (d, *J* = 7.8 Hz, 2H), 7.39 (d, *J* = 7.8 Hz, 2H), 7.35 (d, *J* = 7.8 Hz, 2H), 4.36 – 4.10 (m, 4H), 3.75 (d, *J* = 15.0 Hz, 1H), 3.16 (d, *J* = 15.0 Hz, 1H), 2.47 (s, 3H), 2.42 (s, 3H), 1.41 (s, 9H); **^{13}C-NMR** (150 MHz, $CDCl_3$) δ 155.6, 145.7, 143.1, 137.1, 134.9, 130.3, 130.1, 127.9, 125.8, 80.3, 55.2, 52.9, 51.8, 28.2, 21.7, 21.5, 21.0; **HRMS** (ESI) *m/z* calculated $C_{23}H_{29}NNaO_5S_2$ $[M+Na]^+$ 486.1379, found 486.1385.

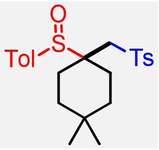

1-(((4,4-dimethyl-1-(*p*-tolylsulfinyl)cyclohexyl)methyl)sulfonyl)-4-methylbenzene (56) White Solid; **^{1}H-NMR** (600 MHz, $CDCl_3$) δ 7.72 (d, *J* = 7.8 Hz, 2H), 7.39 (d, *J* = 7.8 Hz, 2H), 7.33 (d, *J* = 7.8 Hz, 2H), 7.29 (d, *J* = 7.8 Hz, 2H), 3.70 (d, *J* = 14.4 Hz, 1H), 2.63 – 2.57 (m, 2H), 2.47– 2.41 (m, 7H), 2.13 – 2.07 (m, 1H), 1.88 – 1.83 (m, 1H), 1.69 – 1.64 (m, 1H), 1.48 – 1.35 (m, 3H), 1.05 (s, 3H), 1.00 (s, 3H); **^{13}C-NMR** (125 MHz, $CDCl_3$) δ 144.7, 142.3, 138.6, 134.6, 130.0, 129.7, 127.4, 126.3, 63.5, 56.2, 34.8, 34.2, 31.2, 30.0, 25.8, 25.5, 24.3, 21.6, 21.5; **HRMS** (ESI) *m/z* calculated $C_{23}H_{30}NaO_3S_2$ $[M+Na]^+$ 441.1529, found 441.1515.

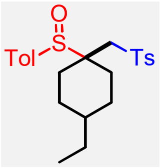

1-(((4-ethyl-1-(*p*-tolylsulfinyl)cyclohexyl)methyl)sulfonyl)-4-methylbenzene (57) White Solid; **^{1}H-NMR** (500 MHz, $CDCl_3$) δ 7.69 (d, *J* = 8.0 Hz, 2H), 7.35 – 7.27 (m, 6H), 3.70 (d, *J* = 14.0 Hz, 1H), 2.57 – 2.48 (m, 3H), 2.43 (s, 3H), 2.40 (s, 3H), 2.32 – 2.28 (m, 1H), 1.84 – 1.77 (m, 1H), 1.74 – 1.67 (m, 2H), 1.62 – 1.59 (m, 1H), 1.48 – 1.41 (m, 2H), 1.38 – 1.32 (m, 2H), 0.93 (t, *J* = 7.0 Hz, 3H); **^{13}C-NMR** (125 MHz, $CDCl_3$) δ 144.6, 142.3, 138.6, 134.6, 129.9, 129.7, 127.4, 126.1, 63.9, 56.3, 38.4, 30.1, 29.5, 28.8, 28.2, 27.6, 21.6, 21.5, 11.4; **HRMS** (ESI) *m/z* calculated $C_{23}H_{30}NaO_3S_2$ $[M+Na]^+$ 441.1529, found 441.1506.

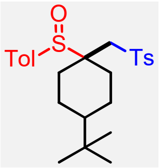

1-(((4-(tert-butyl)-1-(*p*-tolylsulfinyl)cyclohexyl)methyl)sulfonyl)-4-methylbenz ene (58) White Solid; **^{1}H-NMR** (500 MHz, $CDCl_3$) δ 7.69 (d, *J* = 8.5 Hz, 2H), 7.35 – 7.27 (m, 6H), 3.68 (d, *J* = 14.0 Hz, 1H), 2.58 – 2.50 (m, 3H), 2.43 – 2.35 (m, 7H), 1.84 – 1.74 (m, 3H), 1.68 – 1.65 (m, 1H), 1.61 – 1.53 (m, 1H), 1.34 – 1.28 (m, 1H), 0.92 (s, 9H); **^{13}C-NMR** (125 MHz, $CDCl_3$) δ 144.6, 142.3, 138.6, 134.5, 129.9, 129.7, 127.4, 126.1, 63.7, 56.2, 47.1, 32.5, 30.6, 29.4, 27.5, 22.9, 22.3, 21.6, 21.5; **HRMS** (ESI) *m/z* calculated $C_{25}H_{34}NaO_3S_2$ $[M+Na]^+$ 469.1842, found 469.1837.

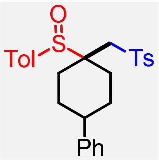

1-methyl-4-(((4-phenyl-1-(*p*-tolylsulfinyl)cyclohexyl)methyl)sulfonyl)benzene (59) White Solid; **^{1}H-NMR** (600 MHz, $CDCl_3$) δ 7.72 (d, *J* = 8.4 Hz, 2H), 7.37 (d, *J* = 8.4 Hz, 2H), 7.35 – 7.32 (m, 4H), 7.29 (d, *J* = 8.4 Hz, 4H), 7.23 (t, *J* = 7.2 Hz, 1H), 3.74 (d, *J* = 13.8 Hz, 1H), 2.83 – 2.71 (m, 3H), 2.58 (d, *J* = 13.8 Hz, 1H), 2.48 – 2.43 (m, 4H), 2.41 (s, 3H), 2.33 – 2.30 (m, 1H), 2.02 – 1.87 (m, 3H), 1.75 – 1.72 (m, 1H); **^{13}C-NMR** (150 MHz, $CDCl_3$) δ 146.0, 144.8, 142.5, 138.6, 134.4, 130.0, 129.9, 128.6, 127.5, 126.8, 126.5, 126.2, 63.3, 56.2, 43.2, 30.5, 29.9, 29.3, 28.6, 21.7, 21.5; **HRMS** (ESI) *m/z* calculated $C_{27}H_{30}NaO_3S_2$ $[M+Na]^+$ 489.1529, found 489.1556.

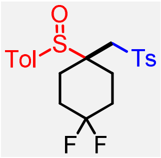

1-(((4,4-difluoro-1-(*p*-tolylsulfinyl)cyclohexyl)methyl)sulfonyl)-4-methylbenzene (60) White Solid; ^{1}**H-NMR** (500 MHz, $CDCl_3$) δ 7.73 (d, J = 8.5 Hz, 2H), 7.39 (d, J = 8.5 Hz, 2H), 7.37-7.31 (m, 4H), 3.68 (d, J = 14.5 Hz, 1H), 2.71 – 2.57 (m, 3H), 2.46 (s, 3H), 2.44 (s, 3H), 2.39 – 2.33 (m, 1H), 2.30 – 2.26 (m, 1H), 2.22 – 2.11 (m, 3H), 1.70 – 1.62 (m, 1H); ^{13}**C-NMR** (125 MHz, $CDCl_3$) δ 145.1, 143.0, 138.0, 133.9, 130.1, 130.0, 127.6, 126.2, 121.78 (t, J = 240.5 Hz), 61.7, 55.3, 30.1 (t, J = 25.0 Hz), 29.7 (t, J = 25.0 Hz), 26.2 (dd, J = 7.5 Hz, 1.5 Hz), 25.1 (dd, J = 7.5 Hz, 1.5Hz), 21.7, 21.5; **HRMS** (ESI) *m/z* calculated $C_{21}H_{25}F_2O_3S_2$ $[M+H]^+$ 427.1208, found 427.1197.

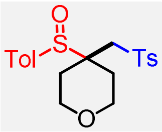

4-(*p*-tolylsulfinyl)-4-(tosylmethyl)tetrahydro-2*H*-pyran (61) White Solid; ^{1}**H-NMR** (600 MHz, $CDCl_3$) δ 7.70 (d, J = 8.4 Hz, 2H), 7.35 (d, J = 8.4 Hz, 2H), 7.29 (d, J = 7.8 Hz, 2H), 7.25 (d, J = 7.8 Hz, 2H), 4.02 – 3.98 (m, 1H), 3.89 – 3.85 (m, 1H), 3.79 – 3.76 (m, 1H), 3.74 – 3.69 (m, 2H), 2.75 (d, J = 14.4 Hz, 1H), 2.49 – 2.44 (m, 1H), 2.38 (s, 3H), 2.35 (s, 3H), 2.33 – 2.90 (m, 1H), 2.07 – 2.03 (m, 1H), 1.62 – 1.59 (m, 1H); ^{13}**C-NMR** (150 MHz, $CDCl_3$) δ 145.1, 142.8, 138.1, 134.1, 130.1, 129.8, 127.6, 126.6, 63.5, 63.4, 61.0, 55.4, 29.1, 27.7, 21.7, 21.5; **HRMS** (ESI) *m/z* calculated $C_{20}H_{24}NaO_4S_2$ $[M+Na]^+$ 415.1008, found 415.1017.

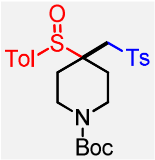

tert-butyl 4-(*p*-tolylsulfinyl)-4-(tosylmethyl)piperidine-1-carboxylate (62) White Solid; ^{1}**H-NMR** (500 MHz, $CDCl_3$) δ 7.74 (d, J = 8.0 Hz, 2H), 7.42 (d, J = 7.5 Hz, 2H), 7.35 (d, J = 8.0 Hz, 2H), 7.32 (d, J = 8.0 Hz, 2H), 3.74 – 3.67 (m, 4H), 3.61 – 3.47 (m, 1H), 2.74 (d, J = 14.5 Hz, 1H), 2.45 – 2.42 (m, 8H), 2.22 – 2.10 (m, 1H), 1.70 – 1.58 (m, 1H), 1.47 (s, 9H); ^{13}**C-NMR** (125 MHz, $CDCl_3$) δ 154.7, 145.1, 142.8, 138.1, 134.0, 130.1, 129.9, 127.5, 126.4, 80.1, 61.8, 55.5, 40.0, 39.1, 28.6, 28.4, 27.2, 21.7, 21.5; **HRMS** (ESI) *m/z* calculated $C_{25}H_{33}NNaO_5S_2$ $[M+Na]^+$ 514.1692, found 514.1699.

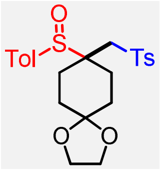

8-(*p*-tolylsulfinyl)-8-(tosylmethyl)-1,4-dioxaspiro[4.5]decane (63) White Solid; ^{1}**H-NMR** (500 MHz, $CDCl_3$) δ 7.73 (d, J = 8.5 Hz, 2H), 7.39 (d, J = 8.0 Hz, 2H), 7.34 – 7.30 (m, 4H), 3.99 – 3.95 (m, 4H), 3.69 (d, J = 14.0 Hz, 1H), 2.73 – 2.66 (m, 1H), 2.64 (d, J = 14.5 Hz, 1H), 2.61 – 2.55 (m, 1H), 2.44 (s, 3H), 2.42 (s, 3H), 2.26 – 2.22 (m, 1H), 2.18 – 2.12 (m, 1H), 2.02 – 1.96 (m, 1H), 1.79 – 1.72 (m, 2H), 1.64 – 1.59 (m, 1H); ^{13}**C-NMR** (125 MHz, $CDCl_3$) δ 144.8, 142.5, 138.4, 134.5, 130.0, 129.8, 127.6, 126.2, 107.3, 64.6, 62.6, 55.6, 31.0, 30.6, 27.4, 26.2, 21.7, 21.5; **HRMS** (ESI) *m/z* calculated $C_{23}H_{28}NaO_5S_2$ $[M+Na]^+$ 471.1270, found 471.1259.

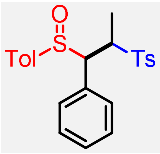

1-methyl-4-(((1*R*,2*R*)-1-phenyl-1-(*p*-tolylsulfinyl)propan-2-yl)sulfonyl)benzene (64) White Solid; ^{1}**H-NMR** (500 MHz, $CDCl_3$) δ 7.28 (d, J = 8.5 Hz, 2H), 7.05 – 6.99 (m, 5H), 6.84 – 6.79 (m, 4H), 6.53 (d, J = 7.0 Hz, 2H), 4.09 – 4.03 (m, 1H), 3.74 (d, J = 10.5 Hz, 1H), 2.31 (s, 3H), 2.29 (s, 3H), 2.08 (d, J = 7.0 Hz, 3H); ^{13}**C-NMR** (125 MHz, $CDCl_3$) δ 144.0, 141.4, 136.9, 135.8, 129.8, 129.34, 129.32, 129.2, 128.2, 127.8, 127.1, 124.3, 71.0, 61.2, 21.5, 21.4, 13.0; **HRMS** (ESI) *m/z* calculated $C_{23}H_{24}NaO_3S_2$ $[M+Na]^+$ 435.1059, found 435.1045.

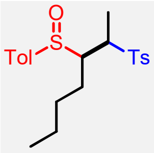

mixture

1-methyl-4-(((2*R*,3*R*)-3-(p-tolylsulfinyl)heptan-2-yl)sulfonyl)benzene (65) White Solid; mixture. ^{1}**H-NMR** (500 MHz, $CDCl_3$) δ 7.75 – 7.71 (m, 0.74H), 7.59 – 7.55 (m, 0.26H), 7.46 – 7.37 (m, 2H), 7.37 – 7.32 (m, 2H), 7.31 – 7.26 (m, 2H), 7.25 – 7.21 (m, 1H), 3.83 – 3.81 (m, 0.12H), 3.74 – 3.68 (m, 0.27H), 3.51 – 3.46 (m, 0.27H), 3.41 – 3.33 (m, 0.59H), 3.27 – 3.23 (m, 0.21H), 3.07 – 3.02 (m, 0.46H), 2.50 – 2.39 (m, 6H), 2.06 – 2.04 (m, 0.58H), 1.99 – 1.84 (m, 0.8H), 1.83 – 1.22 (m, 7.14H), 1.17 (d, J = 7.1 Hz, 0.42H), 1.13 – 1.03 (m, 0.29H), 0.94 – 0.91 (m, 2.16H), 0.90 – 0.82 (m, 1.49H), 0.80 – 0.72 (m, 0.2H). ^{13}**C-NMR** (125 MHz, $CDCl_3$) δ 145.0, 144.9, 144.7, 142.6, 142.2, 141.6, 138.3, 137.1, 134.4, 134.3, 130.1, 130.0, 129.93, 129.92, 129.7, 128.9, 128.7, 128.6, 128.5, 125.5, 125.0, 124.8, 124.6, 64.1, 61.3, 61.1, 60.6, 58.8, 58.5, 56.8, 56.4, 30.4, 30.2, 29.9, 29.8, 24.4, 23.9, 23.8, 23.2, 22.8, 22.7, 22.6, 22.4, 21.70, 21.65, 21.53, 21.49, 21.46, 13.9, 13.8, 13.7, 10.1, 9.2, 8.9,

6.8; **HRMS** (ESI) *m/z* calculated $C_{21}H_{29}O_3S_2$ $[M+H]^+$ 393.1553, found 393.1571.

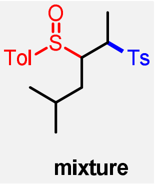

1-methyl-4-(((2*R*)-5-methyl-2-tosylhexan-3-yl)sulfinyl)benzene (66) White Solid; mixture. ^{1}**H-NMR** (600 MHz, $CDCl_3$) δ 7.75 – 7.52 (m, 1H), 7.37 – 7.23 (m, 3H), 7.23 – 7.15 (m, 3H), 7.13 – 7.09 (m, 1H), 3.73 – 3.67 (m, 0.25H), 3.55 – 3.52 (m, 0.25H), 3.34 – 3.28 (m, 0.6H), 3.06 – 3.02 (m, 0.5H), 2.40 – 2.32 (m, 6H), 1.98 – 1.88 (m, 1.3H), 1.82 – 1.63 (m, 1.3H), 1.56 – 1.40 (m, 0.7H), 1.38 – 1.36 (m, 1.6H), 1.21 – 1.18 (m, 0.4H), 1.06 – 1.05 (m, 0.3H), 0.94 – 0.88 (m, 3.8H), 0.85 – 0.68 (m, 3H). ^{13}**C-NMR** (150 MHz, $CDCl_3$) δ 145.0, 144.9, 144.6, 142.6, 142.2, 141.5, 139.7, 139.1, 138.3, 136.7, 135.4, 134.3, 130.01, 129.96, 129.9, 129.8, 129.72, 129.68, 128.9, 128.8, 128.6, 128.5, 128.0, 127.8, 125.6, 124.9, 124.8, 124.4, 124.1, 112.6, 68.0, 62.8, 59.3, 59.1, 58.5, 58.4, 58.2, 58.0, 56.5, 56.1, 55.6, 40.3, 37.3, 35.7, 33.4, 33.12, 33.06, 32.5, 29.9, 28.2, 27.9, 25.9, 25.6, 25.5, 25.4, 25.0, 24.3, 23.4, 23.2, 22.5, 22.4, 22.1, 21.71, 21.68, 21.6, 21.51, 21.48, 21.4, 21.2, 20.6, 12.5, 11. 6, 10.6, 9.8, 9.0, 8.1, 6.1; **HRMS** (ESI) *m/z* calculated $C_{21}H_{28}NaO_3S_2$ $[M+Na]^+$ 415.1372, found 415.1396.

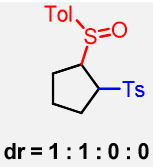

1-methyl-4-(((1*S*,2*R*)-2-(p-tolylsulfinyl)cyclopentyl)sulfonyl)benzene (67) White Solid; dr = 1 : 1 : 0 : 0. ^{1}**H-NMR** (500 MHz, $CDCl_3$) δ 7.81 (d, *J* = 8.0 Hz, 2H), 7.53 (d, *J* = 8.0 Hz, 2H), 7.39 (d, *J* = 8.0 Hz, 2H), 7.37 – 7.34 (m, 4H), 7.31 – 7.23 (m, 6H), 3.93 – 3.89 (m, 1H), 3.88 – 3.82 (m, 1H), 3.62 – 3.55 (m, 1H), 3.50 – 3.46 (m, 1H), 2.49 – 2.39 (m, 12H), 2.31 – 2.08 (m, 5H), 2.04 – 1.71 (m, 6H), 1.55 – 1.50 (m, 1H). ^{13}**C-NMR** (125 MHz, $CDCl_3$) δ 145.2, 144.6, 141.8, 141.5, 138.9, 138.6, 135.1, 135.0, 130.1, 129.91, 129.90, 129.8, 128.6, 128.4, 124.4, 123.9, 65.3, 64.2, 63.8, 62.7, 29.4, 28.6, 28.2, 25.4, 25.2, 22.9, 21.7, 21.6, 21.5, 21.4; **HRMS** (ESI) *m/z* calculated $C_{19}H_{22}NaO_3S_2$ $[M+Na]^+$ 385.0903, found 385.0894.

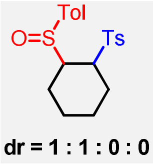

1-methyl-4-(((1*S*,2*R*)-2-(*p*-tolylsulfinyl)cyclohexyl)sulfonyl)benzene (68) White Solid; dr = 1 : 1 : 0 : 0. ^{1}**H-NMR** (500 MHz, $CDCl_3$) δ 7.73 (d, *J* = 8.0 Hz, 2H), 7.64 (d, *J* = 8.0 Hz, 2H), 7.50 (d, *J* = 8.0 Hz, 2H), 7.37 – 7.34 (m, 4H), 7.30 – 7.26 (m, 4H), 7.23 (d, *J* = 8.0 Hz, 2H), 3.59 – 3.53 (m, 1H), 3.25 – 3.19 (m, 2H), 3.00 –2.96 (m, 1H), 2.47 – 2.41 (m, 12H), 2.40 – 2.27 (m, 2H), 2.08 – 1.90 (m, 6H), 1.86 – 1.68 (m, 4H), 1.61 – 1.46 (m, 2H), 1.45 – 1.38 (m, 2H). ^{13}**C-NMR** (125 MHz, $CDCl_3$) δ 144.92, 144.87, 142.3, 141.7, 138.5, 138.3, 135.2, 134.3, 129.94, 129.87, 129.86, 128.8, 128.6, 125.5, 124.8, 60.7, 60.2, 59.7, 58.3, 24.9, 23.0, 22.7, 22.4, 22.1, 21.9, 21.7, 21.52, 21.47, 21.1, 19.5; **HRMS** (ESI) *m/z* calculated $C_{20}H_{24}NaO_3S_2$ $[M+Na]^+$ 399.1059, found 399.1064.

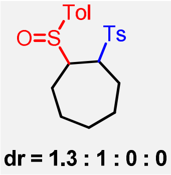

(1*R*,2*S*)-1-(*p*-tolylsulfinyl)-2-tosylcycloheptane (69) White Solid; dr = 1.3 : 1 : 0 : 0. (major isomer): ^{1}**H-NMR** (500 MHz, $CDCl_3$) δ 7.67 (d, *J* = 8.0 Hz, 2H), 7.41 (d, *J* = 8.0 Hz, 2H), 7.33 (d, *J* = 8.0 Hz, 2H), 7.29 (d, *J* = 8.0 Hz, 2H), 3.94 – 3.87 (m, 1H), 3.70 –3.67 (m, 1H), 2.46 (s, 3H), 2.42 (s, 3H), 2.16 – 2.07 (m, 1H), 2.02 – 1.94 (m, 2H), 1.94 – 1.80 (m, 3H), 1.78 – 1.70 (m, 2H), 1.60 – 1.53 (m, 1H), 1.49 – 1.40 (m, 1H). ^{13}**C-NMR** (125 MHz, $CDCl_3$) δ 144.8, 142.1, 139.4, 134.8, 129.9, 129.8, 129.0, 125.0, 62.7, 60.4, 30.8, 27.1, 26.5, 25.2, 24.3, 21.7, 21.5; (minor isomer): ^{1}**H-NMR** (500 MHz, $CDCl_3$) δ 7.72 (d, *J* = 8.0 Hz, 2H), 7.51 (d, *J* = 8.0 Hz, 2H), 7.35 (d, *J* = 8.0 Hz, 2H), 7.31 (d, *J* = 8.0 Hz, 2H), 3.76 – 3.71 (m, 1H), 3.43 – 3.39 (m, 1H), 2.47 (s, 3H), 2.43 (s, 3H), 2.07 – 2.06 (m, 1H), 2.01 – 1.86 (m, 4H), 1.83 – 1.74 (m, 1H), 1.57 – 1.47 (m, 4H). ^{13}**C-NMR** (125 MHz, $CDCl_3$) δ 145.1, 141.7, 138.4, 135.0, 130.0, 129.9, 128.8, 125.0, 64.3, 62.2, 30.7, 27.3, 26.0, 24.8, 23.3, 21.7, 21.5; **HRMS** (ESI) *m/z* calculated $C_{21}H_{26}NaO_3S_2$ $[M+Na]^+$ 413.1216, found 413.1211.

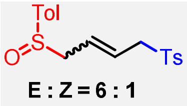

1-methyl-4-((4-(*p*-tolylsulfinyl)but-2-en-1-yl)sulfonyl)benzene (70) White Solid; E : Z = 6 : 1. (major isomer): ^{1}**H-NMR** (600 MHz, $CDCl_3$) δ 7.73 (d, *J* = 7.8 Hz, 2H), 7.41 (d, *J* = 7.8 Hz, 2H), 7.35 (d, *J* = 8.4 Hz, 2H), 7.30 (d, *J* = 7.8 Hz, 2H), 5.62 – 5.55 (m, 1H), 5.47 – 5.19 (m, 1H), 3.82 – 3.68 (m, 2H), 3.51 (dd, *J* = 12.6, 7.8 Hz, 1H), 3.42 (dd, *J* = 12.6, 7.8 Hz, 1H), 2.45 (s, 3H), 2.42 (s, 3H).^{13}**C-NMR** (150 MHz, $CDCl_3$) δ 145.0, 141.8, 139.4, 135.5, 129.91, 129.85, 128.5, 127.5, 125.4, 124.2, 59.9, 59.7, 21.7, 21.5; **HRMS** (ESI) *m/z* calculated $C_{18}H_{20}NaO_3S_2$ $[M+Na]^+$ 371.0746, found 371.0742.

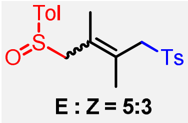

1-((2,3-dimethyl-4-(*p*-tolylsulfinyl)but-2-en-1-yl)sulfonyl)-4-methylbenzene (71) White Solid; E : Z = 5 : 3. (major isomer): **^{1}H-NMR** (500 MHz, $CDCl_3$) δ 7.71 (d, *J* = 8.0 Hz, 2H), 7.45 (d, *J* = 8.0 Hz, 2H), 7.34 – 7.30 (m, 4H), 4.07 (d, *J* = 14.0 Hz, 1H), 3.60 (dd, *J* = 18.0, 14.0 Hz, 2H), 3.21 (d, *J* = 13.5 Hz, 1H), 2.43 (s, 3H), 2.42 (s, 3H), 1.81 (s, 3H), 1.77 (s, 3H). **^{13}C-NMR** (125 MHz, $CDCl_3$) δ 144.8, 141.8, 140.6, 136.6, 129.93, 129.89, 129.1, 128.1, 125.8, 123.9, 63.7, 61.3, 21.7, 21.5, 20.8, 20.4. (minor isomer): **^{1}H-NMR** (500 MHz, $CDCl_3$) δ 7.77 (d, *J* = 8.0 Hz, 2H), 7.54 (d, *J* = 8.0 Hz, 2H), 7.37 – 7.31 (m, 4H), 3.90 – 3.81 (m, 2H), 3.71 (d, *J* = 12.5 Hz, 1H), 3.40 (d, *J* = 12.5 Hz, 1H), 2.45 (s, 3H), 2.42 (s, 3H), 1.71 (q, *J* = 1.5 Hz, 3H), 1.49 (q, *J* = 1.5 Hz, 3H). **^{13}C-NMR** (150 MHz, $CDCl_3$) δ 144.9, 141.9, 140.7, 136.4, 129.98, 129.93, 129.4, 128.3, 125.4, 124.1, 64.5, 61.9, 21.7, 21.5, 20.2, 20.1; **HRMS** (ESI) *m/z* calculated $C_{20}H_{24}NaO_3S_2$ $[M+Na]^+$ 399.1059, found 399.1057.

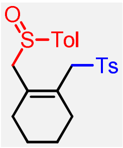

1-methyl-4-(((2-((*p*-tolylsulfinyl)methyl)cyclohex-1-en-1-yl)methyl)sulfonyl)benz ene (72) White Solid; **^{1}H-NMR** (600 MHz, $CDCl_3$) δ 7.72 (d, *J* = 7.8 Hz, 2H), 7.48 (d, *J* = 8.4 Hz, 2H), 7.34 – 7.30 (m, 4H), 4.05 (d, *J* = 14.4 Hz, 1H), 3.67 (d, *J* = 13.2 Hz, 1H), 3.52 (d, *J* = 14.4 Hz, 1H), 3.18 (d, *J* = 13.8 Hz, 1H), 2.43 (s, 3H), 2.42 (s, 3H), 2.11 – 2.03 (m, 4H), 1.59 – 1.53 (m, 4H); **^{13}C-NMR** (125 MHz, $CDCl_3$) δ 144.9, 142.1, 139.9, 136.5, 130.8, 130.0, 129.9, 128.2, 124.2, 62.5, 60.4, 31.4, 31.2, 22.4, 22.3, 21.7, 21.5; **HRMS** (ESI) *m/z* calculated $C_{22}H_{26}NaO_3S_2$ $[M+Na]^+$ 425.1216, found 425.1210.

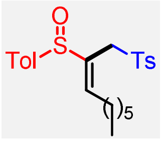

(*E*)-1-methyl-4-((2-(*p*-tolylsulfinyl)non-2-en-1-yl)sulfonyl)benzene (73) White Solid; **^{1}H-NMR** (500 MHz, $CDCl_3$) δ 7.79 (d, *J* = 8.0 Hz, 2H), 7.46 (d, *J* = 8.5 Hz, 2H), 7.37 (d, *J* = 8.0 Hz, 2H), 7.30 (d, *J* = 8.0 Hz, 2H), 7.13 (t, *J* = 7.5 Hz, 1H), 3.82 (d, *J* = 14.0 Hz, 1H), 3.55 (d, *J* = 14.0 Hz, 1H), 2.45 (s, 3H), 2.41 (s, 3H), 2.09 – 2.02 (m, 1H), 1.81 – 1.74 (m, 1H), 1.40 – 1.19 (m, 8H), 0.86 (t, *J* = 7.5 Hz, 3H); **^{13}C-NMR** (125 MHz, $CDCl_3$) δ 149.2, 144.9, 142.1, 140.3, 136.1, 132.8, 130.1, 129.9, 128.4, 124.3, 56.3, 31.5, 28.9, 28.8, 28.1, 22.5, 21.7, 21.5, 14.0; **HRMS** (ESI) *m/z* calculated $C_{23}H_{30}NaO_3S_2$ $[M+Na]^+$ 441.1529, found 441.1518.

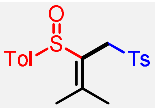

1-methyl-4-((3-methyl-1-tosylbut-2-en-2-yl)sulfinyl)benzene (74) White Solid; **^{1}H-NMR** (600 MHz, $CDCl_3$) δ 7.86 (d, *J* = 7.8 Hz, 2H), 7.61 (d, *J* = 7.8 Hz, 2H), 7.35 (d, *J* = 7.8 Hz, 4H), 4.08 (d, *J* = 13.8 Hz, 1H), 3.98 (d, *J* = 13.8 Hz, 1H), 2.44 (s, 3H), 2.43 (s, 3H), 2.09 (s, 3H), 1.90 (s, 3H); **^{13}C-NMR** (150 MHz, $CDCl_3$) δ 156.7, 144.2, 141.7, 140.7, 139.0, 130.0, 129.8, 129.4, 127.5, 124.1, 59.3, 25.1, 23.2, 21.6, 21.5; **HRMS** (ESI) *m/z* calculated $C_{19}H_{22}NaO_3S_2$ $[M+Na]^+$ 385.0903, found 385.0895.

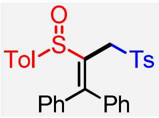

(2-(*p*-tolylsulfinyl)-3-tosylprop-1-ene-1,1-diyl)dibenzene (75) White Solid; **^{1}H-NMR** (600 MHz, $CDCl_3$) δ 7.57 (d, *J* = 8.4 Hz, 2H), 7.35 (d, *J* = 8.4 Hz, 2H), 7.27 – 7.26 (m, 2H), 7.23 – 7.16 (m, 4H), 7.07 (t, *J* = 7.2 Hz, 2H), 7.03 (d, *J* = 8.4 Hz, 2H), 6.91 (d, *J* = 7.2 Hz, 2H), 6.84 (d, *J* = 5.4 Hz, 2H), 4.23 (d, *J* = 12.6 Hz, 1H), 4.14 (d, *J* = 12.6 Hz, 1H), 2.40 (s, 3H), 2.33 (s, 3H); **^{13}C-NMR** (150 MHz, $CDCl_3$) δ 158.2, 143.6, 141.7, 140.7, 139.6, 138.4, 137.5, 134.1, 129.8, 129.3, 129.1, 128.7, 128.6, 128.3, 127.8, 127.6, 124.7, 59.8, 21.6, 21.5; **HRMS** (ESI) *m/z* calculated $C_{29}H_{26}NaO_3S_2$ $[M+Na]^+$ 509.1216, found 509.1236.

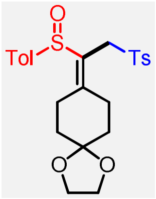

8-(1-(p-tolylsulfinyl)-2-tosylethylidene)-1,4-dioxaspiro[4.5]decane (76) White Solid; **^{1}H-NMR** (500 MHz, $CDCl_3$) δ 7.85 (d, *J* = 8.5 Hz, 2H), 7.62 (d, *J* = 8.0 Hz, 2H), 7.36 – 7.33 (m, 4H), 4.08 (s, 2H), 3.94 – 3.87 (m, 4H), 2.87 – 2.82 (m, 1H), 2.71 – 2.59 (m, 2H), 2.43 (s, 6H), 2.41 – 2.36 (m, 1H), 1.81 – 1.76 (m, 1H), 1.68 – 1.61 (m, 1H), 1.54 – 1.49 (m, 1H), 1.40 – 1.34 (m, 1H); **^{13}C-NMR** (125 MHz, $CDCl_3$) δ 160.6, 144.2, 141.8, 140.6, 139.6, 130.0, 129.9, 128.2, 127.2, 124.1, 107.2, 64.5, 64.4, 58.4, 35.4, 34.5, 31.2, 29.2, 21.6, 21.5; **HRMS** (ESI) *m/z* calculated $C_{24}H_{28}NaO_5S_2$ $[M+Na]^+$ 483.1270, found 483.1273.

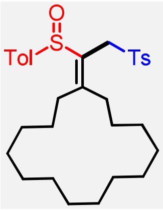

(1-(p-tolylsulfinyl)-2-tosylethylidene)cyclopentadecane (77) White Solid; **^{1}H-NMR** (600 MHz, $CDCl_3$) δ 7.87 – 7.84 (m, 2H), 7.65 – 7.61 (m, 2H), 7.35 – 7.33 (m, 4H), 4.03 – 3.95 (m, 2H), 2.43 – 2.40 (m, 8H), 2.32 – 2.29 (m, 1H), 2.00 –1.94 (m, 1H), 1.33 – 1.25 (m, 23H), 1.01 – 0.98 (m, 1H); **^{13}C-NMR** (150 MHz, $CDCl_3$) δ 164.7, 144.1, 141.6, 140.8, 139.7, 129.9, 129.8, 129.2, 127.4, 124.2, 59.3, 33.8, 33.3, 27.8, 27.7, 26.7, 26.6, 26.4, 26.3, 26.2, 26.0, 25.9, 25.8, 21.6, 21.5; **HRMS** (ESI) *m/z* calculated $C_{31}H_{44}NaO_3S_2$ $[M+Na]^+$ 551.2624, found 551.2629.

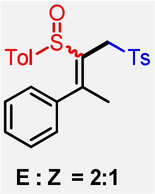

1-methyl-4-((3-phenyl-1-tosylbut-2-en-2-yl)sulfinyl)benzene (78) White Solid; E : Z = 2 : 1. **^{1}H-NMR** (600 MHz, $CDCl_3$) δ 8.03 (d, *J* = 8.4 Hz, major, 2H), 7.74 (d, *J* = 8.4 Hz, minor, 2H), 7.43 – 7.35 (m, major, 4H, minor, 2H), 7.32 – 7.28 (m, major, 2H, minor, 2H), 7.25 – 7.17 (m, major, 2H, minor, 3H), 7.11 – 7.07 (m, major, 2H, minor, 2H), 7.00 (d, *J* = 8.4 Hz, minor, 2H), 6.85 (d, *J* = 7.2 Hz, major, 1H), 4.22 (dd, *J* = 28.8, 7.2 Hz, minor, 2H), 3.93 (d, *J* = 13.2 Hz, major, 1H), 3.85 (dd, *J* = 13.2, 1.2 Hz, major, 1H), 2.47 (s, major, 3H), 2.45 (s, minor, 3H), 2.39 (s, major, 3H), 2.34 (s, minor, 3H), 2.32 (s, major, 3H), 2.08 (s, minor, 3H). **^{13}C-NMR** (150 MHz, $CDCl_3$) δ 157.9, 156.7, 144.5, 143.4, 141.9, 141.5, 141.01, 140.97, 139.6, 138.8, 137.9, 132.8, 131.8, 130.0, 129.9, 129.8, 129.0, 128.5, 128.4, 127.82, 127.79, 127.7, 127.2, 124.22, 124.17, 60.1, 59.1, 26.4, 24.1, 21.7, 21.53, 21.51, 21.4; **HRMS** (ESI) *m/z* calculated $C_{24}H_{24}NaO_3S_2$ $[M+Na]^+$ 447.1059, found 447.1053.

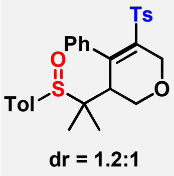

4-phenyl-3-(2-(p-tolylsulfinyl)propan-2-yl)-5-tosyl-3,6-dihydro-2*H*-pyran (79) White Solid; dr = 1.2 : 1. (major isomer): **^{1}H-NMR** (600 MHz, $CDCl_3$) δ 7.60 – 7.35 (m, 1H), 7.30 (d, *J* = 8.4 Hz, 2H), 7.26 – 7.15 (m, 5H), 7.12 (d, *J* = 8.4 Hz, 2H), 7.01 (d, *J* = 8.4 Hz, 2H), 6.70 m, 2H), 4.62 (dd, *J* = 17.4, 2.4 Hz, 1H), 3.59 (dd, *J* = 12.6, 3.0 Hz, 1H), 2.80 (t, *J* = 2.4 Hz, 1H), 2.36 (s, 3H), 2.34 (s, 3H), 1.14 (s, 3H), 0.55 (s, 3H). **^{13}C-NMR** (150 MHz, $CDCl_3$) δ 148.4, 143.8, 141.9, 141.3, 138.4, 137.8, 135.1, 133.1, 129.2, 129.1, 128.6, 127.2, 126.9, 66.0, 65.8, 63.1, 46.4, 21.5, 21.4, 19.6, 19.1; (minor isomer): **^{1}H-NMR** (600 MHz, $CDCl_3$) δ 7.75 – 7.60 (m, 1H), 7.40 – 7.30 (m, 1H), 7.29 – 7.27 (m, 1H), 7.21 – 7.18 (m, 4H), 7.17 (d, *J* = 8.4 Hz, 2H), 7.12 – 7.05 (m, 1H). 7.02 (d, *J* = 8.4 Hz, 2H), 6.66 – 6.50 (m, 1H), 4.86 (d, *J* = 17.2 Hz, 1H), 4.59 (dd, *J* = 17.2, 2.4 Hz, 1H), 4.34 (d, *J* = 12.0 Hz, 1H), 3.57 (dd, *J* = 12.0, 3.0 Hz, 1H), 3.05 (t, *J* = 2.4 Hz, 1H), 2.36 (s, 3H), 2.34 (s, 3H), 1.09 (s, 3H), 1.03 (s, 3H). **^{13}C-NMR** (150 MHz, $CDCl_3$) δ 148.8, 143.8, 140.4, 140.3, 137.9, 134.3, 129.1, 129.0, 127.3, 126.9, 126.6, 66.9, 66.1, 63.2, 44.1, 21.5, 21.4, 20.9, 17.3; **HRMS** (ESI) *m/z* calculated $C_{28}H_{30}NaO_4S_2$ $[M+Na]^+$ 517.1478, found 517.1485.

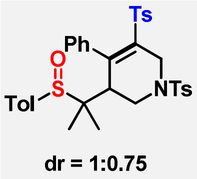

4-phenyl-3-(2-(*p*-tolylsulfinyl)propan-2-yl)-1,5-ditosyl-1,2,3,6-tetrahydropy ridine (80) White Solid; dr = 1 : 0.75. **^{1}H-NMR** (600 MHz, $CDCl_3$) δ 7.78 (d, *J* = 8.4 Hz, major, 2H), 7.72 (d, *J* = 8.4 Hz, minor, 2H), 7.58 (d, *J* = 7.2 Hz, minor, 1H), 7.44 – 7.38 (m, major, 4H), 7.35 – 7.30 (m, major, 1H), 7.30 – 7.26 (minor, 4H), 7.26 – 7.24 (m, minor, 1H), 7.23 – 7.16 (m, major, 4H, minor, 4H), 7.13 – 7.09 (m, major, 1H, minor, 1H), 7.07– 7.04 (m, major, 2H, minor, 1H), 7.00 – 6.97 (m, major, 3H, minor, 1H), 6.59 – 6.49 (m, minor, 1H), 6.48 – 6.29 (m, minor, 1H), 4.75 – 4.64 (m, major, 2H, minor, 1H), 4.10 (d, *J* = 12.3 Hz, minor, 1H), 3.70 –3.63 (m, major, 1H, minor, 1H), 3.36 (br., minor, 1H), 2.99 (br., major, 1H), 2.53 – 2.44 (m, major, 4H, minor, 4H), 2.36 – 3.32 (m, major, 6H, minor, 6H), 1.20 – 1.17 (m, major, 3H, minor, 3H), 1.15 (s, minor, 3H), 0.61 (s, major, 3H); **^{13}C-NMR** (150 MHz, $CDCl_3$) δ 149.2, 148.6, 144.5, 144.4, 144.0, 143.9, 142.0, 141.7, 139.7, 138.1, 138.0, 137.5, 136.6, 135.1, 135.0, 131.9, 131.6, 130.11, 130.08, 129.3, 129.14, 129.09, 129.06, 128.8, 128.05, 128.03, 127.4, 127.2, 127.0, 126.7, 63.71, 63.69, 47.6, 46.4, 46.3, 46.2, 45.2, 45.0, 21.7, 21.6, 21.4, 20.7, 19.4, 19.2, 16.1; **HRMS** (ESI) *m/z* calculated $C_{35}H_{37}NNaO_5S_3$ $[M+Na]^+$ 670.1726, found 670.1735.

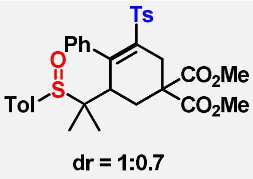

dimethyl 6-(2-(*p*-tolylsulfinyl)propan-2-yl)-2-tosyl-5,6-dihydro-[1,1'- biphenyl]-4,4(3*H*)-dicarboxylate (81) White Solid; dr = 1:0.7. **^{1}H-NMR** (600 MHz, $CDCl_3$) δ 7.70 – 7.60 (m, minor, 1H), 7.30 – 7.29 (m, major, 2H), 7.26 – 7.23 (m, major, 4H), 7.23 – 7.18 (m, major, 3H, minor, 4H), 7.14 – 7.11 (m, minor, 6H), 7.04 – 7.01 (m, major, 2H), 6.99 – 6.97 (m, major, 1H, minor, 2H),6.46 – 6.29 (m, major, 1H), 3.81 (s, major, 3H), 3.80 (s, minor, 3H), 3.66 (s, major, 3H), 3.60 (s, minor, 3H), 3.39 – 3.33 (m, major, 1H, minor, 1H), 3.05 (dd, *J* = 17.2, 3.0 Hz, minor, 1H), 2.87 (dd, *J* = 17.4, 3.6 Hz, major, 1H), 2.83 – 2.79 (m, minor, 1H), 2.65 – 2.61 (m, major, 1H), 2.50 (dd, *J* = 13.2, 10.2 Hz, major, 1H), 2.46 – 2.39 (m, minor, 2H), 2.36 – 2.35 (m, major, 3H, minor, 3H), 2.33 (s, minor, 3H), 2.31 (s, major, 3H), 1.98 (dd, *J* = 13.8, 10.8 Hz, major, 1H), 1.05 (s, major, 3H), 0.78 – 0.77 (m, major, 3H, minor, 3H), 0.64 (s, minor, 3H); **^{13}C-NMR** (150 MHz, $CDCl_3$) δ 171.2, 170.2, 169.9, 150.4, 150.3, 143.4, 143.3, 141.8, 141.4, 141.0, 140.6, 138.1, 137.9, 136.7, 136.3, 135.9, 135.4, 129.13, 129.11, 129.0, 128.9, 128.8, 128.6, 128.2, 127.8, 127.6, 127.6, 126.7, 126.4, 124.3, 64.4, 63.0, 54.8, 54.2, 53.3, 53.2, 52.9, 52.8, 50.6, 48.7, 46.0, 32.1, 31.5, 30.8, 21.5, 21.4, 18.2, 18.1, 17.3; **HRMS** (ESI) *m/z* calculated $C_{33}H_{36}NaO_7S_2$ $[M+Na]^+$ 631.1795, found 631.1788.

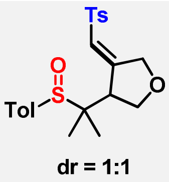

(*Z*)-3-(2-(*p*-tolylsulfinyl)propan-2-yl)-4-(tosylmethylene)tetrahydrofuran (82) White Solid; dr = 1 : 1. **^{1}H-NMR** (600 MHz, $CDCl_3$) δ 7.80 – 7.76 (m, 4H), 7.43 (d, *J* = 8.4 Hz, 2H), 7.39 – 7.33 (m, 6H), 7.32 – 7.29 (m, 4H), 6.71 (s, 1H), 6.33 (s, 1H), 4.96 – 4.89 (m, 2H), 4.88 – 4.77 (m, 2H), 4.57 (dd, *J* = 10.2, 1.8 Hz, 1H), 4.10 (dd, *J* = 9.8, 1.8 Hz, 1H), 3.84 – 3.79 (m, 2H), 3.14 (t, *J* = 6.6 Hz, 2H), 2.44 – 2.41 (m, 12H), 1.06 (s, 3H), 1.01 (s, 3H), 0.97 (s, 3H) , 0.99 (s, 3H); **^{13}C-NMR** (150 MHz, $CDCl_3$) δ 157.3, 155.8, 144.9, 144.8, 142.4, 142.3, 138.1, 138.0, 135.1, 134.9, 130.1, 130.0, 129.49, 129.45, 127.3, 127.2, 126.52, 126.50, 125.5, 125.2, 70.3, 70.2, 68.9, 68.6, 61.3, 61.2, 50.0, 49.5, 21.7, 21.5, 17.64, 17.55, 17.48, 17.2; **HRMS** (ESI) *m/z* calculated $C_{22}H_{26}NaO_4S_2$ $[M+Na]^+$ 441.1165, found 441.1169.

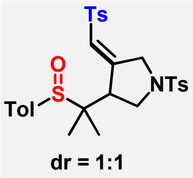

(*Z*)-3-(2-(*p*-tolylsulfinyl)propan-2-yl)-1-tosyl-4-(tosylmethylene)pyrrolidine (83) White Solid; dr = 1 : 1. **^{1}H-NMR** (600 MHz, $CDCl_3$) δ 7.75 – 7.72 (m, 6H), 7.69 (d, *J* = 8.4 Hz, 2H), 7.41 (d, *J* = 7.8 Hz, 2H), 7.39 – 7.36 (m, 4H), 7.34 – 7.27 (m, 10H), 6.66 – 6.64 (m, 1H), 6.32 – 6.20 (m, 1H), 4.49 – 4.45 (m, 2H), 4.37 – 4.26 (m, 2H), 4.13 (dd, *J* = 10.4, 1.2 Hz, 1H), 3.60 (dd, *J* = 10.4, 1.2 Hz, 1H), 3.15 (d, *J* = 6.0 Hz, 1H), 3.11 – 3.04 (m, 3H), 2.46 – 2.40 (m, 18H), 1.03 (s, 3H), 0.99 (s, 3H), 0.95 (s, 3H), 0.88 (s, 3H); **^{13}C-NMR** (150 MHz, $CDCl_3$) δ 152.1, 150.8, 145.1, 145.0, 144.4, 144.3, 142.49, 142.46, 137.8, 137.7, 134.8, 134.7, 131.6, 131.5, 130.2, 130.1, 130.02, 130.00, 129.5, 127.94, 127.89, 127.5, 127.3, 127.2, 126.5, 61.7, 61.6, 50.8, 50.6, 48.7, 48.4, 48.1, 21.7, 21.6, 21.5, 17.53, 17.51, 17.1; **HRMS** (ESI) *m/z* calculated $C_{29}H_{33}NNaO_5S_3$ $[M+Na]^+$ 594.1413, found 594.1420.

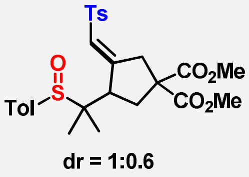

(*E*)-dimethyl 3-(2-(*p*-tolylsulfinyl)propan-2-yl)-4-(tosylmethyle ne)cyclopentane-1,1-dicarboxylate (84) White Solid; dr = 1 : 0.6. **^{1}H-NMR** (600 MHz, $CDCl_3$) δ 7.83 – 7.81 (m, major, 2H, minor, 2H), 7.44 (d, *J* = 8.4 Hz, major, 2H), 7.40 (d, *J* = 8.4 Hz, minor, 2H), 7.36 – 7.34 (m, major, 2H, minor, 2H), 7.32 – 7.30 (m, major, 2H minor, 2H), 6.79– 6.76 (m, major, 1H), 6.53– 6.51 (m, minor, 1H), 4.15 – 4.10 (m, major, 1H, minor, 1H), 3.78 – 3.77 (m, major, 3H, minor, 3H), 3.62 – 3.60 (m, major, 3H, minor, 3H), 3.30–3.26 (m, major, 1H), 3.13–3.09 (m, minor, 1H), 3.00 – 2.93 (m, major, 1H, minor, 1H), 2.78 – 2.70 (m, minor, 1H), 2.66 – 2.62 (m, major, 1H), 2.44 (s, major, 3H, minor, 3H), 2.43 – 2.42 (m, minor, 1H), 2.41 (s, major, 3H, minor, 3H), 2.14 (dd, *J* = 13.8, 10.2 Hz, major, 1H), 1.13 (s, minor, 3H), 1.02 (s, major, 3H), 0.96 (s, major, 3H), 0.92 (s, minor, 3H); **^{13}C-NMR** (150 MHz, $CDCl_3$) δ 170.9, 170.7, 170.63, 170.58, 157.4, 156.3, 144.5, 144.4, 142.3, 142.2, 138.57, 138.55, 135.1, 135.0, 129.94, 129.85, 129.5, 128.2, 128.0, 127.4, 127.3, 126.52, 126.49, 62.5, 62.3, 58.50, 58.47, 53.19, 53.15, 53.1, 53.0, 49.4, 47.2, 39.7, 39.6, 34.5, 34.0, 21.6, 21.5, 17.5, 17.3, 16.9, 16.8; **HRMS** (ESI) *m/z* calculated $C_{27}H_{32}NaO_7S_2$ $[M+Na]^+$ 555.1482, found 555.1487.

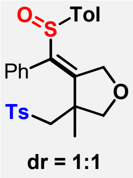

(*Z*)-3-methyl-4-(phenyl(*p*-tolylsulfinyl)methylene)-3-(tosylmethyl)tetrahydrofuran (85) White Solid; dr = 1 : 1. **^{1}H-NMR** (500 MHz, $CDCl_3$) δ 7.54 (d, *J* = 8.5 Hz, 2H), 7.47 (d, *J* = 8.5 Hz, 2H), 7.30 – 7.24 (m, 8H), 7.22 – 7.13 (m, 6H), 7.14 – 7.11 (m, 4H), 7.05 – 7.00 (m, 4H), 5.25 (d, *J* = 15.0 Hz, 1H), 5.09 (d, *J* = 15.0 Hz, 1H), 4.95 (dd, *J* = 15.0, 6.5 Hz, 2H), 4.29 (d, *J* = 9.0 Hz, 1H), 4.23 (d, *J* = 9.0 Hz, 1H), 3.91 (d, *J* = 9.5 Hz, 1H), 3.78 (d, *J* = 9.5 Hz, 1H), 3.07 – 2.98 (m, 3H), 2.87 (d, *J* = 14.5 Hz, 1H), 2.40 (s, 6H), 2.36 (s, 3H), 2.34 (s, 3H), 1.22 (s, 3H), 1.08 (s, 3H); **^{13}C-NMR** (150 MHz, $CDCl_3$) δ 153.1, 152.9, 144.74, 144.66, 141.5, 141.3, 138.8, 138.7, 138.1, 138.0, 137.9, 131.3, 129.9, 129.84, 129.83, 129.6, 129.53, 129.50, 129.4, 128.9, 128.8, 128.0, 127.6, 127.5, 127.3, 127.2, 124.4, 124.1, 78.0, 77.8, 70.9, 70.1, 61.7, 61.1, 47.1, 47.1, 23.7, 23.1, 21.6, 21.5, 21.4; **HRMS** (ESI) *m/z* calculated $C_{27}H_{28}NaO_4S_2$ $[M+Na]^+$ 503.1321, found 503.1324.

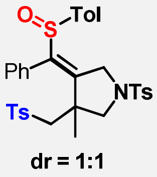

(*Z*)-3-methyl-4-(phenyl(*p*-tolylsulfinyl)methylene)-1-tosyl-3-(tosylmethyl)pyrrolidine (86) White Solid; dr = 1 : 1. **^{1}H-NMR** (600 MHz, $CDCl_3$) δ 7.80 – 7.74 (m, 4H), 7.51 (d, *J* = 8.4 Hz, 2H), 7.42 – 7.40 (m, 4H), 7.38 (d, *J* = 8.4 Hz, 2H), 7.34 – 7.16 (m, 9H), 7.12 – 7.09 (m, 6H), 7.03 – 6.98 (m, 6H), 6.00 –6.10 (m, 1H), 4.68 – 4.60 (m, 2H), 4.41 (d, *J* = 15.0 Hz, 1H), 4.32 (d, *J* = 15.0 Hz, 1H), 3.86 (d, *J* = 10.2 Hz, 1H), 3.60 (d, *J* = 10.2 Hz, 1H), 3.32 (d, *J* = 10.2 Hz, 1H), 3.16 – 3.06 (m, 2H), 2.97 (d, *J* = 14.4 Hz, 1H), 2.82 (d, *J* = 14.4 Hz, 1H), 2.73 (d, *J* = 14.4 Hz, 1H), 2.48 (s, 6H), 2.39 (s, 6H), 2.35 – 2.29 (m, 6H), 1.30 (s, 3H), 1.04 (s, 3H); **^{13}C-NMR** (150 MHz, $CDCl_3$) δ 149.1, 148.8, 144.83, 144.75, 144.5, 144.4, 141.7, 141.5, 141.3, 140.8, 138.0, 137.8, 137.52, 137.46, 131.6, 131.3, 130.11, 130.07, 130.0, 129.9, 129.8, 129.6, 129.5, 129.0, 128.9, 128.12, 128.10, 127.7, 127.5, 127.4, 127.3, 127.1, 124.4, 124.1, 61.52, 61.47, 58.0, 57.5, 51.4, 50.6, 46.8, 46.5, 24.4, 24.2, 21.67, 21.66, 21.6, 21.44, 21.41; **HRMS** (ESI) *m/z* calculated $C_{34}H_{35}NNaO_5S_3$ $[M+Na]^+$ 656.1570, found 656.1589.

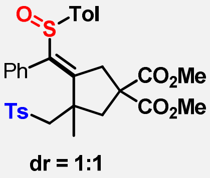

(*E*)-dimethyl 3-methyl-4-(phenyl(*p*-tolylsulfinyl)methylene)-3-(tosylmethyl)cyclopentane-1,1-dicarboxylate (87) White Solid; dr = 1 : 1. **^{1}H-NMR** (500 MHz, $CDCl_3$) δ 7.49 (d, *J* = 8.0 Hz, 2H), 7.43 (d, *J* = 8.0 Hz, 2H), 7.34 – 7.00 (m, 1H), 7.28 – 7.20 (m, 7H), 7.16 – 7.12 (m, 9H), 7.10 – 7.05 (m, 1H), 6.97 – 6.85 (m, 2H), 6.05 – 5.91 (m, 2H), 4.05 (d, *J* = 17.5 Hz, 1H), 3.92 (d, *J* = 17.5 Hz, 1H), 3.83 – 3.81 (m, 12H), 3.75 – 3.65 (m, 2H), 3.29 (d, *J* = 14.5 Hz, 1H), 3.21 (d, *J* = 14.5 Hz, 1H), 3.01 – 3.00 (m, 3H), 2.92 (d, *J* = 14.5 Hz, 1H), 2.60 (d, *J* = 14.5 Hz, 1H), 2.53 (d, *J* = 14.5 Hz, 1H), 2.40 – 2.34 (m, 12H), 1.15 (s, 3H), 1.01 (s, 3H); **^{13}C-NMR** (150 MHz, $CDCl_3$) δ 171.8, 171.32, 171.27, 153.4, 153.3, 144.5, 144.4, 141.90, 141.86, 141.0, 140.9, 138.8, 138.7, 138.5, 138.4, 133.4, 132.4, 131.1, 130.1, 129.8, 129.7, 129.41, 129.36, 128.63, 128.58, 128.2, 128.0, 127.2, 127.1, 124.4, 124.1, 63.8, 63.6, 58.2, 57.8, 53.33, 53.30, 53.22, 53.15, 46.8, 46.7, 45.6, 44.6, 40.0, 39.3, 27.6, 27.1, 21.6, 21.40, 21.37; **HRMS** (ESI) *m/z* calculated $C_{32}H_{34}NaO_7S_2$ $[M+Na]^+$ 617.1638, found 617.1642.

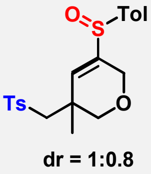

3-methyl-5-(*p*-tolylsulfinyl)-3-(tosylmethyl)-3,6-dihydro-2*H*-pyran (88) White Solid; dr = 1 : 0.8. **^{1}H-NMR** (600 MHz, $CDCl_3$) δ 7.76 (d, *J* = 7.8 Hz, major, 2H), 7.69 (d, *J* = 7.8 Hz, minor, 2H), 7.51 (d, *J* = 7.8 Hz, minor, 2H), 7.45 (d, *J* = 7.8 Hz, major, 2H), 7.36 – 7.27 (m, major, 4H, minor, 4H), 6.07 (s, minor, 1H), 6.01 (s, major, 1H), 4.95 (d, *J* = 15.6 Hz, minor, 1H), 4.84 (d, *J* = 15.6 Hz, major, 1H), 4.75 – 4.71 (m, major, 1H, minor, 1H), 4.28 (d, *J* = 9.6 Hz, major, 1H), 4.22 (d, *J* = 9.6 Hz, minor, 1H), 3.83 (d, *J* = 9.6 Hz, major, 1H), 3.70 (d, *J* = 9.6 Hz, minor, 1H), 3.25 – 3.14 (m, major, 2H, minor, 2H), 2.49 – 2.40 (m, major, 6H, minor, 6H), 1.46 – 1.45 (m, major, 3H, minor, 3H); **^{13}C-NMR** (125 MHz, $CDCl_3$) δ 159.3, 158.3, 145.12, 145.07, 141.9, 141.8, 140.3, 138.0, 137.9, 130.3, 130.2, 130.09, 130.06, 128.9, 127.70, 127.66, 126.9, 126.7, 124.3, 124.1, 77.0, 69.1, 68.7, 62.4, 62.2, 47.0, 46.9, 22.5, 22.4, 21.7, 21.5; **HRMS** (ESI) *m/z* calculated $C_{21}H_{24}NaO_4S_2$ $[M+Na]^+$ 427.1008, found 427.1026.

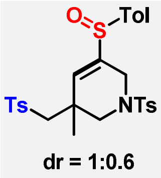

3-methyl-5-(*p*-tolylsulfinyl)-1-tosyl-3-(tosylmethyl)-1,2,3,6-tetrahydropyridine (89) White Solid; dr = 1 : 0.6. **^{1}H-NMR** (600 MHz, $CDCl_3$) δ 7.74 – 7.72 (m, major, 2H, minor, 2H), 7.70 (d, J = 8.4 Hz, major, 2H), 7.65 (d, J = 8.4 Hz, minor, 2H), 7.48 (d, J = 8.4 Hz, minor, 2H), 7.41 (d, J = 8.4 Hz, major, 2H), 7.40 – 7.37 (m, major, 2H, minor, 2H), 7.35 – 7.33 (m, major, 2H), 7.32 – 7.29 (m, major, 2H, minor, 4H), 6.13 (t, J = 2.4 Hz, minor, 1H), 5.99 (t, J = 2.4 Hz, major, 1H), 4.36 – 4.25 (m, major, 2H), 4.19 (dd, J = 16.1, 2.4 Hz, major, 1H), 3.82 (d, J = 10.2 Hz, minor, 1H), 3.51 (d, J = 10.2 Hz, major, 1H), 3.40 (d, J = 10.2 Hz, major, 1H), 3.22 – 3.09 (m, major, 1H, minor, 4H), 2.88 (d, J = 10.2 Hz, minor, 1H), 2.46 – 2.43 (m, major, 6H, minor, 6H), 2.39 (s, major, 3H), 2.38 (s, minor, 3H), 1.43 (s, major, 3H), 1.42 (s, minor, 3H); **^{13}C-NMR** (150 MHz, $CDCl_3$) δ 154.8, 153.5, 145.2, 145.1, 144.5, 144.4, 142.1, 141.9, 139.8, 139.6, 137.8, 137.7, 131.9, 131.8, 130.3, 130.2, 130.10, 130.08, 130.05, 129.7, 128.9, 128.0, 127.9, 127.68, 127.65, 124.3, 124.1, 62.1, 62.0, 57.4, 57.0, 49.5, 49.1, 46.2, 46.1, 23.7, 22.4, 21.7, 21.67, 21.64, 21.4; **HRMS** (ESI) *m/z* calculated $C_{28}H_{31}NNaO_5S_3$ $[M+Na]^+$ 580.1257, found 580.1253.

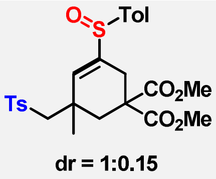

dimethyl 5-methyl-3-(*p*-tolylsulfinyl)-5-(tosylmethyl)cyclohex-3-ene-1,1-dicarboxylate (90) White Solid; dr = 1 : 0.15. (major isomer): **^{1}H-NMR** (600 MHz, $CDCl_3$) δ 7.69 (d, J = 7.8 Hz, 2H), 7.39 (d, J = 7.8 Hz, 2H), 7.28 (d, J = 7.8 Hz, 2H), 7.24 (d, J = 7.8 Hz, 2H), 5.90 (s, 1H), 3.71 (s, 6H), 3.51 (d, J = 18.0 Hz, 1H), 3.36 (dd, J = 18.1, 1.8 Hz, 1H), 3.21– 3.13 (m, 2H), 3.05 (d, J = 13.8 Hz, 1H), 2.52 (d, J = 14.4 Hz, 1H), 2.38 (s, 3H), 2.33 (s, 3H), 1.30 (s, 3H); **^{13}C-NMR** (150 MHz, $CDCl_3$) δ 171.5, 171.4, 160.3, 144.9, 141.5, 140.7, 138.2, 130.18, 130.16, 130.0, 127.6, 123.9, 64.7, 58.5, 53.4, 53.2, 46.3, 44.0, 37.6, 26.7, 21.6, 21.4; **HRMS** (ESI) *m/z* calculated $C_{26}H_{30}NaO_7S_2$ $[M+Na]^+$ 541.1325, found 541.1328.

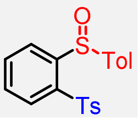

1-(*p*-tolylsulfinyl)-2-tosylbenzene (91) White Solid; mp: 156-157 °C; **^{1}H-NMR** (500 MHz, $CDCl_3$) δ 8.22 (d, J = 8.0 Hz, 1H), 8.10 (d, J = 8.0 Hz, 1H), 7.86 (d, J = 8.0 Hz, 2H), 7.76 – 7.70 (m, 3H), 7.60 (t, J = 7.5 Hz, 1H), 7.32 (d, J = 8.0 Hz, 2H), 7.25 (d, J = 8.0 Hz, 2H), 2.41 (s, 3H), 2.36 (s, 3H); **^{13}C-NMR** (150 MHz, $CDCl_3$) δ 146.5, 145.0, 142.3, 141.4, 139.3, 138.1, 134.5, 131.3, 130.1, 129.9, 129.5, 128.0, 126.4, 125.7, 21.6, 21.3; **HRMS** (ESI) *m/z* calculated $C_{20}H_{18}NaO_3S_2$ $[M+Na]^+$ 393.0590, found 393.0583.

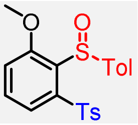

1-Methoxy-2-(*p*-tolylsulfinyl)-3-tosylbenzene (92) White Solid; mp: 199-200 °C; **^{1}H-NMR** (600 MHz, $CDCl_3$) δ 7.95 – 7.92 (m, 1H), 7.90 (d, J = 8.4 Hz, 2H), 7.59 (t, J = 7.8 Hz, 1H), 7.54 (d, J = 8.4 Hz, 2H), 7.35 (d, J = 7.8 Hz, 2H), 7.20 (d, J = 8.4 Hz, 2H), 7.11 (d, J = 8.4 Hz, 1H), 3.61 (s, 3H), 2.41 (s, 3H), 2.36 (s, 3H); **^{13}C-NMR** (150 MHz, $CDCl_3$) δ 160.2, 144.8, 143.1, 139.9, 139.4, 138.1, 133.0, 130.3, 130.0, 128.9, 128.4, 124.9, 120.9, 118.8, 56.2, 21.6, 21.2; **HRMS** (ESI) *m/z* calculated $C_{21}H_{20}NaO_4S_2$ $[M+Na]^+$ 423.0695, found 423.0687.

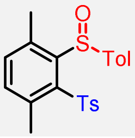

1,4-Dimethyl-2-(*p*-tolylsulfinyl)-3-tosylbenzene (93) White Solid; mp: 145-146 °C; **^{1}H-NMR** (600 MHz, $CDCl_3$) δ 7.91 (d, J = 8.4 Hz, 2H), 7.60 (d, J = 7.8 Hz, 2H), 7.35 (d, J = 8.4 Hz, 2H), 7.29 (d, J = 7.8 Hz, 1H), 7.27 – 7.23 (m, 3H), 2.82 (s, 3H), 2.42 (s, 3H), 2.38 (s, 3H), 2.25 (s, 3H); **^{13}C-NMR** (150 MHz, $CDCl_3$) δ 144.5, 143.6, 141.0, 140.6, 140.1, 140.0, 139.1, 138.6, 137.9, 135.8, 130.0, 129.3, 127.1, 126.1, 22.5, 21.6, 21.2, 18.9; **HRMS** (ESI) *m/z* calculated $C_{22}H_{22}NaO_3S_2$ $[M+Na]^+$ 421.0903, found 421.0897.

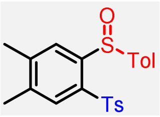

1,2-dimethyl-4-(p-tolylsulfinyl)-5-tosylbenzene (94) White Solid; mp: 149-150 °C; **^{1}H-NMR** (600 MHz, $CDCl_3$) δ 7.89 (s, 1H), 7.85 – 7.82 (m, 3H), 7.72 (d, J = 7.8 Hz, 2H), 7.29 (d, J = 7.8 Hz, 2H), 7.23 (d, J = 7.8 Hz, 2H), 2.39 (s, 3H), 2.35 (s, 3H), 2.33 (s, 3H), 2.32 (s, 3H); **^{13}C-NMR** (150 MHz, $CDCl_3$) δ 144.63, 144.58, 143.0, 142.6, 141.1, 141.0, 138.5, 136.4, 130.3, 130.0, 129.8, 127.8, 127.1, 125.6, 21.5, 21.3, 20.0, 19.7; **HRMS** (ESI) *m/z* calculated $C_{22}H_{22}NaO_3S_2$ $[M+Na]^+$ 421.0903, found 421.0894.

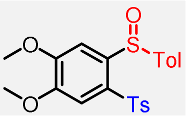

1,2-Dimethoxy-4-(*p*-tolylsulfinyl)-5-tosylbenzene (95) White Solid; mp: 168-169 °C; ^{1}**H-NMR** (600 MHz, $CDCl_3$) δ 7.84 (d, *J* = 8.4 Hz, 2H), 7.71 (d, *J* = 8.4 Hz, 2H), 7.60 (s, 1H), 7.54 (s, 1H), 7.30 (d, *J* = 7.8 Hz, 2H), 7.24 (d, *J* = 7.8 Hz, 2H), 3.97 (s, 3H), 3.93 (s, 3H), 2.40 (s, 3H), 2.36 (s, 3H); ^{13}**C-NMR** (150 MHz, $CDCl_3$) δ 153.7, 150.7, 144.6, 142.6, 141.2, 138.7, 131.2, 130.0, 129.8, 127.5, 125.4, 111.3, 107.6, 56.6, 56.5, 21.5, 21.2; **HRMS** (ESI) *m/z* calculated $C_{22}H_{22}NaO_5S_2$ $[M+Na]^+$ 453.0801, found 453.0799.

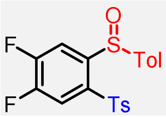

1,2-Difluoro-4-(*p*-tolylsulfinyl)-5-tosylbenzene (96) White Solid; mp: 141-142 °C; ^{1}**H-NMR** (600 MHz, $CDCl_3$) δ 8.02 (dd, *J* = 9.6, 7.2 Hz, 1H), 7.91 (dd, *J* = 9.6, 7.2 Hz, 1H), 7.84 (d, *J* = 8.4 Hz, 2H), 7.74 (d, *J* = 7.8 Hz, 2H), 7.35 (d, *J* = 8.4 Hz, 2H), 7.27 (d, *J* = 7.8 Hz, 2H), 2.43 (s, 3H), 2.38 (s, 3H); ^{13}**C-NMR** (150 MHz, $CDCl_3$) δ 154.0 (dd, *J* = 262.5, 13.5 Hz), 151.18 (dd, *J* = 258.0, 13.5 Hz), 145.6, 144.7 (t, *J* = 4.5 Hz), 142.0, 141.6, 137.4, 136.2 (t, *J* = 4.5 Hz), 130.3, 130.1, 128.0, 125.5, 119.3 (d, *J* = 21.0 Hz), 116.1 (d, *J* = 21.0 Hz), 21.6, 21.3; **HRMS** (ESI) *m/z* calculated $C_{20}H_{16}F_2NaO_3S_2$ $[M+Na]^+$ 429.0401, found 429.0392.

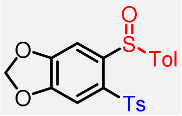

5-(*p*-tolylsulfinyl)-6-tosylbenzo[*d*][1,3]dioxole (97) White Solid; mp: 152-153 °C; ^{1}**H-NMR** (600 MHz, $CDCl_3$) δ 7.85 (d, *J* = 8.4 Hz, 2H), 7.51 (s, 1H), 7.50 (s, 1H), 7.50 (d, *J* = 8.4 Hz, 2H), 7.32 (d, *J* = 7.8 Hz, 2H), 7.25 (d, *J* = 8.4 Hz, 2H), 6.07 (ABq, *J* = 1.2 Hz, 2H),, 2.41 (s, 3H), 2.36 (s, 3H); ^{13}**C-NMR** (150 MHz, $CDCl_3$) δ 152.7, 150.0, 144.8, 142.4, 142.2, 141.3, 138.5, 133.6, 130.1, 129.8, 127.7, 125.3, 108.9, 105.9, 103.2, 21.6, 21.3; **HRMS** (ESI) *m/z* calculated $C_{21}H_{18}NaO_5S_2$ $[M+Na]^+$ 437.0488, found 437.0476.

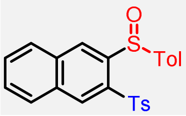

2-(*p*-tolylsulfinyl)-3-tosylnaphthalene (98) White Solid; mp: 148-149 °C; ^{1}**H-NMR** (600 MHz, $CDCl_3$) δ 8.75 (s, 1H), 8.69 (s, 1H), 8.03 (d, *J* = 7.8 Hz, 1H), 8.00 (d, *J* = 7.8 Hz, 1H), 7.86 (d, *J* = 8.4 Hz, 2H), 7.75 – 7.65 (m, 4H), 7.28 (d, *J* = 7.8 Hz, 2H), 7.21 (d, *J* = 8.4 Hz, 2H), 2.38 (s, 3H), 2.33 (s, 3H); ^{13}**C-NMR** (150 MHz, $CDCl_3$) δ 144.7, 142.8, 141.4, 140.8, 138.2, 135.5, 134.9, 133.0, 131.8, 130.1, 130.0, 129.8, 129.3, 129.2, 128.7, 127.9, 127.7, 126.1, 21.6, 21.3; **HRMS** (ESI) *m/z* calculated $C_{24}H_{20}NaO_3S_2$ $[M+Na]^+$ 443.0746, found 443.0740.

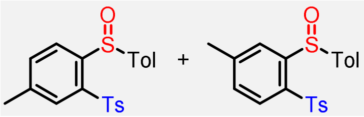

4-Methyl-1-(*p*-tolylsulfinyl)-2-tosylbenzene and 4-Methyl-2-(*p*-tolylsulfinyl)-1-tosylbenzene (99) White Solid; 1:1; ^{1}**H-NMR** (600 MHz, $CDCl_3$) δ 8.08 (d, *J* = 8.4 Hz, 1H), 7.98 (s, 1H), 7.97 (d, *J* = 7.8 Hz, 1H), 7.90 (s, 1H), 7.85 (d, *J* = 8.4 Hz, 2H), 7.82 (d, *J* = 8.4 Hz, 2H), 7.73 (d, *J* = 7.8 Hz, 2H), 7.71 (d, *J* = 8.4 Hz, 2H), 7.51 (d, *J* = 7.8 Hz, 1H), 7.37 (d, *J* = 8.4 Hz, 1H), 7.30 (d, *J* = 8.4 Hz, 2H), 7.28 (d, *J* = 8.4 Hz, 2H), 7.25 – 7.20 (m, 4H), 2.43 (s, 3H), 2.42 (s, 3H), 2.39 (s, 3H), 2.38 (s, 3H), 2.35 (s, 3H), 2.34 (s, 3H); ^{13}**C-NMR** (150 MHz, $CDCl_3$) δ 145.94, 145.90, 144.8, 144.6, 143.2, 142.6, 142.31, 142.29, 141.22, 141.16, 139.0, 138.4, 138.2, 136.4, 135.2, 132.0, 129.99, 129.95, 129.76, 129.72, 129.66, 129.60, 127.9, 127.7, 126.46, 126.45, 125.6, 125.5, 21.6, 21.52, 21.49, 21.24, 21.23, 21.18; **HRMS** (ESI) *m/z* calculated $C_{21}H_{20}NaO_3S_2$ $[M+Na]^+$ 407.0746, found 407.0750.

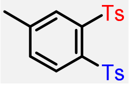

4,4'-(4-Methyl-1,2-phenylenedisulfonyl)bis(methylbenzene) (100) White Solid; mp: 140-141 °C; ^{1}**H-NMR** (500 MHz, $CDCl_3$) δ 8.33 (d, *J* = 8.0 Hz, 1H), 8.26 (s, 1H), 7.85 (d, *J* = 8.5 Hz, 2H), 7.82 (d, *J* = 8.0 Hz, 2H), 7.56 (d, *J* = 7.5 Hz, 1H), 7.30 – 7.25 (m, 4H), 2.51 (s, 3H), 2.39 (s, 3H), 2.38 (s, 3H); ^{13}**C-NMR** (125 MHz, $CDCl_3$) δ 145.2, 144.0, 143.9, 140.1, 138.9, 138.7, 137.5, 134.0, 133.5, 133.2, 129.23, 129.20, 128.0, 127.9, 21.6, 21.5, 21.4; **HRMS** (ESI) *m/z* calculated $C_{21}H_{20}NaO_4S_2$ $[M+Na]^+$ 423.0695, found 423.0696.

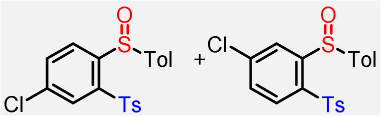

4-Chloro-1-(*p*-tolylsulfinyl)-2-tosylbenzene and 4-Chloro-2-(*p*-tolylsulfinyl)-1-tosylbenzene (101) White Solid; 1:1; ^{1}**H-NMR** (600 MHz, $CDCl_3$) δ 8.17 (d, *J* = 2.4 Hz, 1H), 8.15 (d, *J* = 8.4 Hz, 1H), 8.05 (d, *J* = 2.4 Hz, 1H), 8.00 (d, *J* = 8.4 Hz, 1H), 7.86 (d, *J* = 8.4 Hz, 2H), 7.82 (d, *J* = 8.4 Hz, 2H), 7.75 (d, *J* = 8.4 Hz, 2H), 7.72 (d, *J* = 8.4 Hz, 2H), 7.66 (dd, *J* = 8.4, 1.8 Hz, 1H), 7.52 (dd, *J* = 8.4, 2.1 Hz, 1H), 7.34 (d, *J* = 8.4 Hz, 2H), 7.32 (d, *J* = 7.8 Hz, 2H), 7.28

– 7.23 (m, 4H), 2.42 (s, 3H), 2.41 (s, 3H), 2.37 (s, 3H), 2.36 (s, 3H); ^{13}**C-NMR** (150 MHz, $CDCl_3$) δ 148.6, 145.5, 145.3, 144.9, 142.0, 141.8, 141.74, 141.69, 141.6, 140.8, 137.80, 137.78, 137.5, 137.4, 134.5, 131.6, 131.0, 130.3, 130.2, 130.03, 129.98, 129.2, 128.1, 128.1, 127.9, 126.4, 125.7, 125.6, 21.7, 21.6, 21.4, 21.3; **HRMS** (ESI) *m/z* calculated $C_{20}H_{17}ClNaO_3S_2$ $[M+Na]^+$ 427.0200, found 427.0213.

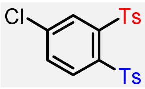

4,4'-(4-Chloro-1,2-phenylenedisulfonyl)bis(methylbenzene) (102) White Solid; mp: 143-144 °C; ^{1}**H-NMR** (600 MHz, $CDCl_3$) δ 8.43 (d, *J* = 2.4 Hz, 1H), 8.38 (d, *J* = 8.4 Hz, 1H), 7.87 (d, *J* = 8.4 Hz, 2H), 7.83 (d, *J* = 8.4 Hz, 2H), 7.73 (dd, *J* = 8.4, 2.4 Hz, 1H), 7.32 (d, *J* = 8.4 Hz, 2H), 7.30 (d, *J* = 8.4 Hz, 2H), 2.42 (s, 3H), 2.41 (s, 3H); ^{13}**C-NMR** (150 MHz, $CDCl_3$) δ 144.7, 144.5, 142.3, 140.6, 139.0, 138.3, 138.0, 134.5, 133.5, 133.0, 129.5, 129.4, 128.4, 128.2, 21.7, 21.6; **HRMS** (ESI) *m/z* calculated $C_{20}H_{17}ClNaO_4S_2$ $[M+Na]^+$ 443.0149, found 443.0133.

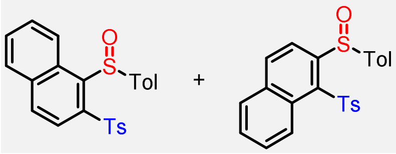

1-(*p*-tolylsulfinyl)-2-tosylnaphthalene and 2-(*p*-tolylsulfinyl)-1-tosylnaphthalene (103) White Solid; 1:1; ^{1}**H-NMR** (600 MHz, $CDCl_3$) δ 8.89 (d, *J* = 9.0 Hz, 1H), 8.79 (d, *J* = 9.0 Hz, 1H), 8.56 (d, *J* = 9.0 Hz, 1H), 8.42 (d, *J* = 9.0 Hz, 1H), 8.25 (d, *J* = 9.0 Hz, 1H), 8.14 (d, *J* = 8.4 Hz, 1H), 7.93 (d, *J* = 8.4 Hz, 2H), 7.89 (d, *J* = 8.4 Hz, 2H), 7.84 – 7.80 (m, 5H), 7.64 – 7.61 (m, 3H), 7.62 – 7.58 (m, 1H), 7.58 – 7.52 (m, 2H), 7.45 – 7.41 (m, 1H), 7.34 (d, *J* = 8.4 Hz, 2H), 7.26 – 7.20 (m, 6H), 2.40 (s, 3H), 2.36 (s, 3H), 2.34 (s, 3H), 2.33 (s, 3H); ^{13}**C-NMR** (150 MHz, $CDCl_3$) δ 149.5, 144.9, 144.7, 143.6, 141.19, 141.15, 140.3, 140.0, 139.3, 138.7, 138.3, 136.9, 135.9, 134.9, 133.6, 133.3, 130.1, 130.0, 129.9, 129.8, 129.7, 129.1, 129.1, 129.02, 128.97, 128.3, 128.0, 127.0, 126.4, 126.2, 125.1, 125.0, 123.5, 120.3, 21.6, 21.5, 21.24, 21.17; **HRMS** (ESI) *m/z* calculated $C_{24}H_{20}NaO_3S_2$ $[M+Na]^+$ 443.0746, found 443.0752.

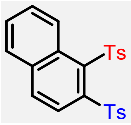

1,2-Ditosylnaphthalene (104) White Solid; mp: 178-179 °C; ^{1}**H-NMR** (600 MHz, $CDCl_3$) δ 8.84 – 8.79 (m, 2H), 8.29 (d, *J* = 8.4 Hz, 1H), 8.06 (d, *J* = 8.4 Hz, 2H), 7.94 (d, *J* = 8.4 Hz, 1H), 7.78 (d, *J* = 8.4 Hz, 2H), 7.62 (t, *J* = 7.2 Hz, 1H), 7.60 – 7.56 (m, 1H), 7.30 – 7.26 (m, 4H), 2.40 (s, 3H), 2.37 (s, 3H); ^{13}**C-NMR** (150 MHz, $CDCl_3$) δ 144.4, 143.3, 142.3, 140.9, 139.6, 137.4, 136.2, 134.8, 130.6, 129.8, 129.1, 129.01, 128.97, 128.7, 127.2, 127.0, 126.9, 126.6, 21.59, 21.57; **HRMS** (ESI) *m/z* calculated $C_{24}H_{20}NaO_4S_2$ $[M+Na]^+$ 459.0695, found 459.0683.

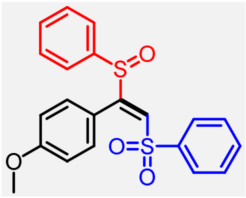

(*E*)-1-Methoxy-4-(1-(phenylsulfinyl)-2-(phenylsulfonyl)vinyl)benzene (105) White Solid; mp: 139-140 °C; ^{1}**H-NMR** (600 MHz, $CDCl_3$) δ 7.71– 7.68 (m, 2H), 7.57 (t, *J* = 7.8 Hz, 1H), 7.47 – 7.39 (m, 3H), 7.33 – 7.29 (m, 3H), 7.22 – 7.20 (m, 2H), 6.91 (d, *J* = 9.0 Hz, 2H), 6.78 (d, *J* = 9.0 Hz, 2H), 3.79 (s, 3H); ^{13}**C-NMR** (150 MHz, $CDCl_3$) δ 161.2, 161.0, 140.5, 140.4, 133.6, 131.8, 130.8, 129.03, 128.99, 128.5, 127.7, 125.1, 119.8, 113.6, 55.2; **HRMS** (ESI) *m/z* calculated $C_{21}H_{18}NaO_4S_2$ $[M+Na]^+$ 421.0539, found 421.0553.

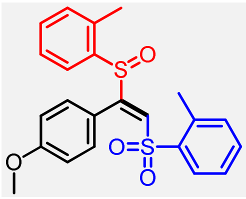

(*E*)-1-((2-(4-Methoxyphenyl)-2-(*o*-tolylsulfinyl)vinyl)sulfonyl)-2-methylbenzene (106) White Solid; mp: 151-152 °C; ^{1}**H-NMR** (600 MHz, $CDCl_3$) δ 7.68 (dd, *J* = 7.8, 0.6 Hz, 1H), 7.62 (dd, *J* = 7.8, 0.6 Hz, 1H), 7.43 (s, 1H), 7.41 (td, *J* = 7.8, 1.2 Hz, 1H), 7.35 (t, *J* = 7.2 Hz, 1H), 7.31 (td, *J* = 7.2, 1.2 Hz, 1H), 7.25 (d, *J* = 7.8 Hz, 1H), 7.14 (t, *J* = 7.8 Hz, 1H), 6.98 (d, *J* = 7.2 Hz, 1H), 6.72 (d, *J* = 8.4 Hz, 2H), 6.66 (d, *J* = 8.4 Hz, 2H), 3.76 (s, 3H), 2.61 (s, 3H), 1.86 (s, 3H); ^{13}**C-NMR** (150 MHz, $CDCl_3$) δ 161.4, 160.9, 139.3, 138.7, 138.0, 137.9, 133.6, 132.3, 131.8, 130.6, 130.4, 129.4, 129.2, 127.5, 126.2, 125.0, 119.7, 113.6, 55.3, 20.5, 18.5; **HRMS** (ESI) *m/z* calculated $C_{23}H_{22}NaO_4S_2$ $[M+Na]^+$ 449.0852, found 449.0834.

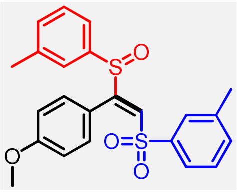

(*E*)-1-((2-(4-Methoxyphenyl)-2-(*m*-tolylsulfinyl)vinyl)sulfonyl)-3-methylbenzene (107) White Solid; mp: 139-140 °C; **^{1}H-NMR** (600 MHz, $CDCl_3$) δ 7.50 (d, *J* = 7.8 Hz, 1H), 7.45 (s, 1H), 7.37 (d, *J* = 7.8 Hz, 1H), 7.33 (t, *J* = 7.2 Hz, 1H), 7.29 (s, 1H), 7.22 (d, *J* = 7.2 Hz, 1H), 7.17 (t, *J* = 7.8 Hz, 1H), 7.10 (s, 1H), 6.93 – 6.89 (m, 3H), 6.83 – 6.77 (m, 2H), 3.82 (s, 3H), 2.35 (s, 3H), 2.30 (s, 3H); **^{13}C-NMR** (150 MHz, $CDCl_3$) δ 161.1, 160.7, 140.2, 139.5, 139.3, 134.4, 132.7, 130.9, 128.9, 128.8, 128.7, 128.2, 125.2, 124.9, 122.6, 120.1, 113.6, 55.3, 21.3, 21.2; **HRMS** (ESI) *m/z* calculated $C_{23}H_{22}NaO_4S_2$ $[M+Na]^+$ 449.0852, found 449.0854.

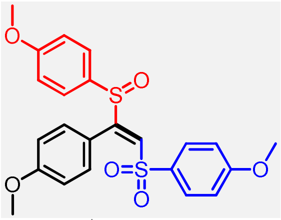

(*E*)-1-Methoxy-4-((2-(4-methoxyphenyl)-2-((4-ethoxyphenyl)sulfinyl) vinyl)sulfonyl)benzene (108) White Solid; mp: 126-127 °C; **^{1}H-NMR** (600 MHz, $CDCl_3$) δ 7.61 (d, *J* = 8.4 Hz, 2H), 7.29 (s, 1H), 7.20 (d, *J* = 8.4 Hz, 2H), 6.91 (d, *J* = 8.4 Hz, 2H), 6.89 (d, *J* = 8.4 Hz, 2H), 6.82 (d, *J* = 8.4 Hz, 2H), 6.78 (d, *J* = 8.4 Hz, 2H), 3.85 (s, 3H), 3.80 (s, 3H), 3.79 (s, 3H); **^{13}C-NMR** (150 MHz, $CDCl_3$) δ 163.7, 162.5, 161.0, 160.0, 132.1, 131.3, 130.8, 130.0, 129.1, 127.5, 120.3, 114.6, 114.2, 113.6, 55.7, 55.5, 55.2; **HRMS** (ESI) *m/z* calculated $C_{23}H_{22}NaO_6S_2$ $[M+Na]^+$ 481.0750, found 481.0754.

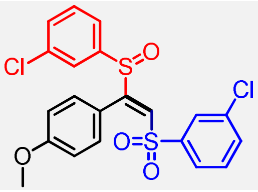

(*E*)-1-Chloro-3-((2-((3-chlorophenyl)sulfinyl)-2-(4-methoxyphenyl)vinyl)sulfonyl)benzene (109) White Solid; mp: 122-123 °C; **^{1}H-NMR** (600 MHz, $CDCl_3$) δ 7.60 (d, *J* = 7.8 Hz, 1H), 7.56 – 7.51 (m, 2H), 7.42 – 7.37 (m, 2H), 7.27 (s, 1H), 7.25 (t, *J* = 7.8 Hz, 1H), 7.19 (s, 1H), 7.00 (d, *J* = 7.8 Hz, 1H), 6.91 (d, *J* = 8.4 Hz, 2H), 6.85 (d, *J* = 8.4 Hz, 2H), 3.84 (s, 3H); **^{13}C-NMR** (150 MHz, $CDCl_3$) δ 161.7, 161.5, 142.4, 141.9, 135.5, 135.3, 133.9, 132.1, 130.9, 130.4, 130.2, 128.8, 128.1, 126.0, 124.8, 123.1, 119.2, 114.0, 55.4; **HRMS** (ESI) *m/z* calculated $C_{21}H_{16}Cl_2NaO_4S_2$ $[M+Na]^+$ 488.9759, found 488.9764.

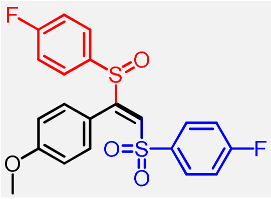

(*E*)-1-Fluoro-4-((2-((4-fluorophenyl)sulfinyl)-2-(4-methoxyphenyl)vinyl)sulfonyl)benzene (110) White Solid; mp: 150-151 °C; **^{1}H-NMR** (600 MHz, $CDCl_3$) δ 7.71 – 7.66 (m, 2H), 7.28 (s, 1H), 7.24 – 7.19 (m, 2H), 7.11 (t, *J* = 8.4 Hz, 2H), 7.03 (t, *J* = 8.4 Hz, 2H), 6.92 (d, *J* = 8.4 Hz, 2H), 6.81 (d, *J* = 8.4 Hz, 2H), 3.82 (s, 3H); **^{13}C-NMR** (150 MHz, $CDCl_3$) δ 165.7 (d, *J* = 256.0 Hz), 164.7 (d, *J* = 253.5 Hz), 161.4, 161.0, 136.4 (d, *J* = 3.0 Hz), 135.9 (d, *J* = 3.0 Hz), 130.8, 130.7 (d, *J* = 9.0 Hz), 128.7, 127.5 (d, *J* = 9.0 Hz), 119.6, 116.5 (d, *J* = 22.5 Hz), 116.3 (d, *J* = 22.5 Hz), 113.9, 55.3; **^{19}F-NMR** (564 MHz, $CDCl_3$) δ (-102.93)–(-103.00) (m), (-106.19)–(-106.27) (m); **HRMS** (ESI) *m/z* calculated $C_{21}H_{16}F_2NaO_4S_2$ $[M+Na]^+$ 457.0350, found 457.0349.

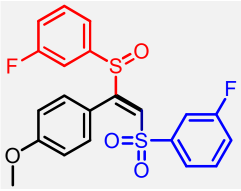

(*E*)-1-Fluoro-3-((2-((3-fluorophenyl)sulfinyl)-2-(4-methoxyphenyl)vinyl) sulfonyl)benzene (111) White Solid; mp: 93-94 °C; **^{1}H-NMR** (600 MHz, $CDCl_3$) δ 7.54 – 7.51 (m, 1H), 7.46 (td, *J* = 7.8, 5.4 Hz, 1H), 7.37 – 7.33 (m, 1H), 7.32 – 7.27 (m, 2H), 7.26 (s, 1H), 7.14 – 7.10 (m, 1H), 7.00 – 6.94 (m, 3H), 6.92 – 6.89 (m, 1H), 6.85 (d, *J* = 8.4 Hz, 2H), 3.84 (s, 3H); **^{13}C-NMR** (150 MHz, $CDCl_3$) δ 162.6 (d, *J* = 253.0 Hz), 162.3 (d, *J* = 252.6 Hz), 161.6, 161.4, 142.9 (d, *J* = 6.0 Hz), 142.3 (d, *J* = 6.6 Hz), 131.0 (d, *J* = 7.5 Hz), 130.9, 130.7 (d, *J* = 7.5 Hz), 128.4, 123.7 (d, *J* = 3.3 Hz), 121.1 (d, *J* = 21.0 Hz), 120.8 (d, *J* = 3.3 Hz), 119.4, 119.2 (d, *J* = 21.0 Hz), 115.3 (d, *J* = 24.0 Hz), 114.0, 112.1 (d, *J* = 24.0 Hz), 55.4; **^{19}F-NMR** (470 MHz, $CDCl_3$) δ (-108.98)–(-109.07) (m), (-109.21)–(-109.30) (m); **HRMS** (ESI) *m/z* calculated $C_{21}H_{16}F_2NaO_4S_2$ $[M+Na]^+$ 457.0350, found 457.0352.

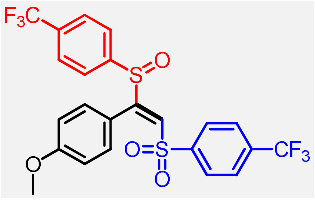

(*E*)-1-Methoxy-4-(1-((4-(trifluoromethyl)phenyl)sulfinyl)-2-((4-(trifluoromethyl)phenyl)sulfonyl)vinyl)benzene (112) White Solid; mp: 141-142 °C; **^{1}H-NMR** (600 MHz, $CDCl_3$) δ 7.81 (d, *J* = 7.8 Hz, 2H), 7.70 (d, *J* = 8.4 Hz, 2H), 7.60 (d, *J* = 8.4 Hz, 2H), 7.32 – 7.26 (m, 3H), 6.95 (d, *J* = 8.4 Hz, 2H), 6.84 (d, *J* = 8.4 Hz, 2H), 3.85 (s, 3H); **^{13}C-NMR** (150 MHz, $CDCl_3$) δ 161.8, 161.6, 144.9, 143.6, 135.4 (q, *J* = 33.0 Hz), 133.8 (q, *J* = 33.0 Hz), 131.0, 128.5, 128.4, 126.2 (q, *J* = 3.6 Hz), 126.1 (q, *J* = 3.6 Hz), 125.2, 123.2 (q, *J* = 271.5 Hz), 123.0 (q, *J* = 271.5 Hz), 119.1, 114.1, 55.4; **^{19}F-NMR** (564 MHz, $CDCl_3$) δ -63.00 (s), -63.29 (s); **HRMS** (ESI) *m/z* calculated $C_{23}H_{16}F_6NaO_4S_2$ $[M+Na]^+$ 557.0286, found 557.0275.

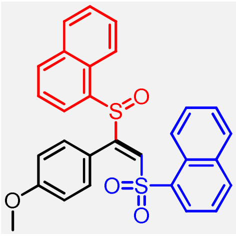

(*E*)-1-((2-(4-Methoxyphenyl)-2-(naphthalen-1-ylsulfinyl)vinyl)sulfonyl) naphthalene (113) White Solid; mp: 164-165 °C; **^{1}H-NMR** (600 MHz, $CDCl_3$) δ 8.54 (d, *J* = 8.4 Hz, 1H), 7.97 (d, *J* = 8.4 Hz, 1H), 7.92 – 7.85 (m, 3H), 7.81 (d, *J* = 8.4 Hz, 1H), 7.78 – 7.73 (m, 2H), 7.71 (s, 1H), 7.67 (t, *J* = 7.8 Hz, 1H), 7.61 (t, *J* = 7.8 Hz, 1H), 7.43 (t, *J* = 7.8 Hz, 1H), 7.40 (t, *J* = 7.8 Hz, 1H), 7.31 (t, *J* = 7.8 Hz, 1H), 7.23 (t, *J* = 7.8 Hz, 1H), 6.44 – 6.35 (m, 4H), 3.63 (s, 3H); **^{13}C-NMR** (150 MHz, $CDCl_3$) δ 161.04, 161.03, 160.7, 136.4, 135.3, 135.0, 134.0, 133.2, 132.6, 130.6, 130.4, 130.0, 129.9, 129.0, 128.6, 128.5, 127.2, 126.9, 126.6, 125.7, 125.2, 124.4, 124.1, 122.4, 119.4, 113.3, 55.2; **HRMS** (ESI) *m/z* calculated $C_{29}H_{22}NaO_4S_2$ $[M+Na]^+$ 521.0852, found 521.0859.

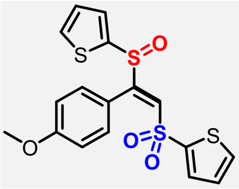

(*E*)-3-(1-(*p*-tolylsulfinyl)-2-tosylvinyl)aniline (114) White Solid; mp: 140-141 °C; **^{1}H-NMR** (600 MHz, $CDCl_3$) δ 7.66 (dd, *J* = 4.8, 1.2 Hz, 1H), 7.59 – 7.57 (m, 1H), 7.49 (dd, *J* = 3.6, 1.2 Hz, 1H), 7.45 (s, 1H), 7.12 – 7.09 (m, 1H), 7.08 – 7.03 (m, 3H), 6.92 – 6.89 (m, 1H), 6.83 – 6.80 (m, 2H), 3.78 (s, 3H); **^{13}C-NMR** (150 MHz, $CDCl_3$) δ 161.2, 159.9, 141.7, 141.3, 134.6, 134.2, 132.8, 132.4, 130.6, 129.2, 127.8, 127.2, 119.6, 113.8, 55.2; **HRMS** (ESI) *m/z* calculated $C_{17}H_{14}NaO_4S_4$ $[M+Na]^+$ 432.9667, found 432.9663.

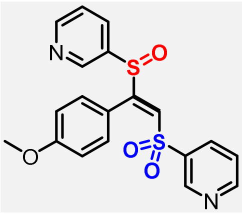

(*E*)-3-((2-(4-methoxyphenyl)-2-(pyridin-3-ylsulfinyl)vinyl)sulfonyl)pyridine (115) White Solid; mp: 148-149 °C; **^{1}H-NMR** (600 MHz, $CDCl_3$) δ 8.85 (d, *J* = 1.8 Hz, 1H), 8.79 (dd, *J* = 4.8, 1.2 Hz, 1H), 8.65 (dd, *J* = 4.8, 1.8 Hz, 1H), 8.16 (d, *J* = 1.8 Hz, 1H), 7.97 – 7.94 (m, 1H), 7.74 – 7.72 (m, 1H), 7.40 – 7.36 (m, 2H), 7.33 (s, 1H), 6.98 (d, *J* = 8.4 Hz, 2H), 6.85 (d, *J* = 9.0 Hz, 2H), 3.82 (s, 3H); **^{13}C-NMR** (150 MHz, $CDCl_3$) δ 161.8, 161.7, 154.1, 152.6, 148.7, 146.3, 137.4, 136.7, 135.4, 132.2, 130.7, 128.5, 124.3, 123.5, 118.6, 114.2, 55.3; **HRMS** (ESI) *m/z* calculated $C_{19}H_{16}N_2NaO_4S_4$ $[M+Na]^+$ 423.0444, found 423.0434.

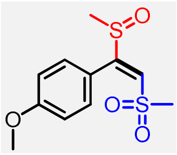

(*E*)-1-methoxy-4-(1-(methylsulfinyl)-2-(methylsulfonyl)vinyl)benzene (116) White Solid; **^{1}H-NMR** (500 MHz, $CDCl_3$) δ 7.41 – 7.39 (m, 2H), 7.12 (s, 1H), 7.01 – 6.98 (m, 2H), 3.85 (s, 3H), 2.93 (s, 3H), 2.47 (s, 3H); **^{13}C-NMR** (125 MHz, $CDCl_3$) δ 161.9, 161.4, 130.1, 127.9, 120.1, 114.7, 55.4, 43.4, 39.3; **HRMS** (ESI) *m/z* calculated $C_{11}H_{14}NaO_4S_2$ $[M+Na]^+$ 297.0226, found 297.0226.

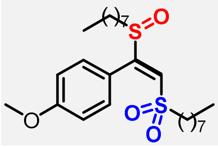

(*E*)-1-methoxy-4-(1-(octylsulfinyl)-2-(octylsulfonyl)vinyl)benzene (117) White Solid; **^{1}H-NMR** (600 MHz, $CDCl_3$) δ 7.40 (d, *J* = 9.0 Hz, 2H), 6.98 (d, *J* = 8.4 Hz, 2H), 6.97 (s, 1H), 3.84 (s, 3H), 2.98 – 2.93 (m, 2H), 2.61 – 2.55 (m, 1H), 2.40 – 2.34 (m, 1H), 1.80 – 1.71 (m, 3H), 1.61 – 1.55 (m, 1H), 1.37 – 1.33 (m, 3H), 1.30 – 1.17 (m, 17H), 0.90 – 0.83 (m, 6H); **^{13}C-NMR** (150 MHz, $CDCl_3$) δ 161.6, 159.6, 129.9, 127.1, 120.4, 114.3,

55.2, 55.1, 51.1, 31.48, 31.47, 28.8, 28.74, 28.73, 28.69, 28.2, 28.1, 22.4, 22.0, 21.0, 13.9; **HRMS** (ESI) *m/z* calculated $C_{25}H_{42}NaO_4S_2$ $[M+Na]^+$ 493.2417, found 493.2415.

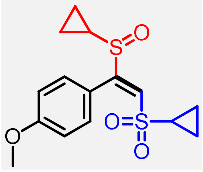

(*E*)-1-(1-(cyclopropylsulfinyl)-2-(cyclopropylsulfonyl)vinyl)-4-methoxyben zene (118) White Solid; **^{1}H-NMR** (500 MHz, $CDCl_3$) δ 7.40 (d, *J* = 9.0 Hz, 2H), 7.01 (s, 1H), 6.97 (d, *J* = 9.0 Hz, 2H), 3.84 (s, 3H), 2.42 – 2.37 (m, 1H), 1.92 – 1.87 (m, 1H), 1.26 – 1.22 (m, 1H), 1.18 – 1.43 (m, 1H), 1.04 – 0.96 (m, 3H), 0.79 – 0.71 (m, 3H); **^{13}C-NMR** (125 MHz, $CDCl_3$) δ 161.5, 159.8, 130.3, 127.4, 121.0, 114.2, 55.3, 32.0, 29.2, 5.40, 5.35, 4.1, 0.8; **HRMS** (ESI) *m/z* calculated $C_{15}H_{18}NaO_4S_2$ $[M+Na]^+$ 349.0539, found 349.0520.

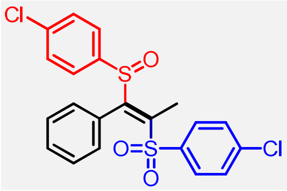

(*E*)-1-Chloro-4-((1-((4-chlorophenyl)sulfinyl)-1-phenylprop-1-en-2-yl)sulfonyl)benzene (119) White Solid; mp: 183-184 °C; **^{1}H-NMR** (600 MHz, $CDCl_3$) δ 7.33 (d, *J* = 8.4 Hz, 2H), 7.28 (t, *J* = 7.8 Hz, 1H), 7.26 – 7.22 (m, 4H), 7.14 (t, *J* = 7.2 Hz, 1H), 7.06 – 7.00 (m, 3H), 6.68 (d, *J* = 7.2 Hz, 1H), 6.20 (d, *J* = 7.2 Hz, 1H), 2.78 (s, 3H); **^{13}C-NMR** (150 MHz, $CDCl_3$) δ 153.3, 144.9, 140.3, 139.4, 137.9, 137.8, 131.0, 130.8, 129.3, 129.2, 129.1, 126.9, 126.8, 126.2, 125.6, 15.5; **HRMS** (ESI) *m/z* calculated $C_{21}H_{16}Cl_2NaO_3S_2$ $[M+Na]^+$ 472.9810, found 472.9820.

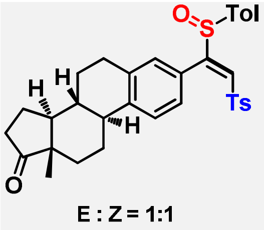

(8*R*,9*S*,13*S*,14*S*)-13-Methyl-3-((*E*)-1-(*p*-tolylsulfinyl)-2-tosylvinyl)-7,8, 9,11,12,13,15,16-octahydro-6*H*-cyclopenta[a]phenanthren-17(14*H*)-one (120) White Solid; E : Z = 1 : 1. mp: 169-170 °C; **^{1}H-NMR** (600 MHz, $CDCl_3$) δ 7.60 – 7.56 (m, 4H), 7.30 – 7.26 (m, 2H), 7.23 – 7.21 (m, 4H), 7.17 – 7.10 (m, 10H), 6.71 – 6.66 (m, 2H), 6.63 (s, 1H), 6.59 (s, 1H), 2.86 – 2.78 (m, 2H), 2.75 – 2.63 (m, 2H), 2.55 – 2.49 (m, 2H), 2.41 (s, 6H), 2.39 – 2.33 (m, 8H), 2.32 – 2.25 (m, 2H), 2.21 – 2.11 (m, 2H), 2.10 – 2.04 (m, 2H), 2.03 – 1.94 (m, 4H), 1.68 – 1.44 (m, 12H), 0.94 (s, 6H); **^{13}C-NMR** (150 MHz, $CDCl_3$) δ 220.44, 220.42, 160.6, 160.5, 144.6, 142.6, 142.0, 141.9, 137.6, 137.2, 136.3, 136.2, 129.70, 129.67, 129.59, 129.56, 129.2, 129.0, 128.9, 127.9, 126.9, 126.5, 125.5, 125.4, 125.0, 50.50, 50.47, 47.84, 47.82, 44.4, 44.3, 37.81, 37.76, 35.8, 31.5, 29.1, 29.0, 26.24, 26.20, 25.5, 25.4, 21.6, 21.5, 21.4, 13.84, 13.82; **HRMS** (ESI) *m/z* calculated $C_{34}H_{36}NaO_4S_2$ $[M+Na]^+$ 595.1947, found 595.1933. **HRMS** (ESI) *m/z* calculated $C_{34}H_{36}NaO_4S_2$ $[M+Na]^+$ 595.1947, found 595.1958.

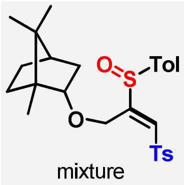

(1S,2S,4R)-1,7,7-trimethyl-2-(((E)-2-(p-tolylsulfinyl)-3-tosylallyl)oxy) bicyclo[2.2.1]heptane (121) White Solid; mixture. **^{1}H-NMR** (600 MHz, $CDCl_3$) δ 7.85 – 7.80 (m, 2H), 7.55 – 7.50 (m, 2H), 7.37 (d, *J* = 7.8 Hz, 2H), 7.31 – 7.27 (m, 2H), 7.19 – 7.14 (m, 1H), 5.25 – 5.12 (m, 1H), 4.08 – 3.94 (m, 0.5H), 3.90 (d, *J* = 15.0 Hz, 0.25H), 3.78 (d, *J* = 15.0 Hz, 0.25H), 3.54 (t, *J* = 10.2 Hz, 0.5H), 3.23 (d, *J* = 5.4 Hz, 0.25H), 3.15 (d, *J* = 5.4 Hz, 0.25H),2.46 (s, 3H), 2.41 (s, 3H), 2.14 – 1.74 (m, 1.33H), 1.74 – 1.51 (m, 3H), 1.26 – 1.11 (m, 1H), 1.03 – 0.70 (m, 12H); **^{13}C-NMR** (150 MHz, $CDCl_3$) δ 161.00, 160.95, 160.8, 145.3, 143.00, 142.97, 138.3, 138.1, 137.4, 130.30, 130.28, 130.24, 130.16, 130.14, 128.6, 128.2, 128.1, 127.8, 127.7, 127.6, 126.40, 126.35, 126.2, 88.7, 87.5, 86.7, 85.5, 63.9, 63.4, 63.0, 62.0, 49.6, 49.5, 49.43, 49.37, 48.02, 48.00, 46.59, 46.56, 45.0, 44.93, 44.86, 44.8, 38.2, 37.9, 35.8, 35.3, 34.29, 34.25, 28.14, 28.12, 27.2, 27.1, 26.6, 26.5, 21.7, 21.6, 20.23, 20.20, 20.17, 19.72, 19.70, 18.8, 14.1, 12.12, 12.08; **HRMS** (ESI) *m/z* calculated $C_{27}H_{34}NaO_4S_2$ $[M+Na]^+$ 509.1791, found 509.1796.

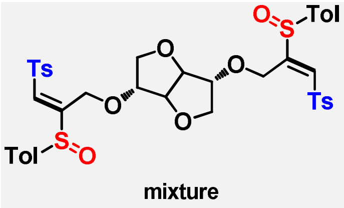

(3*R*,6*R*)-3,6-bis(((*E*)-2-(*p*-tolylsulfinyl)-3-tosylallyl)oxy)hexahydrofuro[3,2-b]furan (122) White Solid; mixture. **^{1}H-NMR** (600 MHz, $CDCl_3$) δ 7.86 – 7.76 (m, 4H), 7.58 – 7.46 (m, 4H), 7.42 – 7.34 (m, 4H), 7.31 – 7.30 (m, 4H), 7.23 – 7.19 (m, 2H), 5.45 – 5.34 (m, 1H), 5.24 (d, *J* = 14.4 Hz, 1H), 4.69 – 4.64 (m, 0.5H), 4.60 – 4.56 (m, 0.5H), 4.48 – 4.44 (m, 0.5H), 4.33 – 4.27 (m, 0.5H), 4.20 – 4.09 (m, 1.5H), 4.06 – 3.95 (m, 1.5H), 3.94 – 3.78 (m, 3.5H), 3.75 – 3.70 (m, 0.5H), 3.69 – 3.63 (m, 0.5H), 3.49 – 3.46 (m, 0.5H), 2.51 – 2.37 (m, 12H); **^{13}C-NMR** (150 MHz, $CDCl_3$) δ 159.4, 159.32, 159.27, 159.2, 158.74, 158.67, 158.63, 158.58, 145.61, 145.57, 145.55, 143.4, 143.2, 143.1, 138.3, 137.9, 137.83, 137.79, 137.1, 137.0, 136.9, 130.4, 130.33, 130.28, 130.26, 130.2, 130.08, 130.05, 129.3, 129.2, 129.1, 129.0, 128.9, 128.5, 128.3, 127.9, 127.83, 127.78, 127.4, 126.4, 126.33, 126.29, 126.26, 126.2, 85.79, 85.75, 85.4, 85.3, 84.5, 84.4, 84.3, 80.6, 80.5, 80.44, 80.39, 80.3, 80.0, 79.9, 73.2, 73.0, 72.44, 72.41, 70.4, 70.1, 70.0, 64.53, 64.48, 63.6, 63.5, 62.93, 62.91, 62.89, 62.85, 21.8, 21.6; **HRMS** (ESI) *m/z* calculated $C_{40}H_{42}NaO_{10}S_4$ $[M+Na]^+$ 833.1553, found 833.1559.

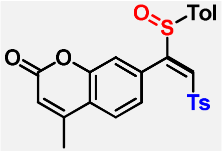

(*E*)-4-Methyl-7-(1-(*p*-tolylsulfinyl)-2-tosylvinyl)-2H-chromen-2-one (123) White Solid; mp: 177-178 °C; **^{1}H-NMR** (600 MHz, $CDCl_3$) δ 7.64 (d, *J* = 7.8 Hz, 2H), 7.50 (d, *J* = 8.4 Hz, 1H), 7.35 (s, 1H), 7.30 (d, *J* = 7.8 Hz, 2H), 7.19 – 7.14 (m, 4H), 6.92 (dd, *J* = 7.8, 1.8 Hz, 1H), 6.85 (d, *J* = 1.8 Hz, 1H), 6.35 (q, *J* = 1.2 Hz, 1H), 2.44 (s, 3H), 2.43 (d, *J* = 1.2 Hz, 3H), 2.37 (s, 3H); **^{13}C-NMR** (150 MHz, $CDCl_3$) δ 159.9, 158.3, 152.6, 151.7, 145.4, 143.5, 137.1, 136.4, 132.0, 130.5, 130.2, 130.0, 127.9, 125.7, 125.5, 124.3, 120.9, 117.1, 116.4, 21.7, 21.6, 18.6; **HRMS** (ESI) *m/z* calculated $C_{26}H_{22}NaO_5S_2$ $[M+Na]^+$ 501.0801, found 501.0800.

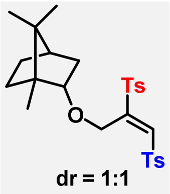

(1S,2S,4R)-2-(((*E*)-2,3-ditosylallyl)oxy)-1,7,7-trimethylbicyclo[2.2.1]heptane (124) White Solid; dr = 1:1; **^{1}H-NMR** (600 MHz, $CDCl_3$) δ 7.86 – 7.81 (m, 4H), 7.74 – 7.70 (m, 4H), 7.47 (s, 1H), 7.46 (s, 1H), 7.39 (d, *J* = 8.4 Hz, 4H), 7.35 – 7.31 (m, 4H), 4.65 – 4.61 (m, 2H), 4.59 (d, *J* = 11.4 Hz, 1H),4.49 (d, *J* = 11.4 Hz, 1H), 3.57 – 3.53 (m, 1H), 3.23 – 3.19 (m, 1H), 2.48 (s, 6H), 2.44 (m, 3H), 2.43 (m, 3H), 2.02 – 1.96 (m, 1H), 1.61 – 1.53 (m, 5H), 1.50 – 1.38 (m, 3H), 1.03 – 0.91 (m, 4H), 0.82 – 0.79 (m, 1H), 0.78 (s, 3H), 0.77 (s, 3H), 0.72 (s, 3H), 0.69 (s, 3H), 0.664 (s, 3H), 0.655 (s, 3H); **^{13}C-NMR** (150 MHz, $CDCl_3$) δ 149.6, 149.5, 145.9, 145.5, 145.4, 138.5, 138.4, 136.39, 136.37, 135.1, 134.9, 130.26, 130.25, 130.0, 129.9, 128.9, 128.8, 128.21, 128.17, 88.8, 86.4, 60.7, 60.1, 49.4, 49.2, 47.9, 46.3, 45.0, 44.8, 37.6, 35.2, 34.3, 27.9, 27.1, 26.2, 21.7, 21.6, 20.1, 19.9, 19.6, 18.8, 13.6, 11.5; **HRMS** (ESI) *m/z* calculated $C_{27}H_{34}NaO_5S_2$ $[M+Na]^+$ 525.1740, found 525.1749.

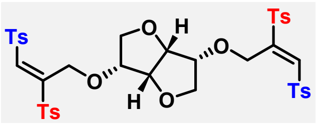

(3R,3aR,6R,6aR)-3,6-bis(((*E*)-2,3-ditosylallyl)oxy)hexahydrofuro[3,2-b]furan (125) White Solid; **^{1}H-NMR** (600 MHz, $CDCl_3$) δ 7.84 – 7.81 (m, 4H), 7.73 (d, *J* = 8.4 Hz, 2H), 7.70 (d, *J* = 8.4 Hz, 2H), 7.58 (s, 1H), 7.52 (s, 1H), 7.40 (d, *J* = 8.4 Hz, 4H), 7.35 (d, *J* = 8.4 Hz, 2H), 7.32 (d, *J* = 7.8 Hz, 2H), 4.96 (d, *J* = 12.6 Hz, 1H), 4.85 (d, *J* = 13.2 Hz, 1H), 4.77 – 4.73 (m, 2H), 4.47 (t, *J* = 3.6 Hz, 1H), 4.18 (d, *J* = 4.2 Hz, 1H), 3.85 – 3.82 (m, 1H), 3.78 (t, *J* = 3.0 Hz, 1H), 3.63 (d, *J* = 2.4 Hz, 2H), 3.38 (dd, *J* = 9.0, 6.6 Hz, 1H), 3.14 (dd, *J* = 9.0, 6.6 Hz, 1H), 2.48 (s, 3H), 2.46 (s, 3H), 2.44 (s, 3H), 2.43 (s, 3H); **^{13}C-NMR** (150 MHz, $CDCl_3$) δ 149.3, 148.6, 146.21, 146.19, 145.8, 145.7, 139.6, 139.3, 136.0, 135.9, 135.2, 135.0, 130.382, 130.375, 130.0, 129.9, 129.0, 128.8, 128.23, 128.19, 85.3, 84.0, 80.2, 79.1, 72.6, 69.9, 61.02, 59.98, 21.72, 21.70, 21.65; **HRMS** (ESI) *m/z* calculated $C_{40}H_{42}NaO_{12}S_4$ $[M+Na]^+$ 865.1451, found 865.1462.

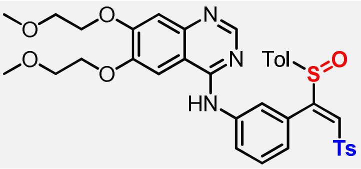

(*E*)-6,7-bis(2-methoxyethoxy)-N-(3-(1-(*p*-tolylsulfinyl)-2-tosylvinyl)phenyl)quinazolin-4-amine (126) White Solid; mp: 190-191 °C; ^{1}**H-NMR** (600 MHz, $CDCl_3$) δ 8.56 (s, 1H), 7.89 (d, *J* = 7.8 Hz, 1H), 7.81 (br., 1H), 7.61 (d, *J* = 8.4 Hz, 2H), 7.36 (s, 1H), 7.32 (s, 2H), 7.25 – 7.18 (m, 6H), 7.11 (d, *J* = 8.4 Hz, 2H), 6.63 (d, *J* = 7.2 Hz, 1H), 4.33 – 4.23 (m, 4H), 3.85 – 3.77 (m, 4H), 3.45 (s, 6H), 2.35 (s, 3H), 2.28 (s, 3H); ^{13}**C-NMR** (150 MHz, $CDCl_3$) δ 160.4, 156.1, 154.6, 153.0, 149.0, 145.0, 142.8, 138.9, 137.2, 136.7, 129.9, 129.8, 129.2, 128.64, 128.61, 127.8, 125.6, 124.3, 123.0, 121.8, 109.1, 108.3, 102.6, 70.9, 70.4, 69.2, 68.3, 59.23, 59.21, 21.6, 21.4; **HRMS** (ESI) *m/z* calculated $C_{36}H_{37}N_3NaO_7S_2$ $[M+Na]^+$ 710.1965, found 710.1950.

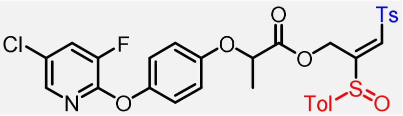

(*E*)-2-(*p*-tolylsulfinyl)-3-tosylallyl 2-(4-((5-chloro-3-fluoropyridin-2-yl)oxy)phenoxy)propanoate (127) White Solid; dr = 1 : 1; ^{1}**H-NMR** (600 MHz, $CDCl_3$) δ 7.88 – 7.84 (m, 4H), 7.82 (d, *J* = 2.4 Hz, 1H), 7.80 (d, *J* = 2.4 Hz, 1H), 7.50 – 7.46 (m, 2H), 7.46 – 7.44 (m, 4H), 7.39 (d, *J* = 7.8 Hz, 4H), 7.30 (d, *J* = 8.4 Hz, 2H), 7.27 (d, *J* = 7.8 Hz, 2H), 7.25 (d, *J* = 10.2 Hz, 2H), 7.11 – 7.07 (m, 4H), 6.92 – 6.88 (m, 4H), 5.88 (d, *J* = 13.8 Hz, 1H), 5.82 (d, *J* = 14.4 Hz, 1H), 4.76 – 4.73 (m, 2H), 4.59 (d, *J* = 14.4 Hz, 1H), 4.50 (d, *J* = 14.4 Hz, 1H), 2.47 (s, 6H), 2.41 (s, 3H), 2.40 (s, 3H), 1.63 (d, *J* = 6.6 Hz, 3H), 1.58 (d, *J* = 7.2 Hz, 3H); ^{13}**C-NMR** (150 MHz, $CDCl_3$) δ 171.0, 155.6, 155.5, 154.64, 154.60, 151.23 (d, *J* = 11.0 Hz), 151.21 (d, *J* = 11.0 Hz), 147.3, 146.97 (d, *J* = 264.2 Hz), 146.93 (d, *J* = 264.2 Hz), 145.66, 145.65, 143.8, 140.10 (d, *J* = 6.1 Hz), 140.09 (d, *J* = 11.0 Hz), 137.0, 136.9, 136.59, 136.56, 130.7, 130.6, 130.5, 130.2, 128.03, 127.99, 126.33, 126.27, 125.06 (d, *J* = 1.4 Hz), 124.95 (d, *J* = 18.1 Hz), 124.90 (d, *J* = 18.1 Hz), 124.8 (d, *J* = 1.4 Hz), 122.5, 122.4, 116.10, 116.07, 73.0, 72.8, 57.6, 57.3, 21.7, 21.6, 18.49, 18.46; **HRMS** (ESI) *m/z* calculated $C_{31}H_{27}ClFNNaO_7S_2$ $[M+Na]^+$ 666.0794, found 666.0796.

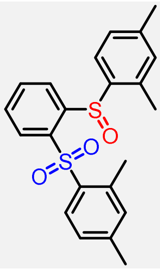

1-((2-((2,4-Dimethylphenyl)sulfinyl)phenyl)sulfonyl)-2,4-dimethylbenzene (128) White Solid; mp: 151-152 °C; ^{1}**H-NMR** (600 MHz, $CDCl_3$) δ 8.21 (dd, *J* = 8.4, 1.2 Hz, 1H), 8.02 (d, *J* = 8.4 Hz, 1H), 7.97 (dd, *J* = 7.8, 1.2 Hz, 1H), 7.81 (td, *J* = 7.2, 1.2 Hz, 1H), 7.64 (td, *J* = 7.2, 1.2 Hz, 1H), 7.16 (d, *J* = 7.8 Hz, 1H), 7.08 (d, *J* = 7.8 Hz, 1H), 7.03 – 6.97 (m, 3H), 2.52 (s, 3H), 2.35 (s, 3H), 2.30 (s, 3H), 2.29 (s, 3H); ^{13}**C-NMR** (125 MHz, $CDCl_3$) δ 145.0, 143.7, 141.7, 140.3, 139.5, 137.8, 137.7, 135.6, 133.9, 133.4, 131.9, 131.0, 130.0, 129.9, 128.0, 127.6, 127.1, 126.3, 21.4, 21.2, 19.9, 19.0; **HRMS** (ESI) *m/z* calculated $C_{22}H_{22}NaO_3S_2$ $[M+Na]^+$ 421.0903, found 421.0903.

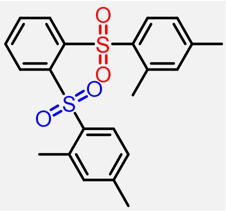

1,2-Bis((2,4-dimethylphenyl)sulfonyl)benzene (129) White Solid; mp: 148-149 °C; ^{1}**H-NMR** (500 MHz, $CDCl_3$) δ 8.29 – 8.26 (m, 2H), 7.85 (d, *J* = 8.0 Hz, 2H), 7.78 – 7.75 (m, 2H), 7.14 (d, *J* = 8.5 Hz, 2H), 7.01 (s, 2H), 2.36 (s, 6H), 2.30 (s, 6H); ^{13}**C-NMR** (125 MHz, $CDCl_3$) δ 144.2, 140.7, 136.9, 136.6, 133.1, 133.0, 132.6, 130.0, 126.4, 21.4, 20.1; **HRMS** (ESI) *m/z* calculated $C_{22}H_{22}NaO_4S_2$ $[M+Na]^+$ 437.0852, found 437.0855.

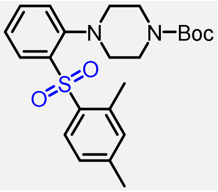

***tert*-Butyl 4-(2-((2,4-dimethylphenyl)sulfonyl)phenyl)piperazine-1-carboxylate (130)** Colorless oil; ^{1}**H-NMR** (500 MHz, $CDCl_3$) δ 8.31 (dd, *J* = 8.0, 1.5 Hz, 1H), 8.15 (d, *J* = 8.0 Hz, 1H), 7.60 (td, *J* = 8.0, 1.5 Hz, 1H), 7.43 – 7.38 (m, 1H), 7.27 – 7.24 (m, 1H), 7.19 (d, *J* = 8.0 Hz, 1H), 6.95 (s, 1H), 3.27 (br., 4H), 2.63 (br., 4H), 2.35 (s, 3H), 2.10 (s, 3H), 1.47 (s, 9H); ^{13}**C-NMR** (150 MHz, $CDCl_3$) δ 154.7, 152.3, 143.5, 137.9, 137.7, 136.3, 134.6, 132.8, 130.9, 130.3, 126.2, 125.7, 124.3, 79.8, 52.6, 42.6, 28.4, 21.3, 19.6; **HRMS** (ESI) *m/z* calculated $C_{23}H_{30}N_2NaO_4S$ $[M+Na]^+$ 453.1818, found 453.1816.

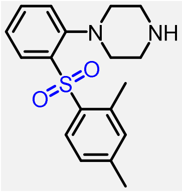

1-(2-((2,4-Dimethylphenyl)sulfonyl)phenyl)piperazine (131) Colorless oil; ^{1}**H-NMR** (500 MHz, $CDCl_3$) δ 8.27 (d, *J* = 8.0 Hz, 1H), 8.18 (d, *J* = 8.0 Hz, 1H), 7.59 (t, *J* = 7.5 Hz, 1H), 7.37 (t, *J* = 7.5 Hz, 1H), 7.30 (d, *J* = 8.0 Hz, 1H), 7.19 (d, *J* = 8.0 Hz, 1H), 6.93 (s, 1H), 2.79 (br., 1H), 2.72 – 2.61 (m, 8H), 2.33 (s, 3H), 2.08 (s, 3H); ^{13}**C-NMR** (125 MHz, $CDCl_3$) δ 152.5, 143.3, 137.8, 137.6, 136.2, 134.6, 132.6, 130.6, 130.4, 126.2, 125.6, 124.5, 53.8, 45.0, 21.2, 19.5; **HRMS** (ESI) *m/z* calculated $C_{18}H_{22}N_2NaO_2S$ $[M+Na]^+$ 353.1294, found 353.1299.

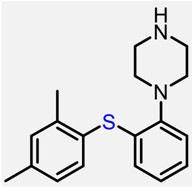

1-(2-((2,4-Dimethylphenyl)thio)phenyl)piperazine (132) White Solid; mp: 99-100 °C; ^{1}**H-NMR** (600 MHz, $CDCl_3$) δ 7.38 (d, *J* = 7.8 Hz, 1H), 7.14 (s, 1H), 7.09 – 7.04 (m, 2H), 7.02 (d, *J* = 7.8 Hz, 1H), 6.87 – 6.83 (m, 1H), 6.52 (d, *J* = 7.8 Hz, 1H), 3.08 – 3.02 (m, 8H), 2.36 (s, 3H), 2.32 (s, 3H), 1.80 (br. 1H); ^{13}**C-NMR** (150 MHz, $CDCl_3$) δ 149.7, 142.4, 139.1, 136.1, 134.6, 131.6, 128.1, 127.7, 126.2, 125.5, 124.3, 119.9, 53.1, 46.5, 21.2, 20.6; **HRMS** (ESI) *m/z* calculated $C_{18}H_{22}N_2NaS$ $[M+Na]^+$ 321.1396, found 321.1399.

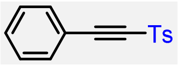

Methyl-4-((phenylethynyl)sulfonyl)benzene (133) White Solid; mp: 73-74 °C; ^{1}**H-NMR** (600 MHz, $CDCl_3$) δ 7.96 (d, *J* = 8.4 Hz, 2H), 7.53 – 7.50 (m, 2H), 7.47 (t, *J* = 7.8 Hz, 1H), 7.41 – 7.34 (m, 4H), 2.47 (s, 3H); ^{13}**C-NMR** (150 MHz, $CDCl_3$) δ 145.4, 138.9, 132.7, 131.4, 130.0, 128.6, 127.5, 118.0, 92.9, 85.6, 21.7; **HRMS** (ESI) *m/z* calculated $C_{15}H_{12}NaO_2S$ $[M+Na]^+$ 279.0450, found 279.0455.

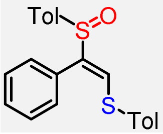

(*E*)-Phenyl(2-phenyl-2-(phenylsulfinyl)vinyl)sulfane (134) White Solid; mp: 117-118 °C; ^{1}**H-NMR** (600 MHz, $CDCl_3$) δ 7.42 (s, 1H), 7.36 (d, *J* = 7.8 Hz, 2H), 7.32 – 7.28 (m, 3H), 7.26 (d, *J* = 8.4 Hz, 2H), 7.18 – 7.15 (m, 4H), 7.11 (d, *J* = 7.8 Hz, 2H), 2.35 (s, 3H), 2.30 (s, 3H); ^{13}**C-NMR** (150 MHz, $CDCl_3$) δ 141.4, 139.8, 139.1, 138.1, 132.0, 131.5, 130.8, 130.4, 130.1, 129.5, 128.9, 128.8, 128.5, 125.0, 21.3, 21.1; **HRMS** (ESI) *m/z* calculated $C_{22}H_{20}NaOS_2$ $[M+Na]^+$ 387.0848, found 387.0844.

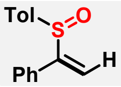

1-methyl-4-((1-phenylvinyl)sulfinyl)benzene (135) White Solid; mp: 105-106 °C; ^{1}**H-NMR** (600 MHz, $CDCl_3$) δ 7.33 (d, *J* = 8.4 Hz, 2H), 7.29 – 7.26 (m, 3H), 7.22 – 7.18 (m, 2H), 7.12 (d, *J* = 7.8 Hz, 2H), 6.24 (s, 1H), 5.92 (s, 1H), 2.30 (s, 3H); ^{13}**C-NMR** (150 MHz, $CDCl_3$) δ 154.4, 141.7, 139.5, 133.8, 129.7, 129.0, 128.6, 127.5, 125.5, 116.1, 21.4; **HRMS** (ESI) *m/z* calculated $C_{15}H_{14}NaOS$ $[M+Na]^+$ 265.0658, found 265.0655.

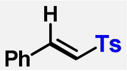

(*E*)-1-methyl-4-(styrylsulfonyl)benzene (136) White Solid; ^{1}**H-NMR** (600 MHz, $CDCl_3$) δ 7.83 (d, *J* = 8.4 Hz, 2H), 7.66 (d, *J* = 15.6 Hz, 1H), 7.48 (d, *J* = 6.6 Hz, 2H), 7.41 – 7.37 (m, 3H), 7.34 (d, *J* = 7.8 Hz, 2H), 6.85 (d, *J* = 15.6 Hz, 1H), 2.44 (s, 3H); ^{13}**C-NMR** (150 MHz, $CDCl_3$) δ 144.4, 142.0, 137.8, 132.5, 131.1, 130.0, 129.1, 128.6, 127.74, 127.65, 21.6; **HRMS** (ESI) *m/z* calculated $C_{15}H_{14}NaO_2S$ $[M+Na]^+$ 281.0607, found 281.0622.

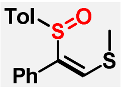

(*Z*)-methyl(2-phenyl-2-(p-tolylsulfinyl)vinyl)sulfane (137) White Solid; ^{1}**H-NMR** (600 MHz, $CDCl_3$) δ 7.28 – 7.25 (m, 5H), 7.18 (s, 1H), 7.12 – 7.10 (m, 4H), 2.45 (s, 3H), 2.31 (s, 3H); ^{13}**C-NMR** (150 MHz, $CDCl_3$) δ 141.5, 140.0, 137.8, 133.4, 131.9, 129.6, 128.9, 128.6, 128.5, 125.1, 21.4, 17.7; **HRMS** (ESI) *m/z* calculated $C_{16}H_{16}NaOS_2$ $[M+Na]^+$ 311.0535, found 311.0552.

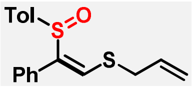

(*Z*)-allyl(2-phenyl-2-(p-tolylsulfinyl)vinyl)sulfane (138) White Solid; ^{1}**H-NMR** (600 MHz, $CDCl_3$) δ 7.29 – 7.24 (m, 5H), 7.22 (s, 1H), 7.12 – 7.10 (m, 4H), 5.91 – 5.84 (m, 1H), 5.33 – 5.29 (m, 1H), 5.23 (d, *J* = 9.6 Hz, 1H), 3.47 (d, *J* = 6.6 Hz, 2H), 2.31 (s, 3H); ^{13}**C-**

NMR (150 MHz, $CDCl_3$) δ 141.4, 140.0, 138.8, 133.3, 131.9, 131.2, 129.6, 128.9, 128.7, 128.5, 125.1, 118.8, 37.1, 21.4; **HRMS** (ESI) *m/z* calculated $C_{18}H_{18}NaOS_2$ $[M+Na]^+$ 337.0691, found 337.0690.

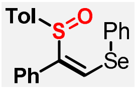

(*Z*)-phenyl(2-phenyl-2-(*p*-tolylsulfinyl)vinyl)selane (139) White Solid; **^{1}H-NMR** (600 MHz, $CDCl_3$) δ 7.74 (s, 1H), 7.60 – 7.58 (m, 2H), 7.34 – 7.30 (m, 6H), 7.25 (d, *J* = 8.4 Hz, 2H), 7.13 – 7.10 (m, 4H), 2.31 (s, 3H); **^{13}C-NMR** (150 MHz, $CDCl_3$) δ 142.5, 141.6, 139.8, 133.1, 132.6, 129.60, 129.55, 129.47, 129.1, 128.73, 128.70, 128.4, 128.2, 125.1, 21.4; **HRMS** (ESI) *m/z* calculated $C_{21}H_{18}NaOSSe$ $[M+Na]^+$ 421.0136, found 421.0156.

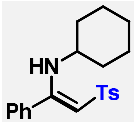

(*Z*)-N-(1-phenyl-2-tosylvinyl)cyclohexanamine (140) White Solid; **^{1}H-NMR** (600 MHz, $CDCl_3$) δ 7.81 (d, *J* = 8.4 Hz, 2H), 7.41 – 7.38 (m, 1H), 7.35 (t, *J* = 7.2 Hz, 2H), 7.30 – 7.26 (m, 4H), 4.71 (s, 1H), 3.01 – 2.98 (m, 1H), 2.42 (s, 3H), 1.75 – 1.72 (m, 2H), 1.68 – 1.65 (m, 2H), 1.50 – 1.47 (m, 1H), 1.30 – 1.24 (m, 3H), 1.17 – 1.08 (m, 3H); **^{13}C-NMR** (150 MHz, $CDCl_3$) δ 159.3, 142.7, 142.2, 135.9, 129.7, 129.5, 128.5, 127.8, 125.9, 92.9, 53.1, 34.4, 25.2, 24.6, 21.5; **HRMS** (ESI) *m/z* calculated $C_{21}H_{25}NNaO_2S$ $[M+Na]^+$ 378.1498,found 378.1512.

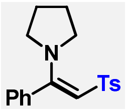

(*Z*)-1-(1-phenyl-2-tosylvinyl)pyrrolidine (141) White Solid; **^{1}H-NMR** (600 MHz, $CDCl_3$) δ 7.35 (t, *J* = 7.8 Hz, 1H), 7.31 – 7.26 (m, 4H), 7.07 (d, *J* = 7.8 Hz, 2H), 7.00 (d, *J* = 7.2 Hz, 2H), 5.24 (s, 1H), 3.23 (s, br., 2H), 2.85 (s, br., 2H), 2.35 (s, 3H), 2.07 – 1.91 (m, 2H), 1.81 – 1.69 (m, 2H); **^{13}C-NMR** (150 MHz, $CDCl_3$) δ 158.2, 143.1, 141.5, 133.9, 128.82, 128.79, 128.6, 127.9, 126.7, 95.6, 29.3, 25.3, 21.4; **HRMS** (ESI) *m/z* calculated $C_{19}H_{21}NNaO_2S$ $[M+Na]^+$ 350.1185, found 350.1183.

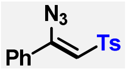

(*Z*)-1-((2-azido-2-phenylvinyl)sulfonyl)-4-methylbenzene (142) White Solid; **^{1}H-NMR** (600 MHz, $CDCl_3$) δ 7.29 – 7.26 (m, 5H), 7.21 (s, 1H), 7.14 (d, *J* = 7.8 Hz, 2H), 7.10 – 7.08 (m, 2H), 2.33 (s, 3H); **^{13}C-NMR** (150 MHz, $CDCl_3$) δ 141.8, 139.4, 134.2, 129.8, 129.7, 129.3, 129.2, 128.9, 128.4, 125.1, 21.4; **HRMS** (ESI) *m/z* calculated $C_{15}H_{13}N_3NaO_2S$ $[M+Na]^+$ 322.0621, found 322.0628.

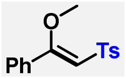

(*Z*)-1-((2-methoxy-2-phenylvinyl)sulfonyl)-4-methylbenzene (143) White Solid; **^{1}H-NMR** (600 MHz, $CDCl_3$) δ 7.44 – 7.39 (m, 3H), 7.33 – 7.28 (m, 4H), 7.13 (d, *J* = 7.8 Hz, 2H), 5.89 (s, 1H), 3.75 (s, 3H), 2.37 (s, 3H); **^{13}C-NMR** (150 MHz, $CDCl_3$) δ 169.2, 143.3, 140.2, 132.9, 130.3, 129.2, 129.1, 127.7, 127.2, 106.2, 56.7, 21.5; **HRMS** (ESI) *m/z* calculated $C_{16}H_{16}NaO_3S$ $[M+Na]^+$ 311.0712, found 311.0725.

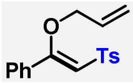

(*Z*)-1-((2-(allyloxy)-2-phenylvinyl)sulfonyl)-4-methylbenzene (144) White Solid; **^{1}H-NMR** (600 MHz, $CDCl_3$) δ 7.43 – 7.40 (m, 3H), 7.33 – 7.28 (m, 4H), 7.13 (d, *J* = 7.8 Hz, 2H), 5.96 – 5.92 (m, 1H), 5.90 (s, 1H), 5.38 – 5.33 (m, 1H), 5.30 (d, *J* = 10.8 Hz, 1H), 4.42 (d, *J* = 5.4 Hz, 2H), 2.37 (s, 3H); **^{13}C-NMR** (150 MHz, $CDCl_3$) δ 167.9, 143.2, 140.2, 132.8, 131.0, 130.3, 129.23, 129.20, 127.7, 127.2, 119.2, 107.1, 70.2, 21.5; **HRMS** (ESI) *m/z* calculated $C_{18}H_{18}NaO_3S$ $[M+Na]^+$ 337.0869, found 337.0865.

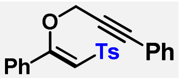

(*Z*)-1-methyl-4-((2-phenyl-2-((3-phenylprop-2-yn-1-yl)oxy)vinyl)sulfonyl) benzene (145) White Solid; **^{1}H-NMR** (600 MHz, $CDCl_3$) δ 7.71 – 7.68 (m, 2H), 7.45 – 7.39 (m, 5H), 7.28 (t, *J* = 7.2 Hz, 2H), 7.25 – 7.23 (m, 1H), 7.21 (d, *J* = 7.2 Hz, 2H), 7.09 (d, *J* = 7.8 Hz, 2H), 6.92 (s, 1H), 4.00 (s, 2H), 2.33 (s, 3H); **^{13}C-NMR** (150 MHz, $CDCl_3$) δ 157.8, 143.8, 140.7, 139.6, 138.7, 130.0, 129.8, 129.4, 129.1, 129.0, 128.5, 128.0, 126.8, 126.5, 126.0, 122.6, 30.6, 21.5; **HRMS** (ESI) *m/z* calculated $C_{24}H_{20}NaO_3S$ $[M+Na]^+$ 411.1025, found 411.1029.

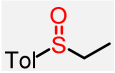

1-(ethylsulfinyl)-4-methylbenzene (146) Cololrless oil; **^{1}H-NMR** (600 MHz, $CDCl_3$) δ 7.50 (d, *J* = 7.8 Hz, 2H), 7.32 (d, *J* = 7.8 Hz, 2H), 2.91 – 2.83 (m, 1H), 2.79 – 2.72 (m, 1H), 2.42 (s, 3H), 1.19 (t, *J* = 7.2 Hz, 2H); **^{13}C-NMR** (150 MHz, $CDCl_3$) δ 141.3, 140.1, 129.8, 124.2, 50.3, 21.4, 6.0; **HRMS** (ESI) *m/z* calculated $C_9H_{12}NaOS$ $[M+Na]^+$ 191.0501, found 191.0501.

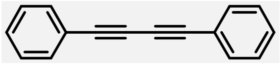

1,4-Diphenylbuta-1,3-diyne (147) White Solid; mp: 87-88 °C; **^{1}H-NMR** (600 MHz, $CDCl_3$) δ 7.55 – 7.51 (m, 4H), 7.39 – 7.32 (m, 6H); **^{13}C-NMR** (150 MHz, $CDCl_3$) δ 132.5, 129.2, 128.4, 121.8, 81.5, 73.9; **HRMS** (ESI) *m/z* calculated $C_{16}H_{10}Na$ $[M+Na]^+$ 225.0675, found 225.0678.

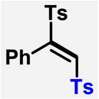

(*E*)-4,4'-(1-Phenylethene-1,2-diyldisulfonyl)bis(methylbenzene) (148) White Solid; mp: 152-153 °C; **^{1}H-NMR** (600 MHz, $CDCl_3$) δ 7.75 (s, 1H), 7.45 (d, *J* = 8.4 Hz, 2H), 7.38 – 7.34 (m, 3H), 7.22 – 7.16 (m, 6H), 6.92 (d, *J* = 7.8 Hz, 2H), 2.41 (s, 3H), 2.39 (s, 3H); **^{13}C-NMR** (150 MHz, $CDCl_3$) δ 152.7, 145.7, 145.4, 137.5, 136.3, 133.1, 130.2, 130.0, 129.8, 129.7, 129.2, 128.2, 127.7, 127.0, 21.69, 21.65; **HRMS** (ESI) *m/z* calculated $C_{22}H_{20}NaO_4S_2$ $[M+Na]^+$ 435.0695, found 435.0686.

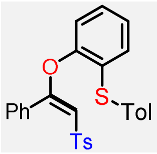

(*E*)-(2-((1-Phenyl-2-tosylvinyl)oxy)phenyl)(*p*-tolyl)sulfane (149) Yellow Liquid; **^{1}H-NMR** (600 MHz, $CDCl_3$) δ 7.62 – 7.59 (m, 2H), 7.46 – 7.42 (m, 1H), 7.40 (d, *J* = 8.4 Hz, 2H), 7.35 (t, *J* = 7.8 Hz, 2H), 7.26 (d, *J* = 8.4 Hz, 2H), 7.21 (td, *J* = 7.8, 1.2 Hz, 1H), 7.17 (d, *J* = 8.4 Hz, 2H), 7.11 – 7.05 (m, 4H), 6.97 (dd, *J* = 7.8, 1.2 Hz, 1H), 5.73 (s, 1H), 2.37 (s, 3H), 2.32 (s, 3H); **^{13}C-NMR** (150 MHz, $CDCl_3$) δ 167.4, 149.4, 143.2, 139.5, 138.7, 133.6, 131.6, 131.3, 130.6, 130.5, 130.4, 129.7, 129.0, 128.2, 127.7, 127.6, 127.2, 127.1, 122.4, 110.2, 21.5, 21.2; **HRMS** (ESI) *m/z* calculated $C_{28}H_{24}NaO_3S_2$ $[M+Na]^+$ 495.1059, found 495.1045.

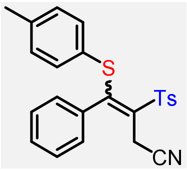

4-Phenyl-4-(*p*-tolylthio)-3-tosylbut-3-enenitrile (150) White Solid; E : Z = 1 : 2; **^{1}H-NMR** (600 MHz, $CDCl_3$) δ 8.21 (d, *J* = 8.4 Hz, minor, 2H), 7.42 (d, *J* = 8.4 Hz, minor, 2H), 7.21 (d, *J* = 8.4 Hz, major, 2H), 7.15 – 7.10 (m, minor, 3H), 7.04 (d, *J* = 8.4 Hz, major, 2H), 7.02 (t, *J* = 7.8 Hz, major, 1H), 7.00 – 6.97 (m, minor, 2H), 6.93 (d, *J* = 7.8 Hz, major, 2H), 6.90 (t, *J* = 7.8 Hz, major, 2H), 6.81 (d, *J* = 7.8 Hz, major, 2H), 6.73 (d, *J* = 7.8 Hz, minor, 2H), 6.69 (d, *J* = 7.2 Hz, major, 2H), 6.58 (d, *J* = 8.4 Hz, minor, 2H), 4.13 (s, major, 2H), 3.46 (s, minor, 2H), 2.48 (s, minor, 3H), 2.33 (s, major, 3H), 2.15 (s, major, 3H), 2.12 (s, minor, 3H); **^{13}C-NMR** (150 MHz, $CDCl_3$) δ 159.4, 158.5, 144.9, 143.9, 139.7, 139.1, 137.27, 136.6, 135.4, 134.5, 132.89, 129.7, 129.40, 129.37, 129.3, 129.2, 129.1, 128.8, 128.41, 128.37, 128.1, 127.4, 127.1, 126.9, 126.7, 126.4, 124.5, 117.0, 115.7, 21.6, 21.4, 21.1, 21.0, 20.9, 19.6 **HRMS** (ESI) *m/z* calculated $C_{24}H_{21}NNaO_2S_2$ $[M+Na]^+$ 442.0906, found 442.0897.

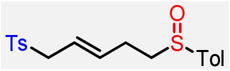

(*E*)-1-methyl-4-((5-(*p*-tolylsulfinyl)pent-2-en-1-yl)sulfonyl)benzene (151) White Solid; **^{1}H-NMR** (600 MHz, $CDCl_3$) δ 7.73 (d, *J* = 8.4 Hz, 2H), 7.32 (d, *J* = 8.4 Hz, 2H), 7.21 (d, *J* = 8.4 Hz, 2H), 7.10 (d, *J* = 8.4 Hz, 2H), 5.60 – 5.51 (m, 1H), 5.51 – 5.39 (m, 1H), 3.73 (d, *J* = 7.2 Hz, 2H), 2.79 (t, *J* = 7.2 Hz, 2H), 2.43 (s, 3H), 2.32 (s, 3H), 2.29 (q, *J* = 7.2 Hz, 2H); **^{13}C-NMR** (150 MHz, $CDCl_3$) δ 144.7, 138.8, 136.5, 135.4, 132.0, 130.4, 129.8, 129.7, 128.6, 118.1, 60.1, 33.6, 32.2, 21.7, 21.0; **HRMS** (ESI) *m/z* calculated $C_{19}H_{22}NaO_3S_2$ $[M+Na]^+$ 385.0903, found 385.0902.

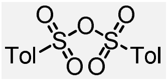

***p*-Toluenesulfonic anhydride (152):** White Solid; mp: 120-121 °C; **^{1}H-NMR** (500 MHz, $CDCl_3$) δ 7.76 (d, *J* = 8.0 Hz, 4H), 7.27 (d, *J* = 8.0 Hz, 4H), 2.38 (s, 6H); **^{13}C-NMR** (125 MHz, $CDCl_3$) δ 145.9, 132.4, 129.2, 127.9, 21.1; **HRMS** (ESI) *m/z* calculated $C_{14}H_{14}NaO_5S_2$ $[M+Na]^+$ 349.0175, found 349.0173.

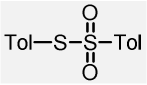

***p*-Tolyl *p*-toluenethiolsulfonate (153):** White Solid; mp: 79-80 °C; **^{1}H-NMR** (500 MHz, $CDCl_3$) δ 7.46 (d, *J* = 8.5 Hz, 2H), 7.25 – 7.20 (m, 4H), 7.14 (d, *J* = 8.0 Hz, 2H), 2.42 (s, 3H), 2.38 (s, 3H); **^{13}C-NMR** (125 MHz, $CDCl_3$) δ 144.6, 142.1, 140.5, 136.5, 130.2, 129.4, 127.6, 124.6, 21.7, 21.5; **HRMS** (ESI) *m/z* calculated $C_{14}H_{14}NaO_2S_2$ $[M+Na]^+$ 301.0327, found 301.0343.

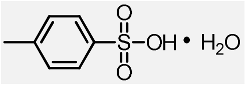

***p*-Toluenesulfonic acid monohydrate (154):** White Solid; mp: 108-109 °C; **^{1}H-NMR** (600 MHz, DMSO) δ 7.51 (d, *J* = 8.4 Hz, 2H), 7.15 (d, *J* = 8.4 Hz, 2H), 6.99 (s, br., 3H), 2.30 (s, 3H); **^{13}C-NMR** (150 MHz, DMSO) δ 145.3, 138.7, 128.7, 126.0, 21.3; **HRMS** (ESI) *m/z* calculated $C_7H_{11}O_4S$ $[M+H]^+$ 191.0373, found 191.0355.

X. Copies of NMR spectra

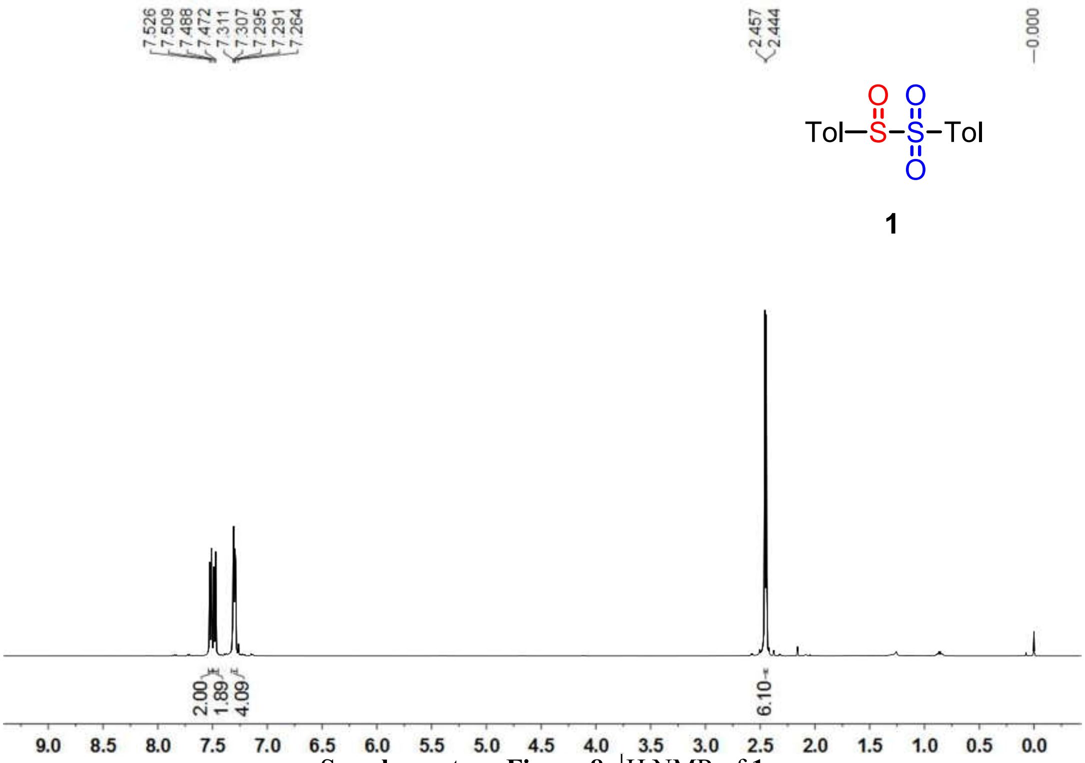

Supplementary Figure 8. ^{1}H NMR of **1**

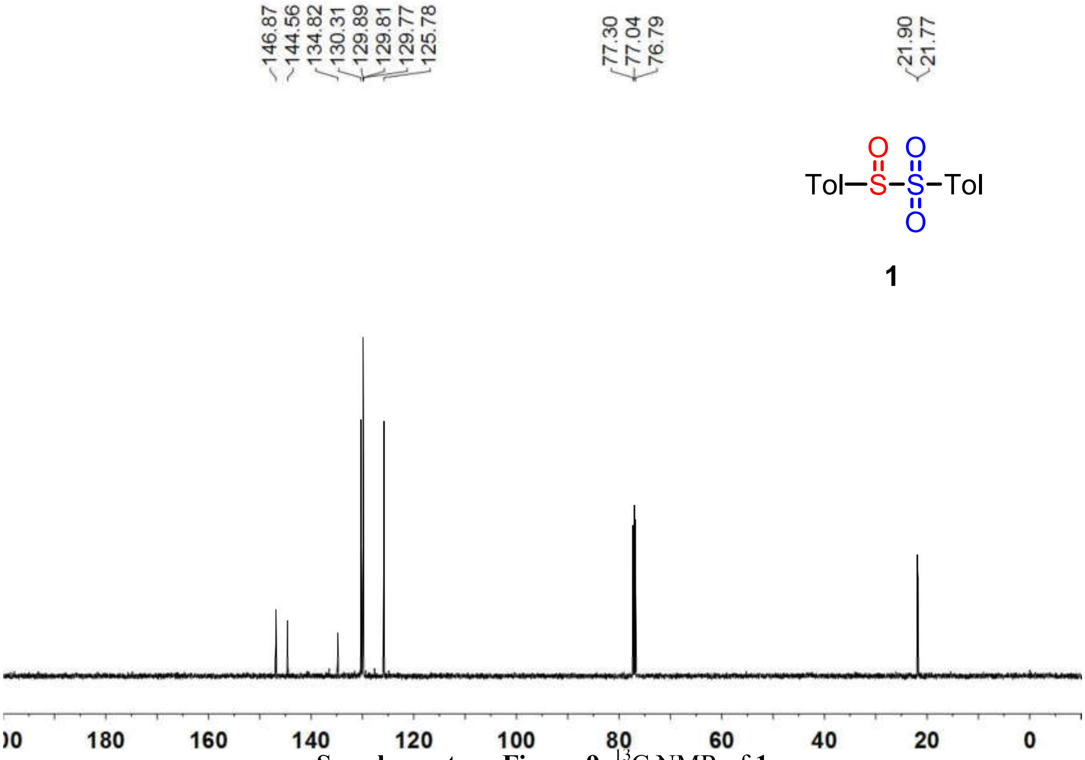

Supplementary Figure 9. ^{13}C NMR of **1**

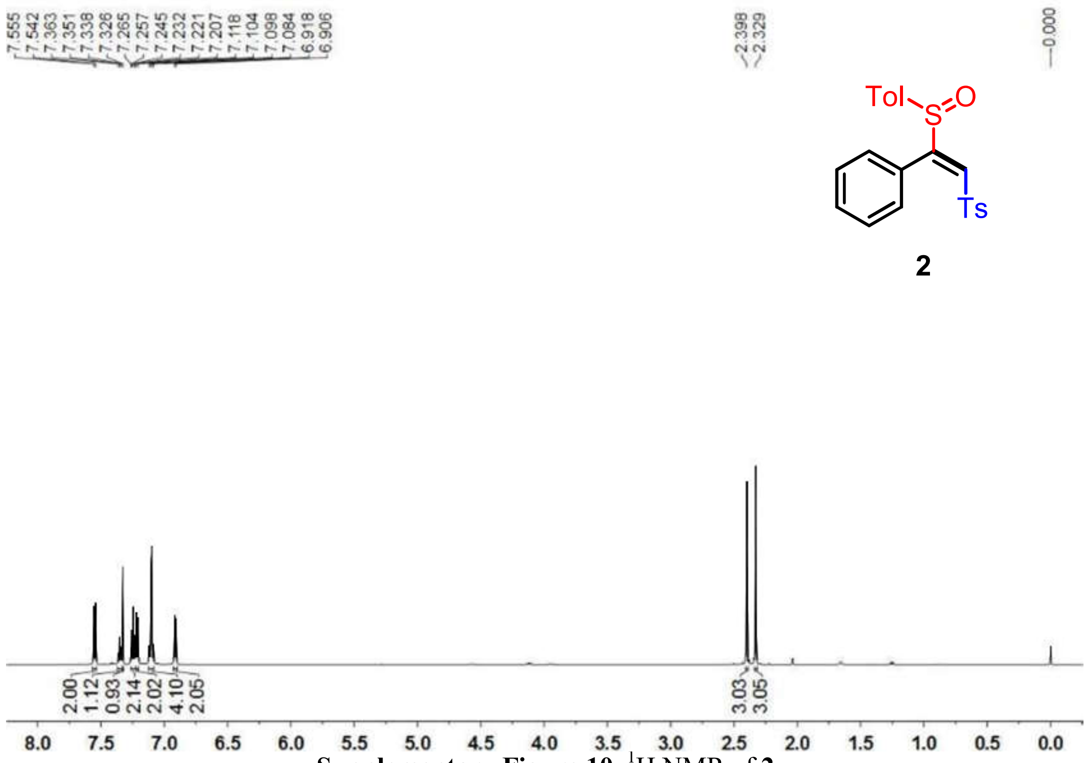

Supplementary Figure 10. ^{1}H NMR of **2**

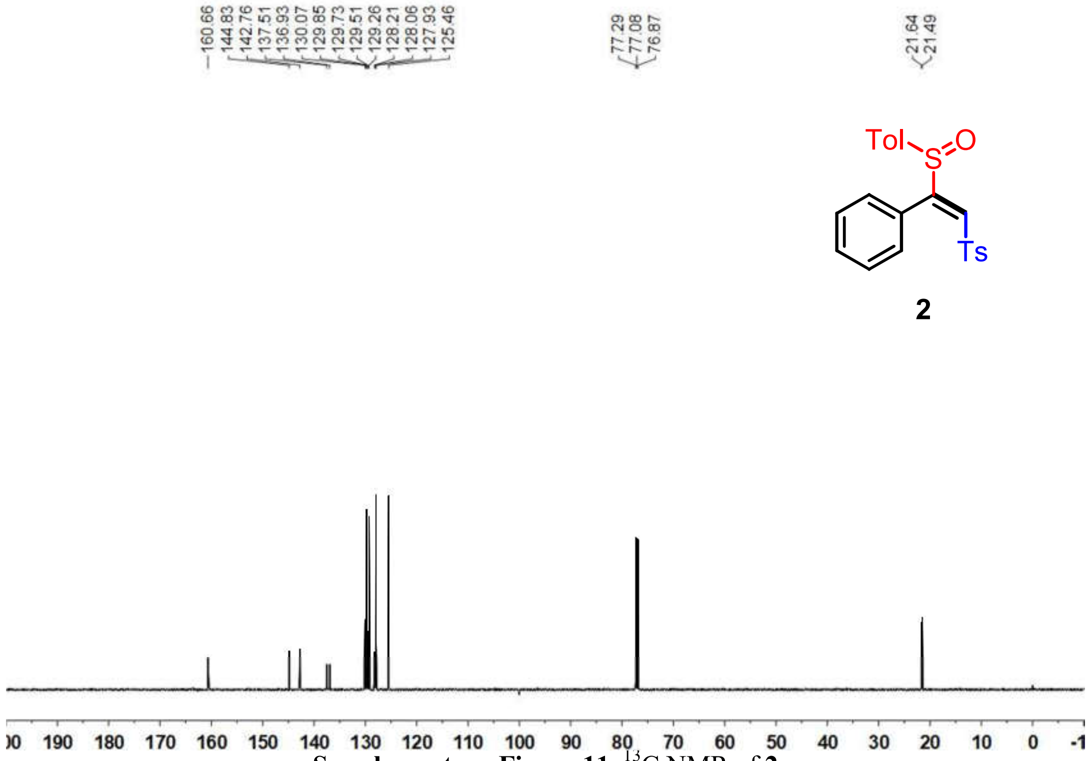

Supplementary Figure 11. ^{13}C NMR of **2**

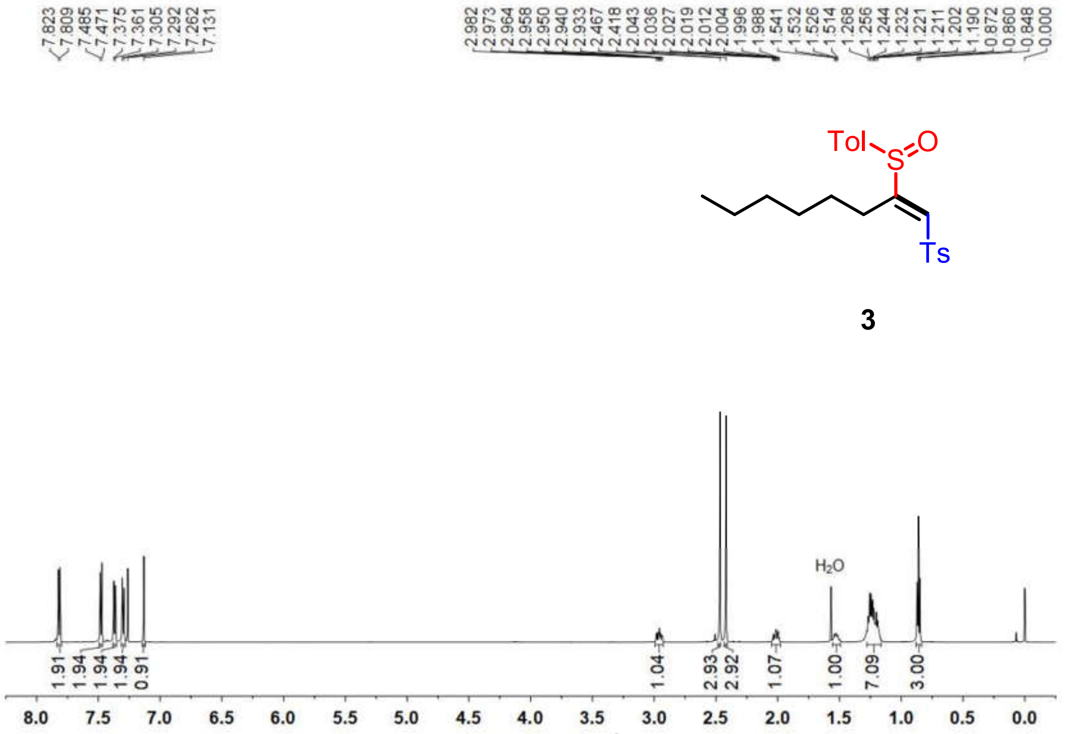

3

Supplementary Figure 12. ^{1}H NMR of **3**

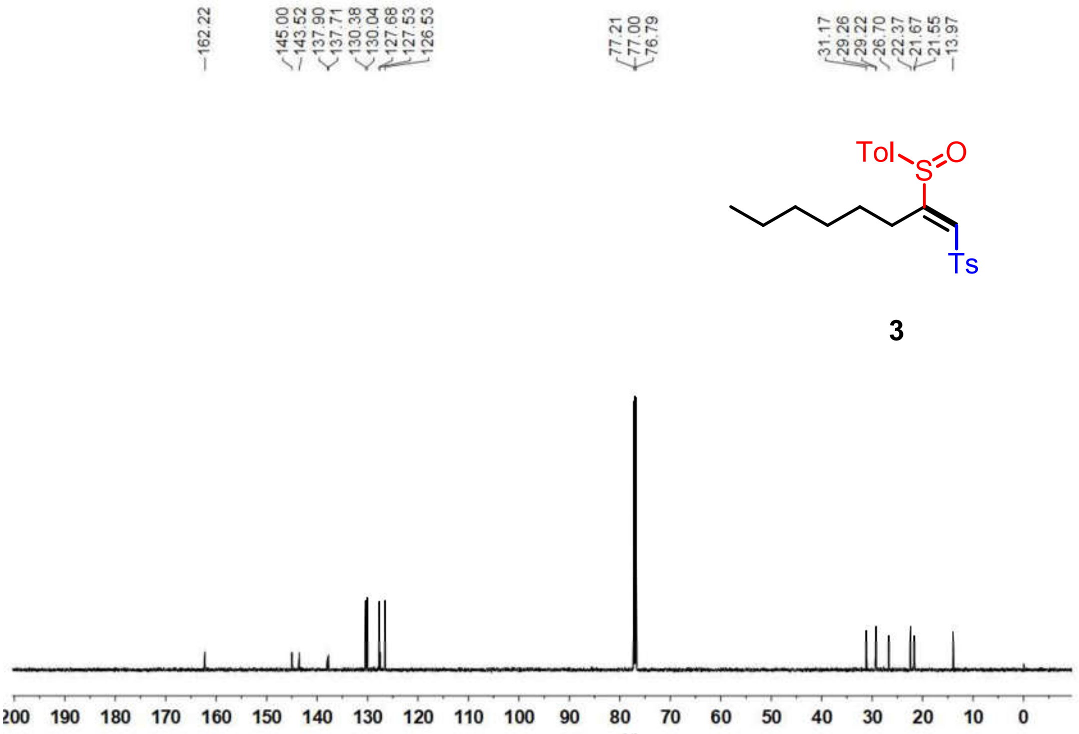

3

Supplementary Figure 13. ^{13}C NMR of **3**

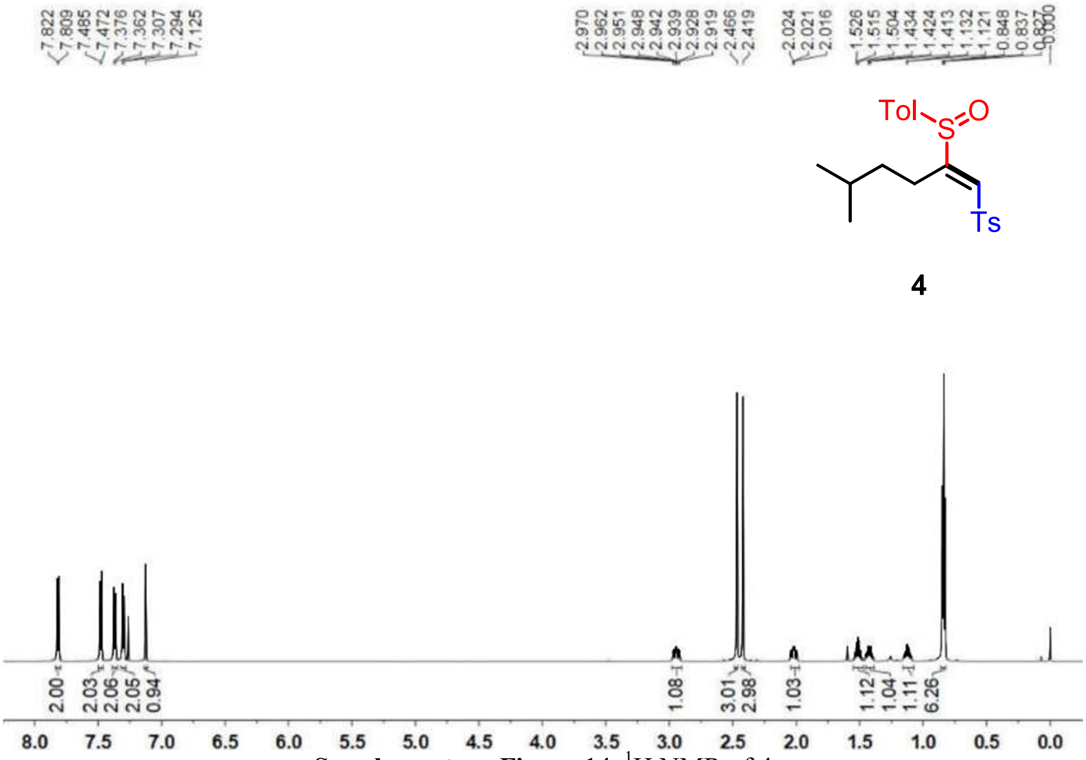

Supplementary Figure 14. ^{1}H NMR of **4**

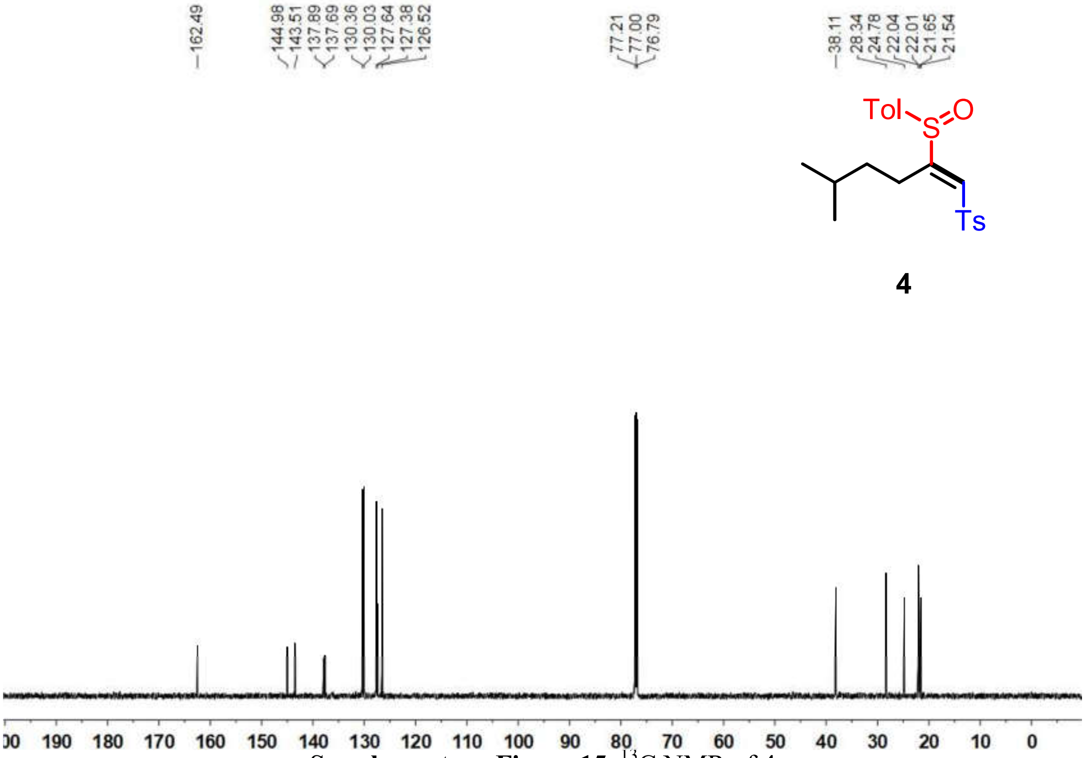

Supplementary Figure 15. ^{13}C NMR of **4**

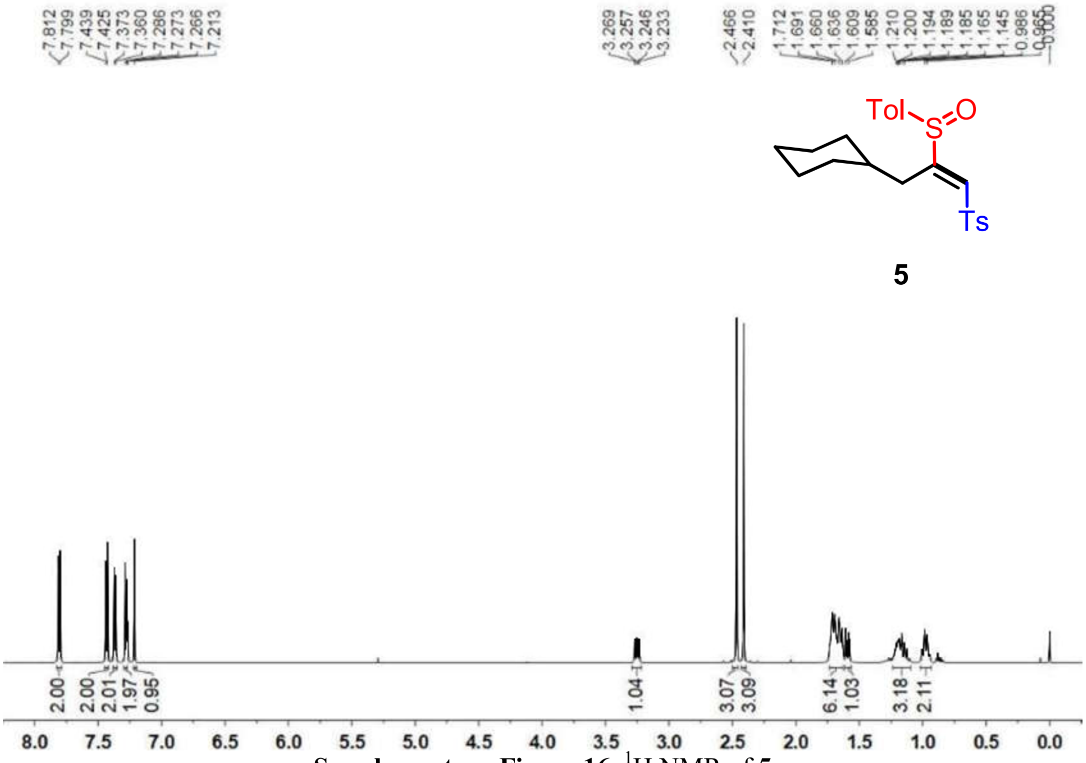

Supplementary Figure 16. ^{1}H NMR of **5**

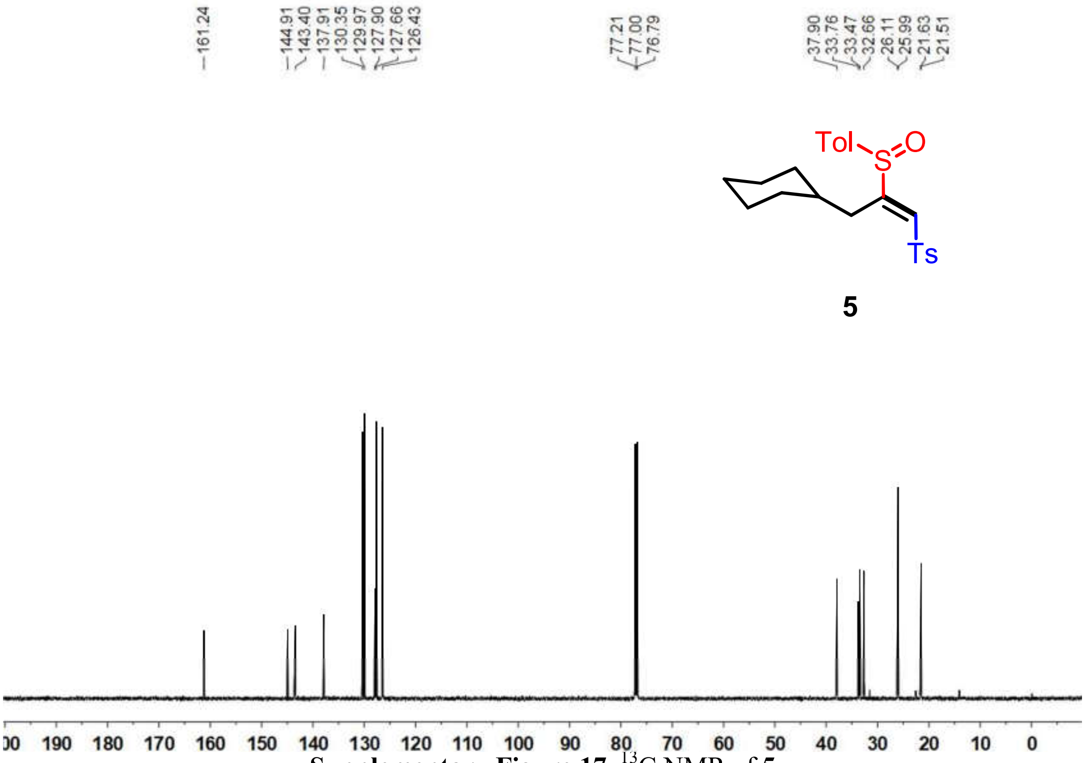

Supplementary Figure 17. ^{13}C NMR of **5**

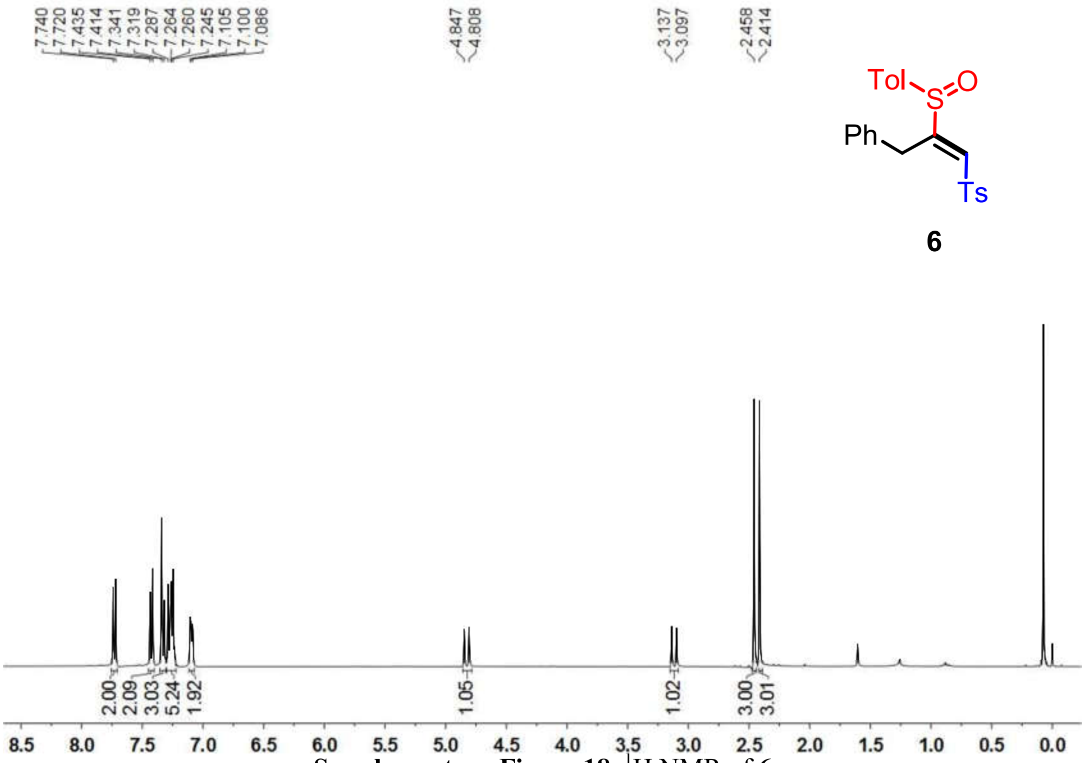

Supplementary Figure 18. ^{1}H NMR of **6**

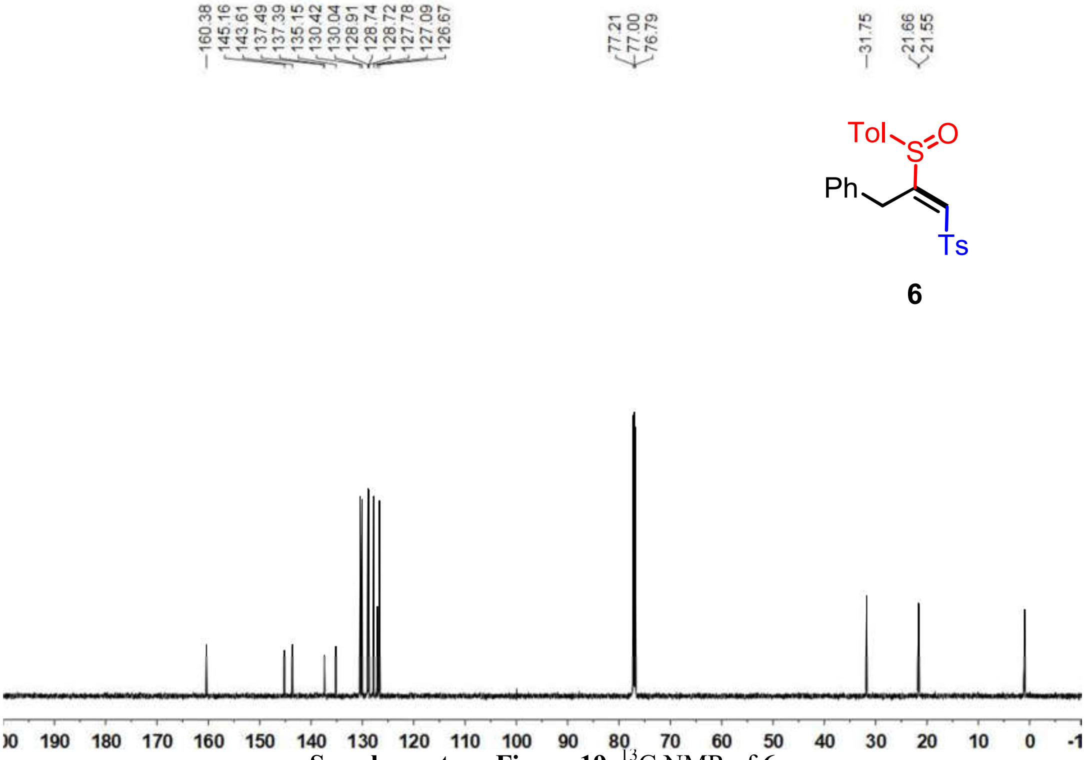

Supplementary Figure 19. ^{13}C NMR of **6**

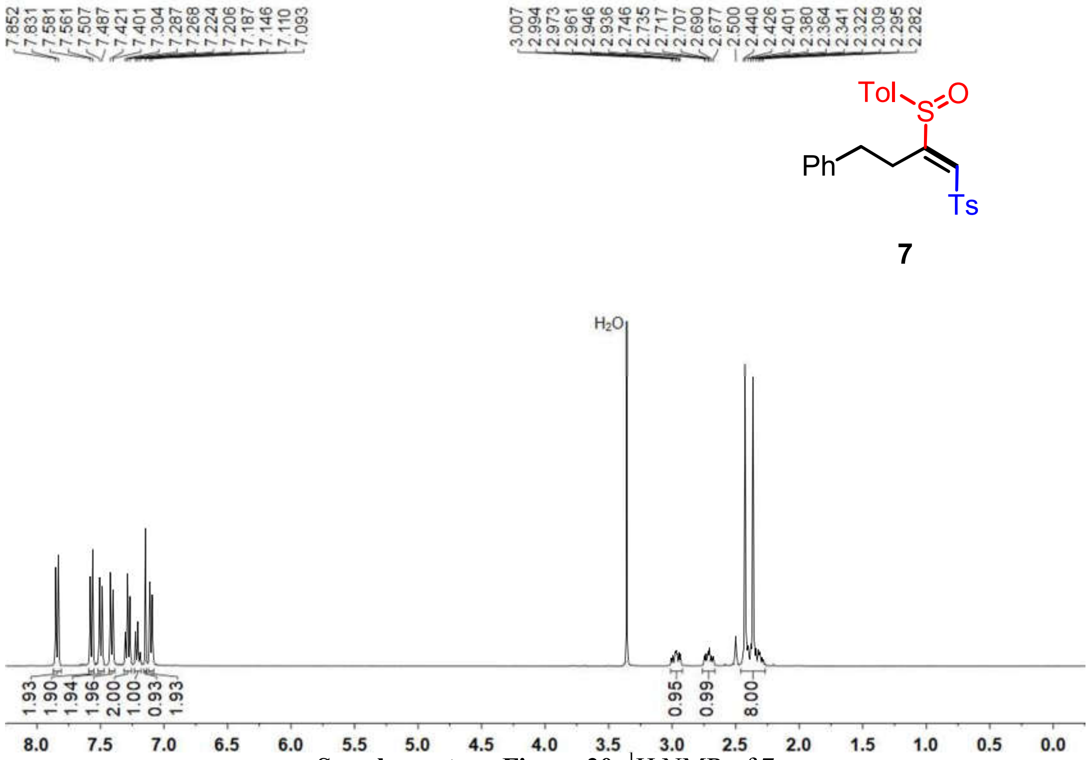

Supplementary Figure 20. ^{1}H NMR of **7**

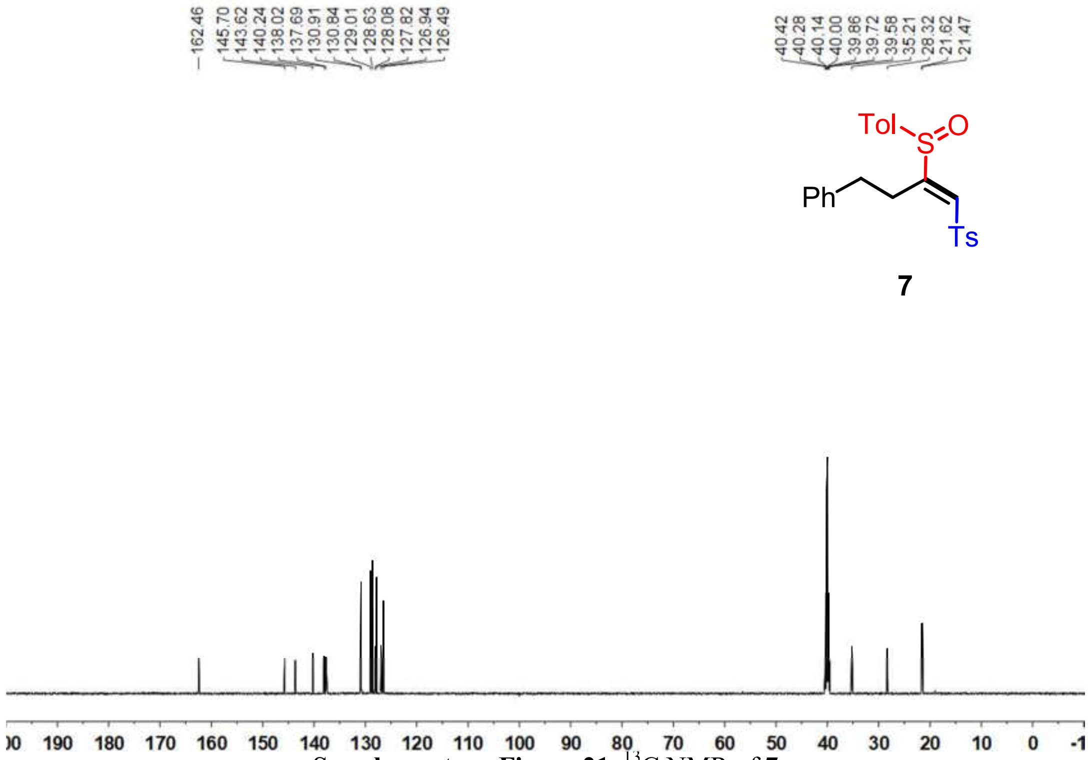

Supplementary Figure 21. ^{13}C NMR of **7**

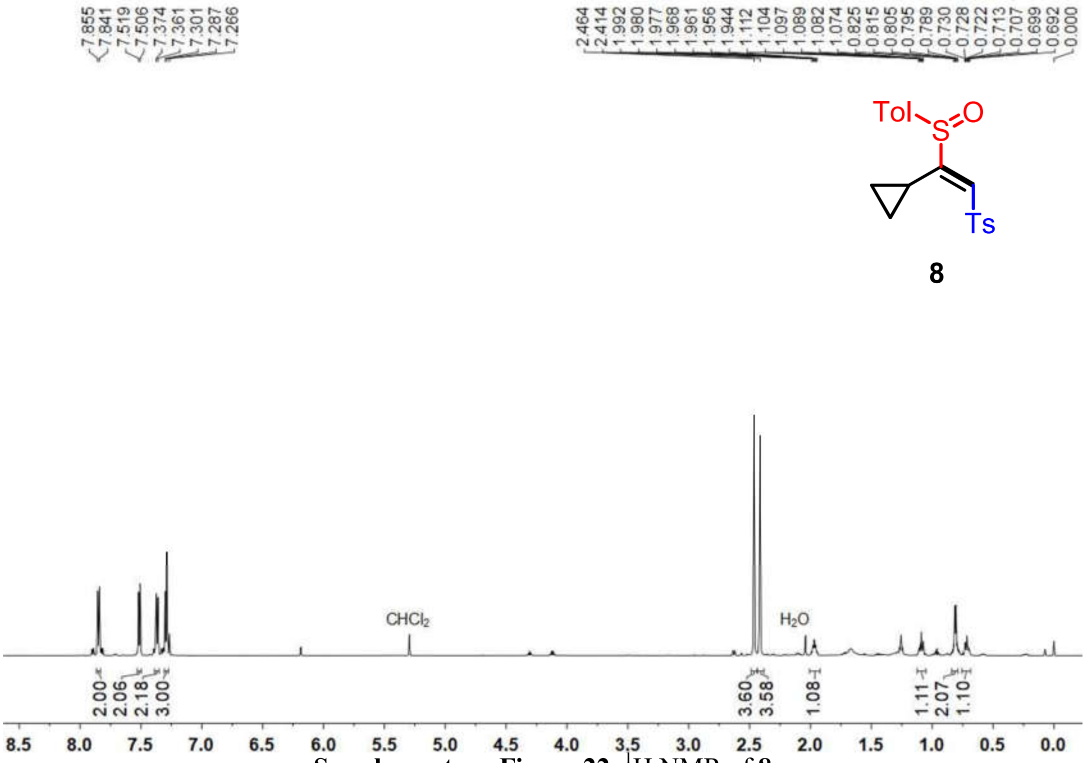

Supplementary Figure 22. ^{1}H NMR of **8**

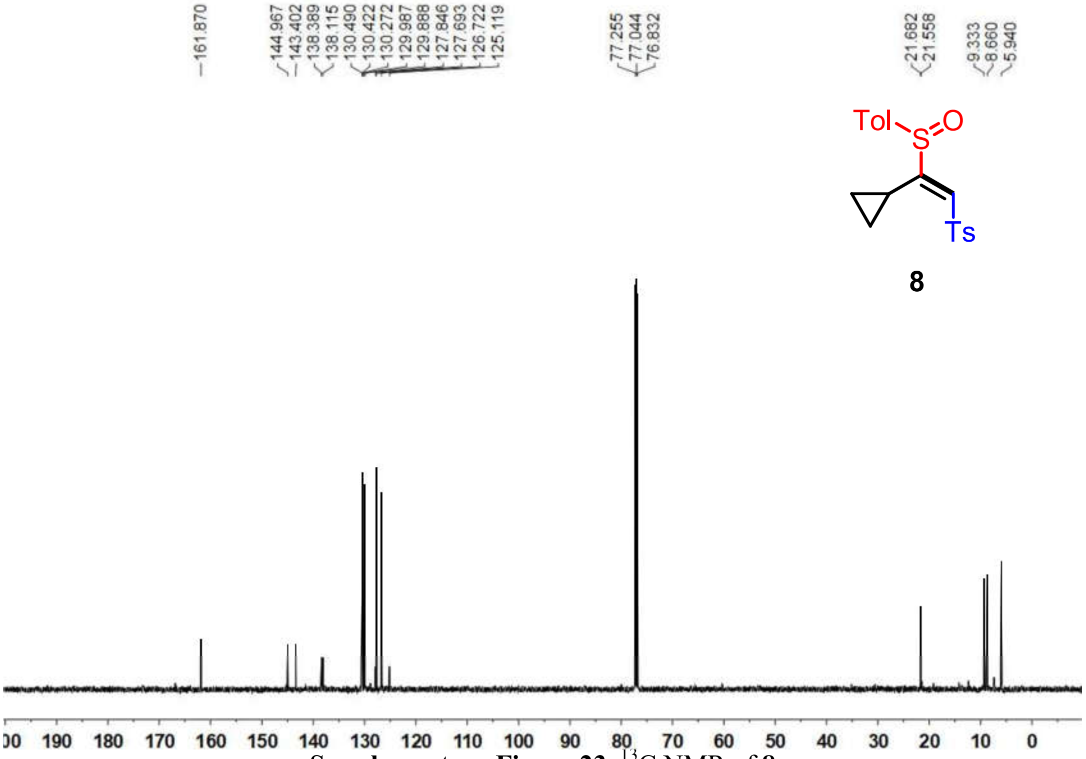

Supplementary Figure 23. ^{13}C NMR of **8**

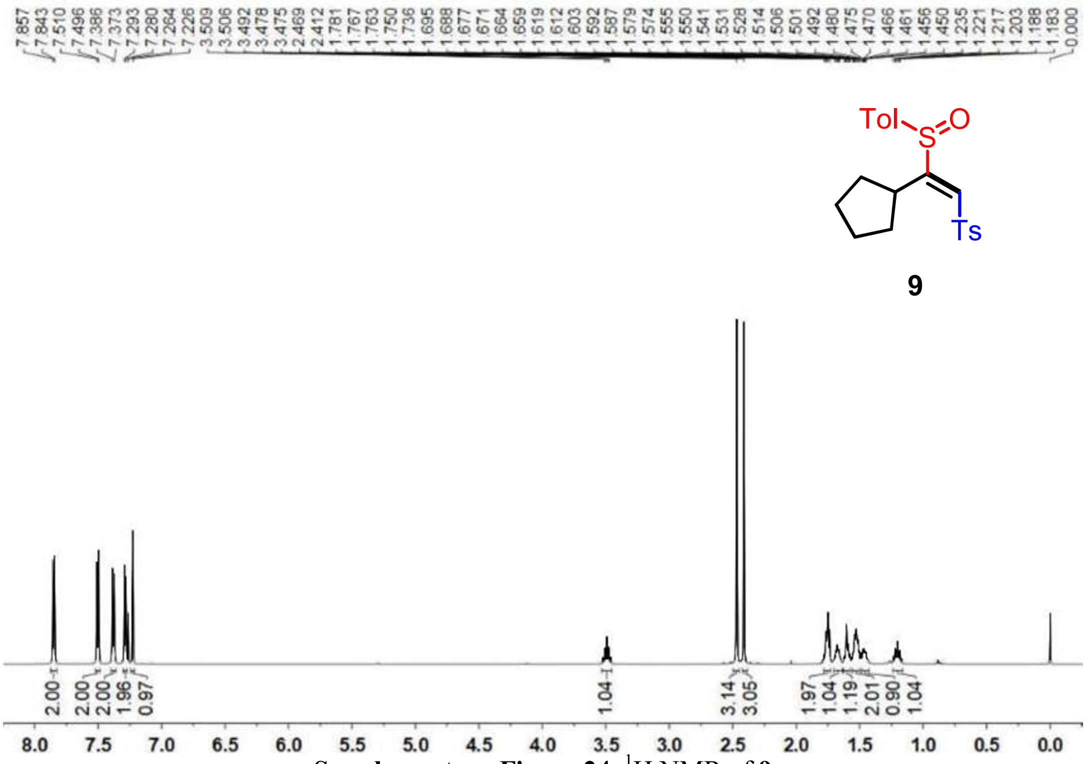

Supplementary Figure 24. ^{1}H NMR of **9**

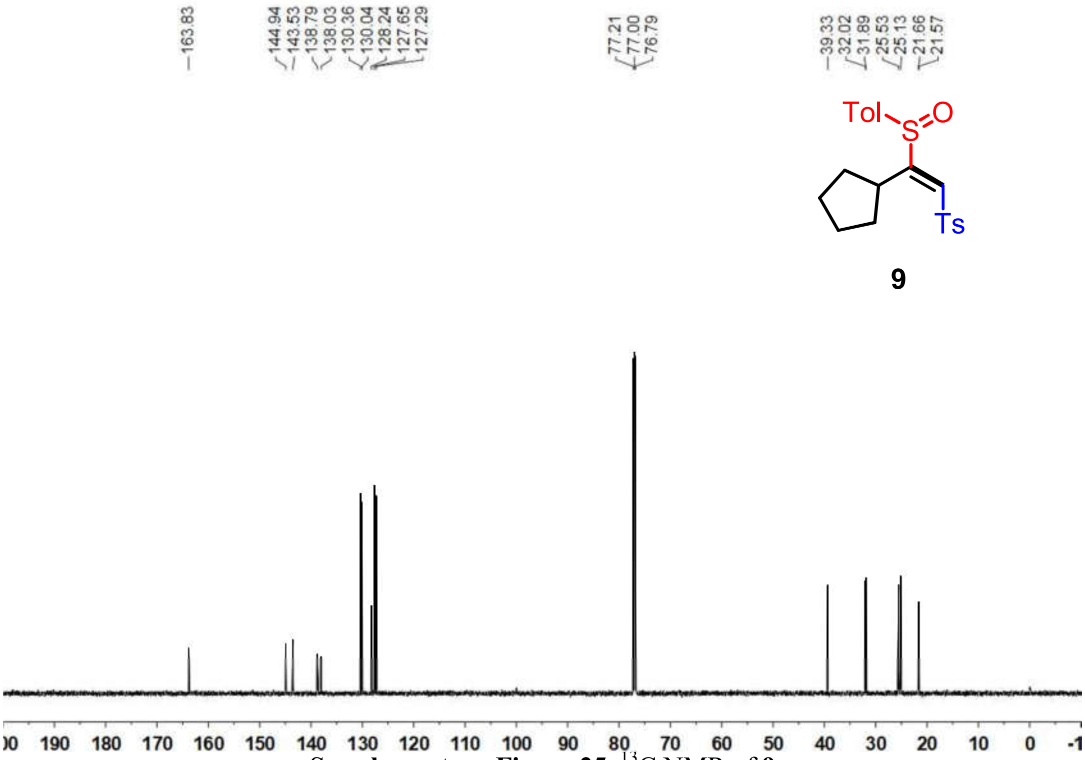

Supplementary Figure 25. ^{13}C NMR of **9**

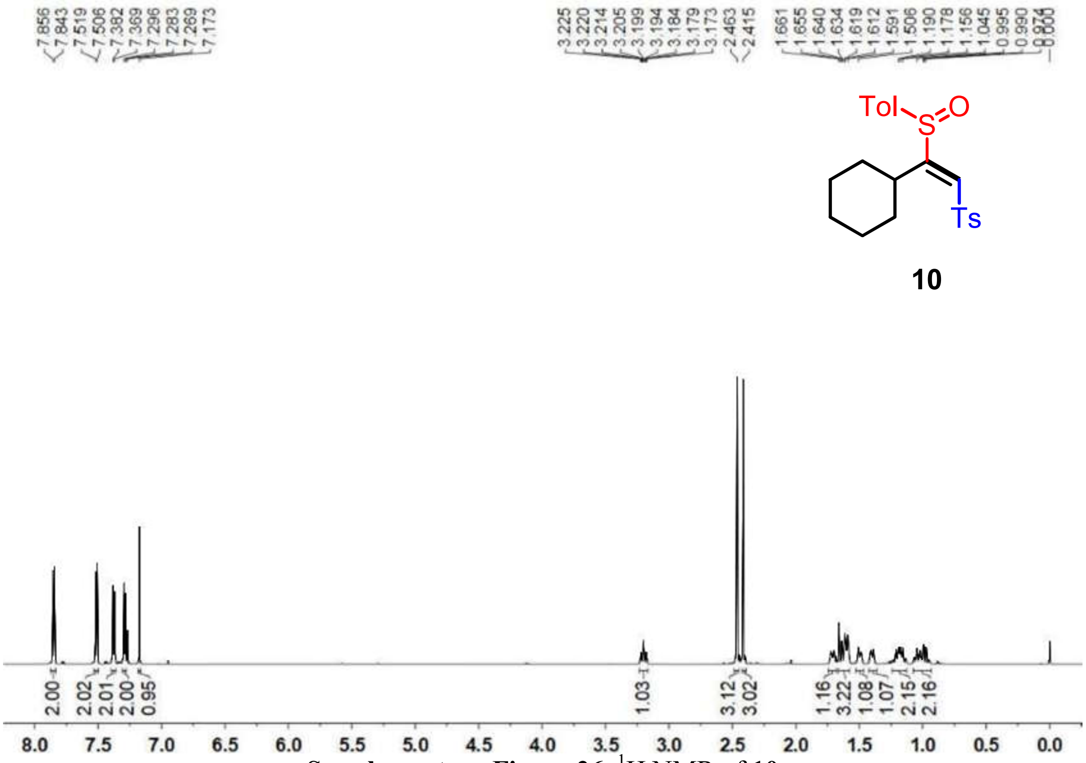

Supplementary Figure 26. ^{1}H NMR of **10**

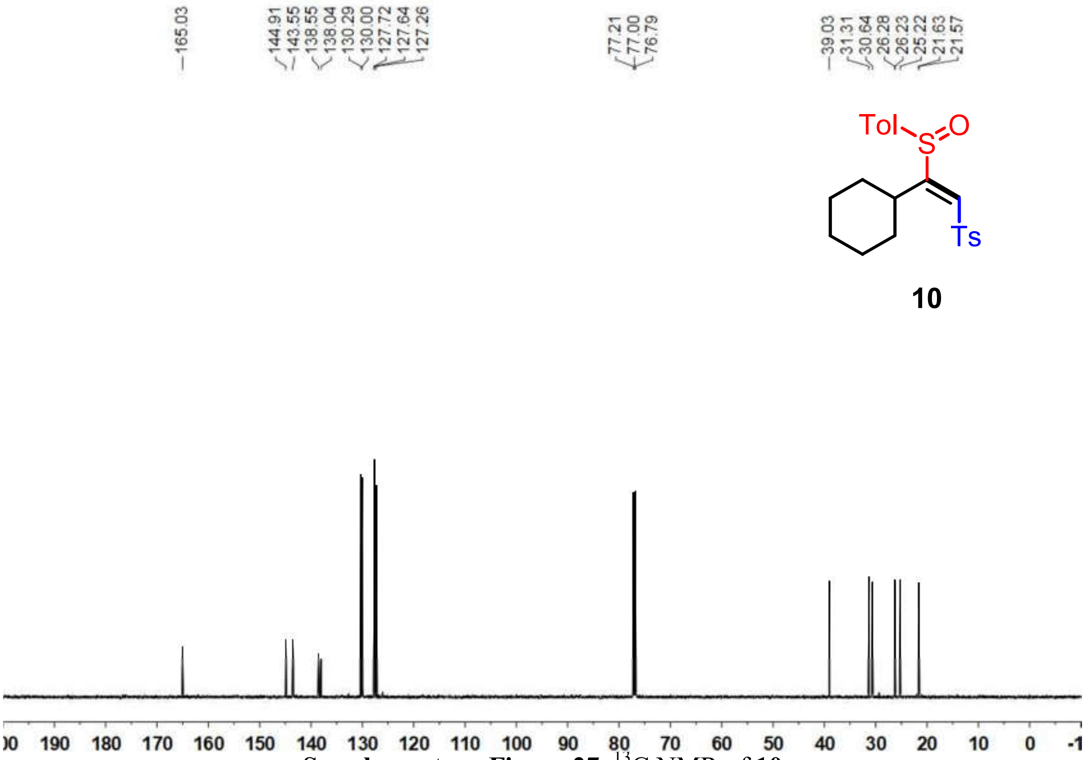

Supplementary Figure 27. ^{13}C NMR of **10**

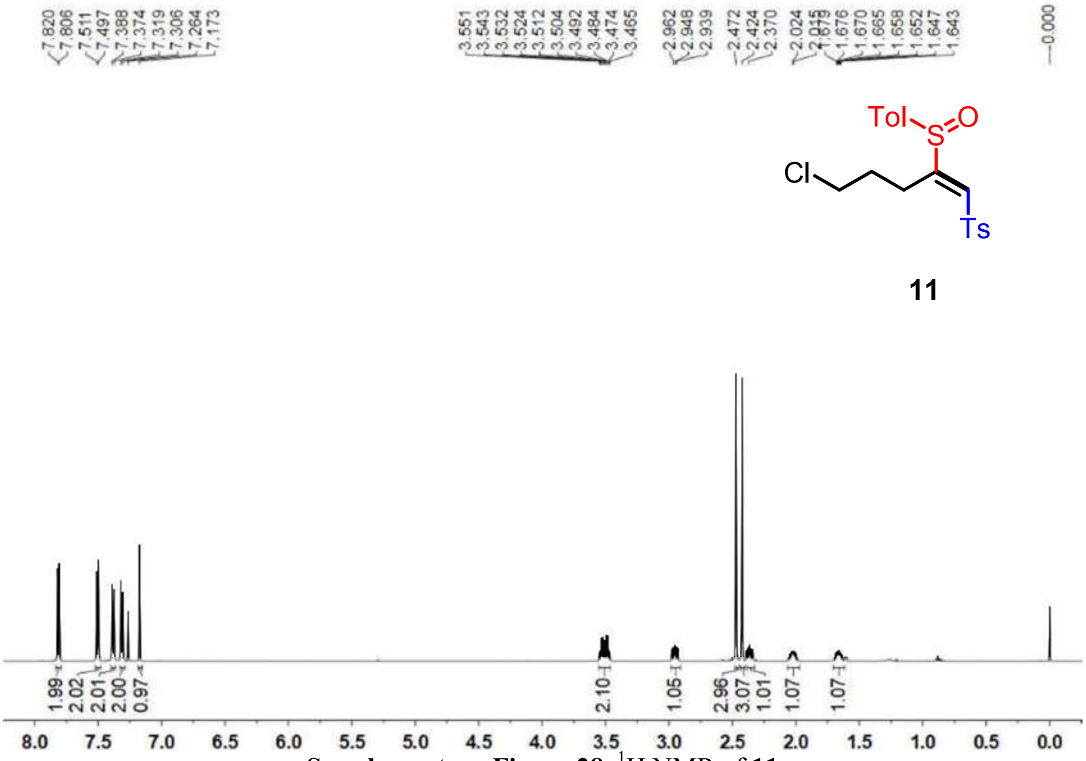

Supplementary Figure 28. ^{1}H NMR of **11**

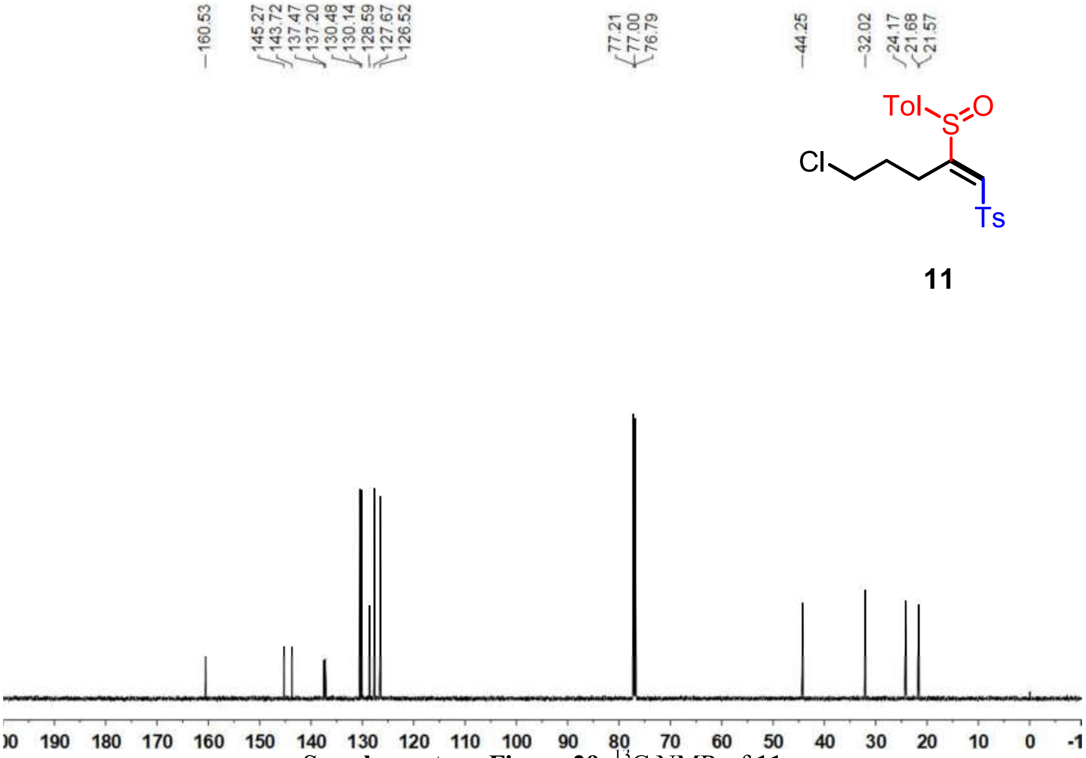

Supplementary Figure 29. ^{13}C NMR of **11**

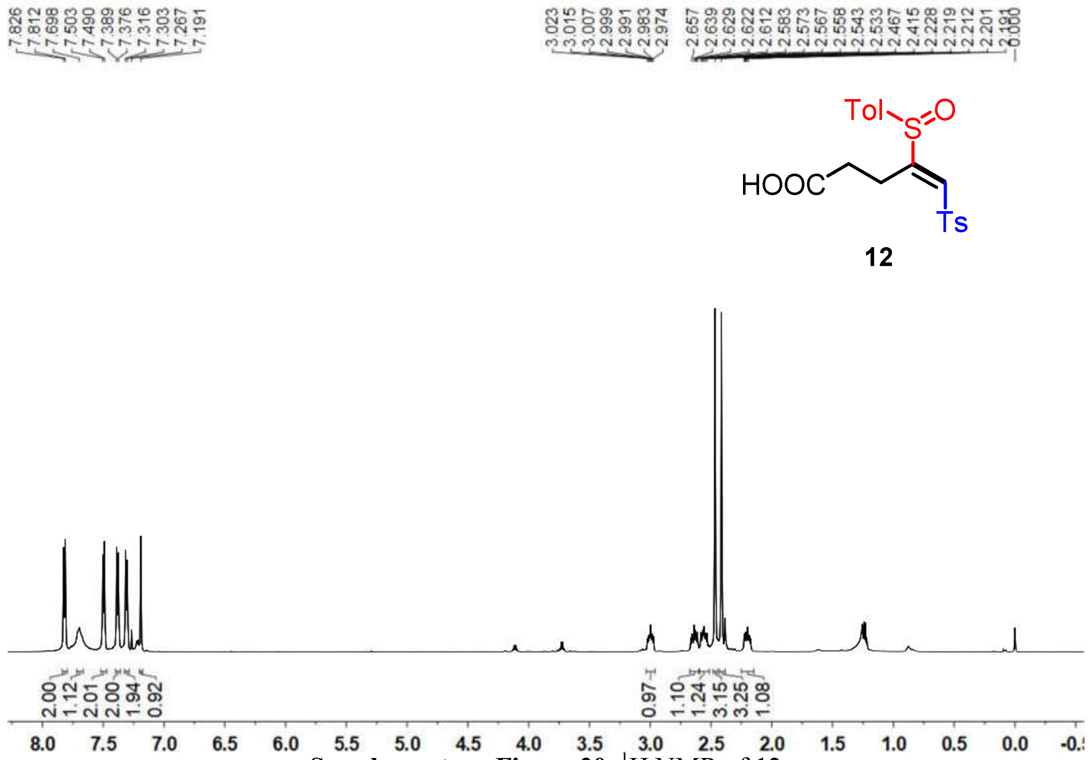

Supplementary Figure 30. ^{1}H NMR of **12**

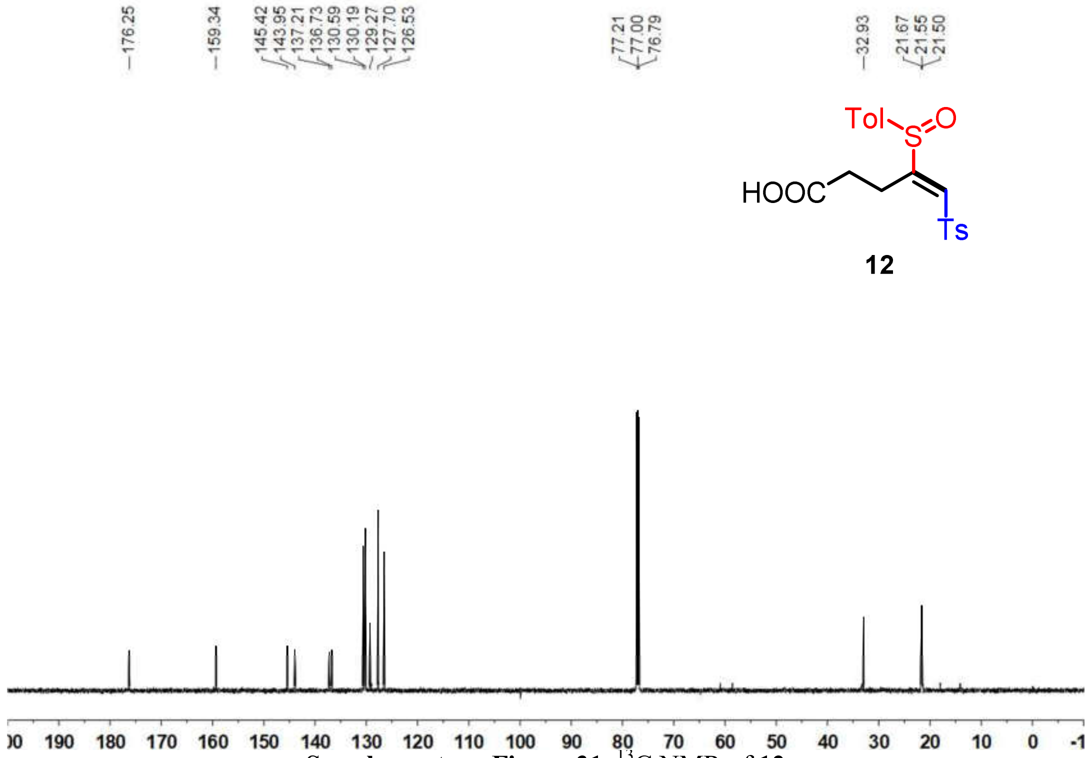

Supplementary Figure 31. ^{13}C NMR of **12**

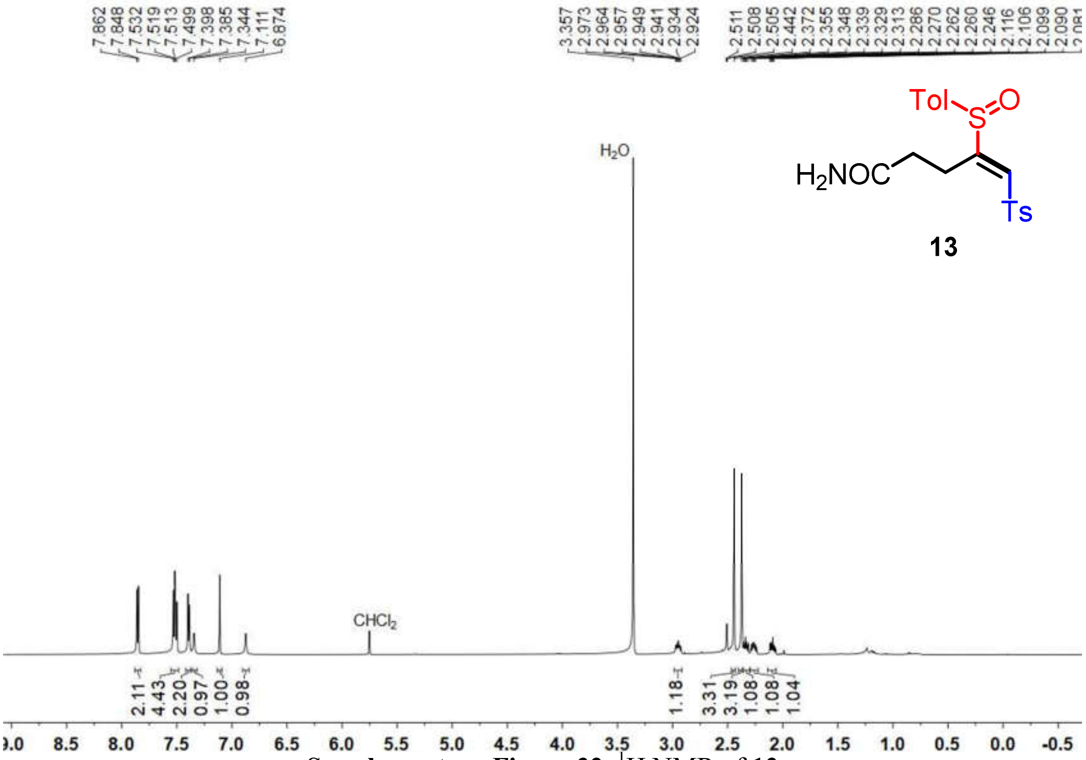

Supplementary Figure 32. ^{1}H NMR of **13**

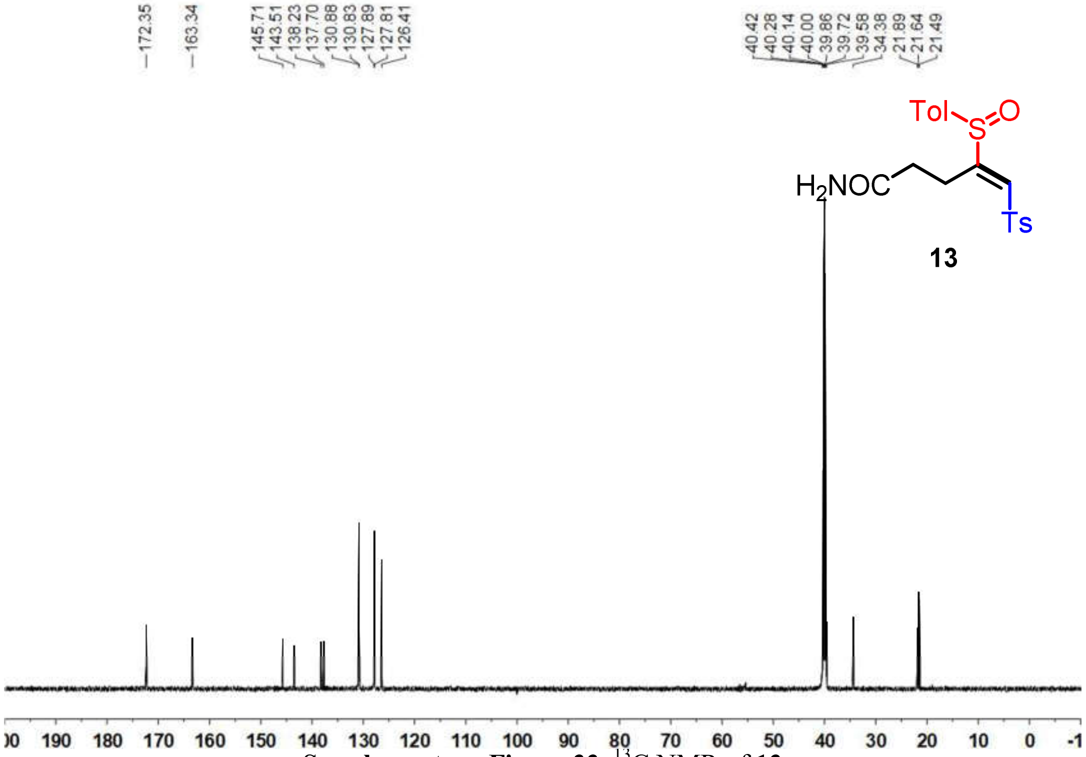

Supplementary Figure 33. ^{13}C NMR of **13**

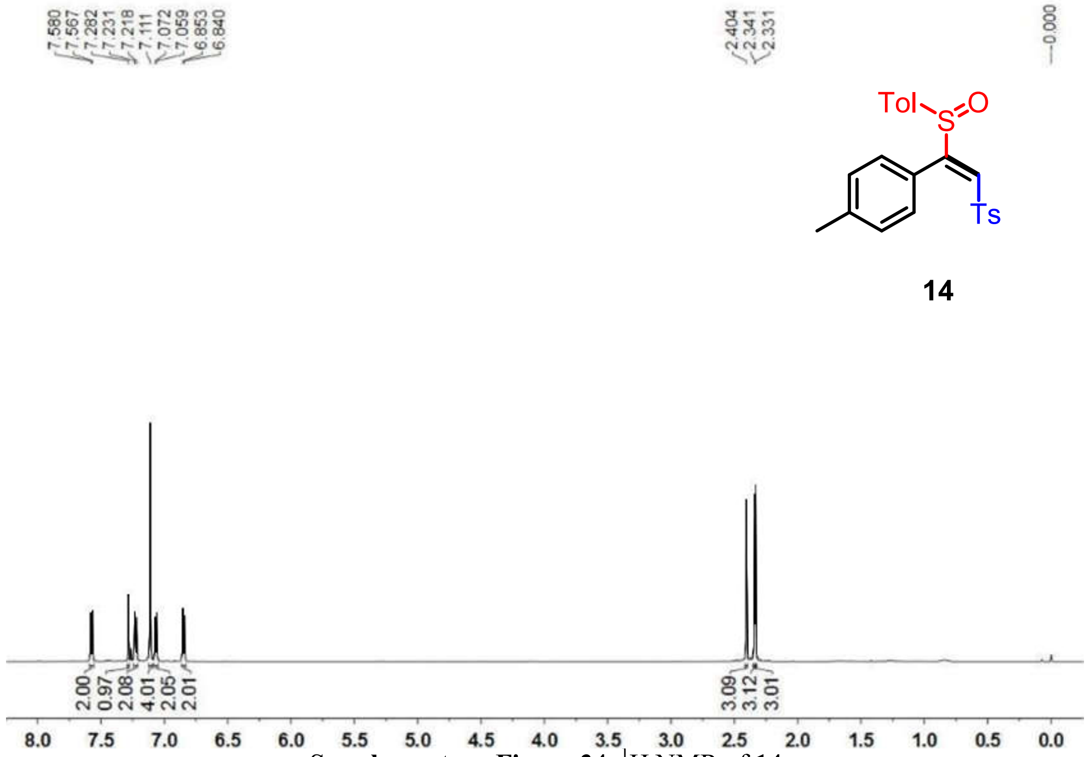

Supplementary Figure 34. ^{1}H NMR of **14**

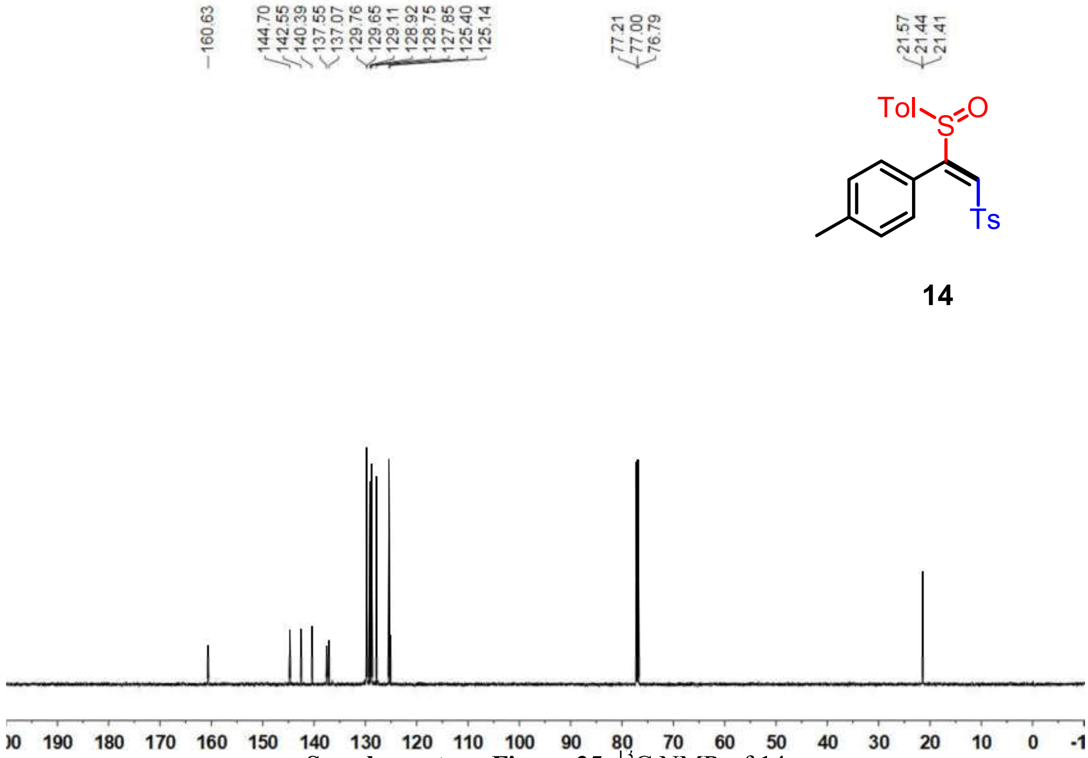

Supplementary Figure 35. ^{13}C NMR of **14**

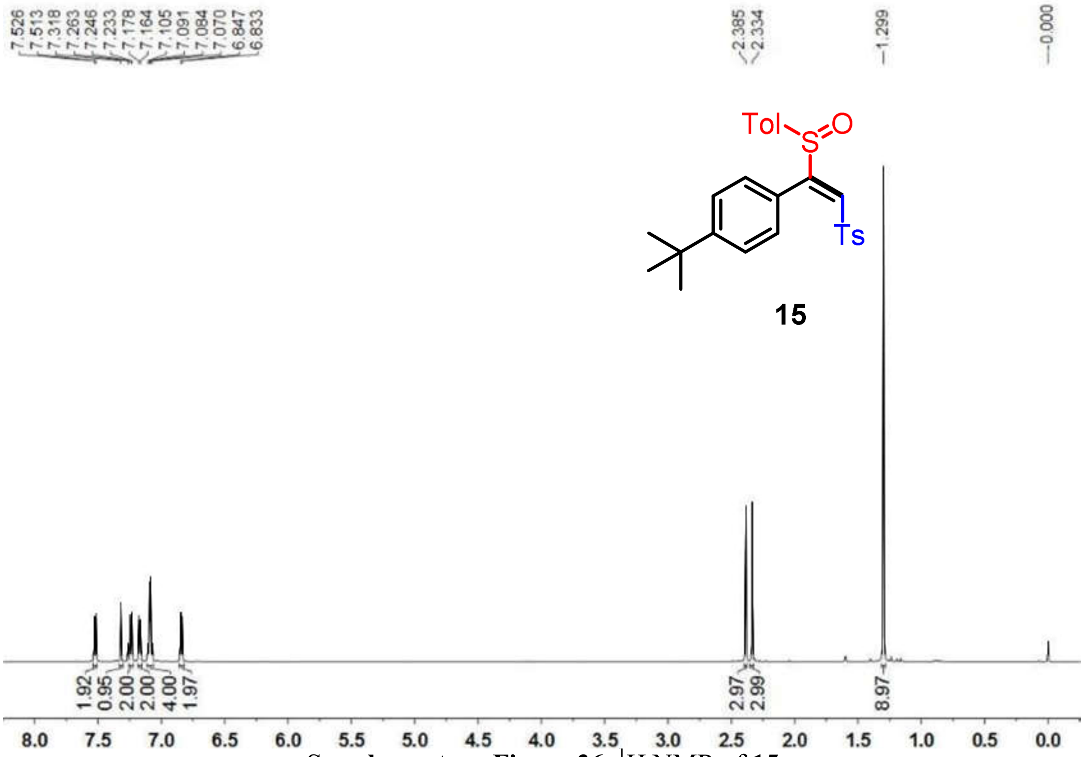

Supplementary Figure 36. ^{1}H NMR of **15**

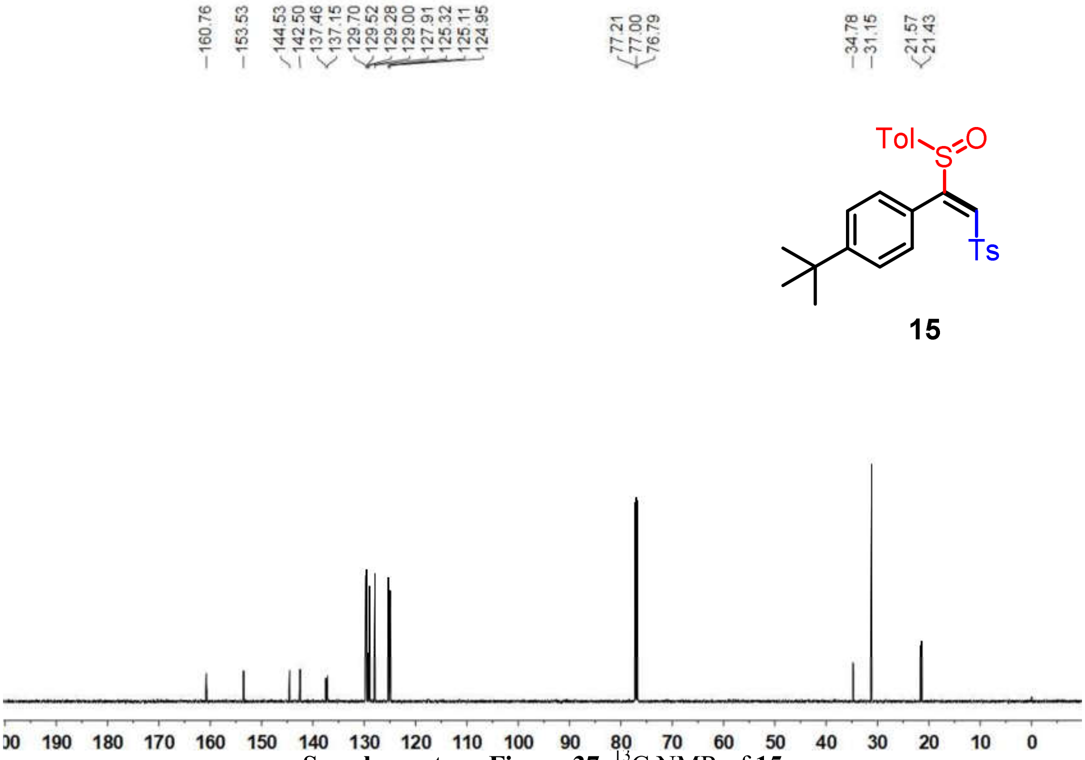

Supplementary Figure 37. ^{13}C NMR of **15**

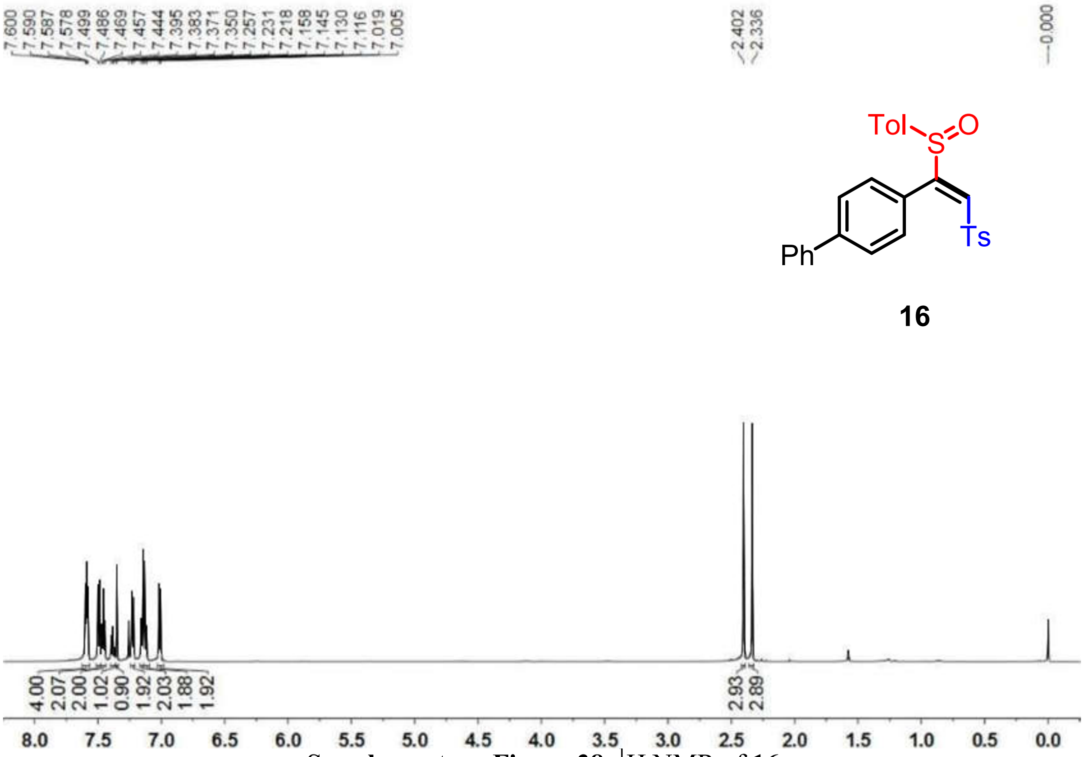

Supplementary Figure 38. ^{1}H NMR of **16**

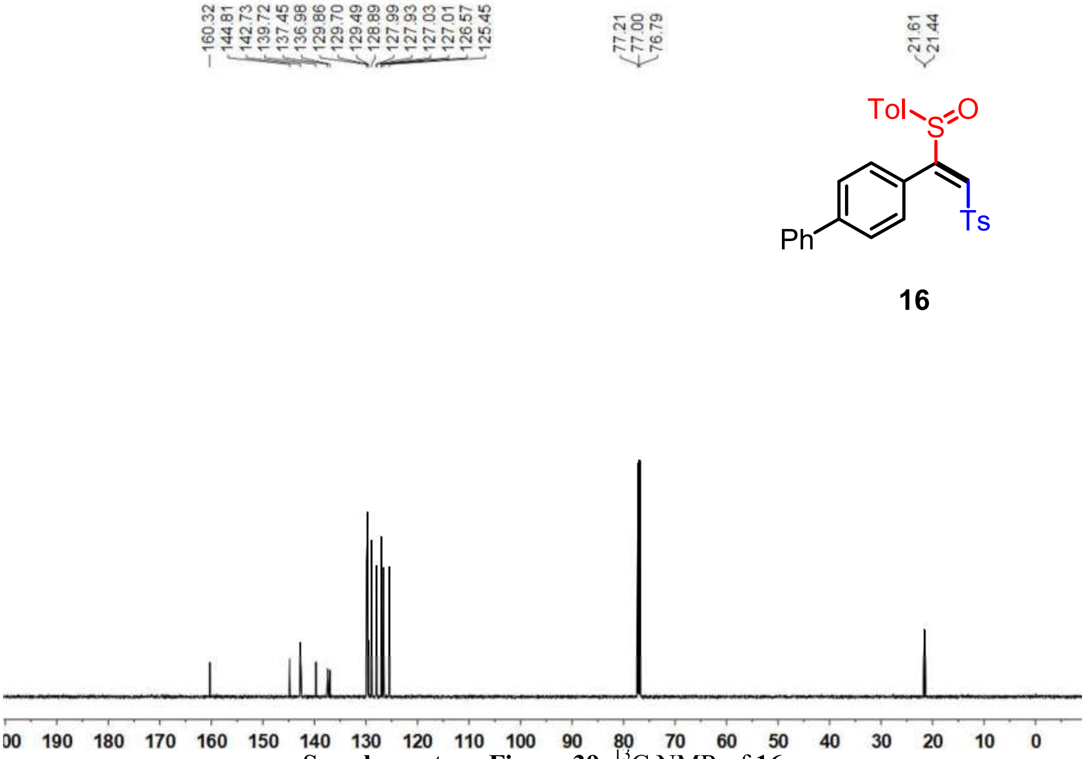

Supplementary Figure 39. ^{13}C NMR of **16**

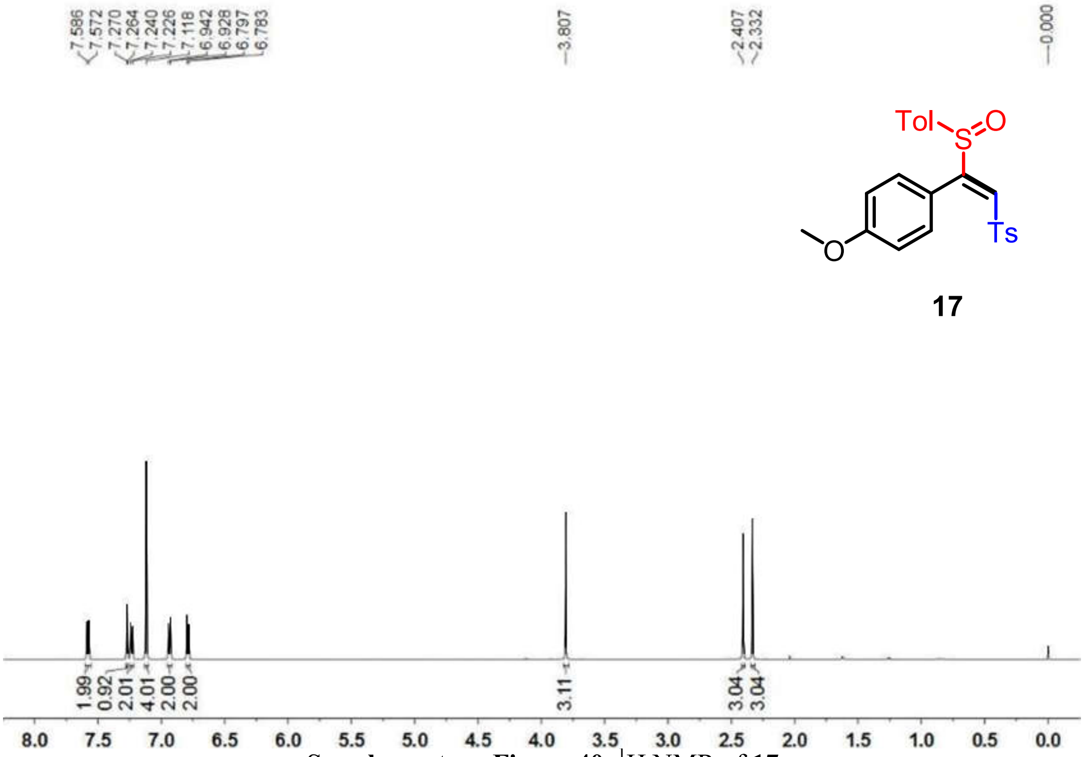

17

Supplementary Figure 40. ^{1}H NMR of **17**

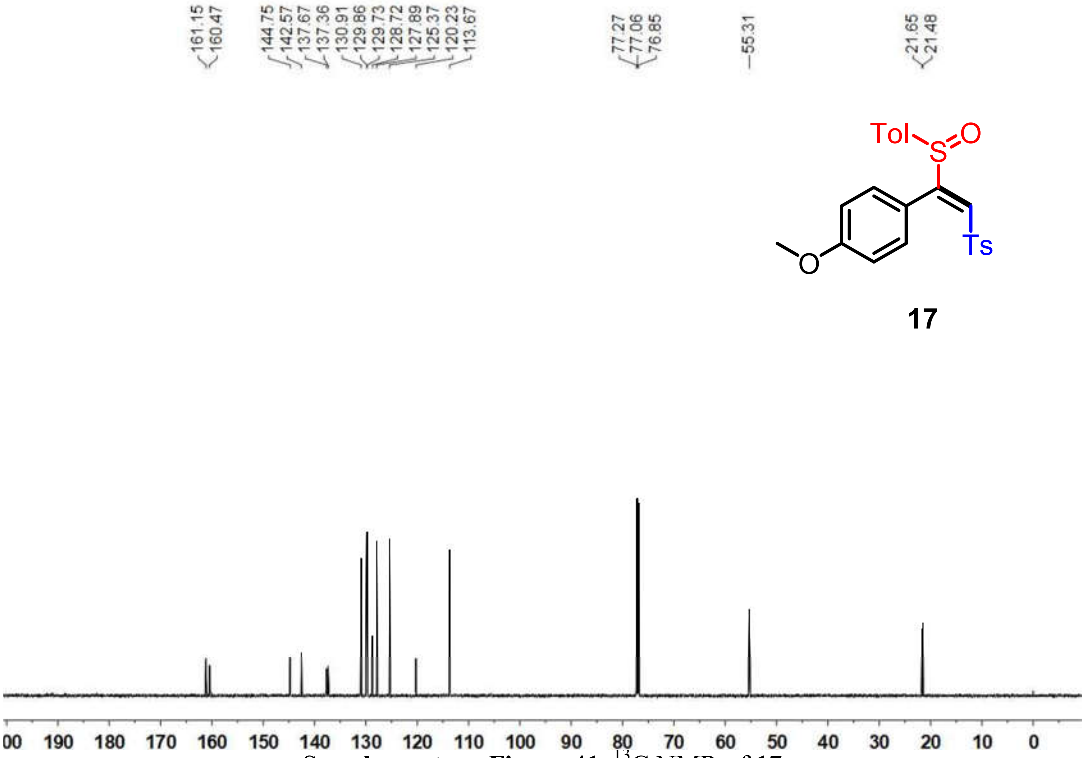

17

Supplementary Figure 41. ^{13}C NMR of **17**

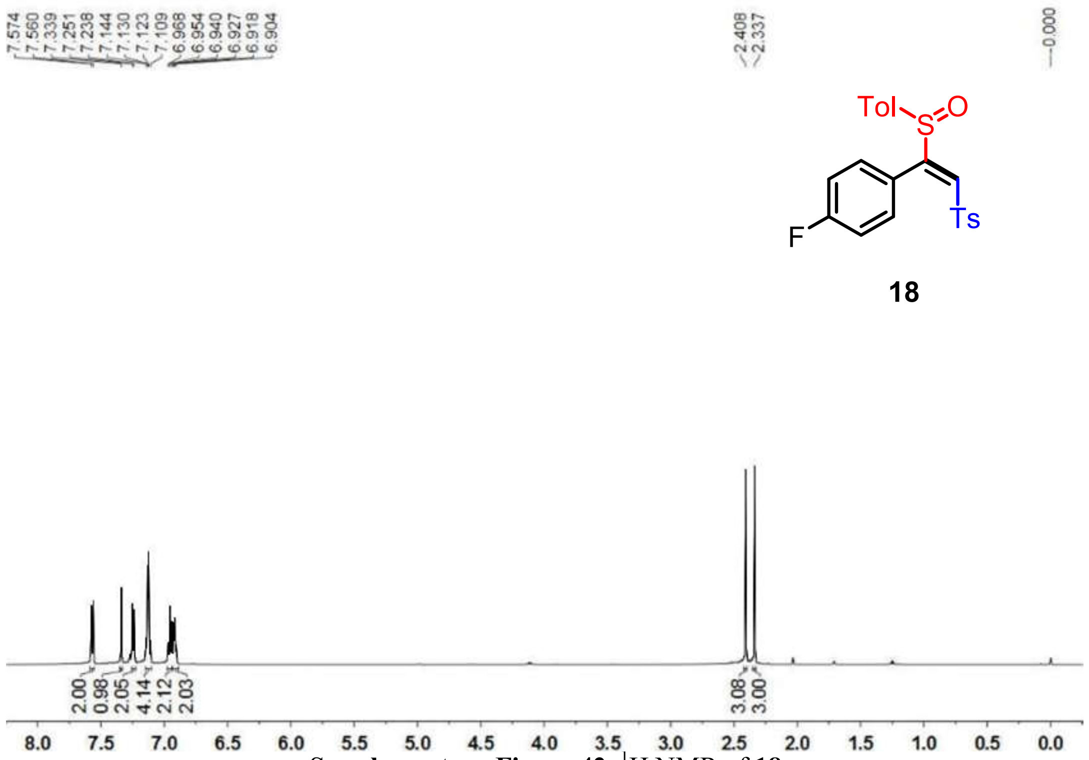

Supplementary Figure 42. ^{1}H NMR of **18**

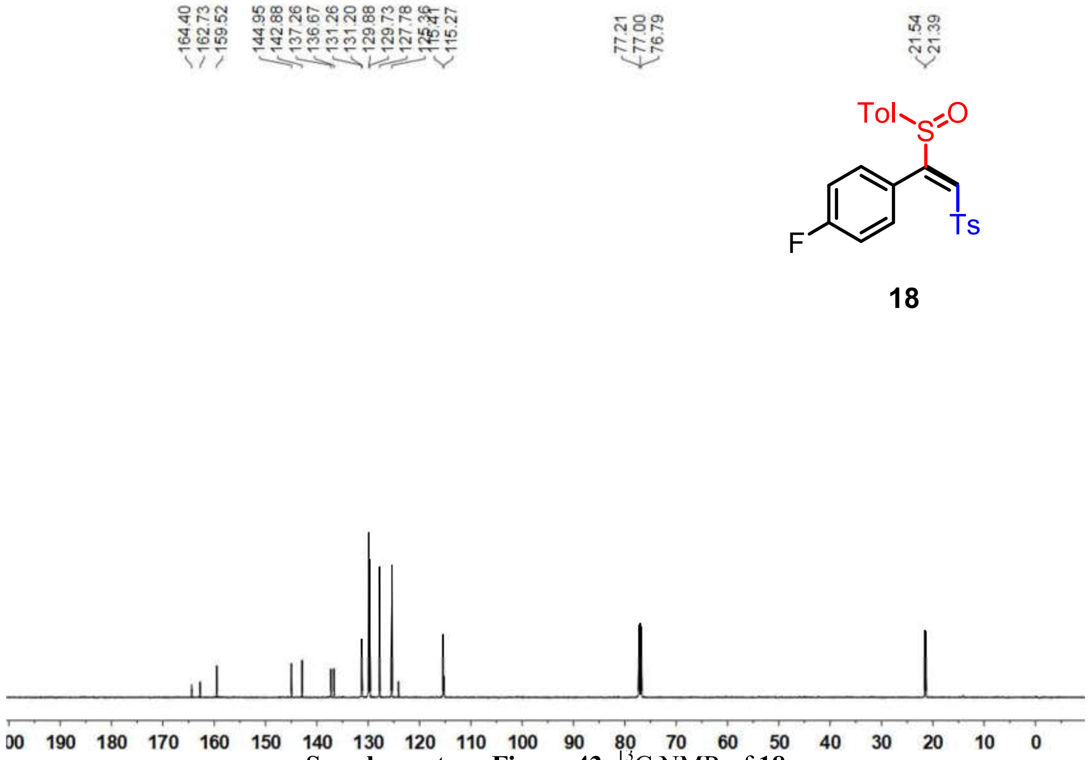

Supplementary Figure 43. ^{13}C NMR of **18**

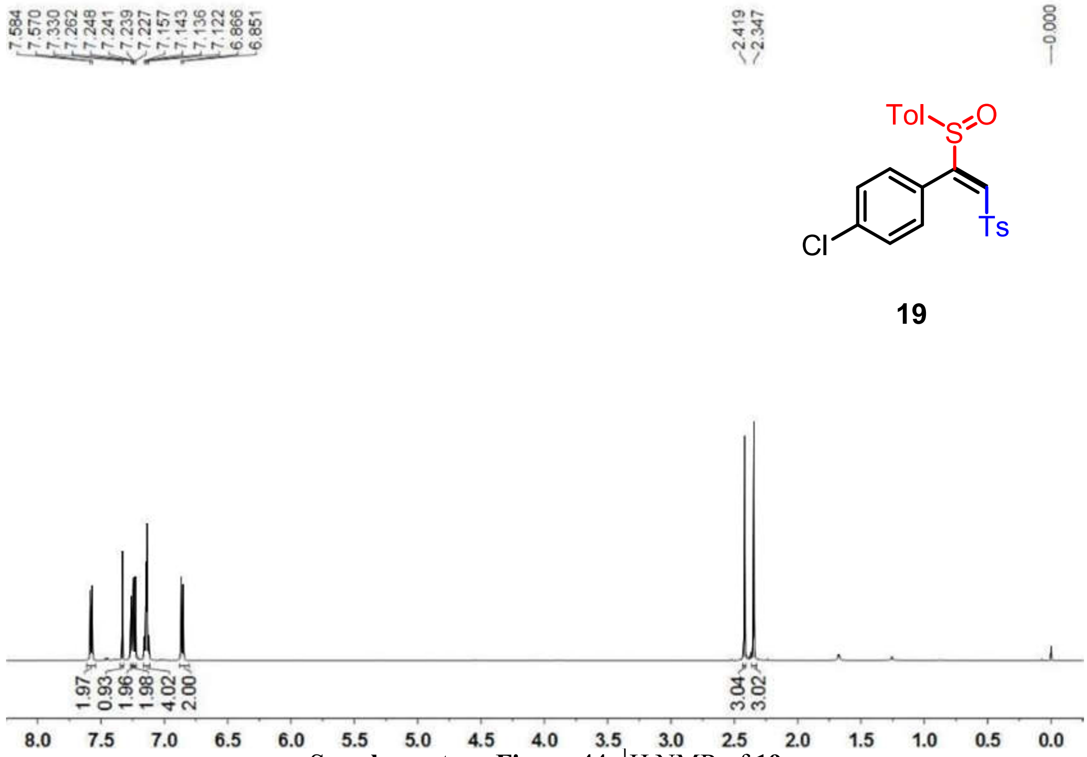

Supplementary Figure 44. ^{1}H NMR of **19**

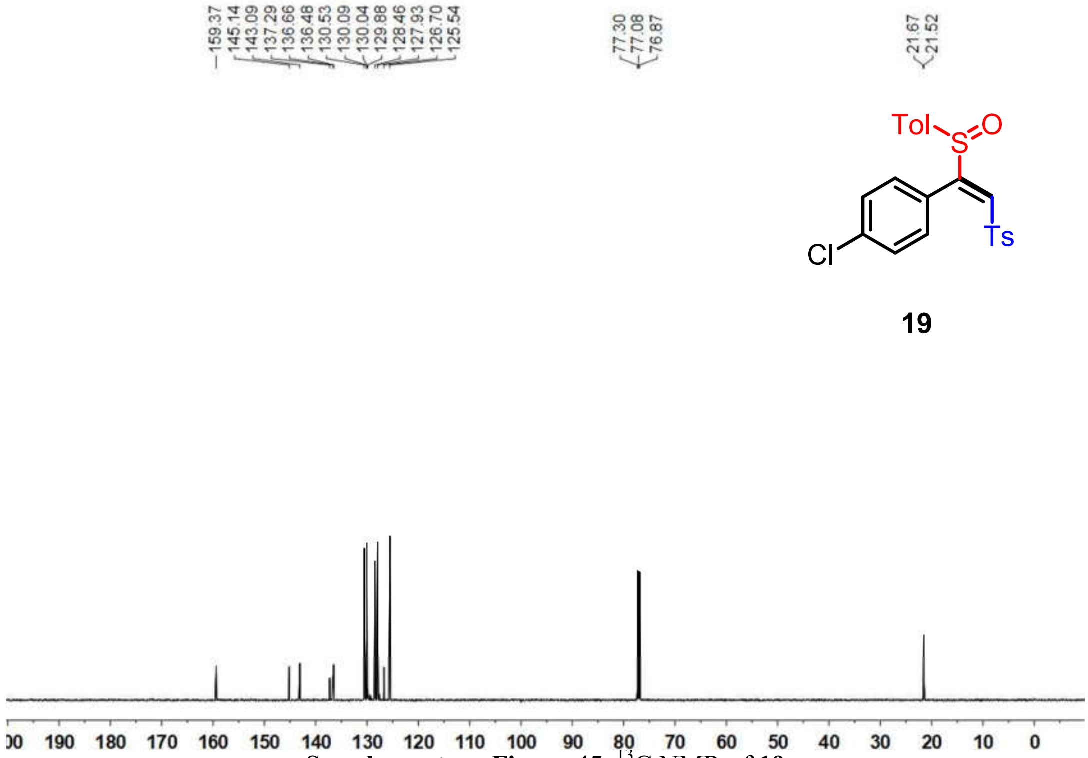

Supplementary Figure 45. ^{13}C NMR of **19**

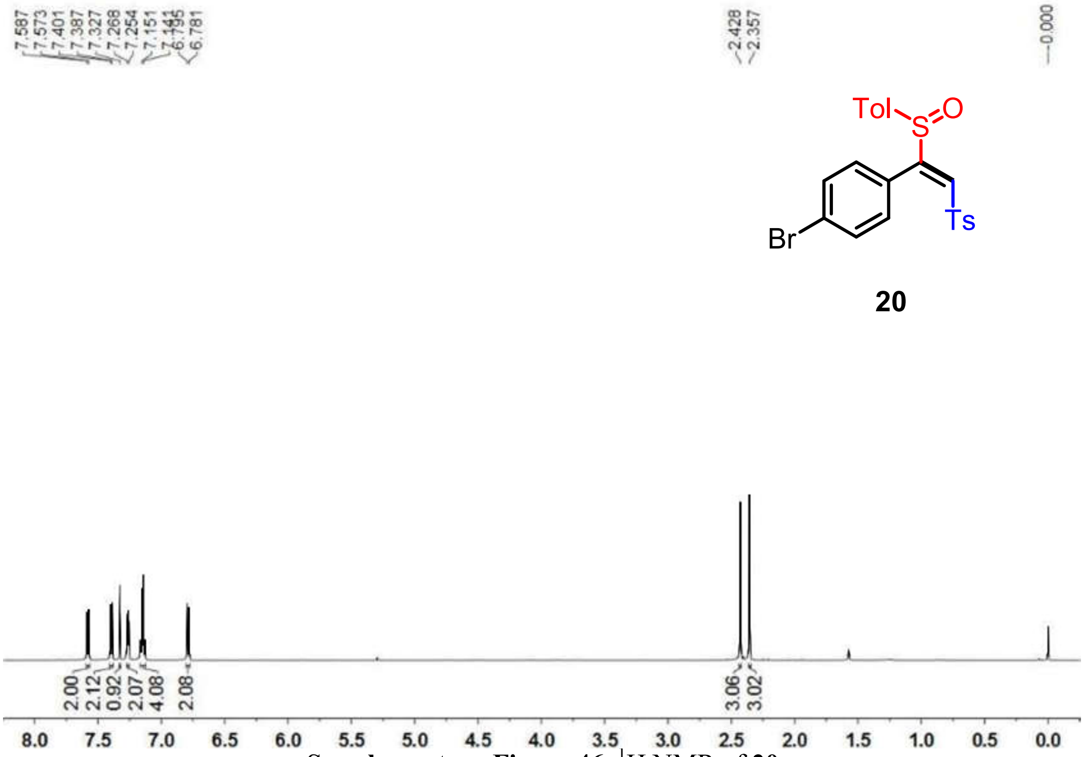

Supplementary Figure 46. ^{1}H NMR of **20**

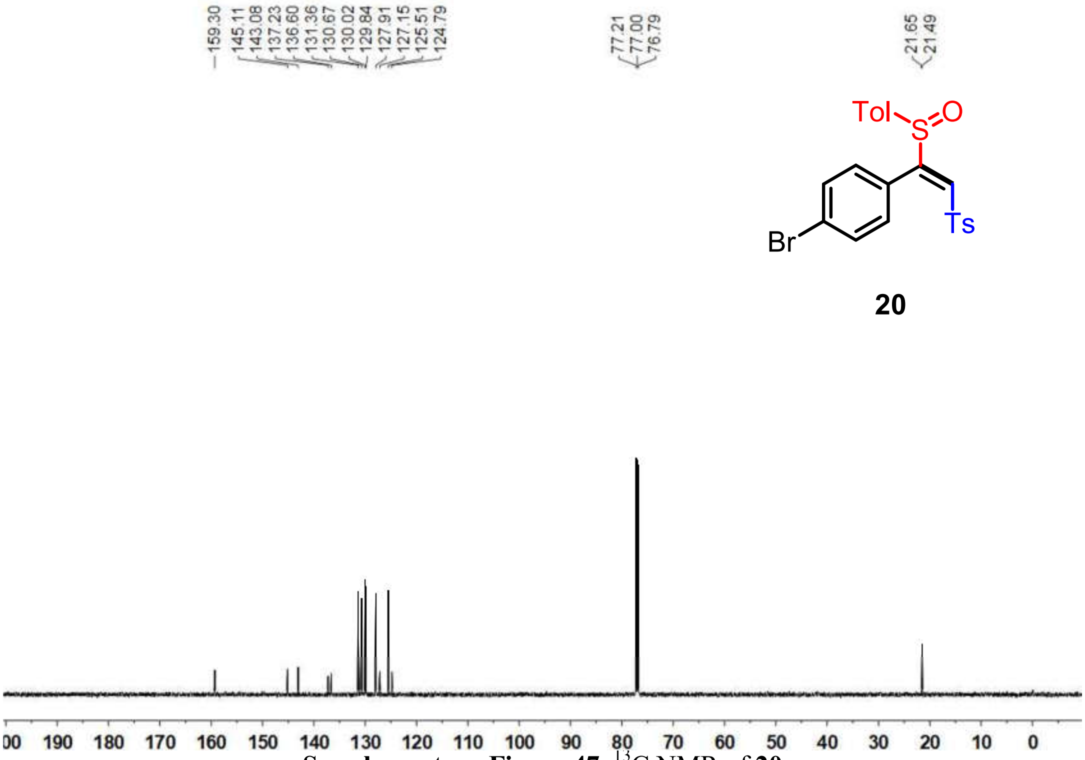

Supplementary Figure 47. ^{13}C NMR of **20**

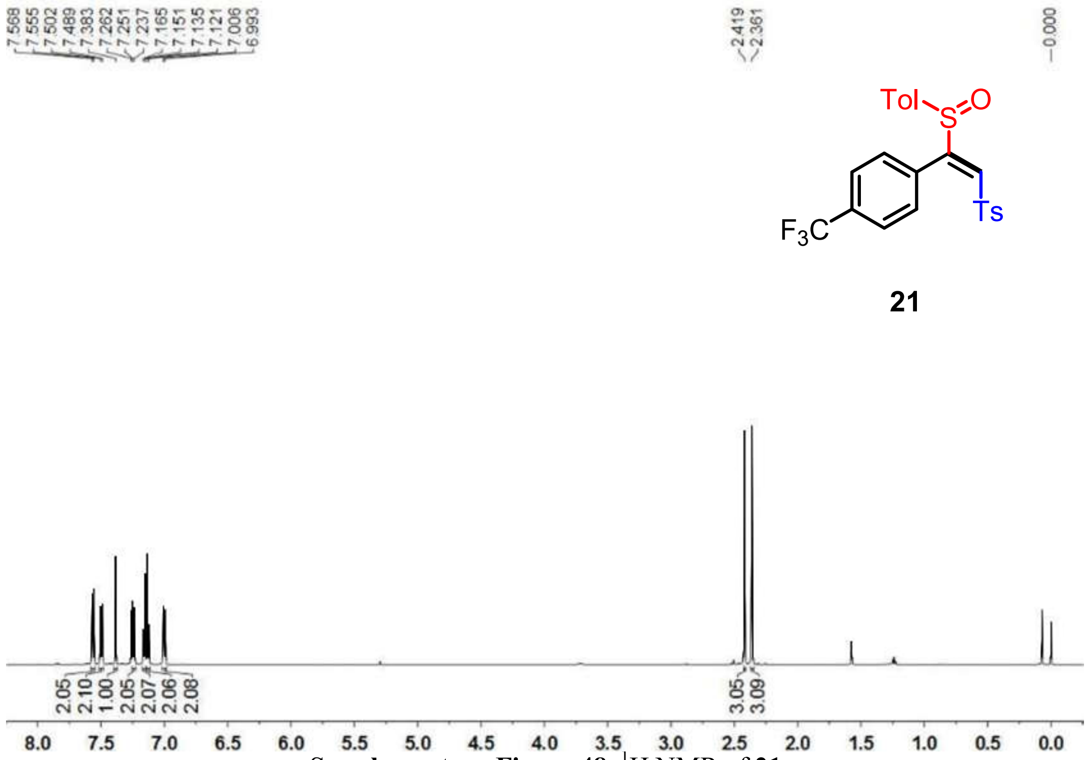

Supplementary Figure 48. ^{1}H NMR of **21**

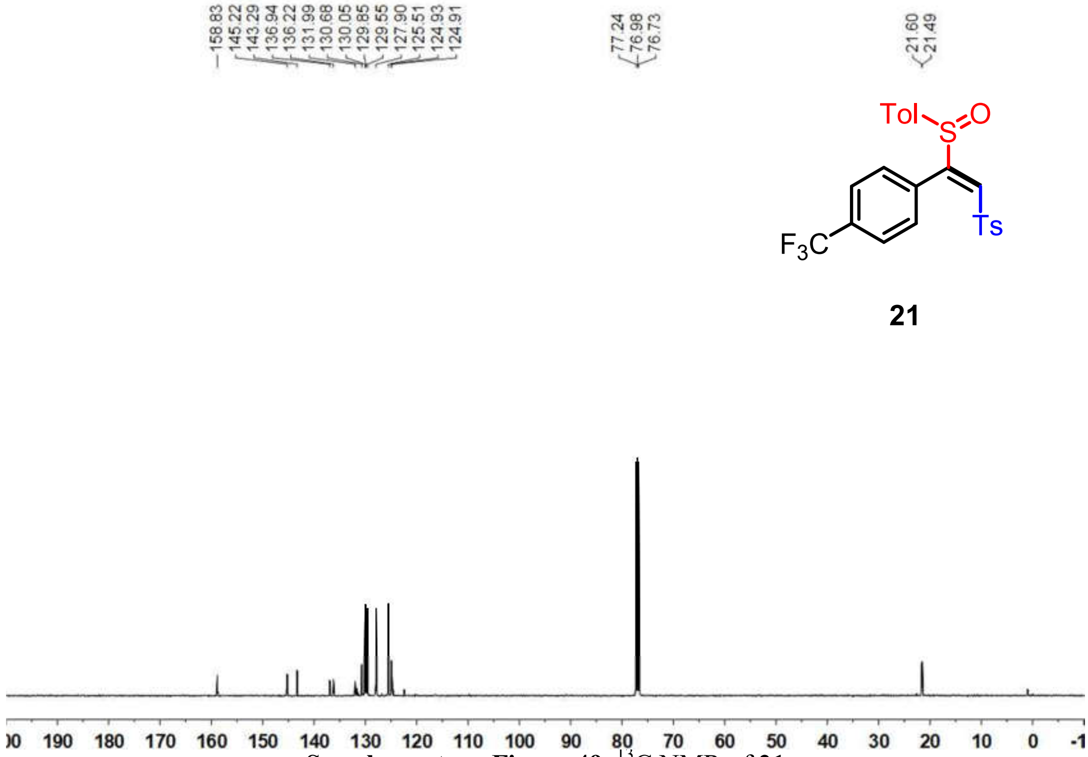

Supplementary Figure 49. ^{13}C NMR of **21**

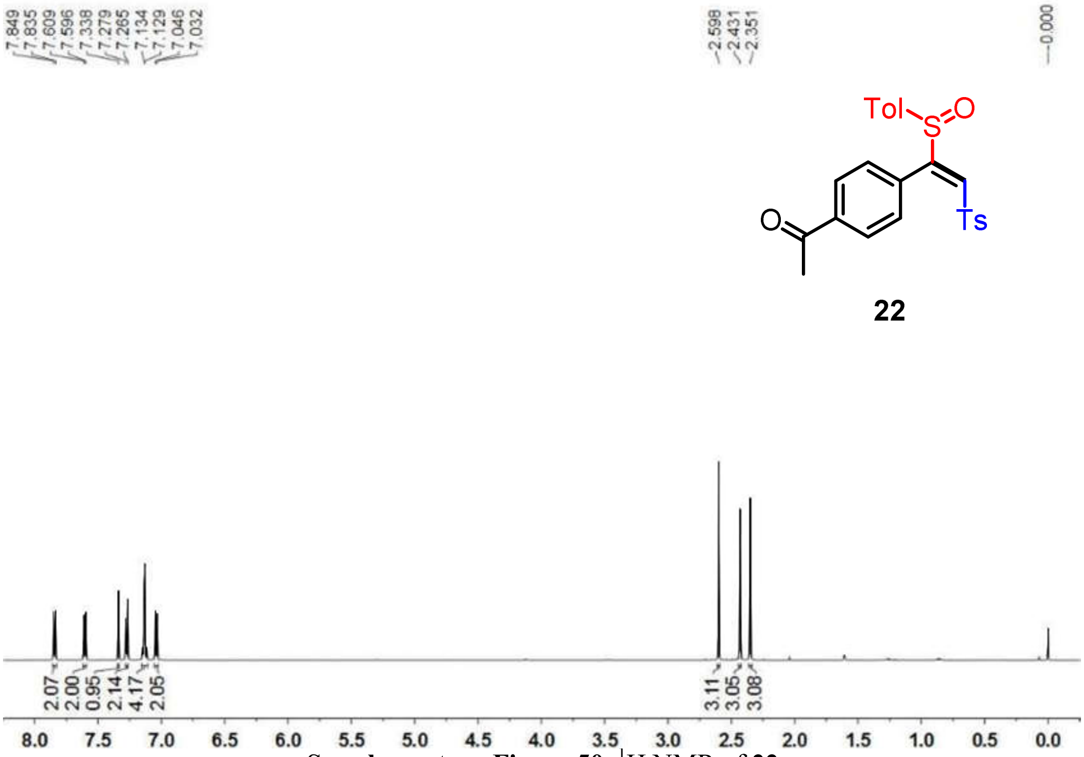

Supplementary Figure 50. ^{1}H NMR of **22**

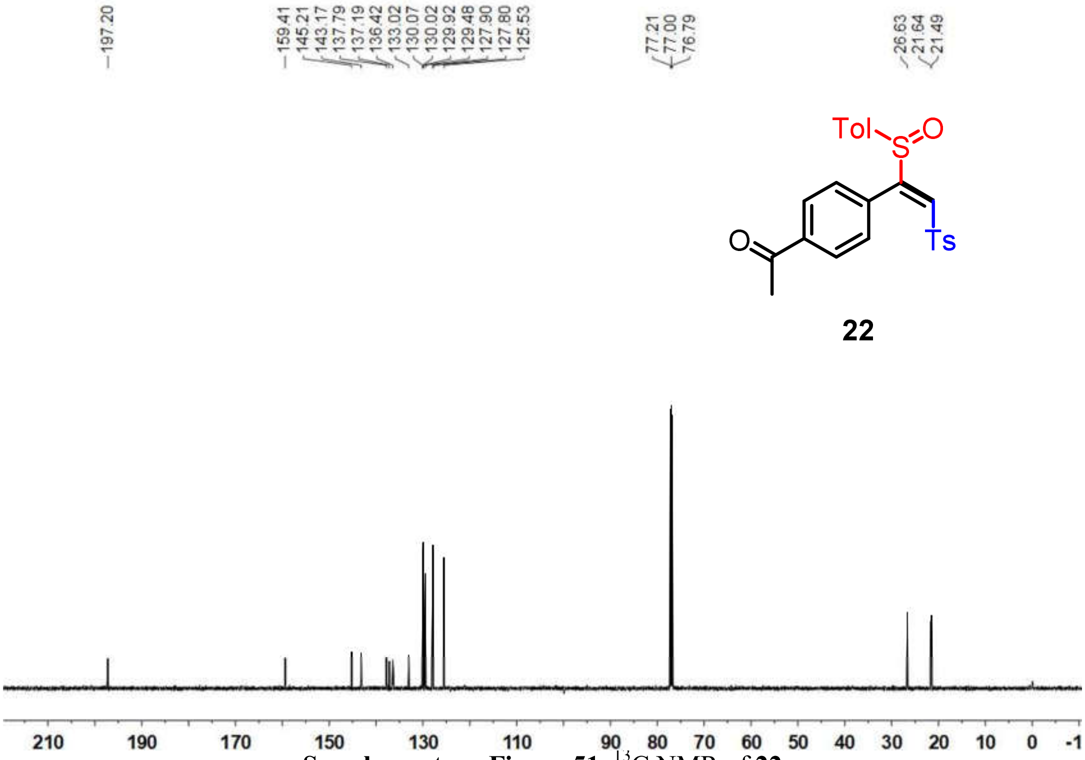

Supplementary Figure 51. ^{13}C NMR of **22**

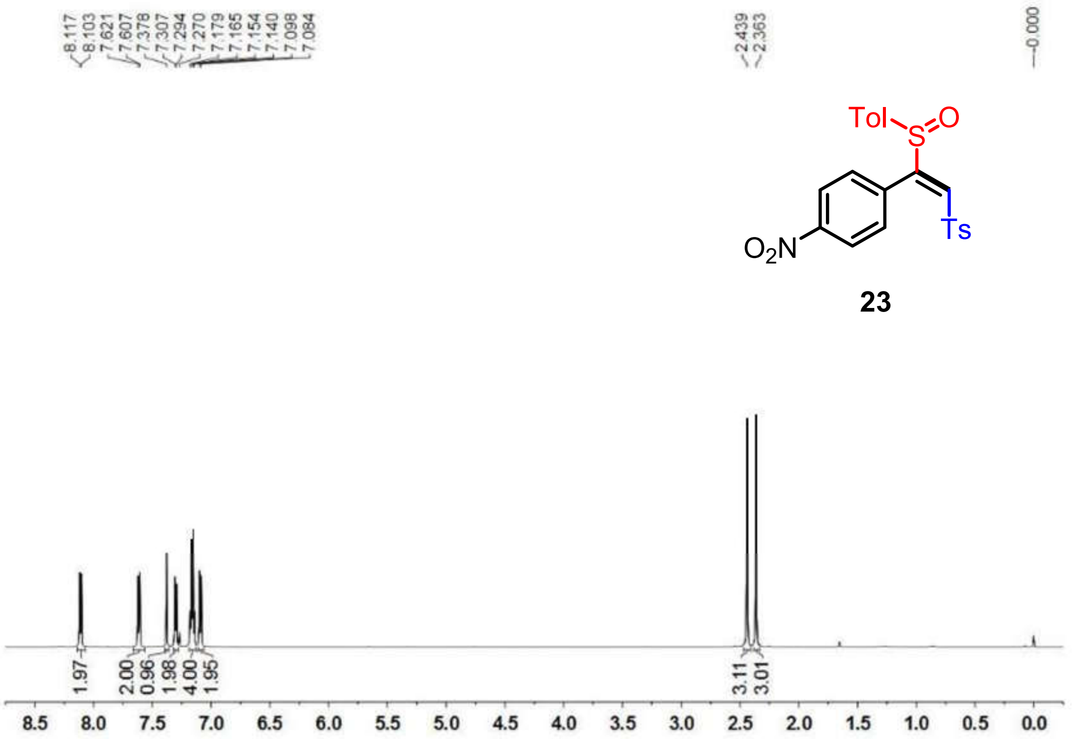

Supplementary Figure 52. ^{1}H NMR of **23**

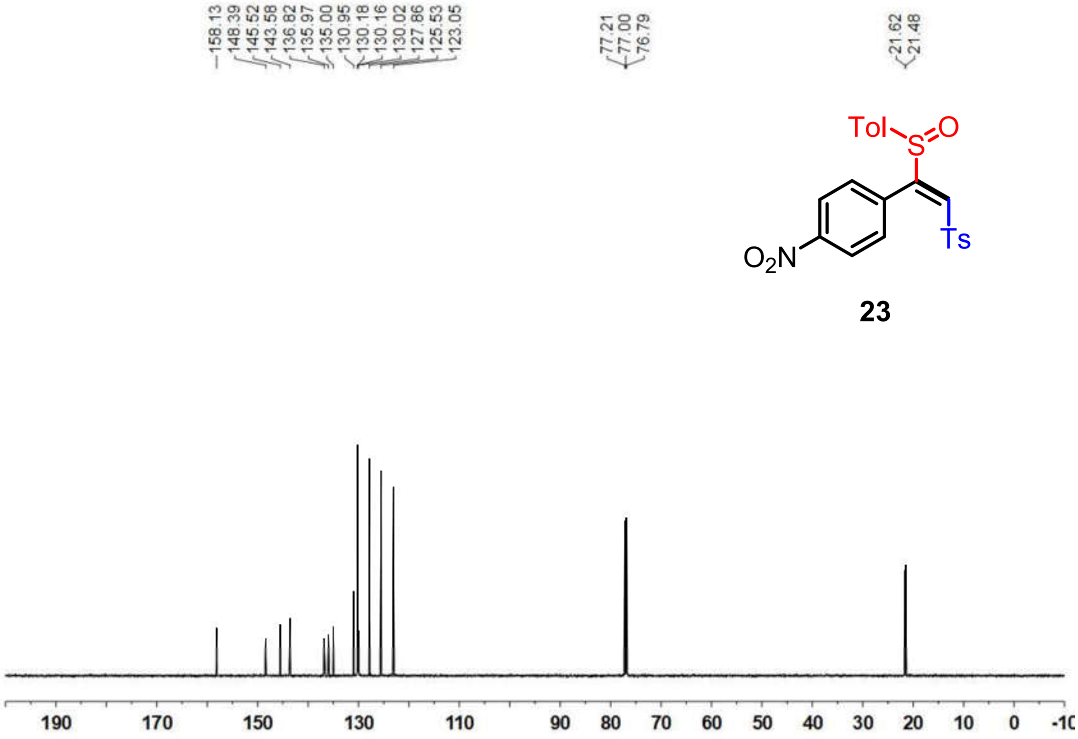

Supplementary Figure 53. ^{13}C NMR of **23**

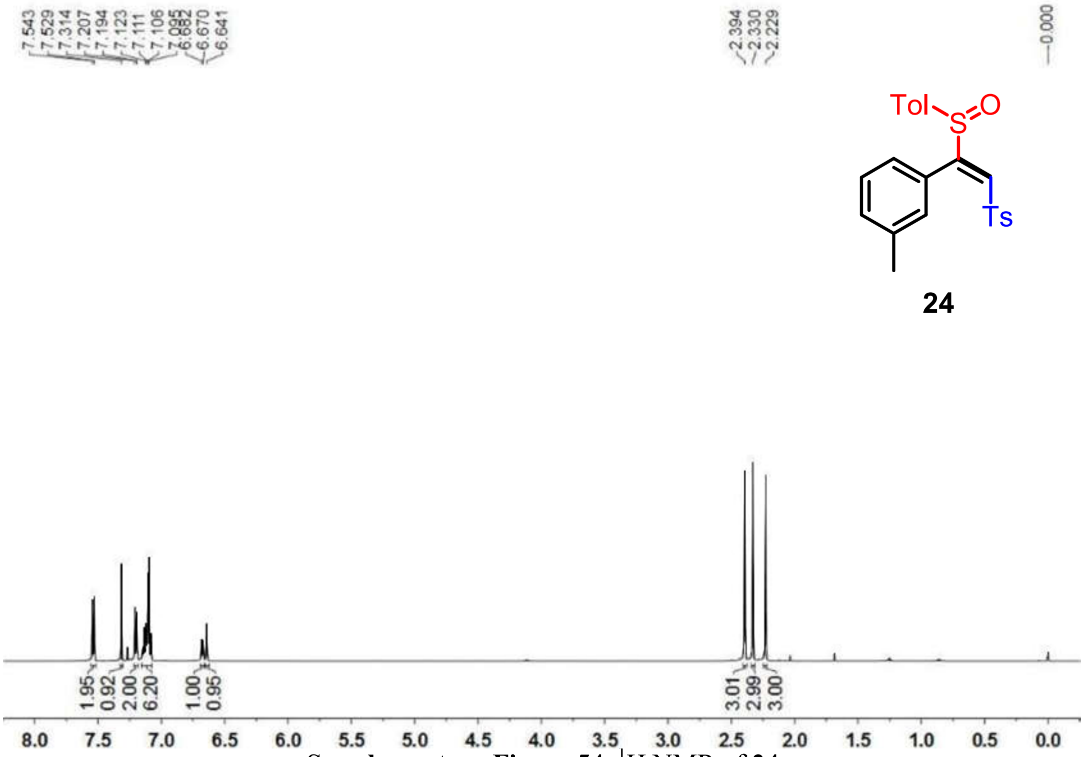

Supplementary Figure 54. ^{1}H NMR of **24**

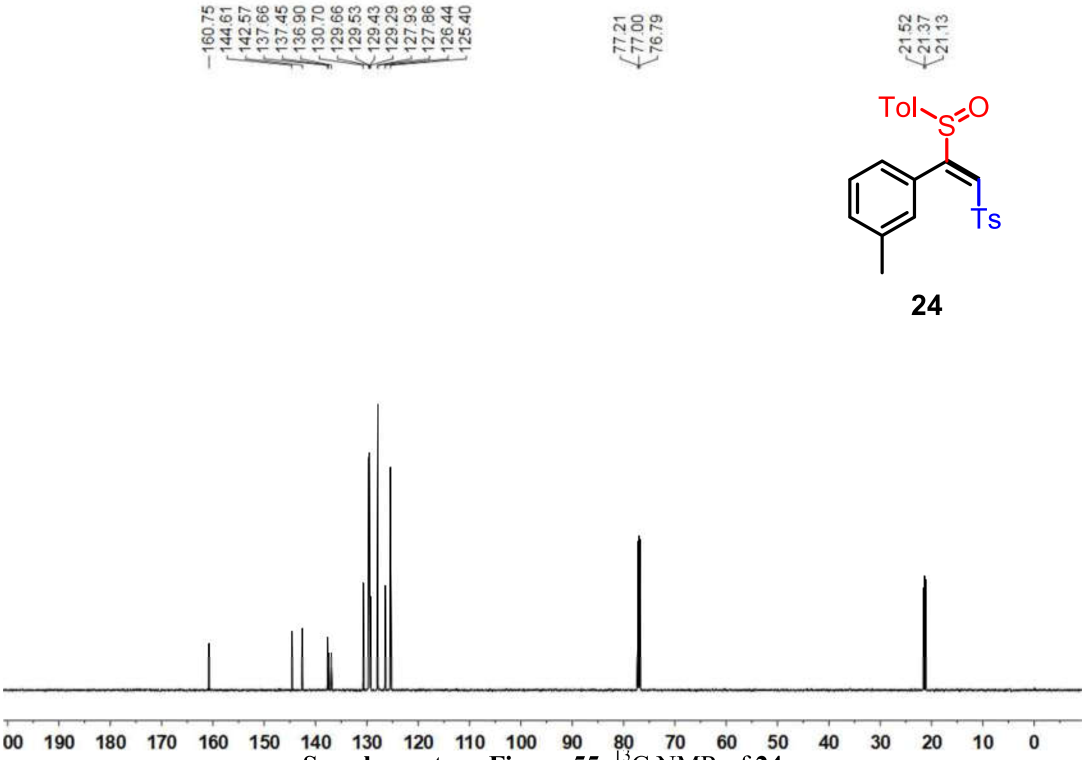

Supplementary Figure 55. ^{13}C NMR of **24**

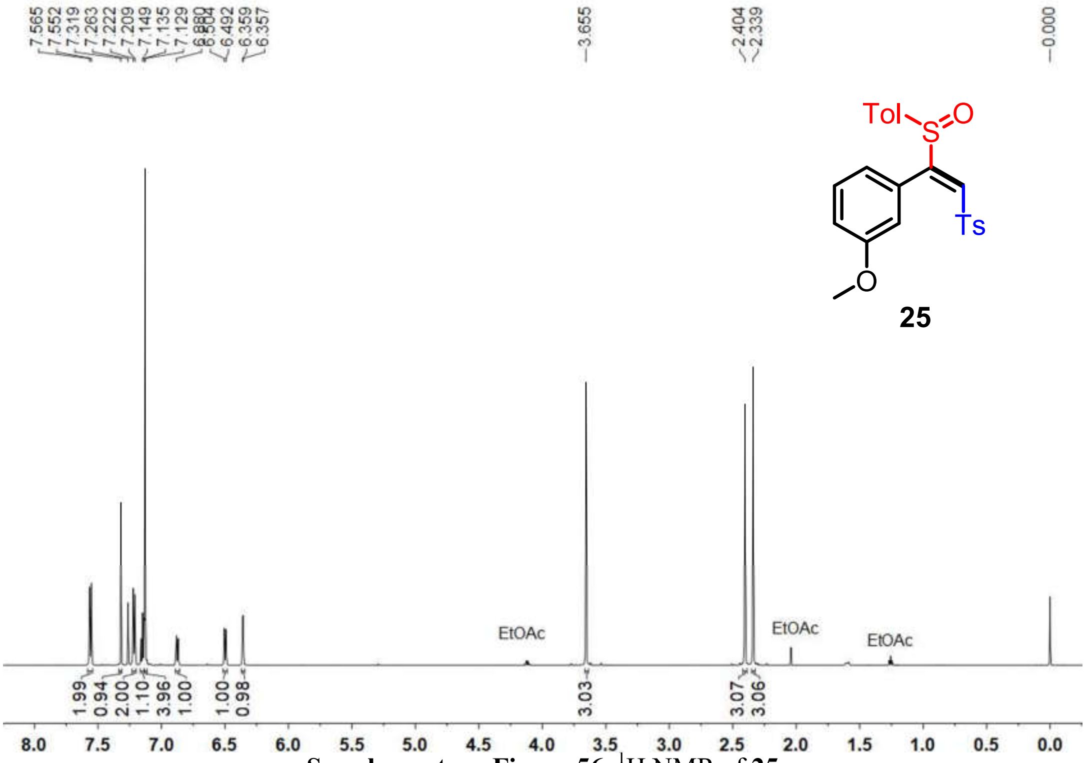

Supplementary Figure 56. ^{1}H NMR of **25**

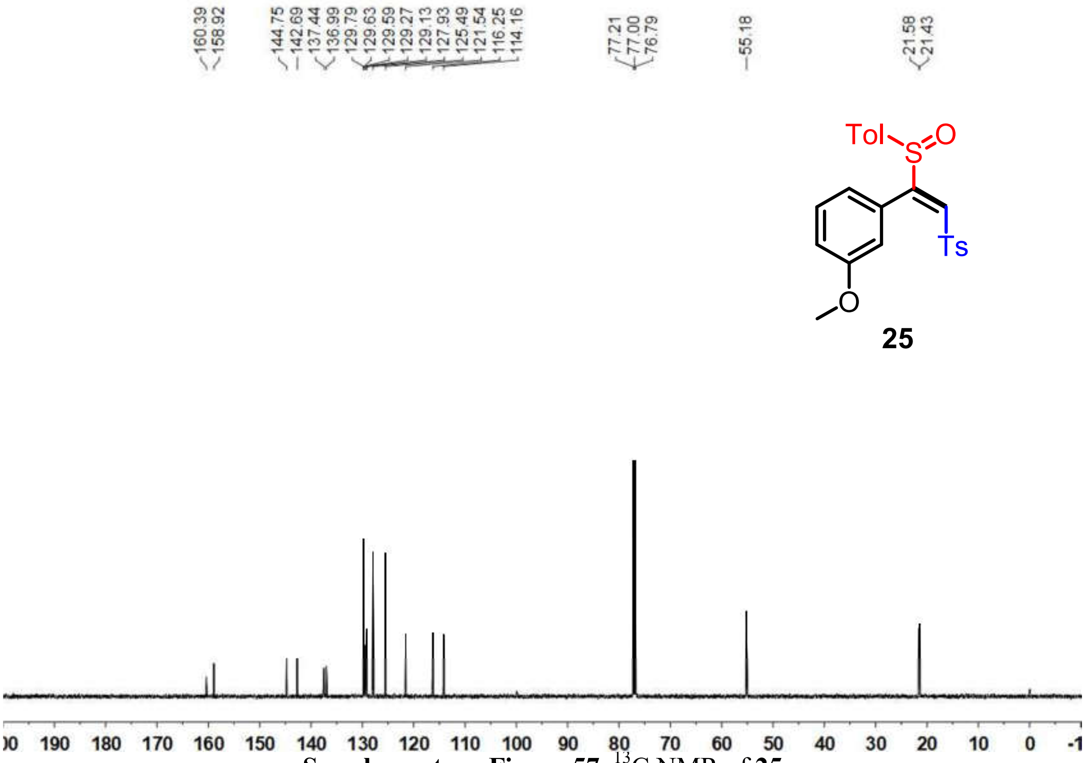

Supplementary Figure 57. ^{13}C NMR of **25**

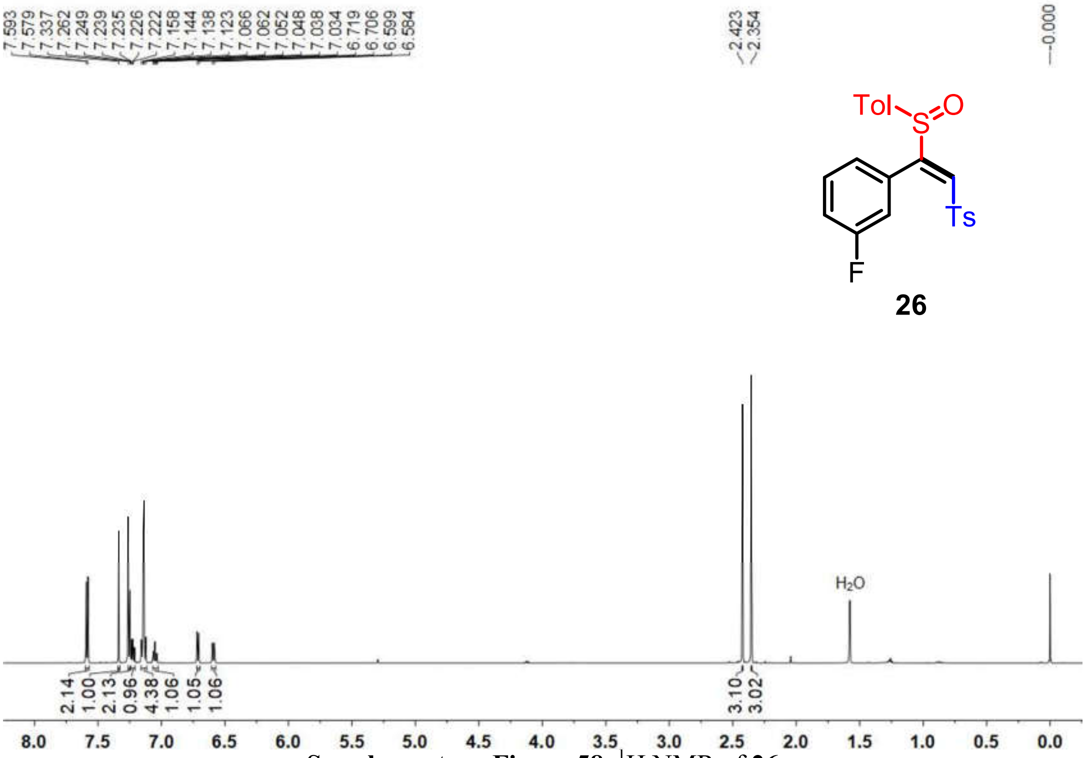

Supplementary Figure 58. ^{1}H NMR of **26**

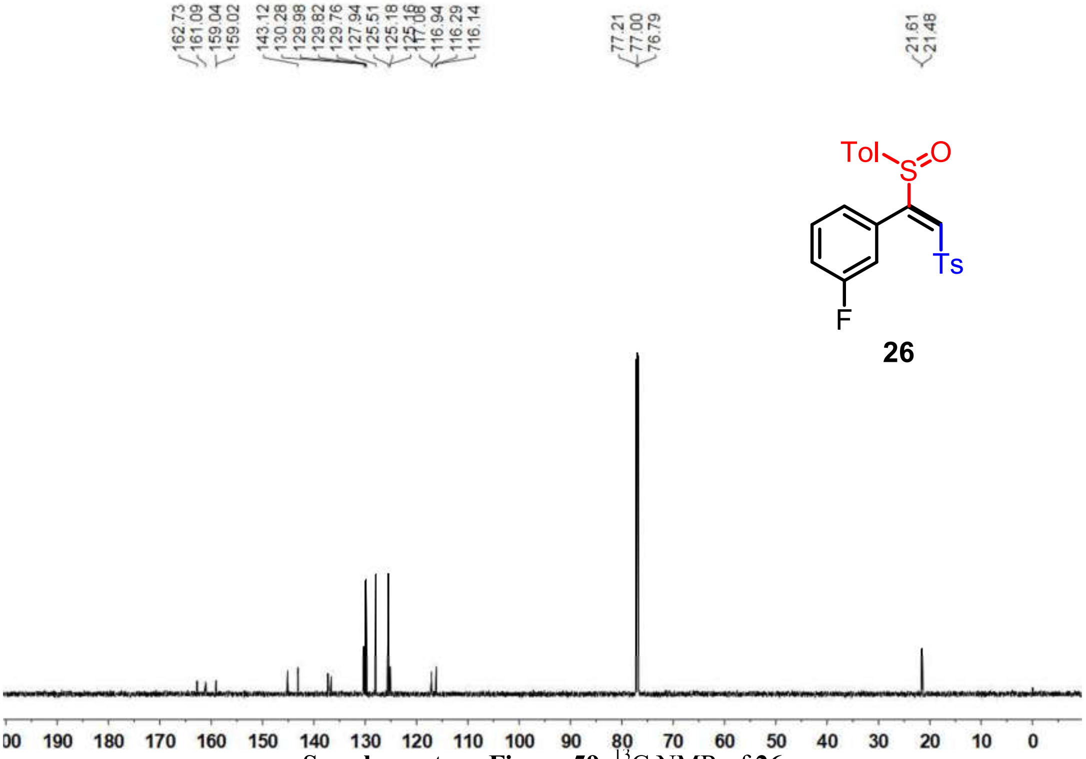

Supplementary Figure 59. ^{13}C NMR of **26**

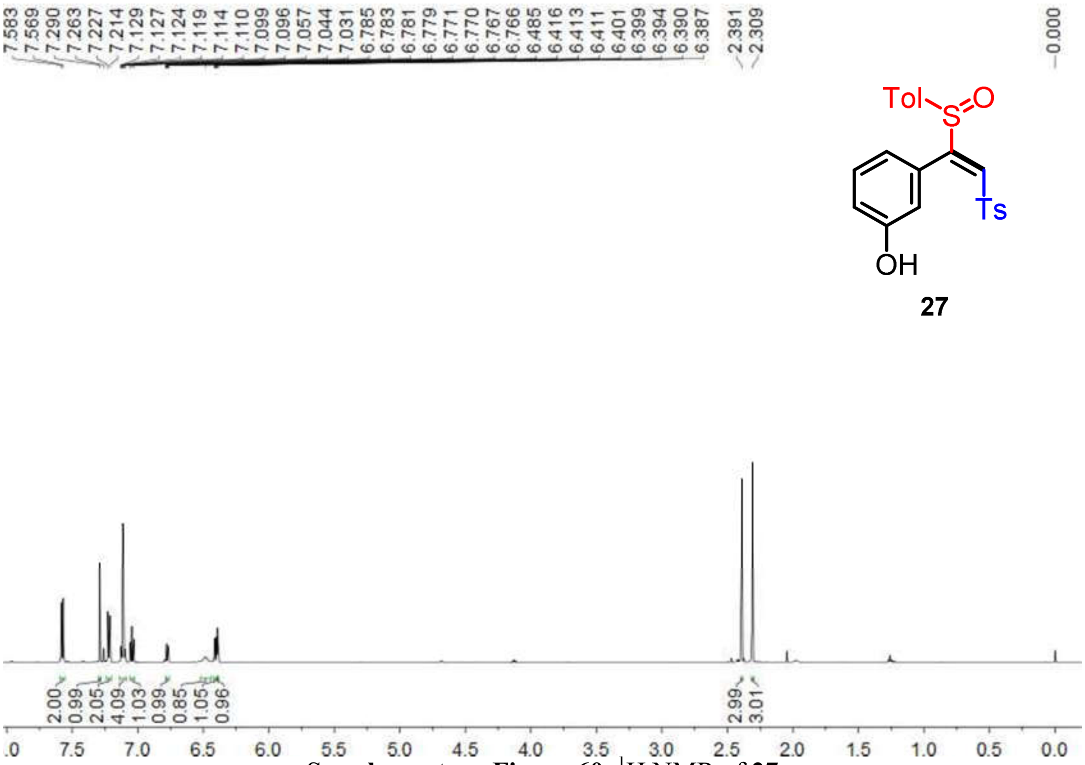

Supplementary Figure 60. ^{1}H NMR of **27**

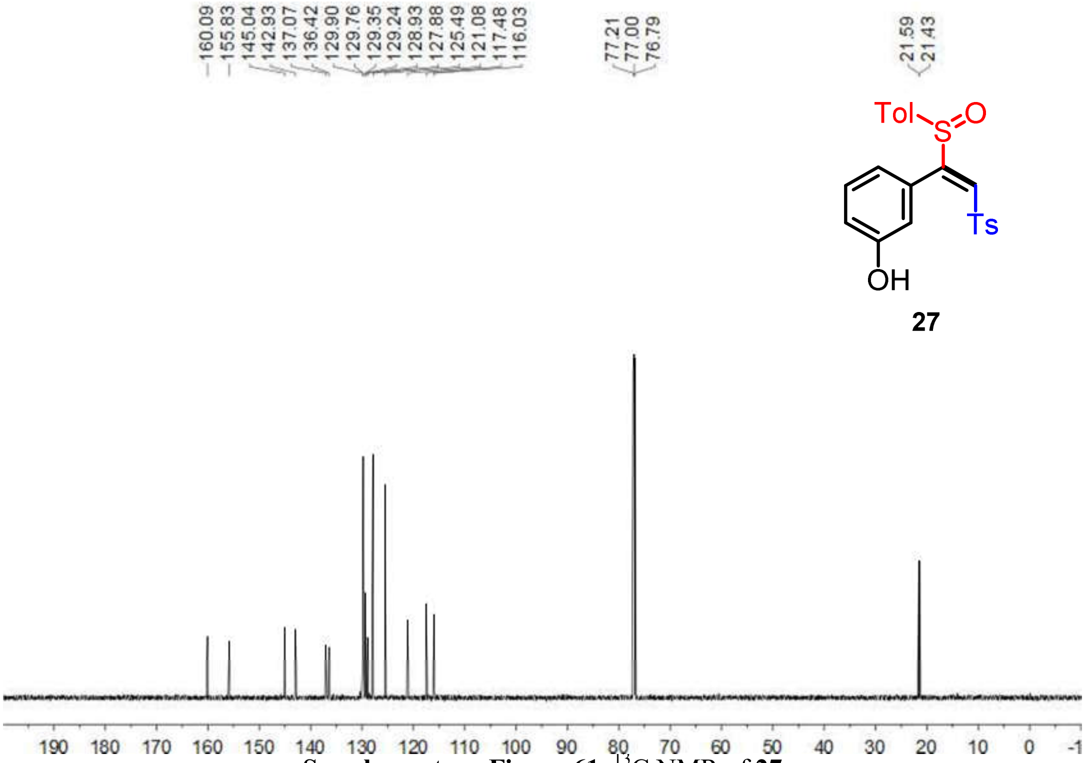

Supplementary Figure 61. ^{13}C NMR of **27**

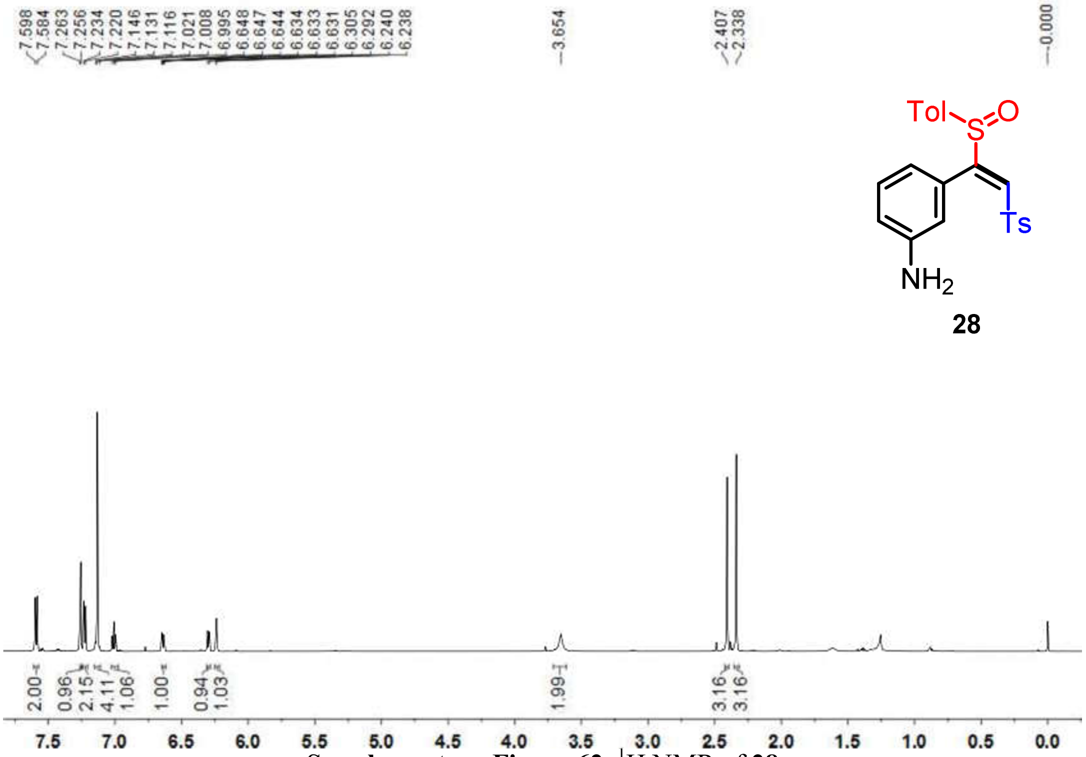

Supplementary Figure 62. ^{1}H NMR of **28**

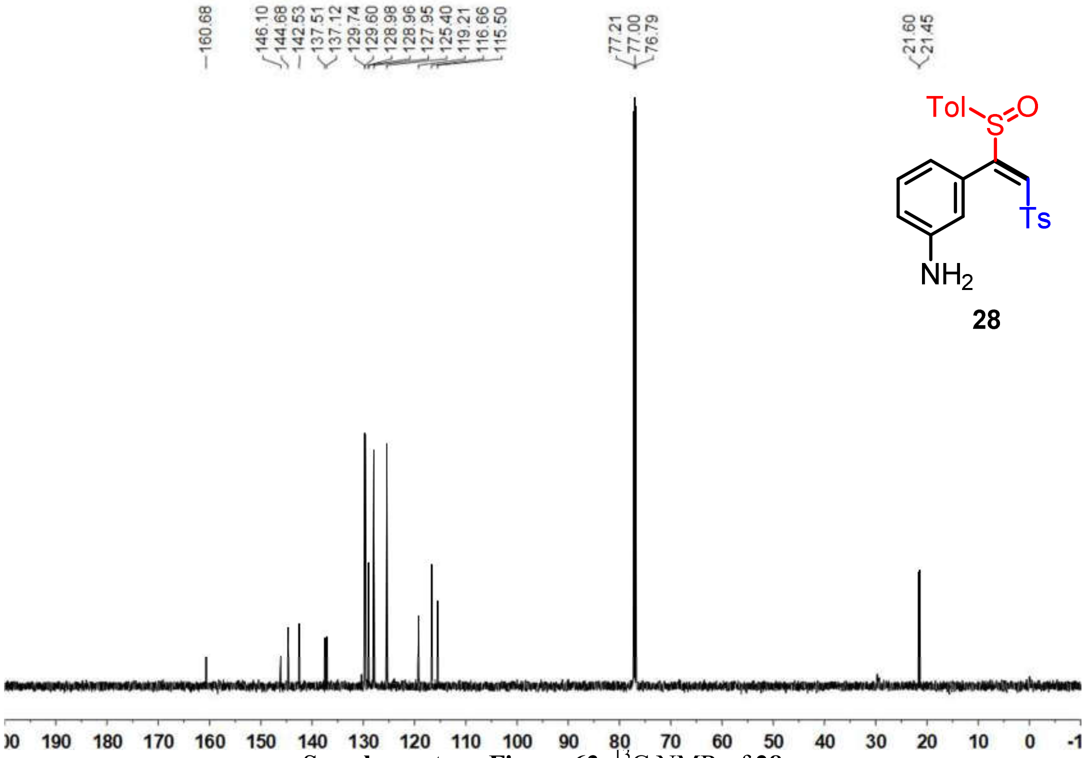

Supplementary Figure 63. ^{13}C NMR of **28**

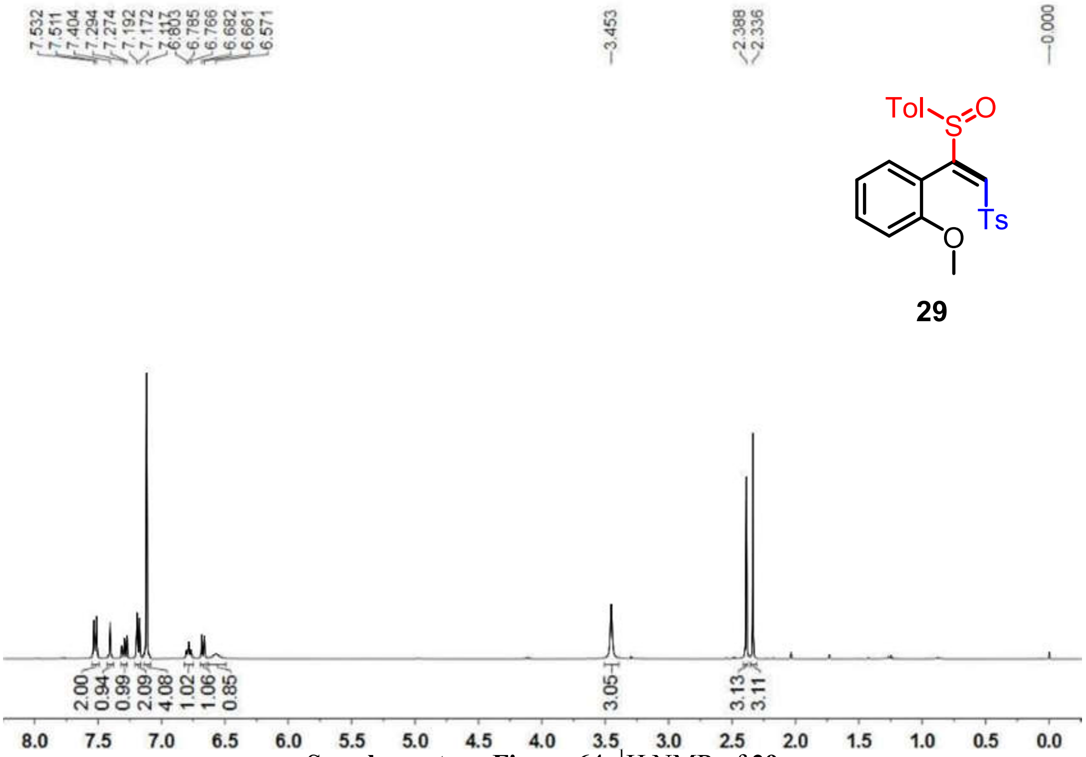

Supplementary Figure 64. ^{1}H NMR of **29**

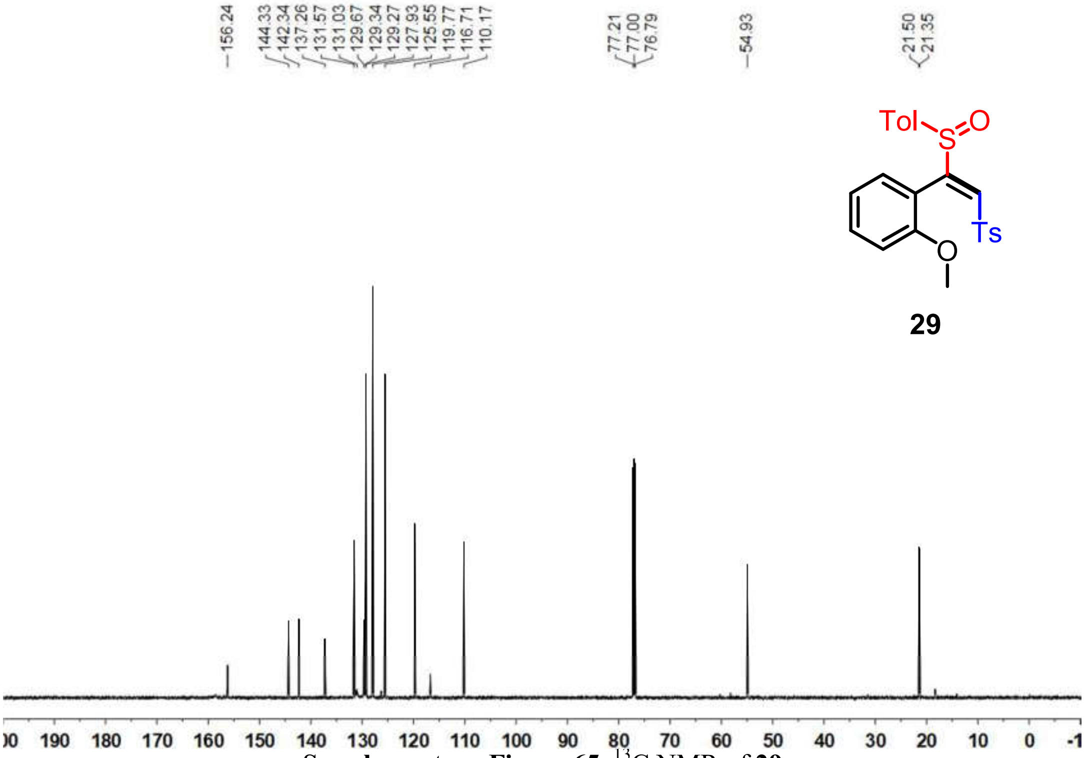

Supplementary Figure 65. ^{13}C NMR of **29**

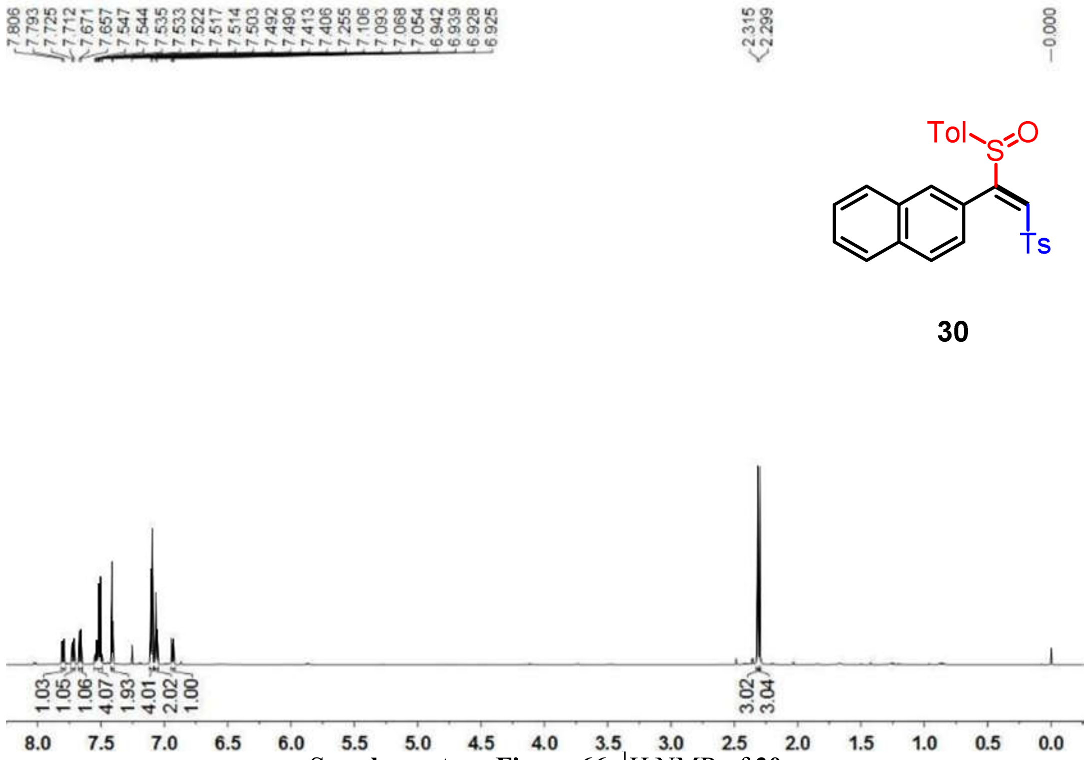

30

Supplementary Figure 66. ^{1}H NMR of **30**

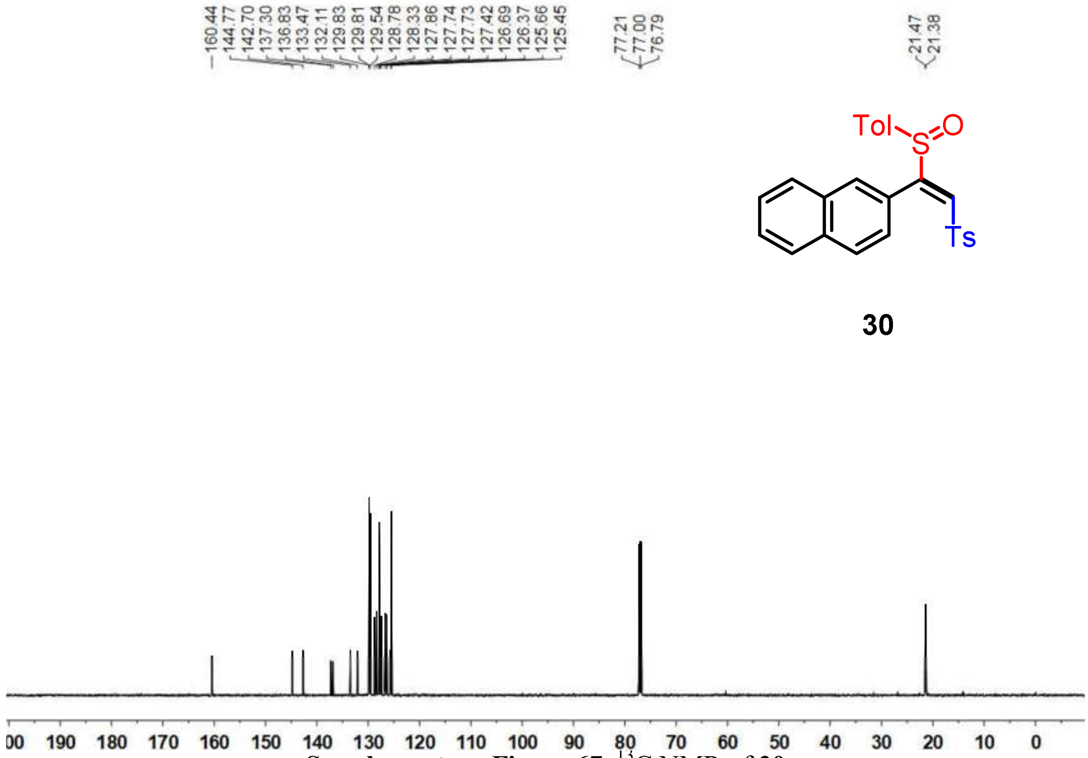

30

Supplementary Figure 67. ^{13}C NMR of **30**

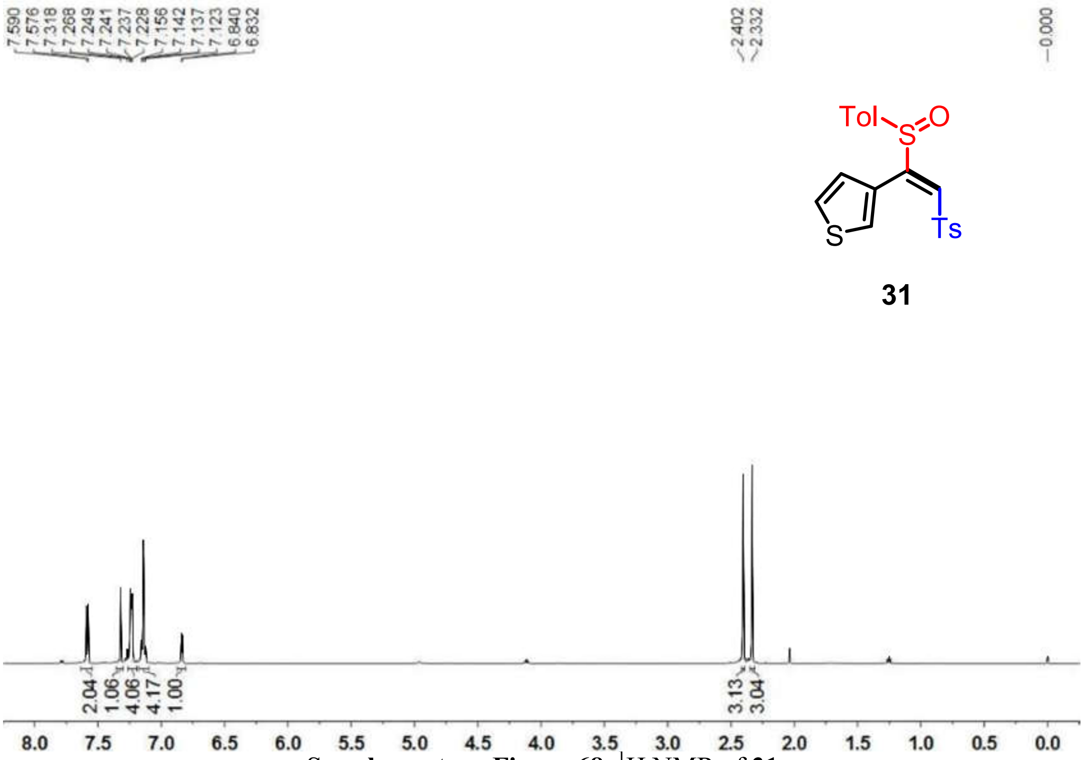

Supplementary Figure 68. ^{1}H NMR of **31**

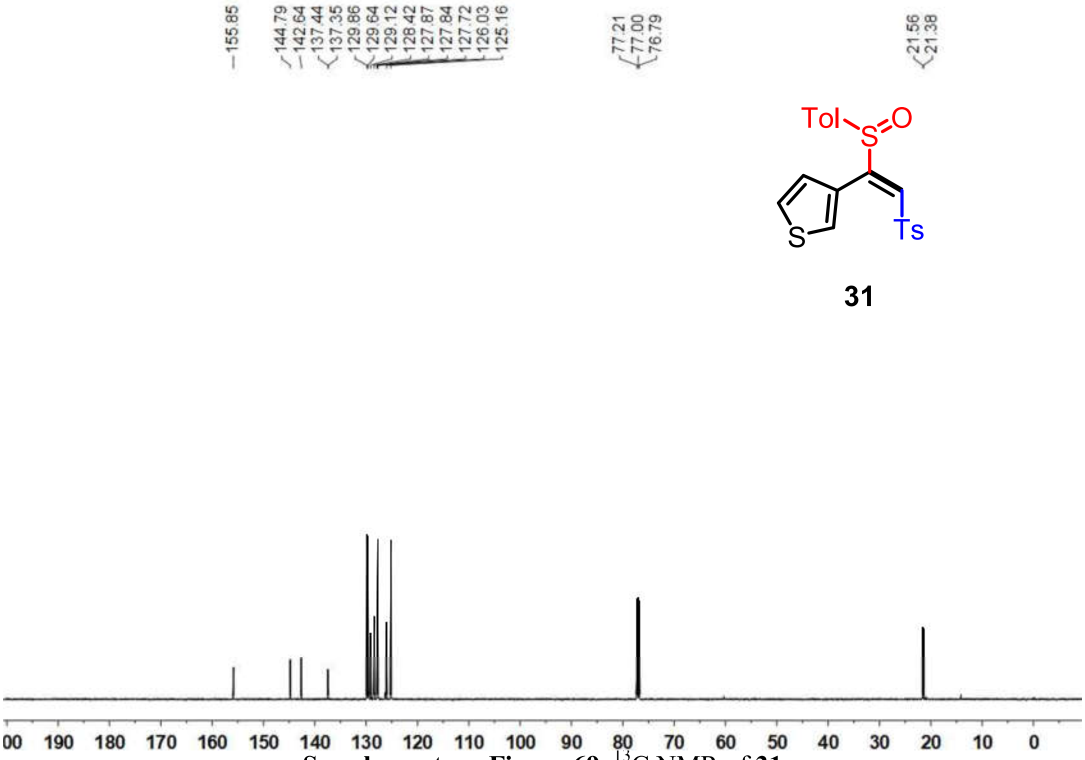

Supplementary Figure 69. ^{13}C NMR of **31**

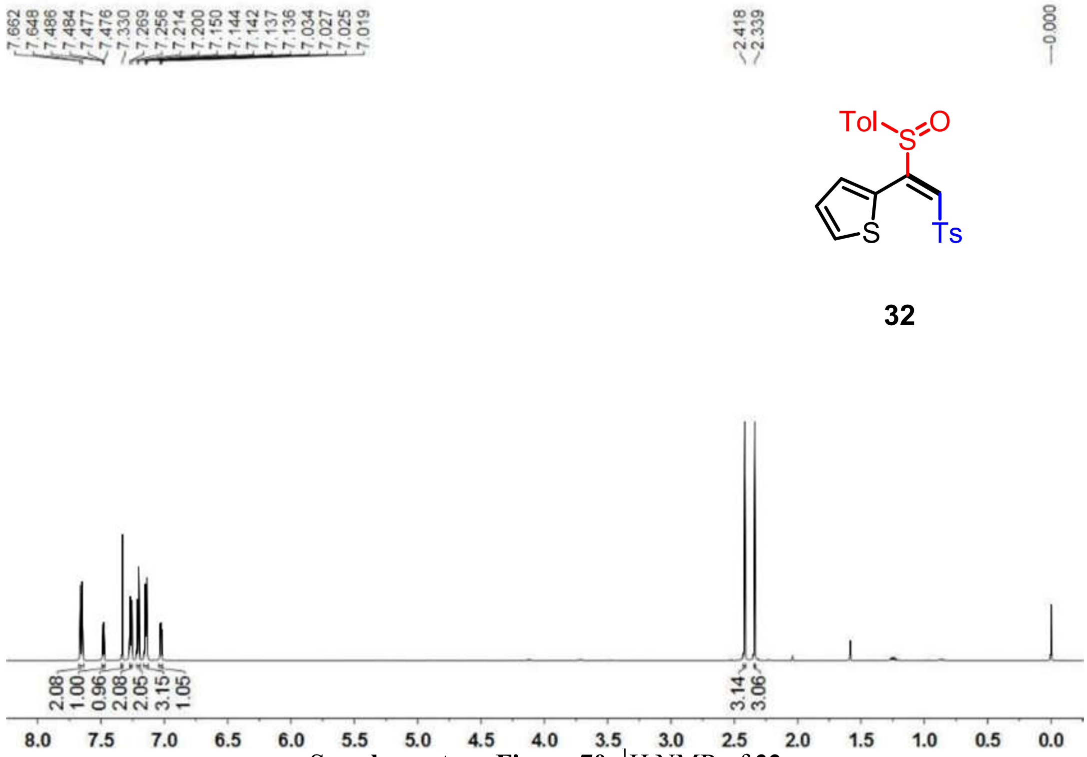

Supplementary Figure 70. ^{1}H NMR of **32**

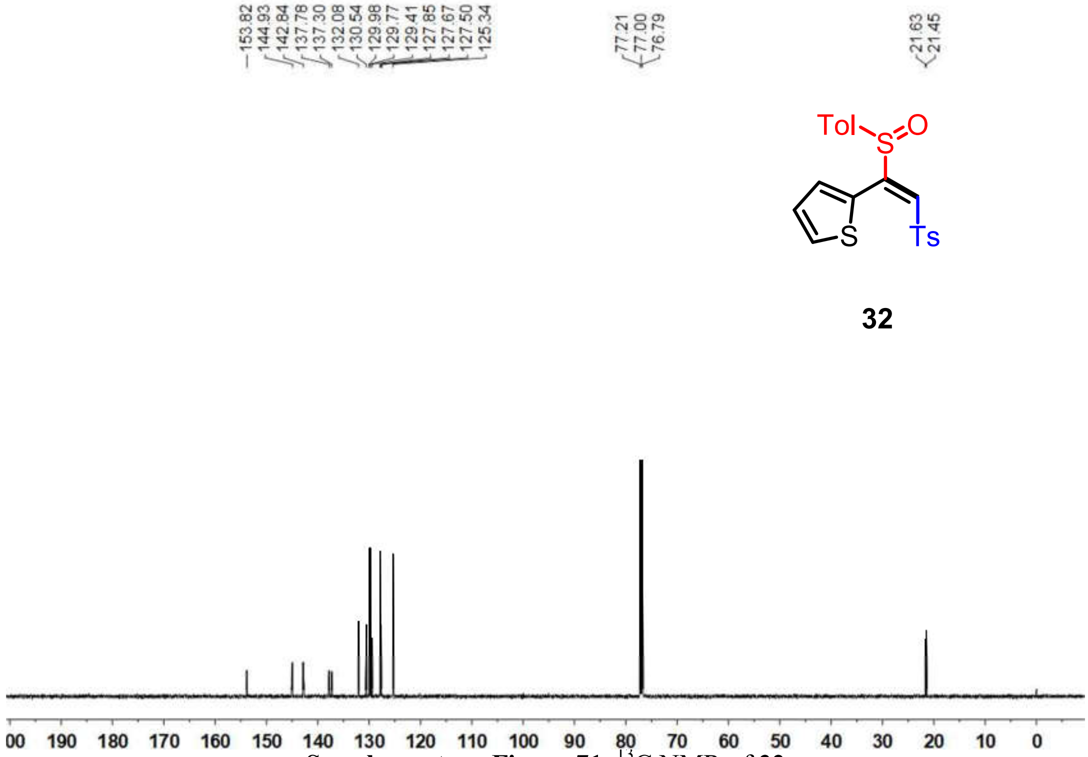

Supplementary Figure 71. ^{13}C NMR of **32**

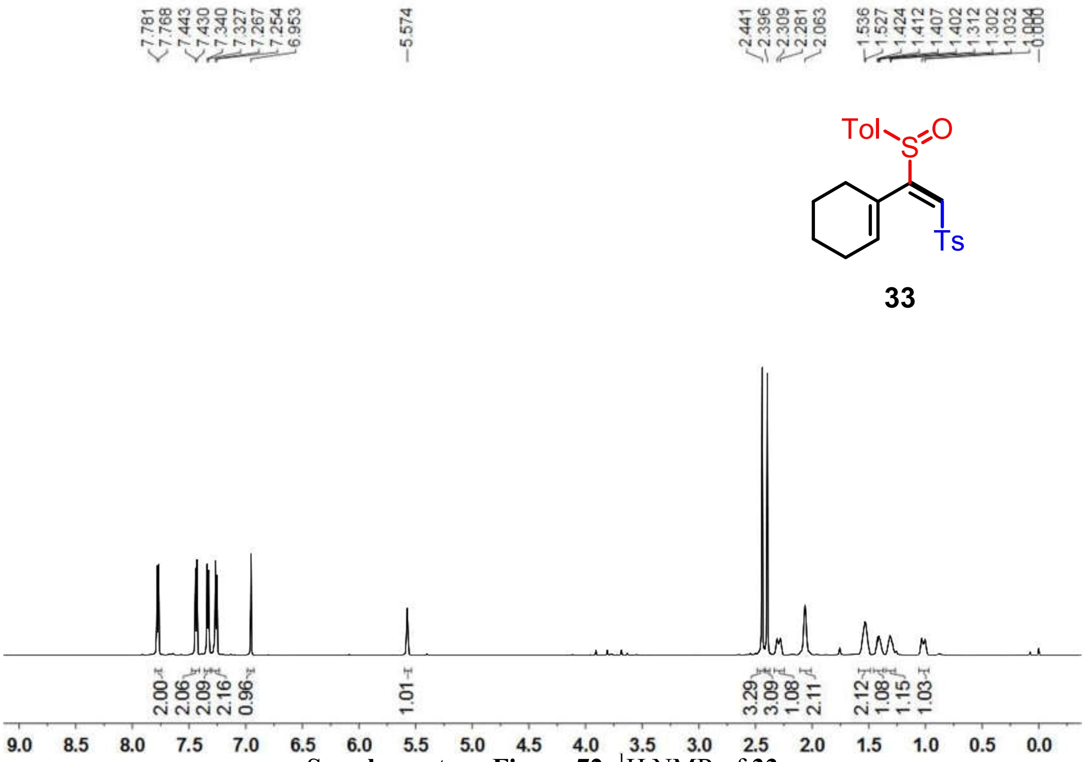

Supplementary Figure 72. ^{1}H NMR of **33**

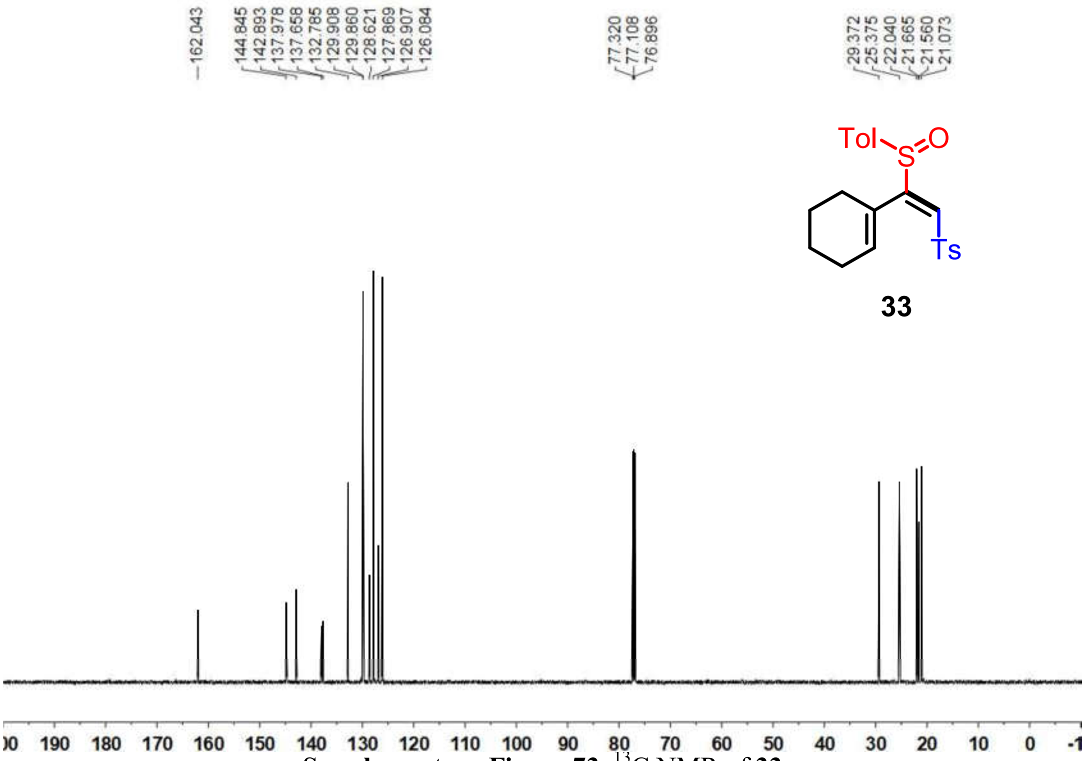

Supplementary Figure 73. ^{13}C NMR of **33**

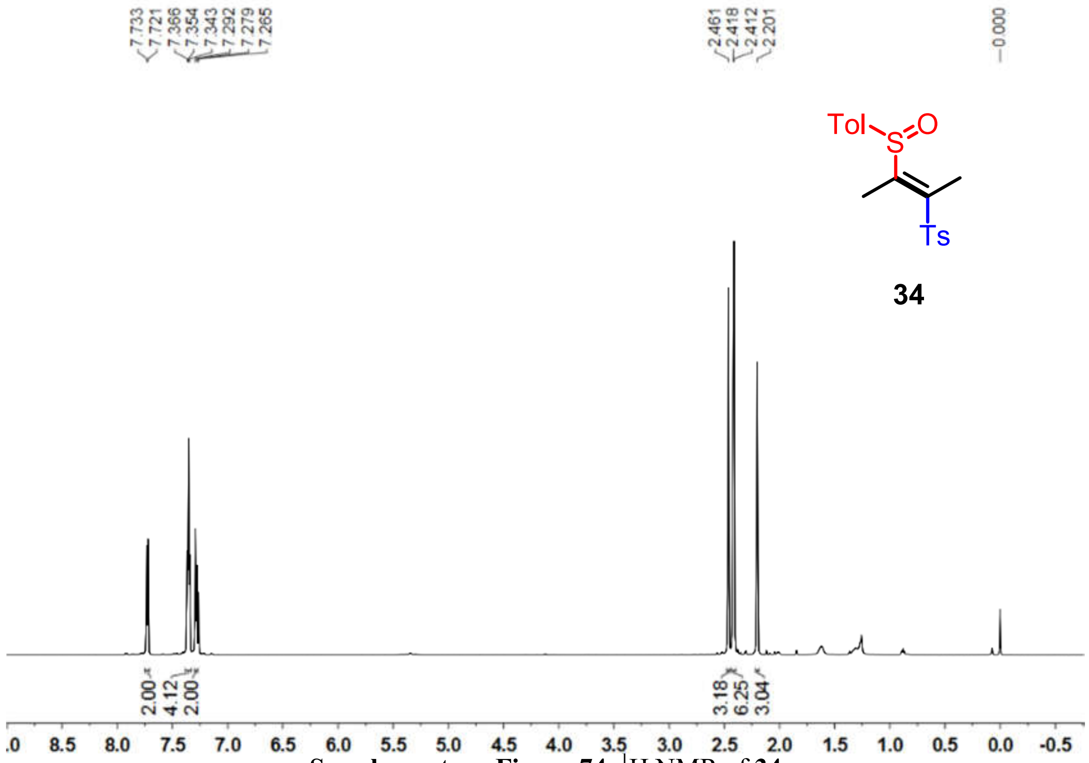

Supplementary Figure 74. ^{1}H NMR of **34**

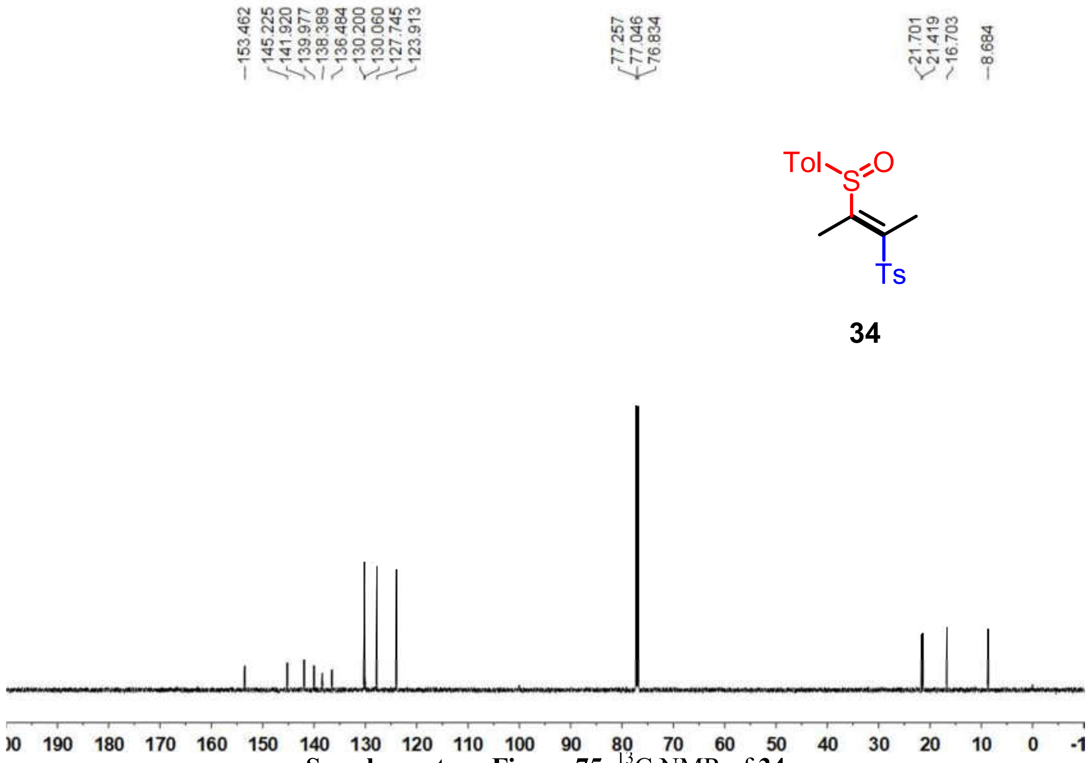

Supplementary Figure 75. ^{13}C NMR of **34**

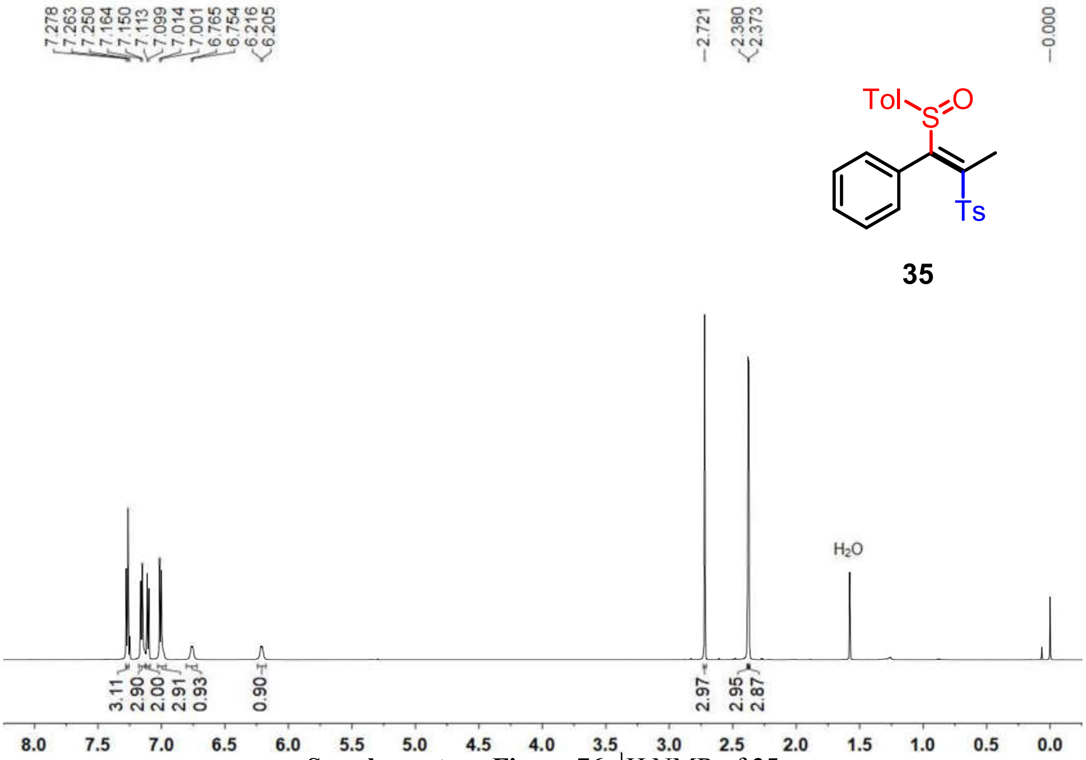

Supplementary Figure 76. ^{1}H NMR of **35**

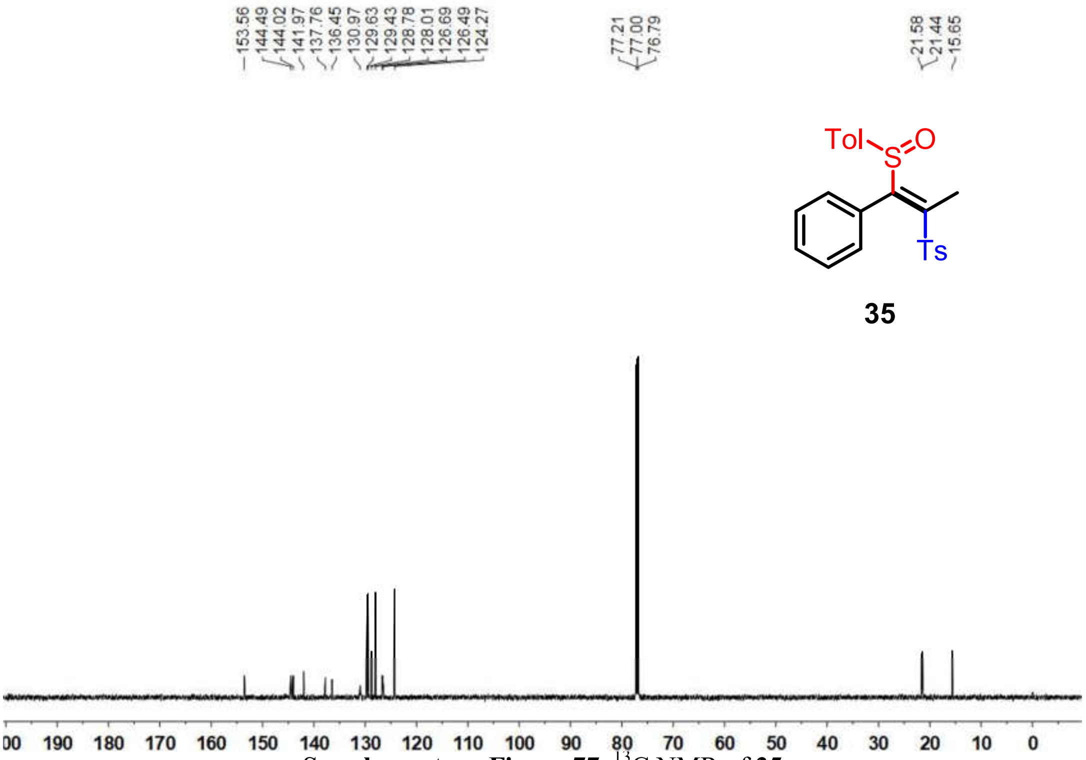

Supplementary Figure 77. ^{13}C NMR of **35**

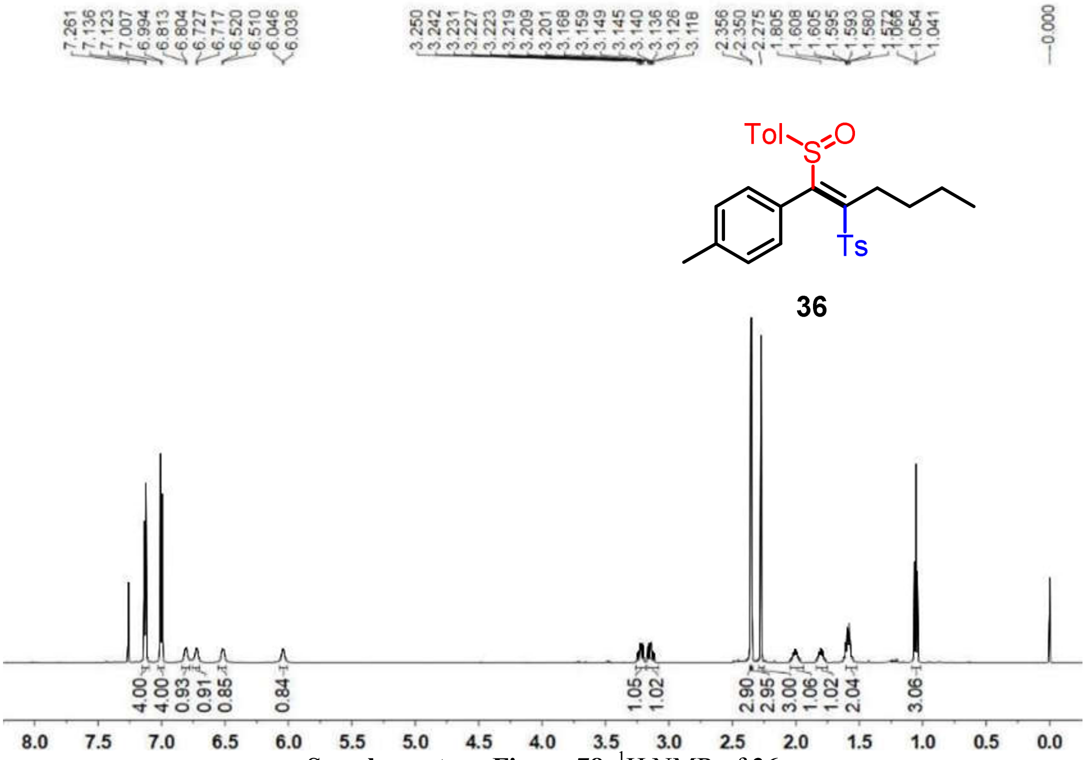

Supplementary Figure 78. ^{1}H NMR of **36**

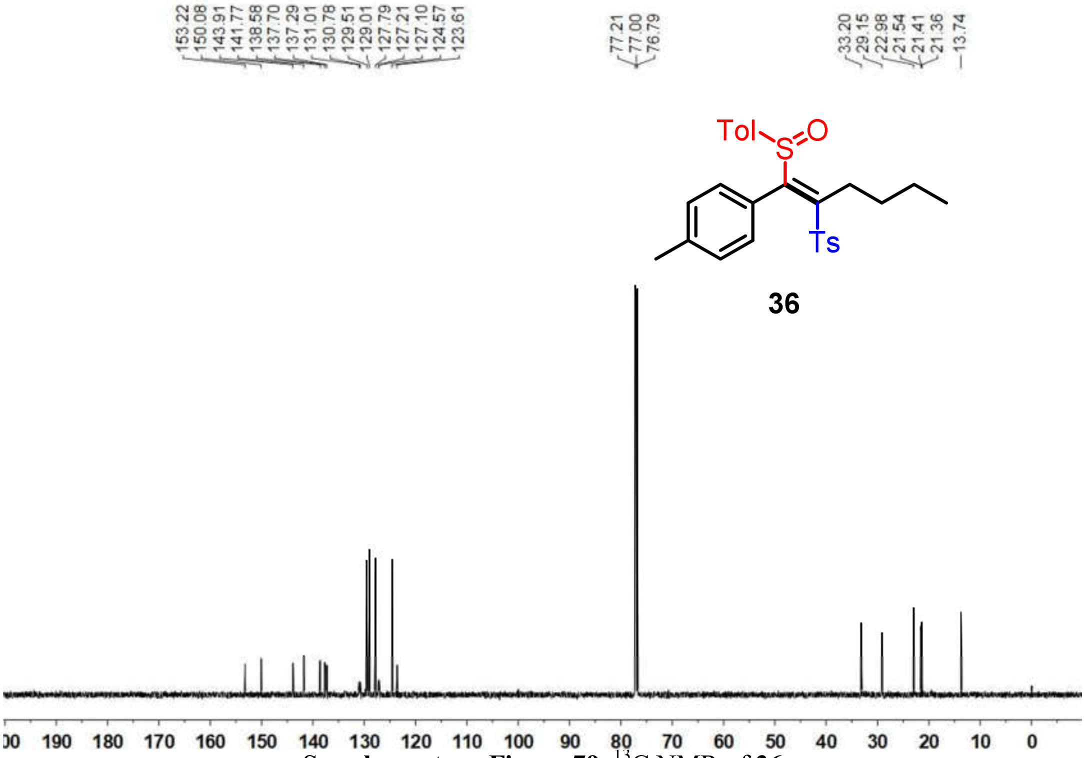

Supplementary Figure 79. ^{13}C NMR of **36**

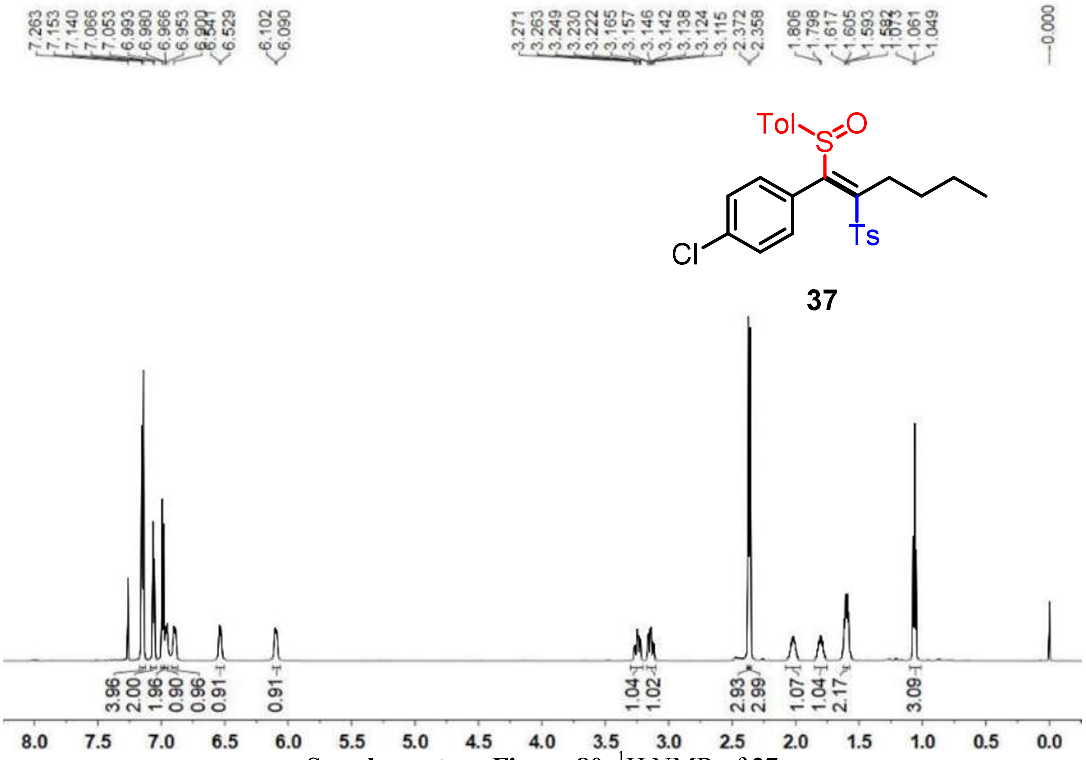

Supplementary Figure 80. ^{1}H NMR of **37**

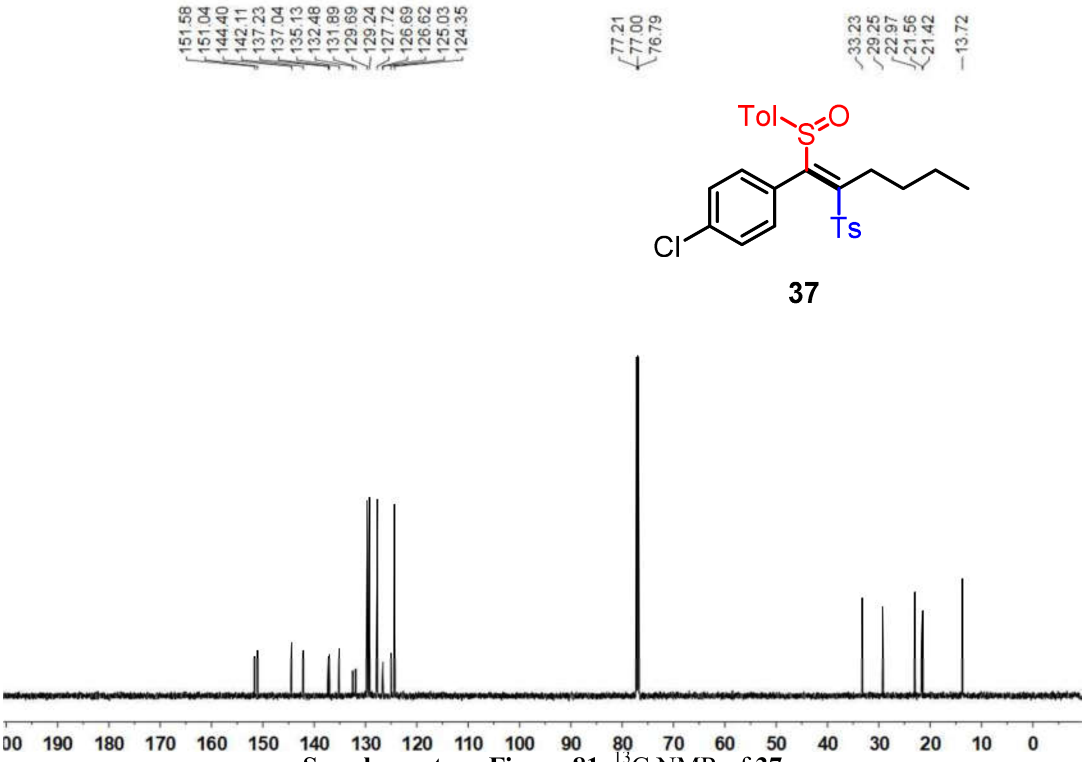

Supplementary Figure 81. ^{13}C NMR of **37**

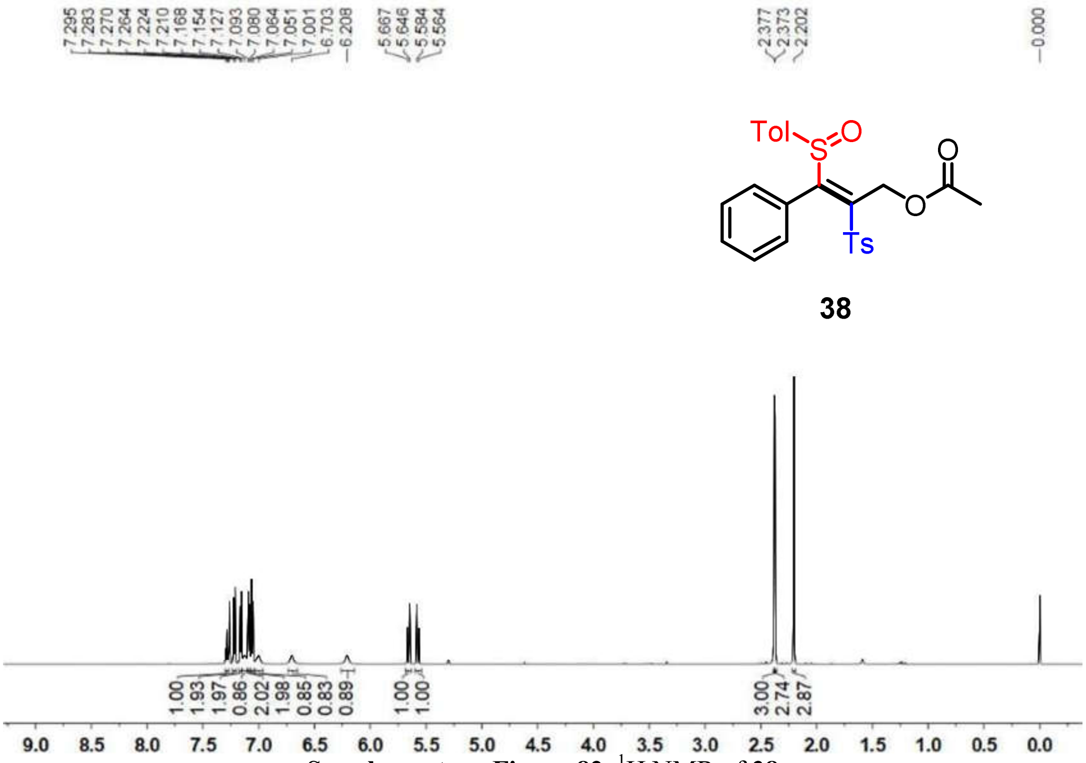

Supplementary Figure 82. ^{1}H NMR of **38**

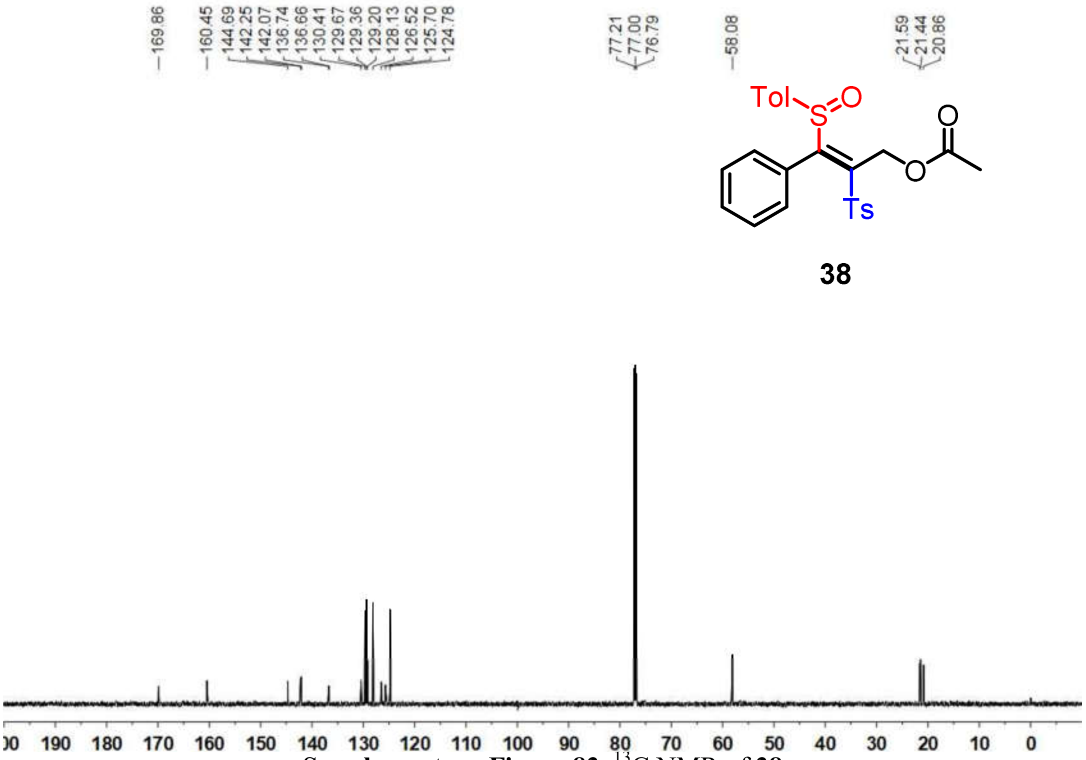

Supplementary Figure 83. ^{13}C NMR of **38**

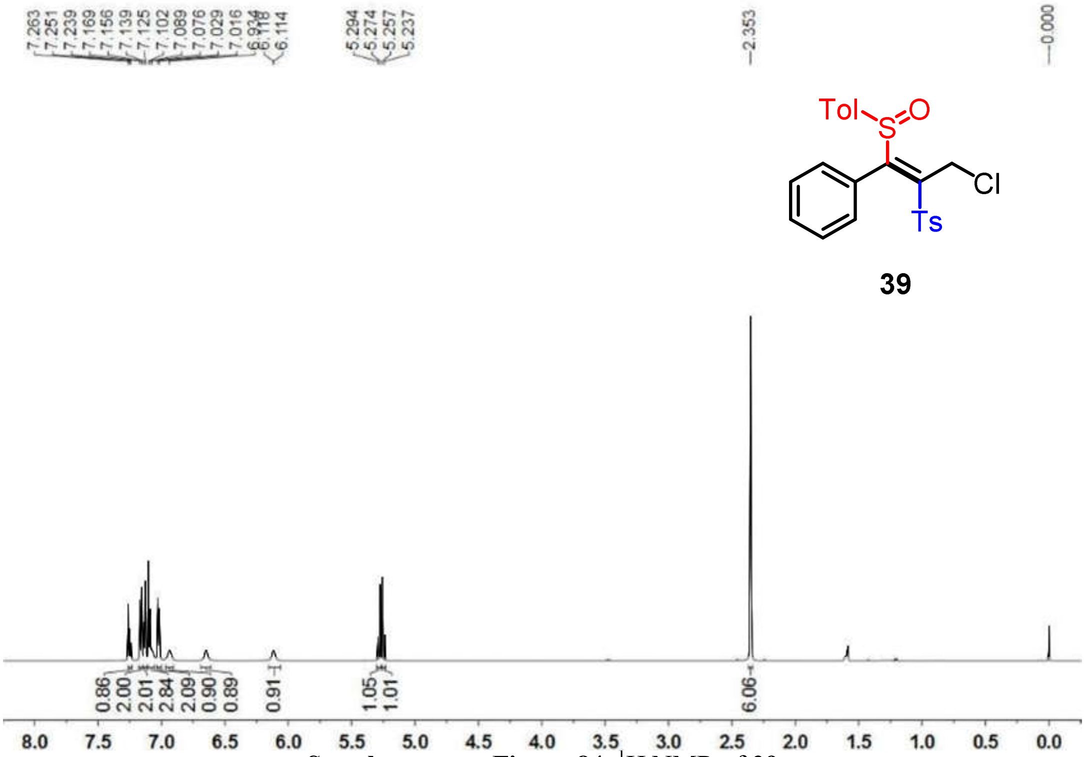

Supplementary Figure 84. ^{1}H NMR of **39**

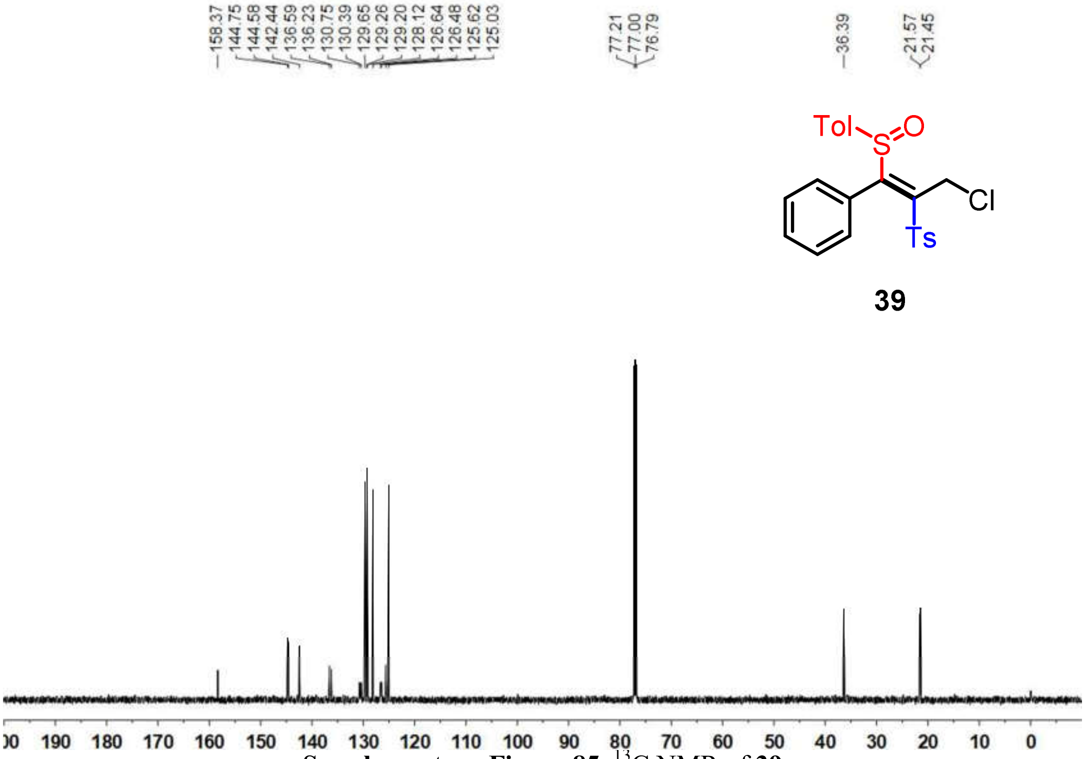

Supplementary Figure 85. ^{13}C NMR of **39**

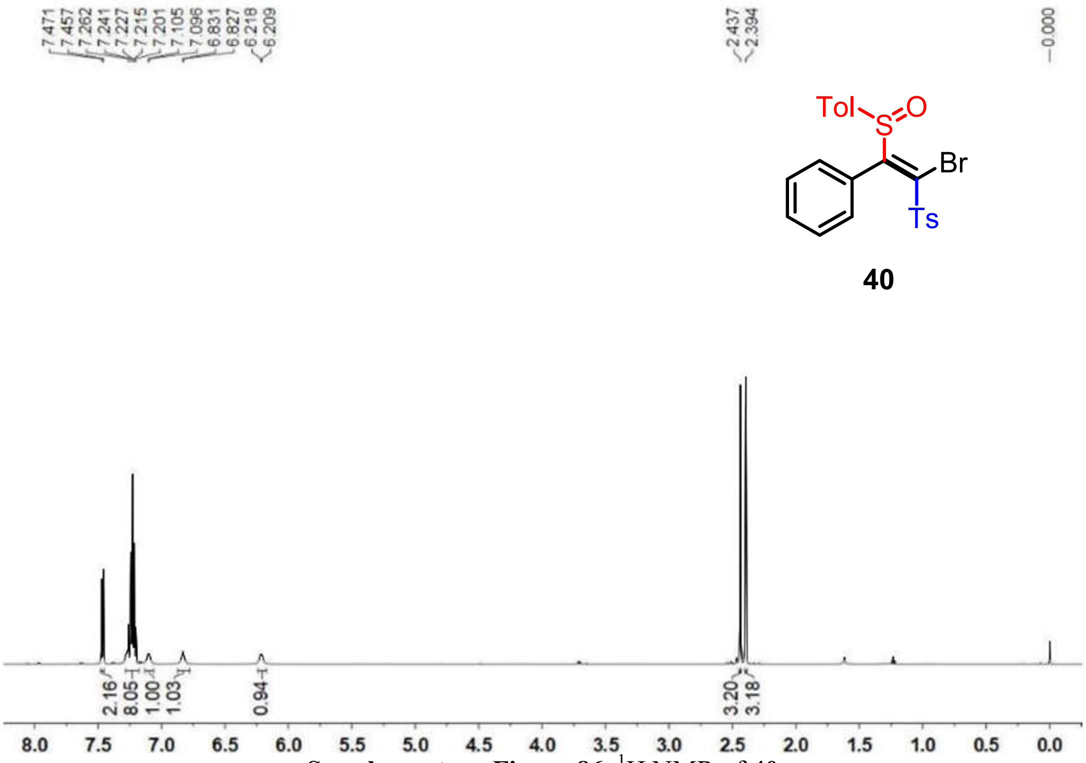

Supplementary Figure 86. ^{1}H NMR of **40**

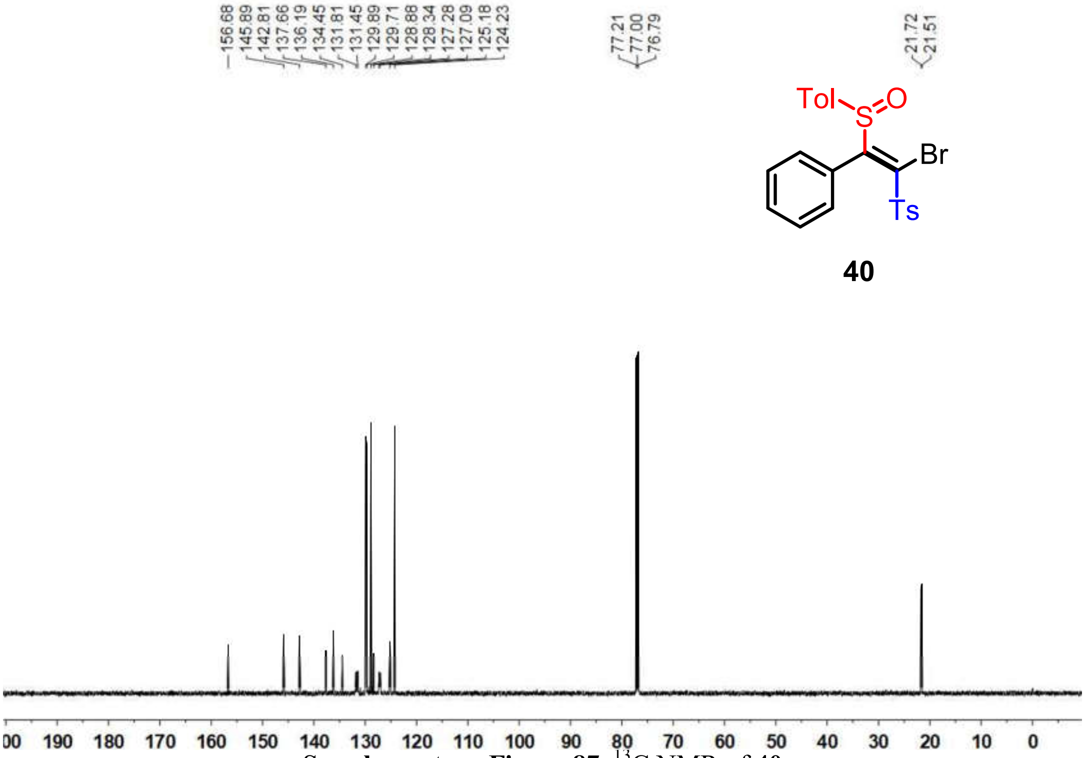

Supplementary Figure 87. ^{13}C NMR of **40**

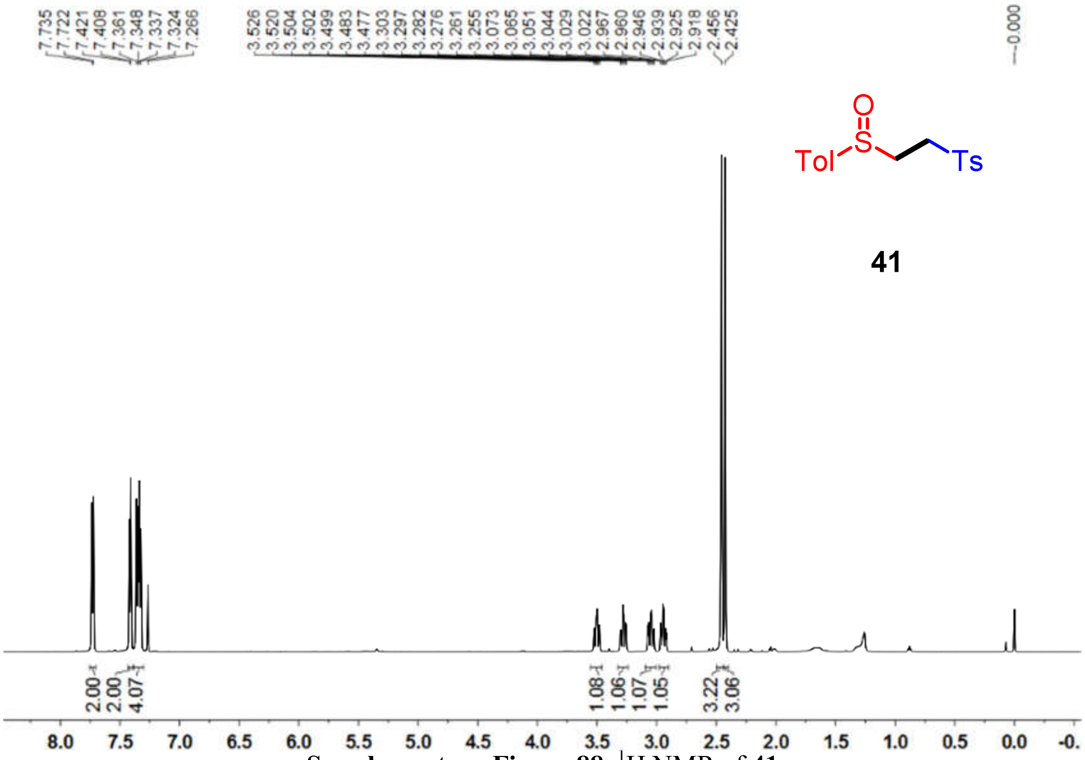

Supplementary Figure 88. ^{1}H NMR of **41**

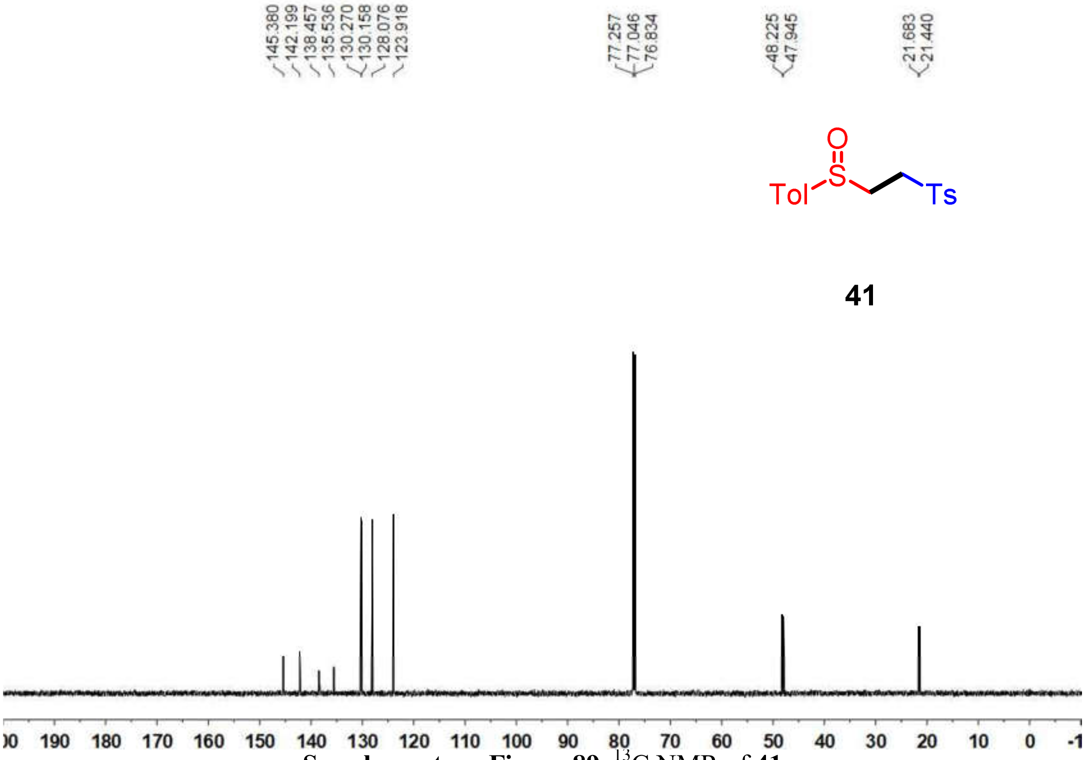

Supplementary Figure 89. ^{13}C NMR of **41**

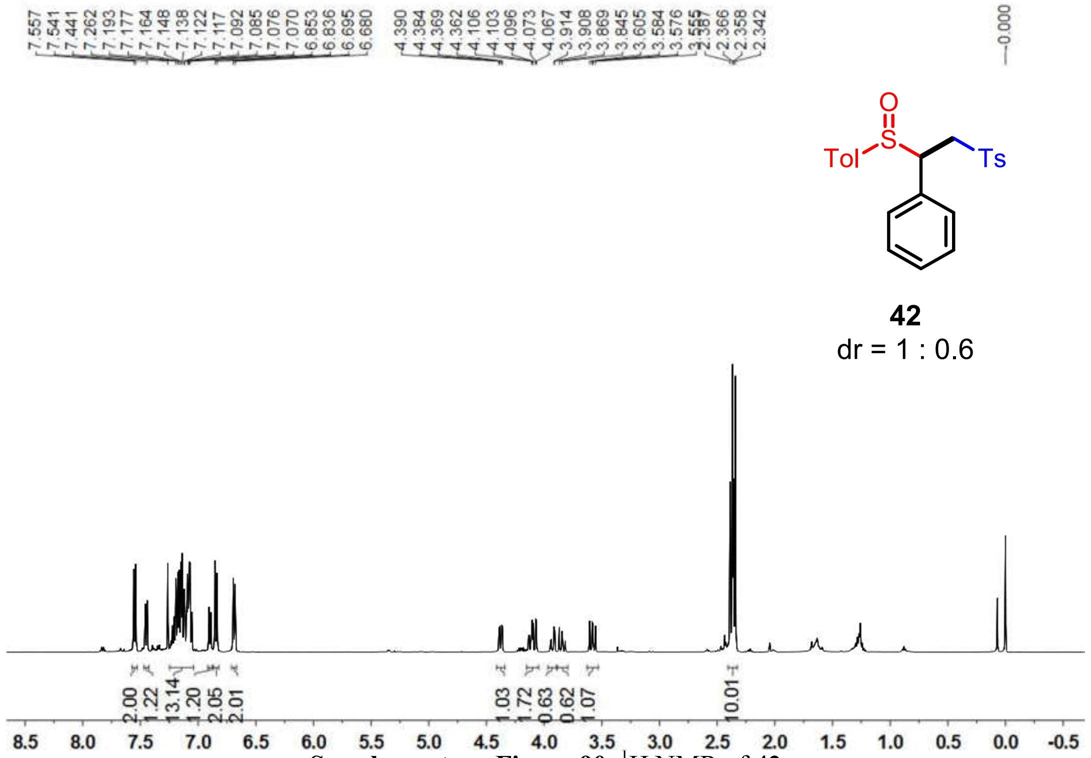

Supplementary Figure 90. ^{1}H NMR of **42**

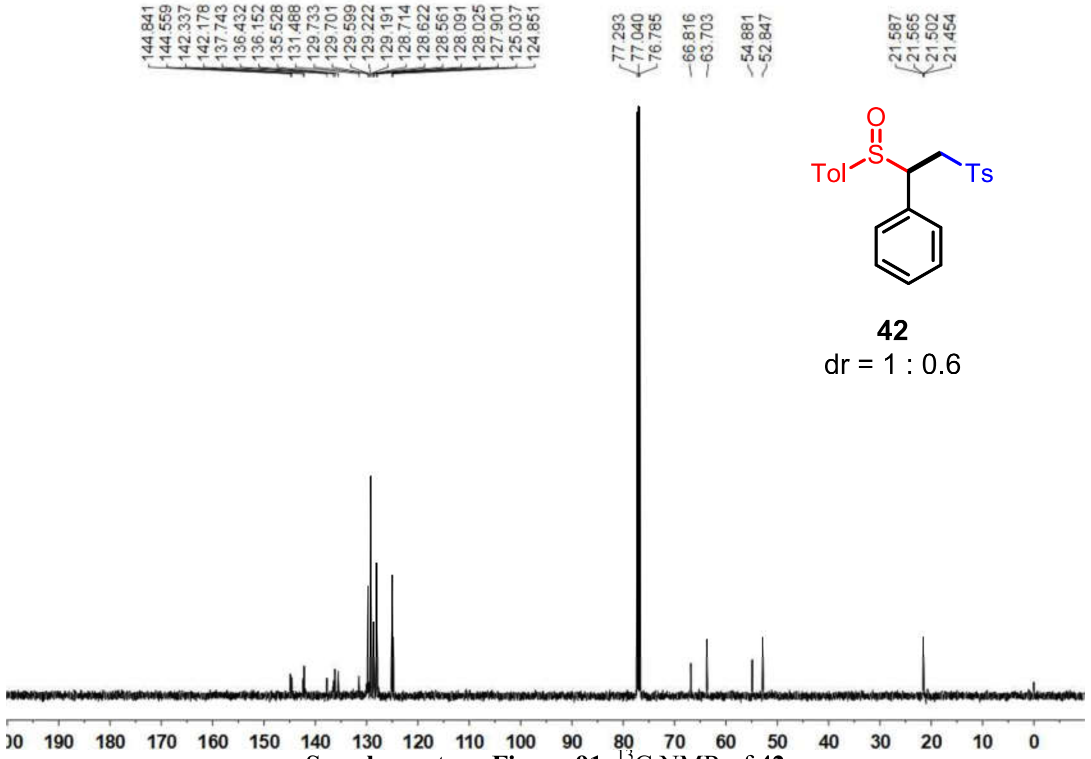

Supplementary Figure 91. ^{13}C NMR of **42**

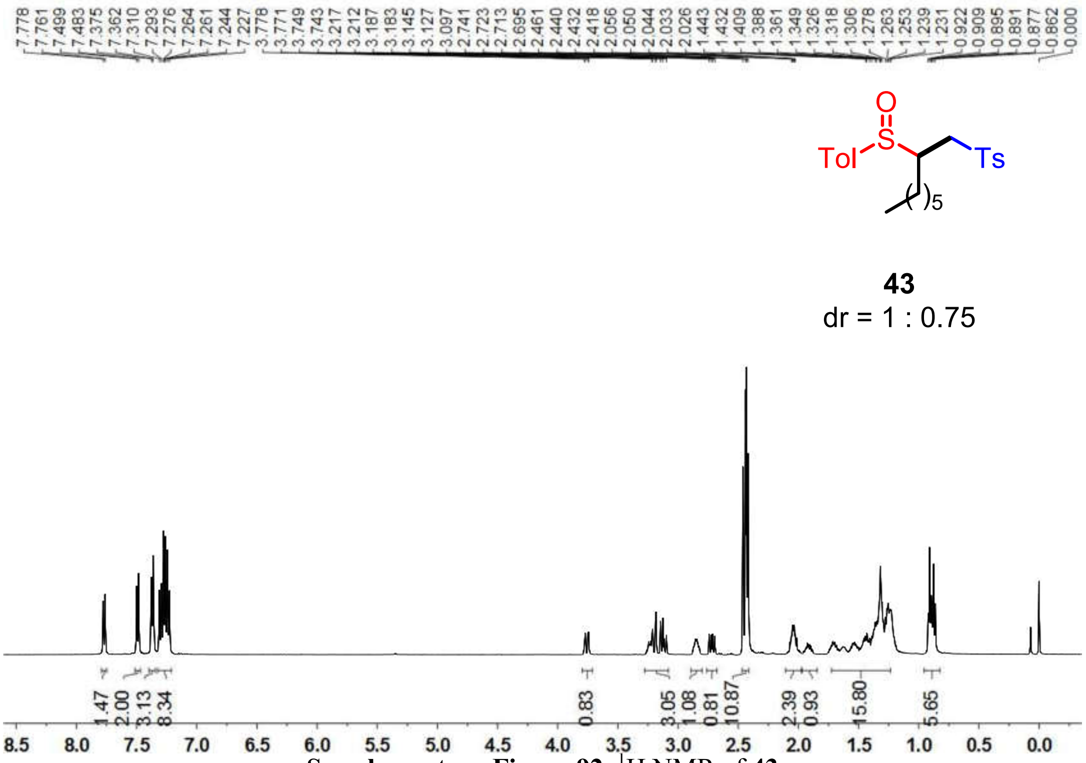

Supplementary Figure 92. ^{1}H NMR of **43**

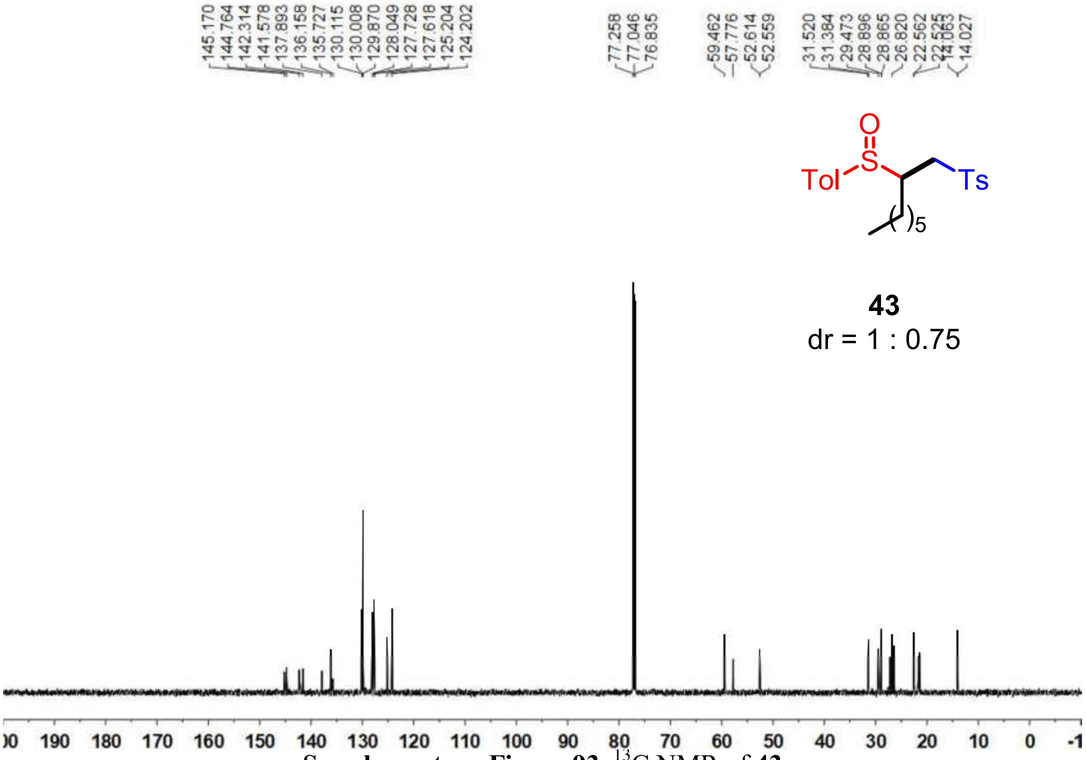

Supplementary Figure 93. ^{13}C NMR of **43**

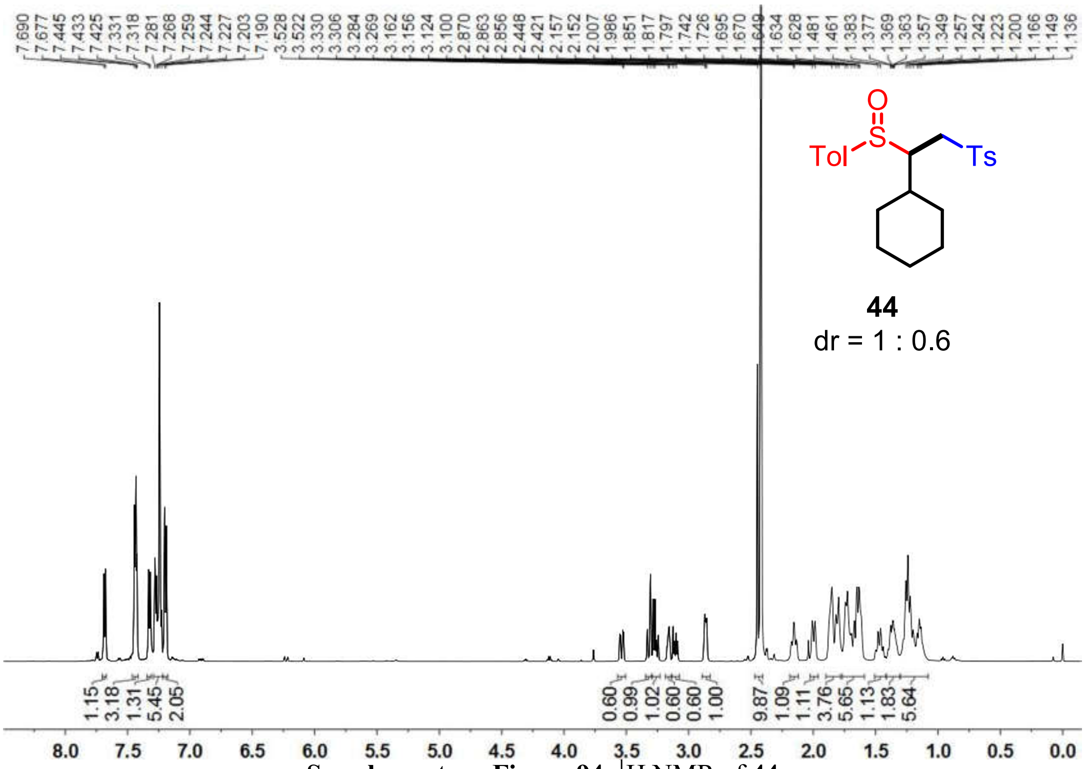

Supplementary Figure 94. ^{1}H NMR of **44**

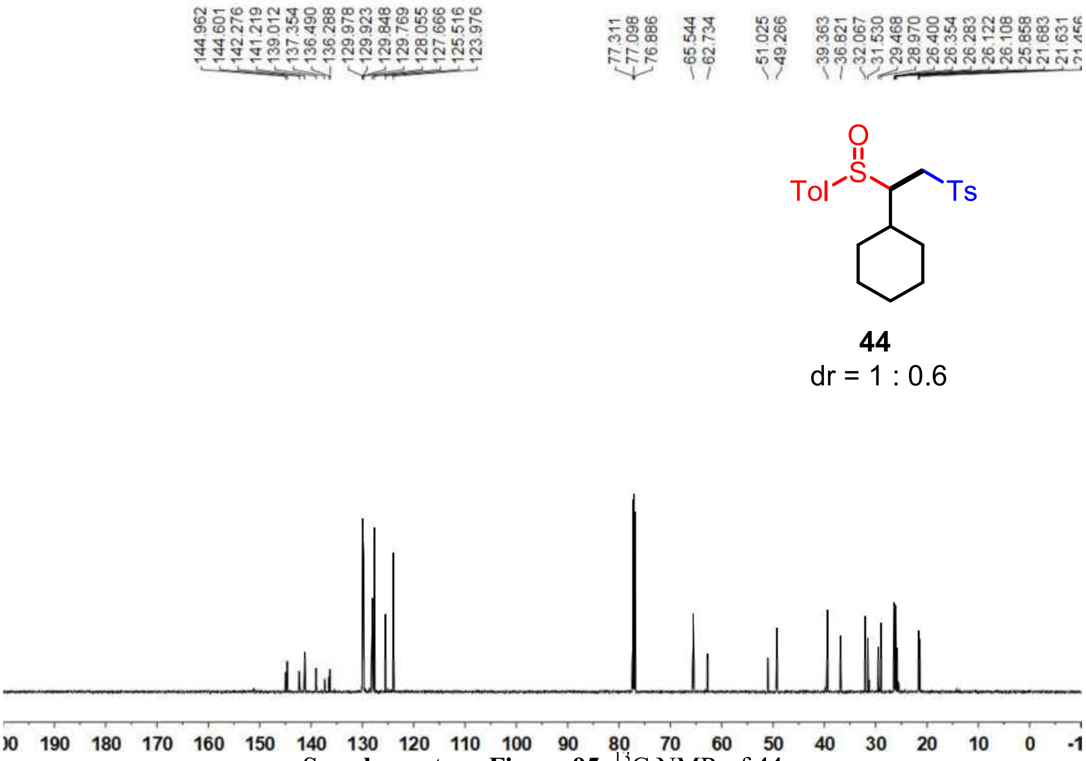

Supplementary Figure 95. ^{13}C NMR of **44**

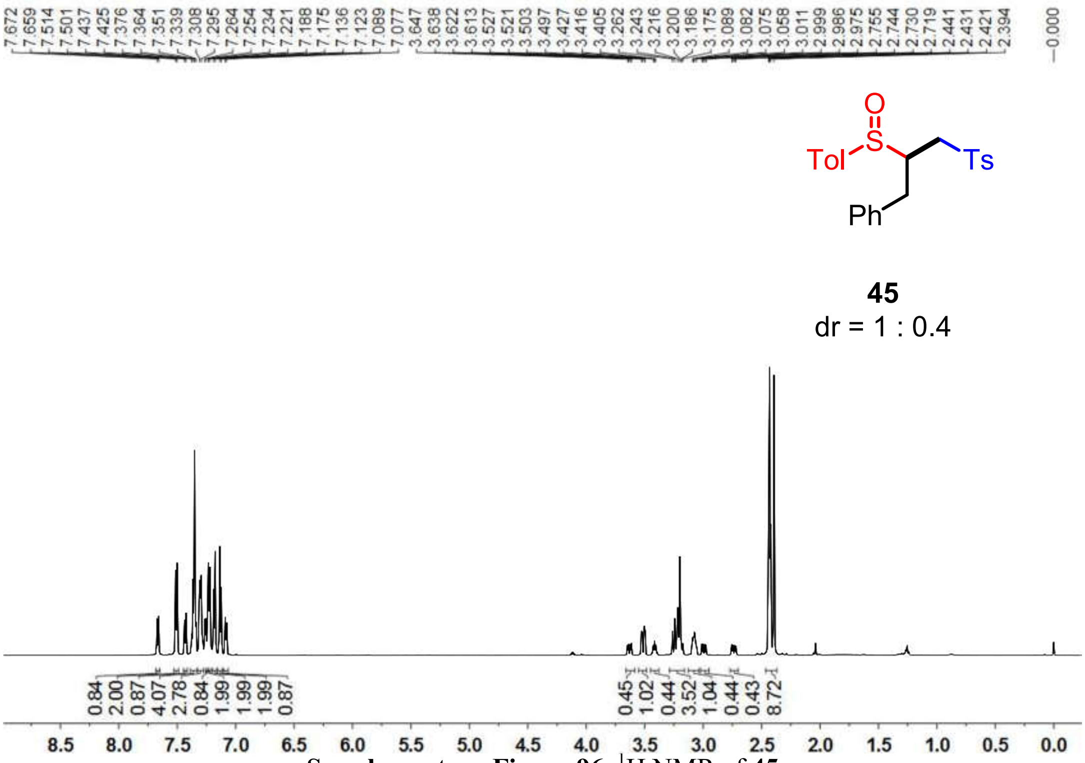

Supplementary Figure 96. ^{1}H NMR of **45**

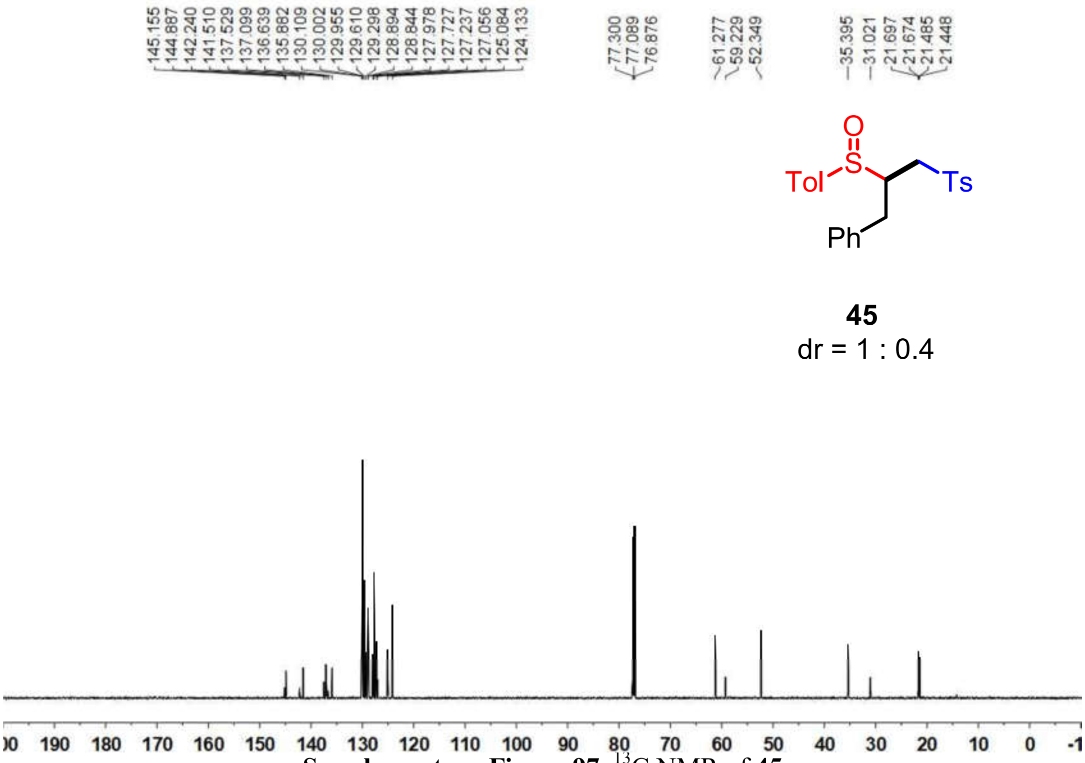

Supplementary Figure 97. ^{13}C NMR of **45**

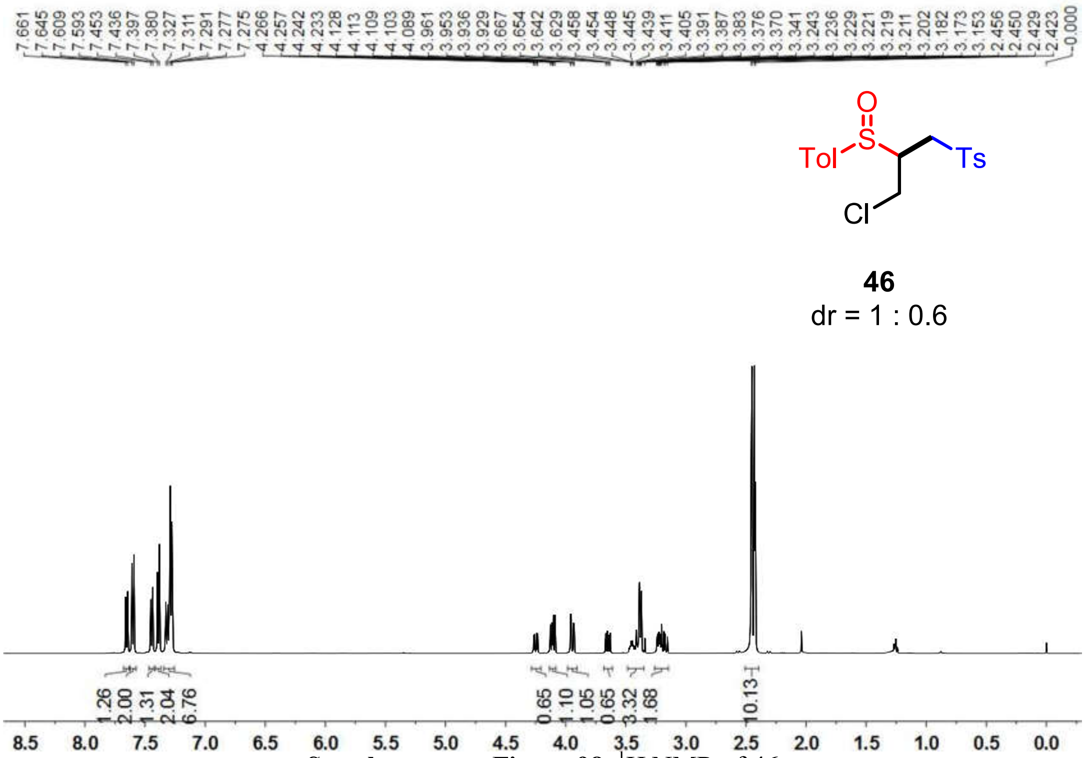

Supplementary Figure 98. ^{1}H NMR of **46**

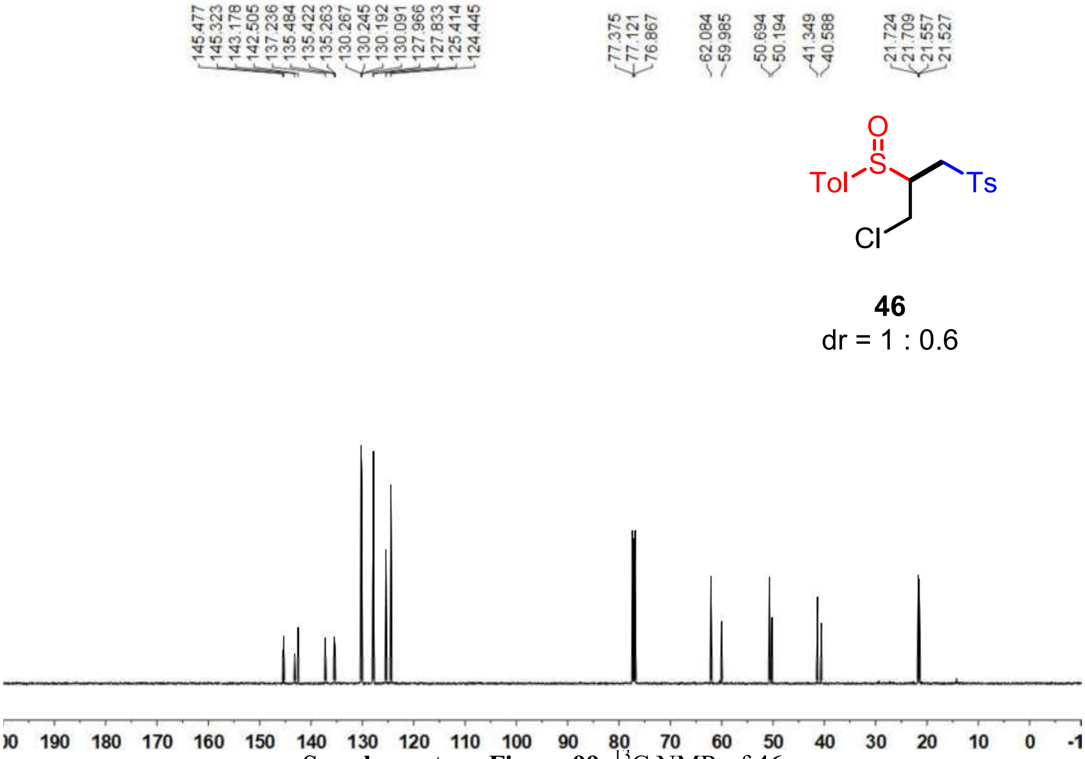

Supplementary Figure 99. ^{13}C NMR of **46**

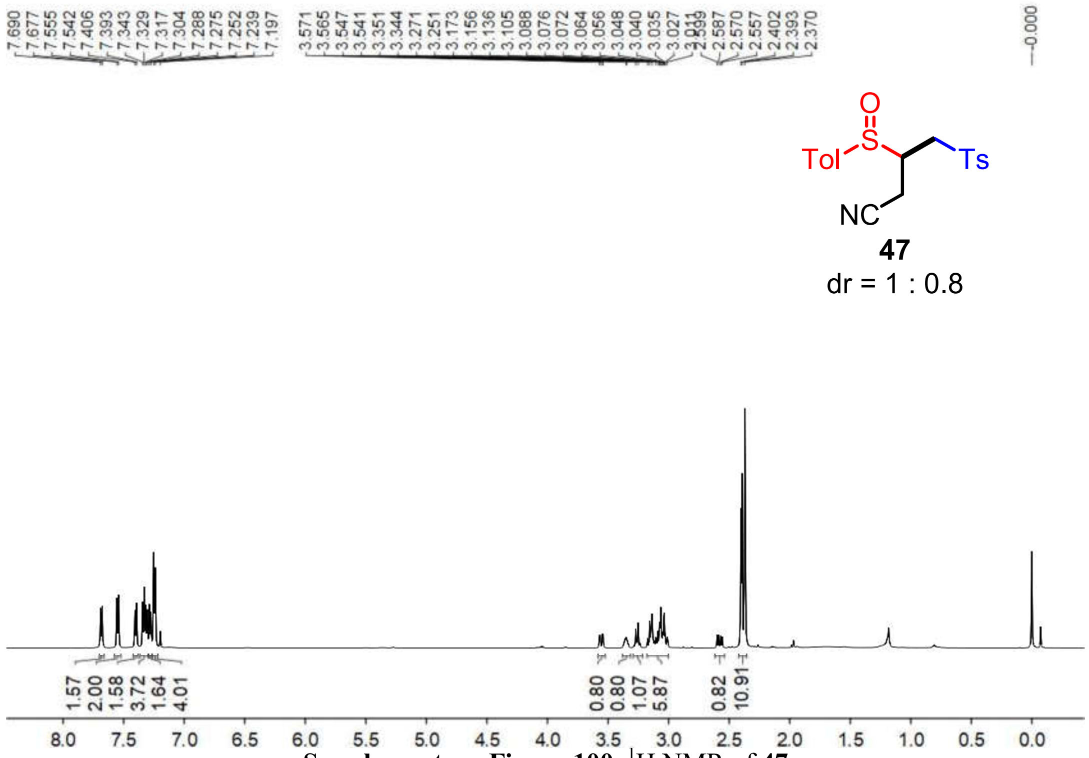

Supplementary Figure 100. ^{1}H NMR of **47**

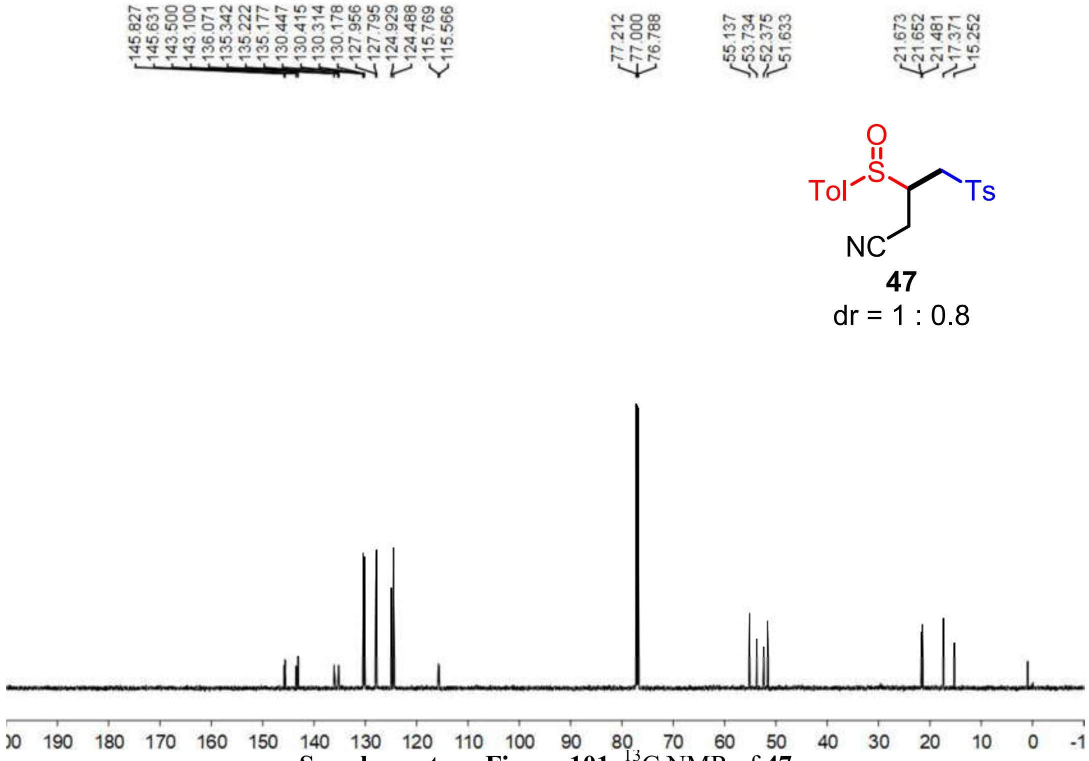

Supplementary Figure 101. ^{13}C NMR of **47**

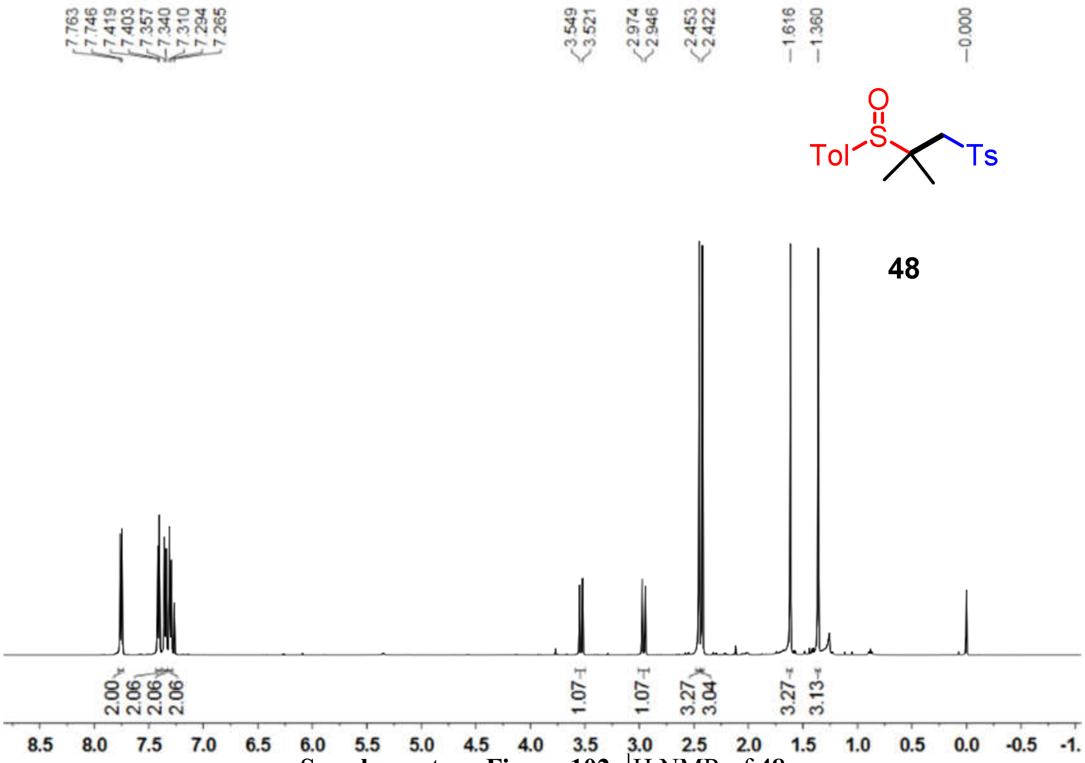

Supplementary Figure 102. ^{1}H NMR of **48**

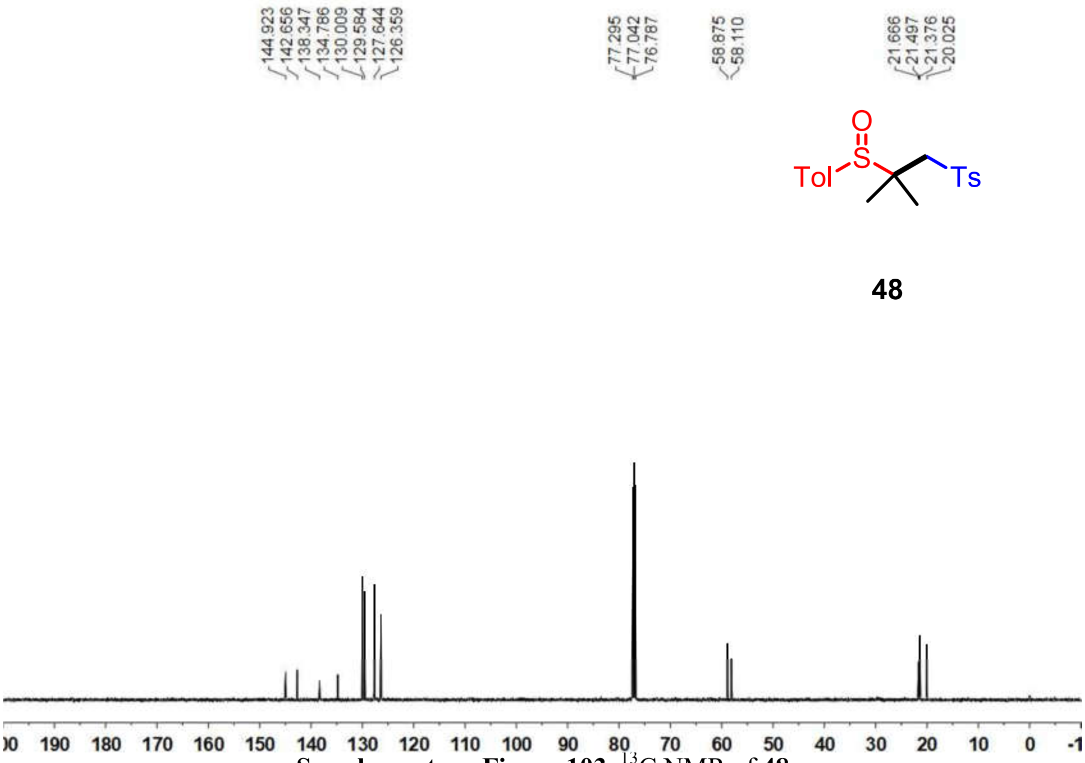

Supplementary Figure 103. ^{13}C NMR of **48**

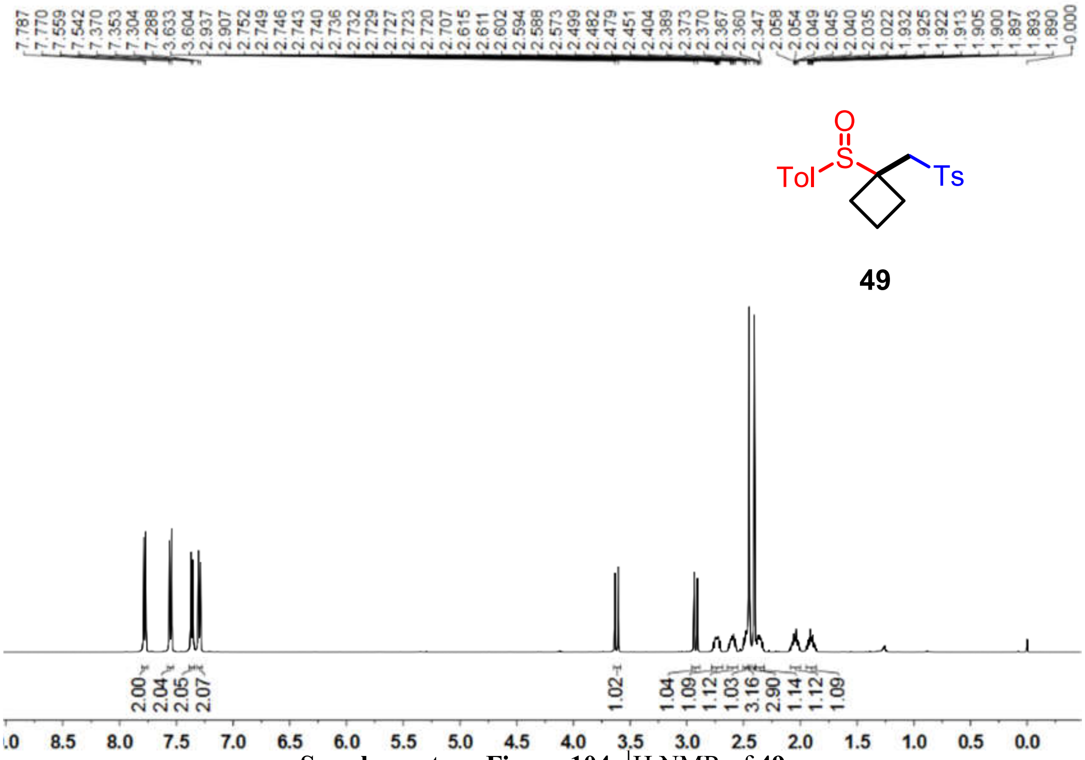

Supplementary Figure 104. ^{1}H NMR of **49**

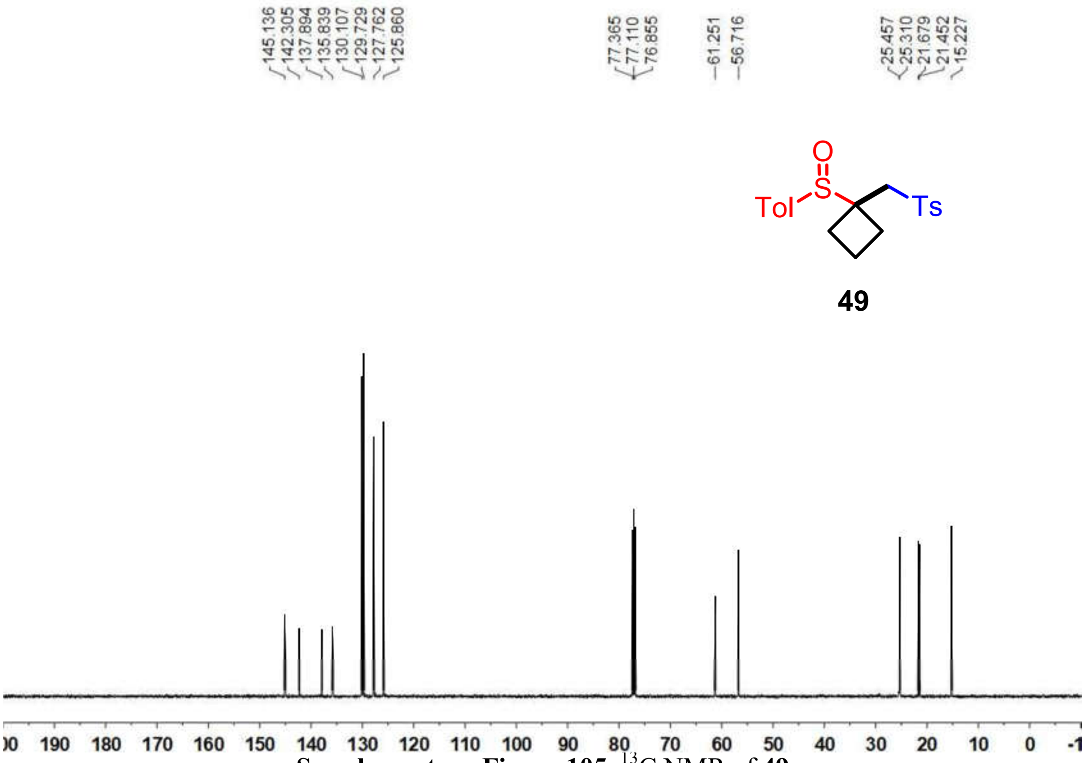

Supplementary Figure 105. ^{13}C NMR of **49**

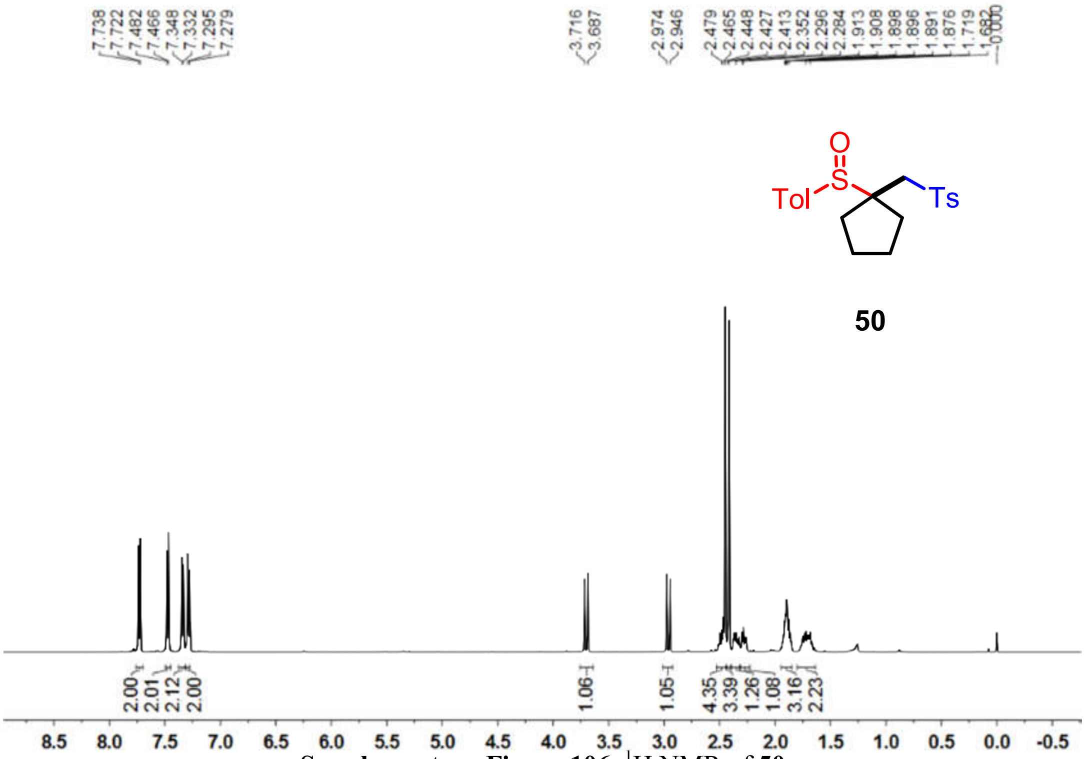

Supplementary Figure 106. ^{1}H NMR of **50**

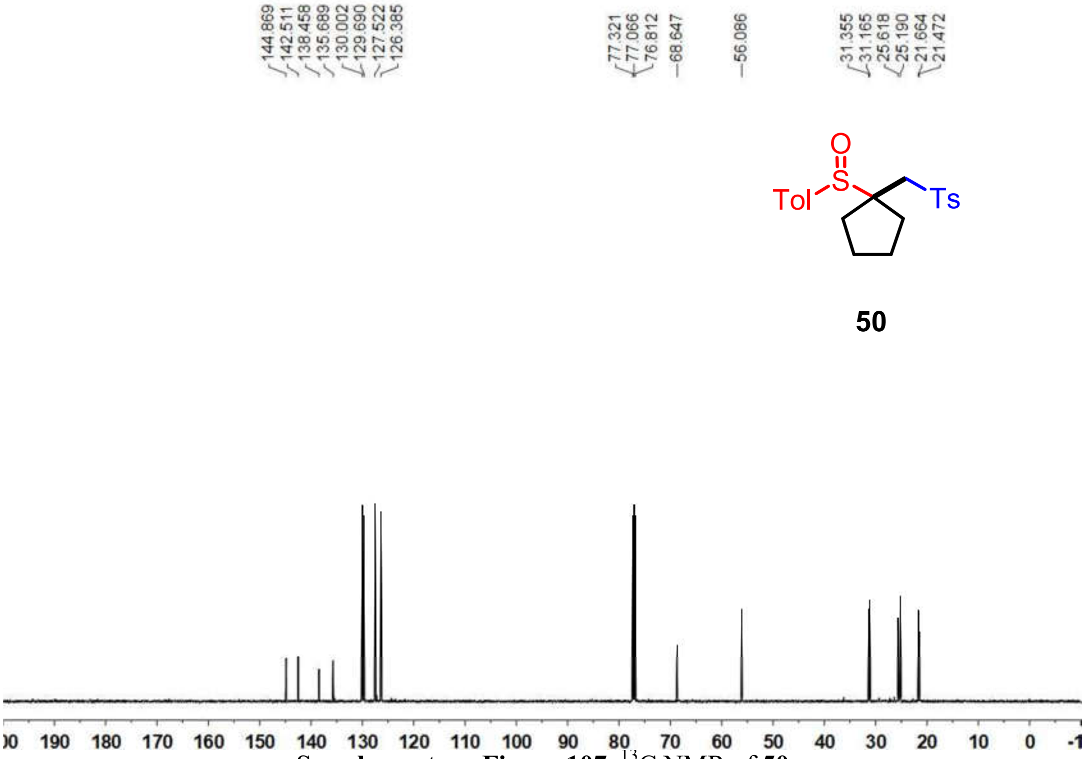

Supplementary Figure 107. ^{13}C NMR of **50**

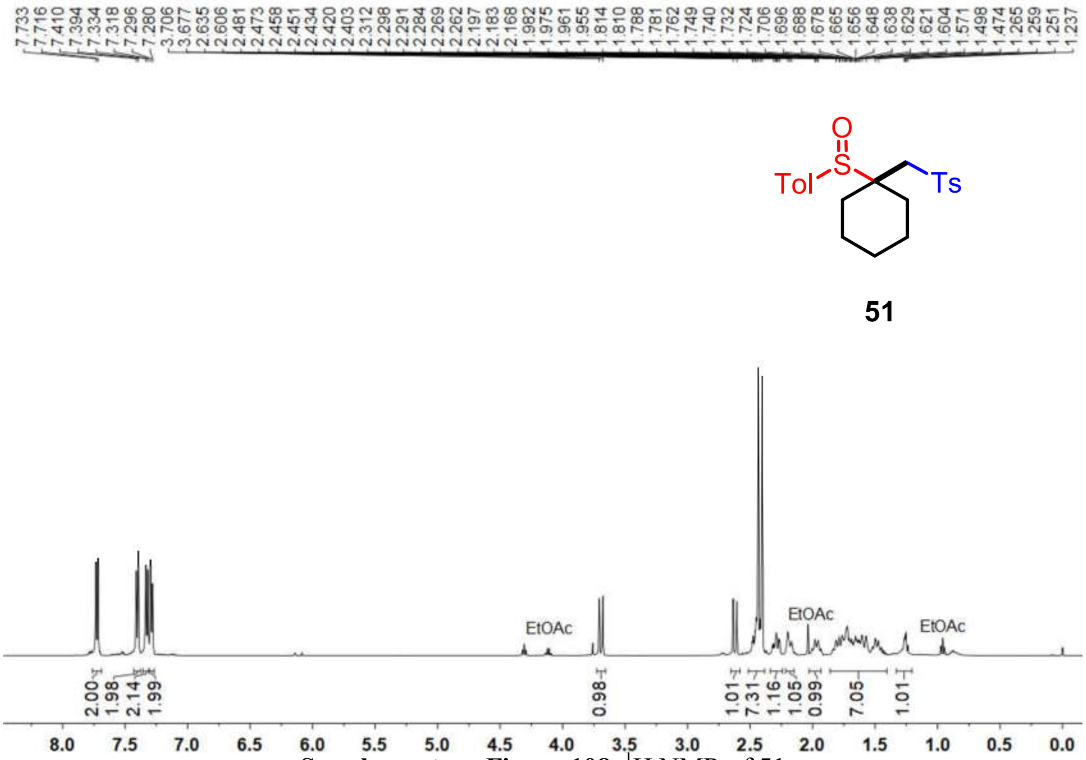

Supplementary Figure 108. ^{1}H NMR of **51**

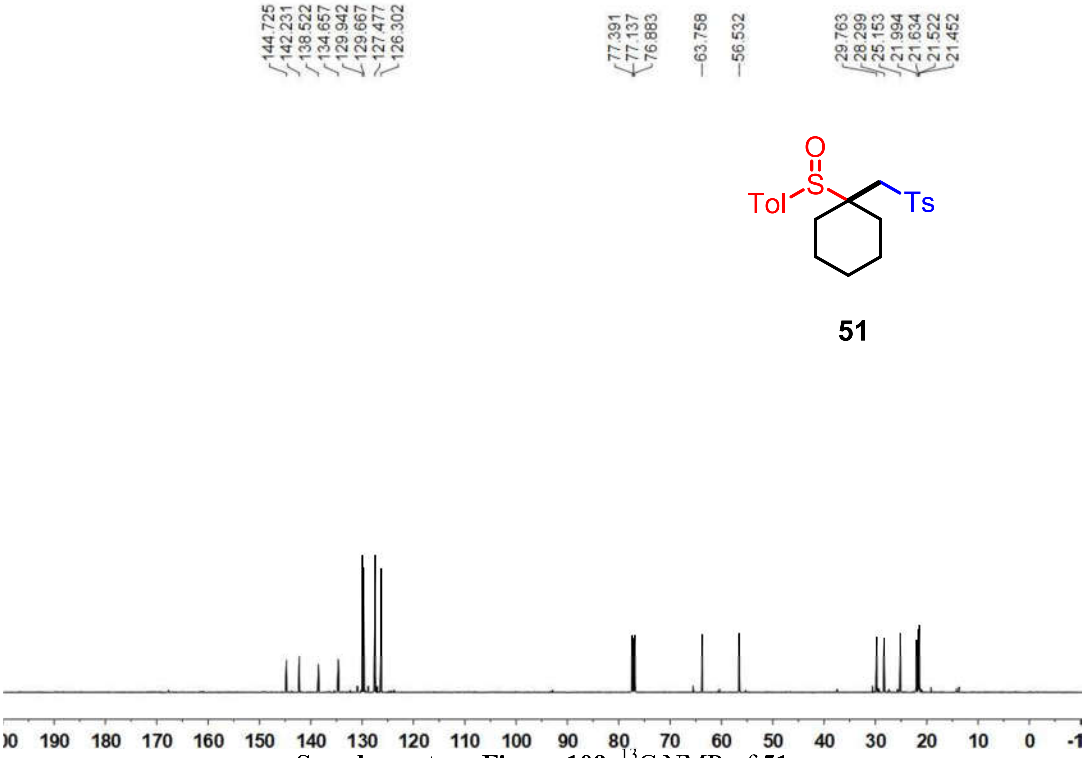

Supplementary Figure 109. ^{13}C NMR of **51**

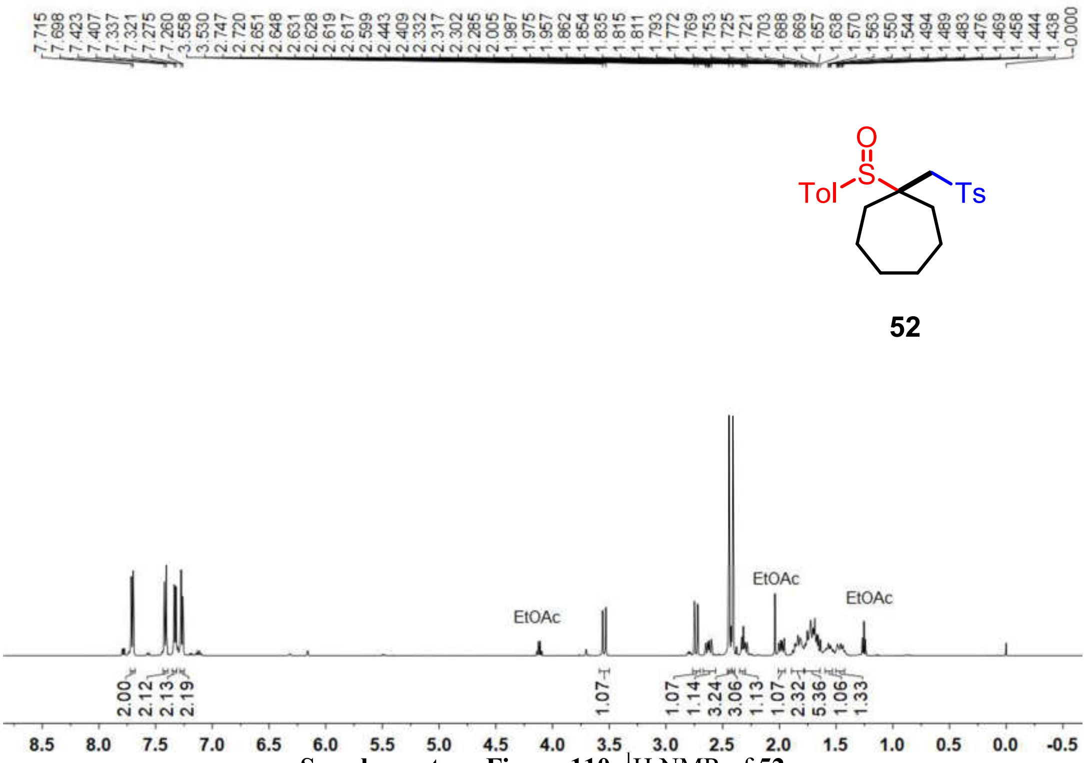

Supplementary Figure 110. ^{1}H NMR of **52**

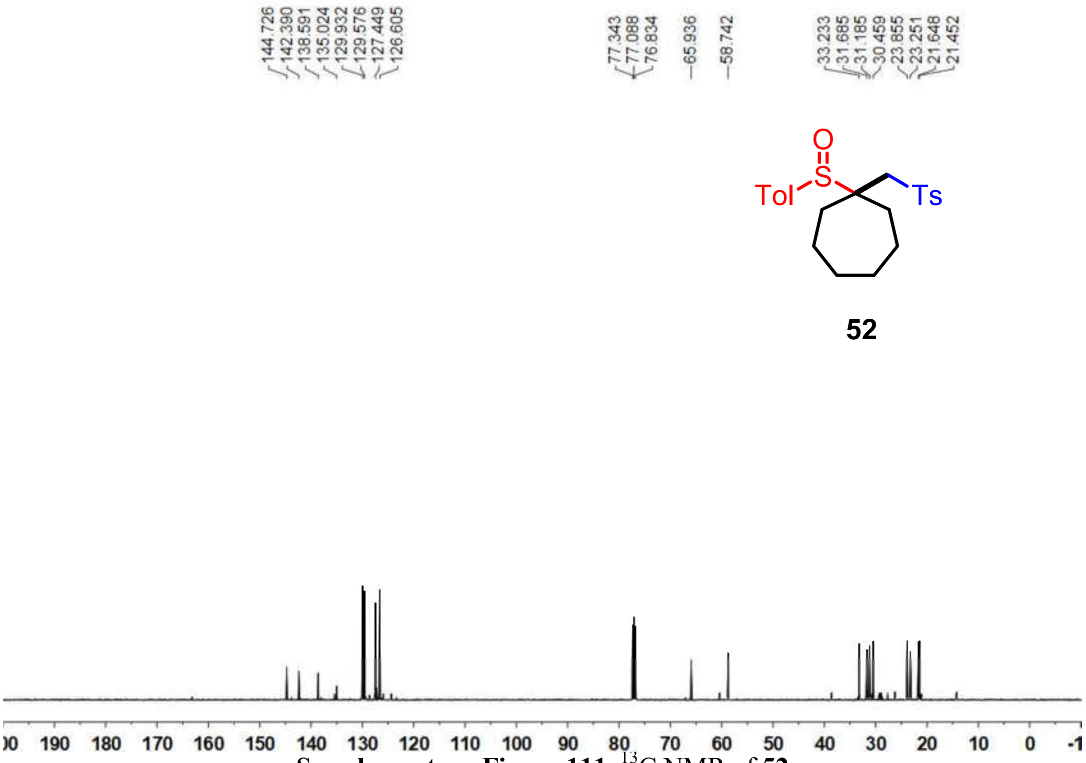

Supplementary Figure 111. ^{13}C NMR of **52**

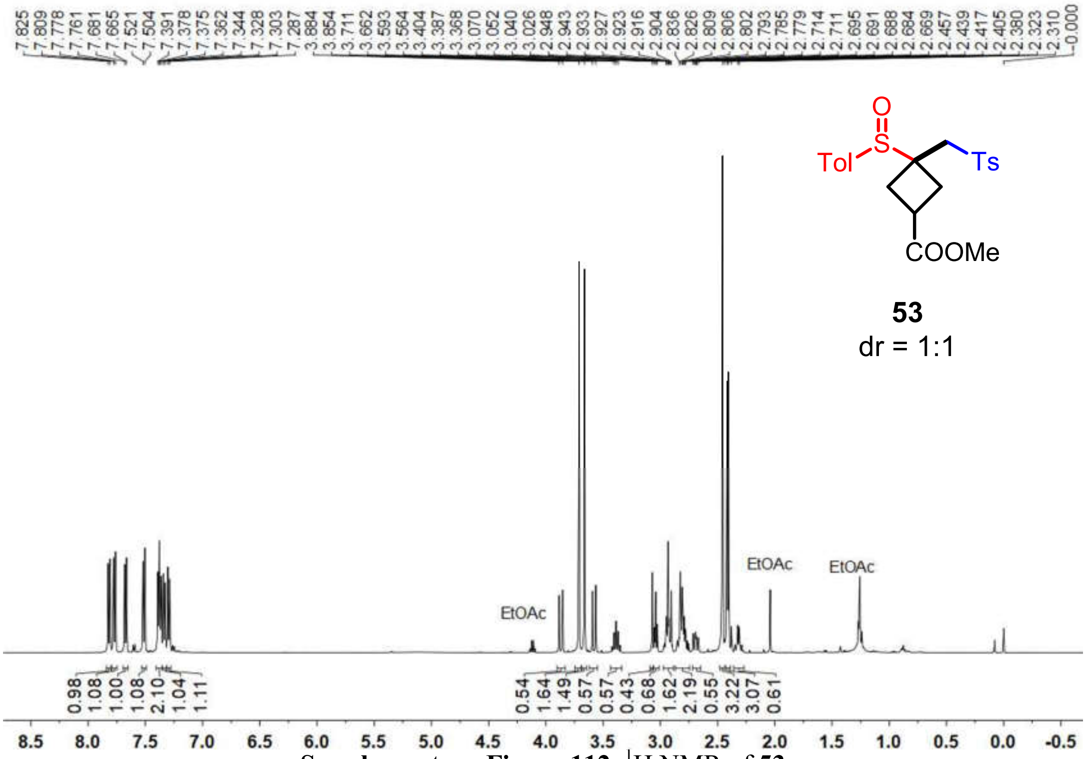

Supplementary Figure 112. ^{1}H NMR of **53**

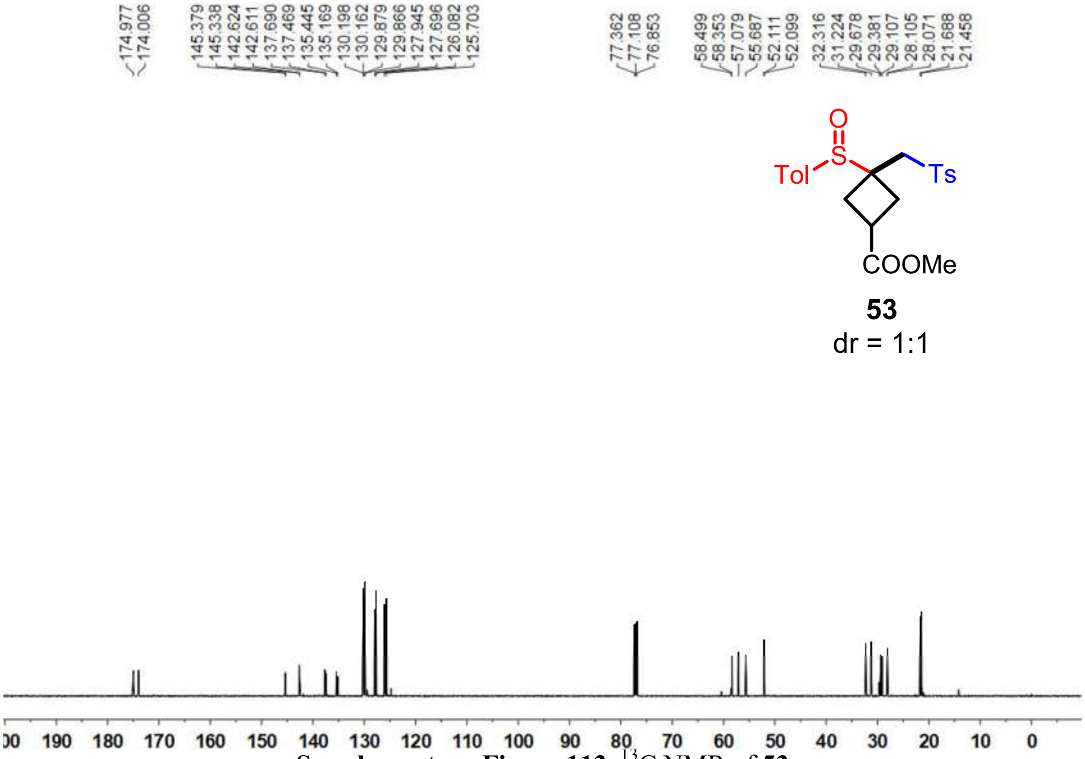

Supplementary Figure 113. ^{13}C NMR of **53**

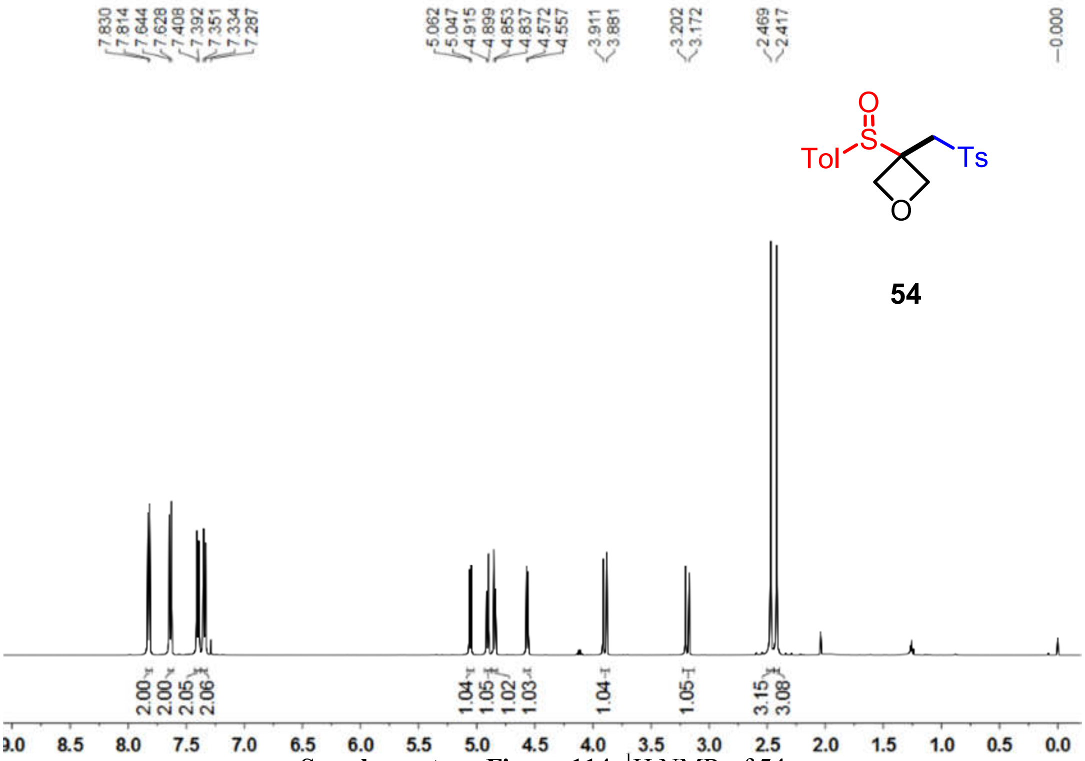

Supplementary Figure 114. ^{1}H NMR of **54**

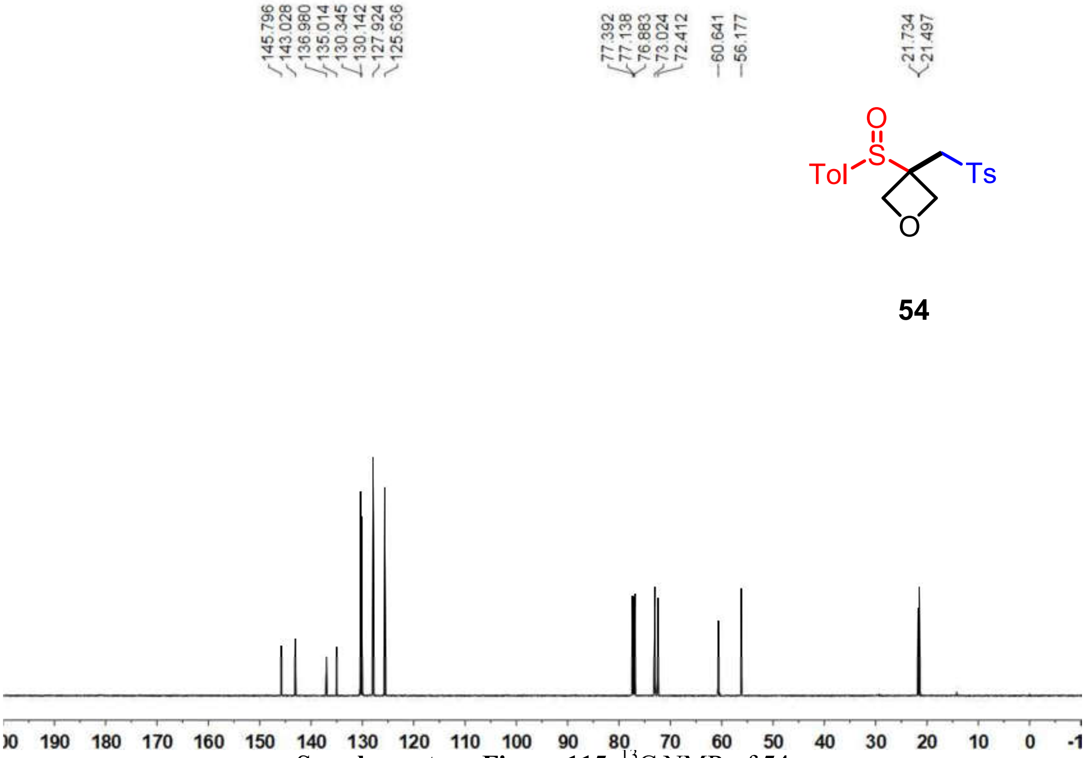

Supplementary Figure 115. ^{13}C NMR of **54**

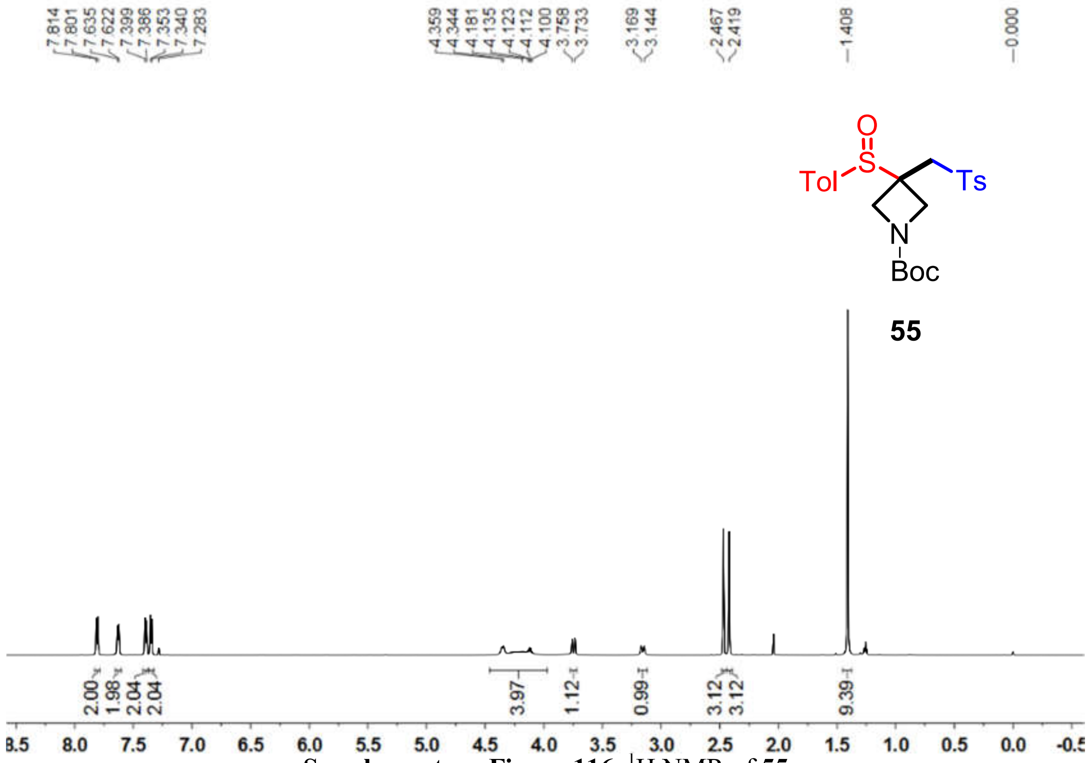

Supplementary Figure 116. ^{1}H NMR of **55**

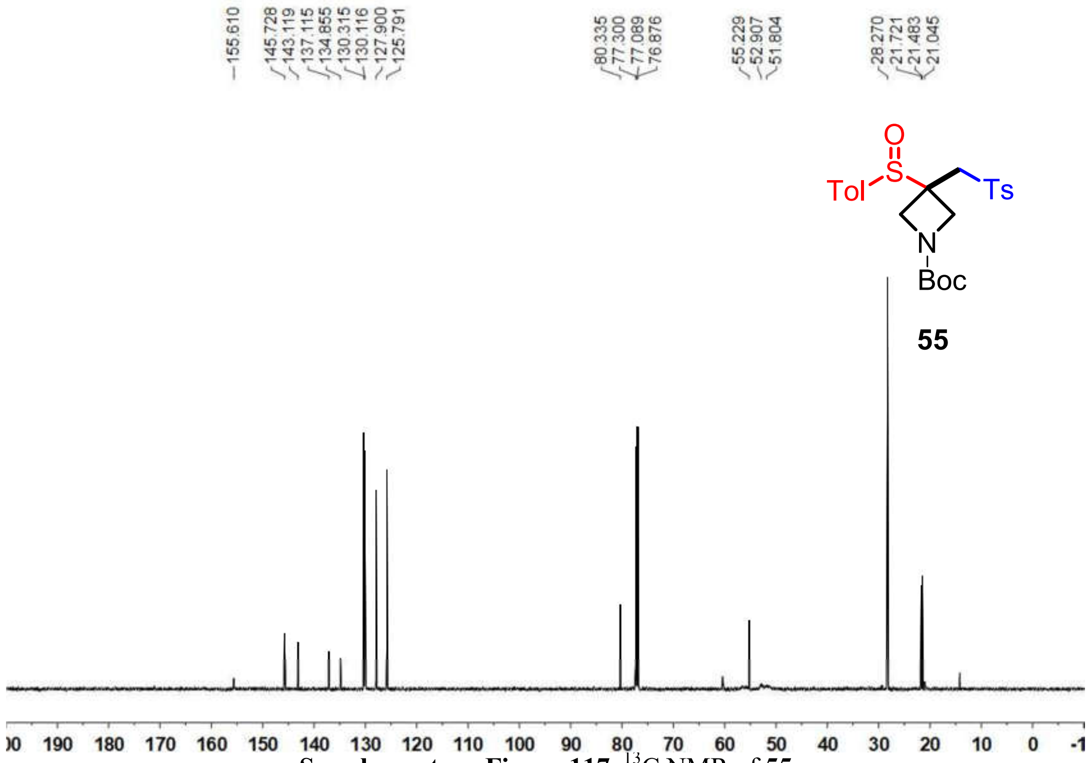

Supplementary Figure 117. ^{13}C NMR of **55**

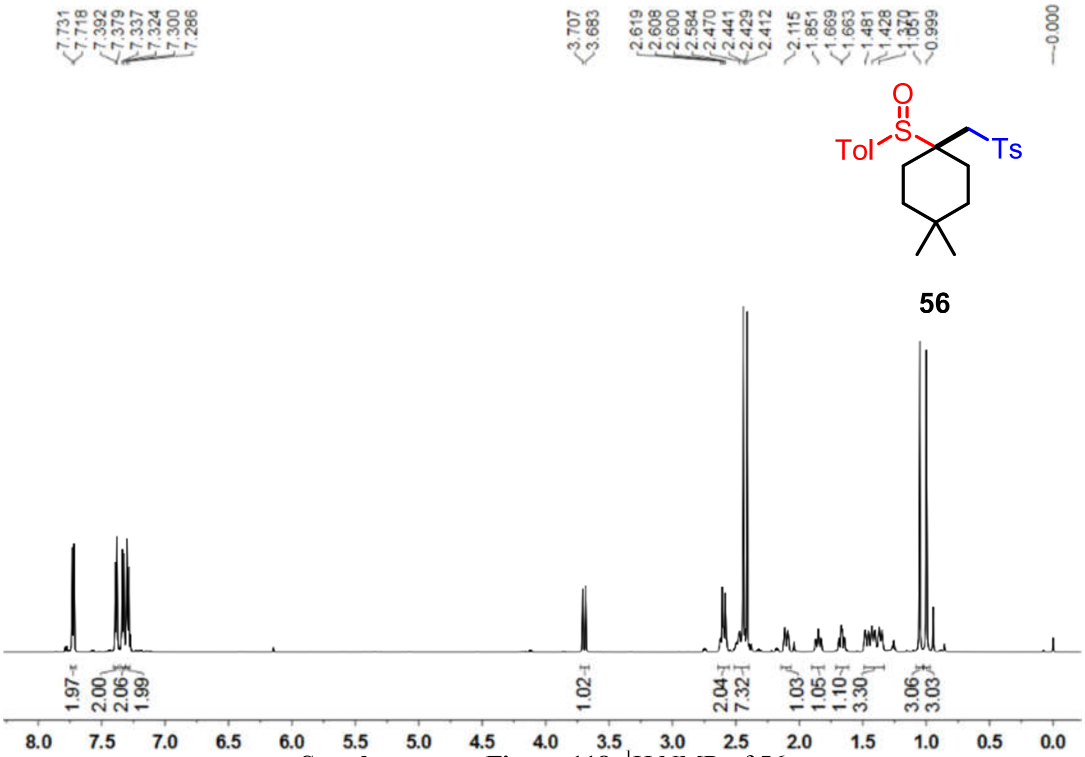

Supplementary Figure 118. ^{1}H NMR of **56**

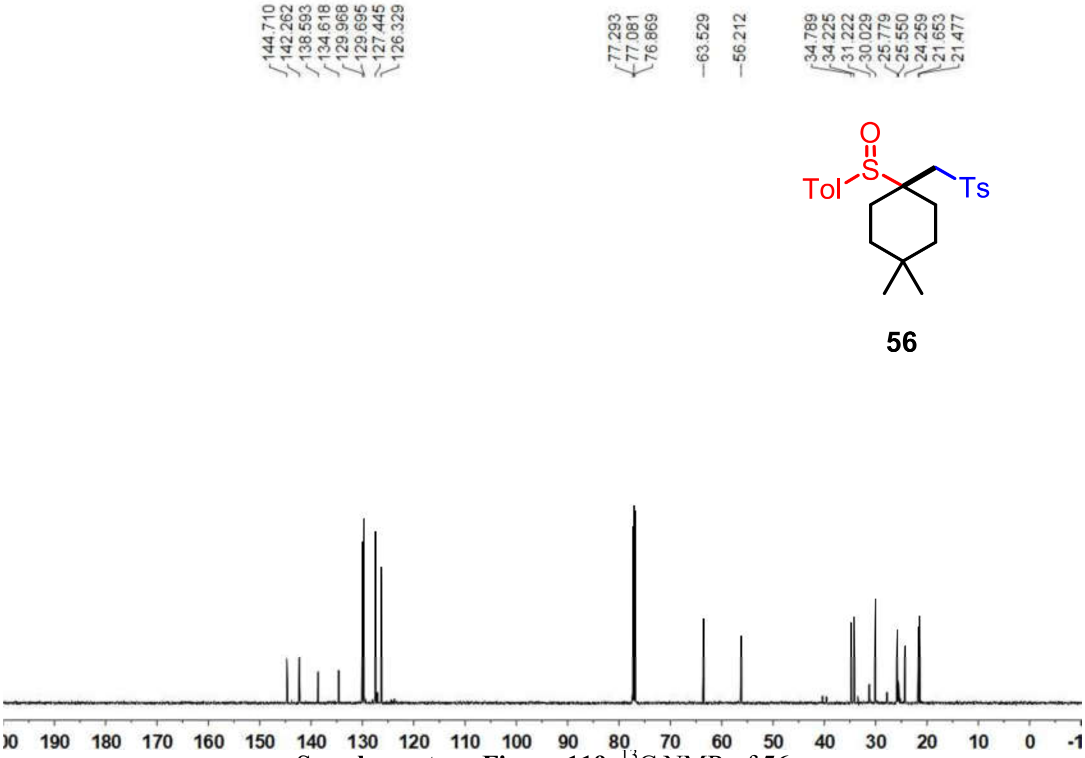

Supplementary Figure 119. ^{13}C NMR of **56**

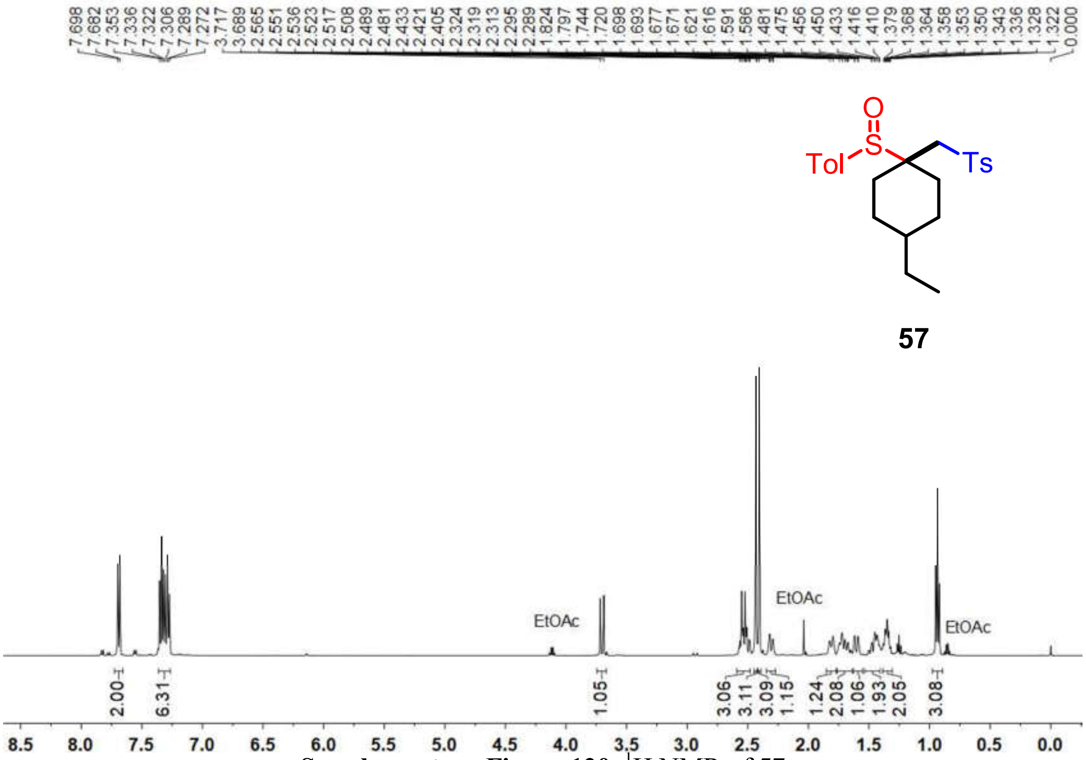

Supplementary Figure 120. ^{1}H NMR of **57**

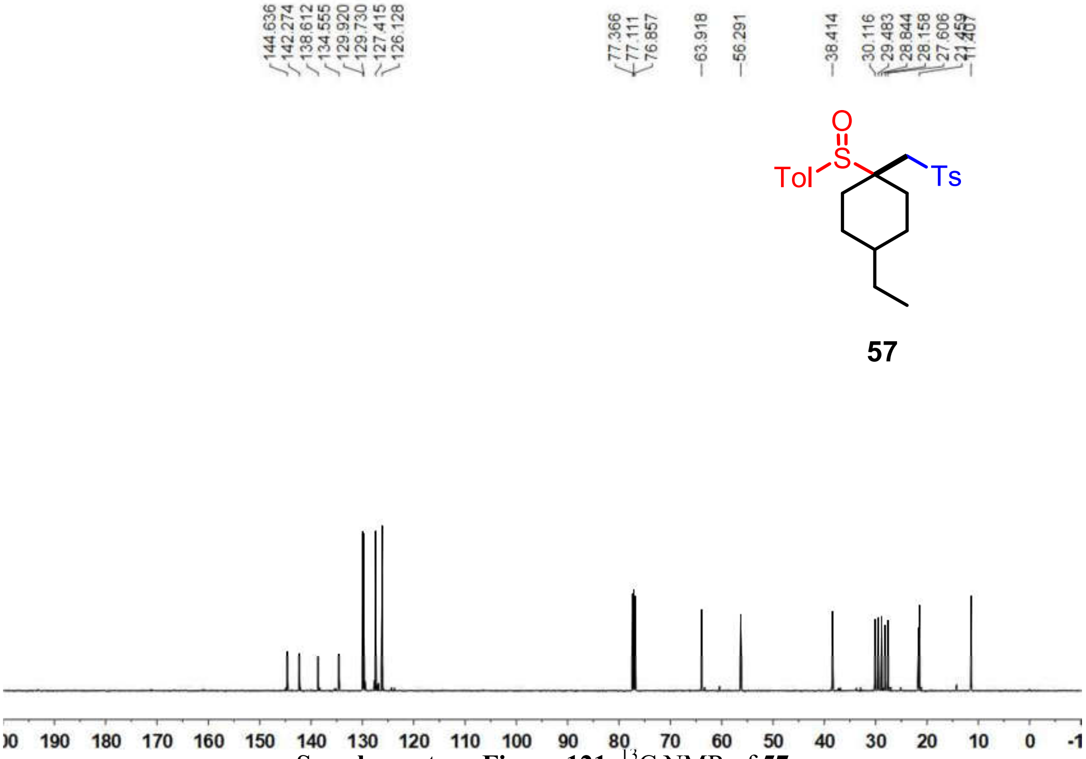

Supplementary Figure 121. ^{13}C NMR of **57**

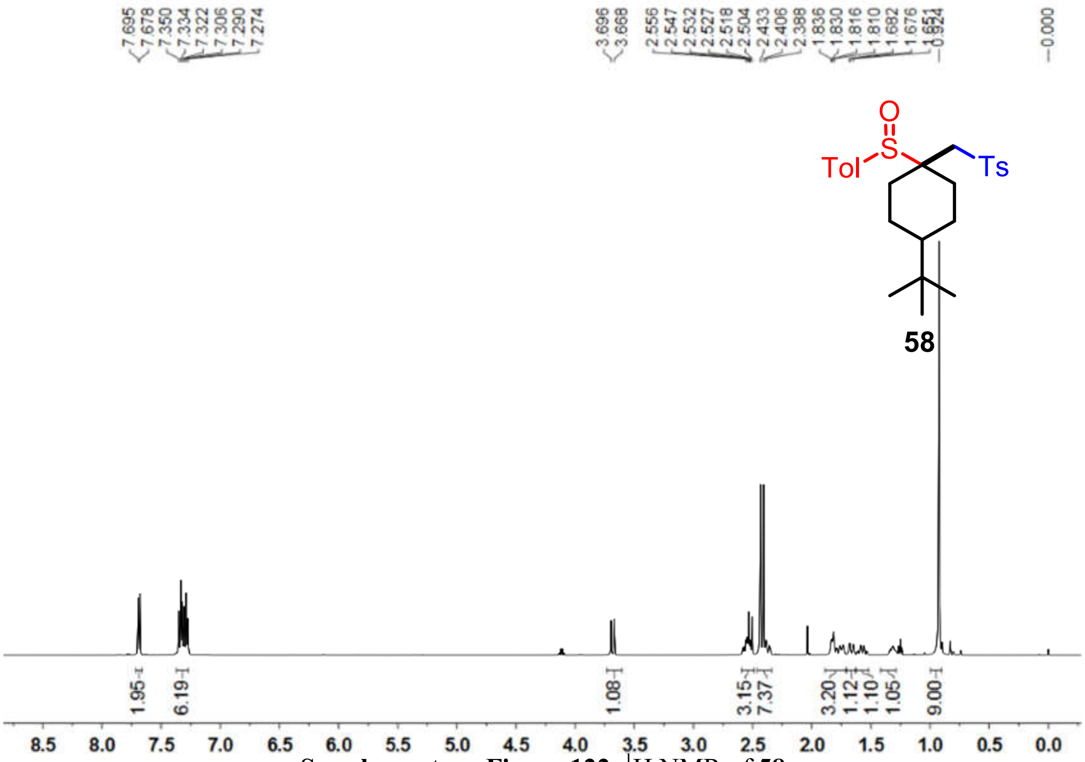

Supplementary Figure 122. ^{1}H NMR of **58**

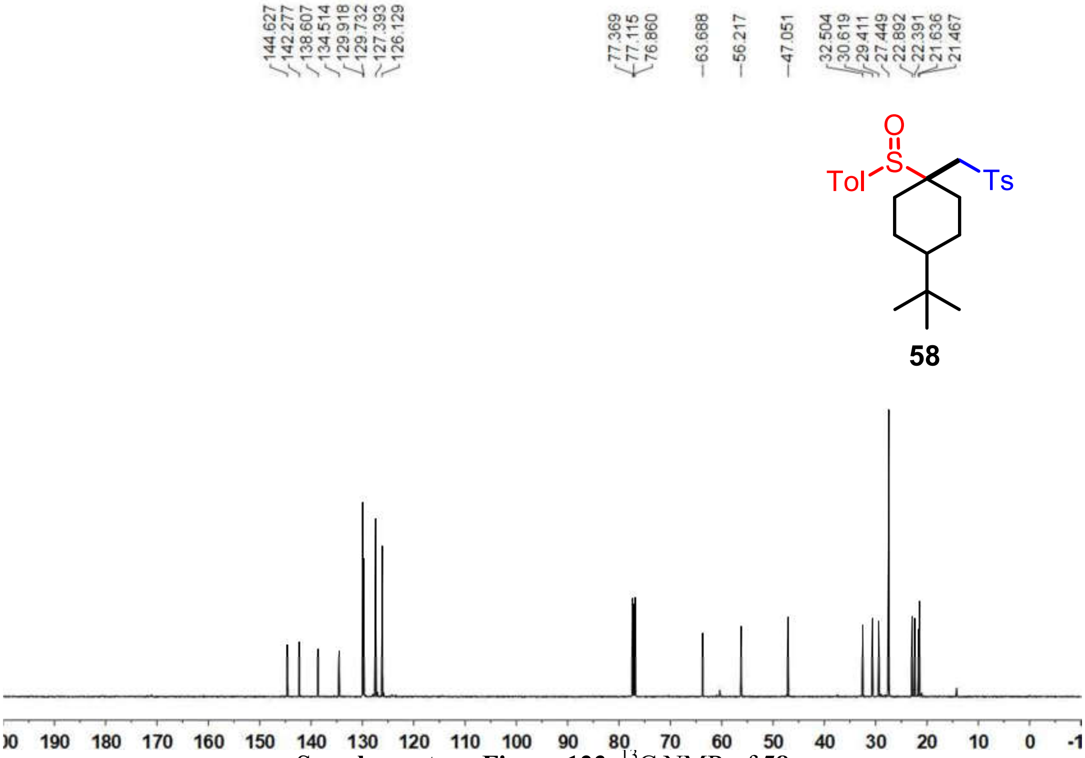

Supplementary Figure 123. ^{13}C NMR of **58**

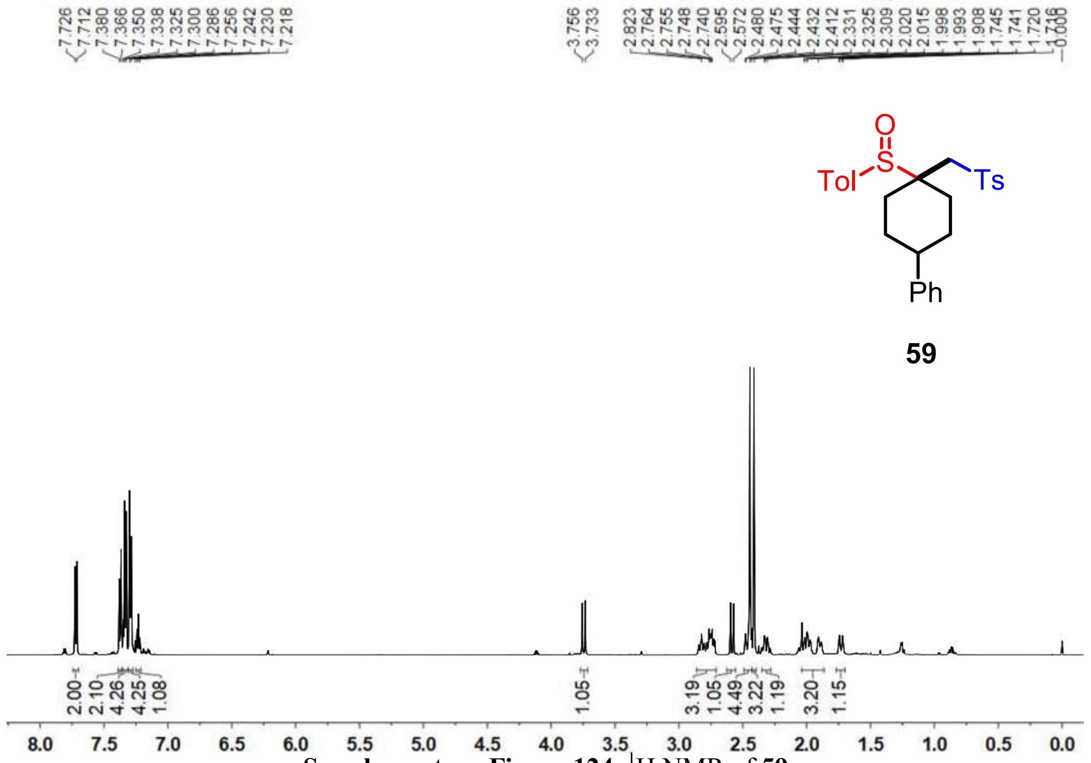

Supplementary Figure 124. ^{1}H NMR of **59**

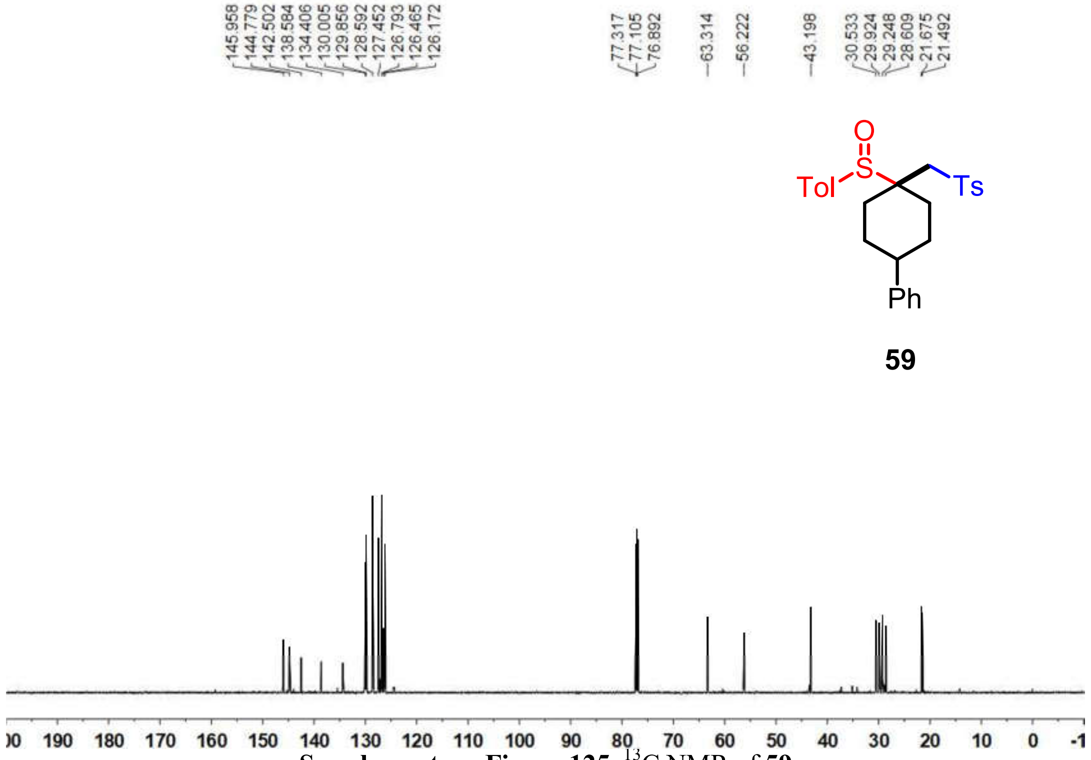

Supplementary Figure 125. ^{13}C NMR of **59**

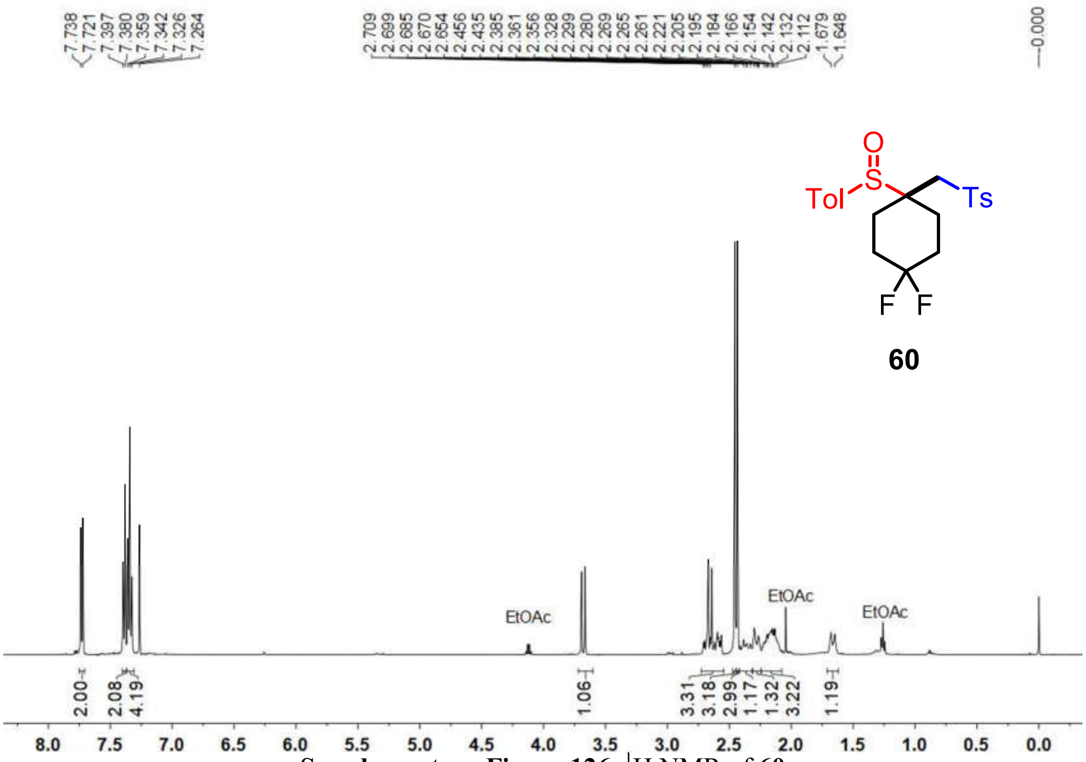

Supplementary Figure 126. ^{1}H NMR of **60**

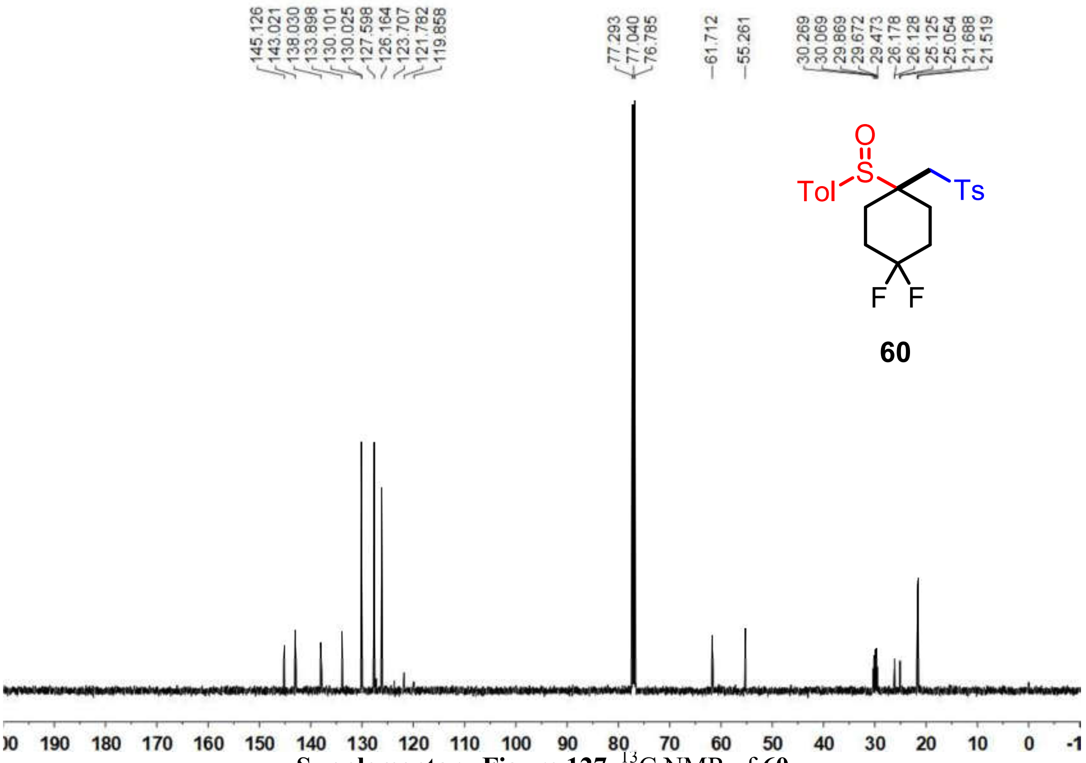

Supplementary Figure 127. ^{13}C NMR of **60**

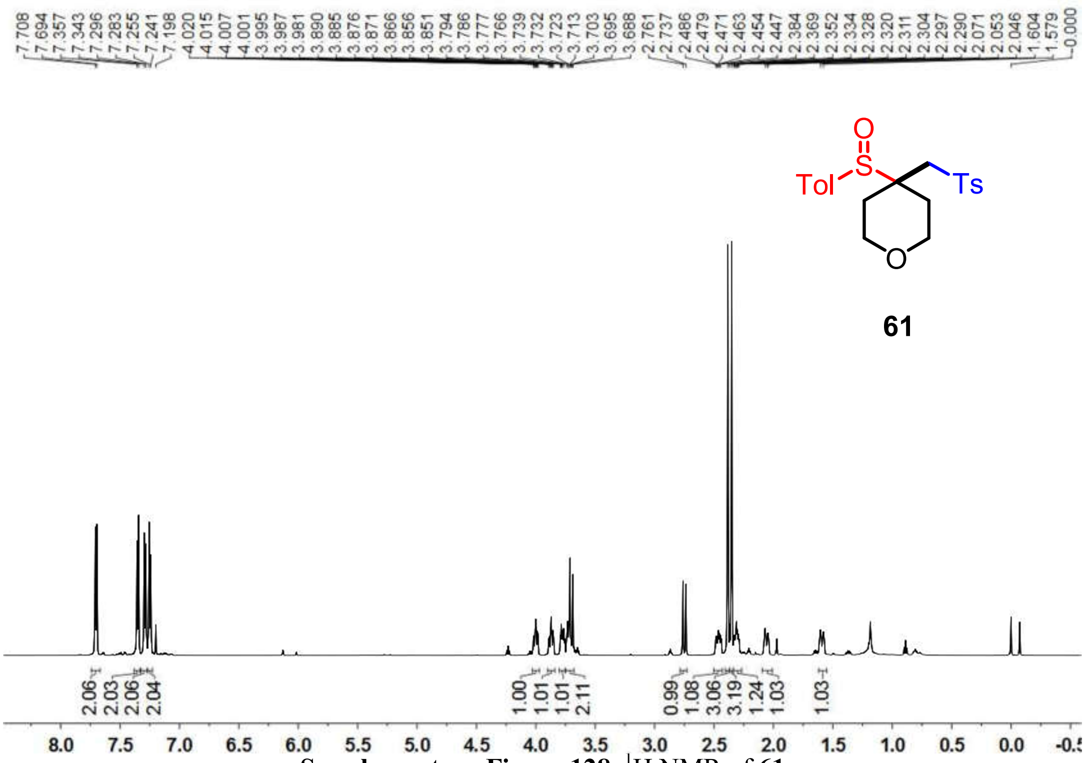

Supplementary Figure 128. ^{1}H NMR of **61**

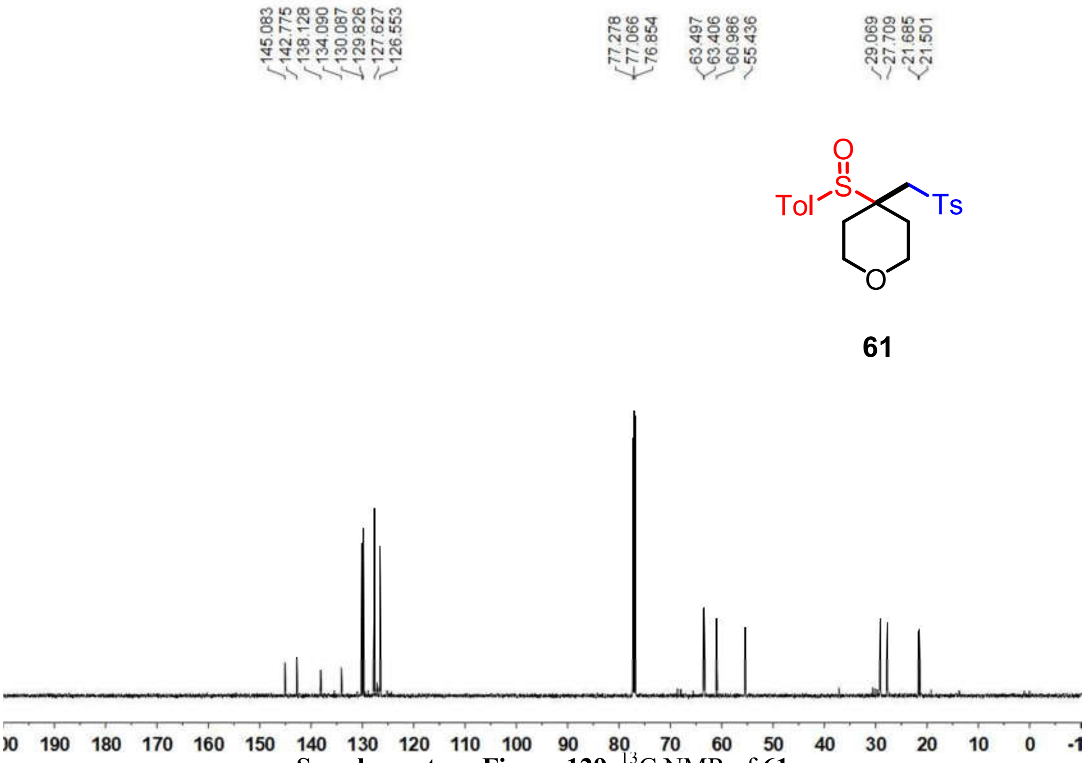

Supplementary Figure 129. ^{13}C NMR of **61**

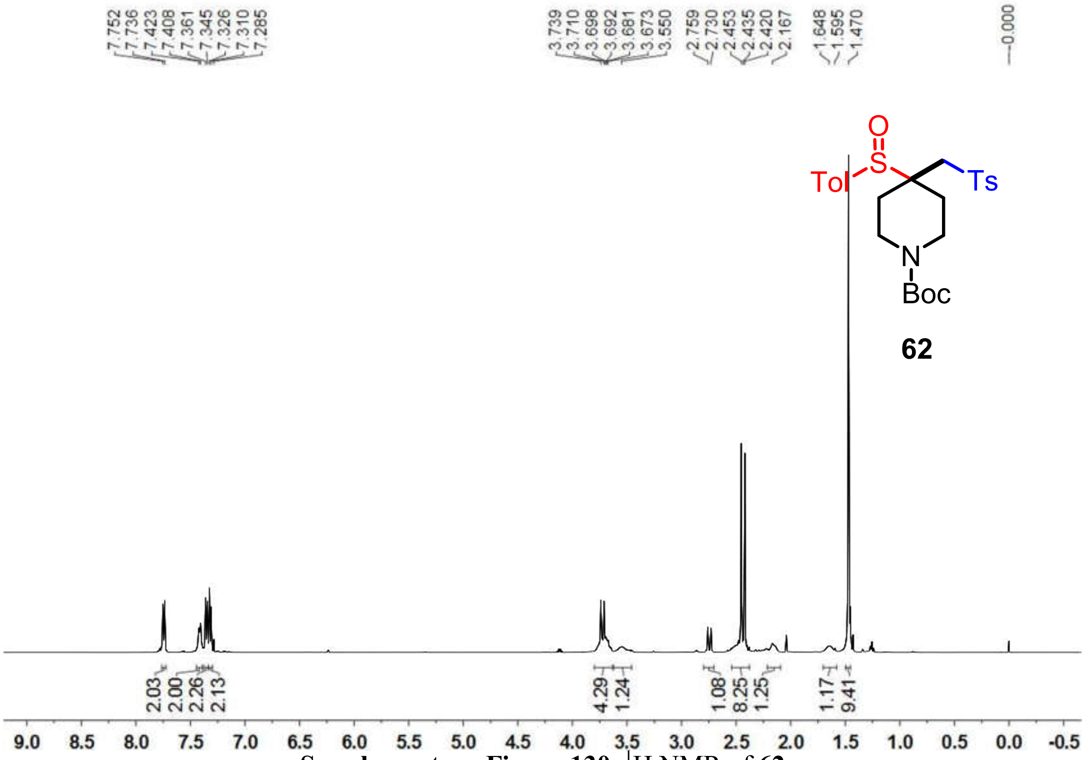

62

Supplementary Figure 130. ^{1}H NMR of **62**

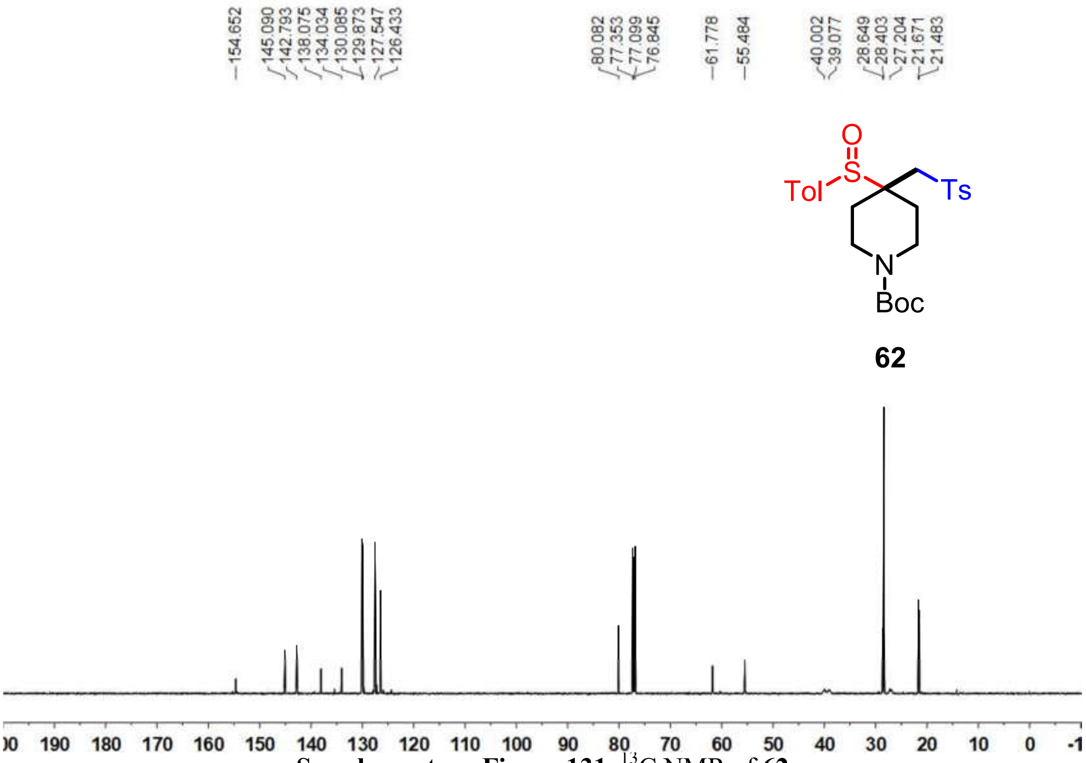

62

Supplementary Figure 131. ^{13}C NMR of **62**

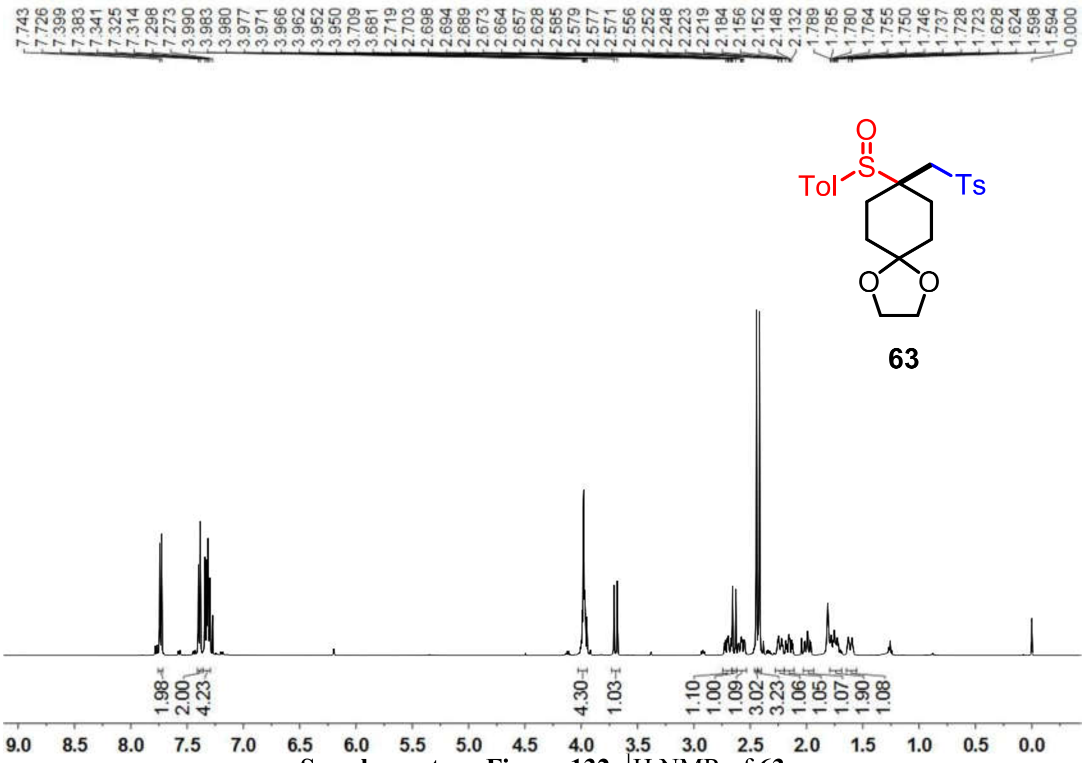

Supplementary Figure 132. ^{1}H NMR of **63**

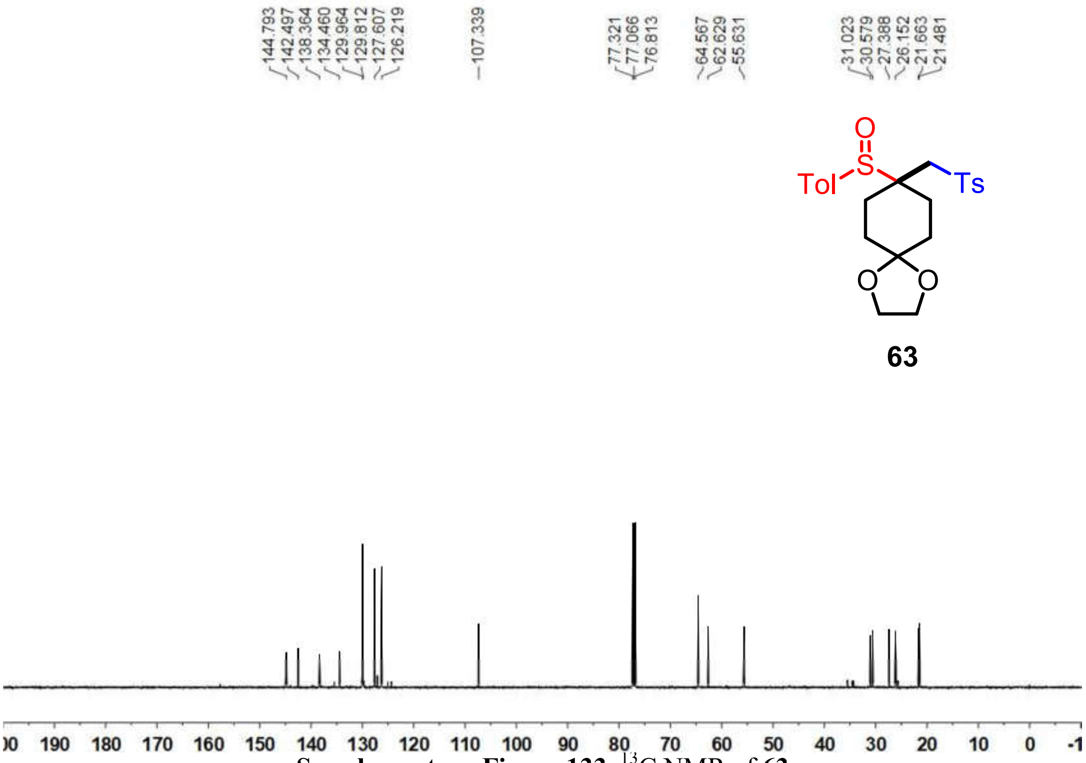

Supplementary Figure 133. ^{13}C NMR of **63**

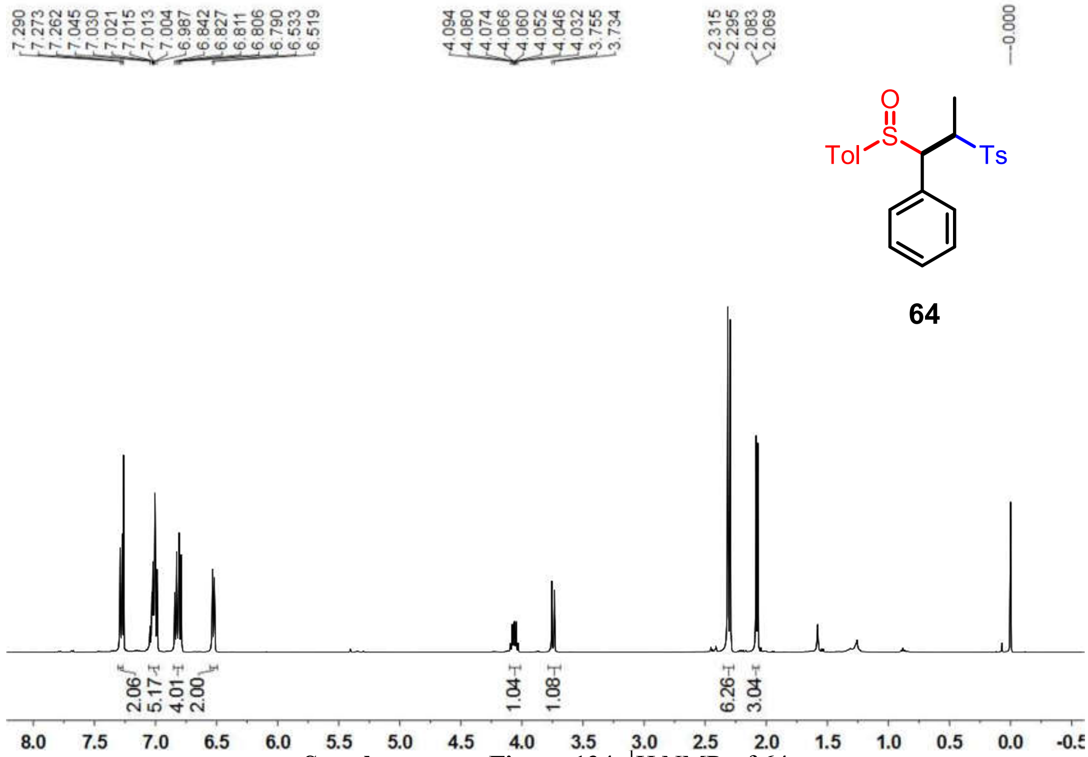

Supplementary Figure 134. ^{1}H NMR of **64**

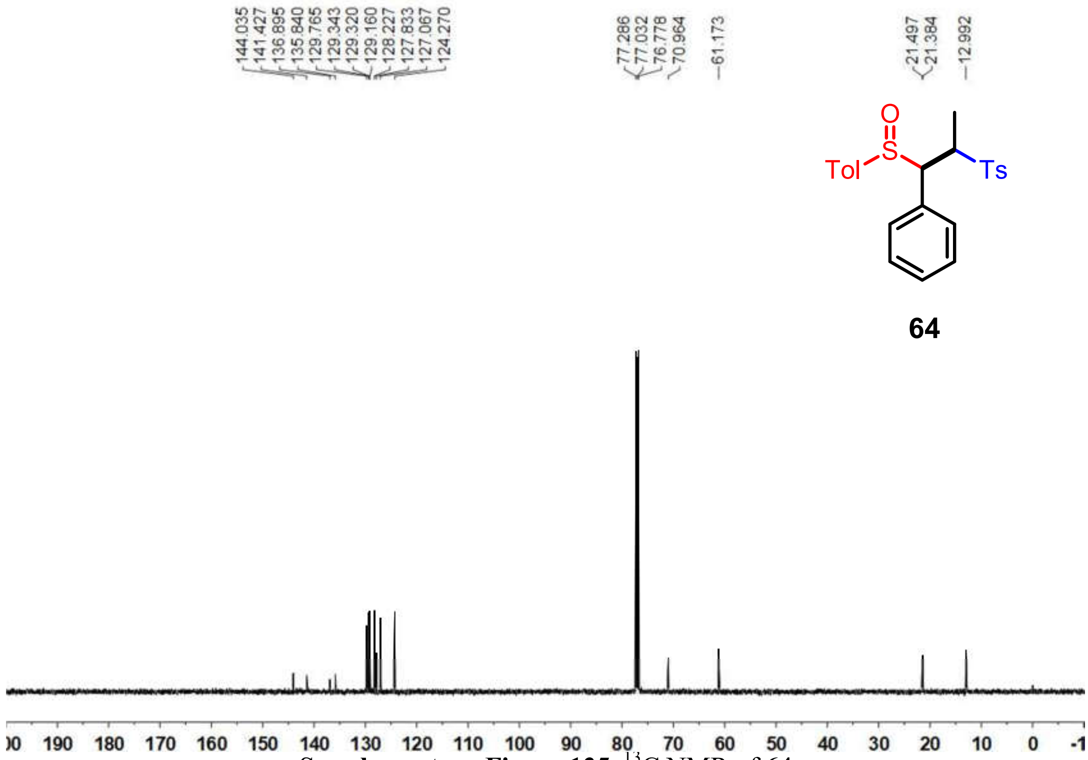

Supplementary Figure 135. ^{13}C NMR of **64**

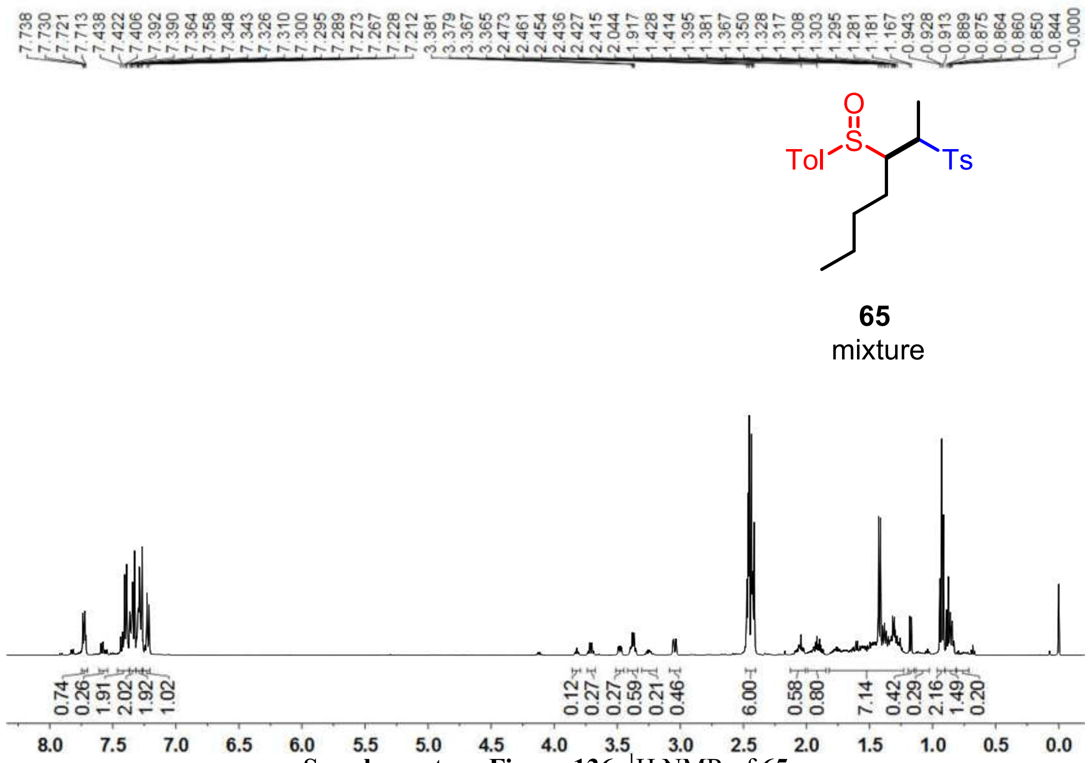

Supplementary Figure 136. ^{1}H NMR of **65**

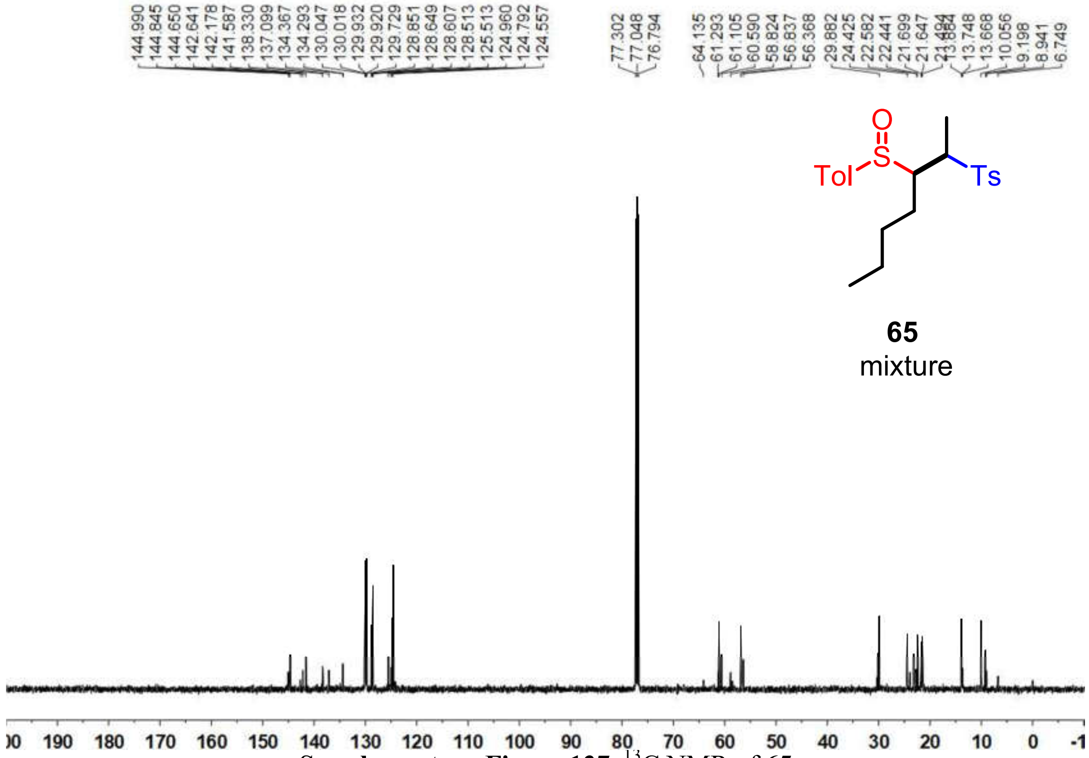

Supplementary Figure 137. ^{13}C NMR of **65**

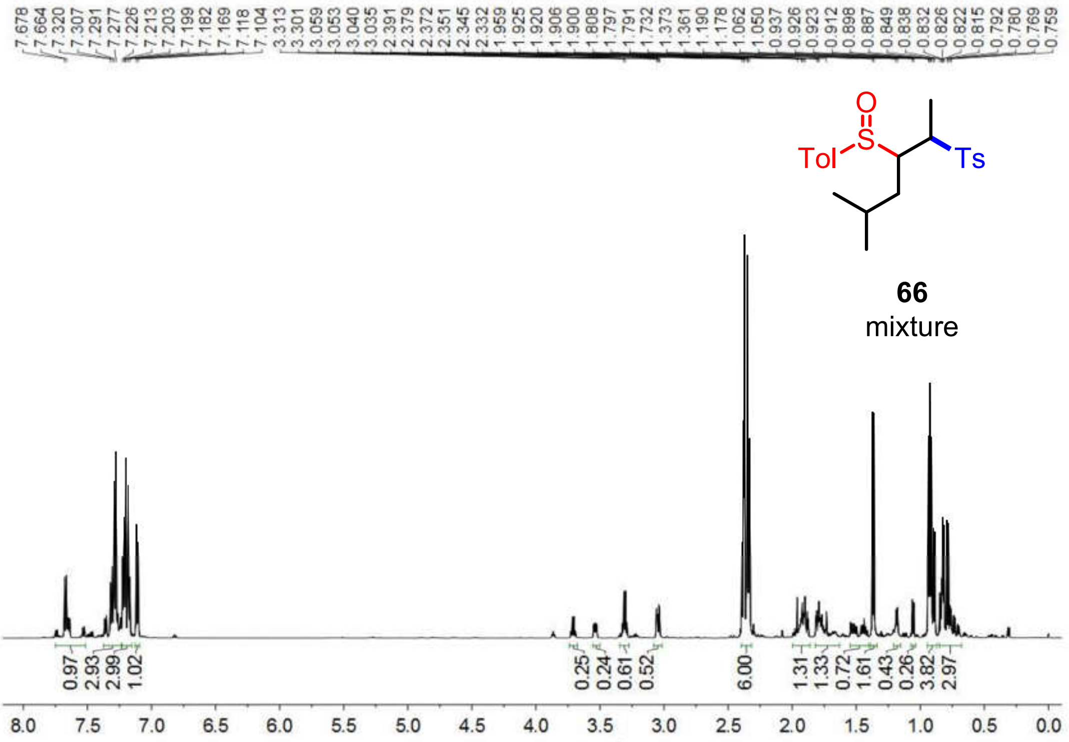

Supplementary Figure 138. ^{1}H NMR of **66**

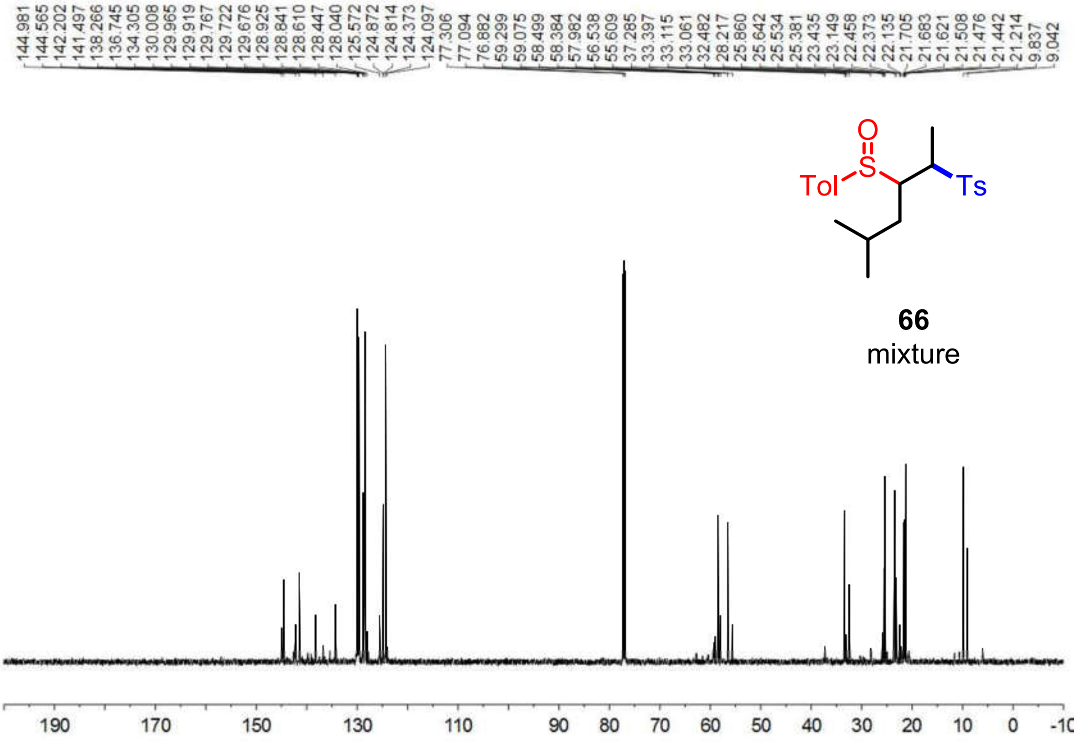

Supplementary Figure 139. ^{13}C NMR of **66**

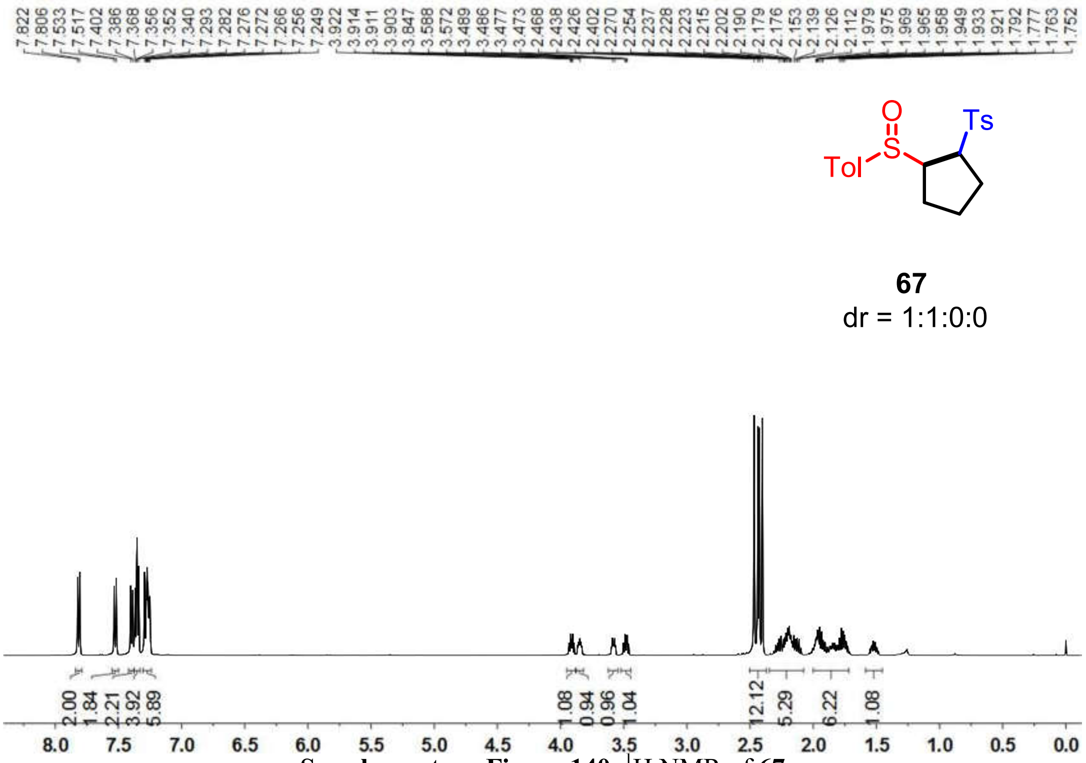

Supplementary Figure 140. ^{1}H NMR of **67**

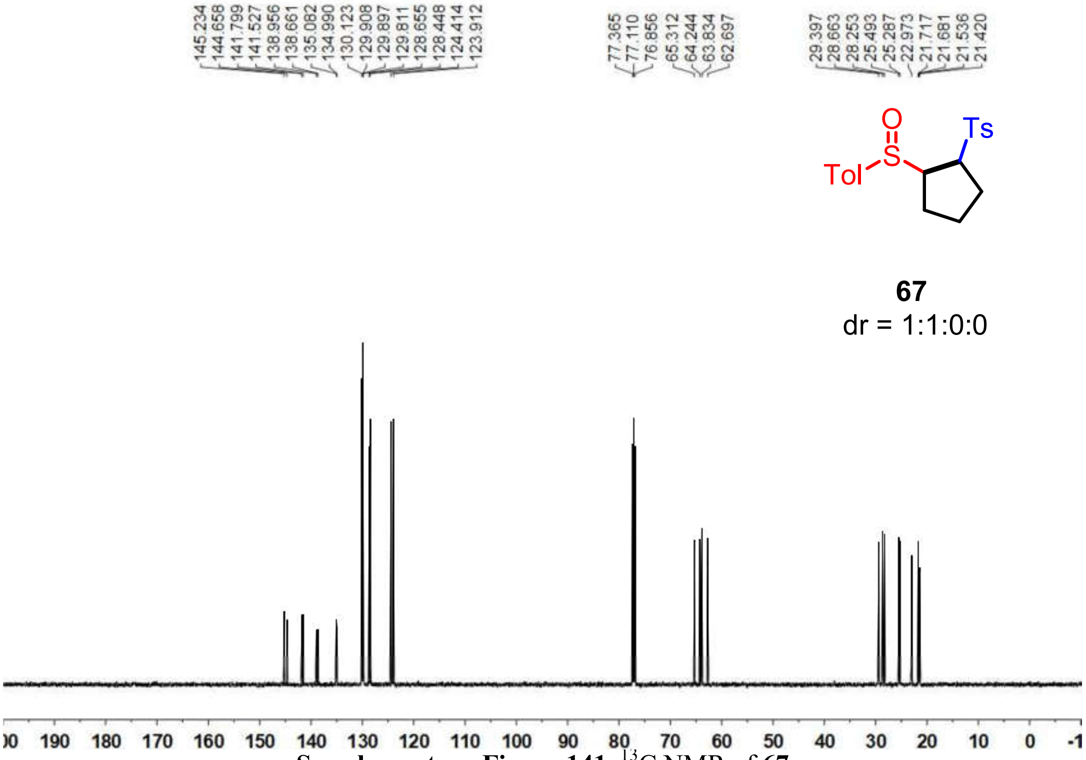

Supplementary Figure 141. ^{13}C NMR of **67**

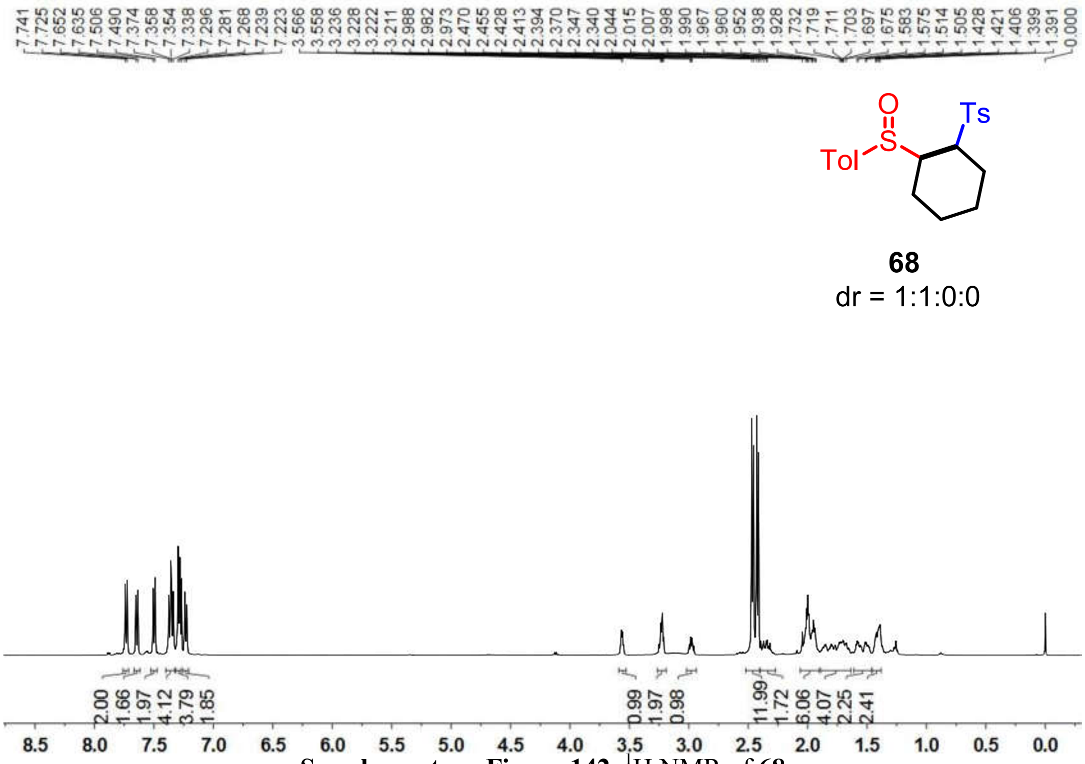

Supplementary Figure 142. ^{1}H NMR of **68**

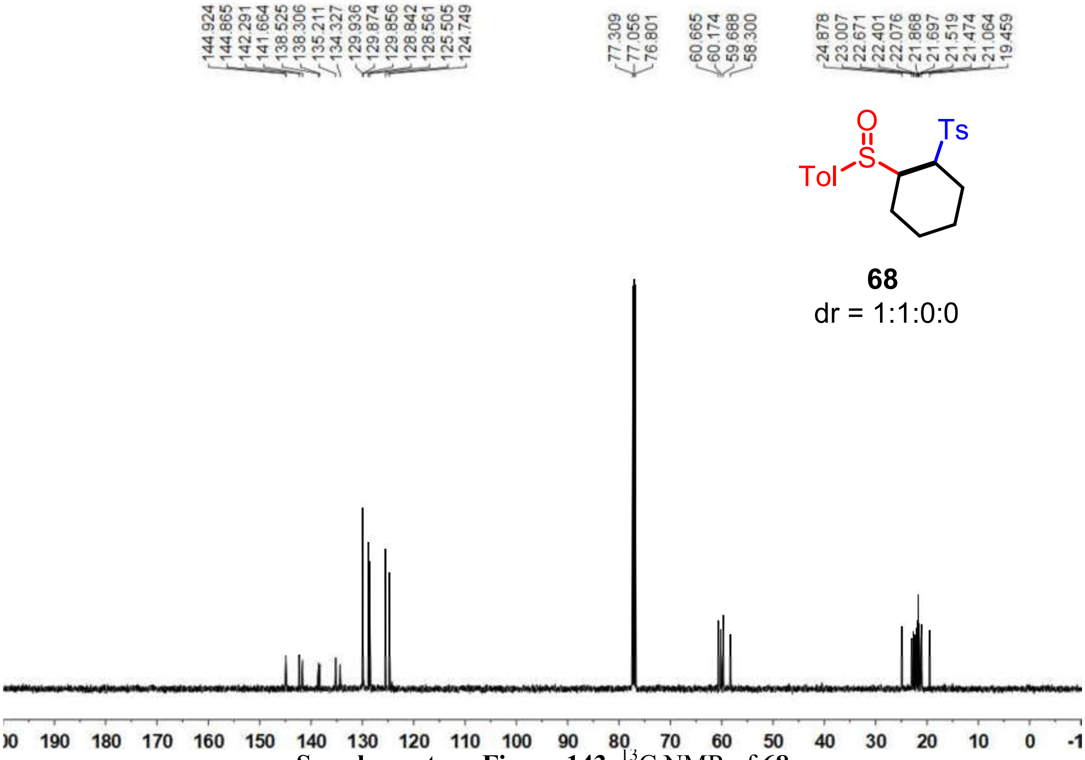

Supplementary Figure 143. ^{13}C NMR of **68**

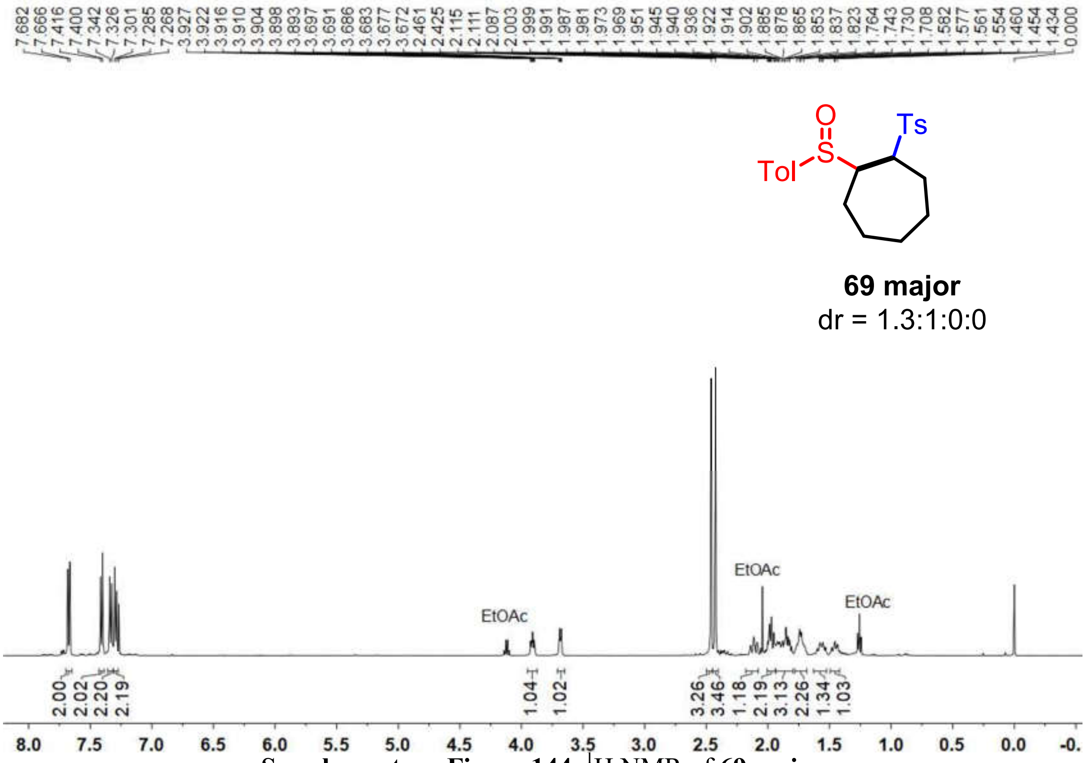

Supplementary Figure 144. ^{1}H NMR of **69 major**

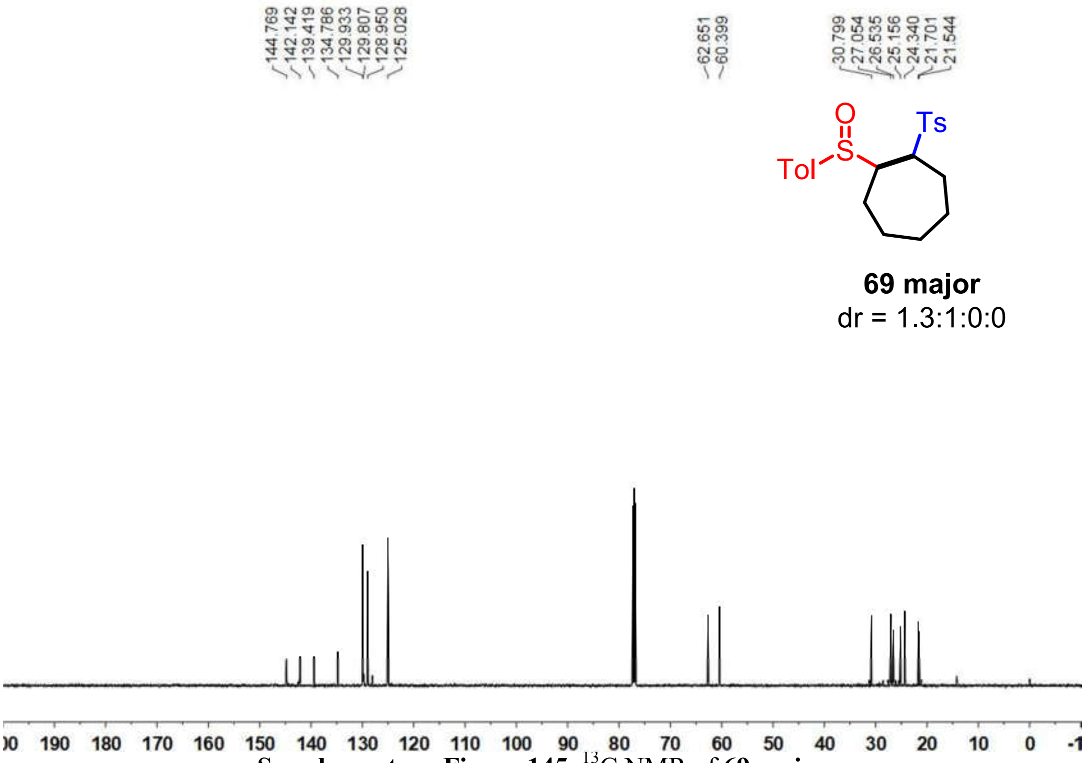

Supplementary Figure 145. ^{13}C NMR of **69 major**

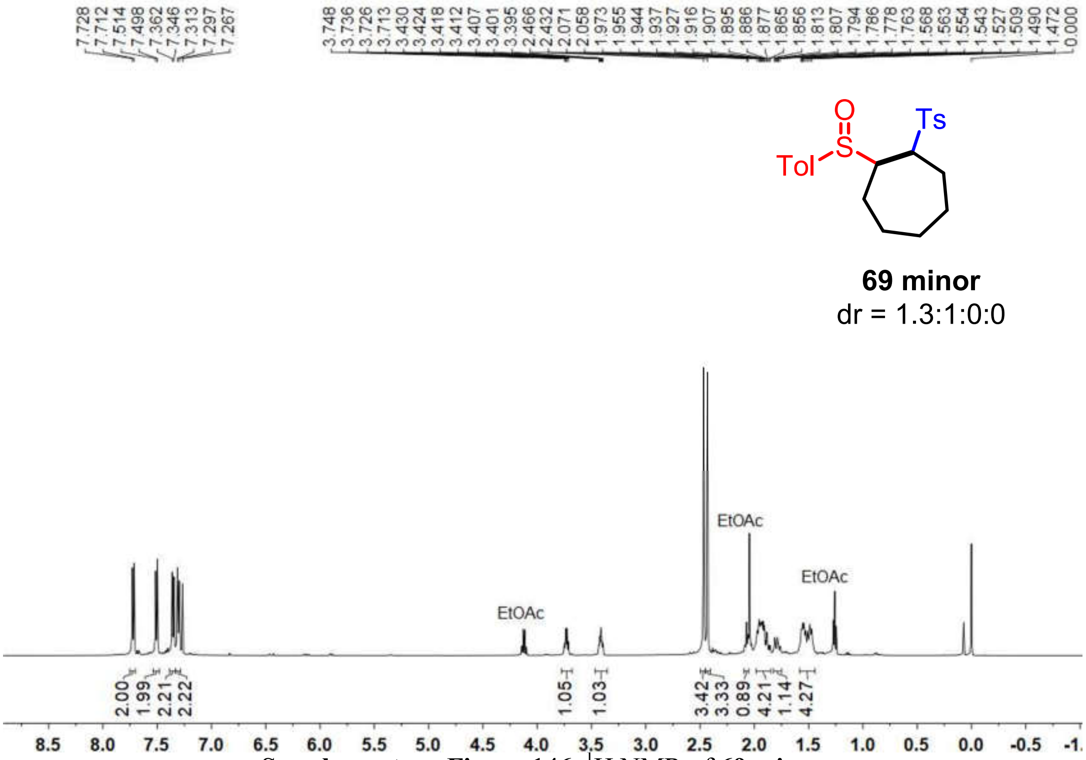

Supplementary Figure 146. ^{1}H NMR of **69 minor**

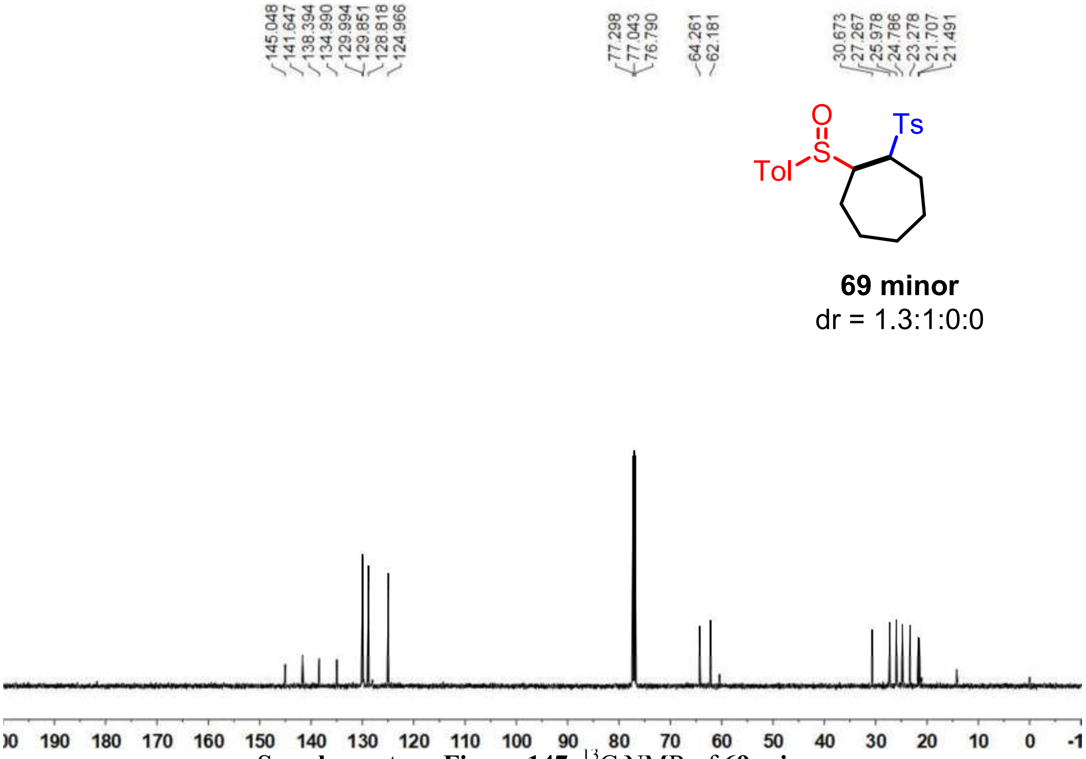

Supplementary Figure 147. ^{13}C NMR of **69 minor**

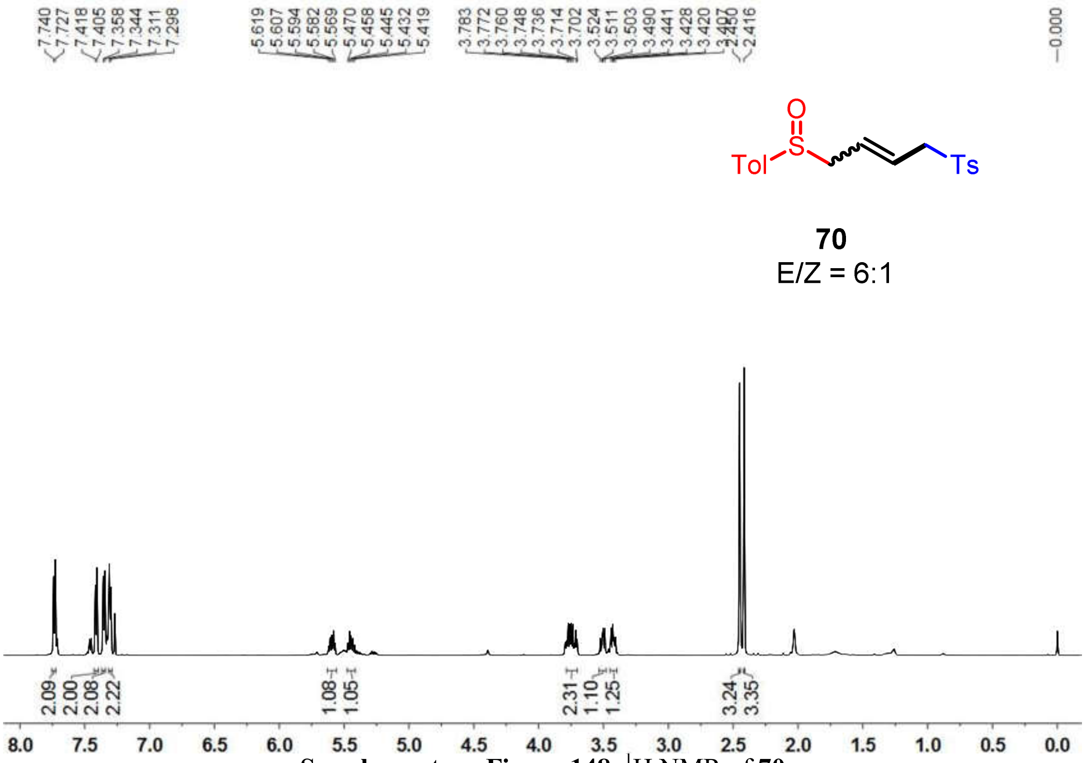

Supplementary Figure 148. ^{1}H NMR of **70**

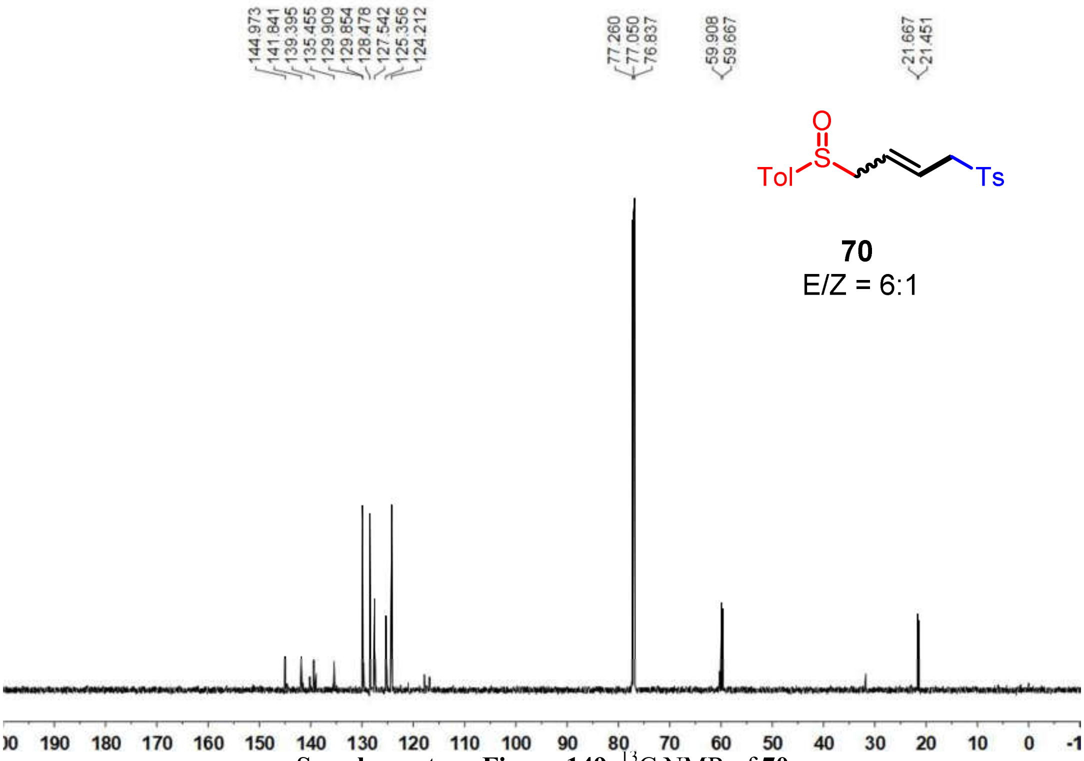

Supplementary Figure 149. ^{13}C NMR of **70**

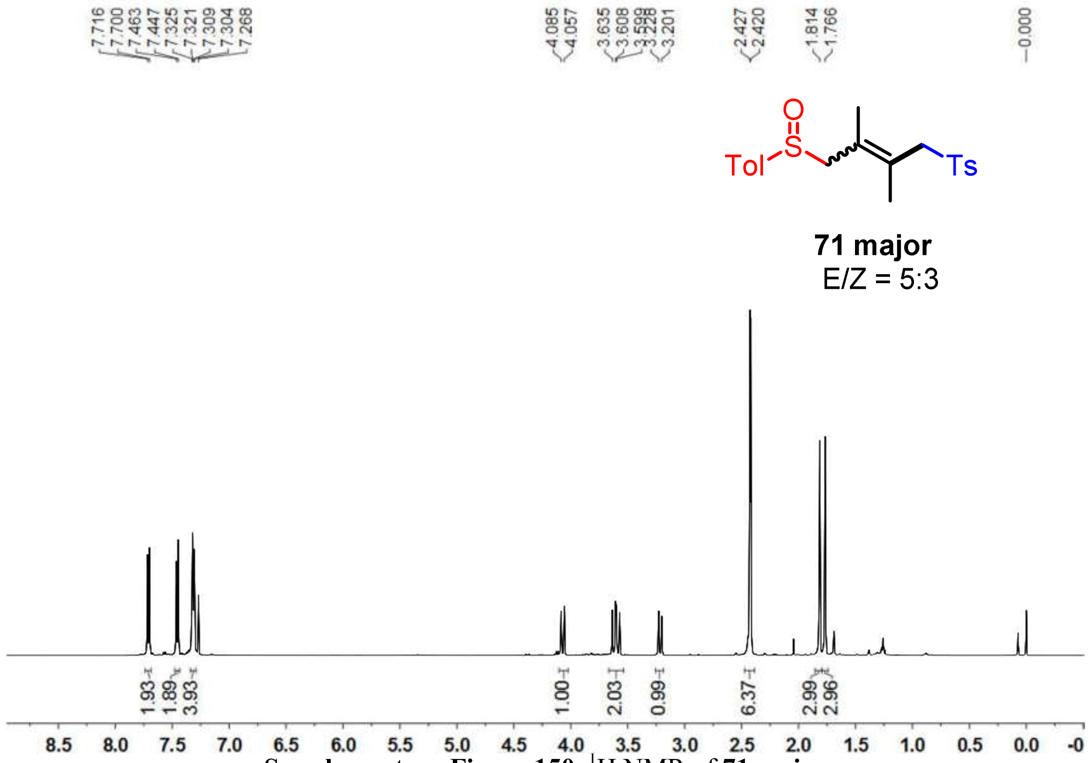

Supplementary Figure 150. ^{1}H NMR of **71 major**

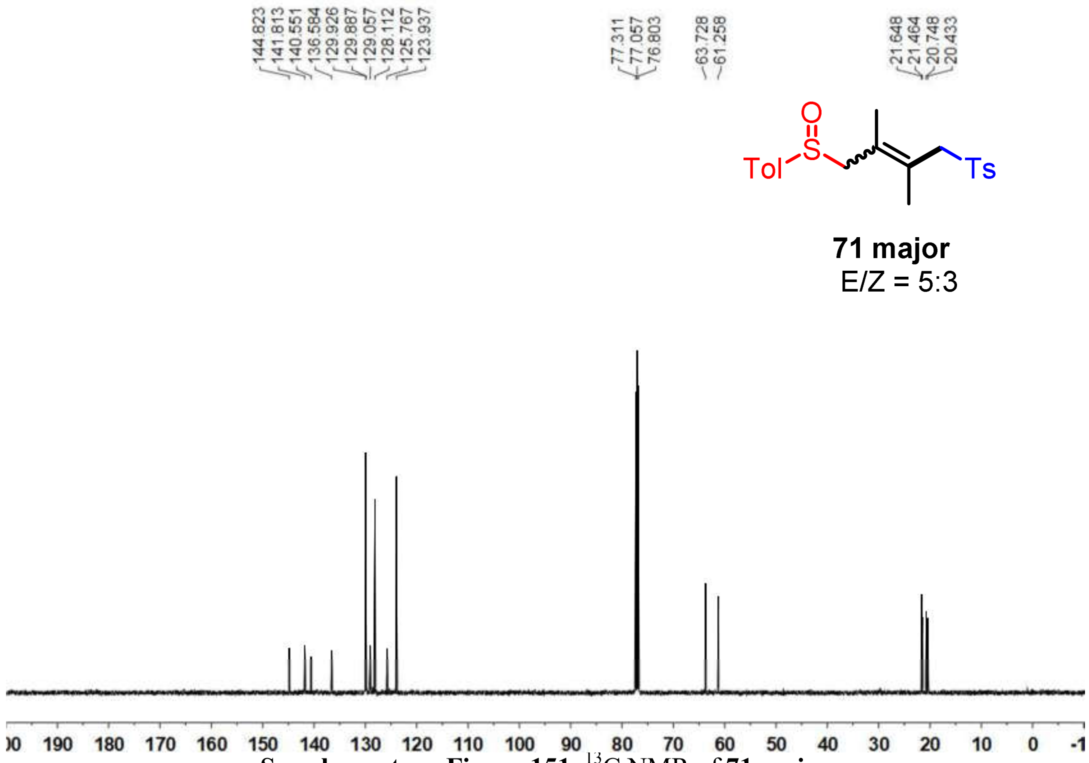

Supplementary Figure 151. ^{13}C NMR of **71 major**

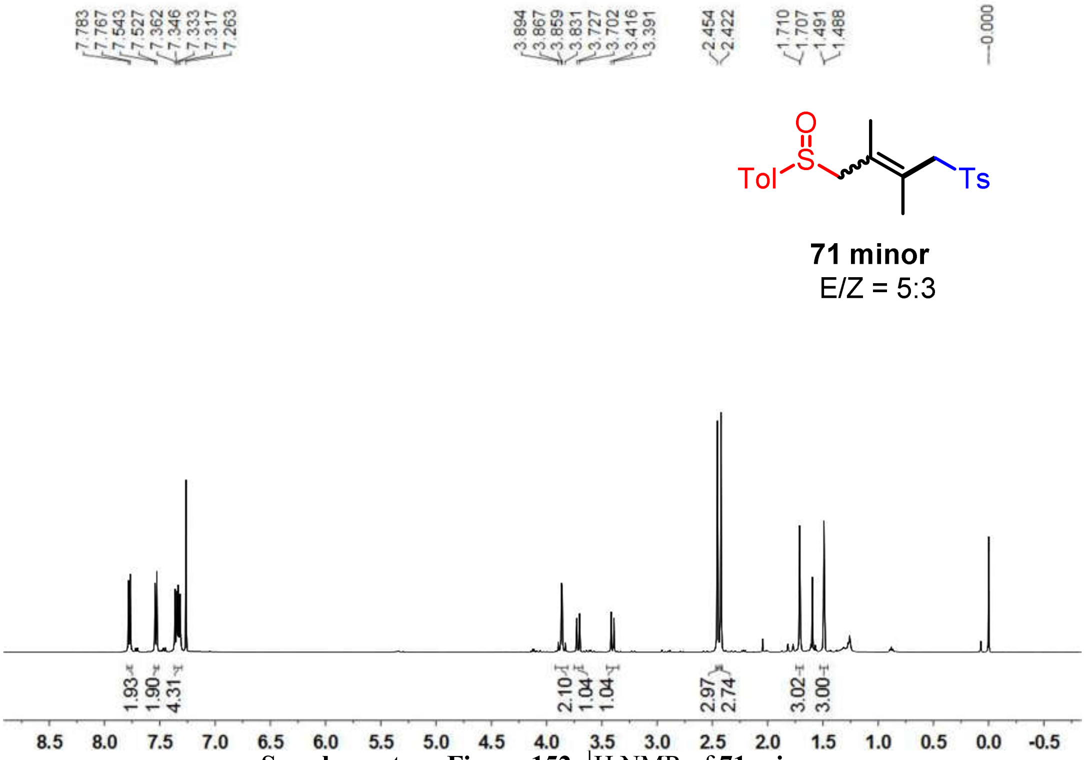

Supplementary Figure 152. ^{1}H NMR of **71 minor**

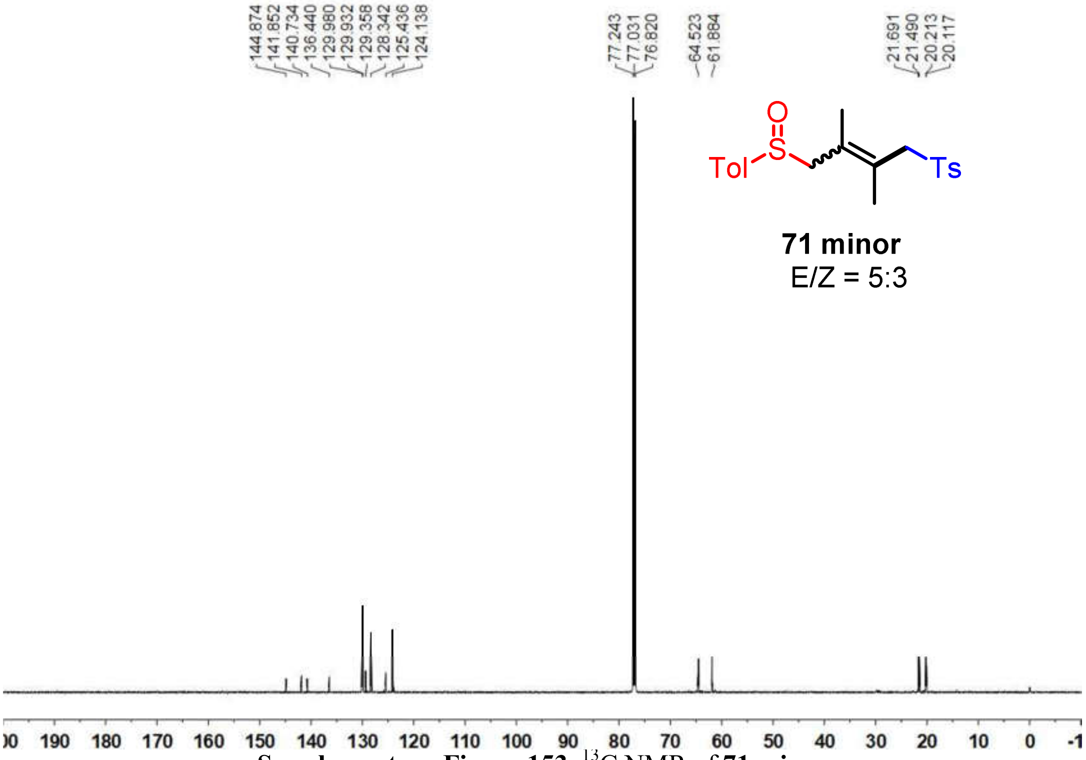

Supplementary Figure 153. ^{13}C NMR of **71 minor**

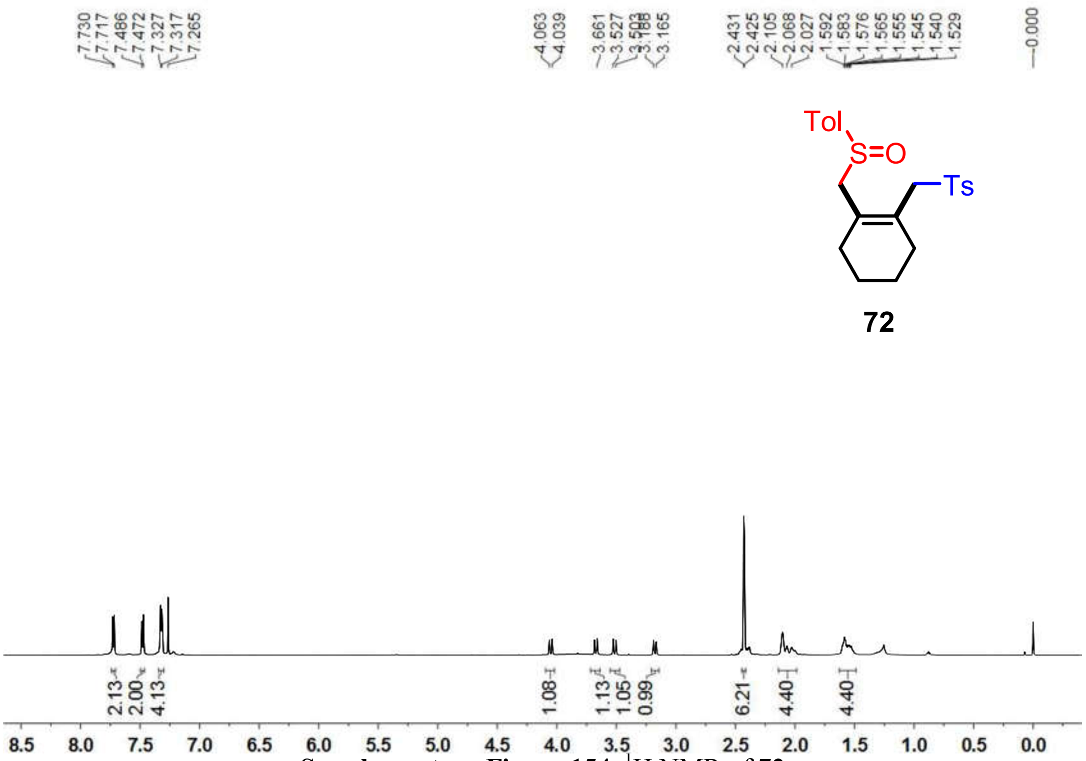

Supplementary Figure 154. ^{1}H NMR of **72**

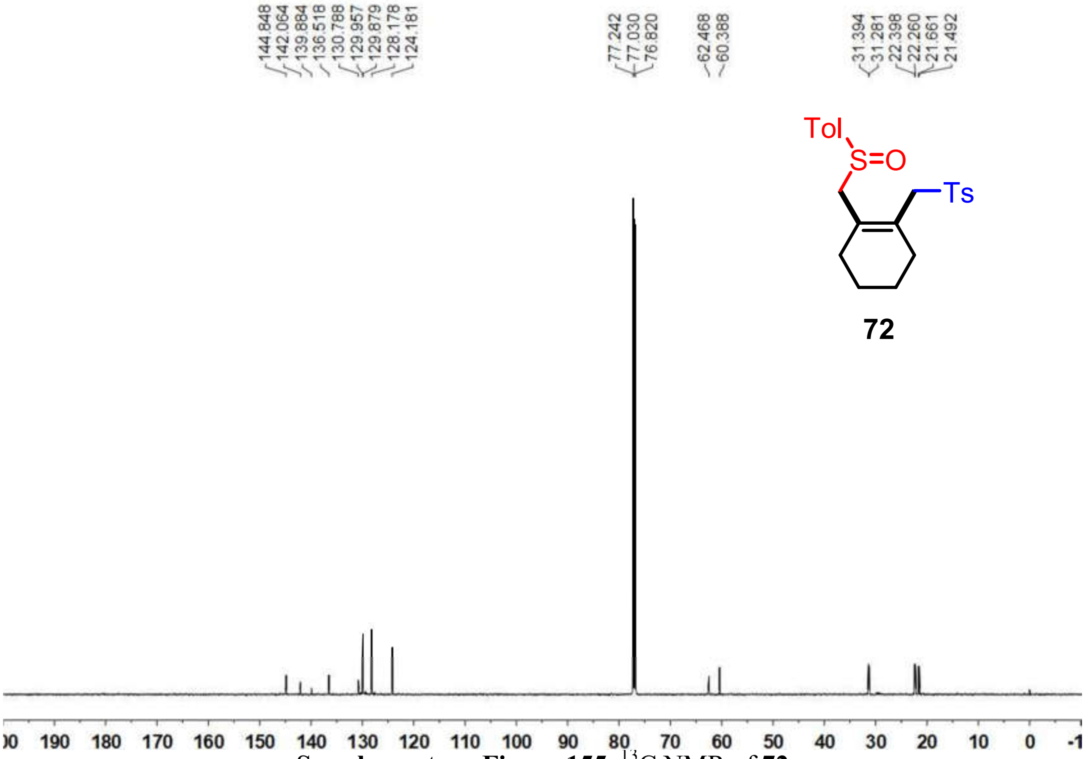

Supplementary Figure 155. ^{13}C NMR of **72**

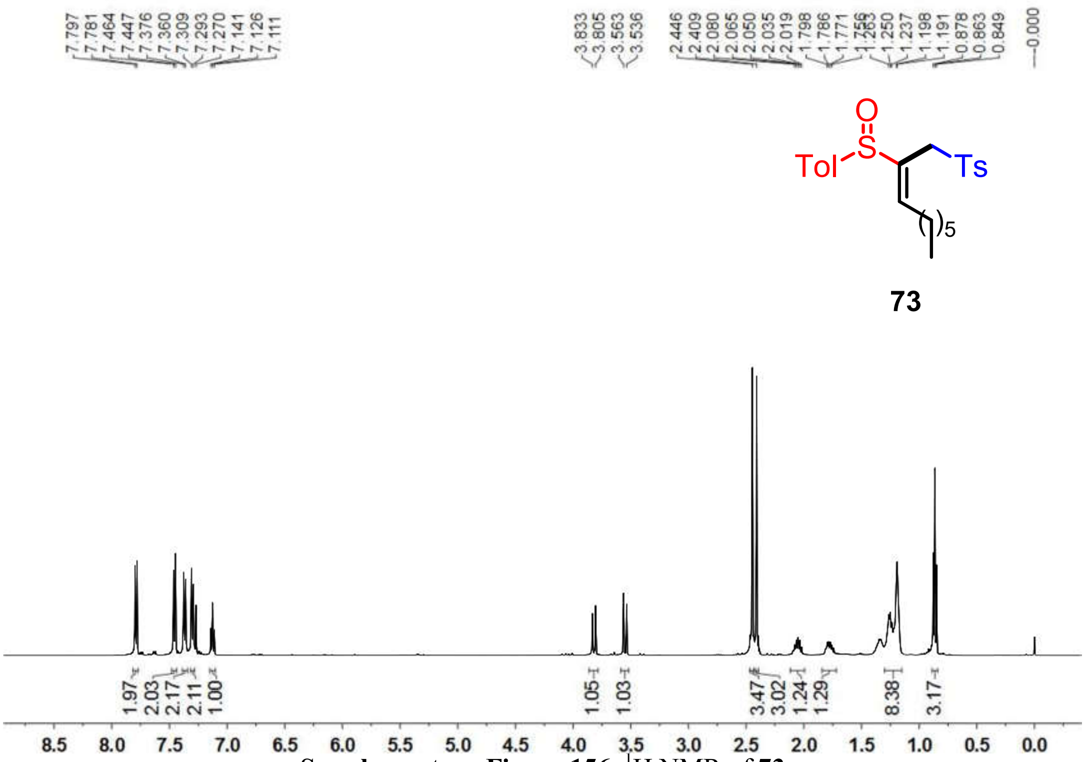

Supplementary Figure 156. ^{1}H NMR of **73**

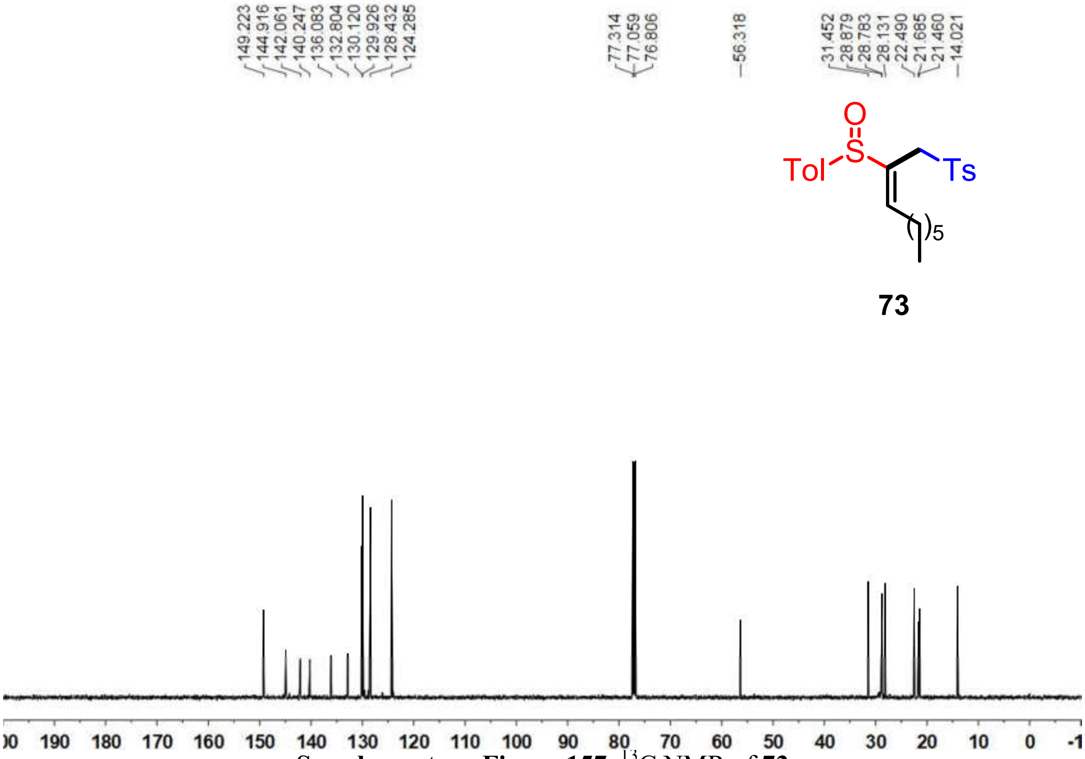

Supplementary Figure 157. ^{13}C NMR of **73**

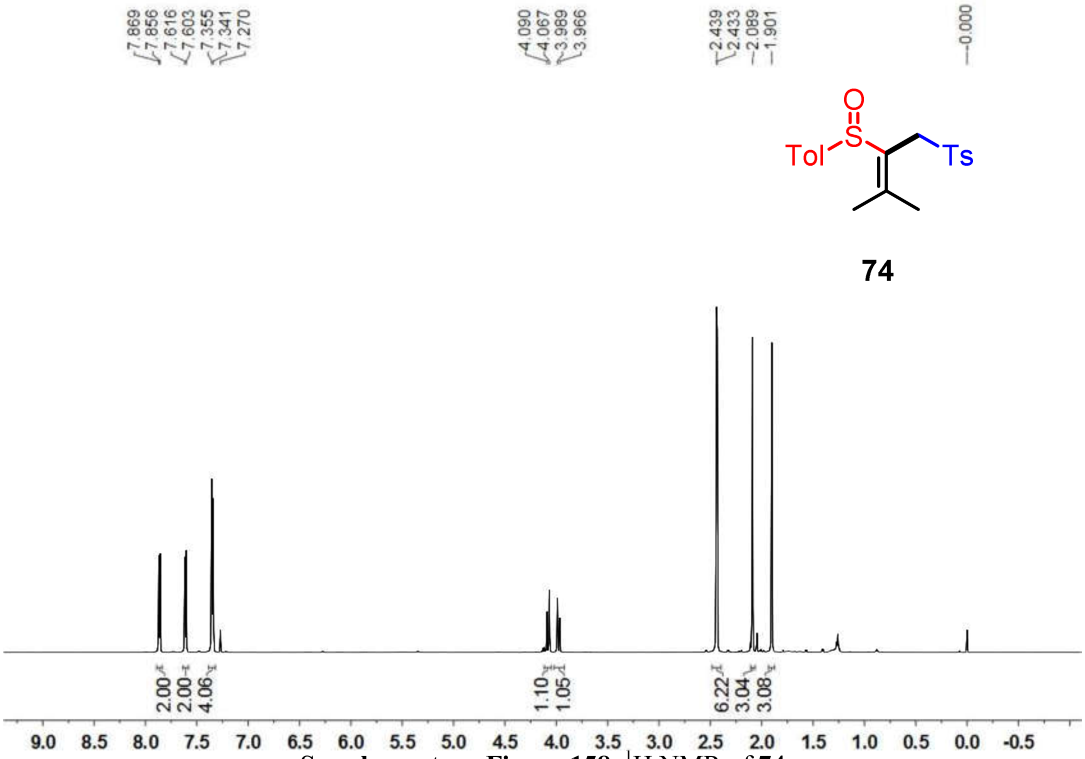

Supplementary Figure 158. ^{1}H NMR of **74**

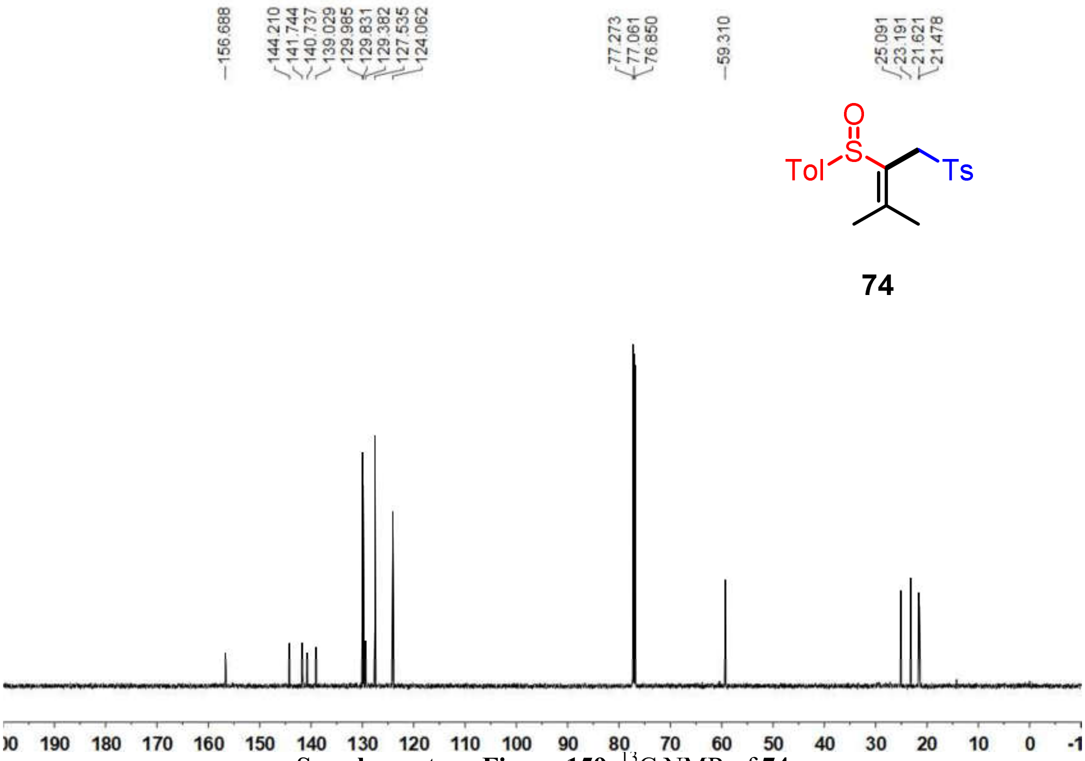

Supplementary Figure 159. ^{13}C NMR of **74**

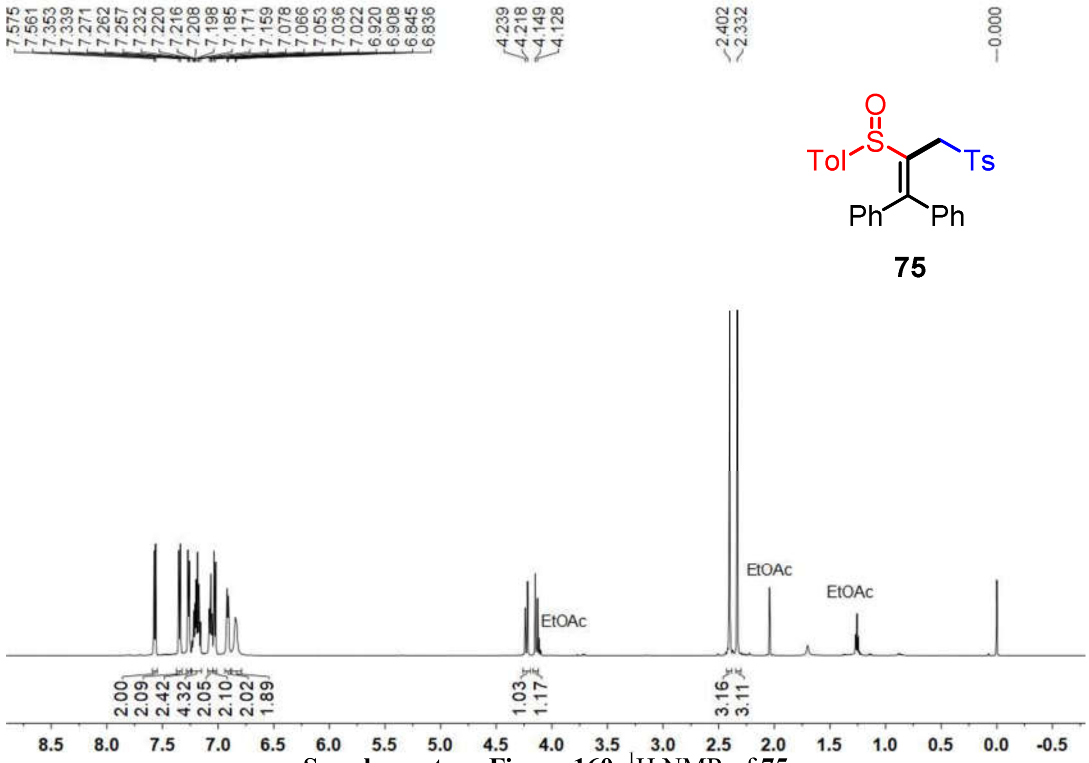

Supplementary Figure 160. ^{1}H NMR of **75**

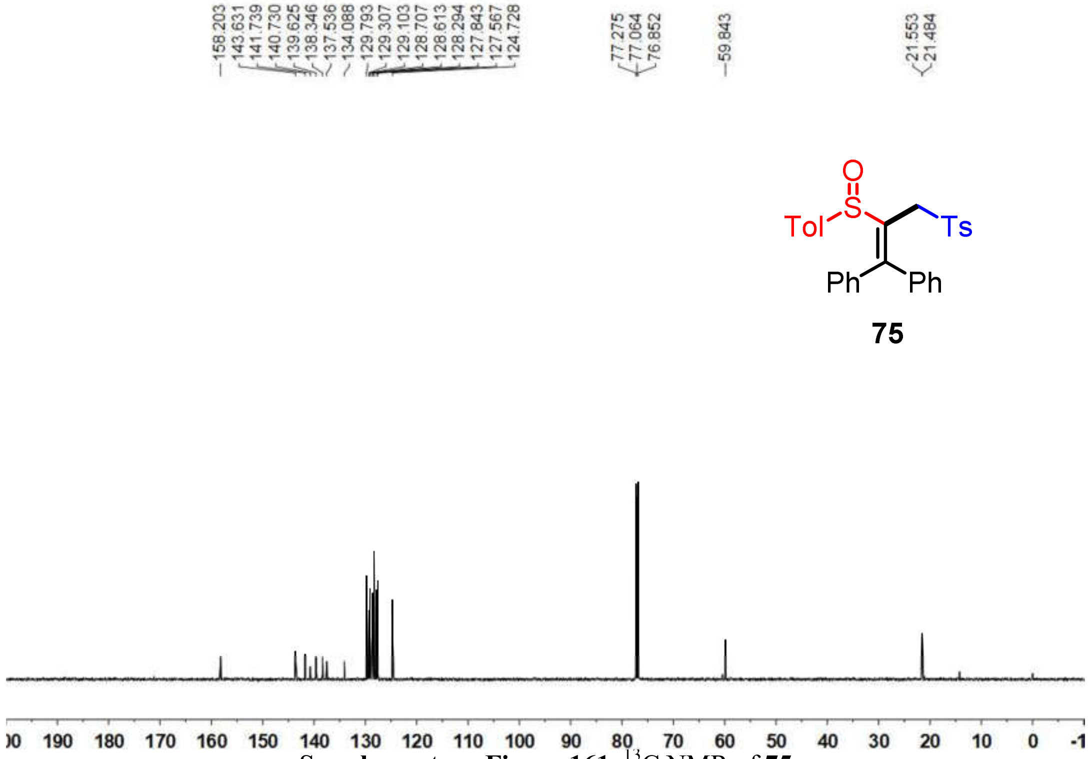

Supplementary Figure 161. ^{13}C NMR of **75**

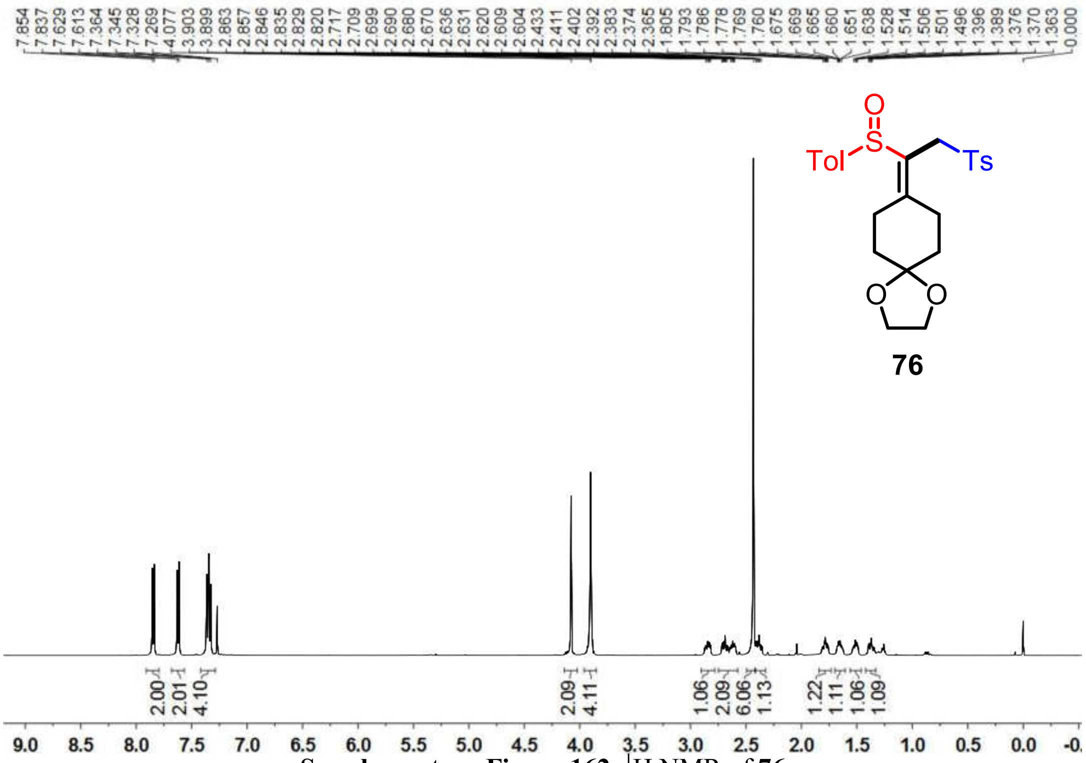

Supplementary Figure 162. ^{1}H NMR of **76**

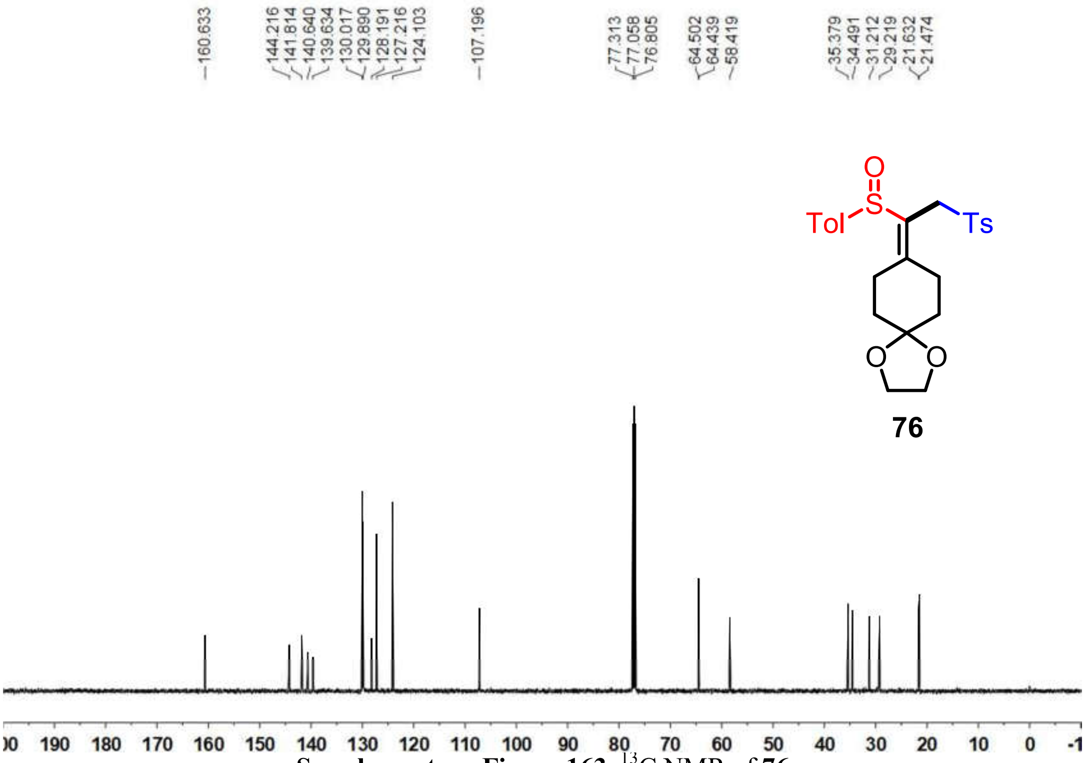

Supplementary Figure 163. ^{13}C NMR of **76**

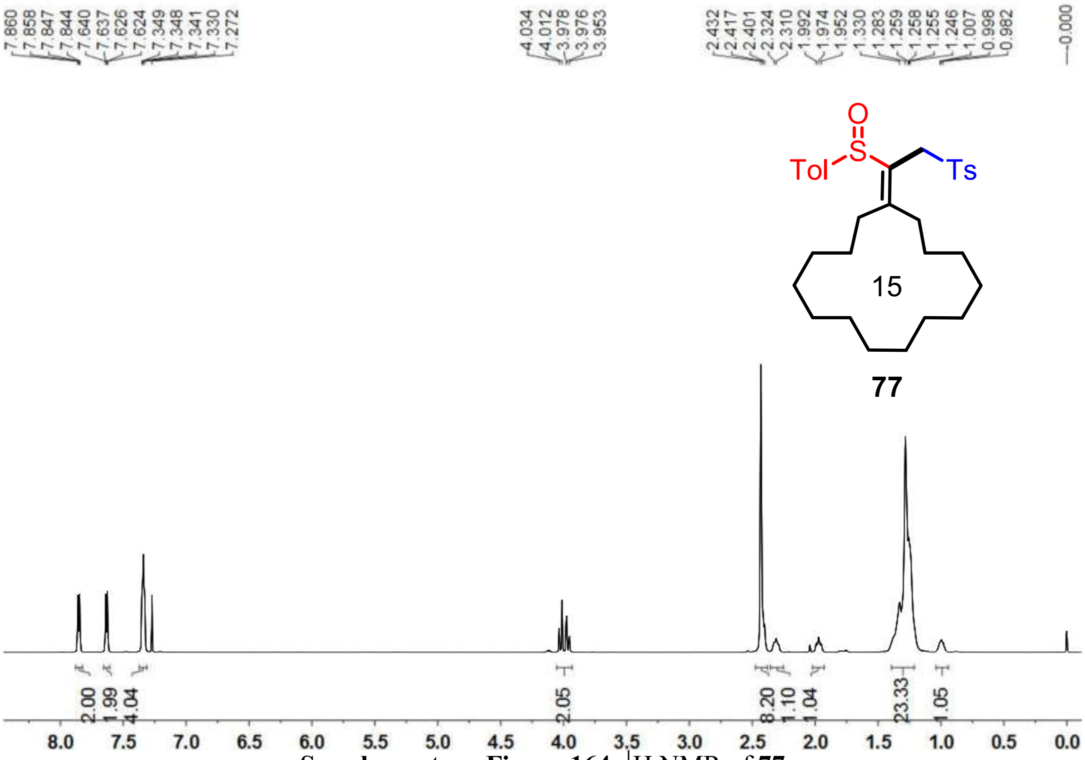

Supplementary Figure 164. ^{1}H NMR of **77**

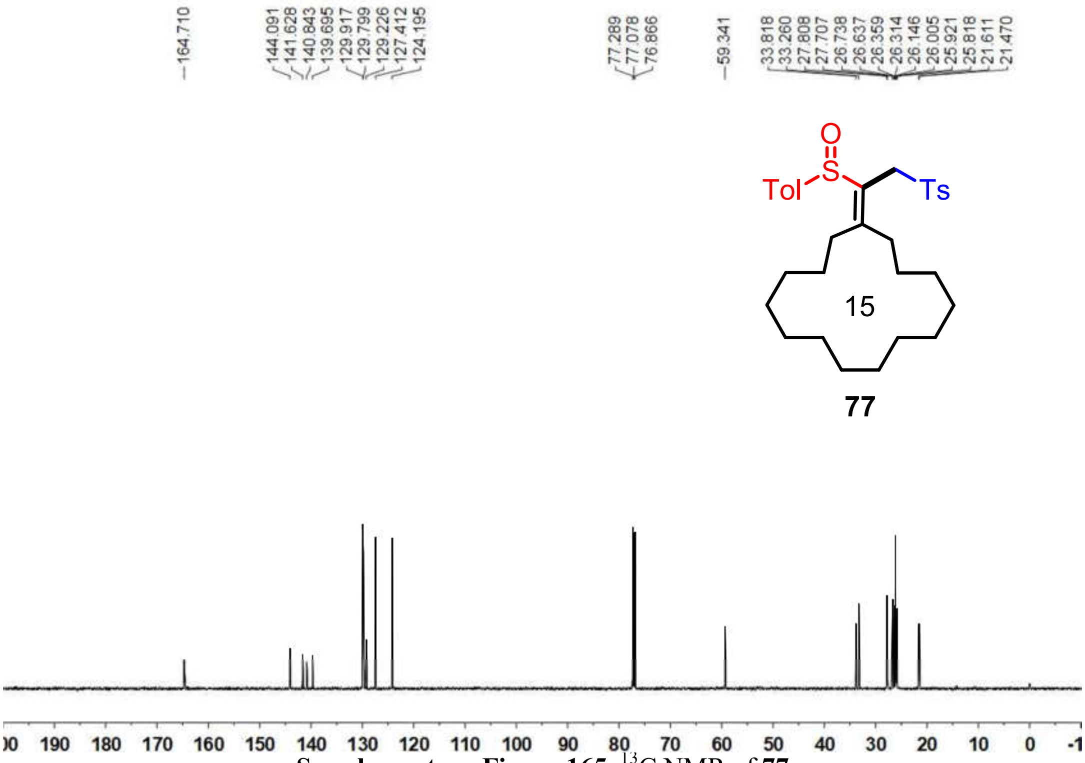

Supplementary Figure 165. ^{13}C NMR of **77**

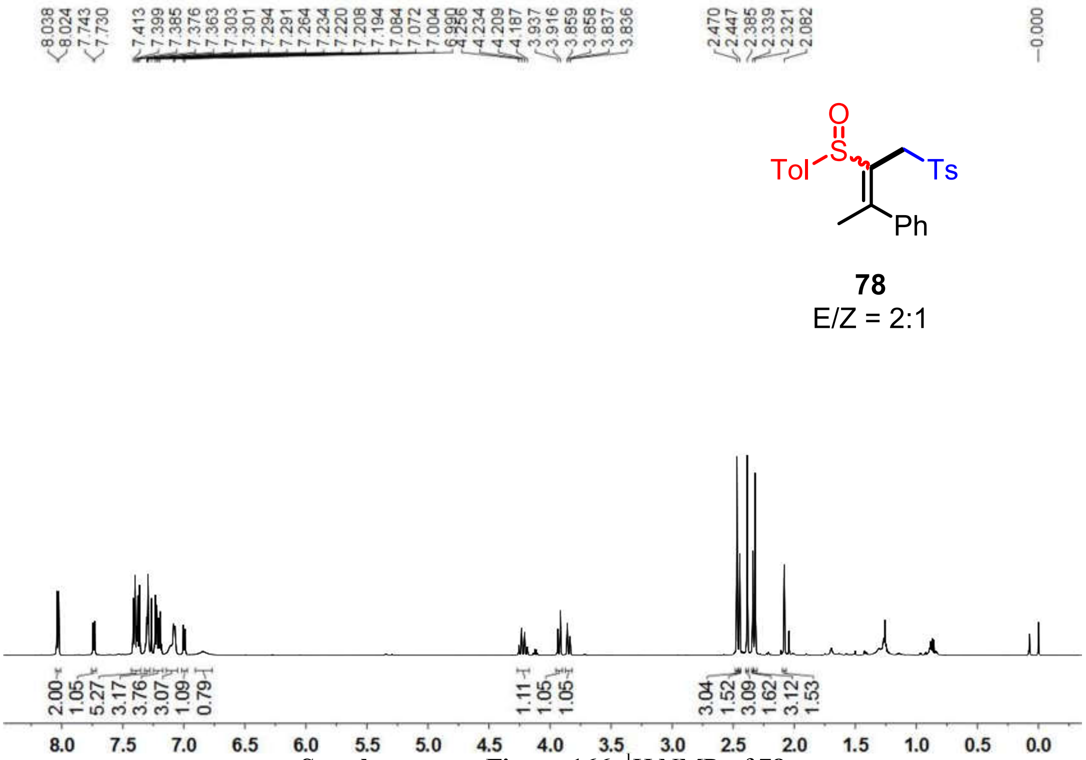

Supplementary Figure 166. ^{1}H NMR of **78**

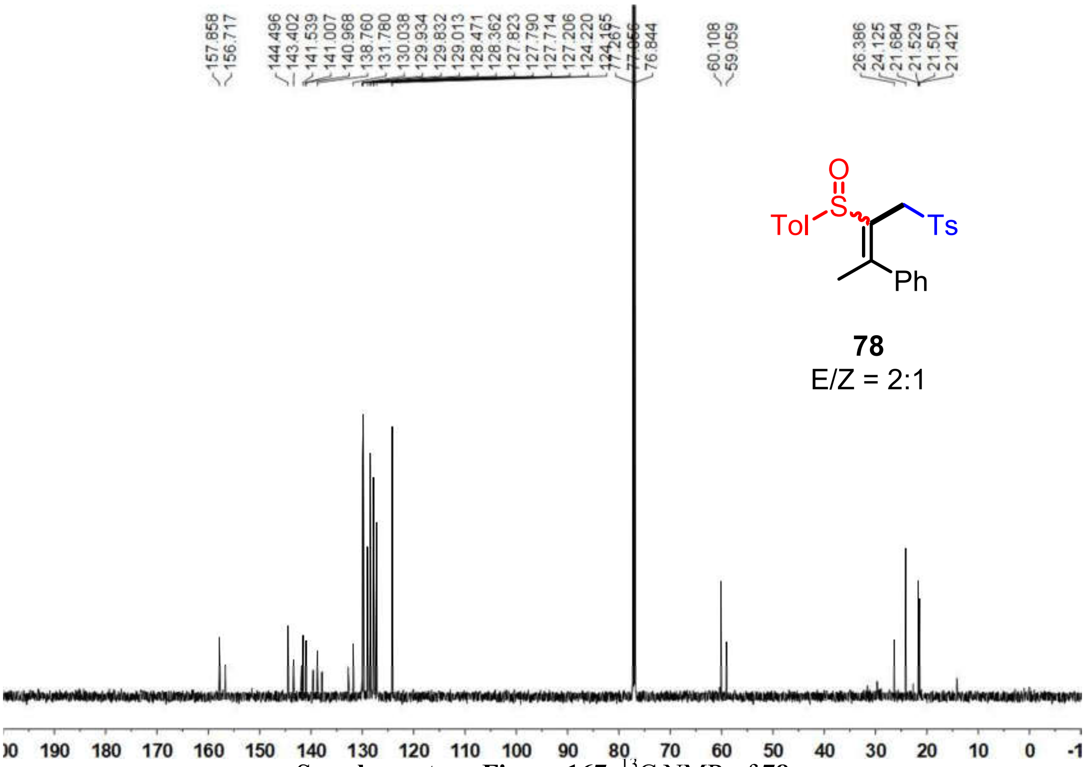

Supplementary Figure 167. ^{13}C NMR of **78**

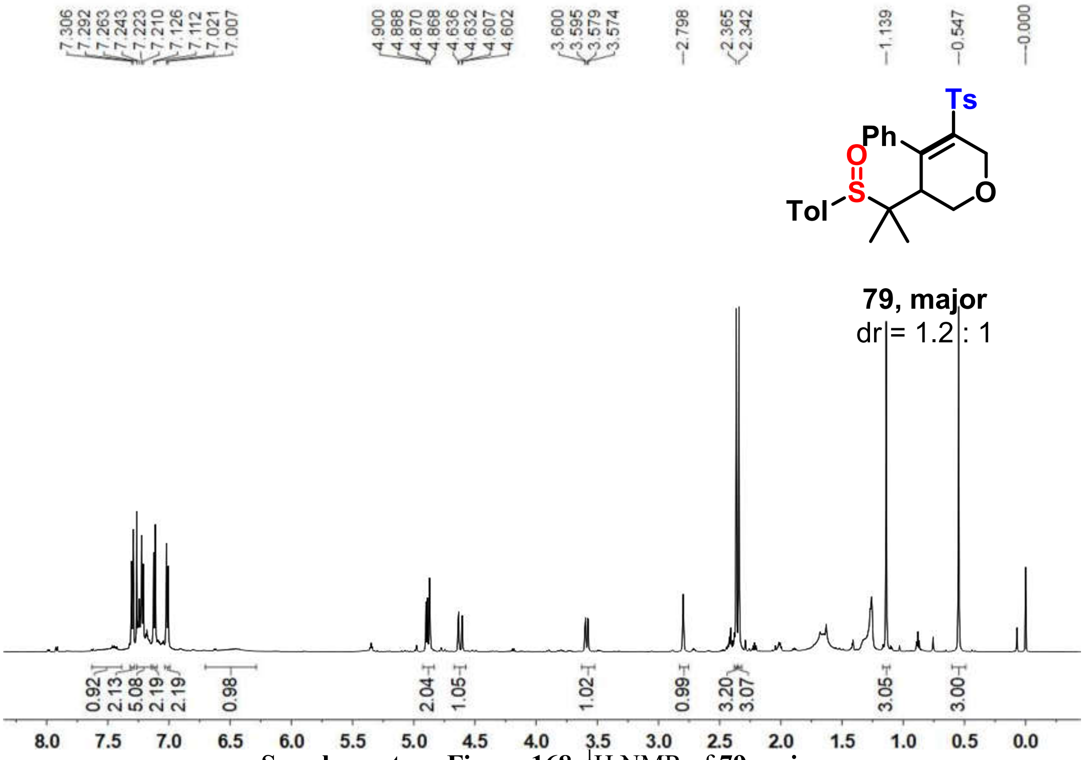

Supplementary Figure 168. ^{1}H NMR of **79 major**

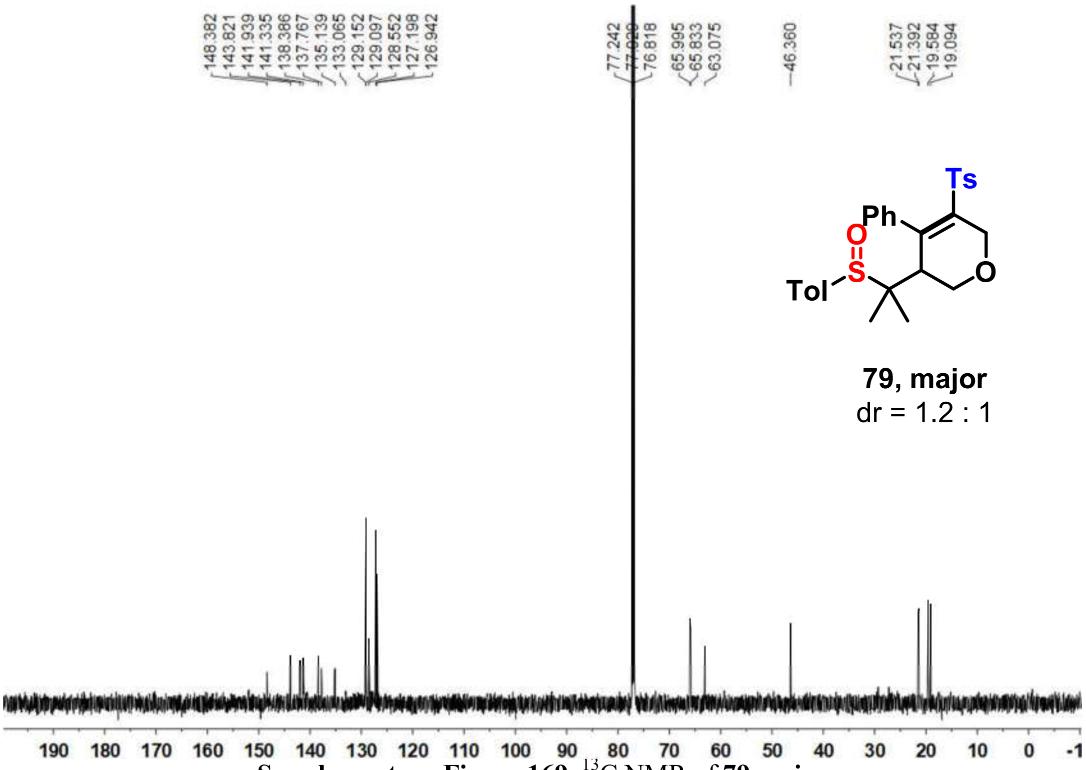

Supplementary Figure 169. ^{13}C NMR of **79 major**

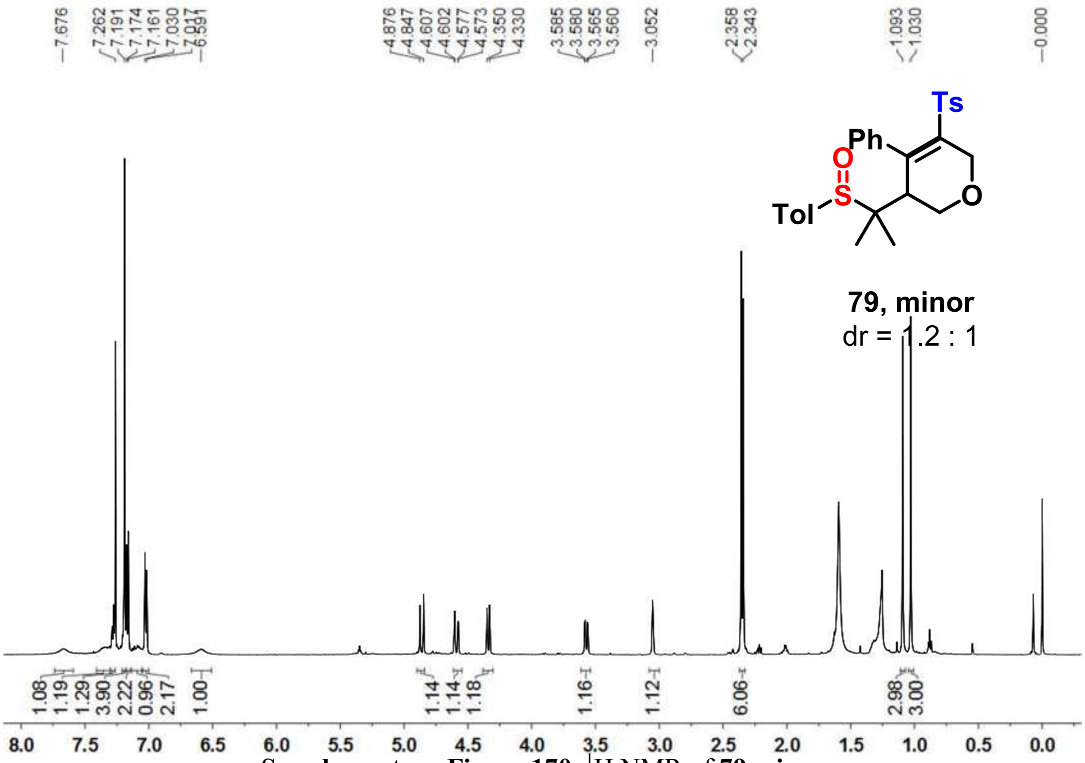

Supplementary Figure 170. ^{1}H NMR of **79 minor**

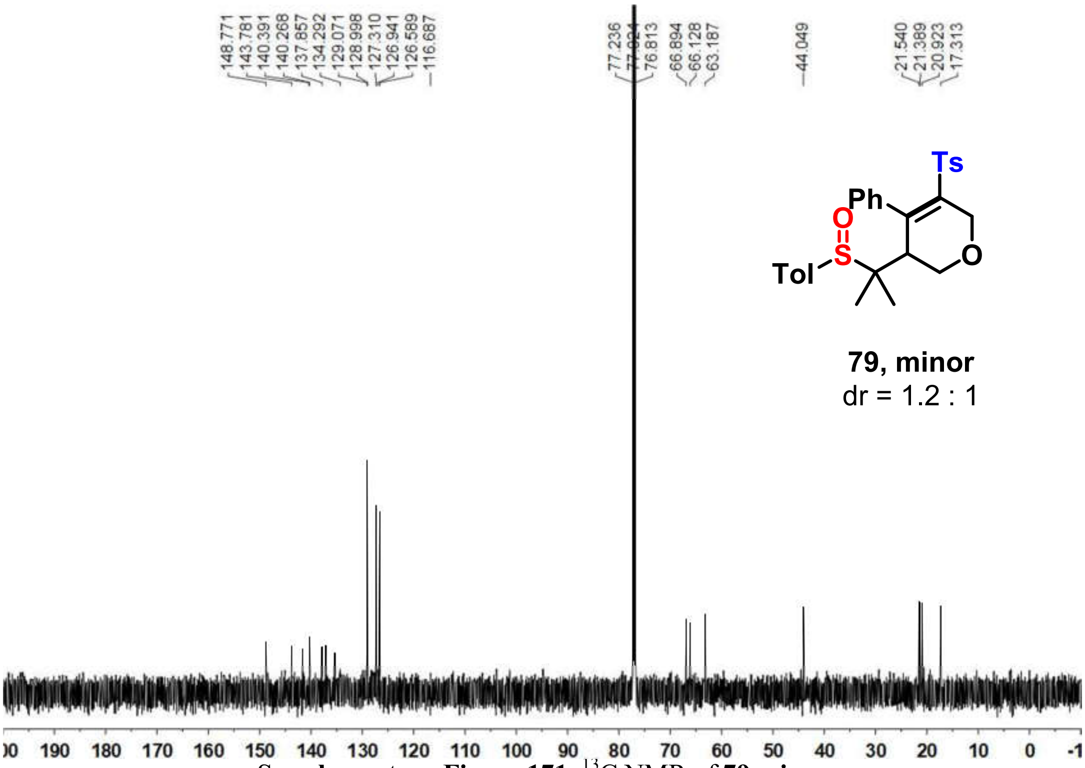

Supplementary Figure 171. ^{13}C NMR of **79 minor**

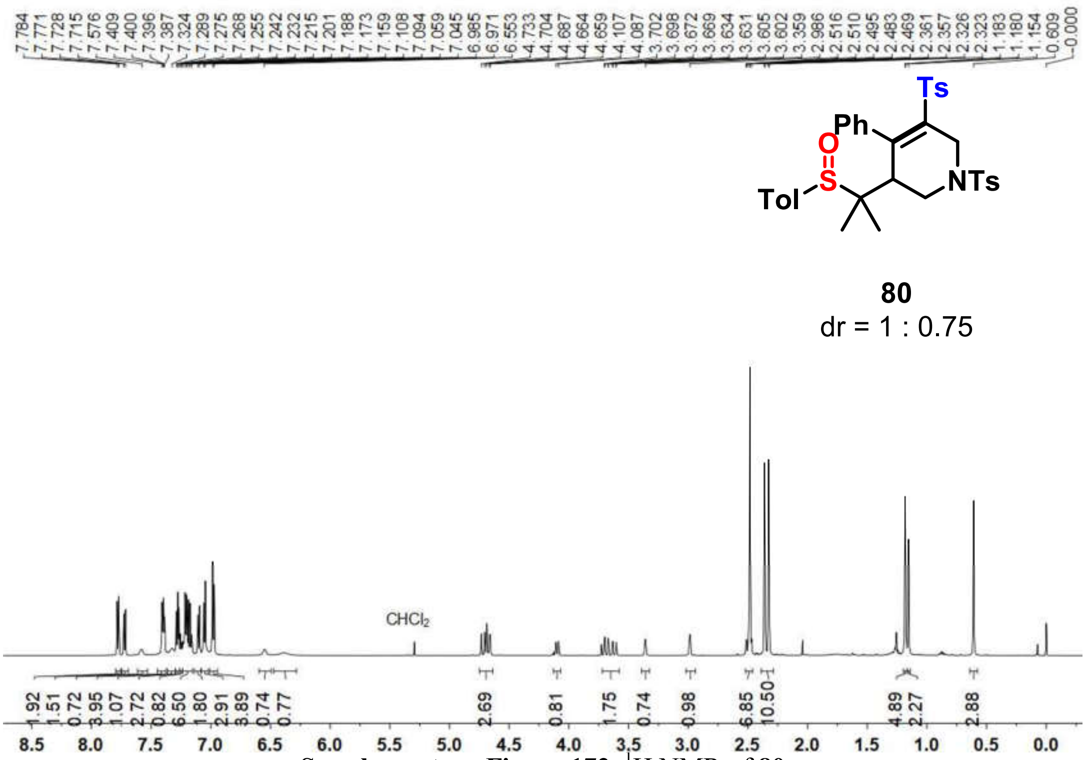

Supplementary Figure 172. ^{1}H NMR of **80**

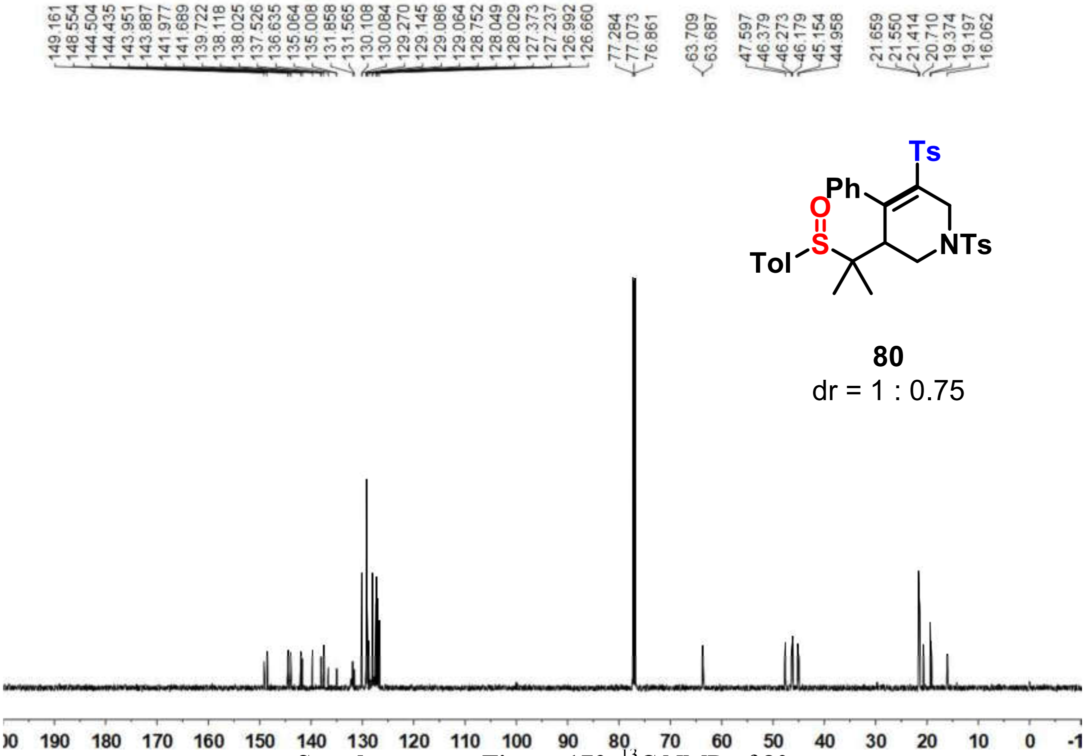

Supplementary Figure 173. ^{13}C NMR of **80**

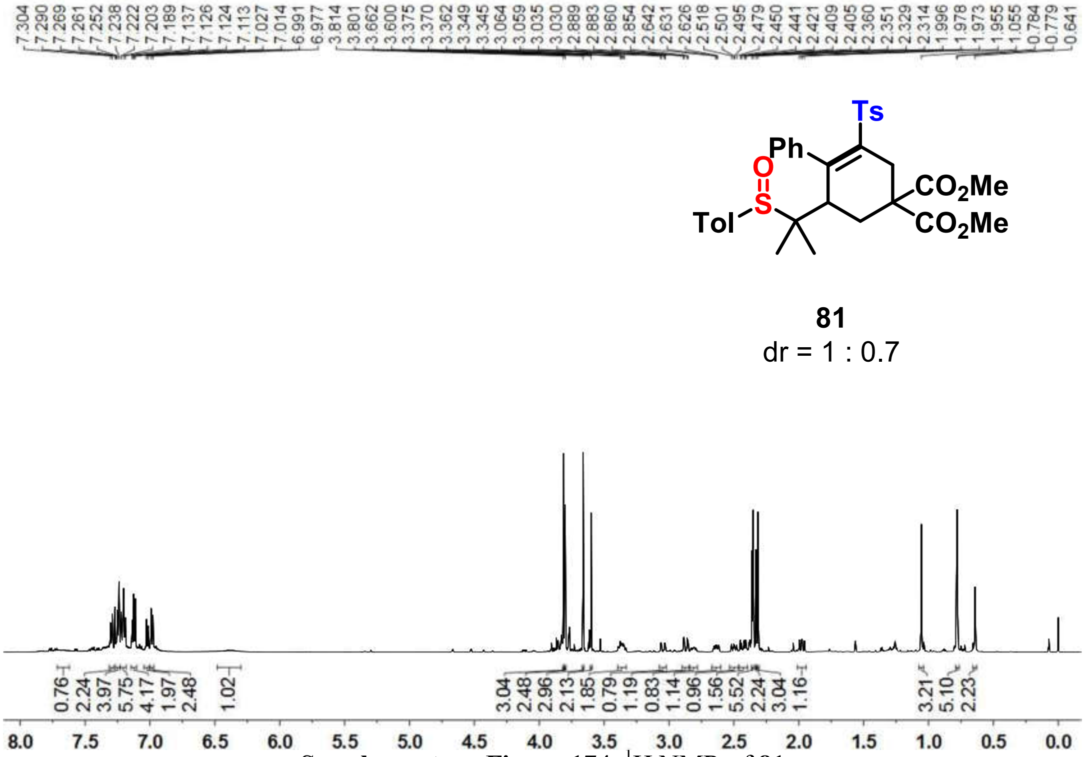

Supplementary Figure 174. ^{1}H NMR of **81**

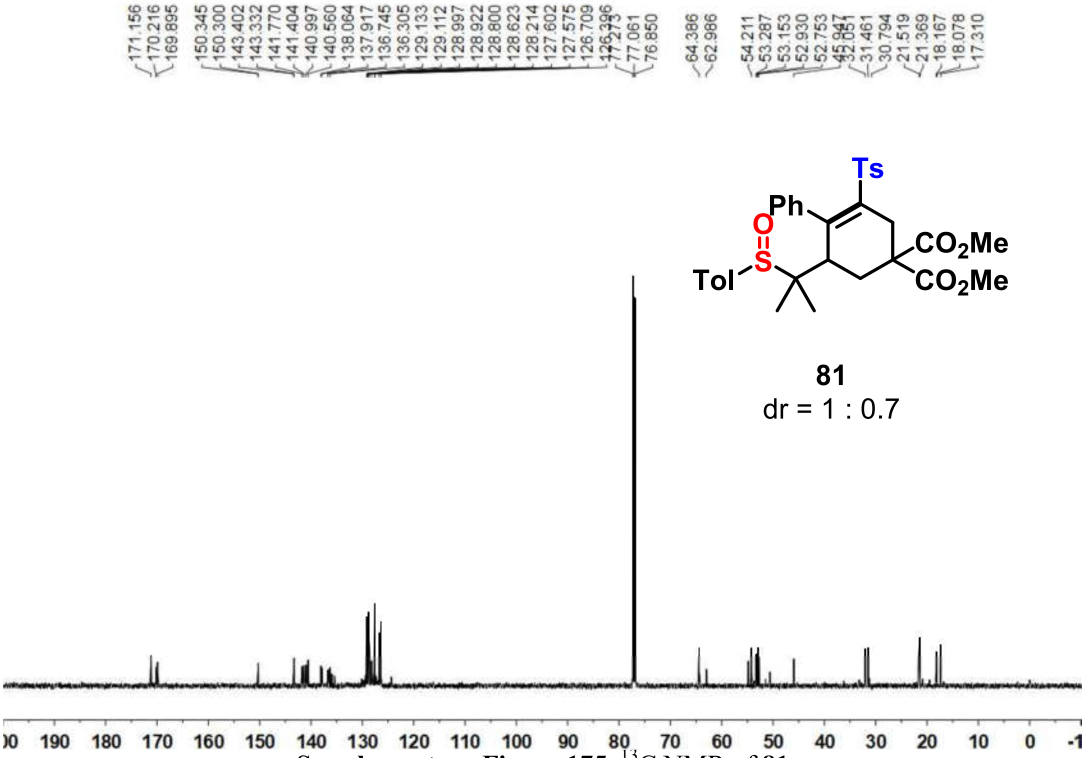

Supplementary Figure 175. ^{13}C NMR of **81**

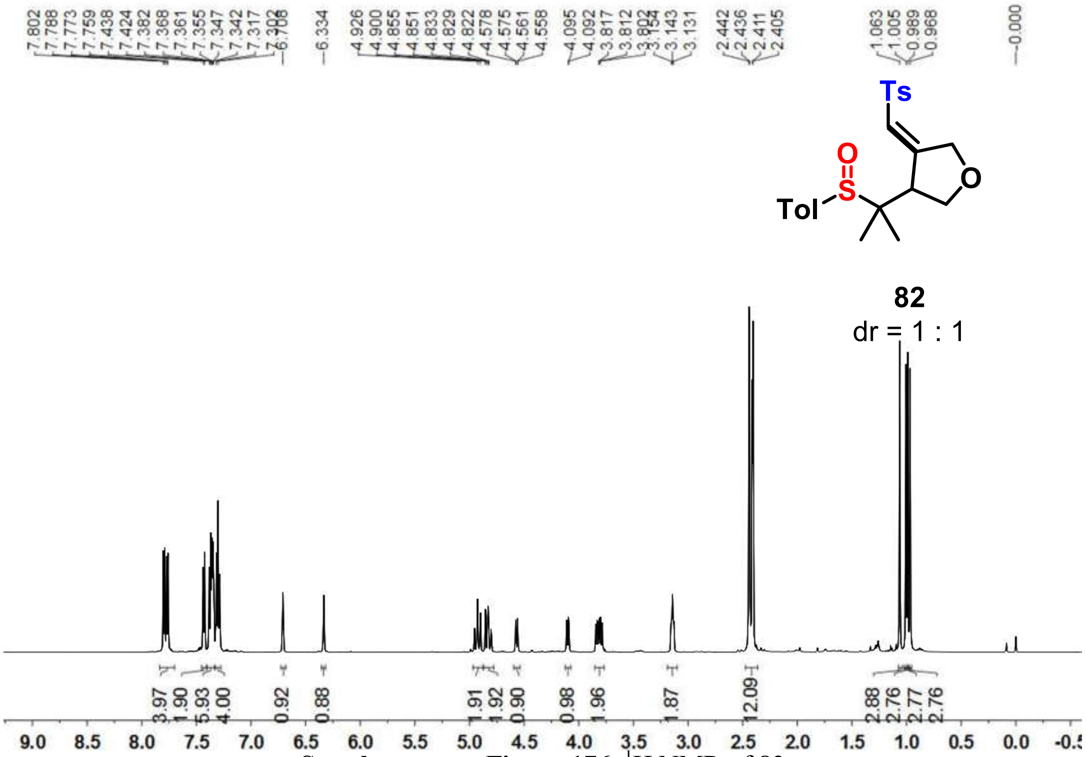

Supplementary Figure 176. ^{1}H NMR of **82**

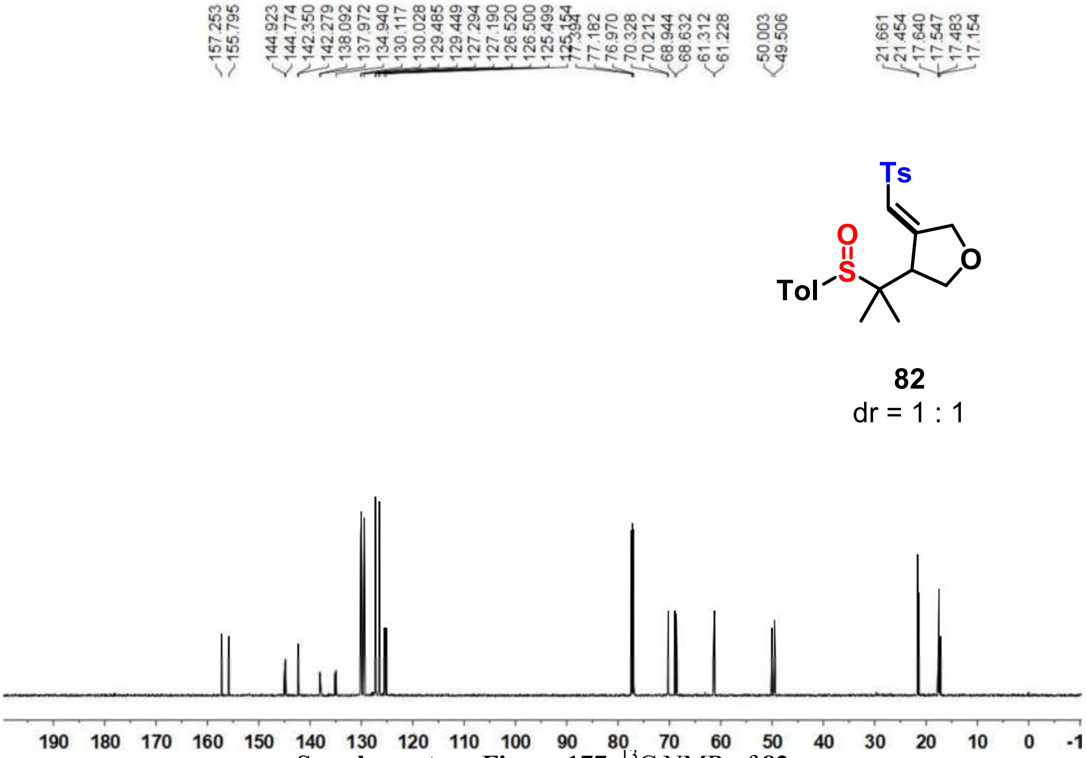

Supplementary Figure 177. ^{13}C NMR of **82**

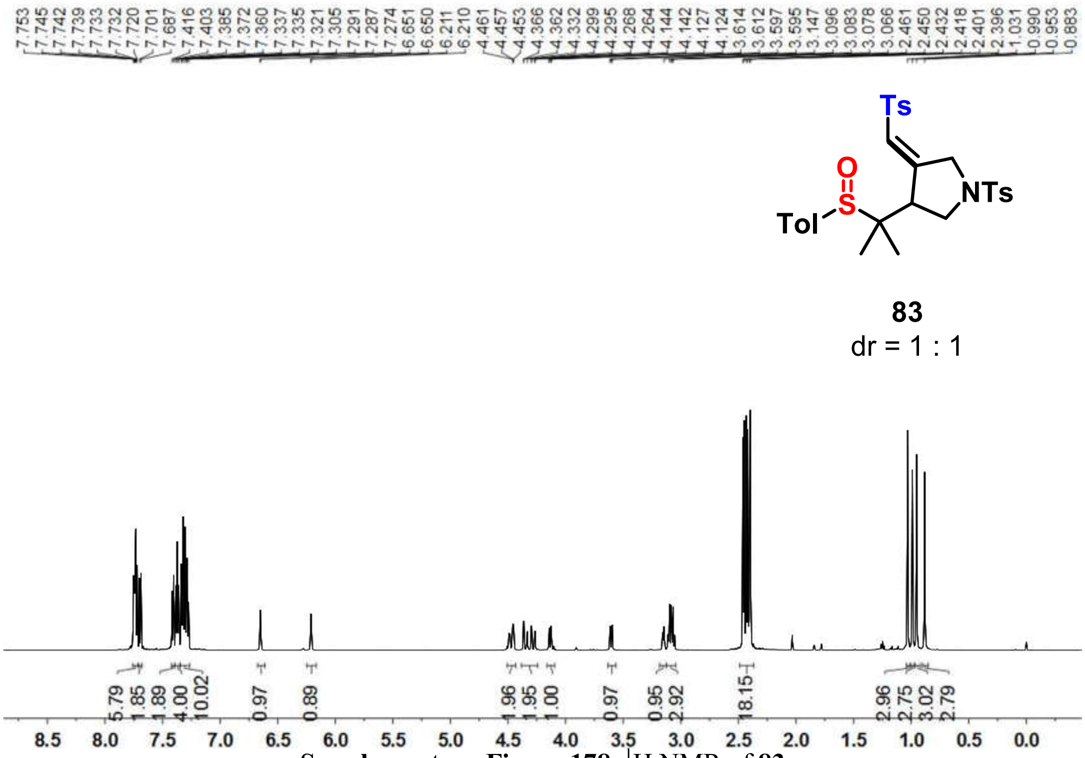

Supplementary Figure 178. ^{1}H NMR of **83**

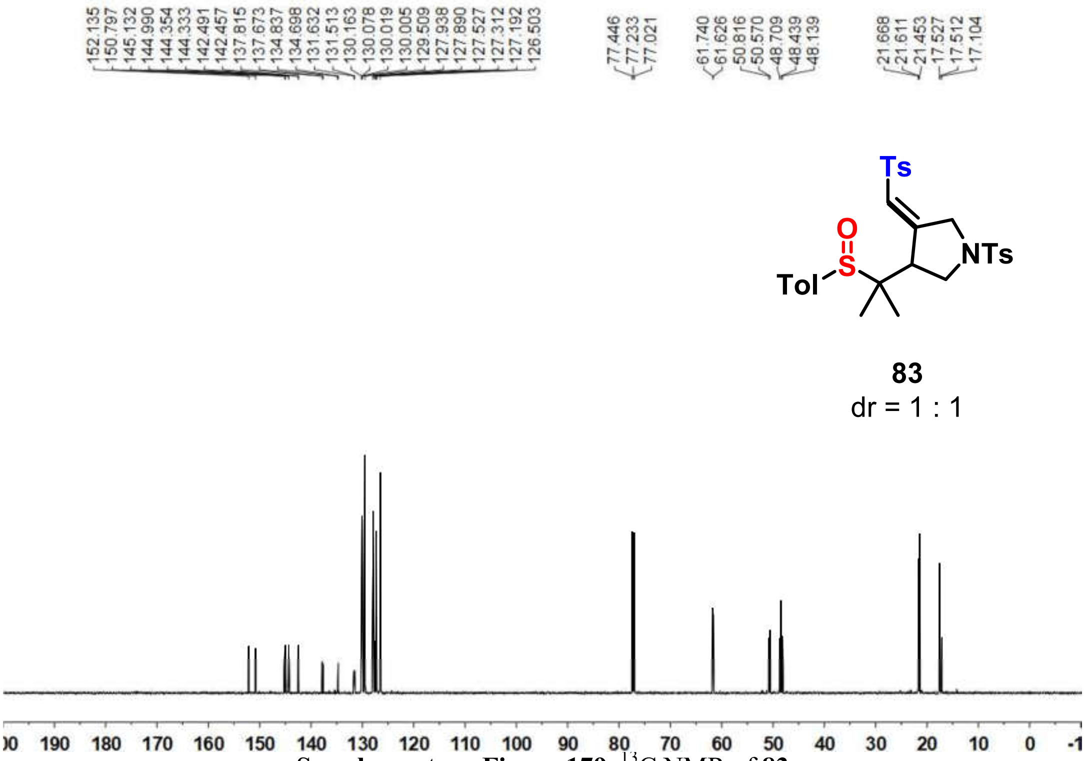

Supplementary Figure 179. ^{13}C NMR of **83**

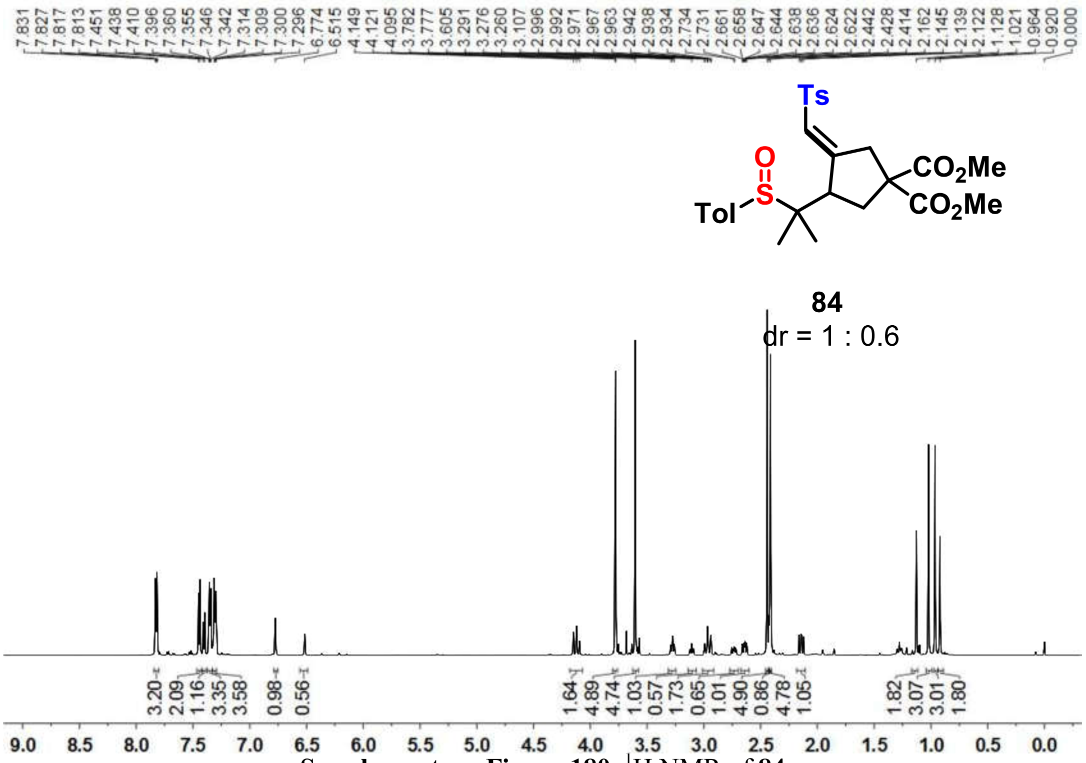

Supplementary Figure 180. ^{1}H NMR of **84**

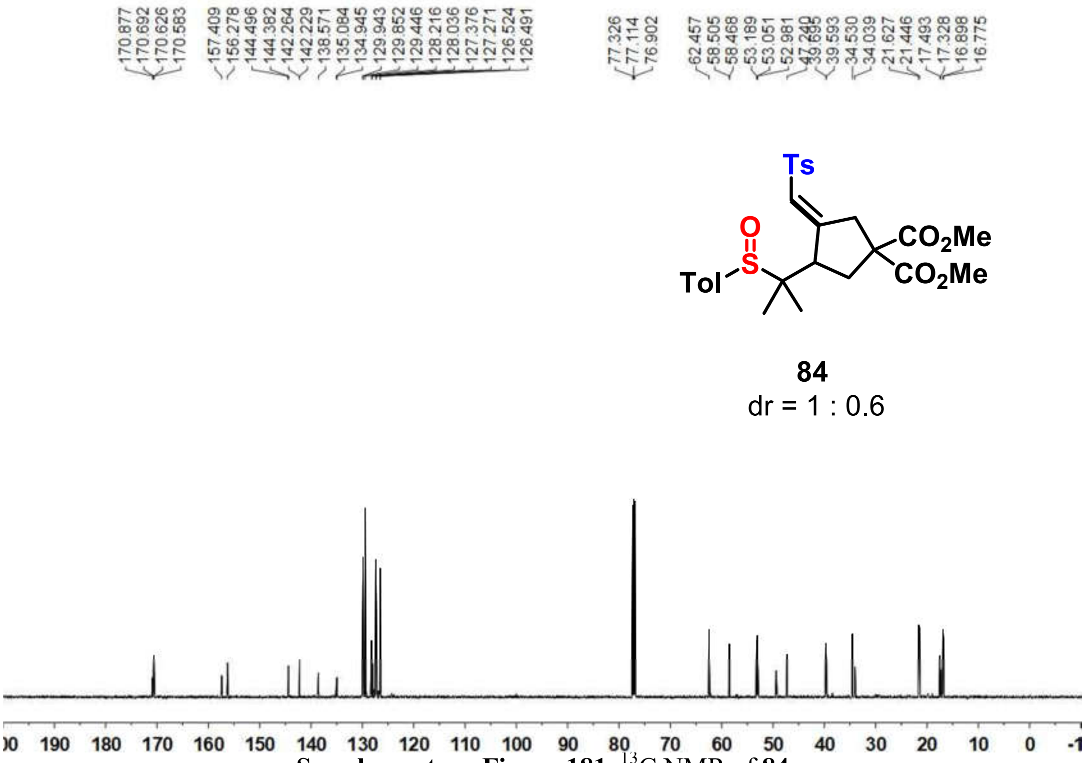

Supplementary Figure 181. ^{13}C NMR of **84**

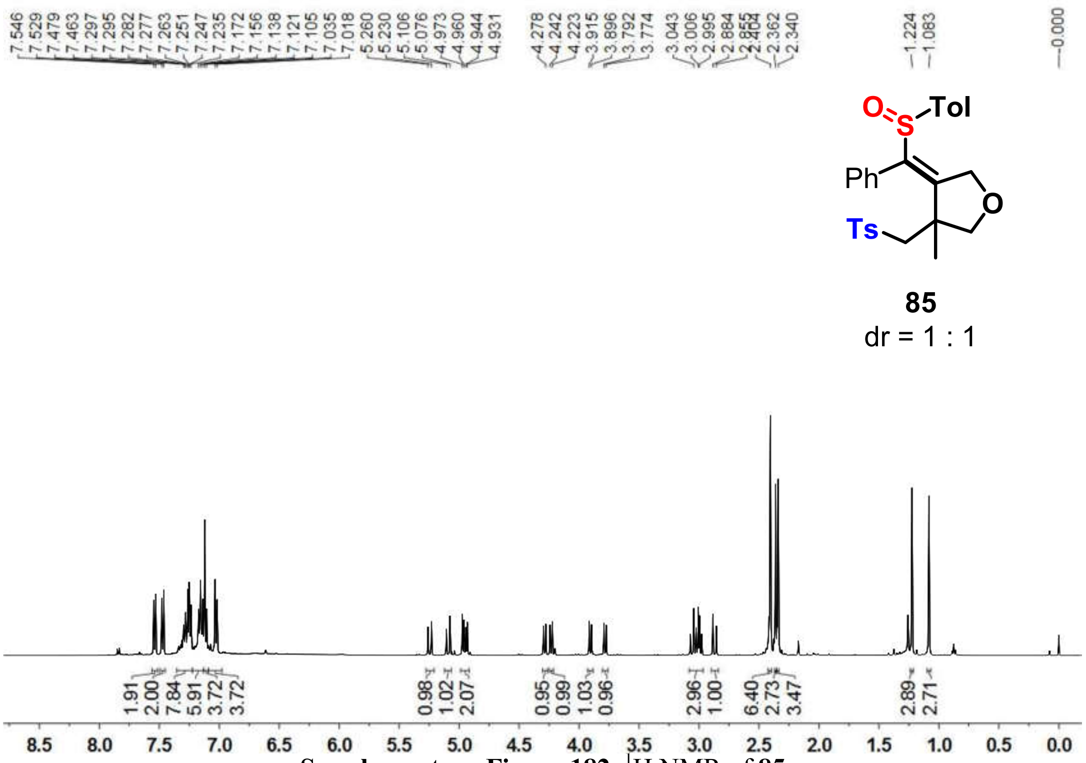

Supplementary Figure 182. ^{1}H NMR of **85**

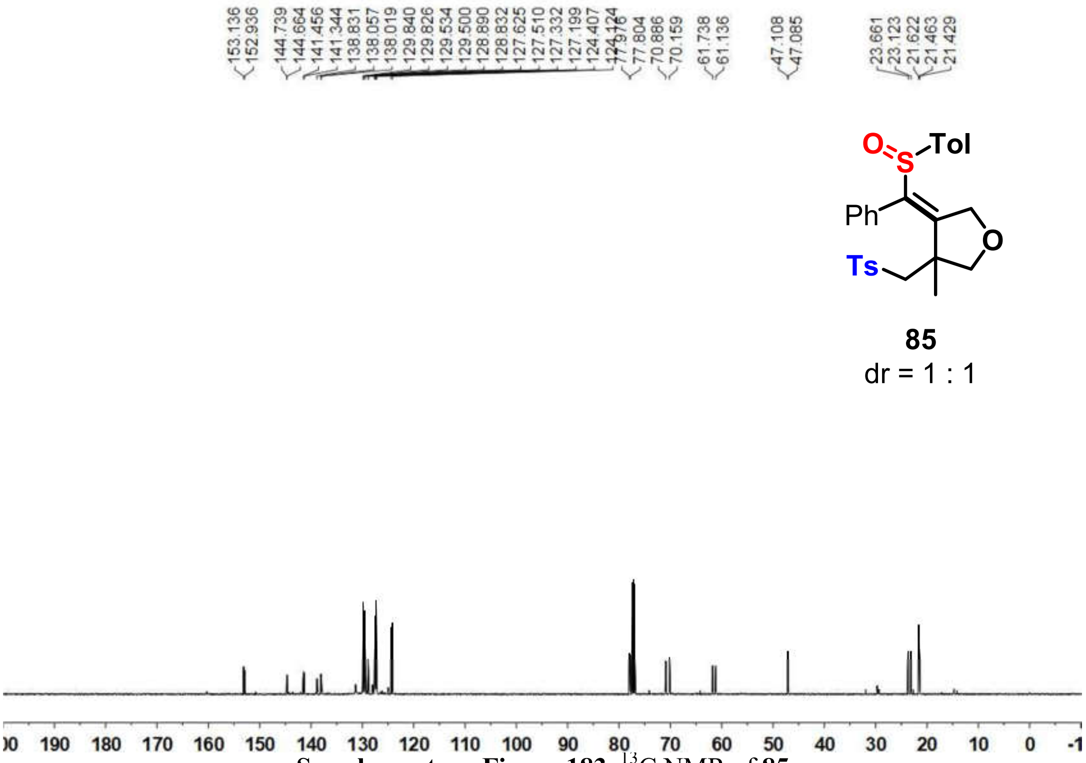

Supplementary Figure 183. ^{13}C NMR of **85**

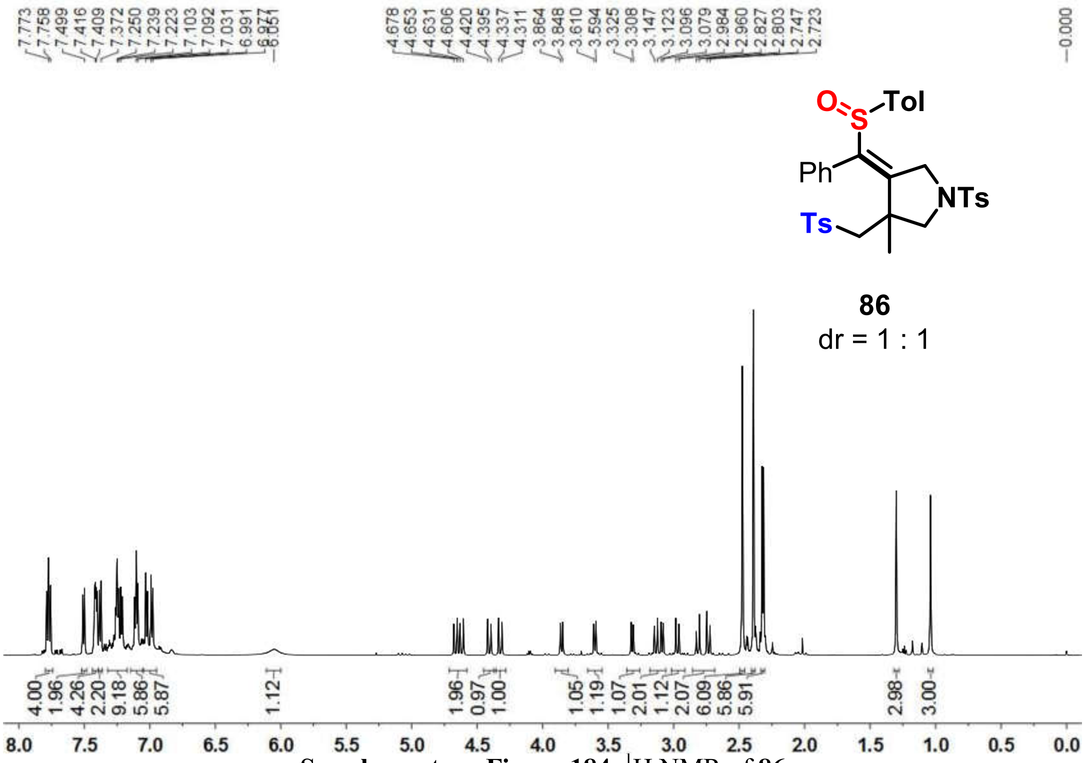

Supplementary Figure 184. ^{1}H NMR of **86**

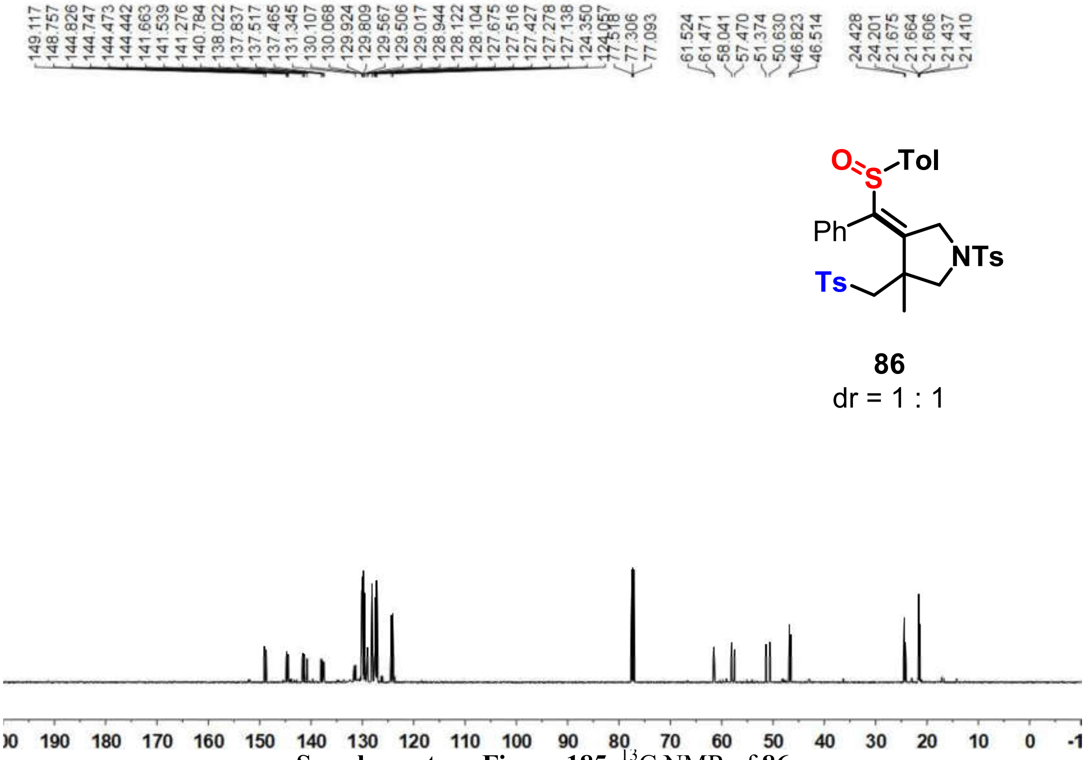

Supplementary Figure 185. ^{13}C NMR of **86**

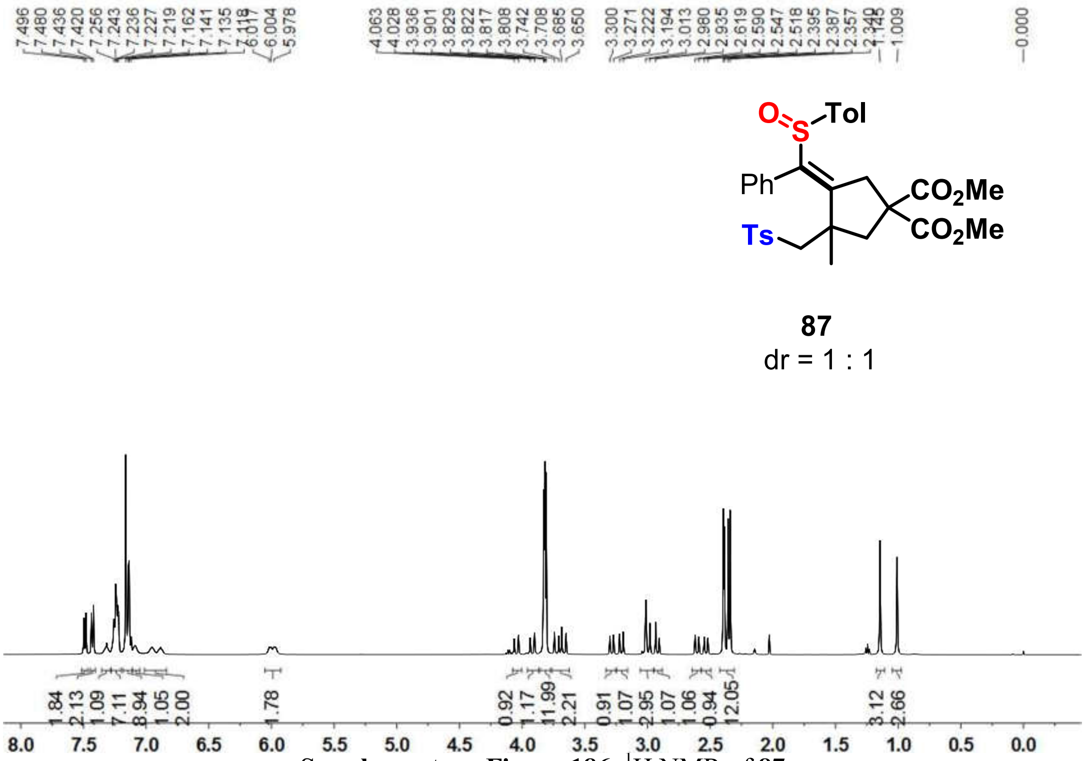

Supplementary Figure 186. ^{1}H NMR of **87**

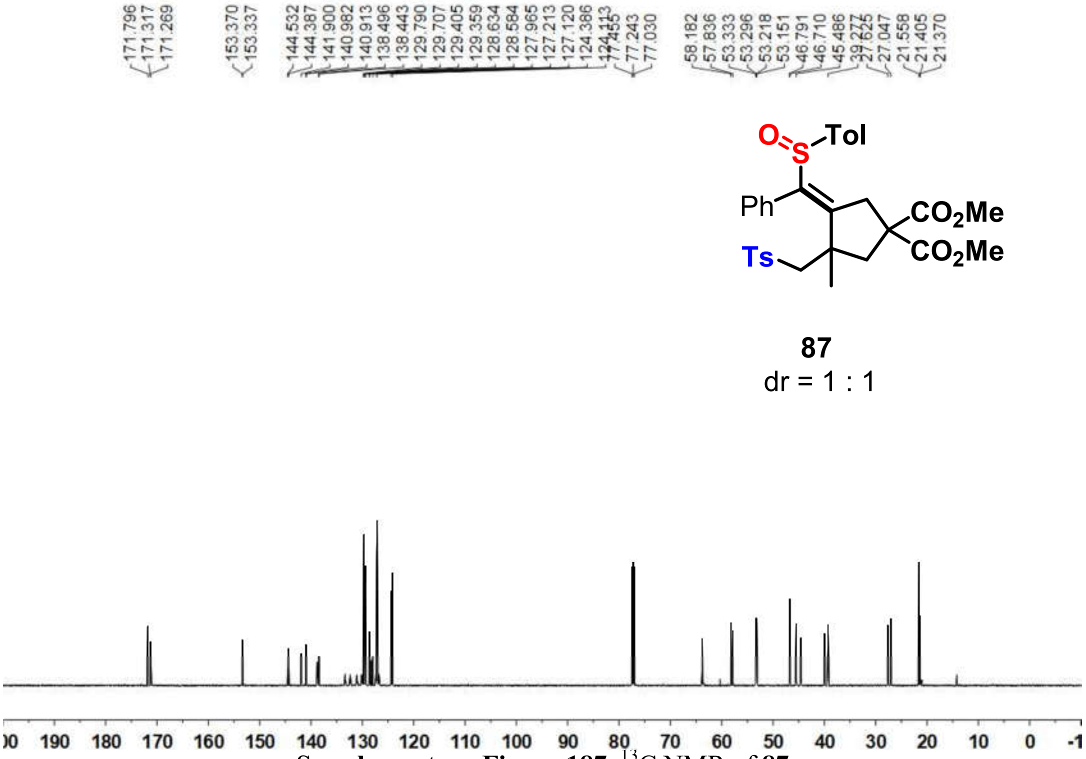

Supplementary Figure 187. ^{13}C NMR of **87**

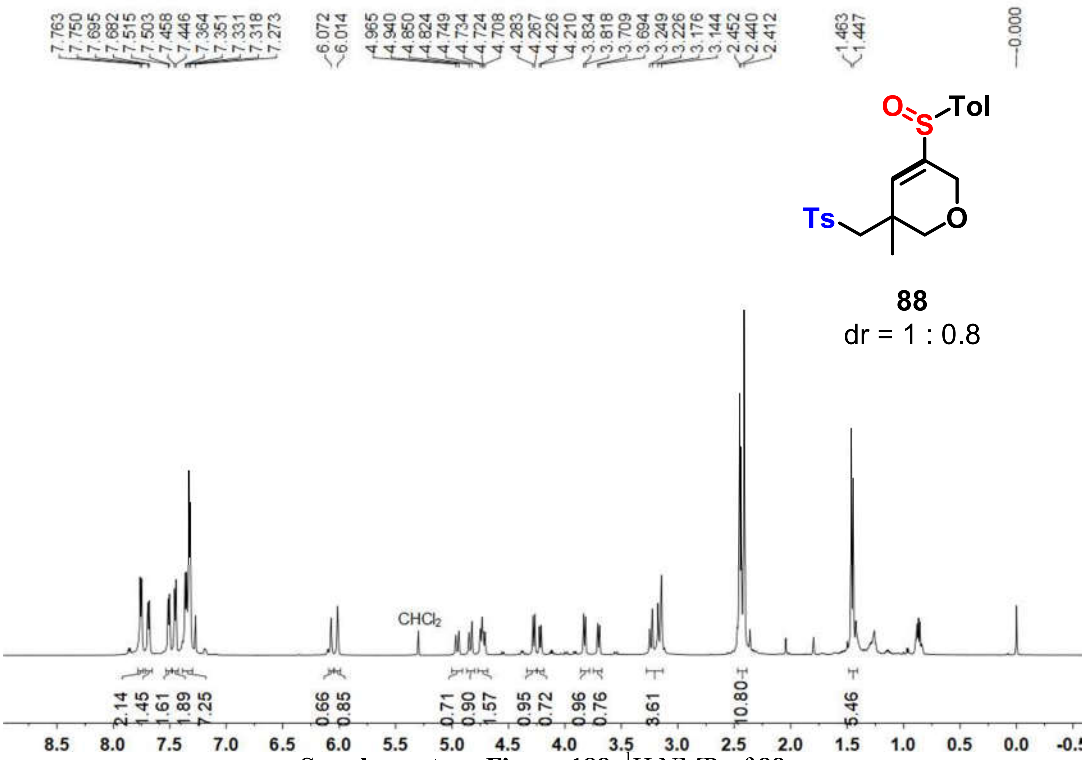

Supplementary Figure 188. ^{1}H NMR of **88**

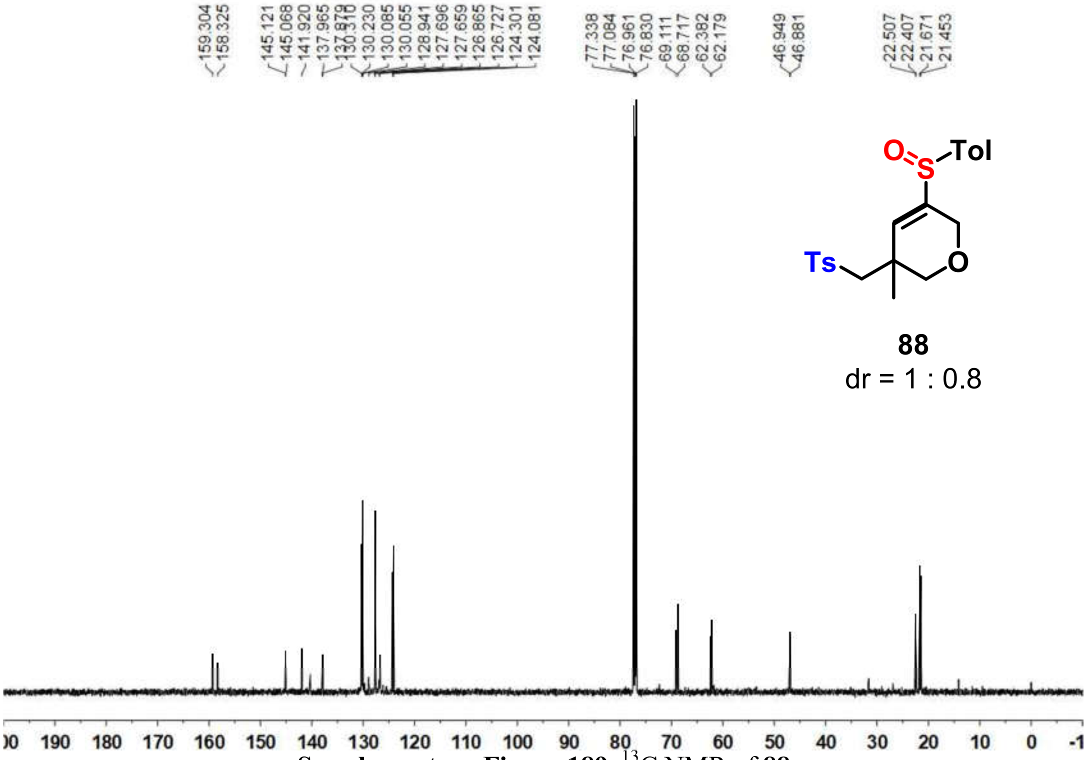

Supplementary Figure 189. ^{13}C NMR of **88**

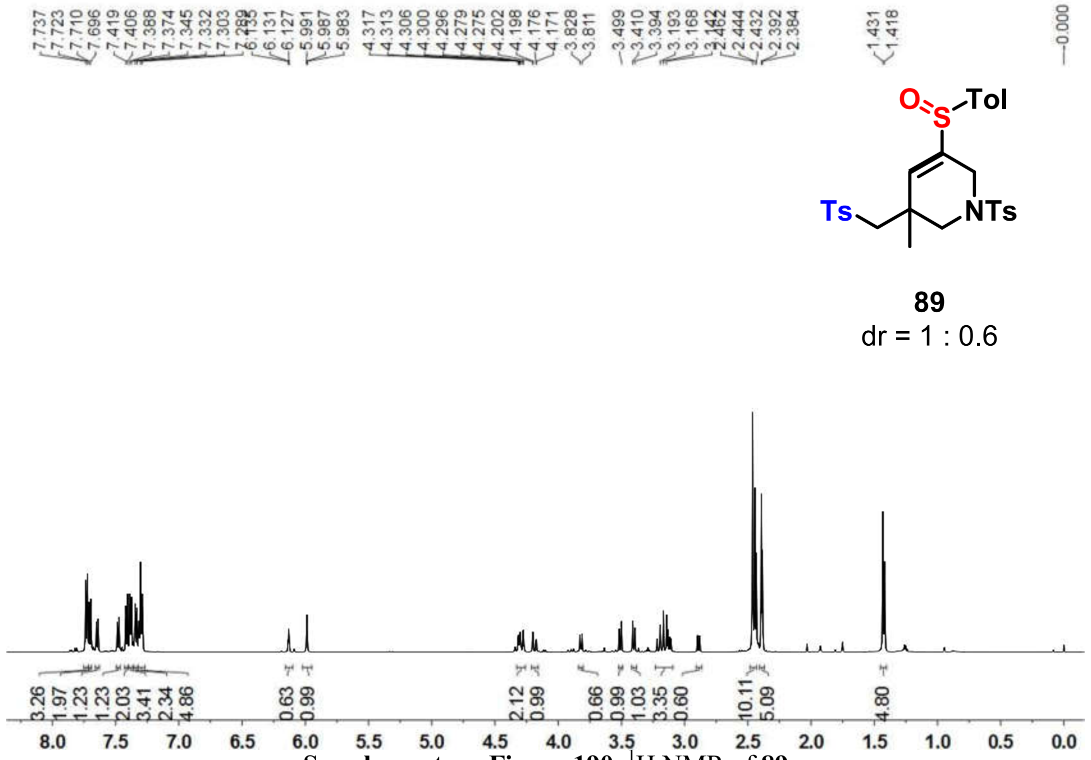

Supplementary Figure 190. ^{1}H NMR of **89**

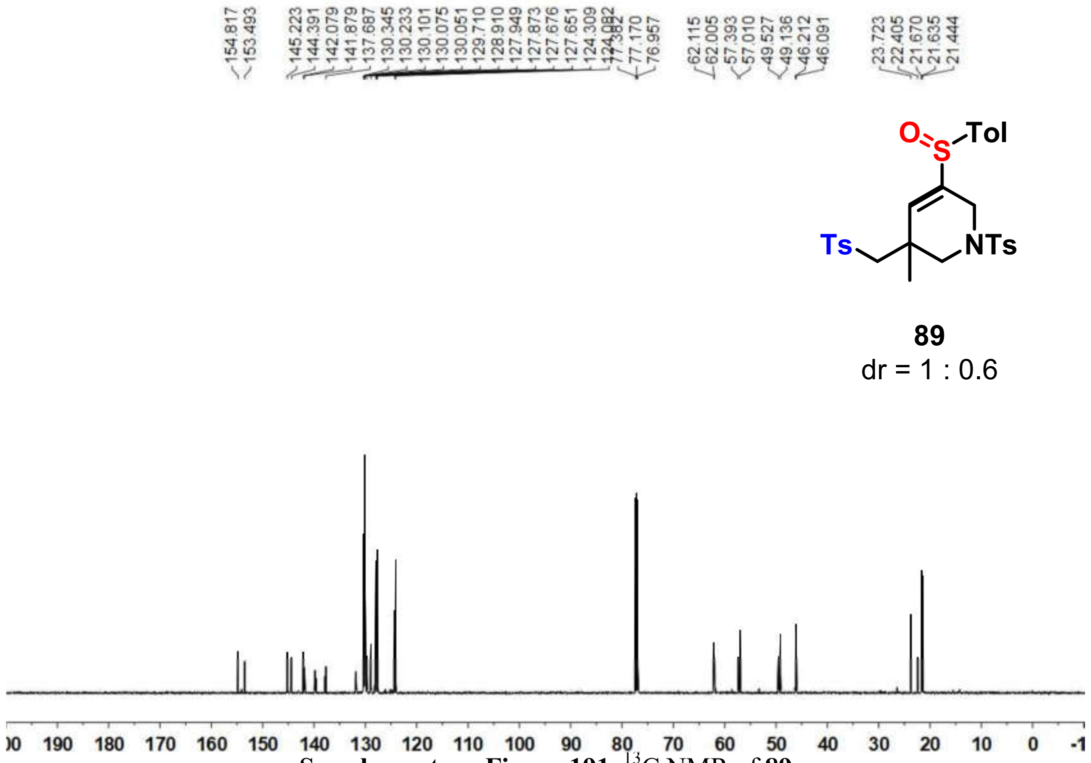

Supplementary Figure 191. ^{13}C NMR of **89**

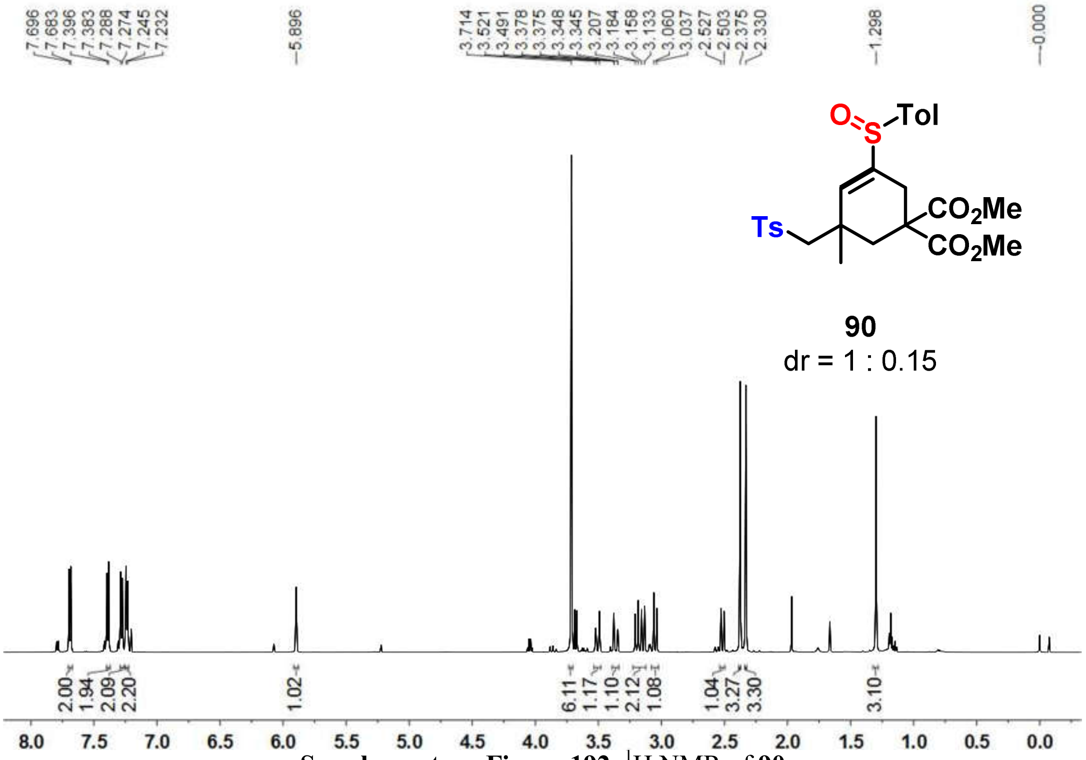

Supplementary Figure 192. ^{1}H NMR of **90**

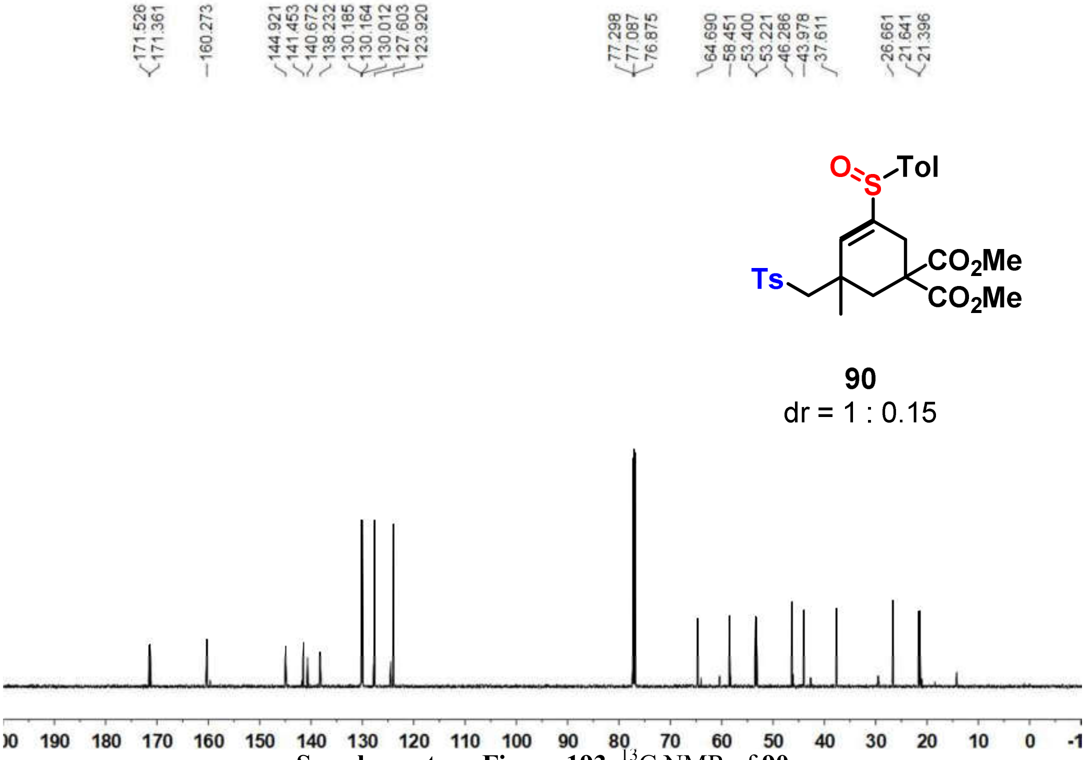

Supplementary Figure 193. ^{13}C NMR of **90**

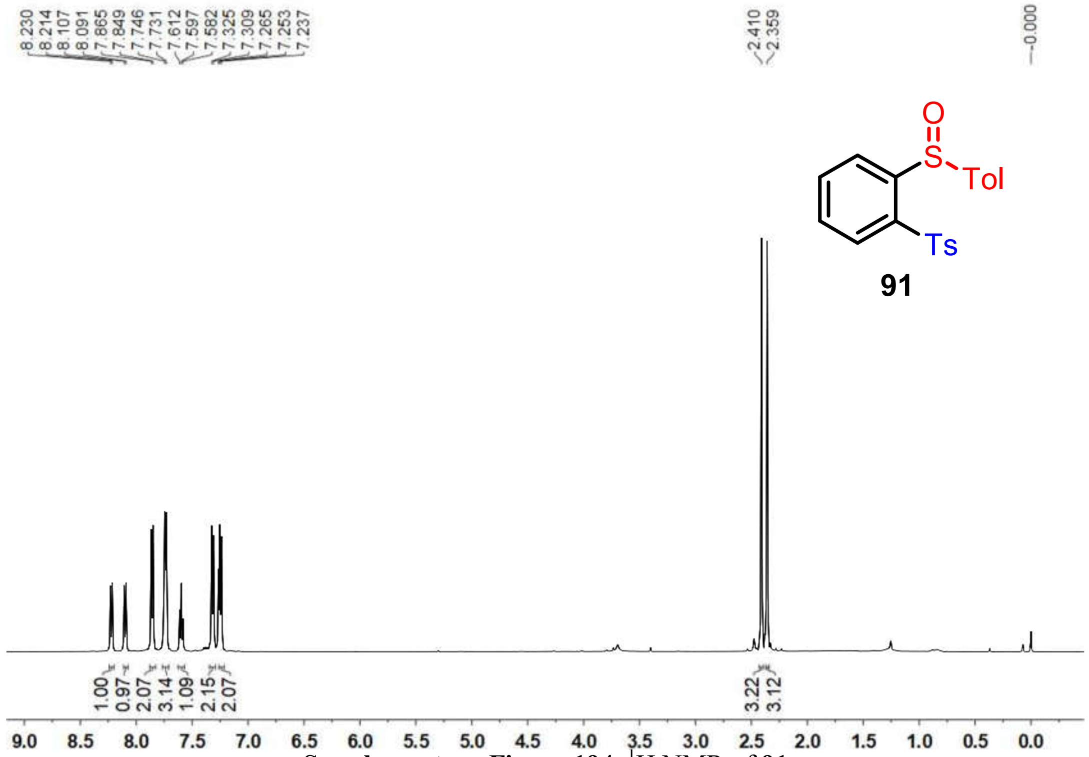

Supplementary Figure 194. ^{1}H NMR of **91**

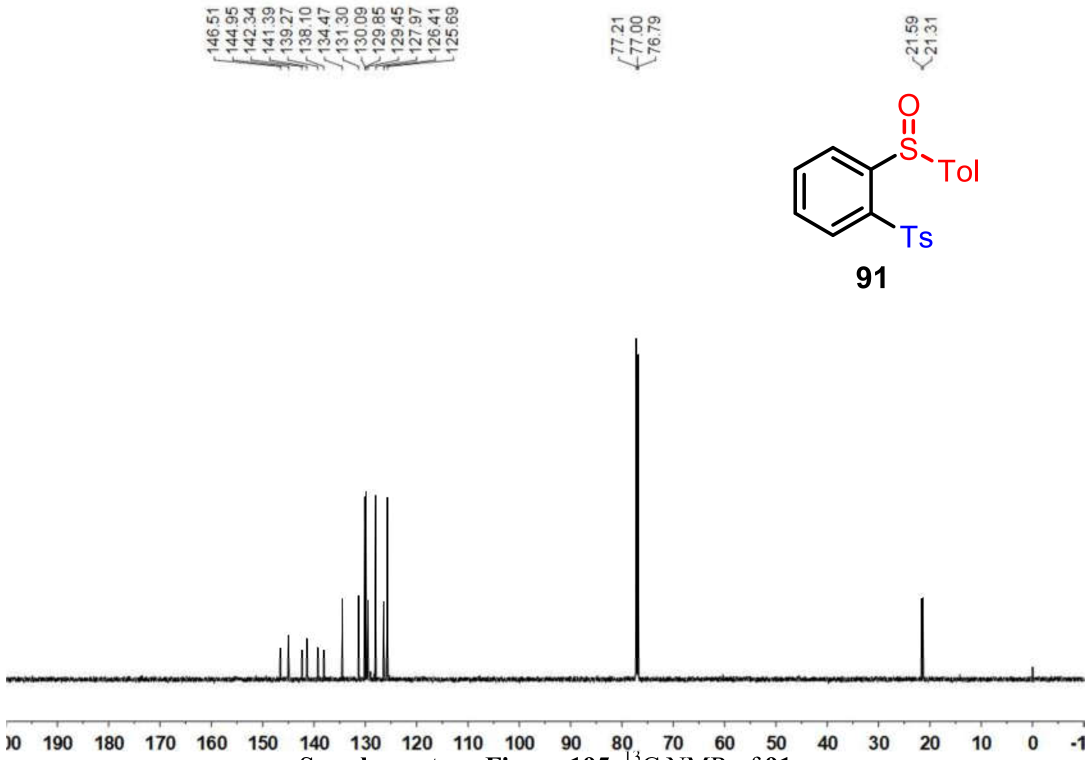

Supplementary Figure 195. ^{13}C NMR of **91**

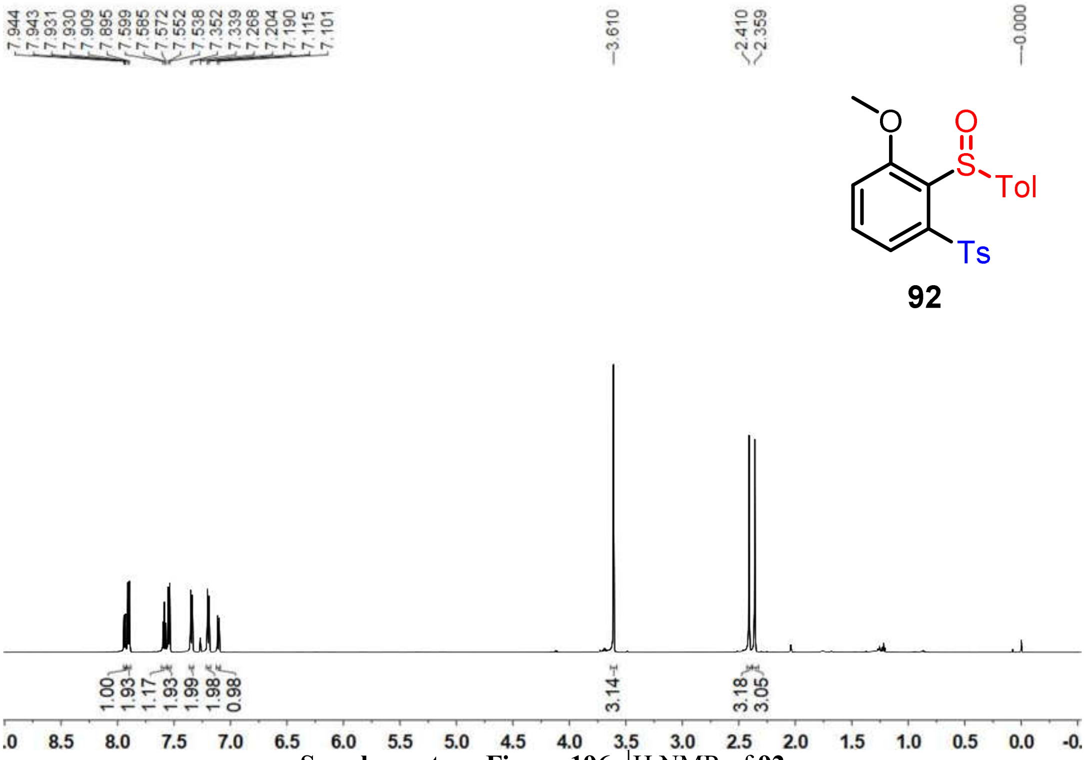

Supplementary Figure 196. ^{1}H NMR of **92**

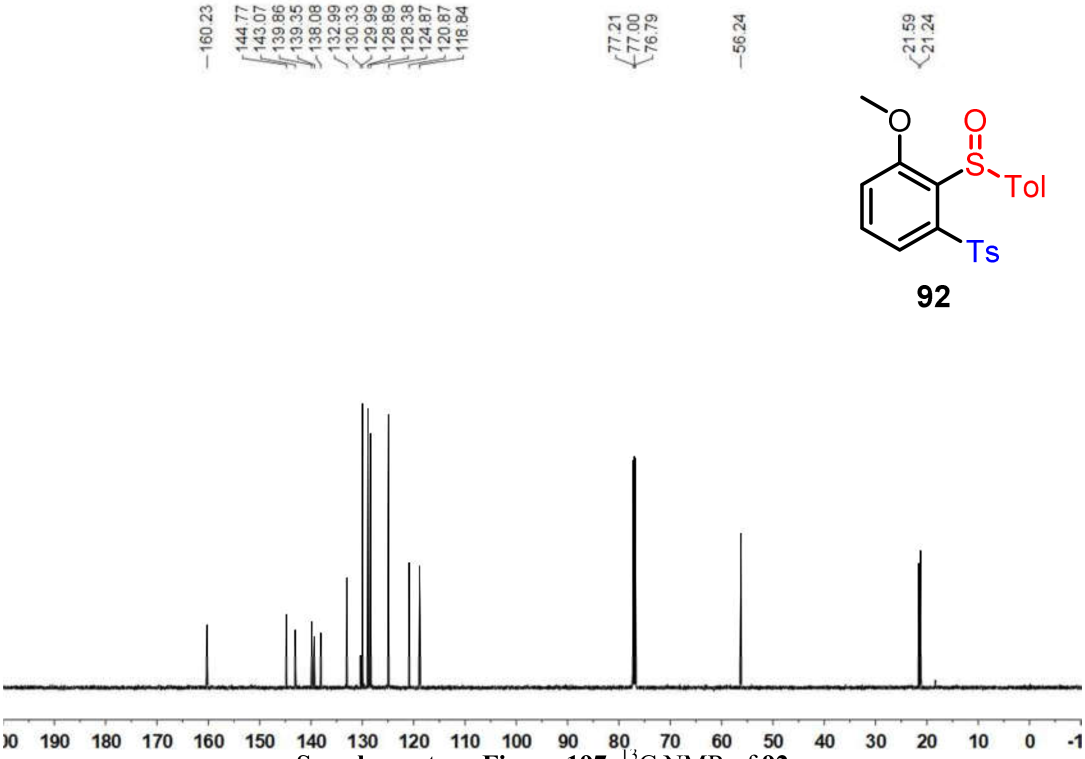

Supplementary Figure 197. ^{13}C NMR of **92**

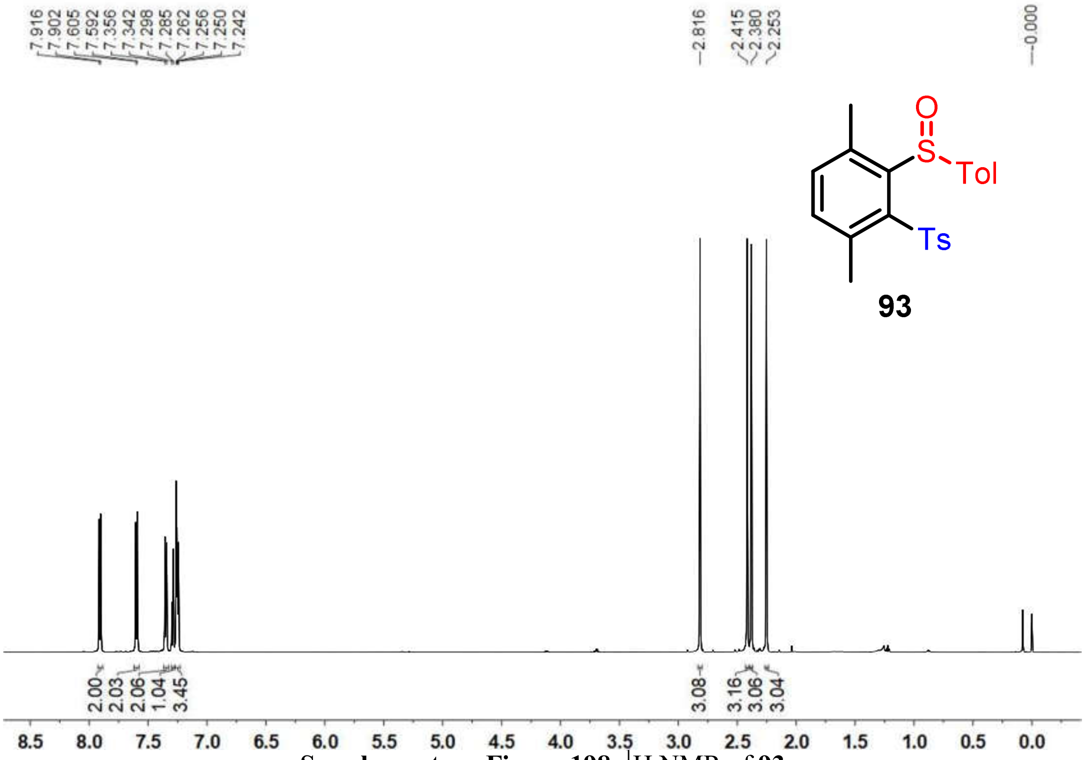

Supplementary Figure 198. ^{1}H NMR of **93**

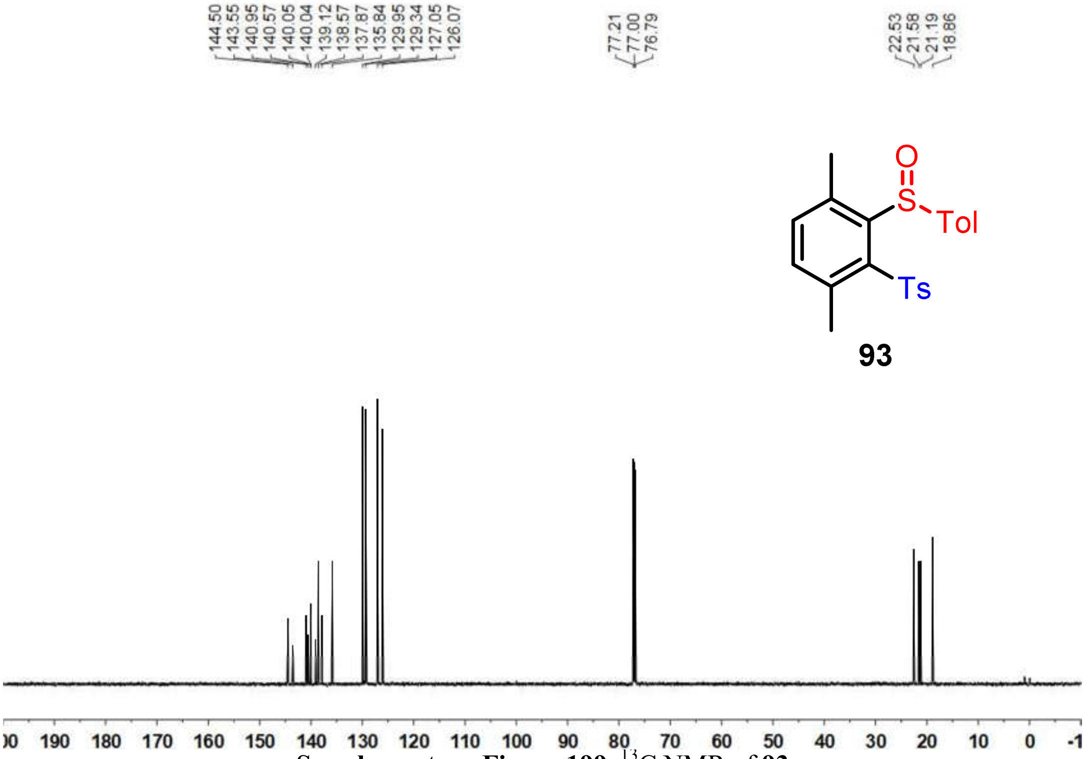

Supplementary Figure 199. ^{13}C NMR of **93**

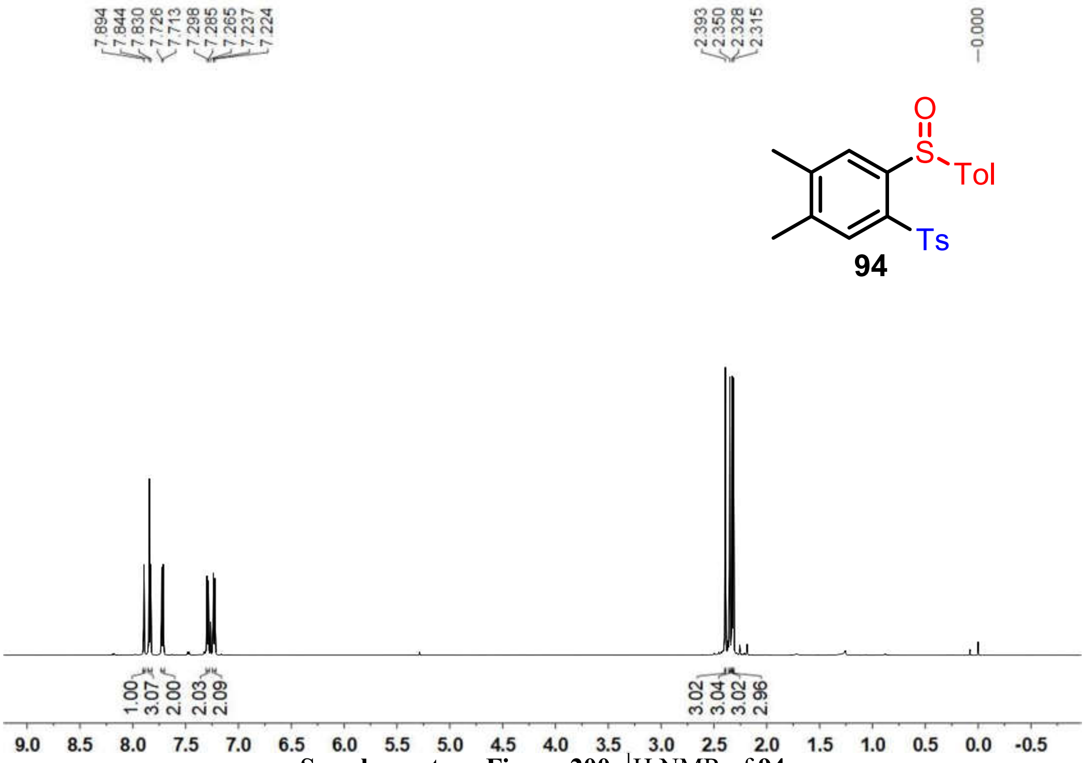

Supplementary Figure 200. ^{1}H NMR of **94**

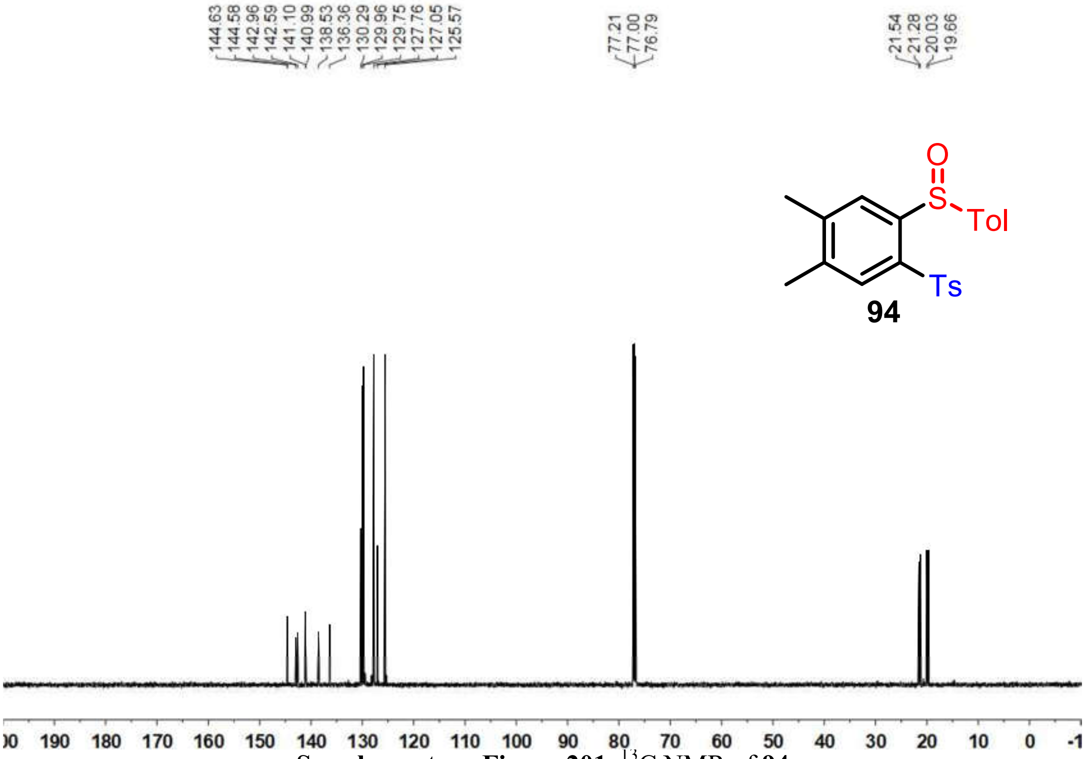

Supplementary Figure 201. ^{13}C NMR of **94**

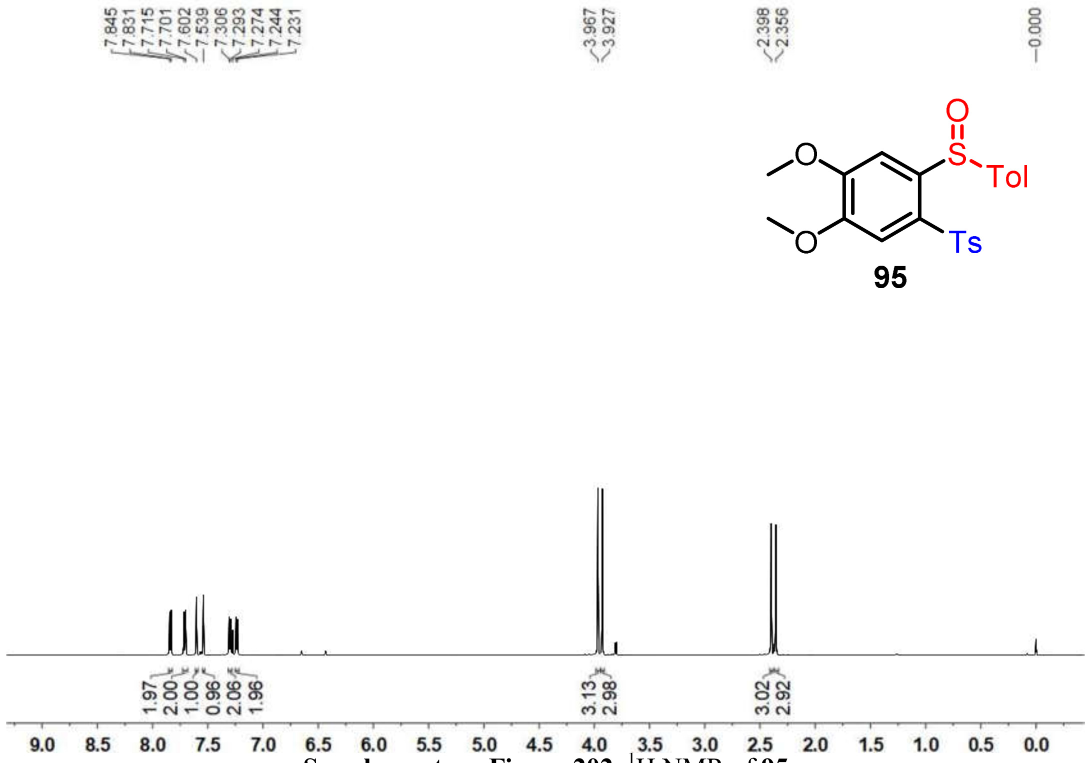

Supplementary Figure 202. ^{1}H NMR of **95**

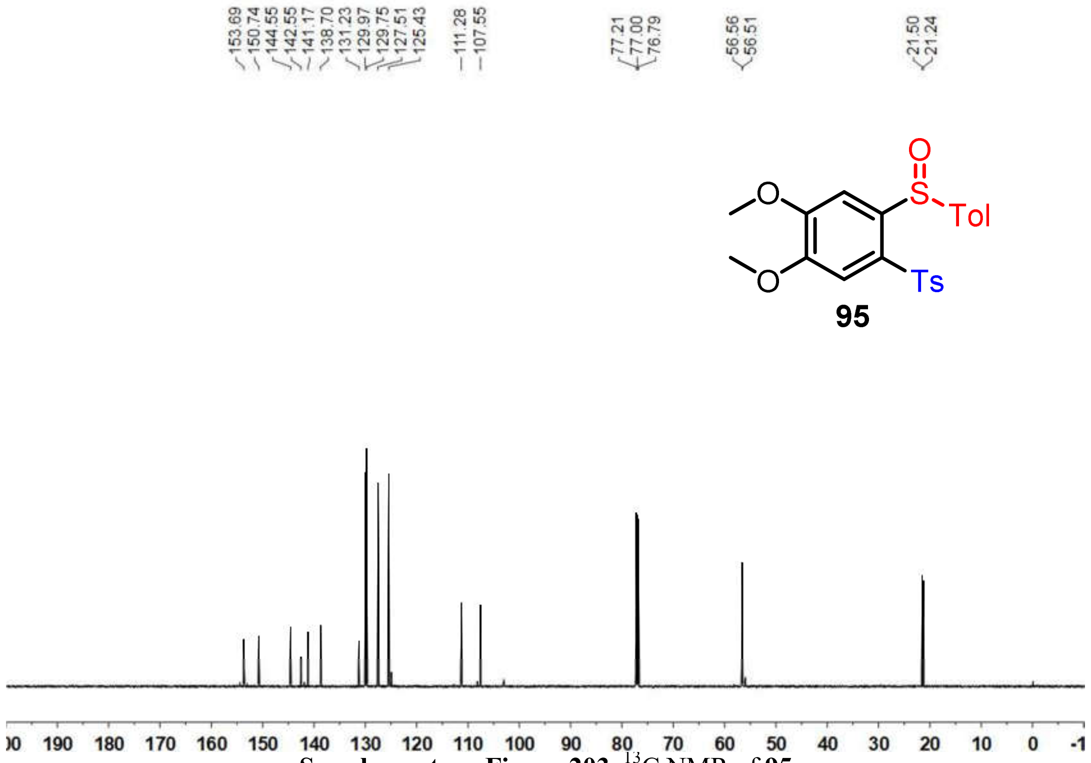

Supplementary Figure 203. ^{13}C NMR of **95**

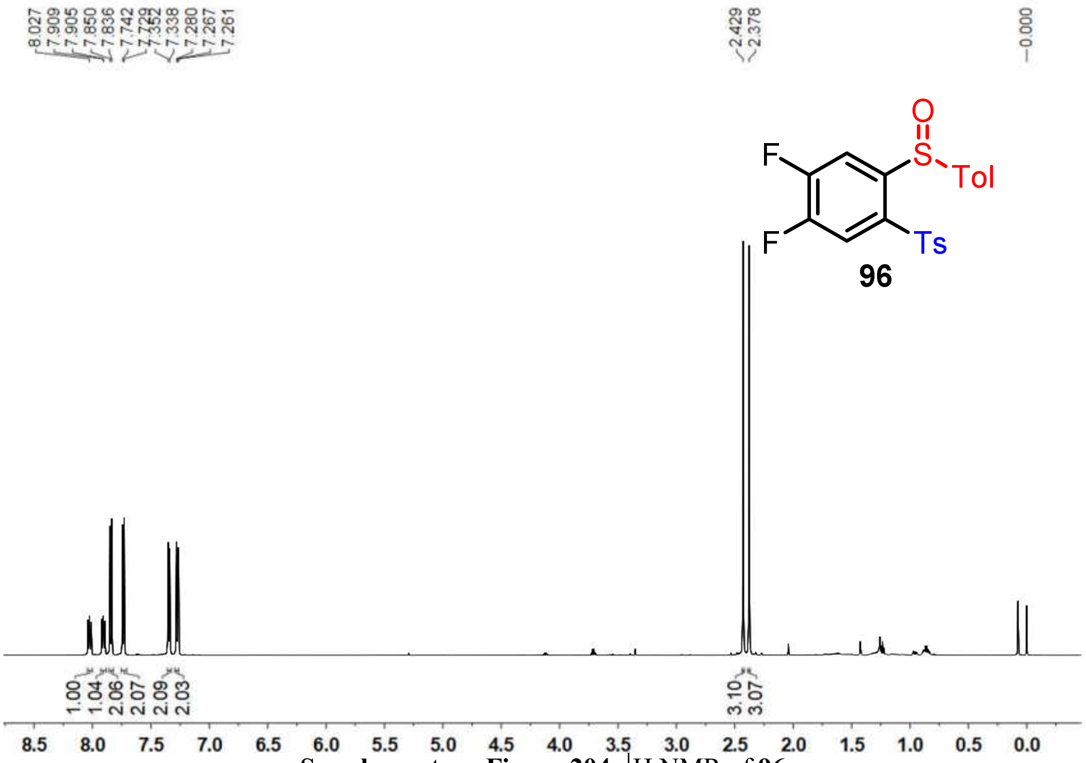

Supplementary Figure 204. ^{1}H NMR of **96**

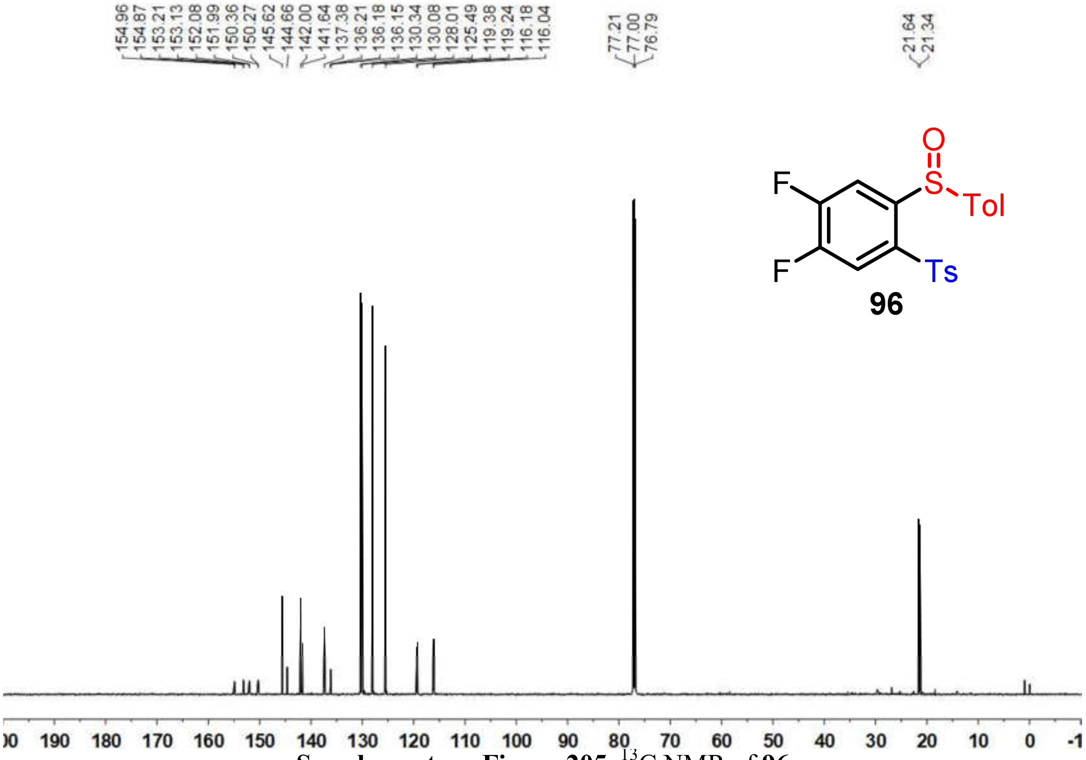

Supplementary Figure 205. ^{13}C NMR of **96**

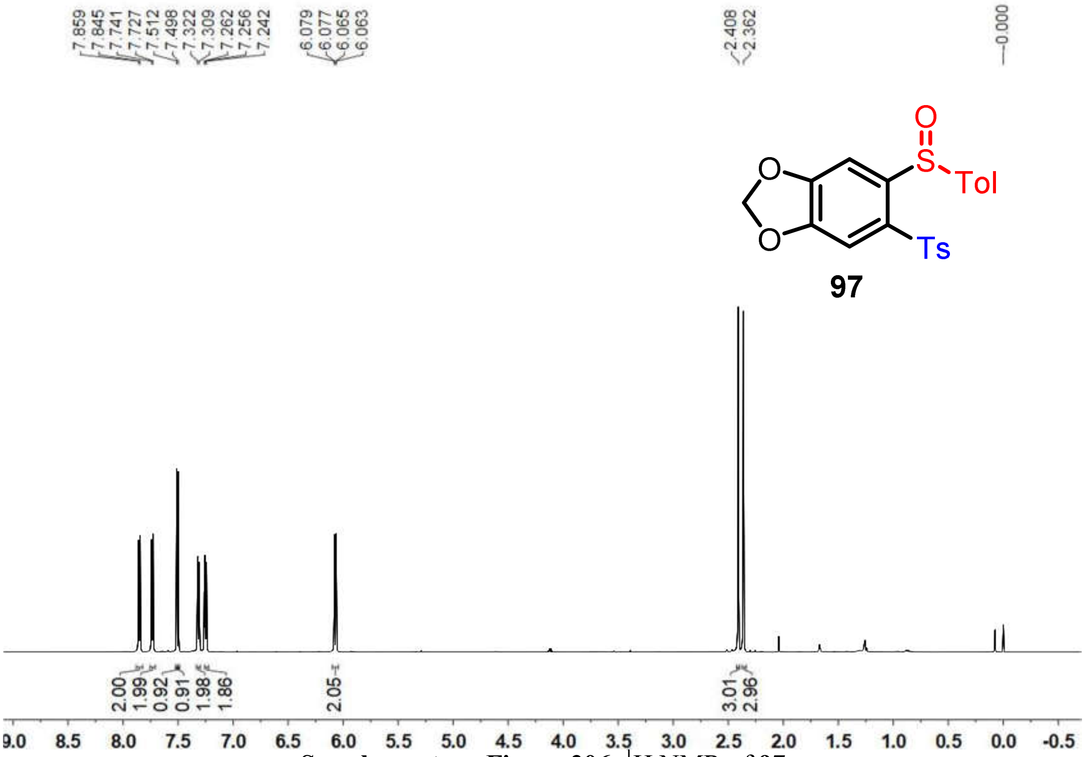

Supplementary Figure 206. ^{1}H NMR of **97**

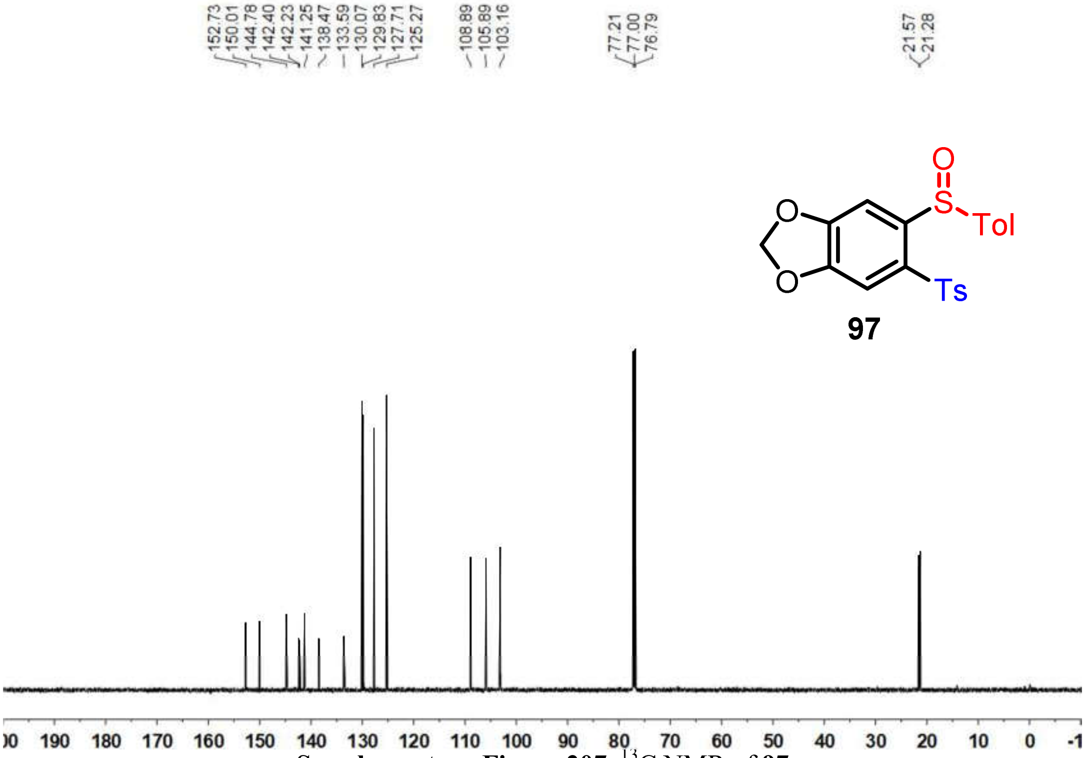

Supplementary Figure 207. ^{13}C NMR of **97**

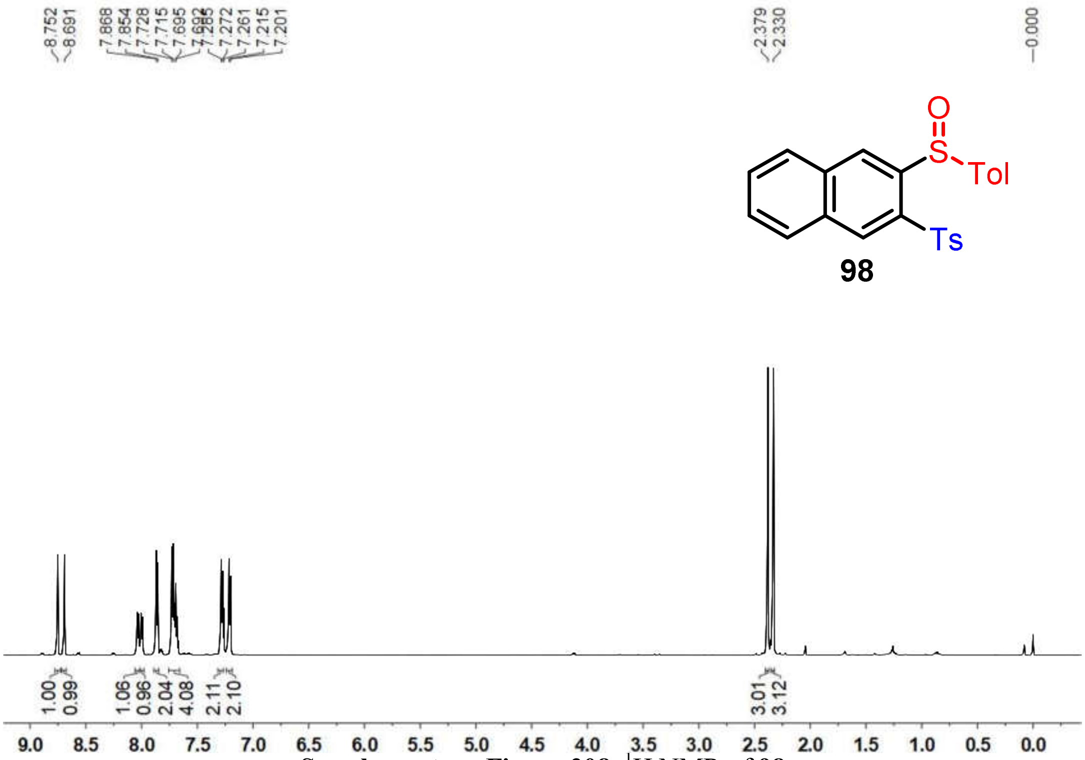

Supplementary Figure 208. ^{1}H NMR of **98**

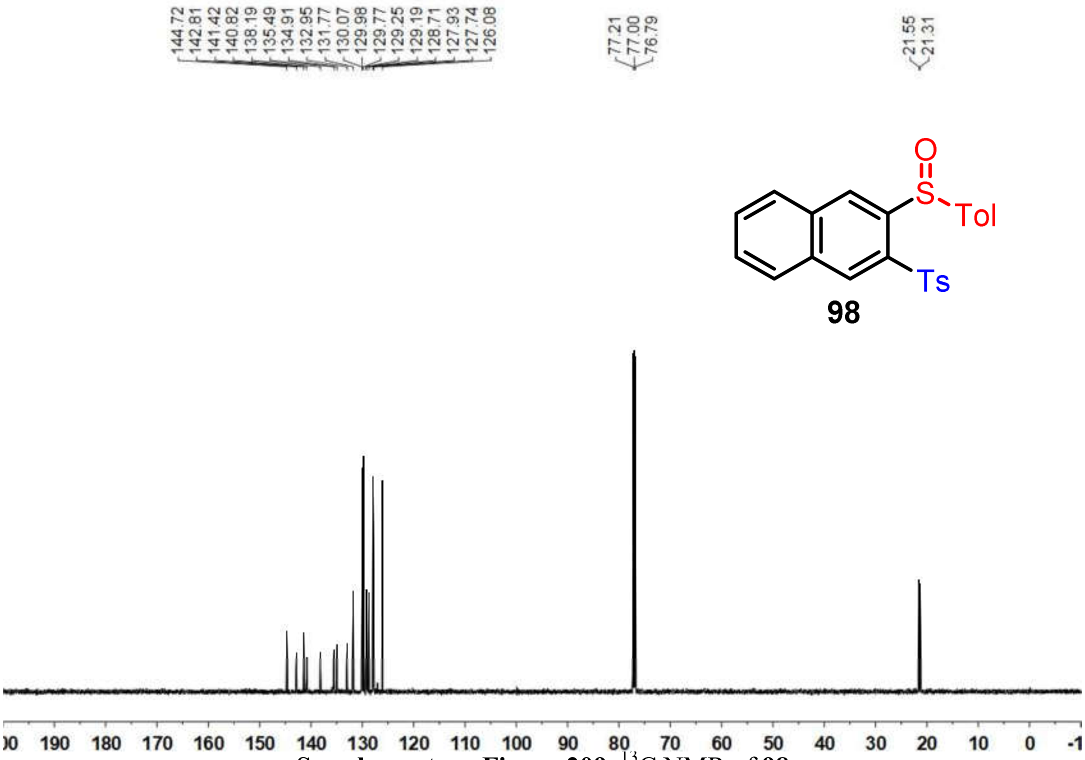

Supplementary Figure 209. ^{13}C NMR of **98**

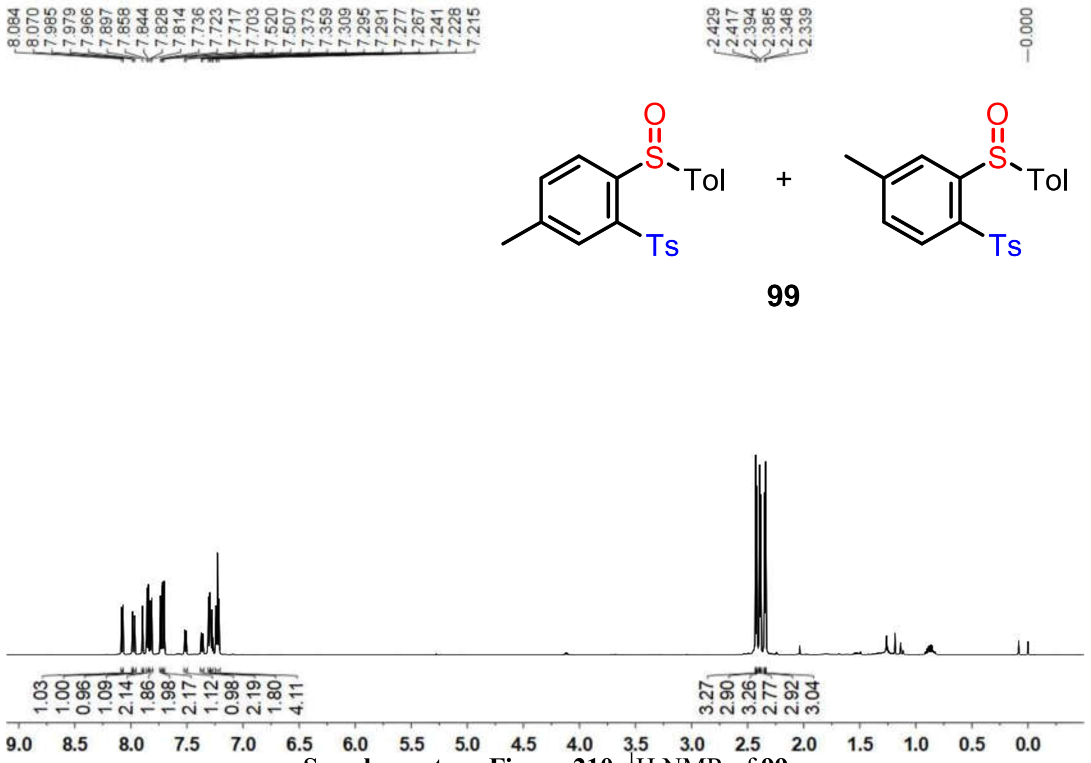

Supplementary Figure 210. ^{1}H NMR of **99**

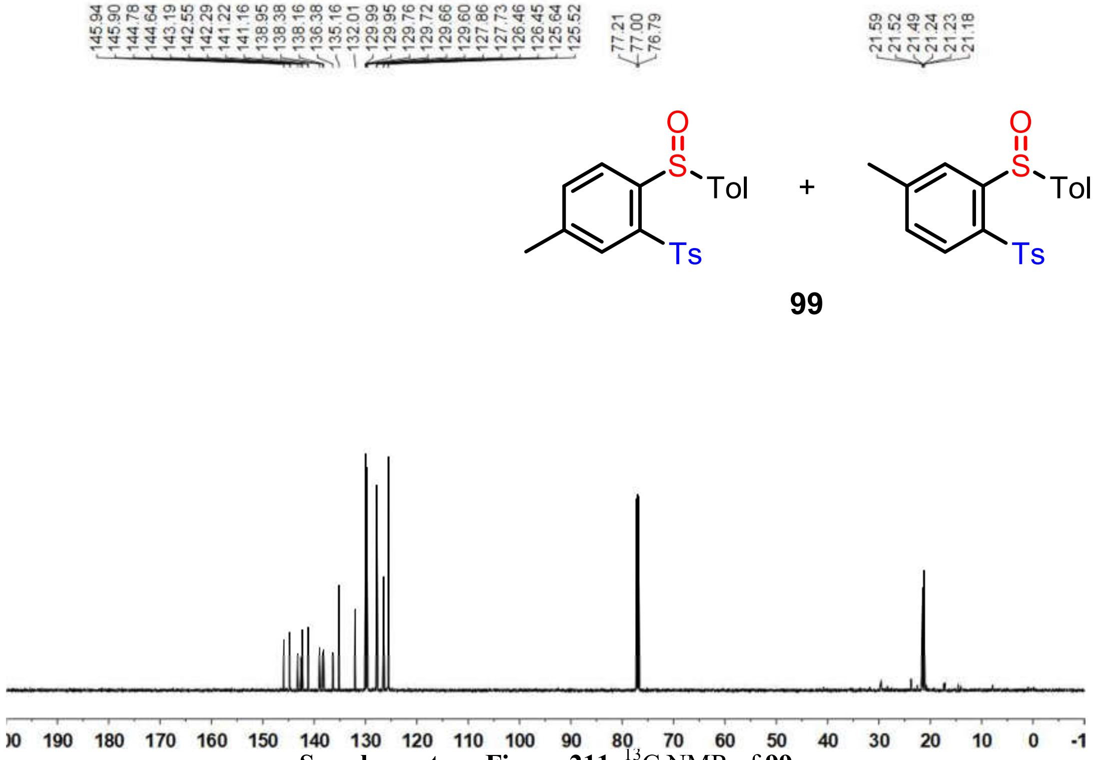

Supplementary Figure 211. ^{13}C NMR of **99**

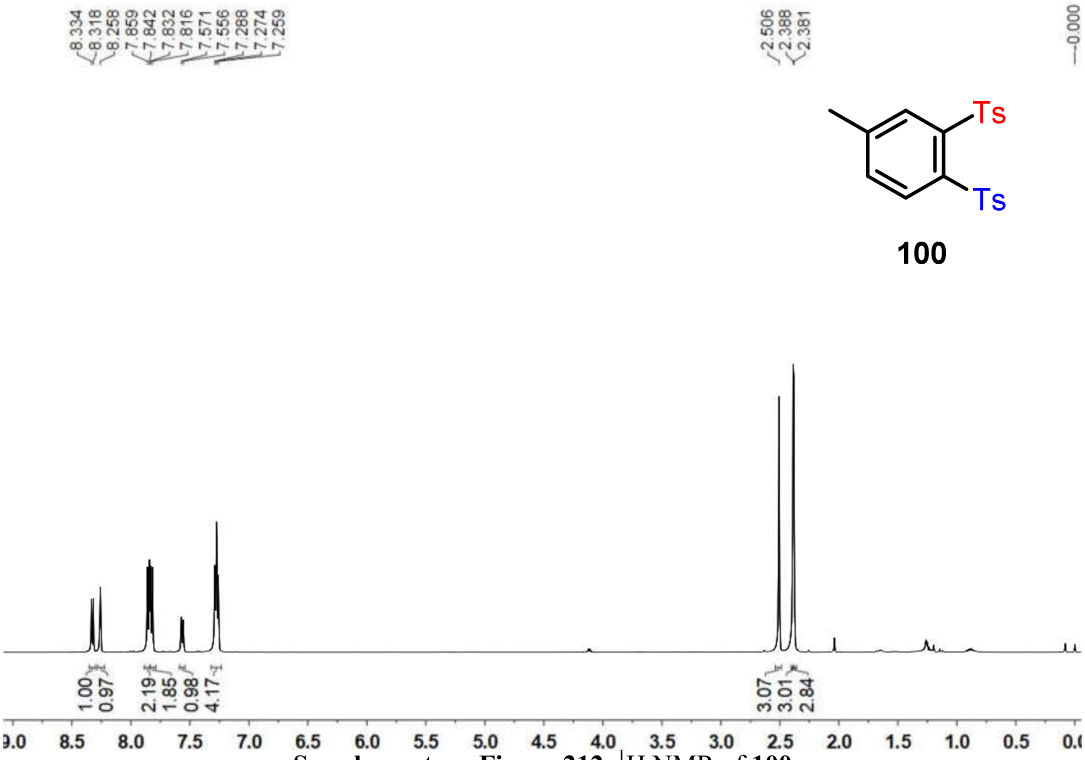

Supplementary Figure 212. ^{1}H NMR of **100**

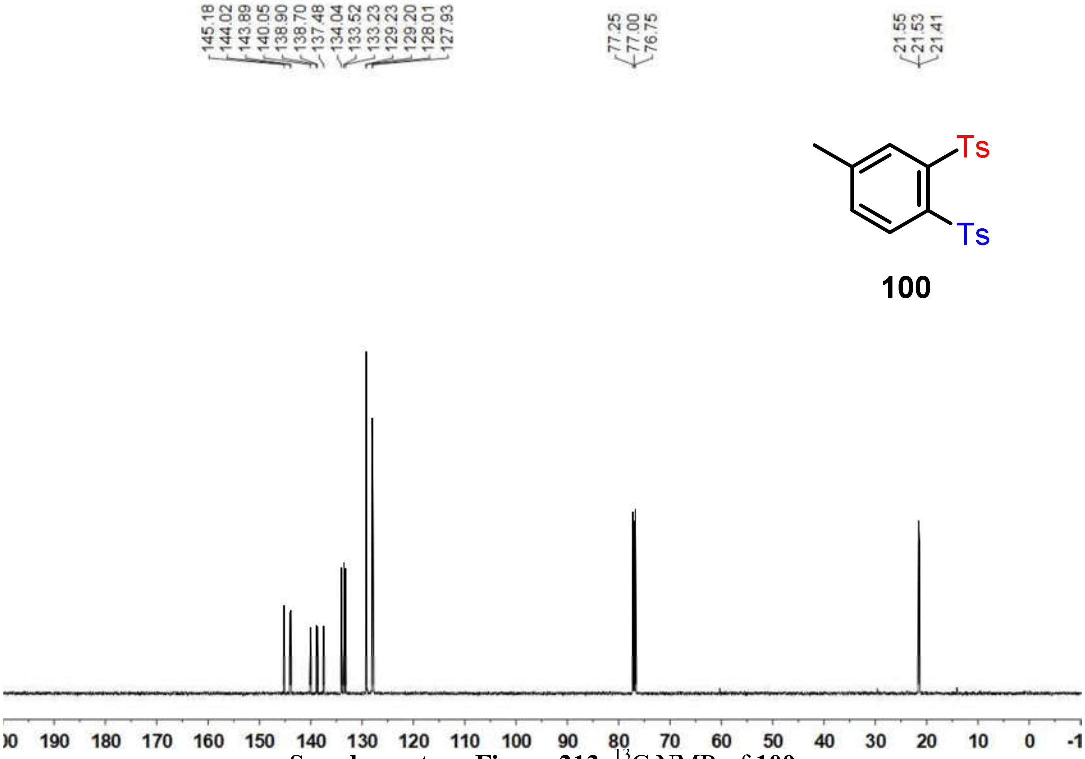

Supplementary Figure 213. ^{13}C NMR of **100**

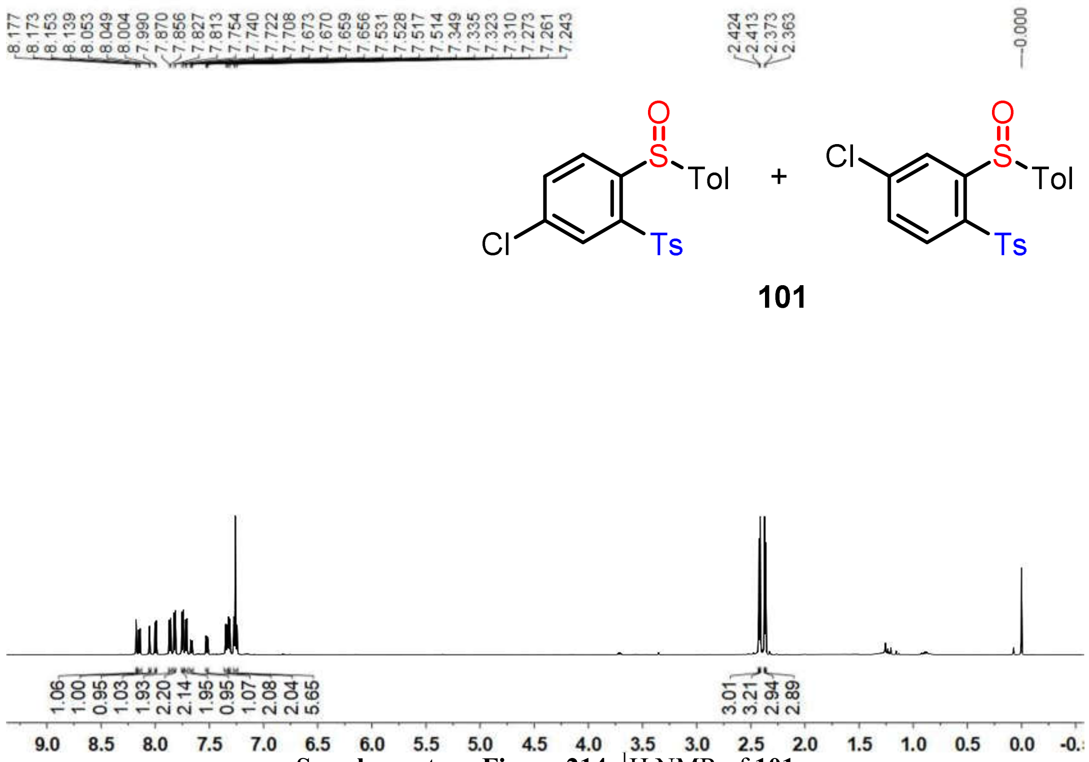

Supplementary Figure 214. ^{1}H NMR of **101**

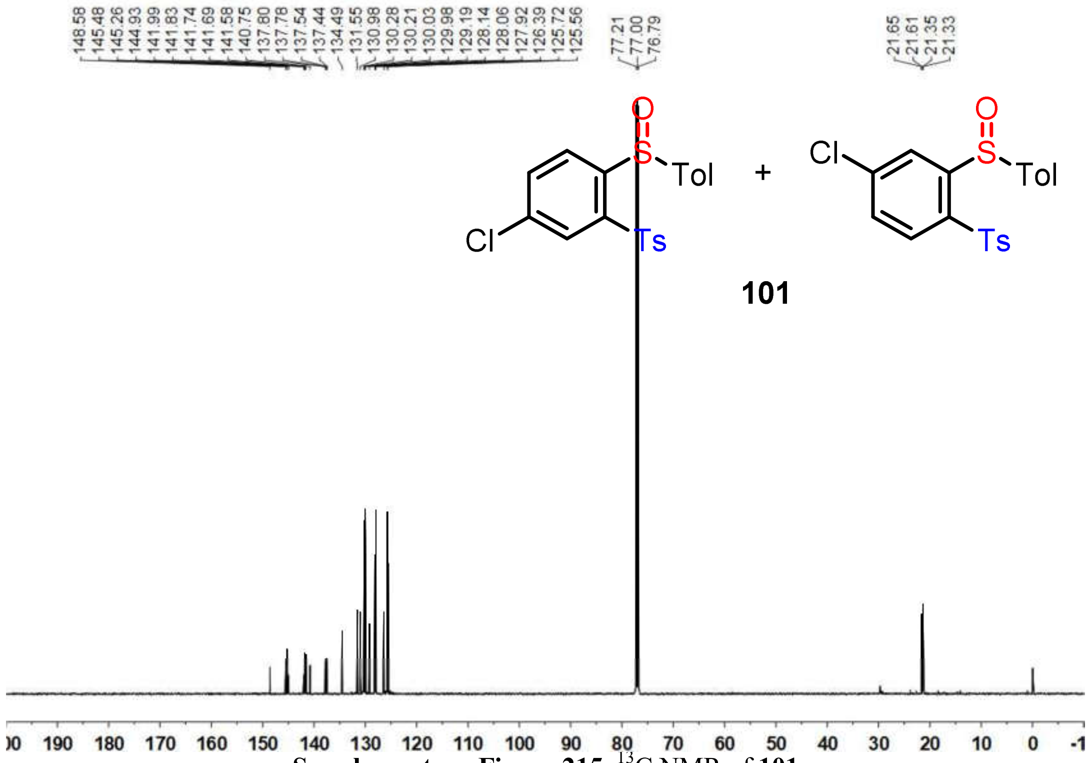

Supplementary Figure 215. ^{13}C NMR of **101**

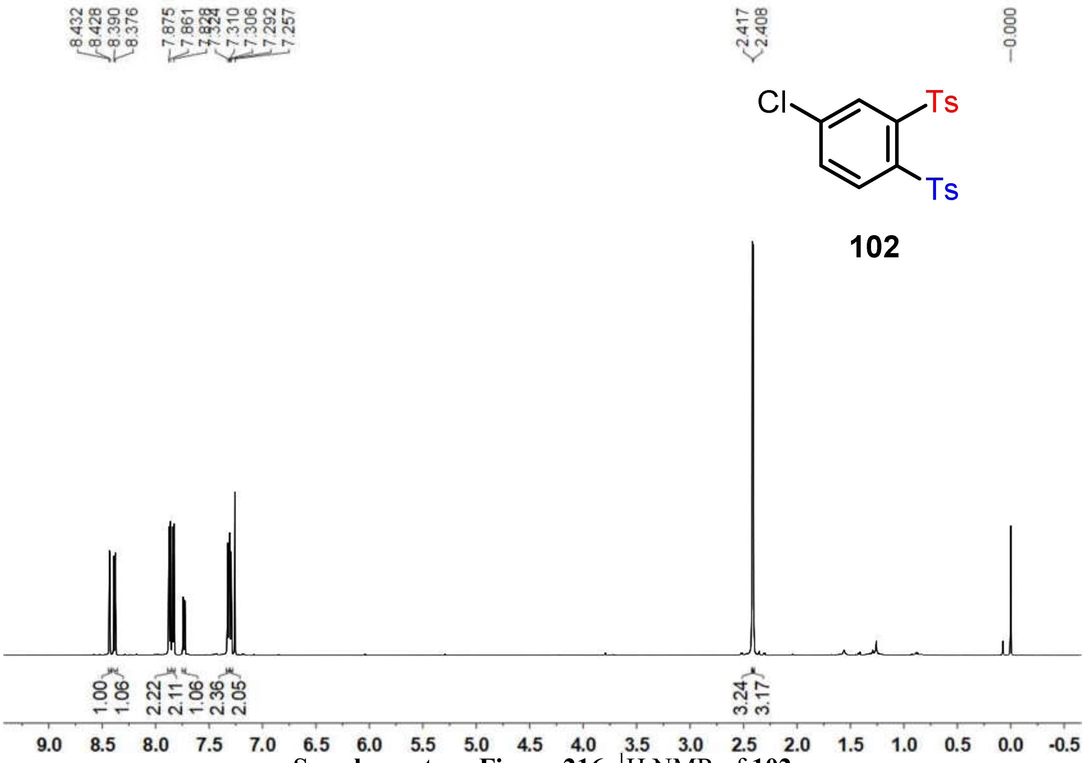

Supplementary Figure 216. ^{1}H NMR of **102**

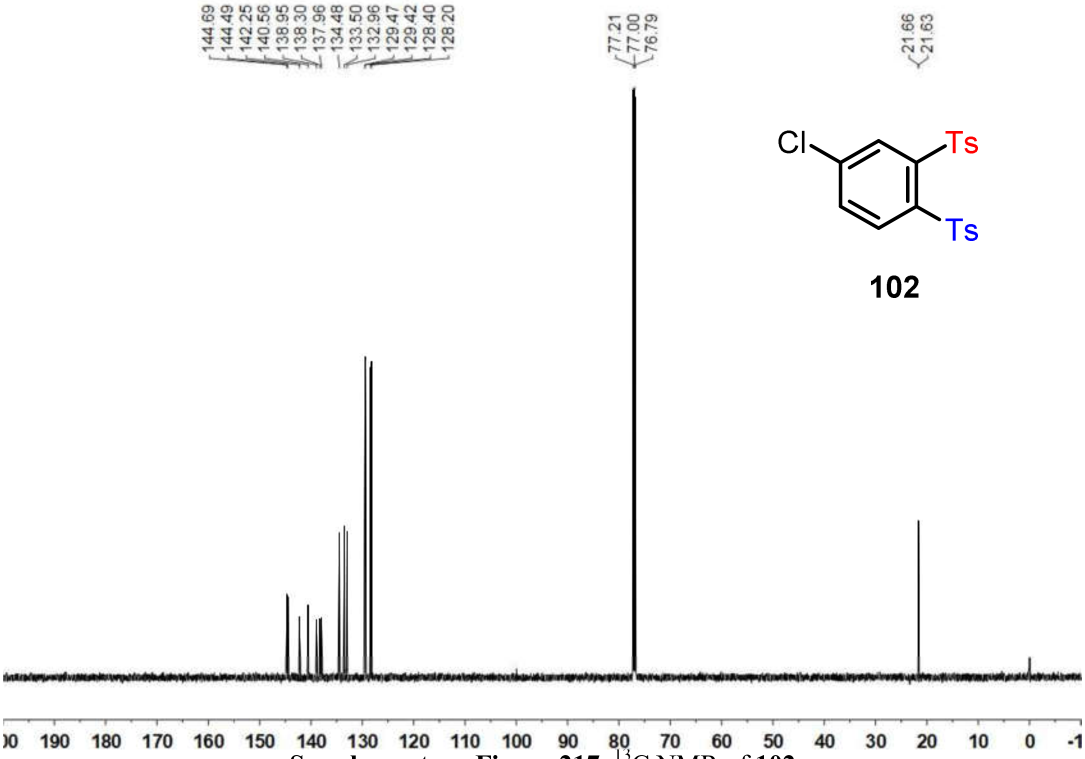

Supplementary Figure 217. ^{13}C NMR of **102**

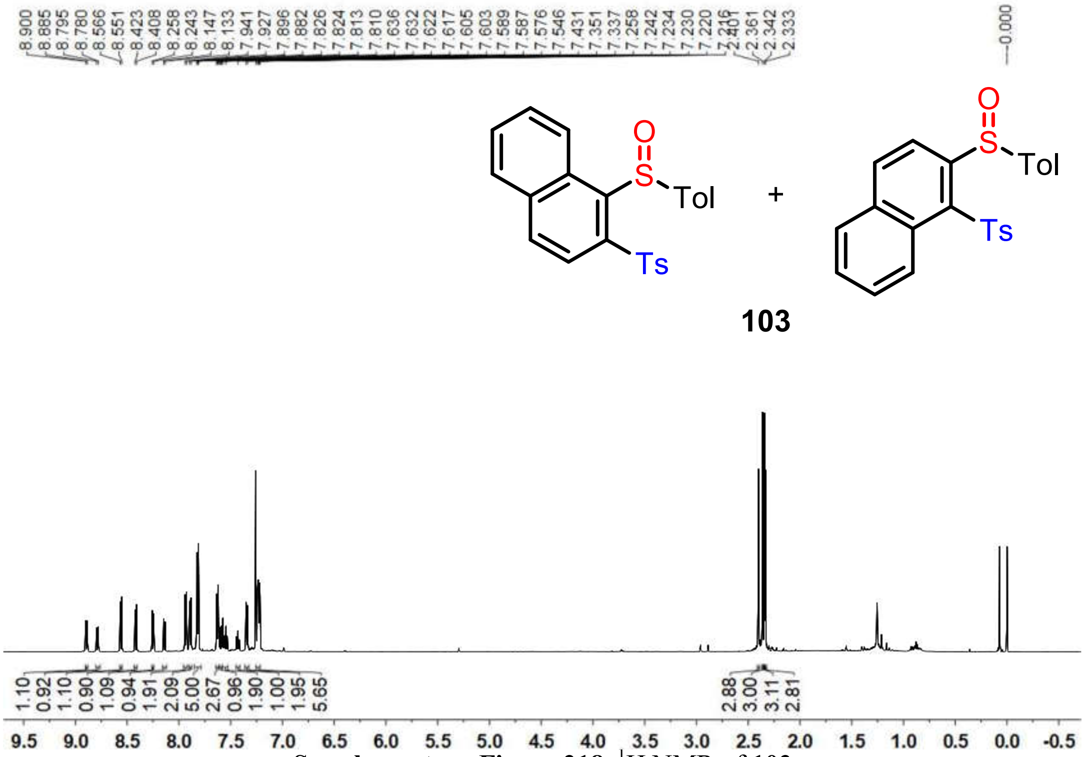

Supplementary Figure 218. ^{1}H NMR of **103**

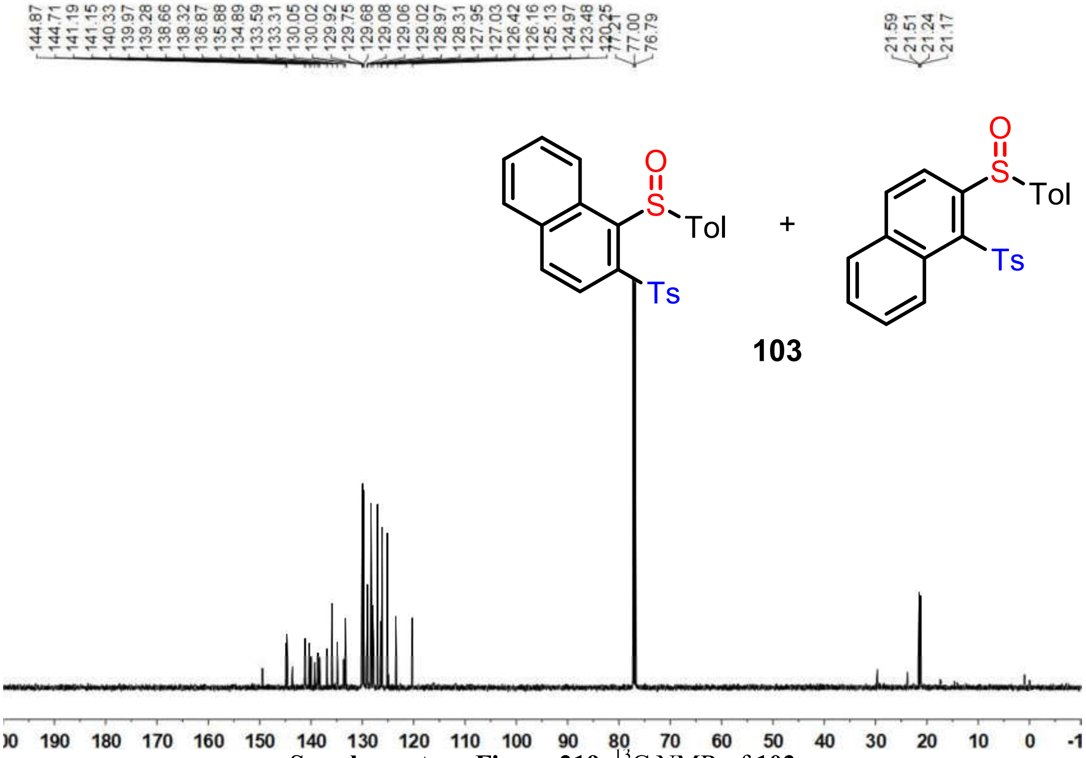

Supplementary Figure 219. ^{13}C NMR of **103**

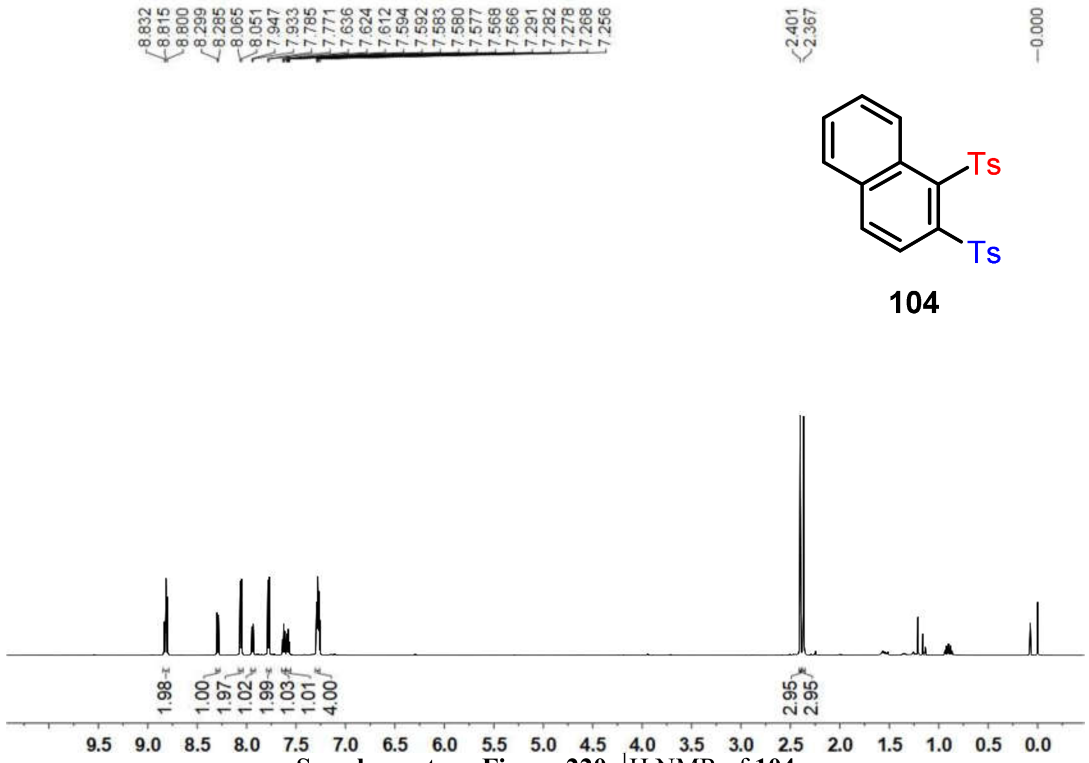

Supplementary Figure 220. ^{1}H NMR of **104**

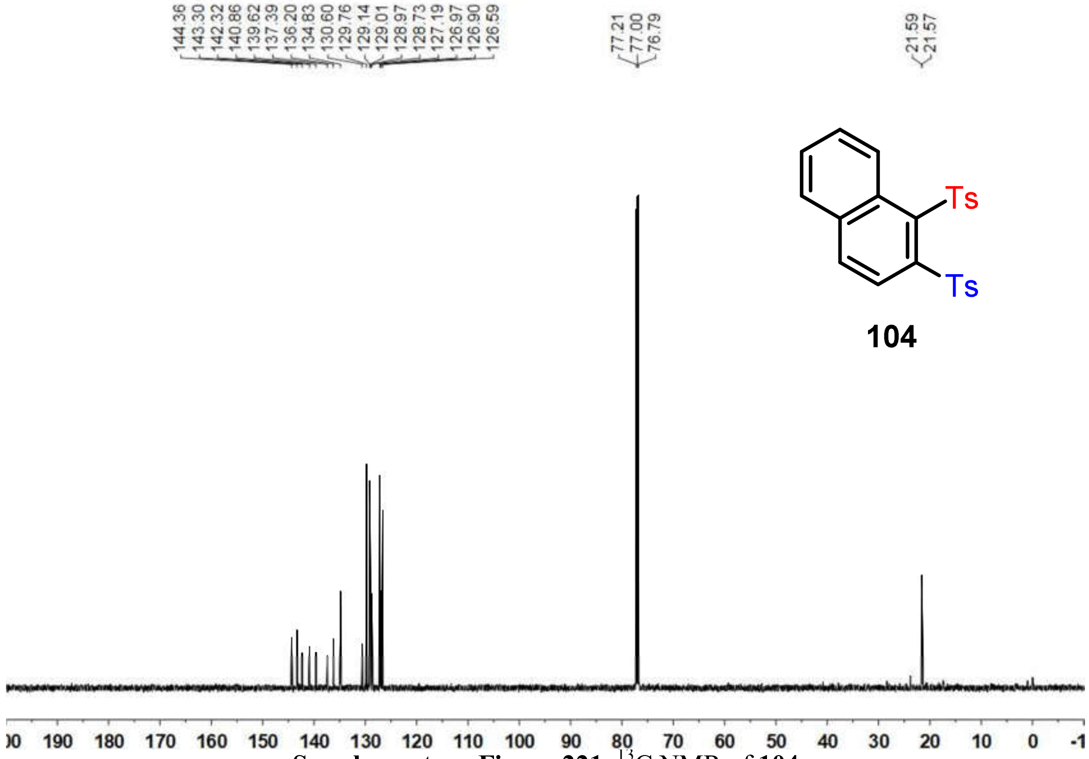

Supplementary Figure 221. ^{13}C NMR of **104**

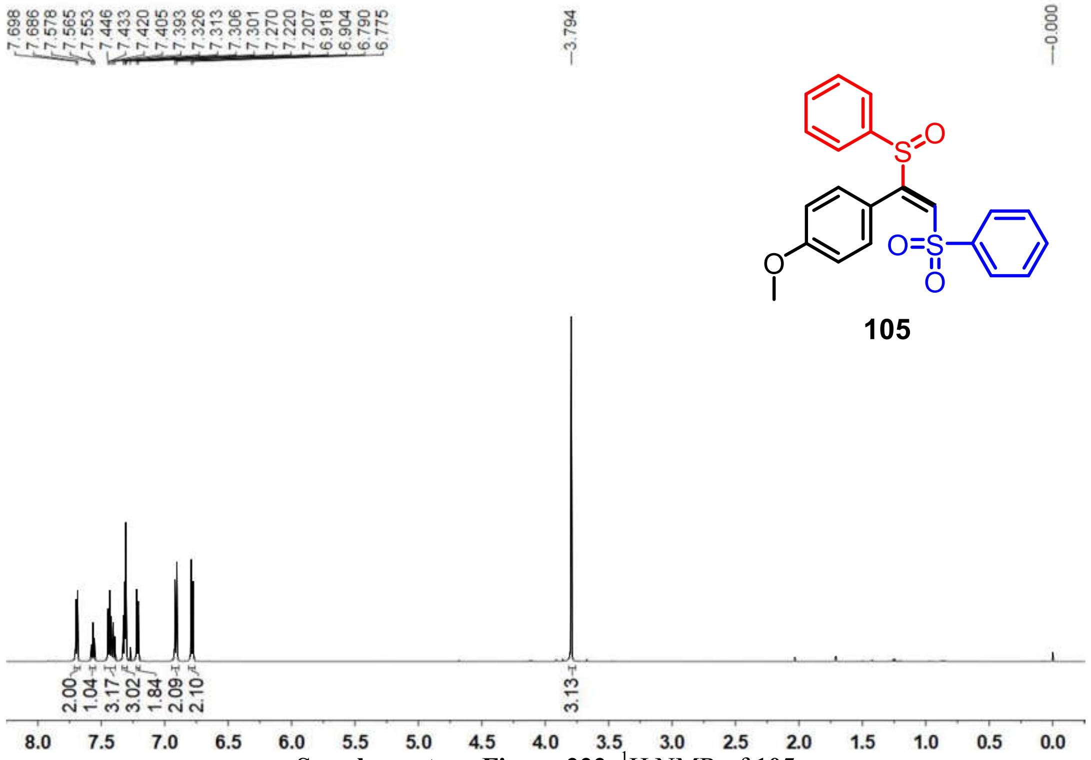

Supplementary Figure 222. ^{1}H NMR of **105**

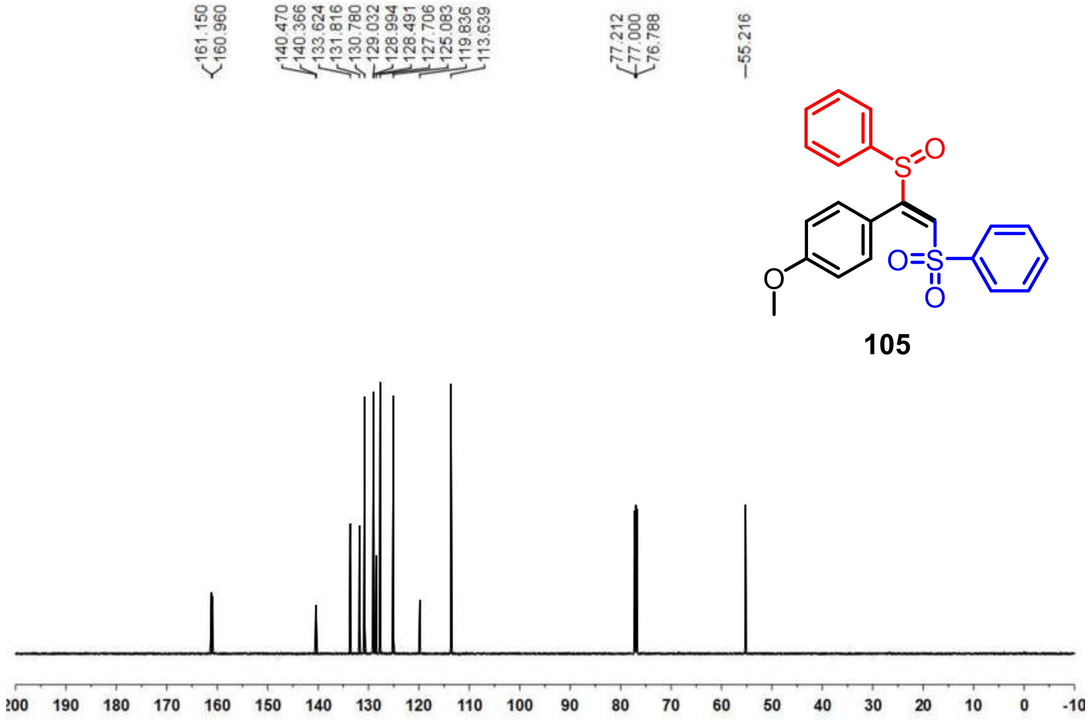

Supplementary Figure 223. ^{13}C NMR of **105**

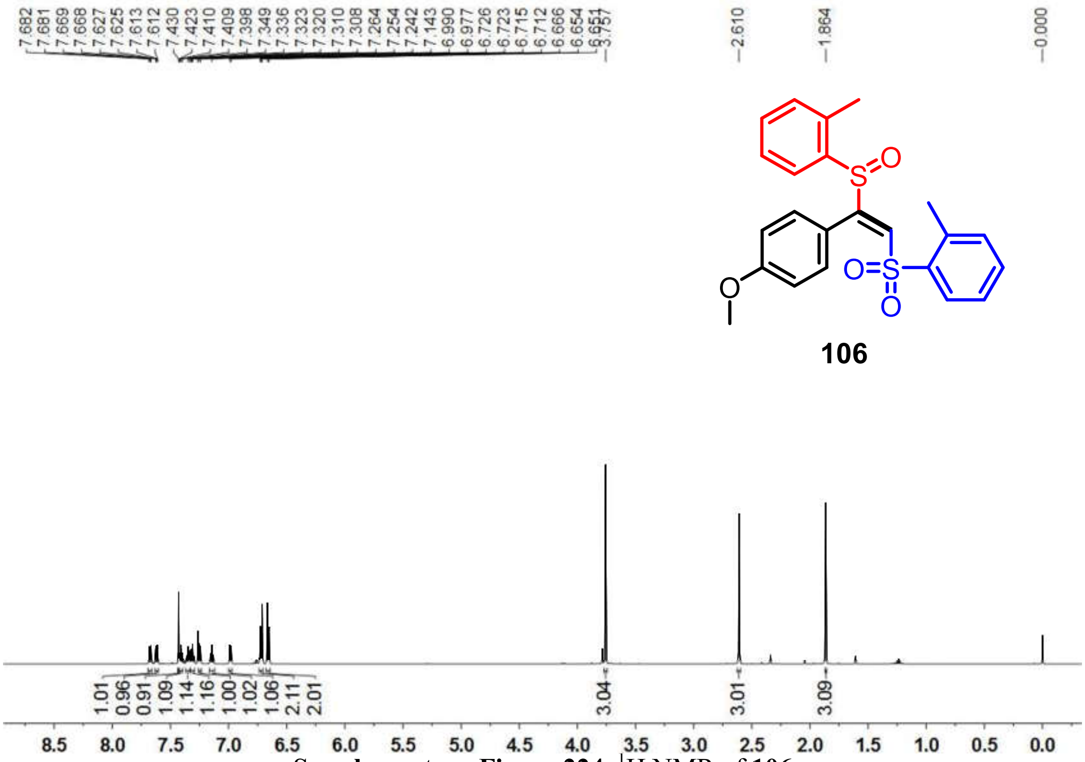

Supplementary Figure 224. ^{1}H NMR of **106**

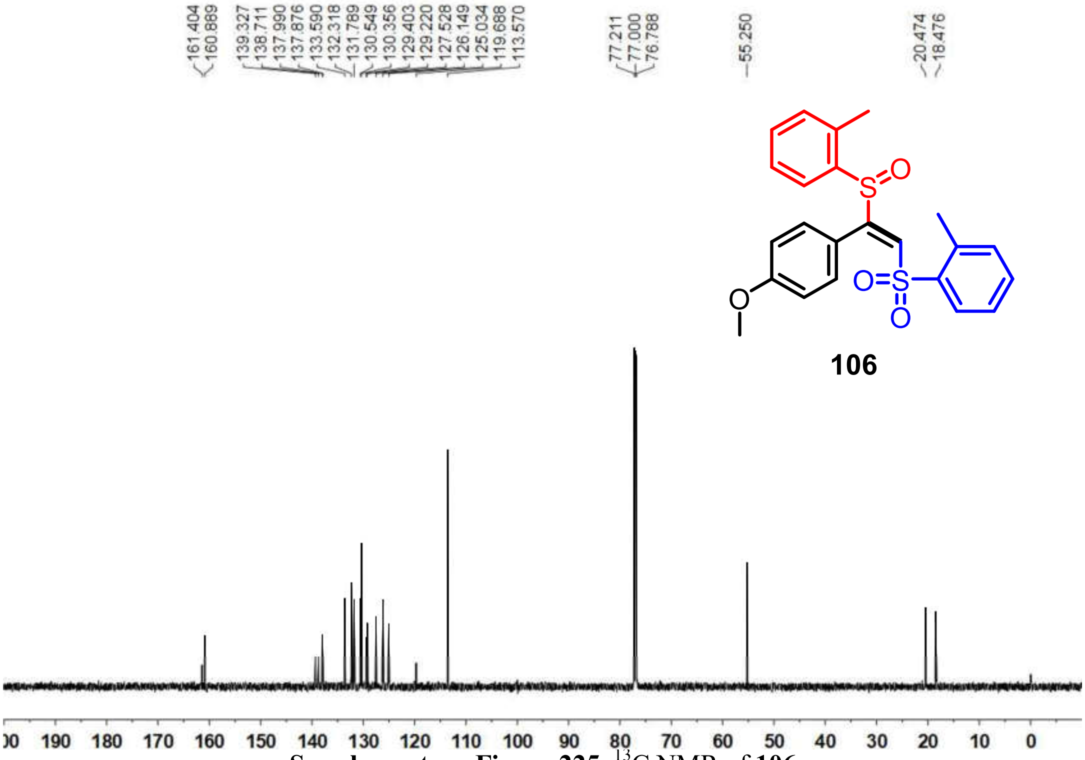

Supplementary Figure 225. ^{13}C NMR of **106**

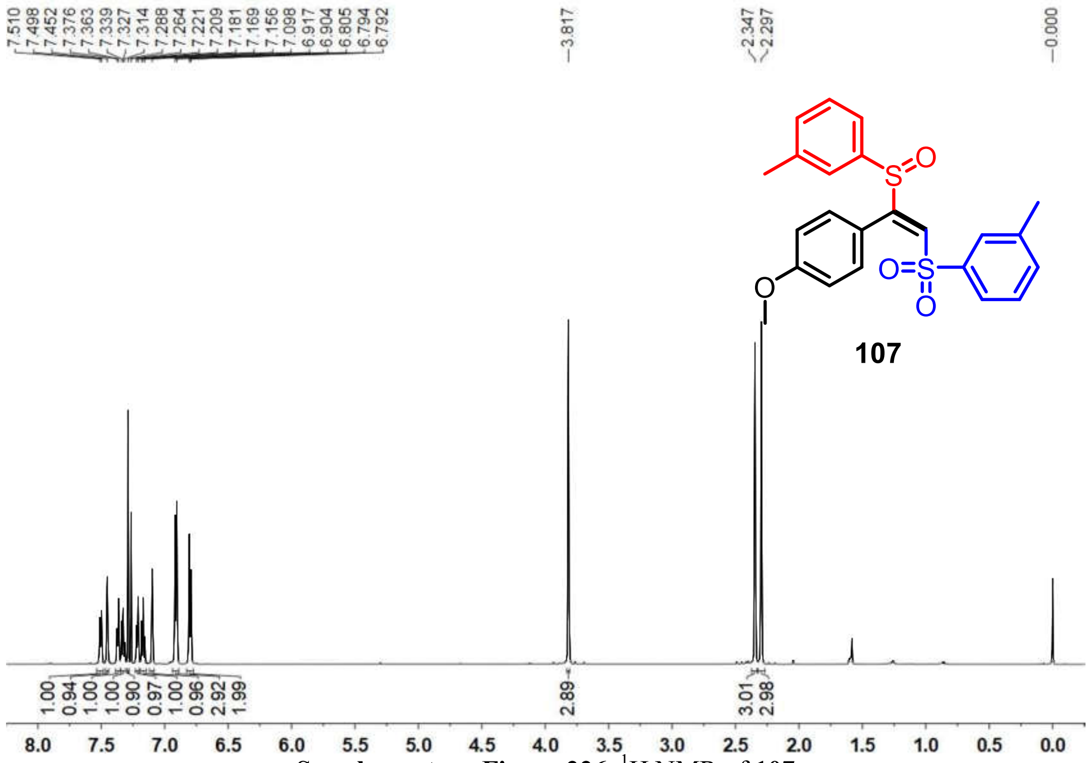

Supplementary Figure 226. ^{1}H NMR of **107**

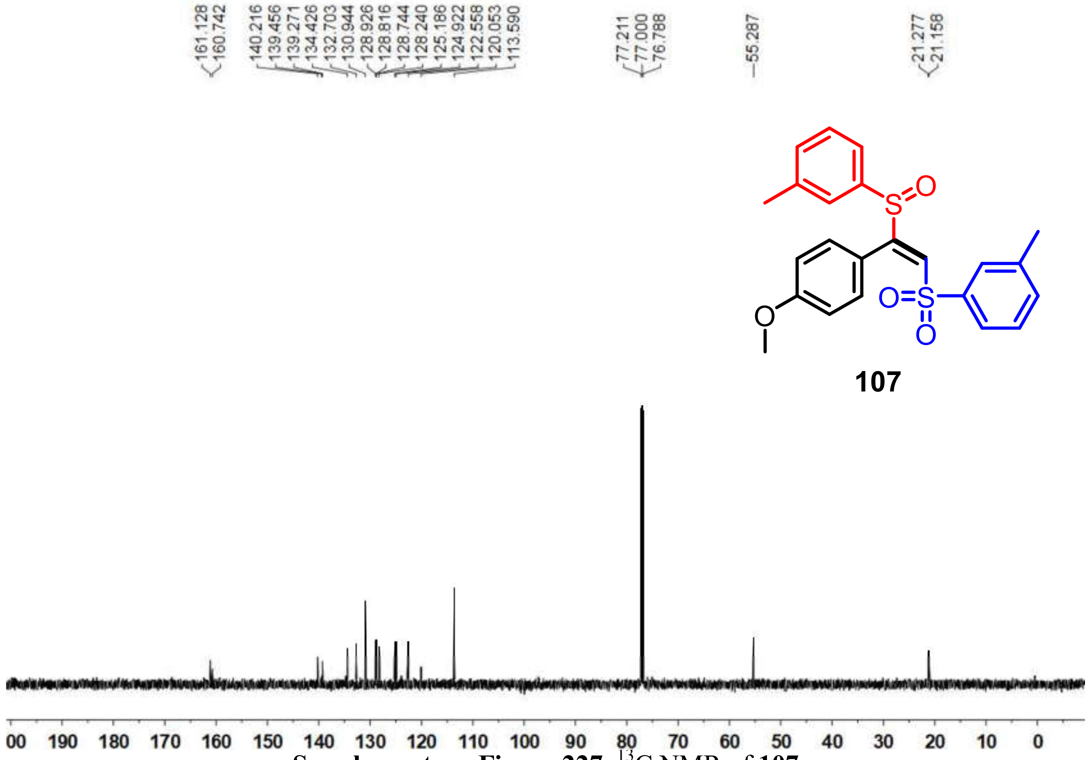

Supplementary Figure 227. ^{13}C NMR of **107**

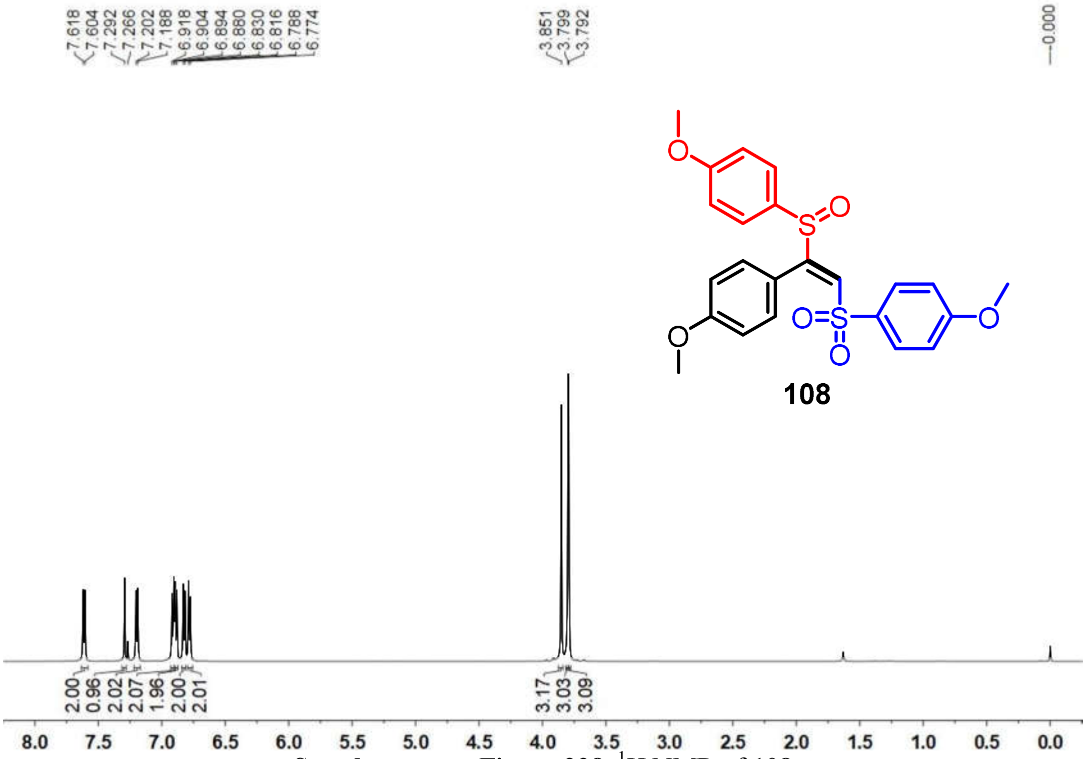

Supplementary Figure 228. ^{1}H NMR of **108**

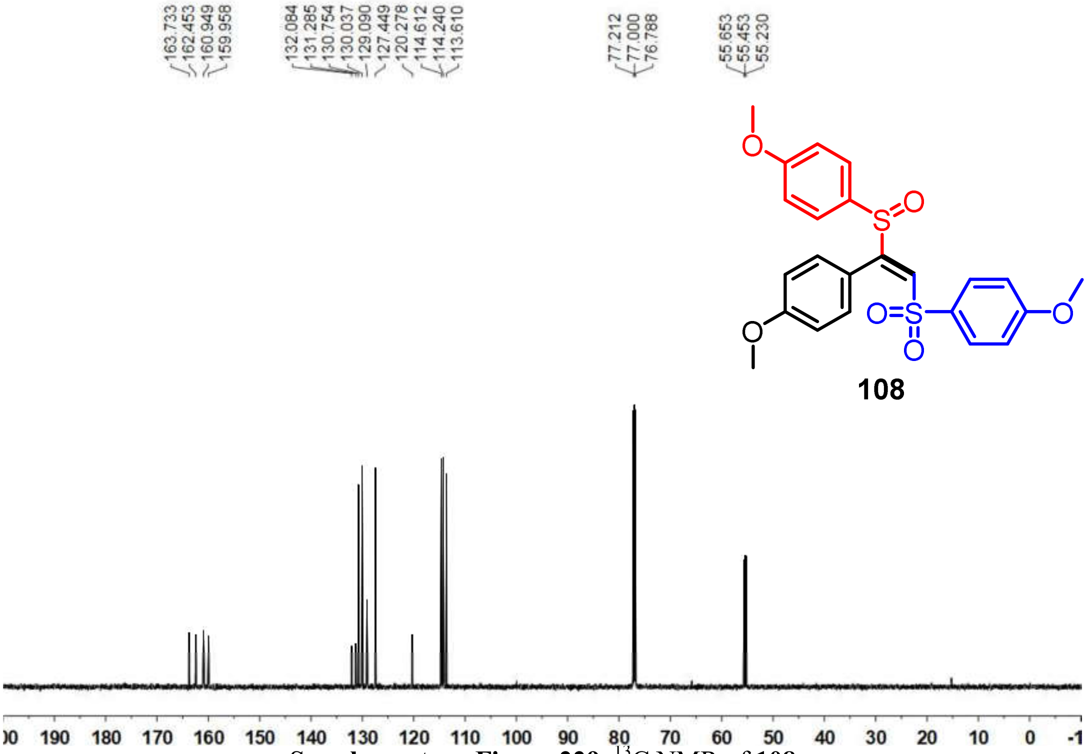

Supplementary Figure 229. ^{13}C NMR of **108**

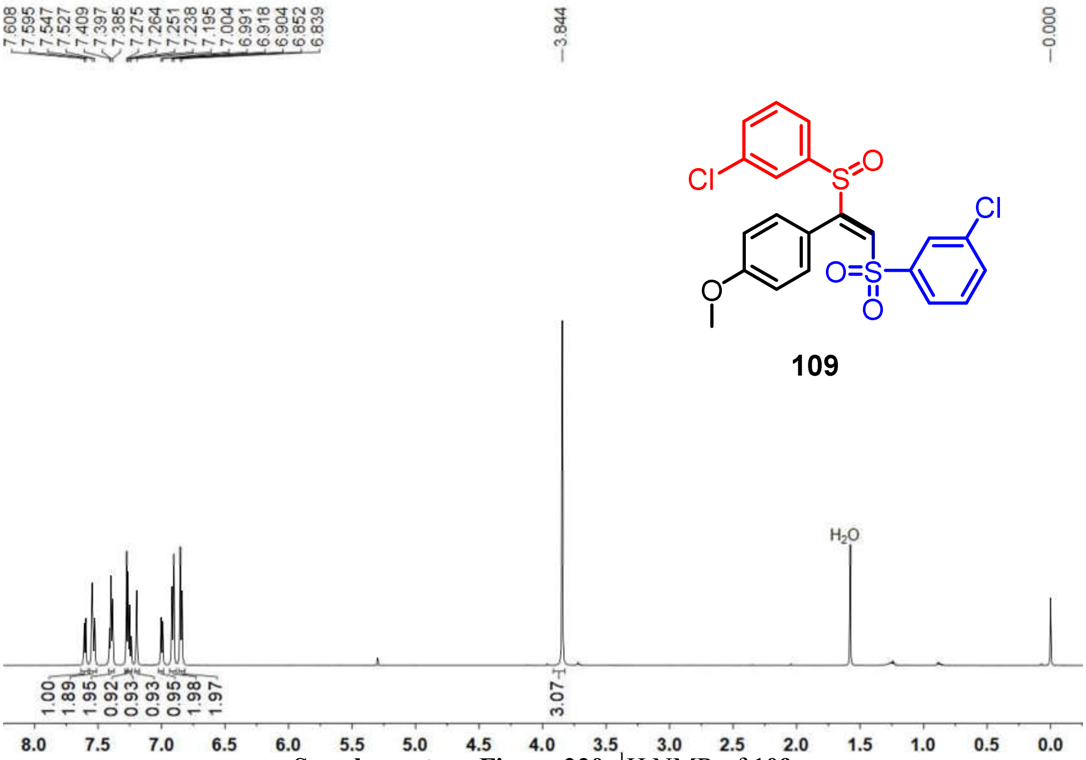

Supplementary Figure 230. ^{1}H NMR of **109**

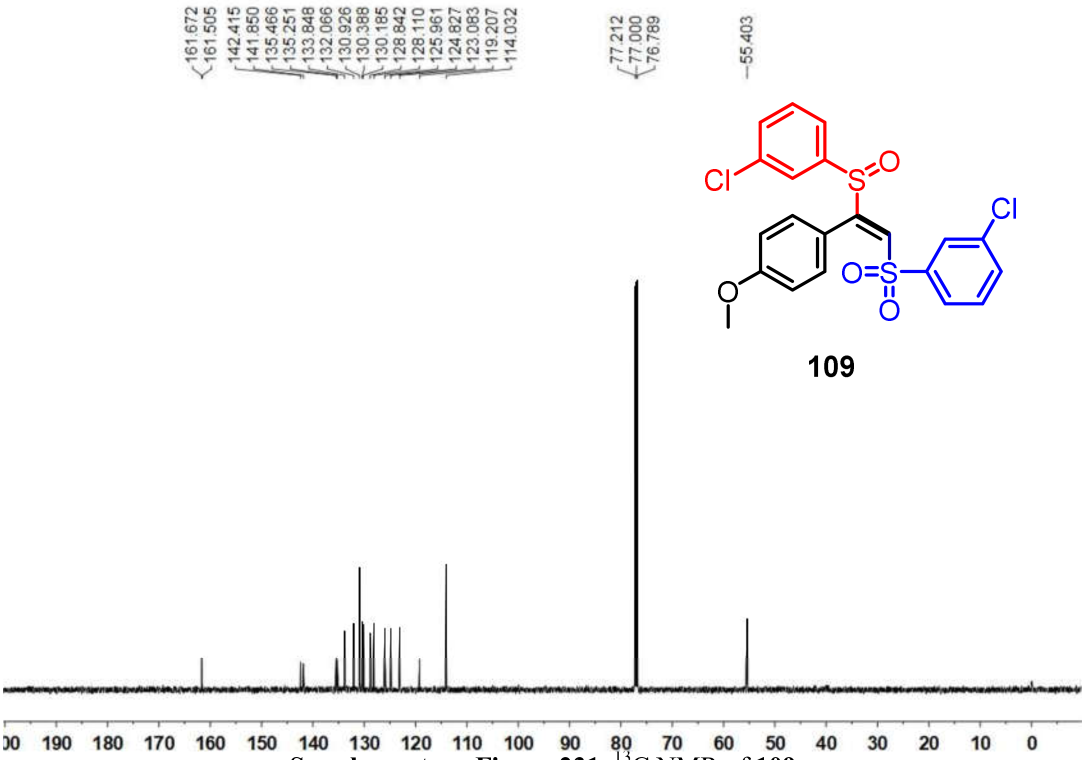

Supplementary Figure 231. ^{13}C NMR of **109**

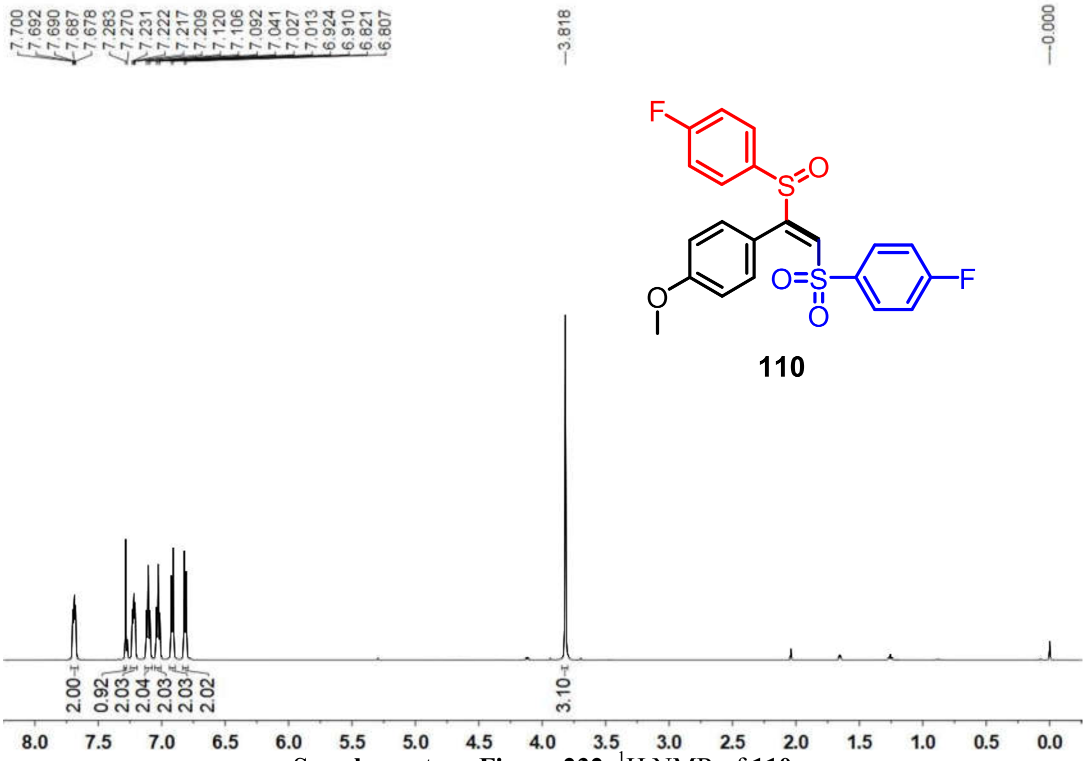

Supplementary Figure 232. ^{1}H NMR of **110**

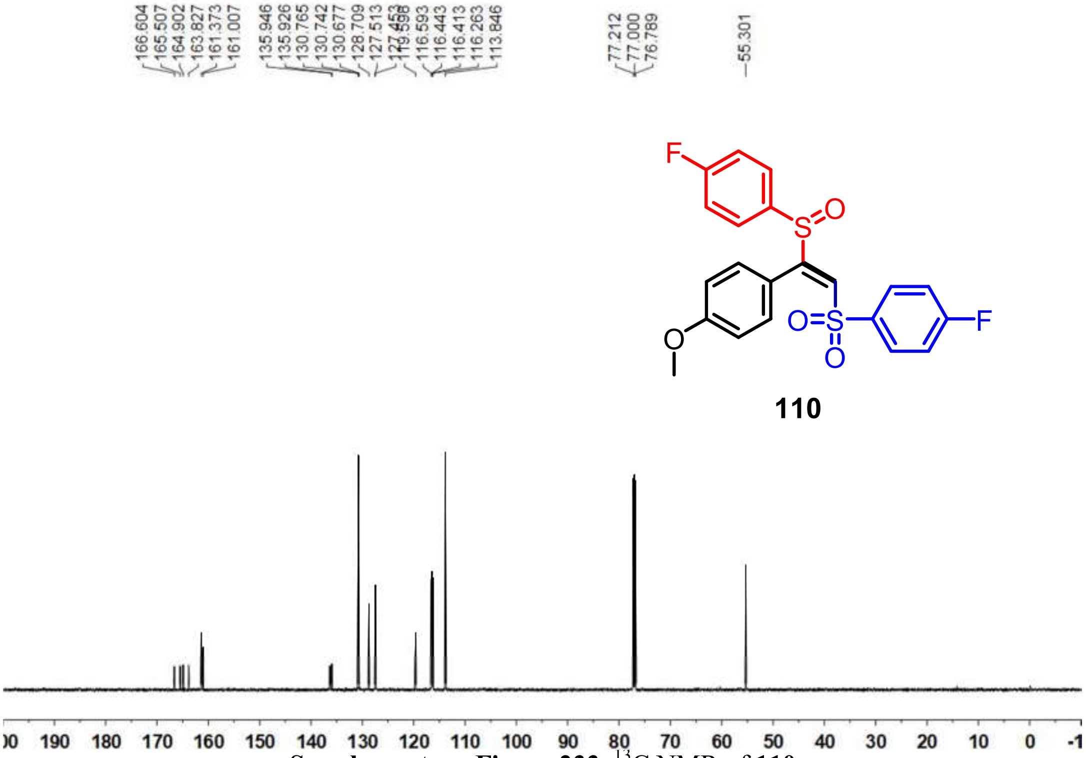

Supplementary Figure 233. ^{13}C NMR of **110**

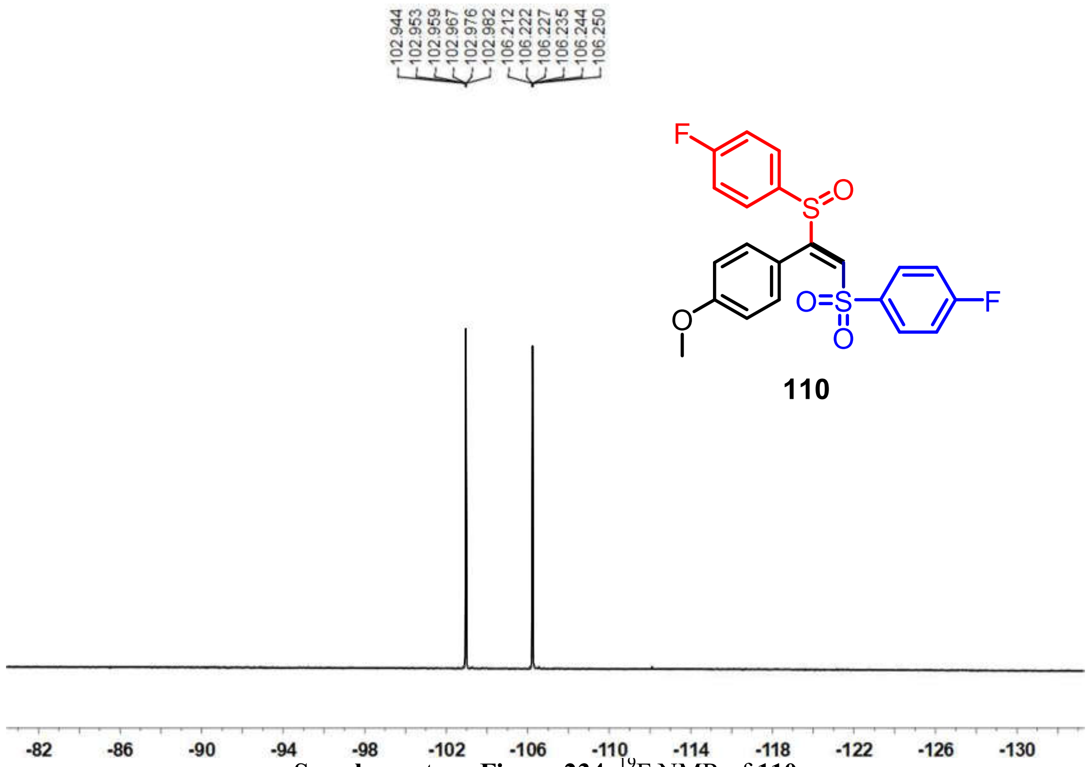

Supplementary Figure 234. ^{19}F NMR of **110**

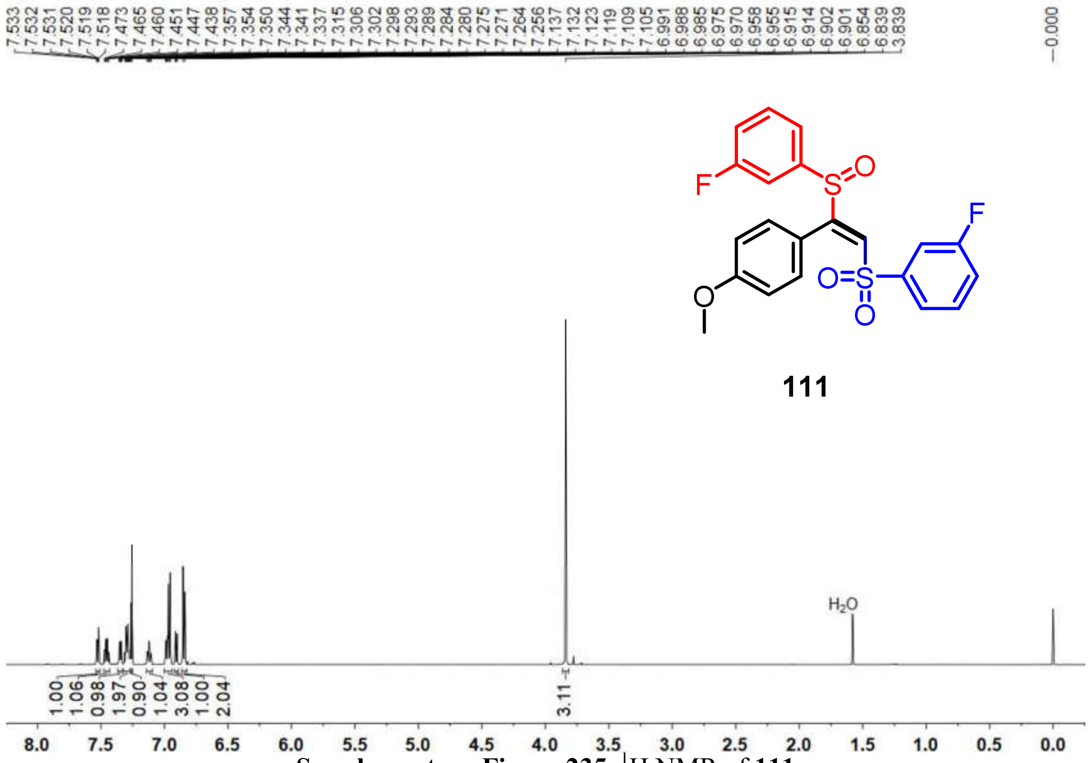

Supplementary Figure 235. ^{1}H NMR of **111**

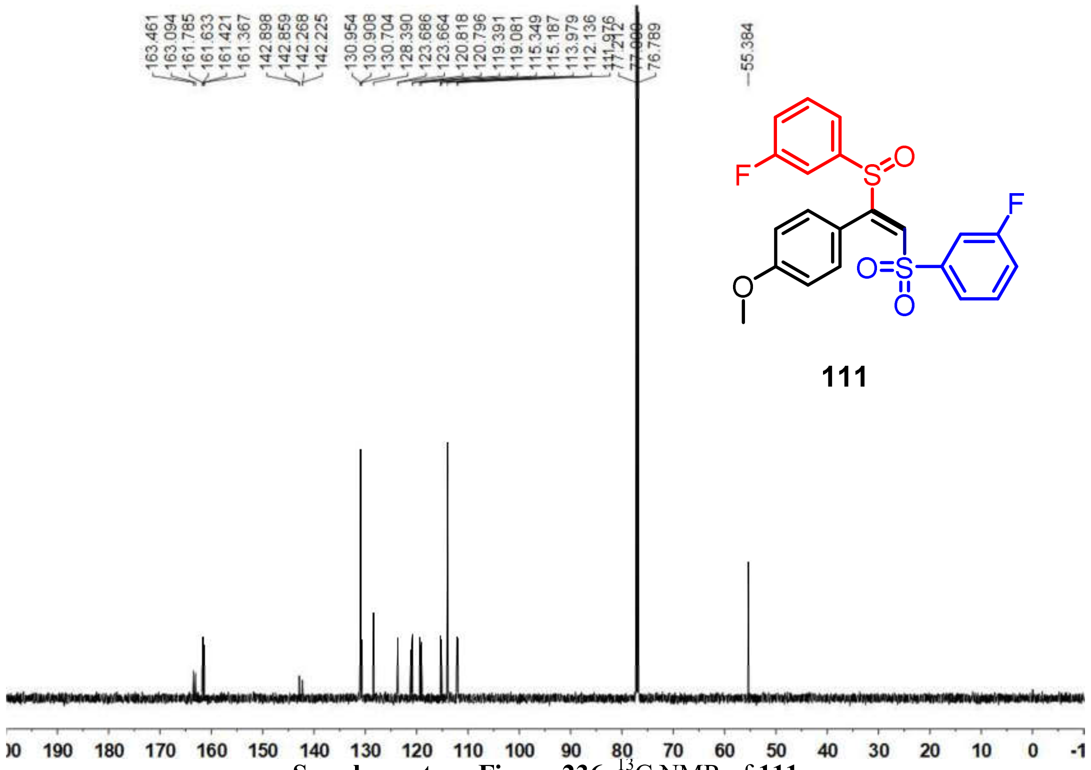

Supplementary Figure 236. ^{13}C NMR of **111**

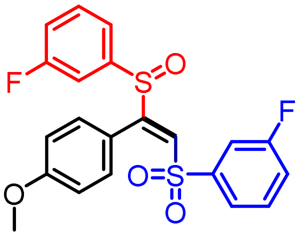

111

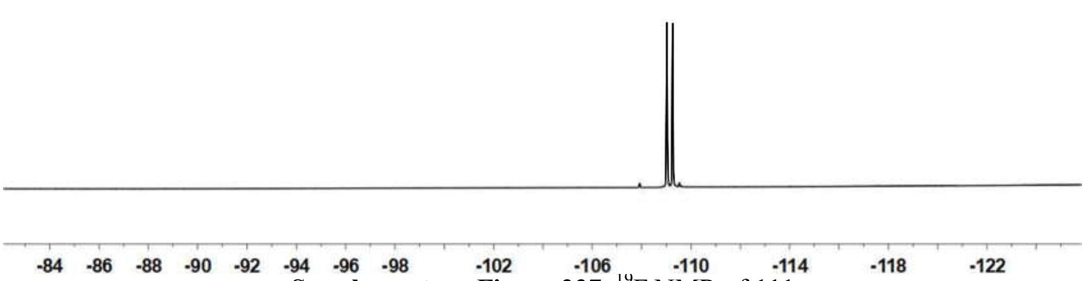

Supplementary Figure 237. ^{19}F NMR of **111**

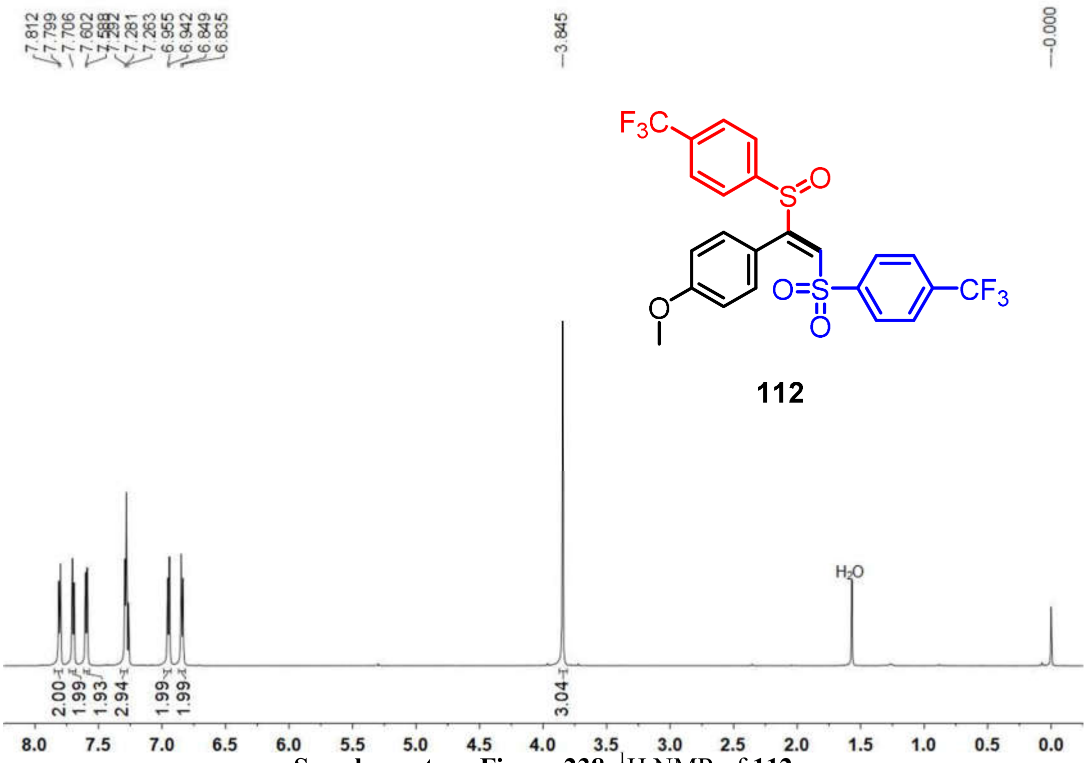

Supplementary Figure 238. ^{1}H NMR of **112**

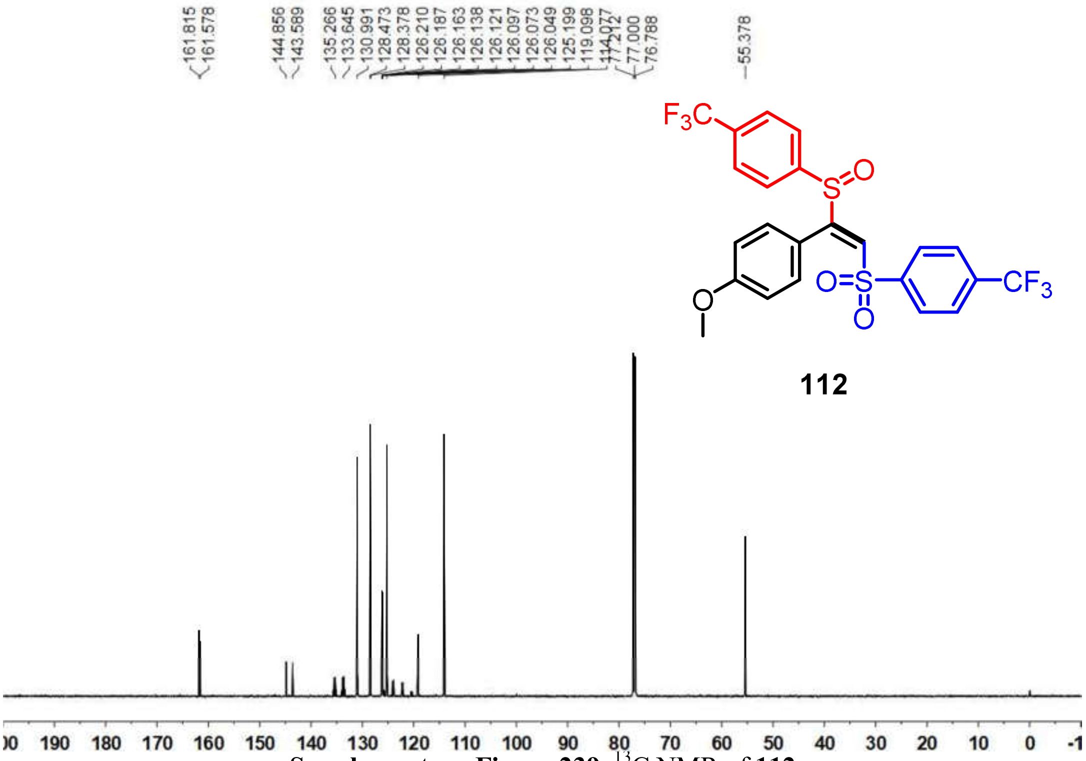

Supplementary Figure 239. ^{13}C NMR of **112**

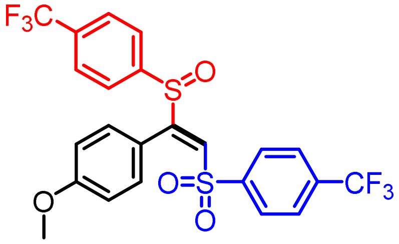

112

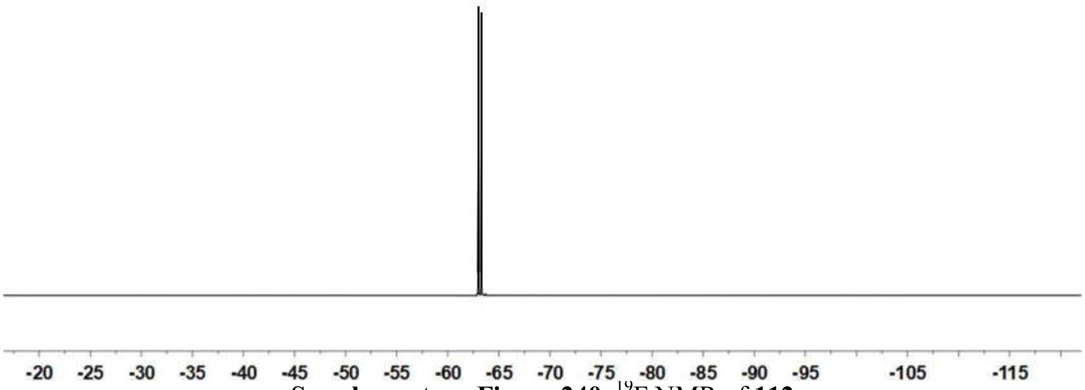

Supplementary Figure 240. ^{19}F NMR of **112**

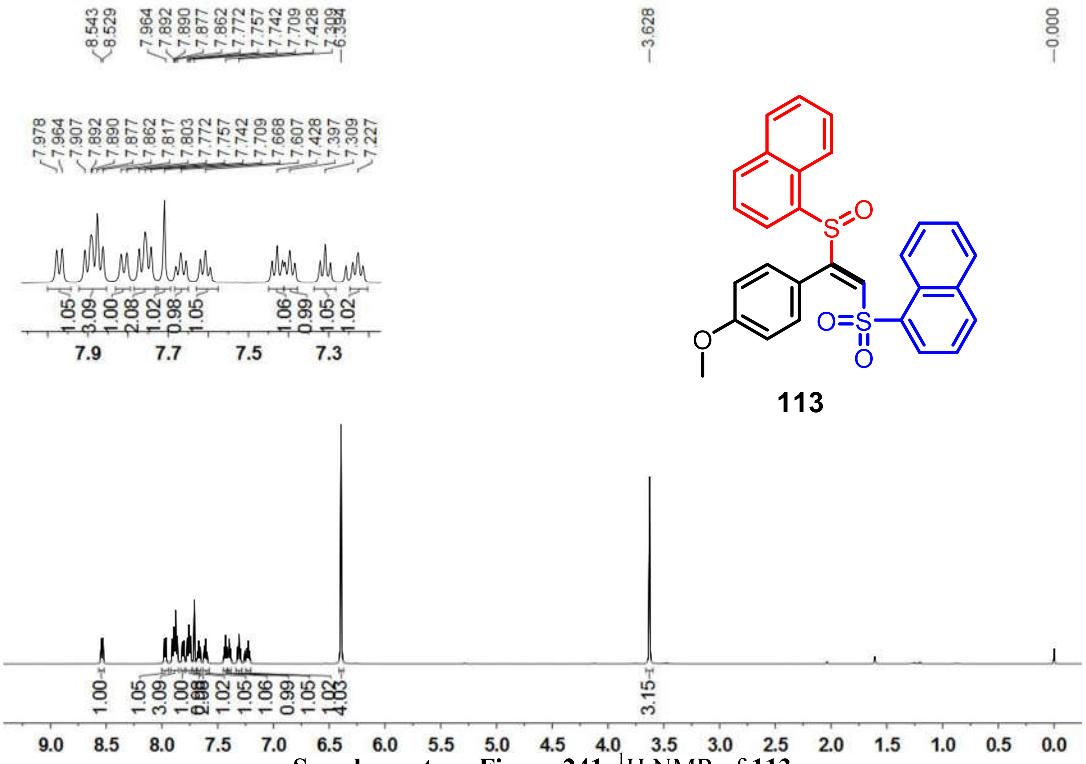

Supplementary Figure 241. ^{1}H NMR of **113**

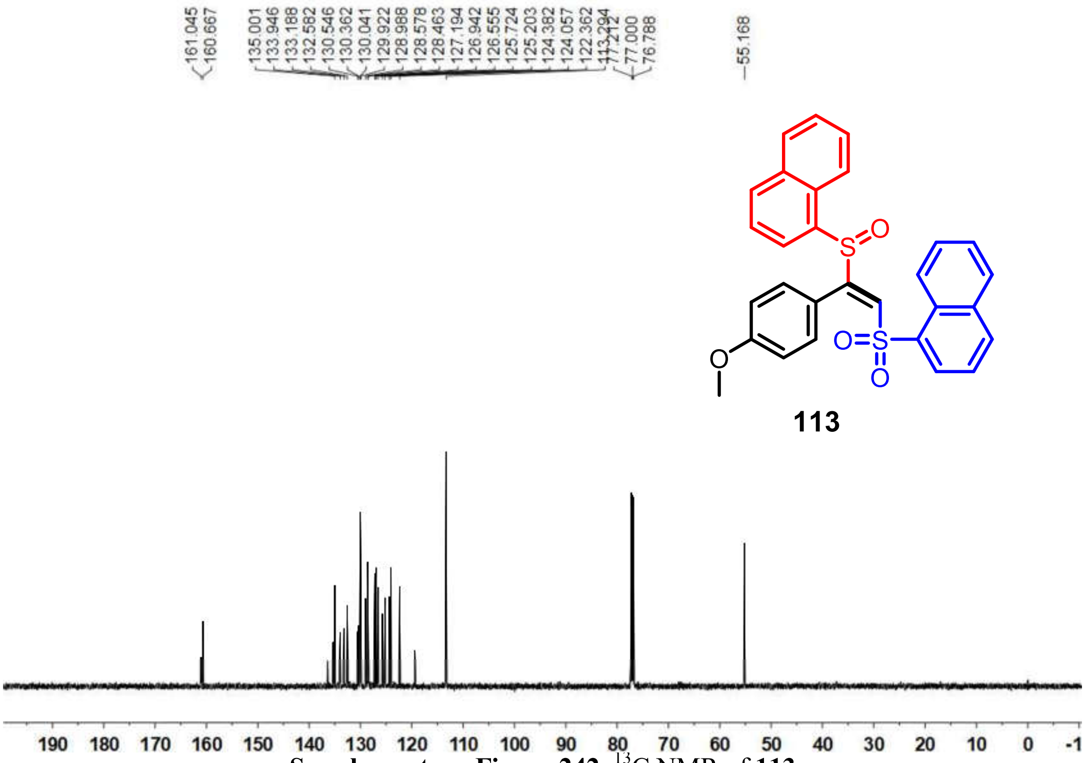

Supplementary Figure 242. ^{13}C NMR of **113**

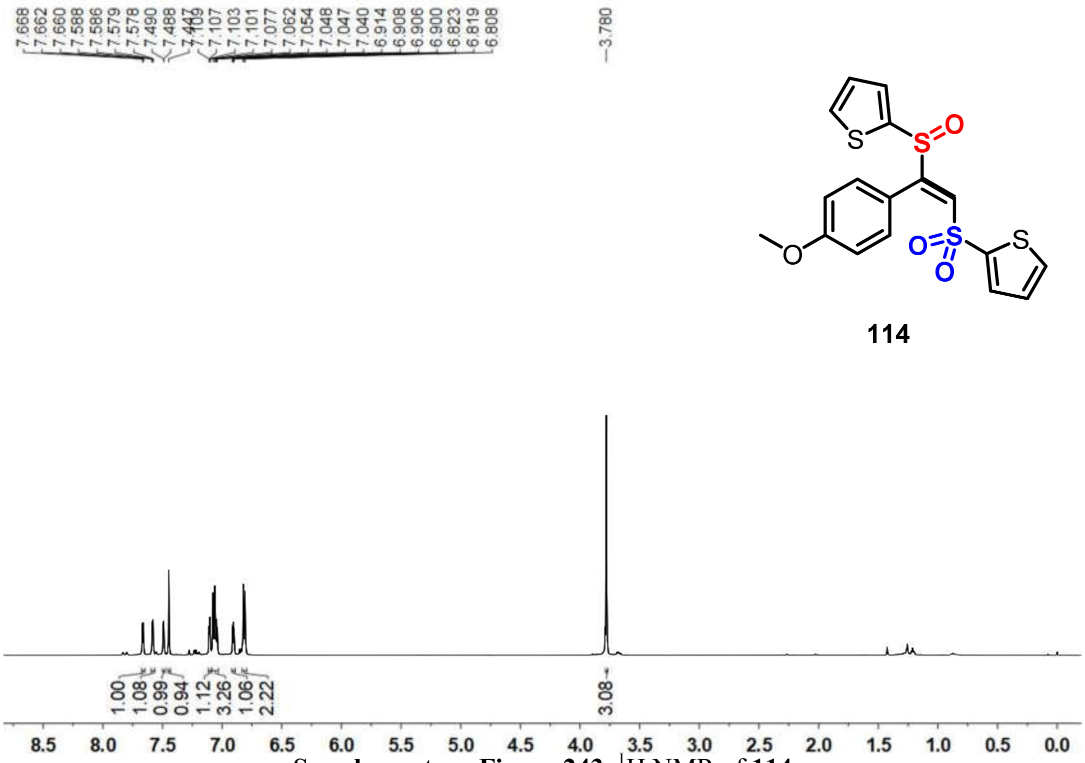

Supplementary Figure 243. ^{1}H NMR of **114**

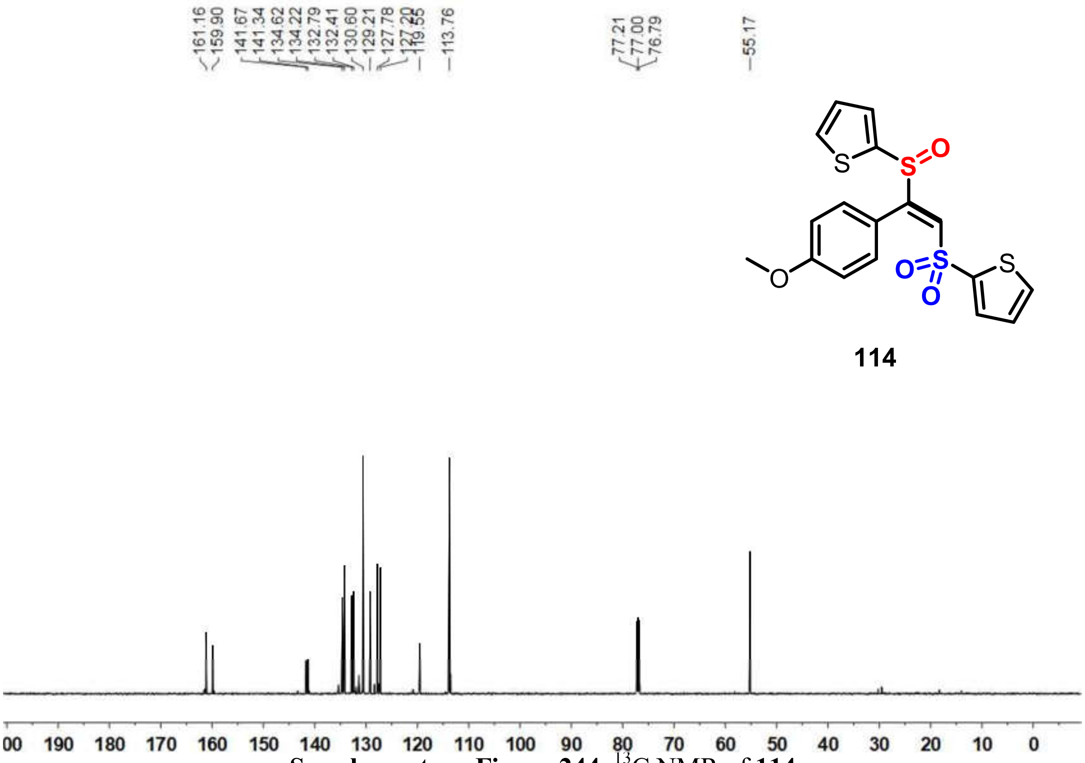

Supplementary Figure 244. ^{13}C NMR of **114**

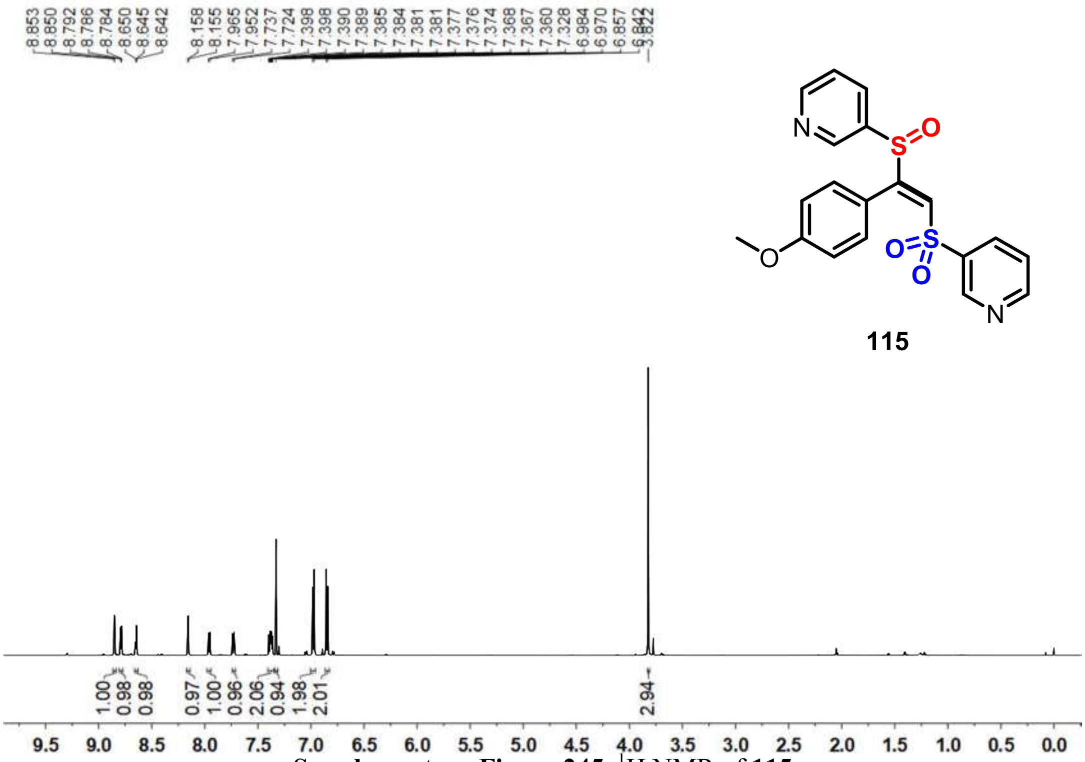

Supplementary Figure 245. ^{1}H NMR of **115**

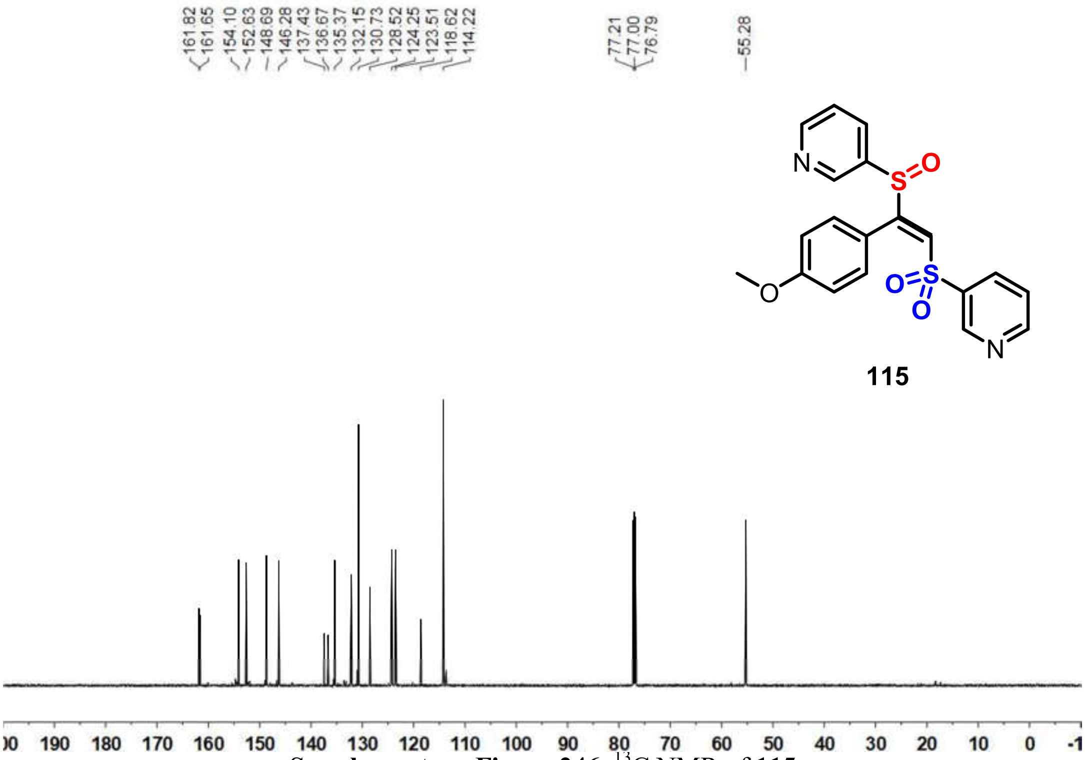

Supplementary Figure 246. ^{13}C NMR of **115**

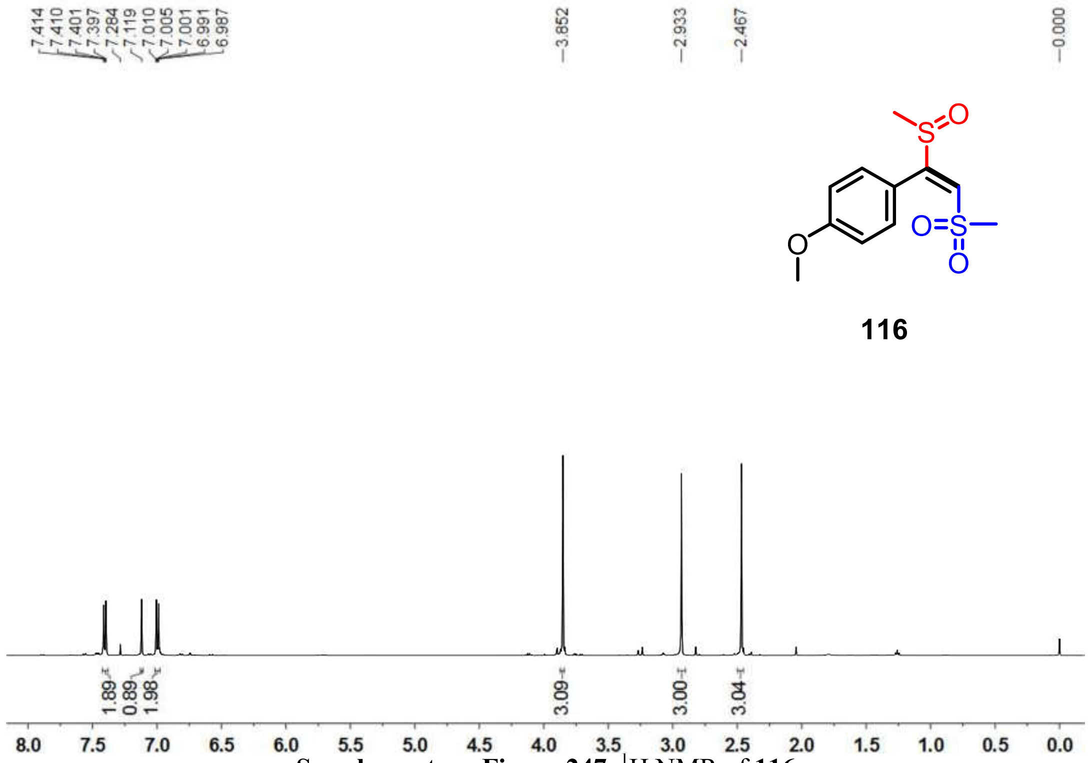

Supplementary Figure 247. ^{1}H NMR of **116**

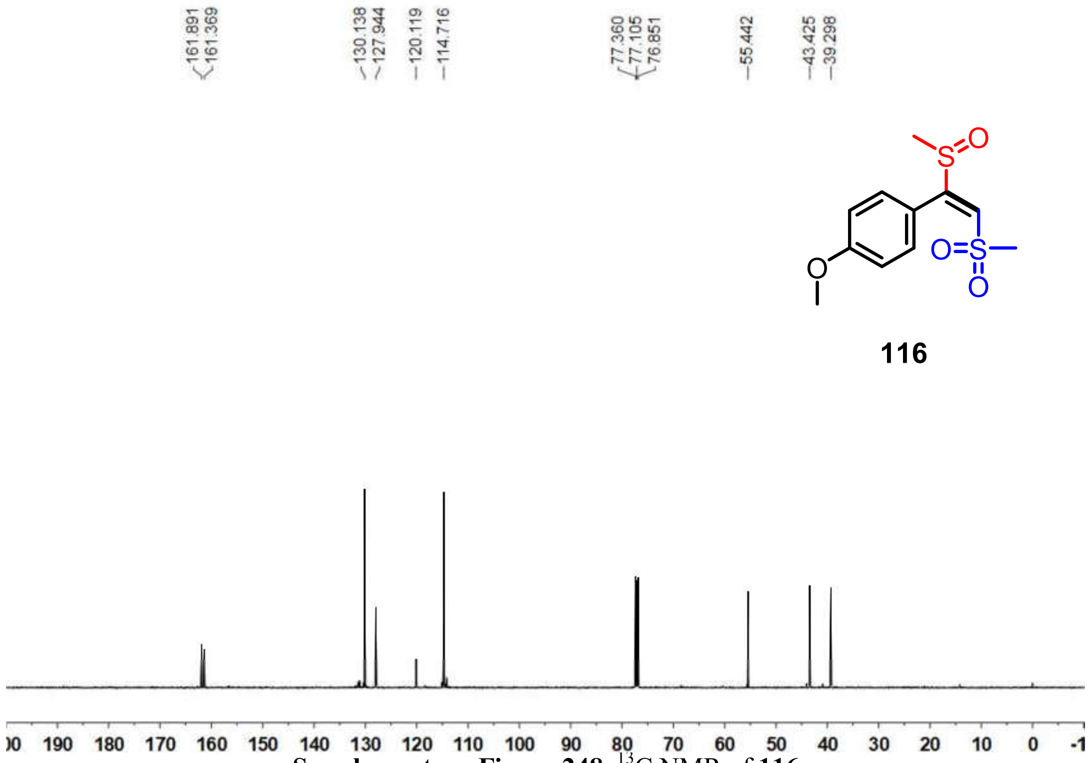

Supplementary Figure 248. ^{13}C NMR of **116**

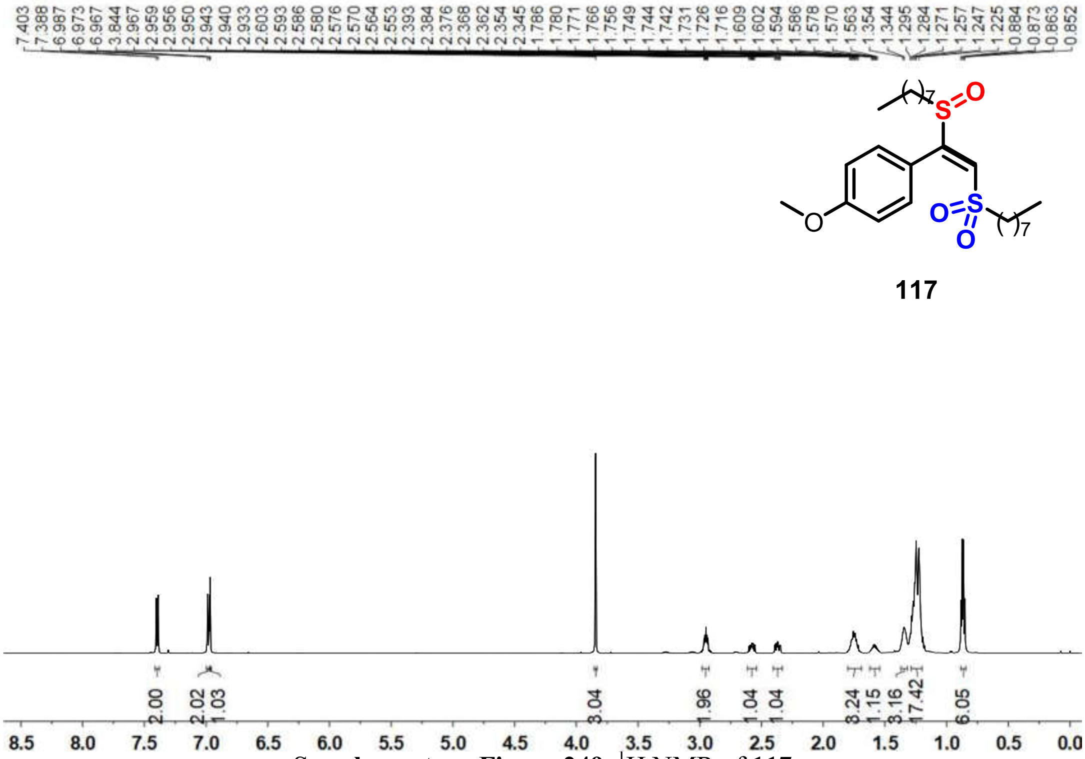

Supplementary Figure 249. ^{1}H NMR of **117**

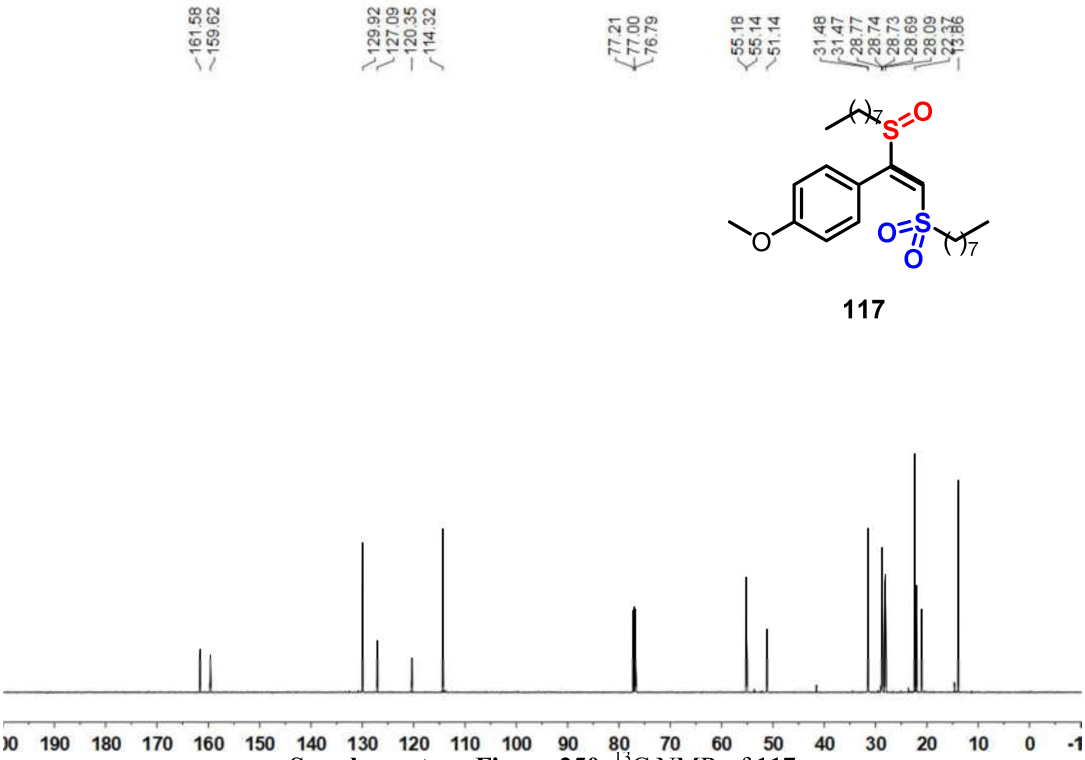

Supplementary Figure 250. ^{13}C NMR of **117**

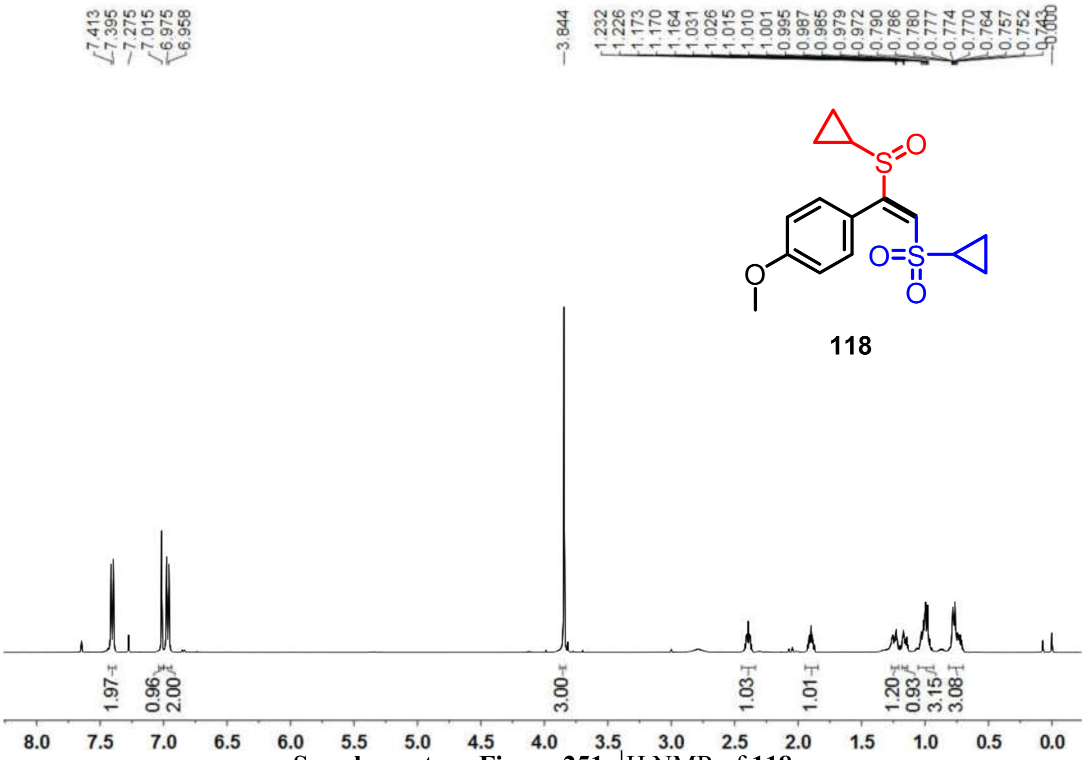

Supplementary Figure 251. ^{1}H NMR of **118**

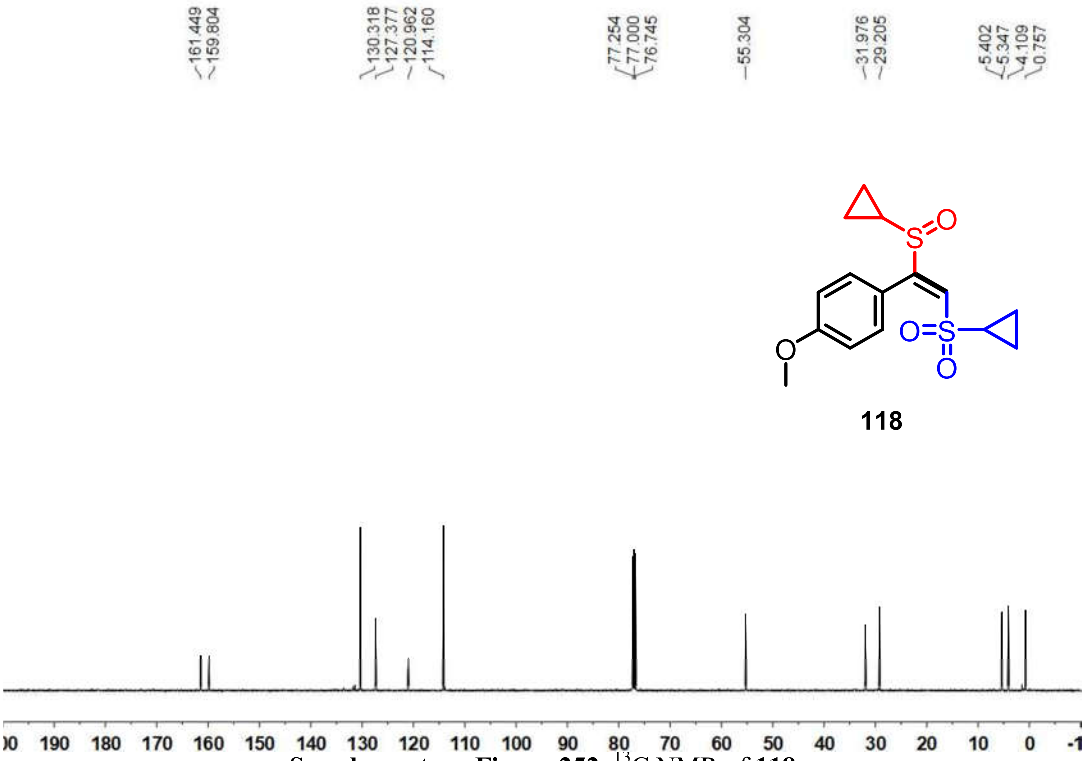

Supplementary Figure 252. ^{13}C NMR of **118**

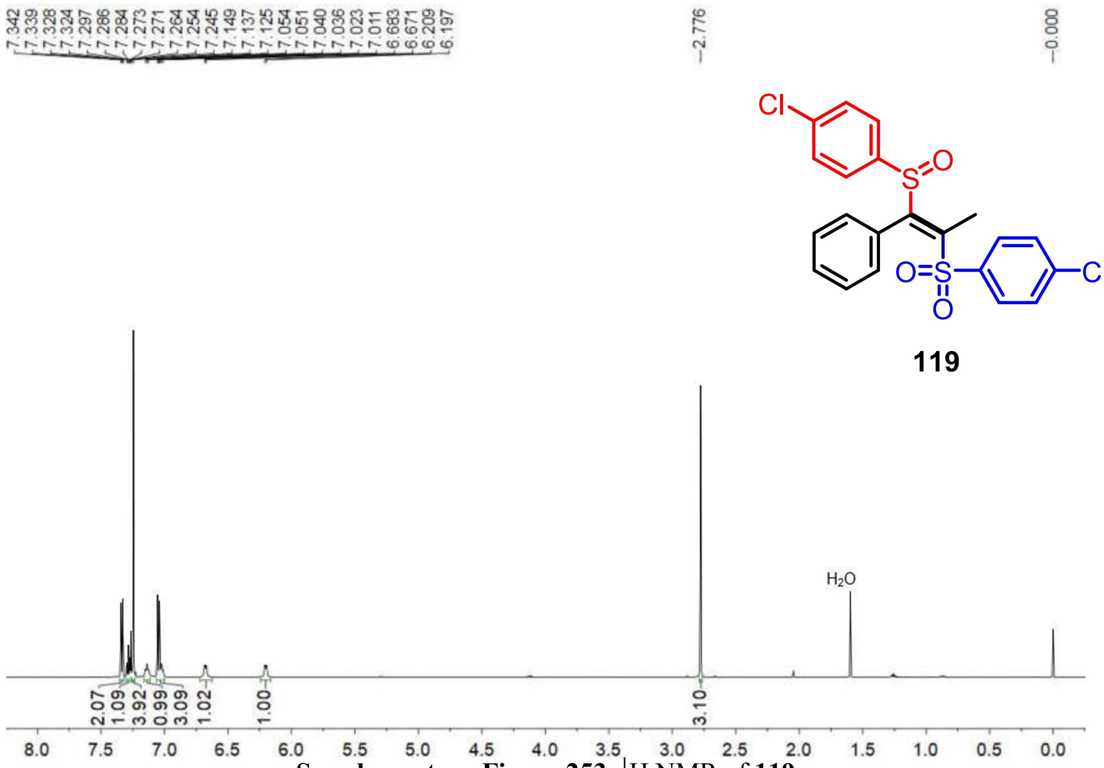

Supplementary Figure 253. ^{1}H NMR of **119**

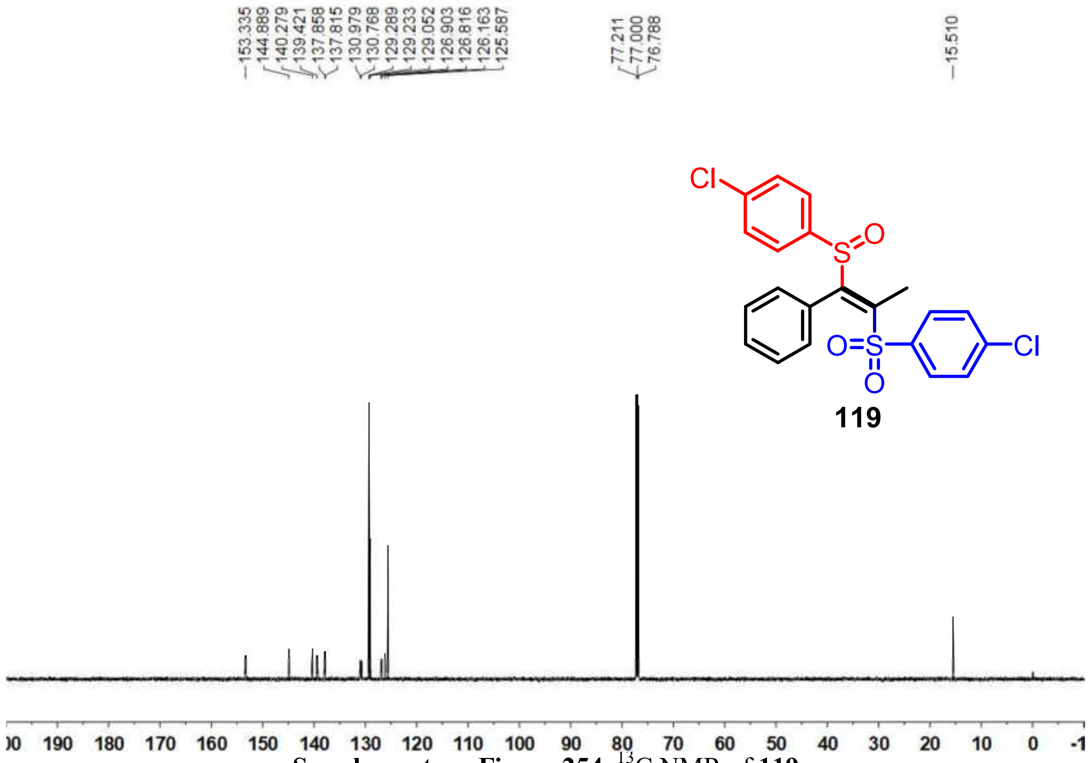

Supplementary Figure 254. ^{13}C NMR of **119**

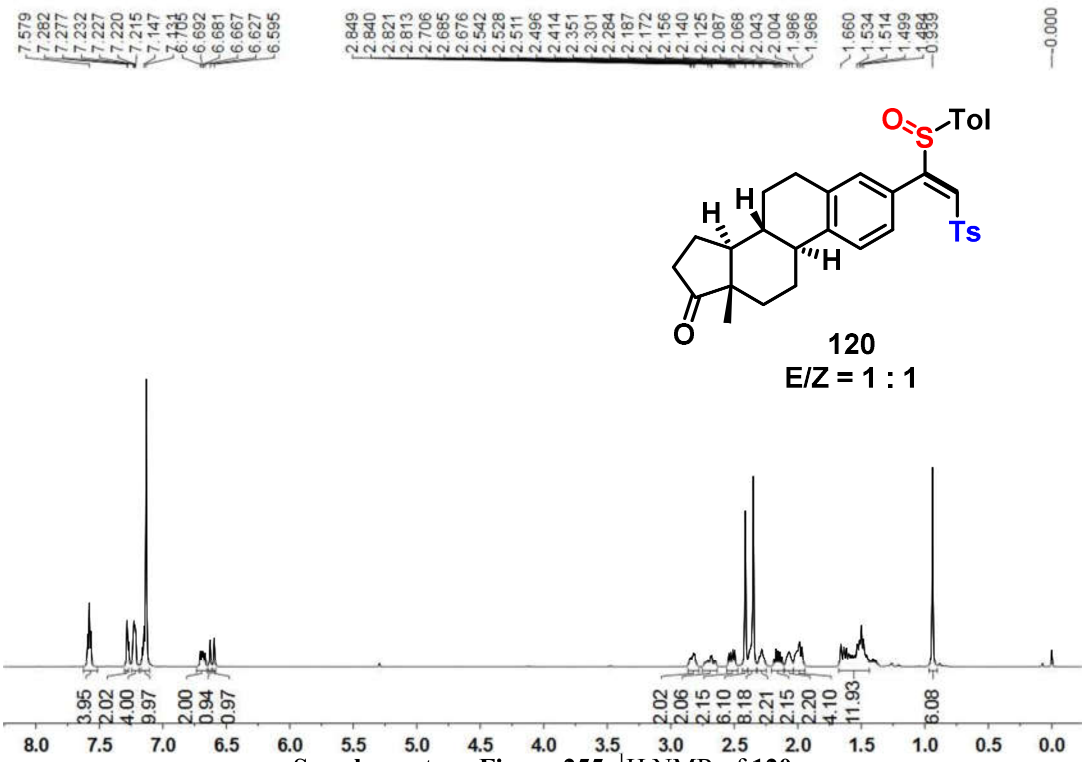

Supplementary Figure 255. ^{1}H NMR of **120**

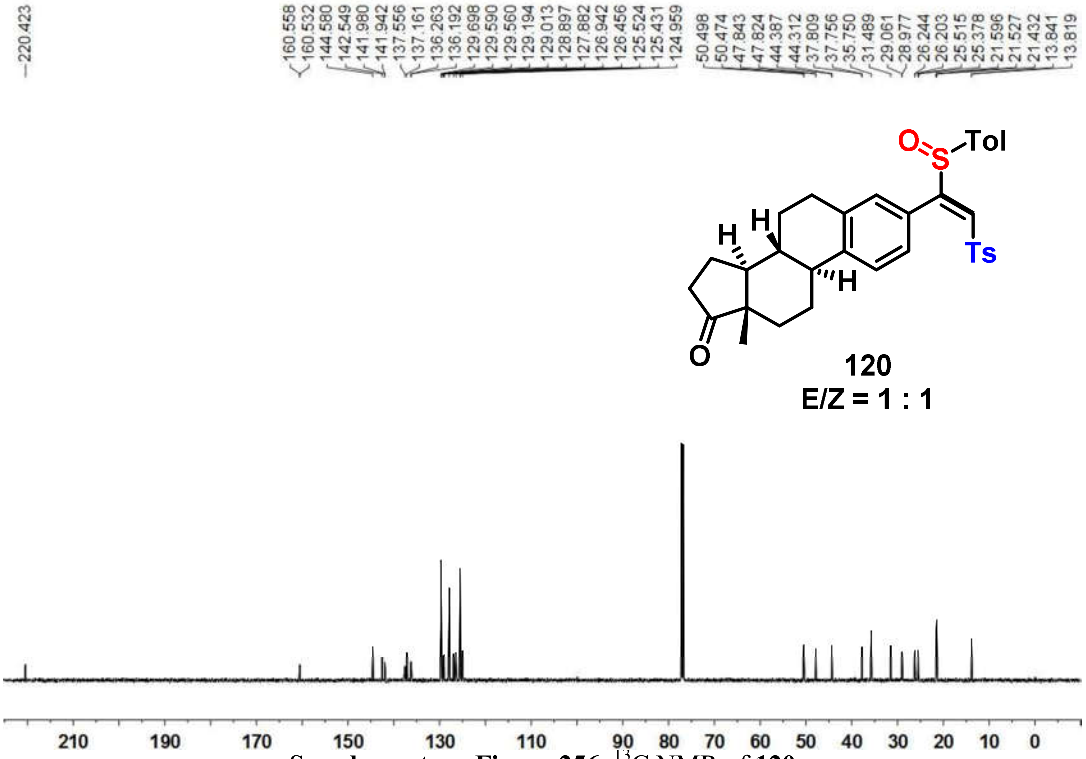

Supplementary Figure 256. ^{13}C NMR of **120**

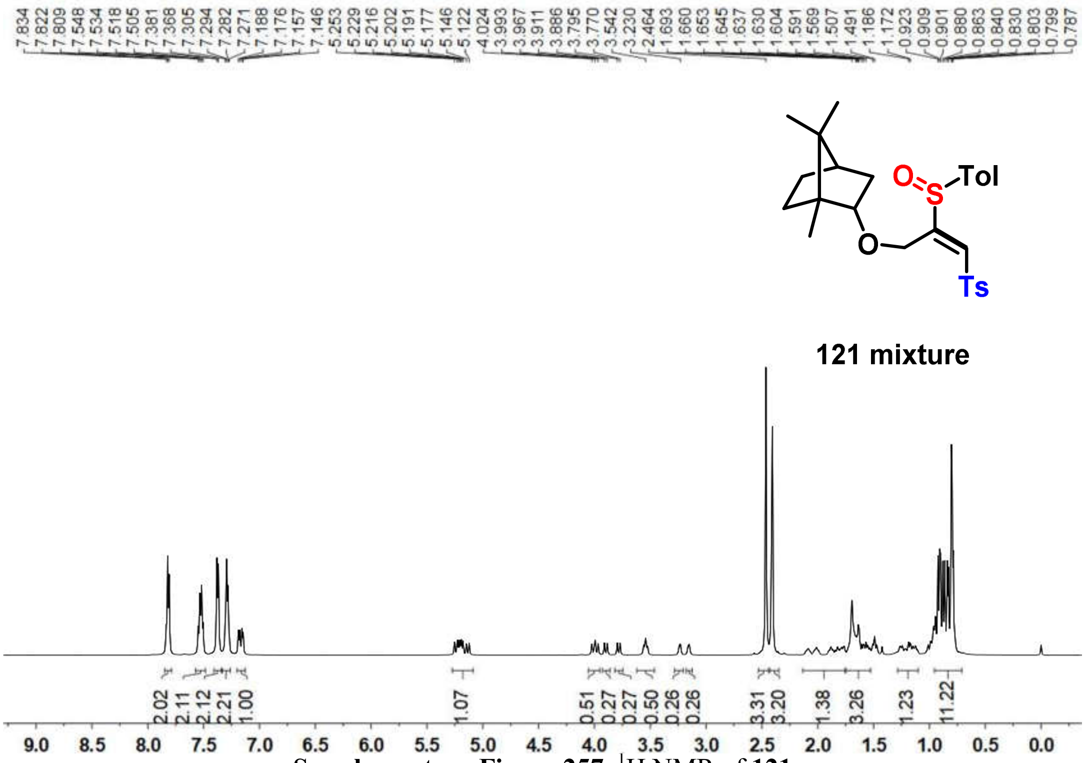

Supplementary Figure 257. ^{1}H NMR of **121**

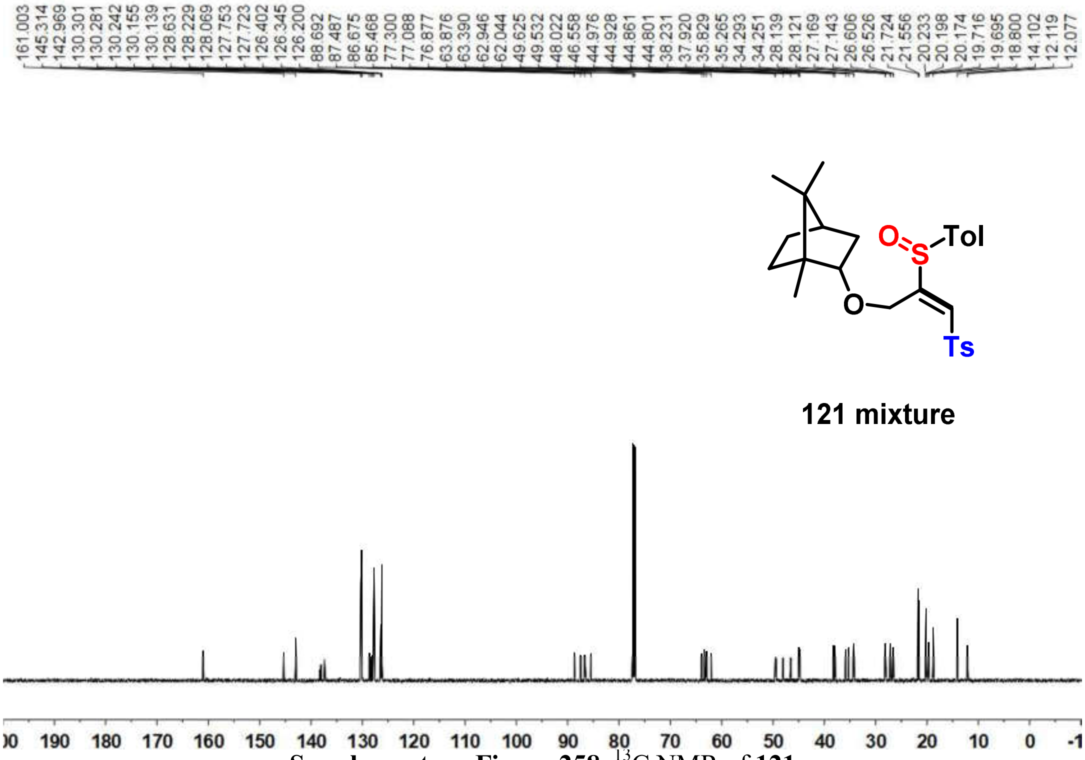

Supplementary Figure 258. ^{13}C NMR of **121**

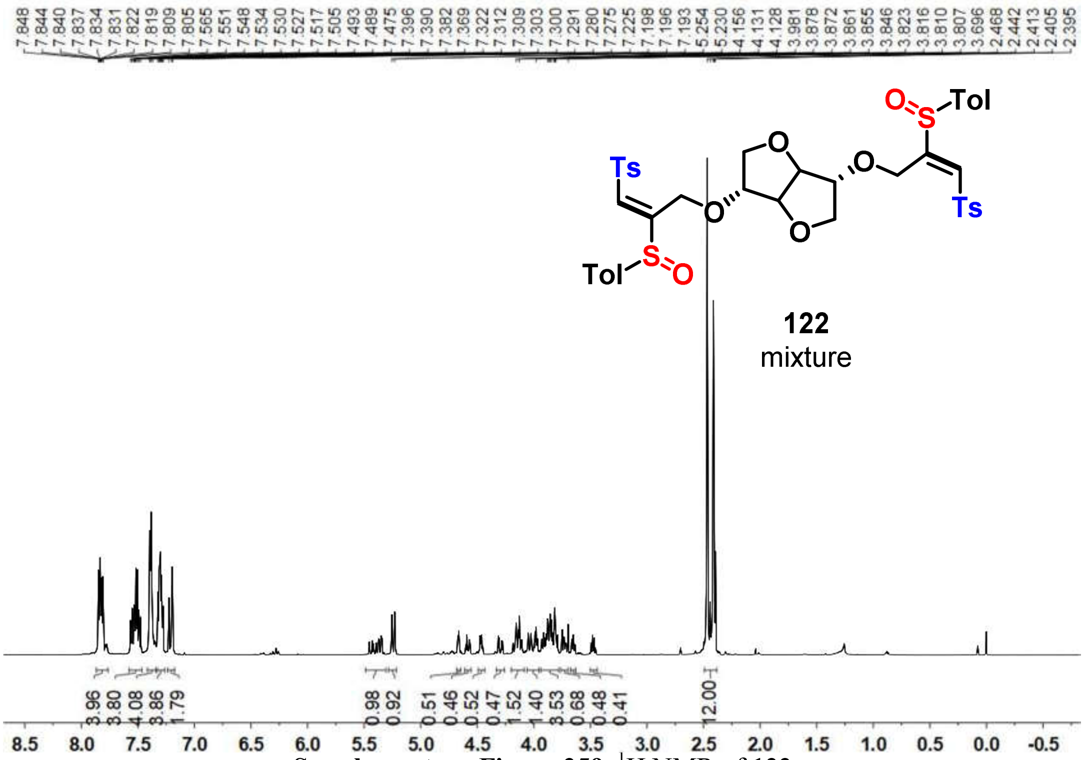

Supplementary Figure 259. ^{1}H NMR of **122**

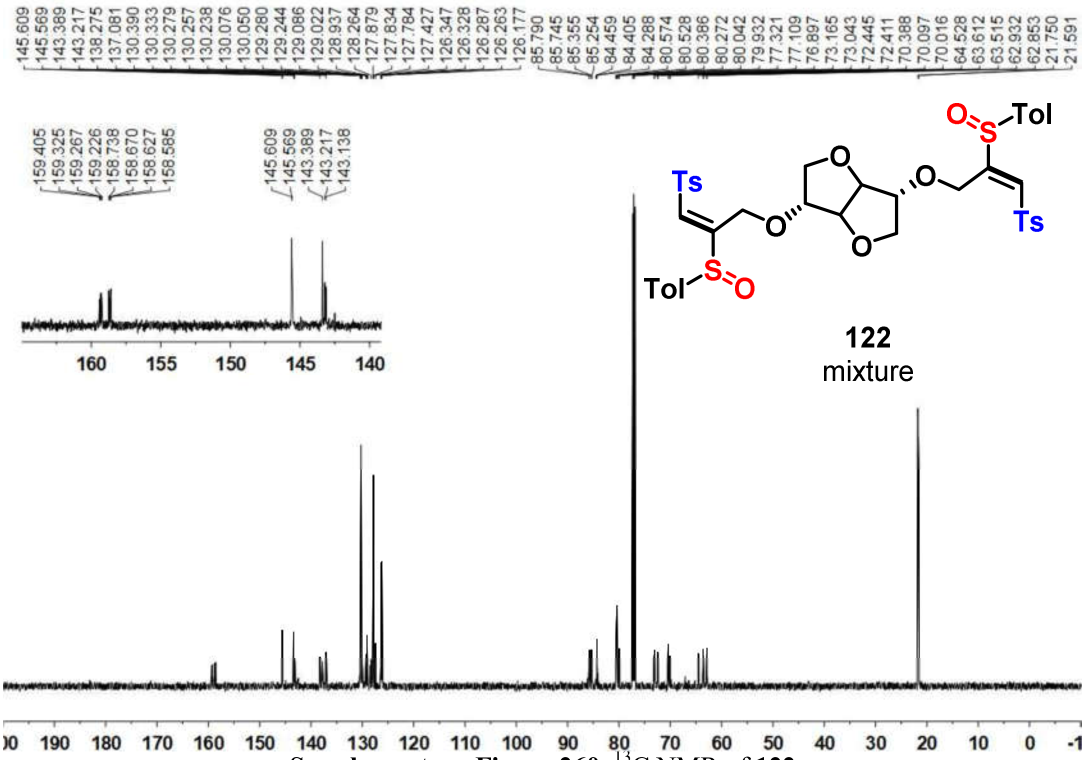

Supplementary Figure 260. ^{13}C NMR of **122**

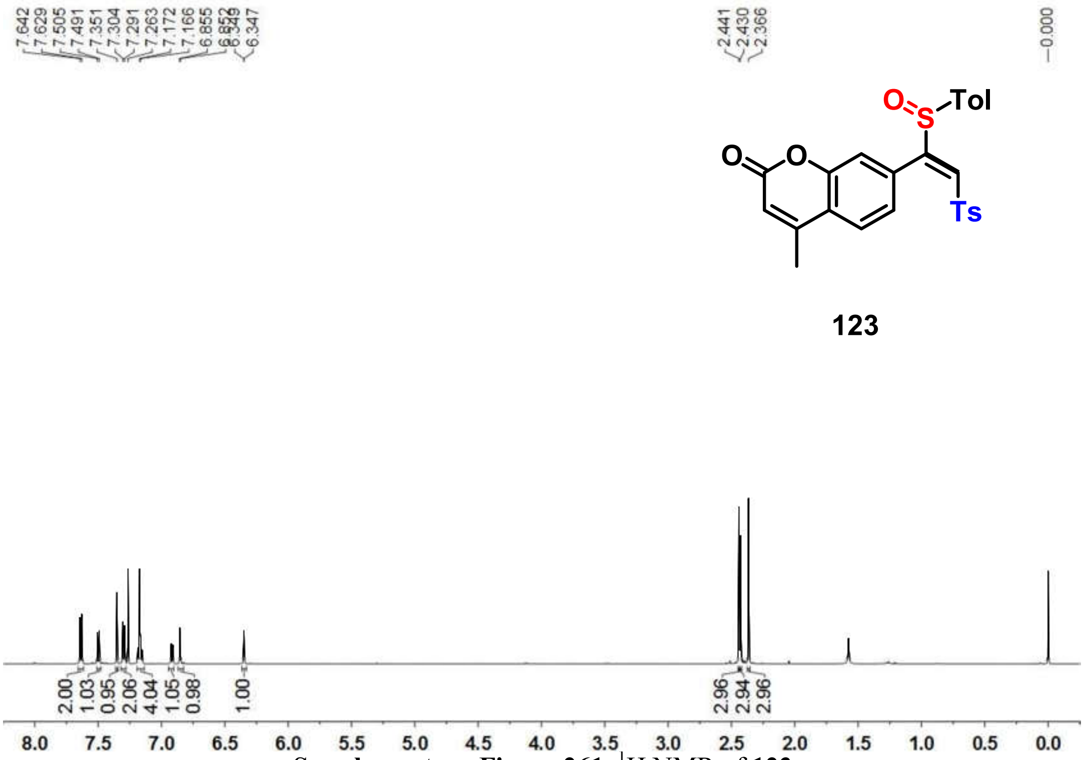

Supplementary Figure 261. ^{1}H NMR of **123**

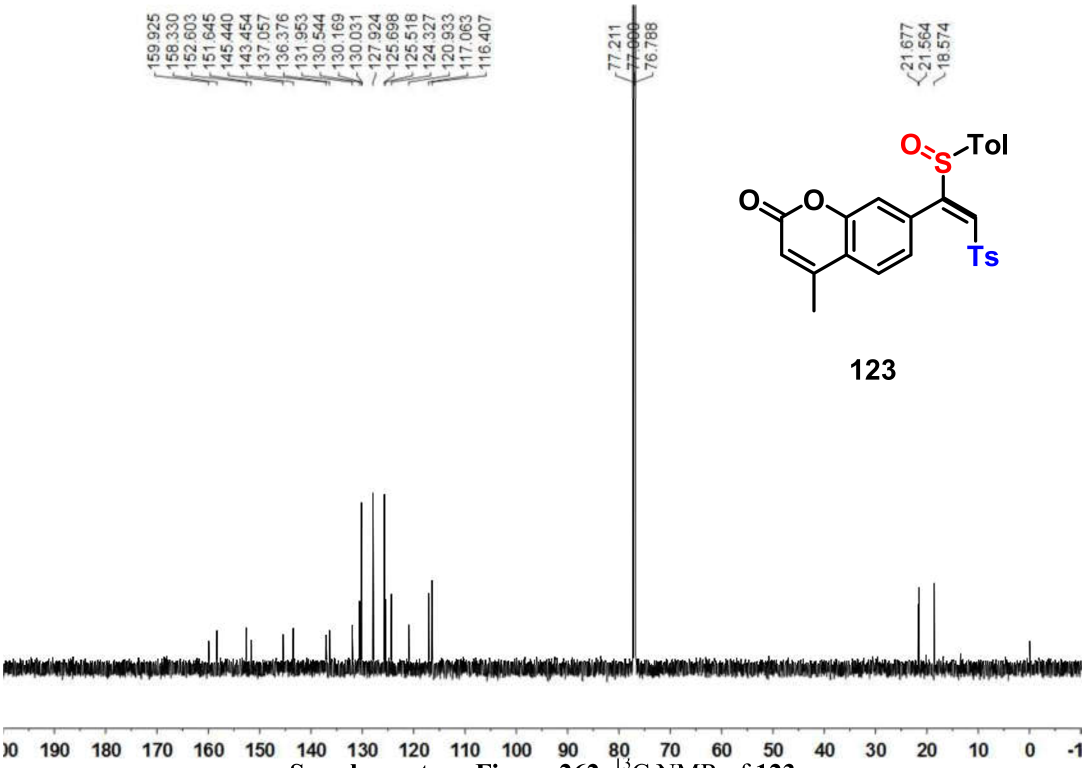

Supplementary Figure 262. ^{13}C NMR of **123**

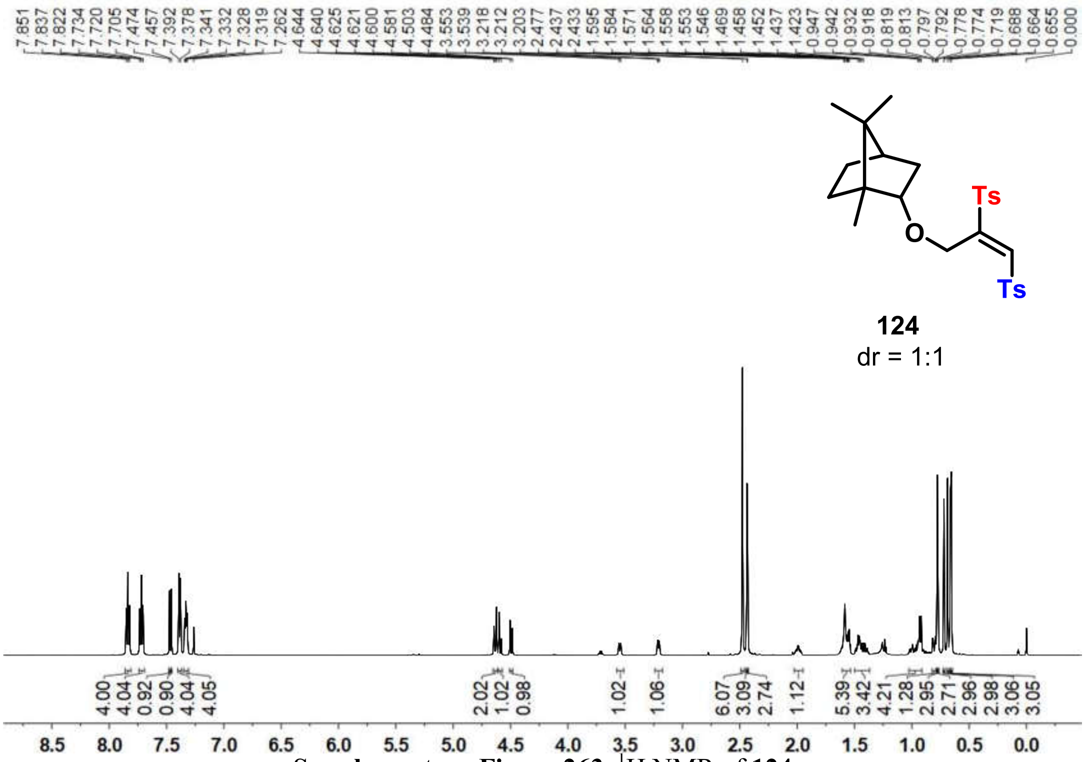

Supplementary Figure 263. ^{1}H NMR of **124**

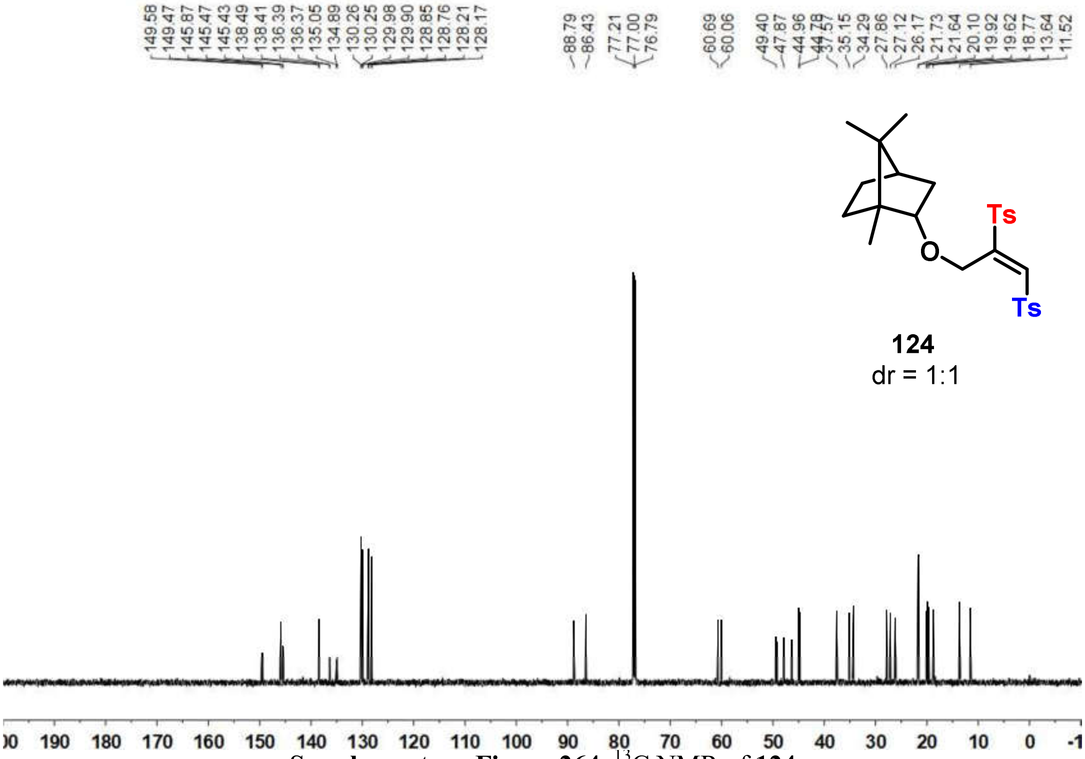

Supplementary Figure 264. ^{13}C NMR of **124**

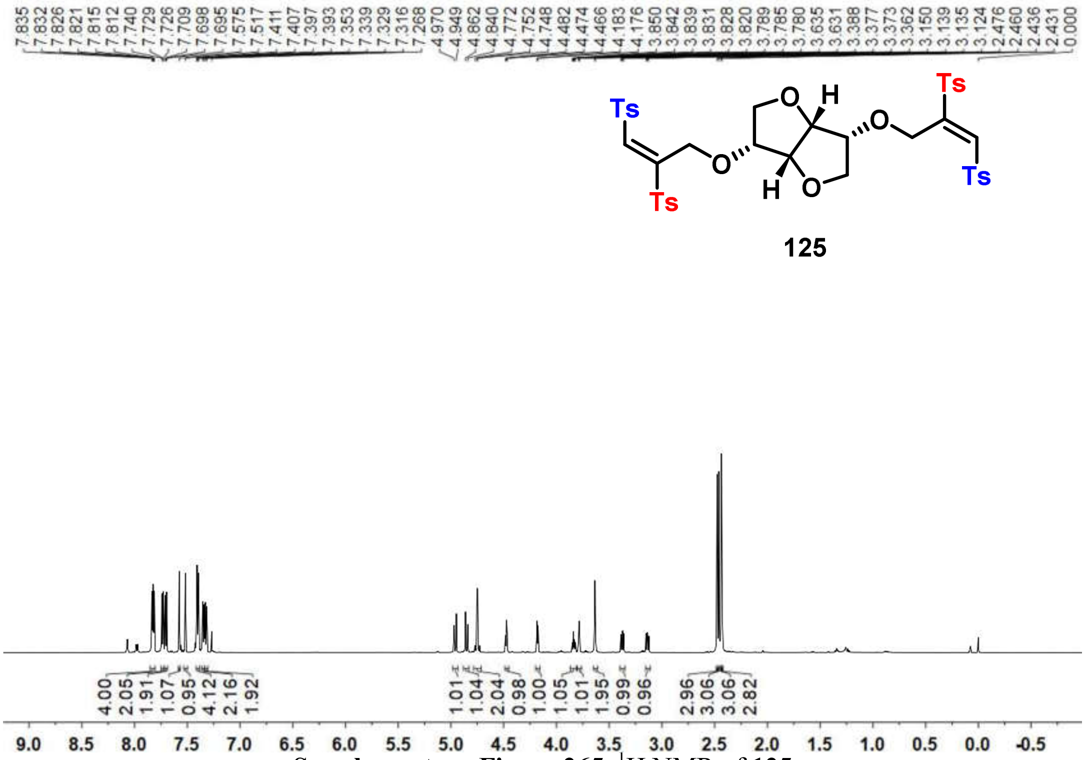

Supplementary Figure 265. ^{1}H NMR of **125**

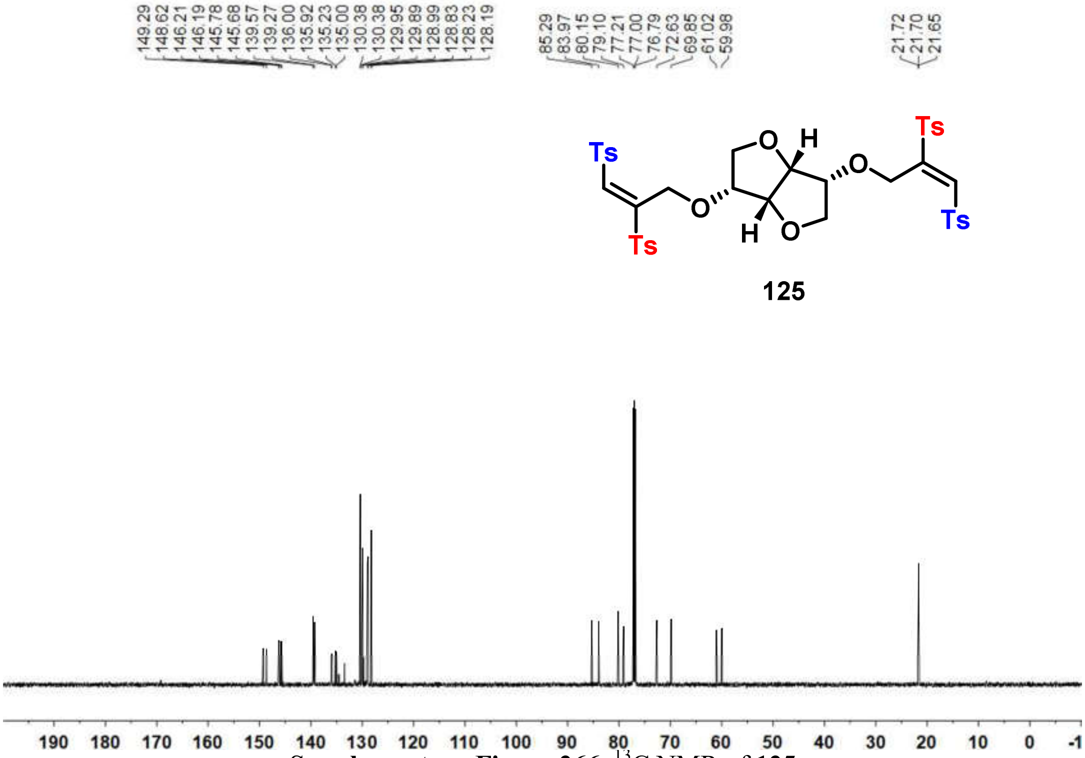

Supplementary Figure 266. ^{13}C NMR of **125**

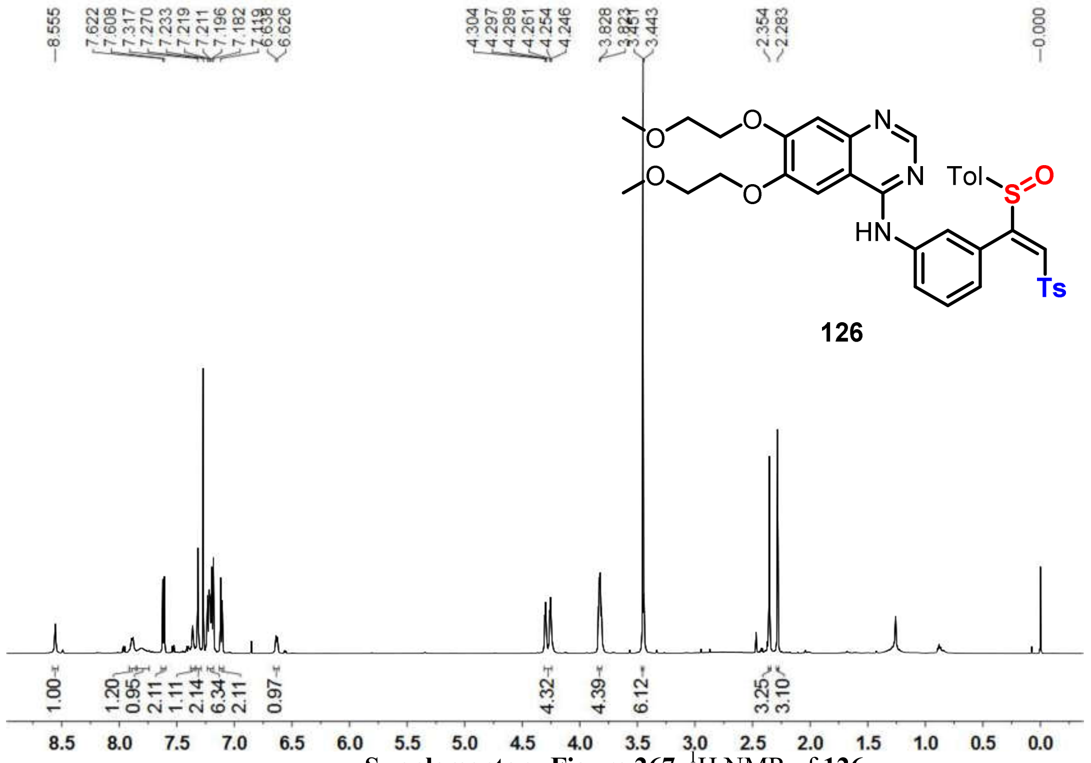

Supplementary Figure 267. ^{1}H NMR of **126**

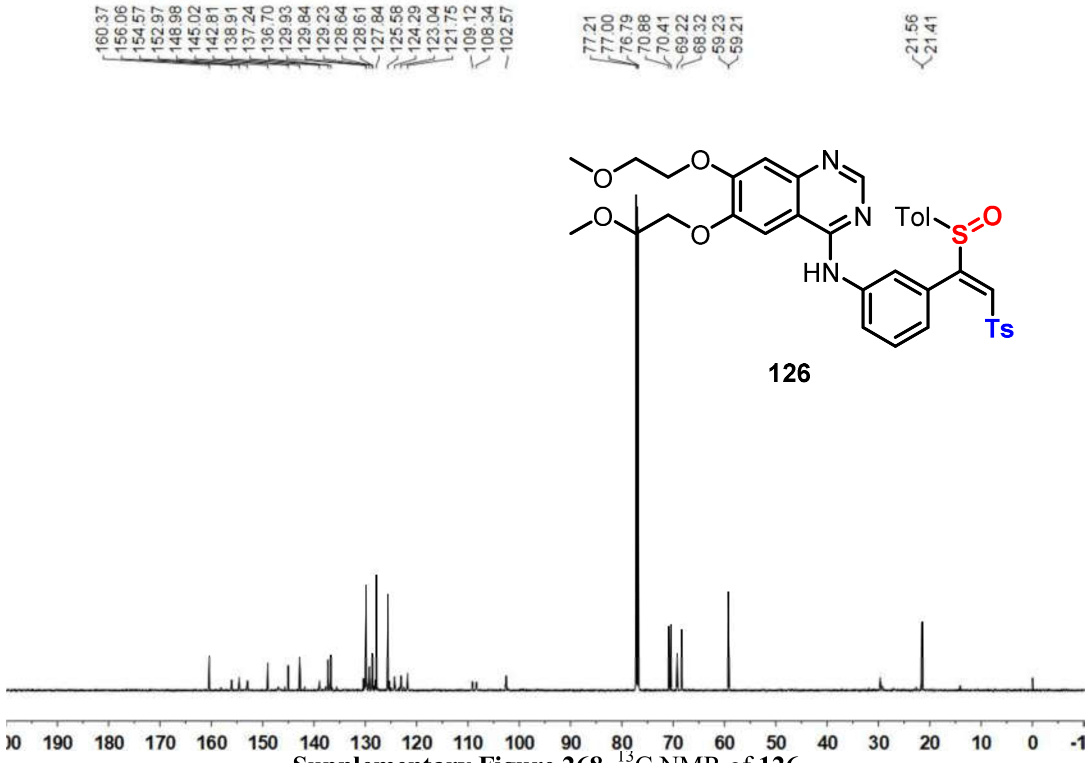

Supplementary Figure 268. ^{13}C NMR of **126**

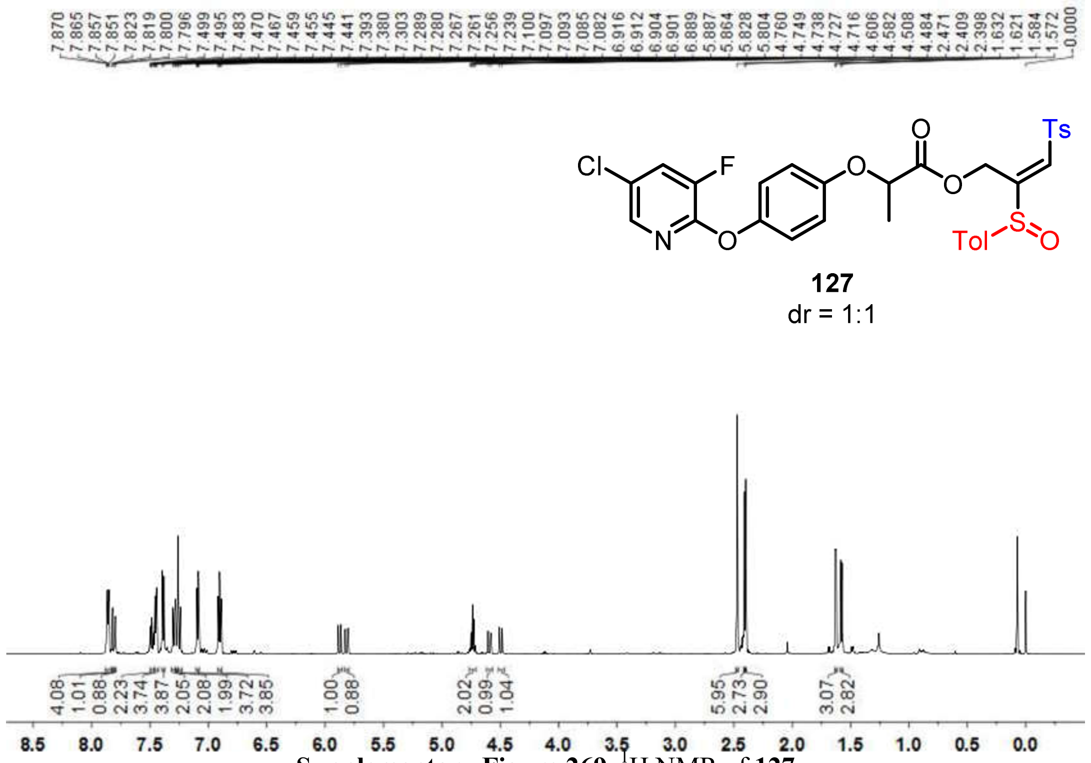

Supplementary Figure 269. ^{1}H NMR of **127**

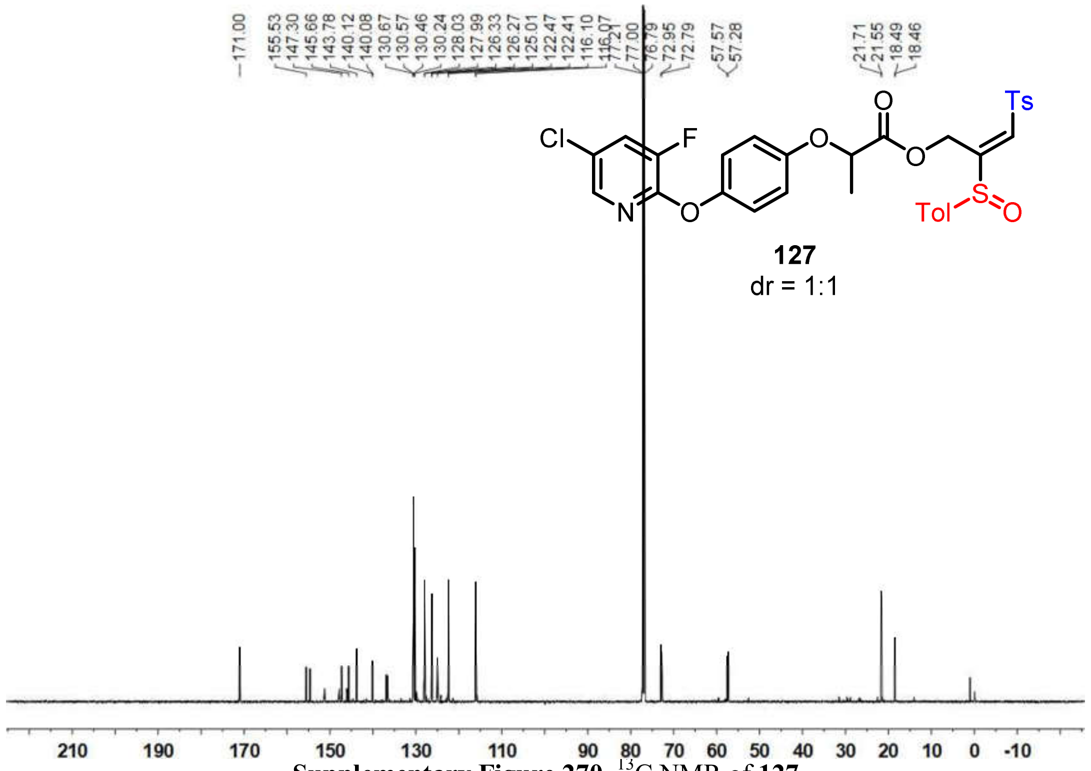

Supplementary Figure 270. ^{13}C NMR of **127**

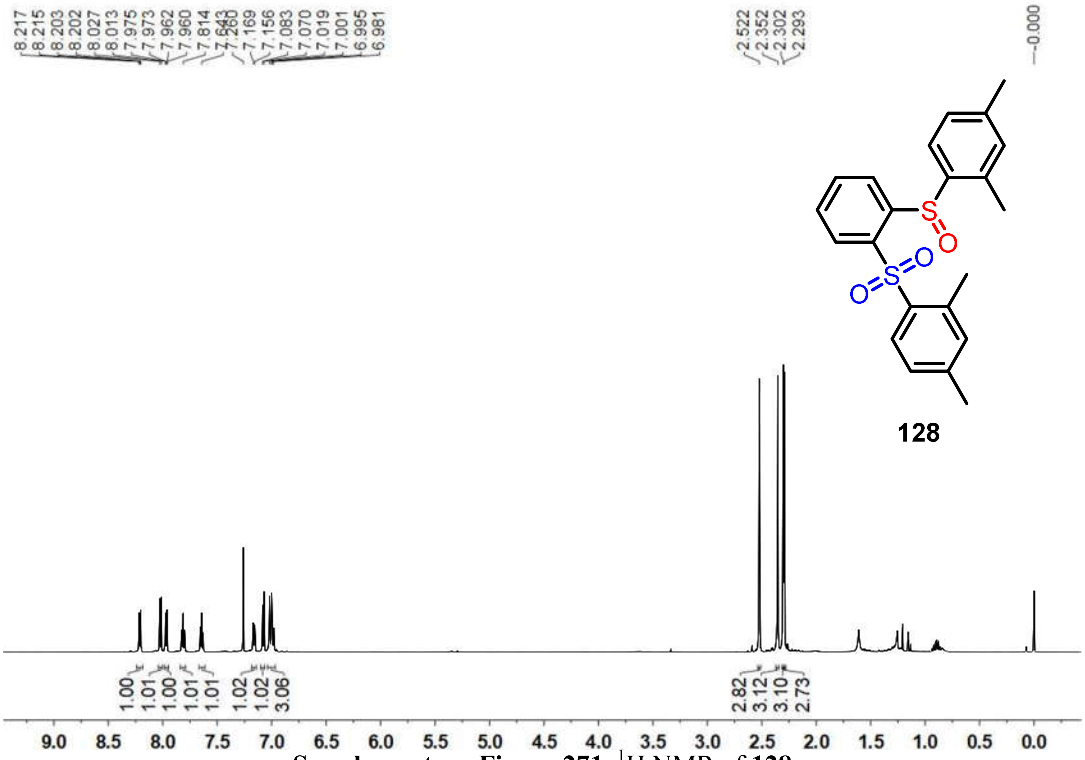

128

Supplementary Figure 271. ^{1}H NMR of 128

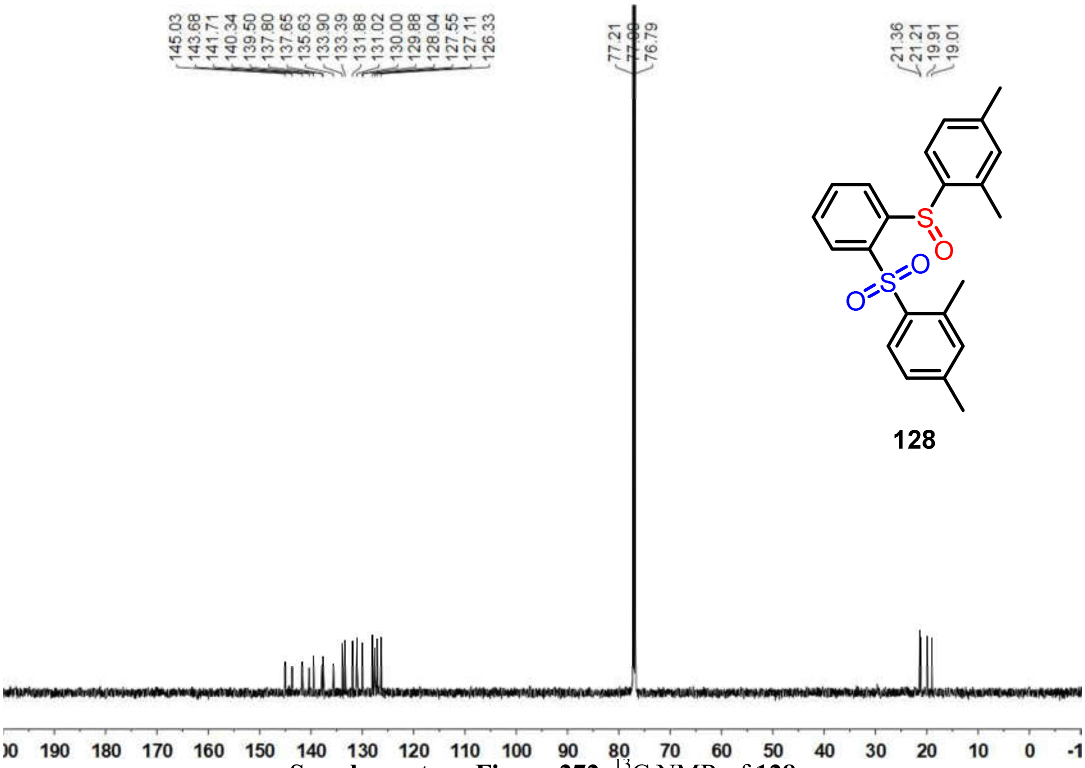

128

Supplementary Figure 272. ^{13}C NMR of 128

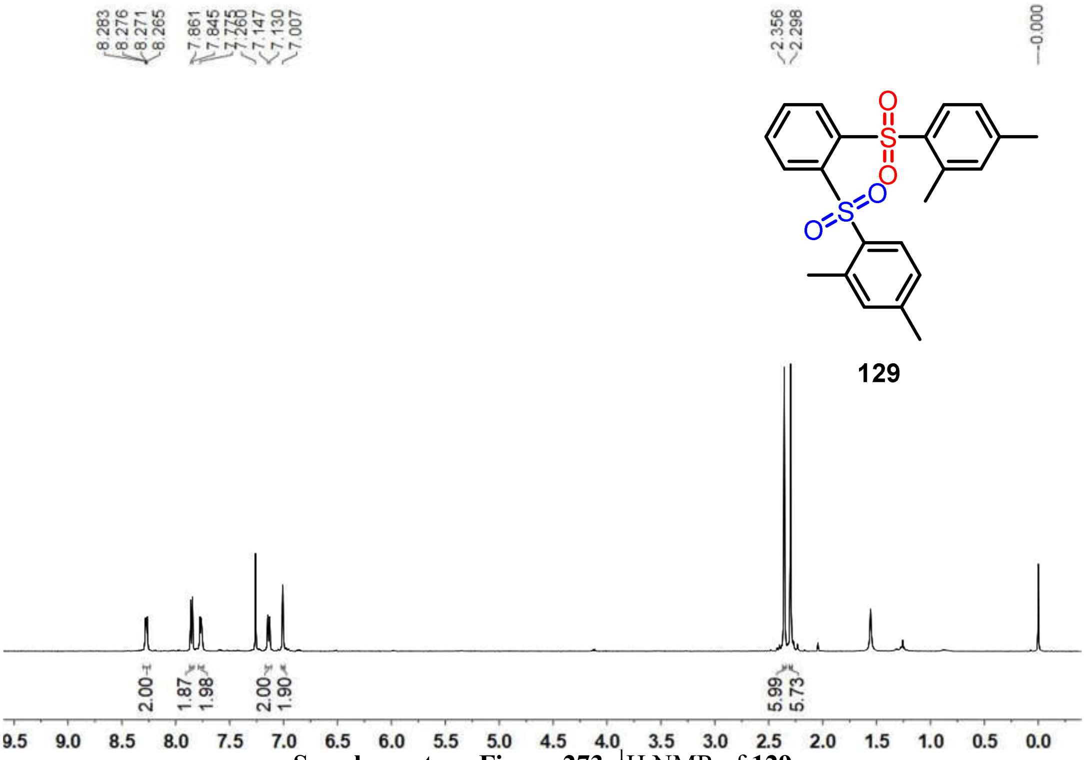

Supplementary Figure 273. ^{1}H NMR of **129**

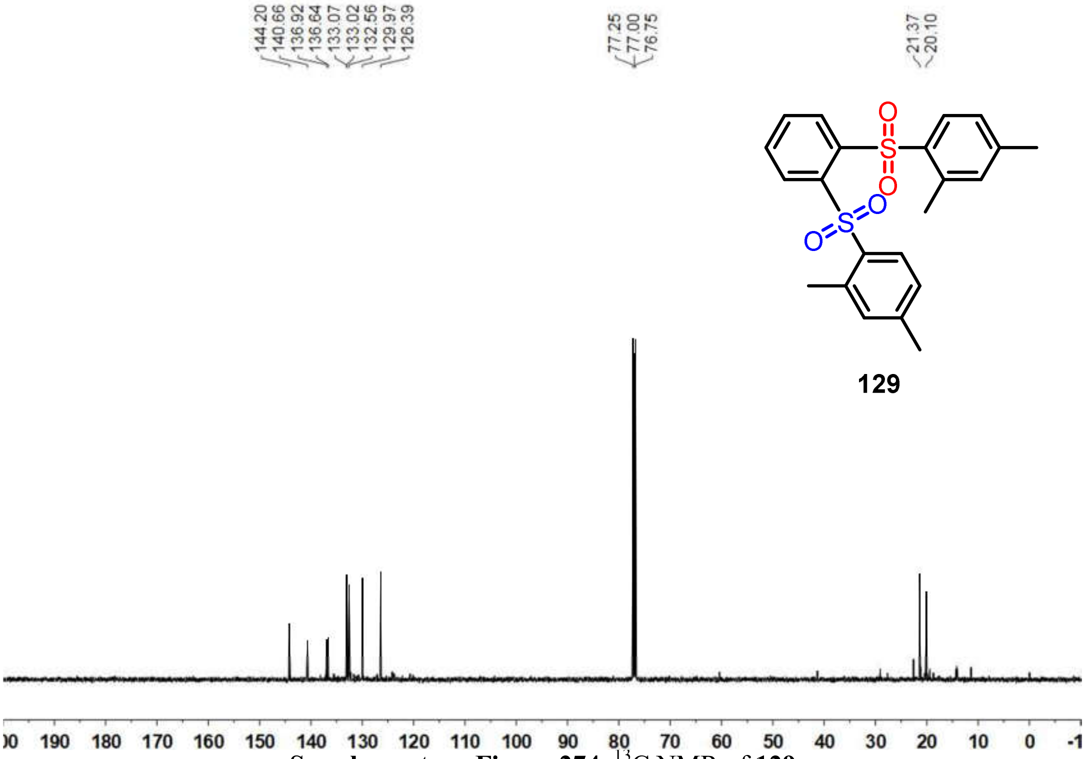

Supplementary Figure 274. ^{13}C NMR of **129**

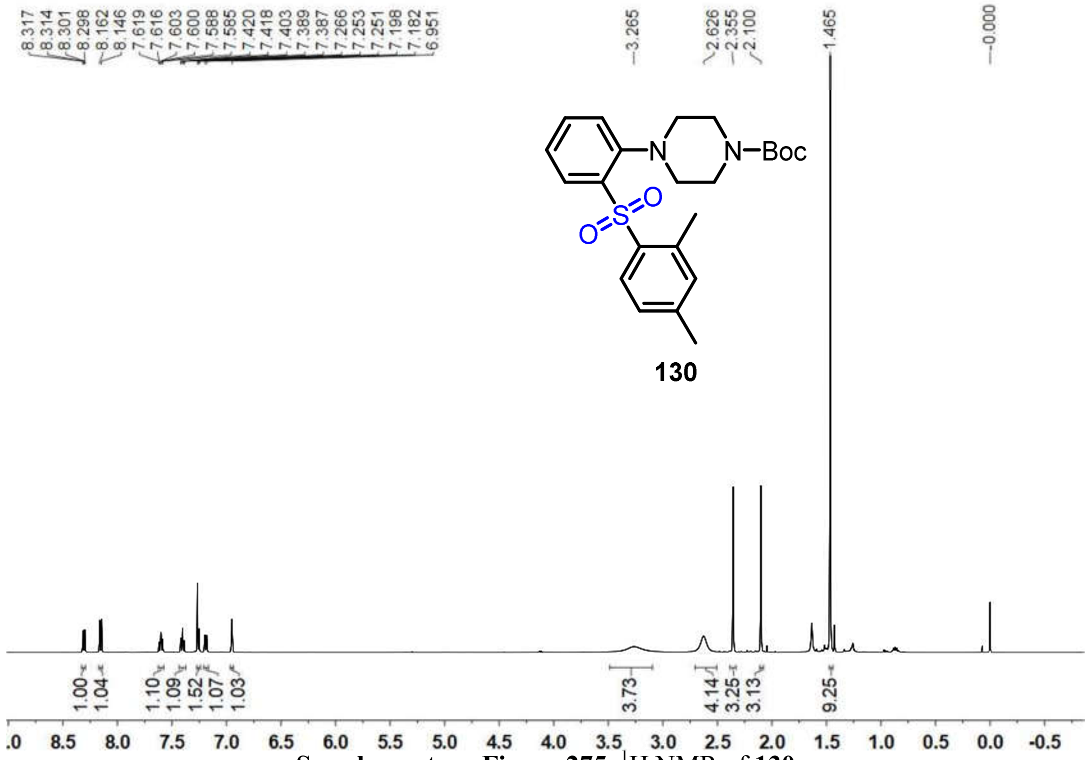

Supplementary Figure 275. ^{1}H NMR of **130**

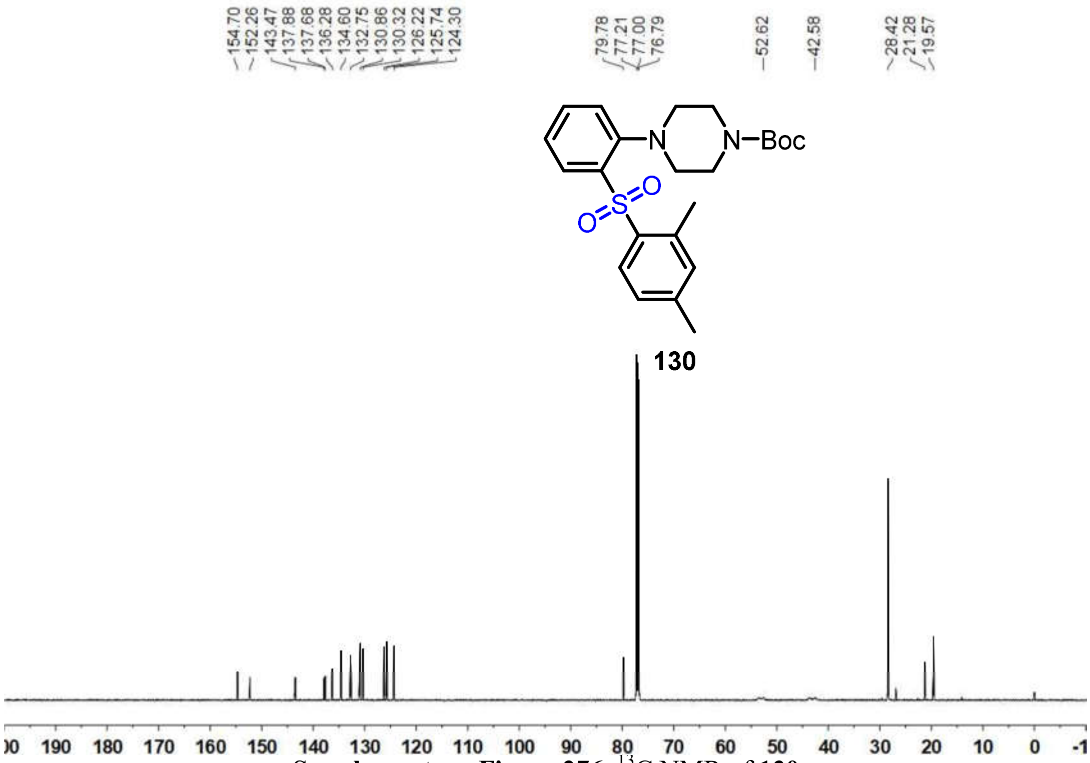

Supplementary Figure 276. ^{13}C NMR of **130**

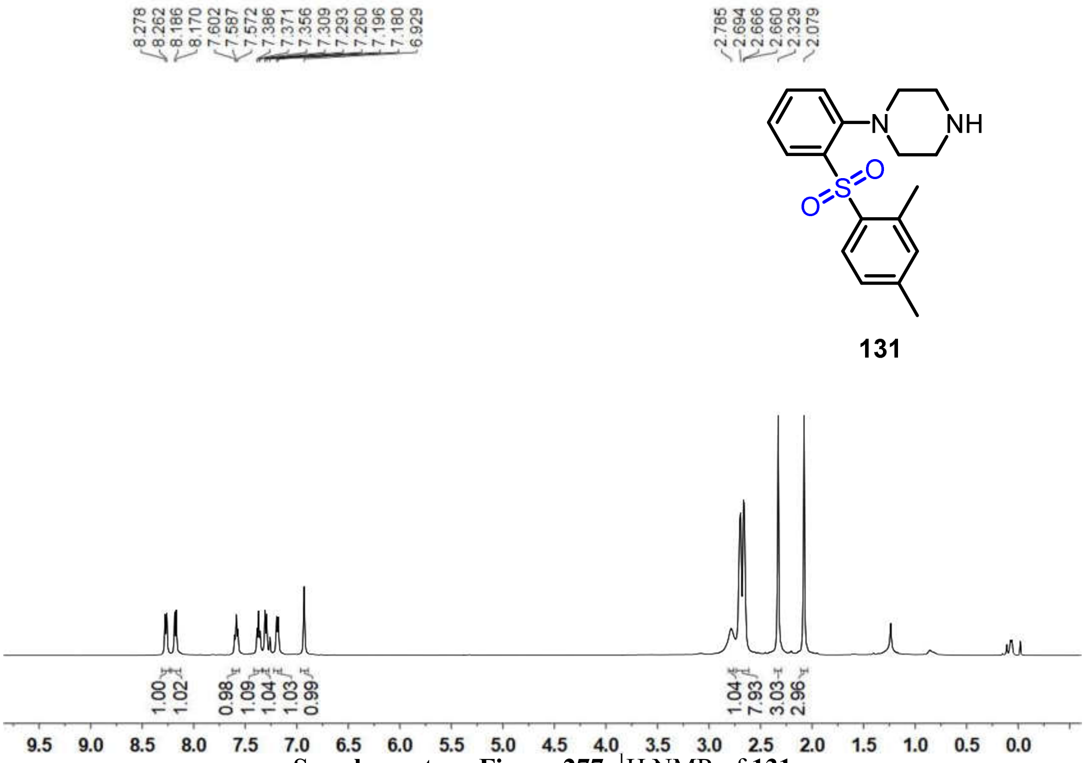

Supplementary Figure 277. ^{1}H NMR of **131**

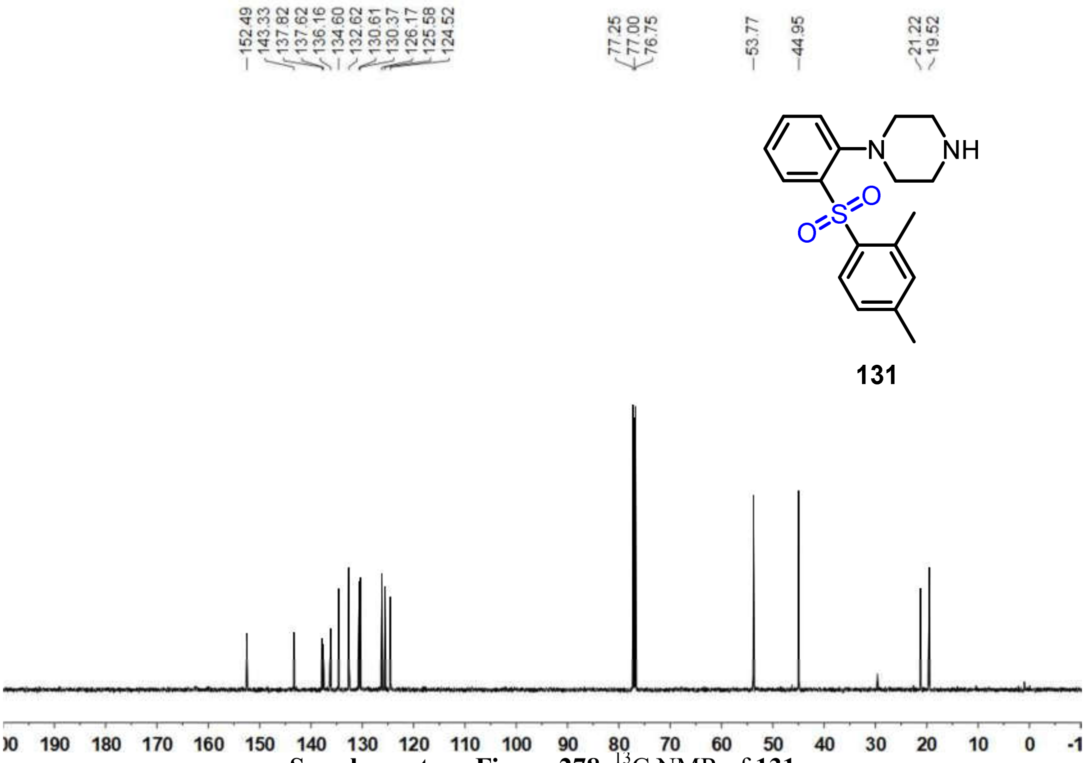

Supplementary Figure 278. ^{13}C NMR of **131**

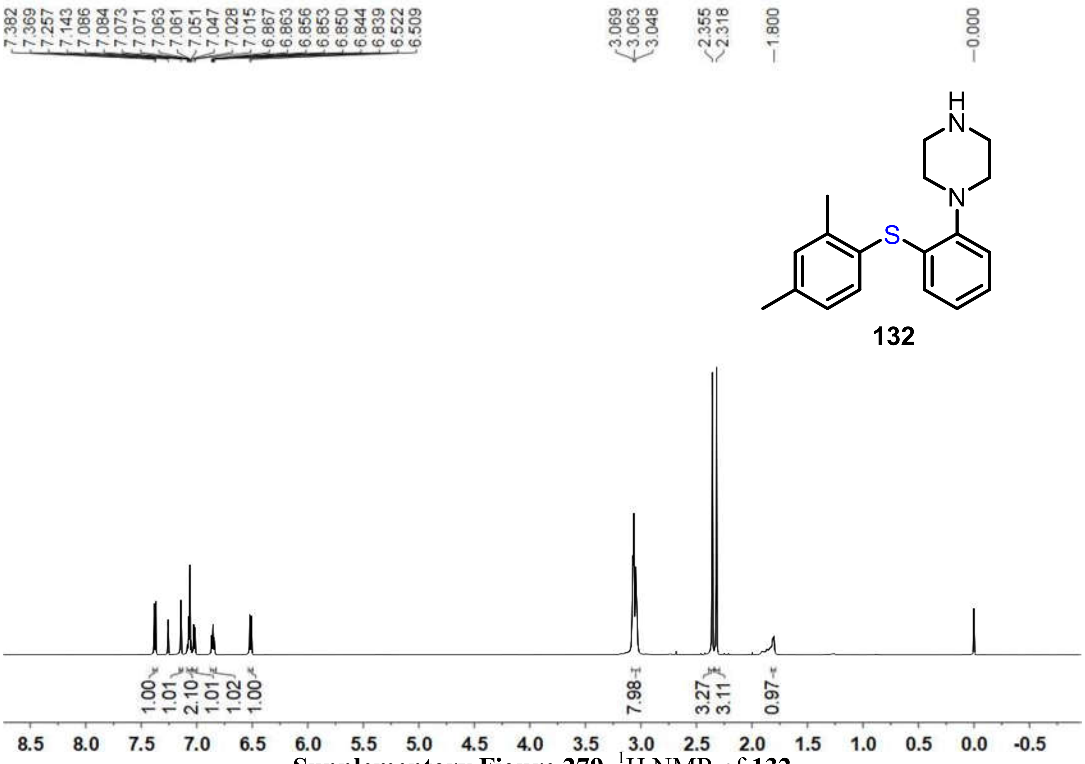

Supplementary Figure 279. ^{1}H NMR of **132**

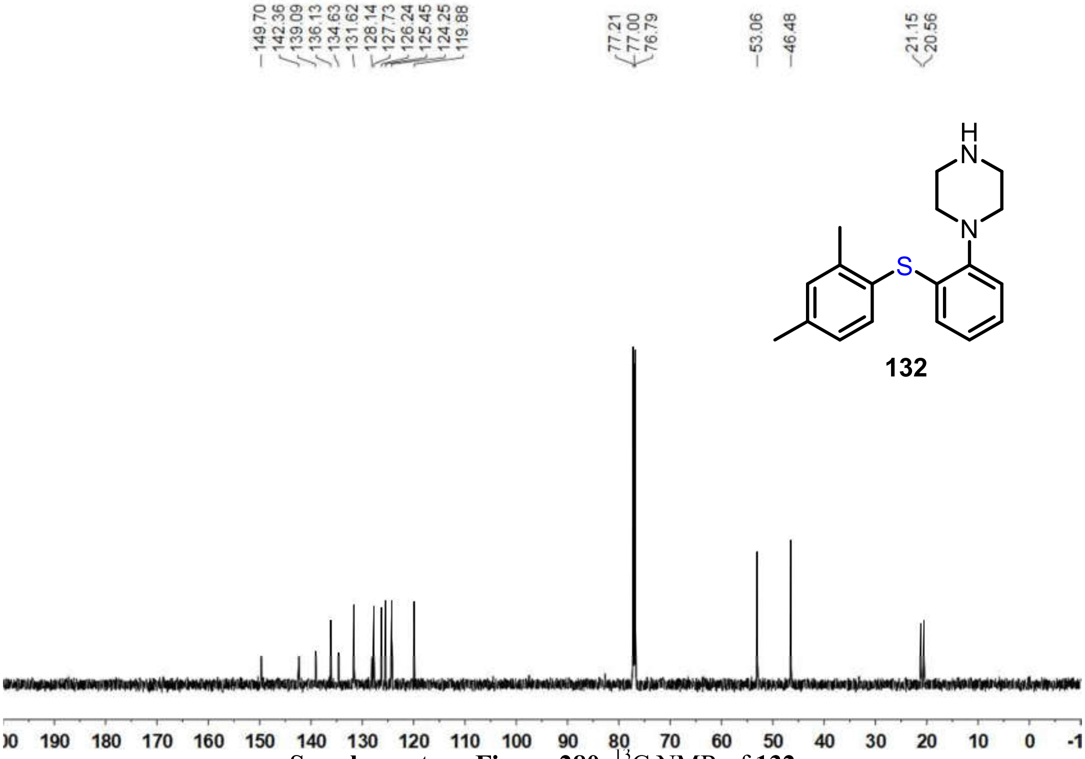

Supplementary Figure 280. ^{13}C NMR of **132**

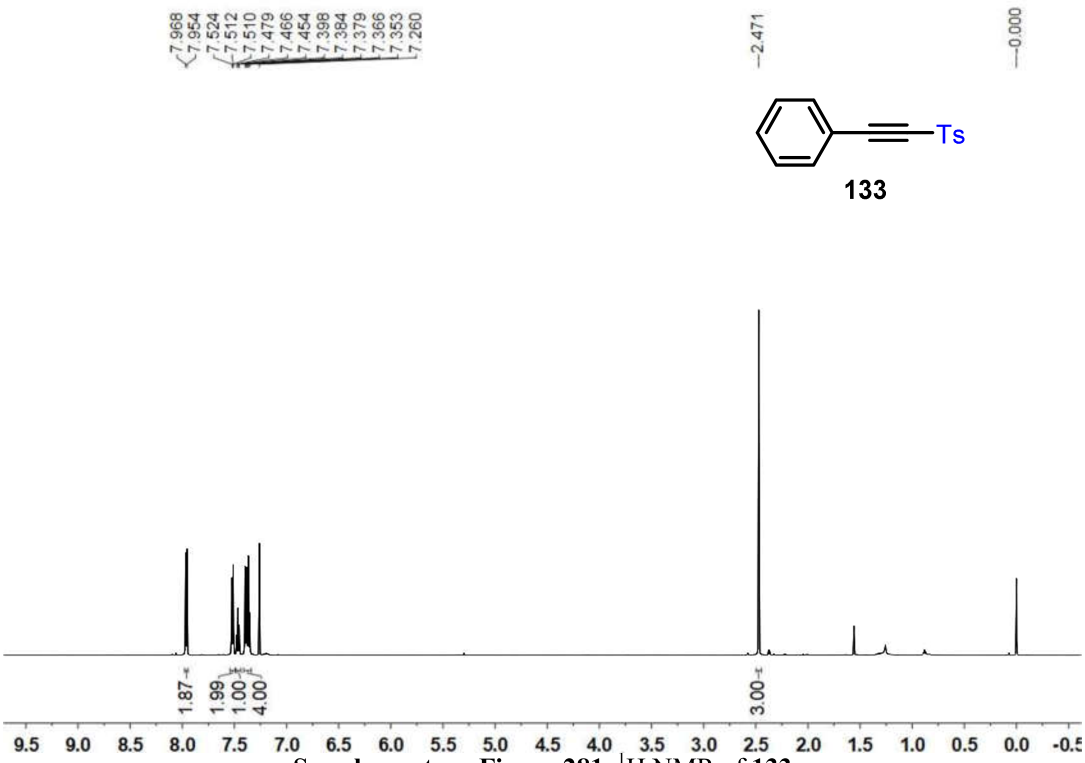

Supplementary Figure 281. ^{1}H NMR of **133**

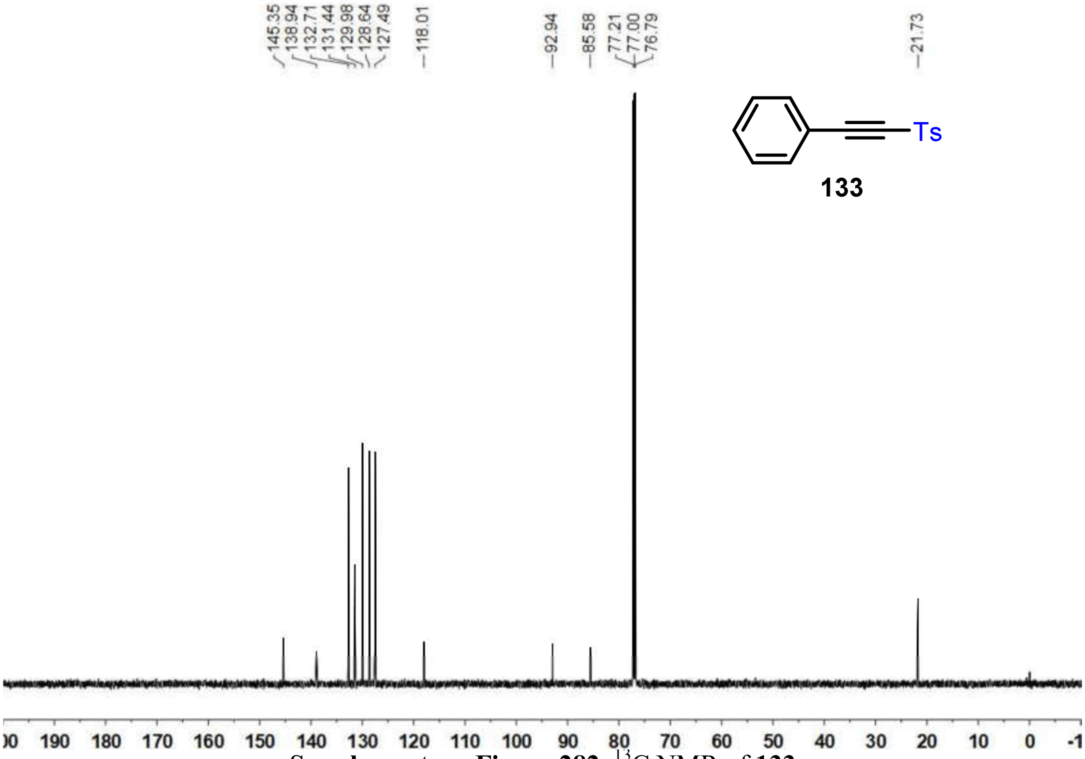

Supplementary Figure 282. ^{13}C NMR of **133**

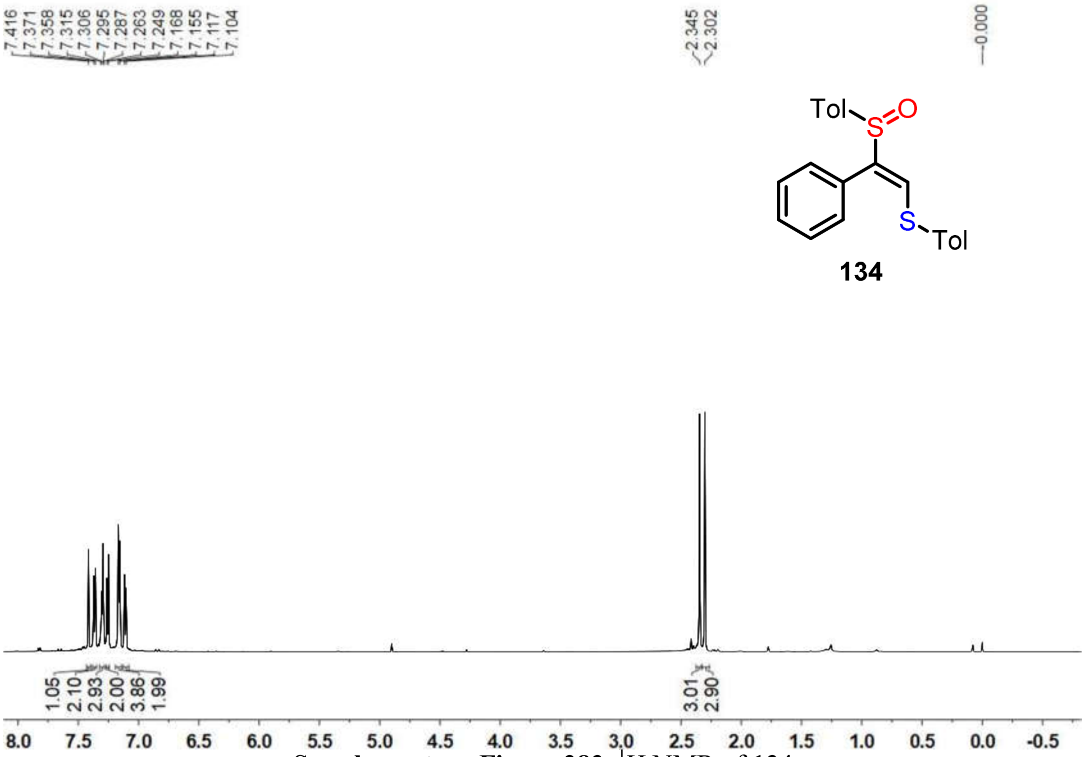

Supplementary Figure 283. ^{1}H NMR of **134**

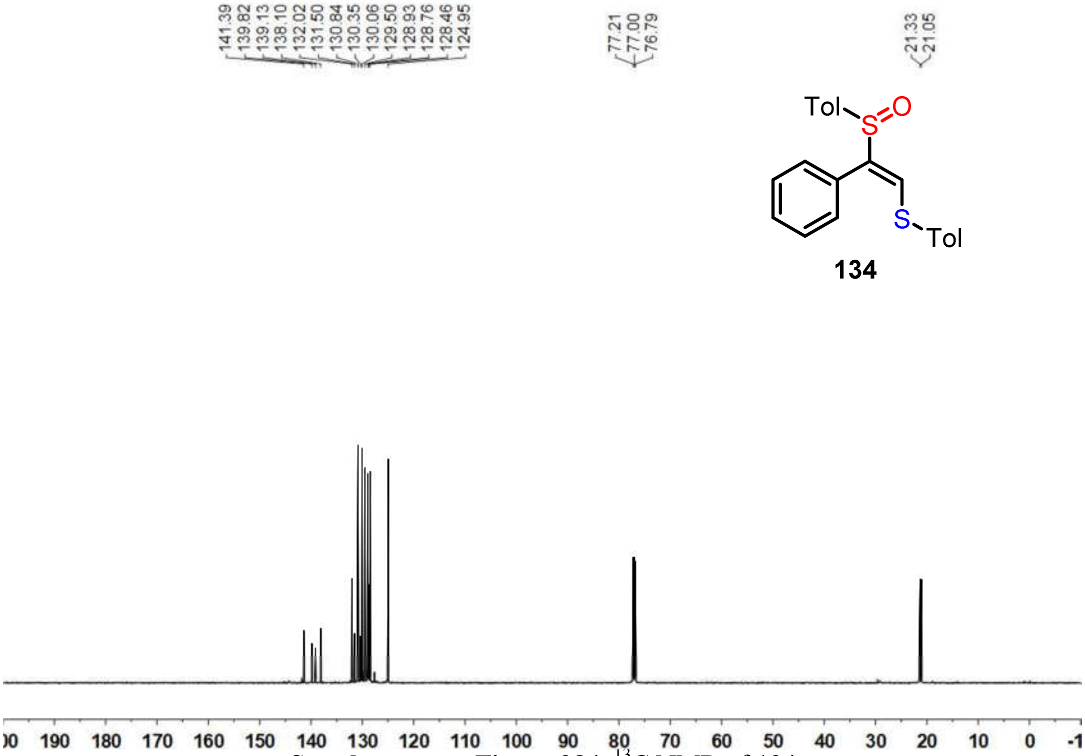

Supplementary Figure 284. ^{13}C NMR of **134**

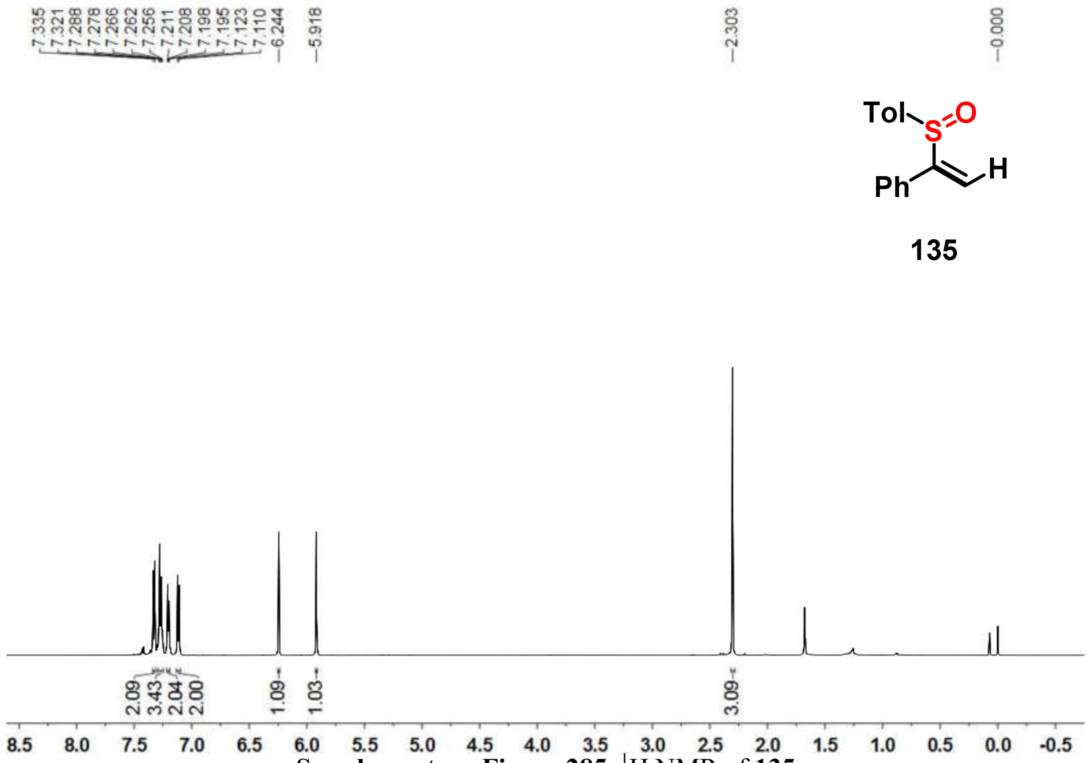

Supplementary Figure 285. ^{1}H NMR of **135**

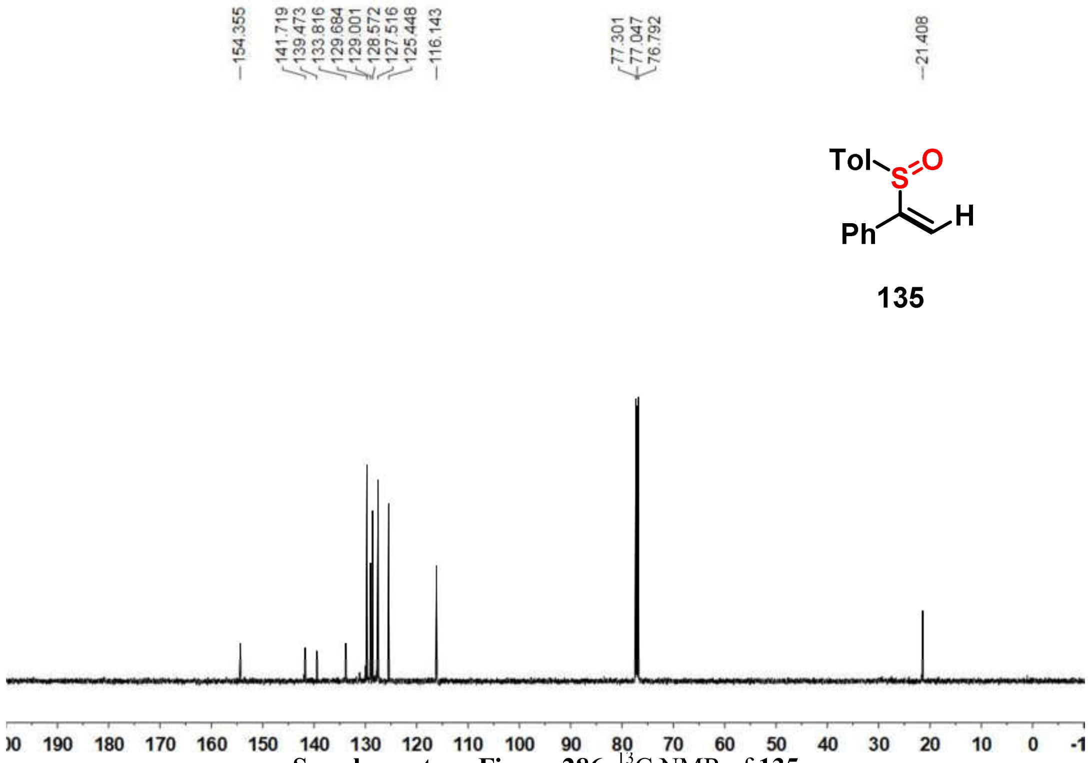

Supplementary Figure 286. ^{13}C NMR of **135**

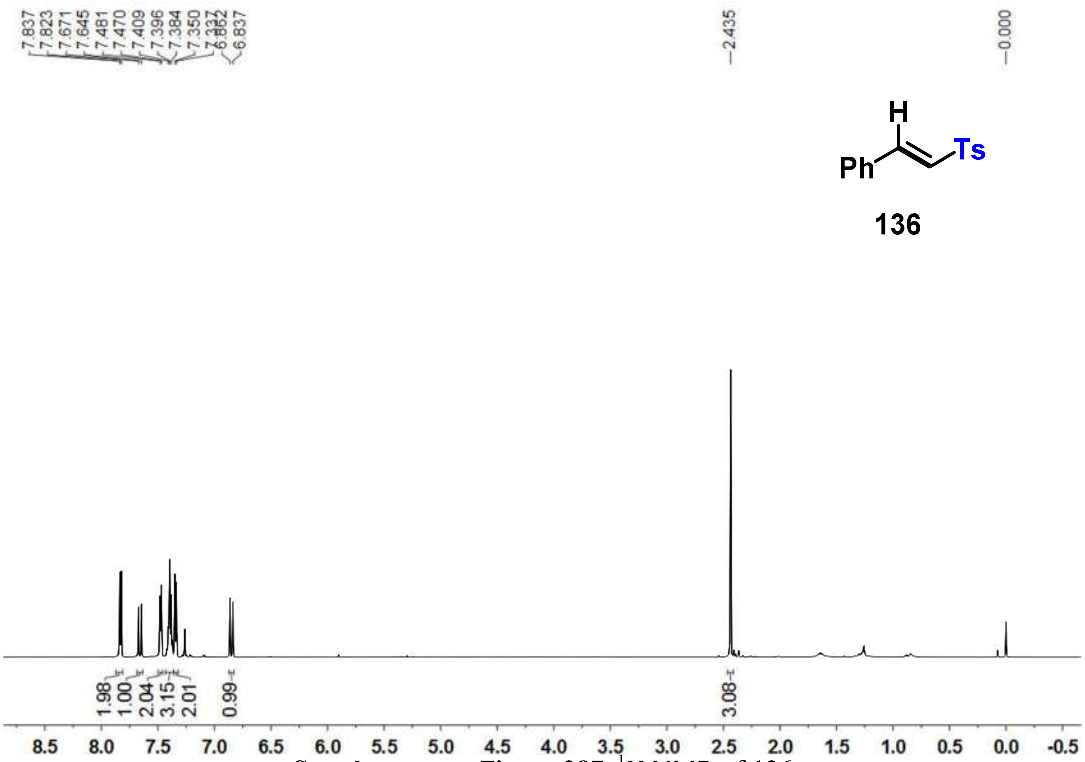

Supplementary Figure 287. ^{1}H NMR of **136**

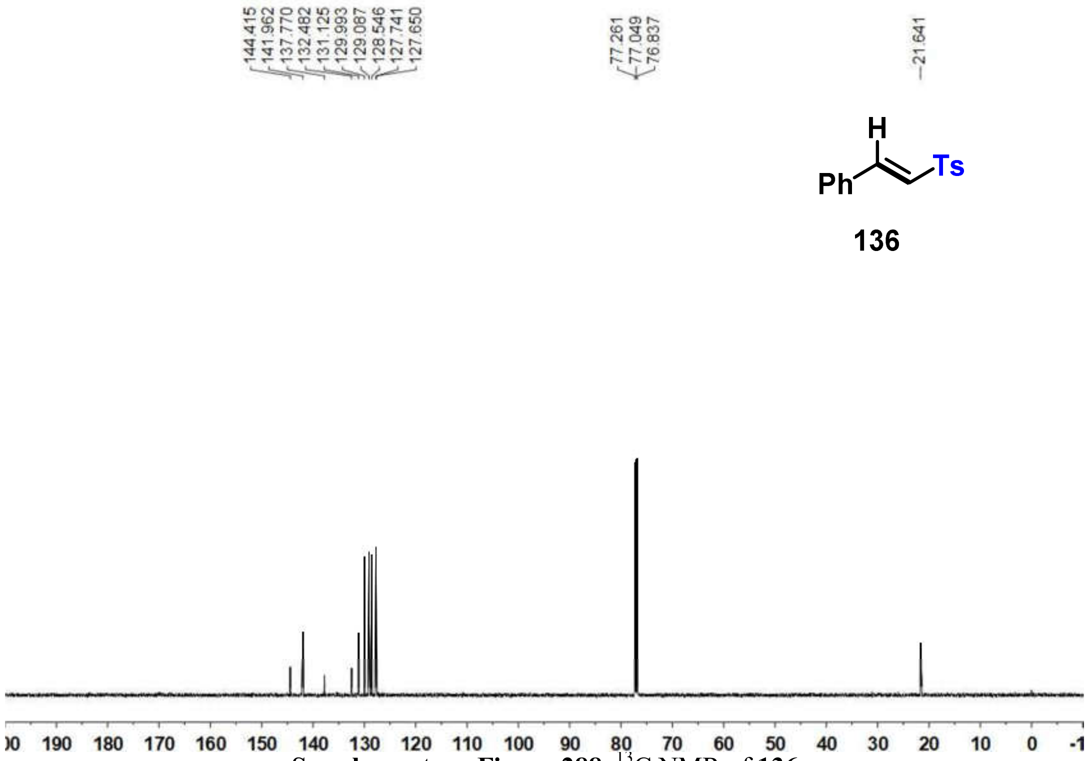

Supplementary Figure 288. ^{13}C NMR of **136**

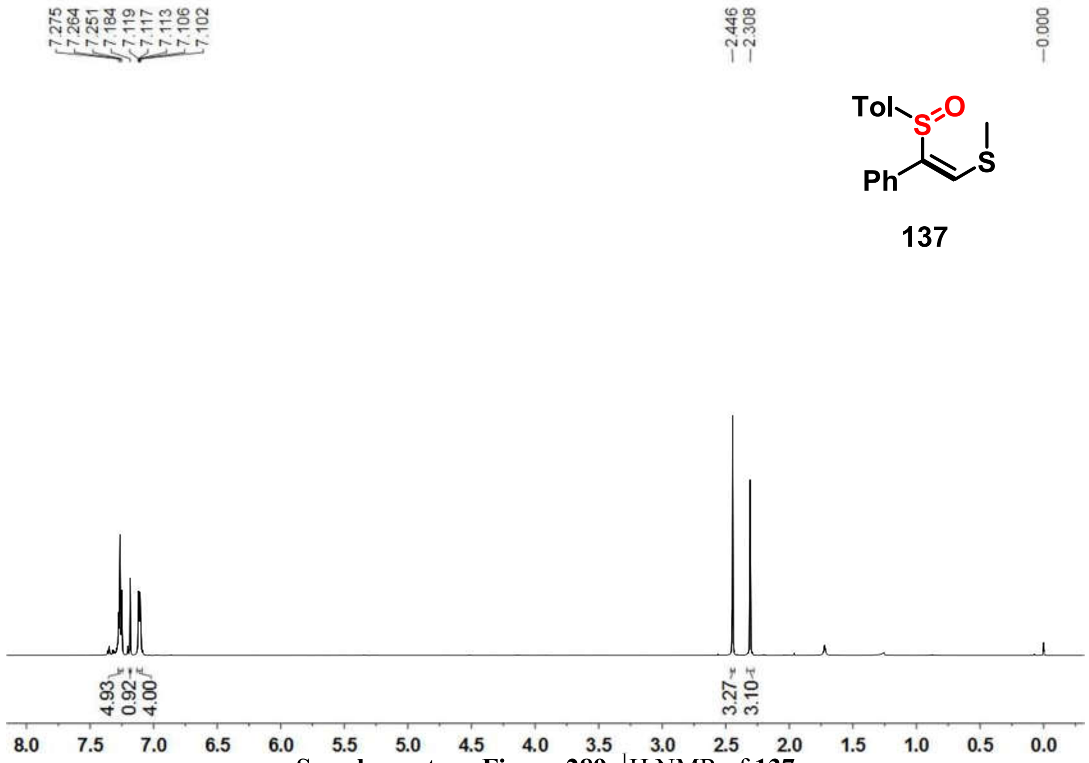

Supplementary Figure 289. ^{1}H NMR of **137**

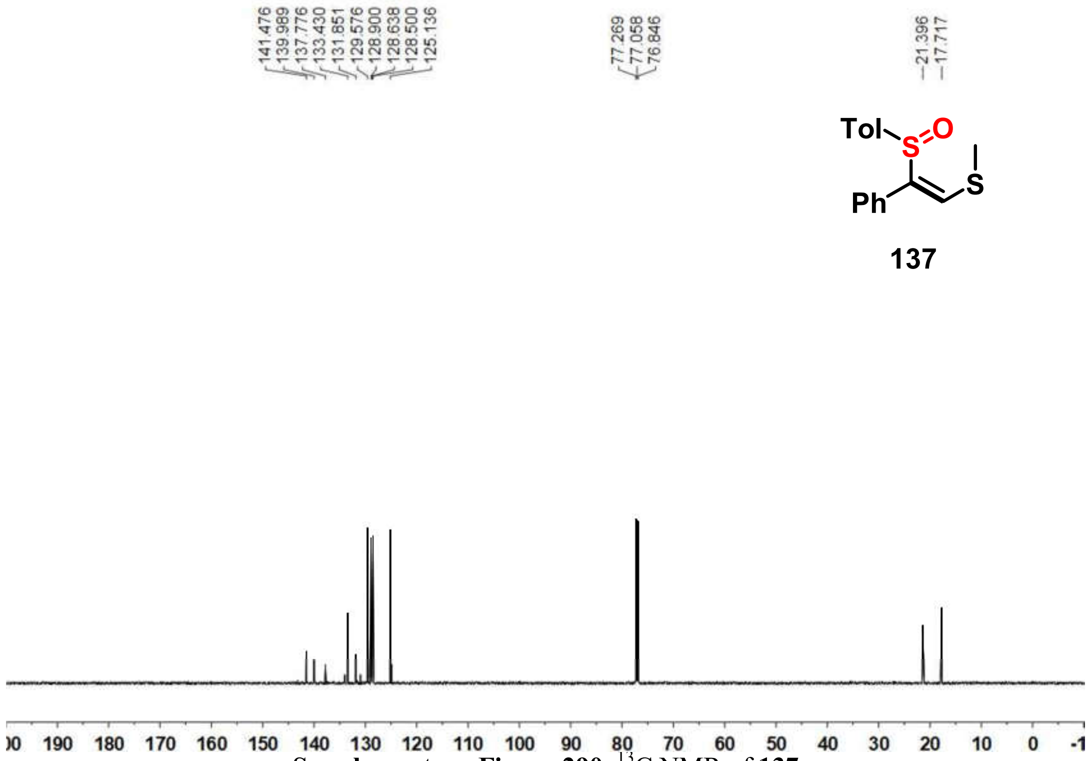

Supplementary Figure 290. ^{13}C NMR of **137**

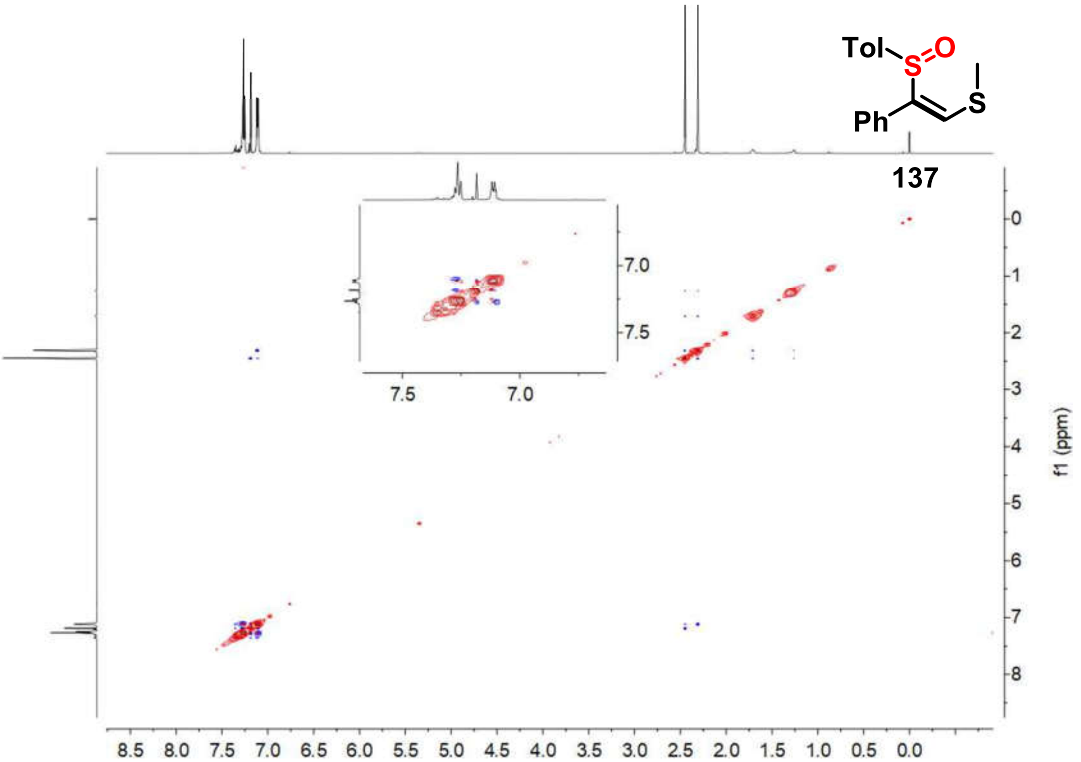

Supplementary Figure 291. NOESY Spectrum of compound **137**.

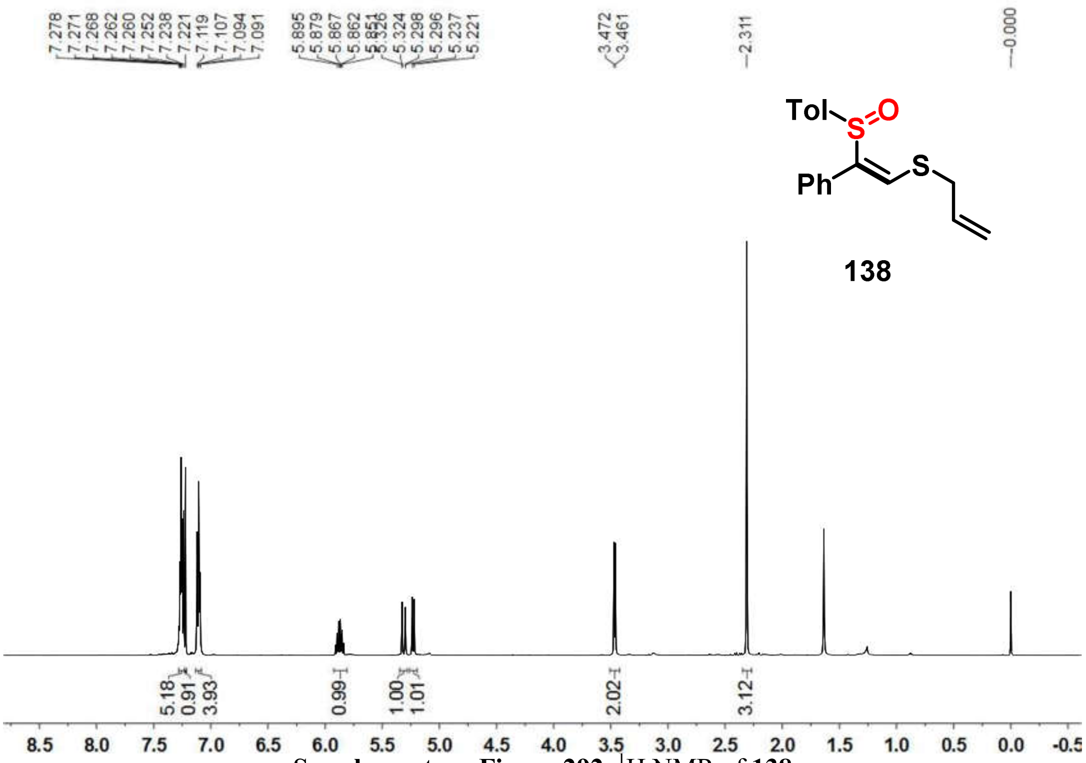

Supplementary Figure 292. ^{1}H NMR of **138**

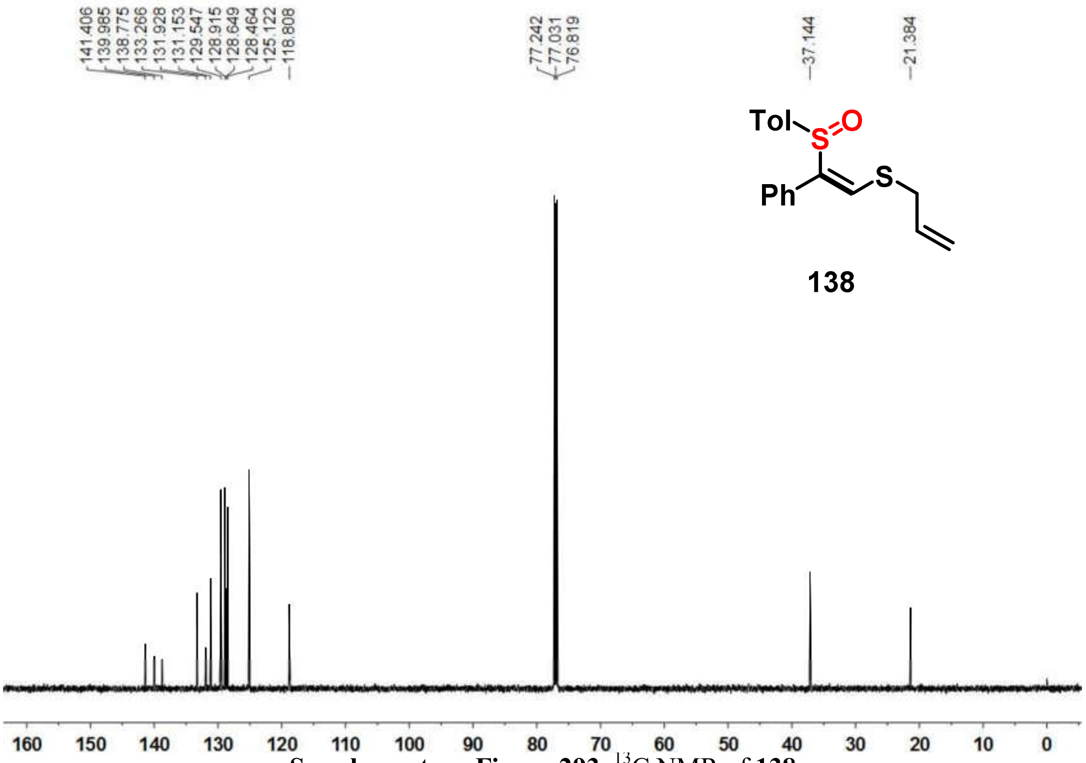

Supplementary Figure 293. ^{13}C NMR of **138**

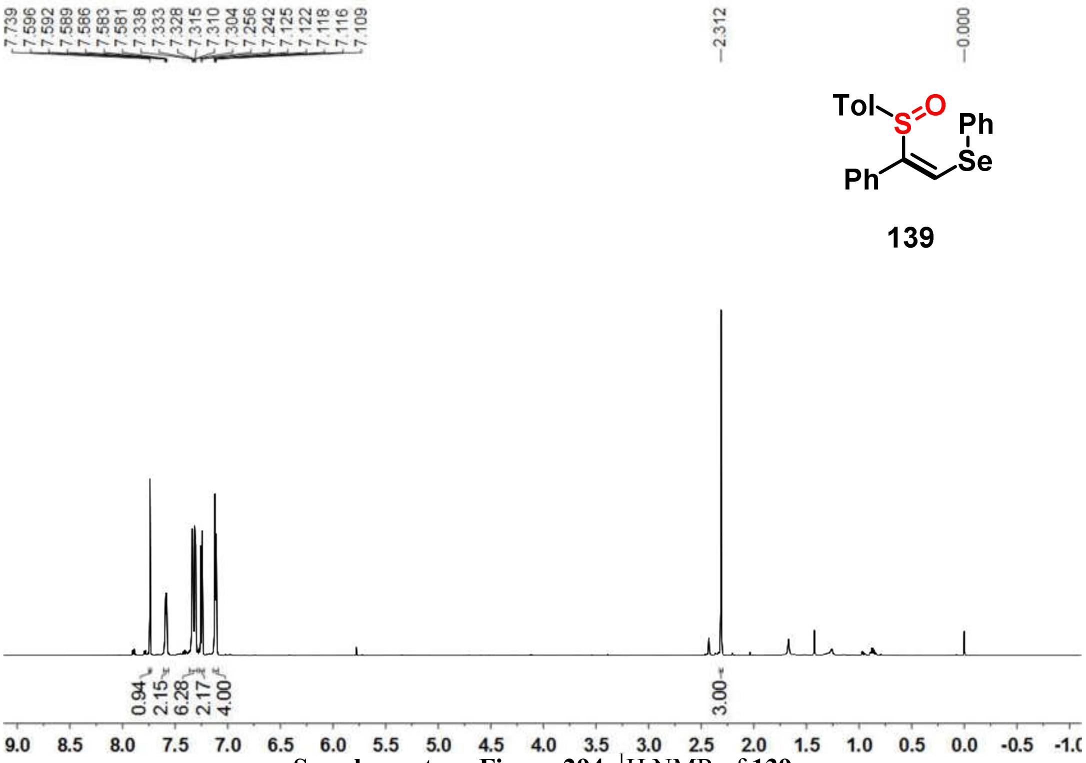

Supplementary Figure 294. ^{1}H NMR of **139**

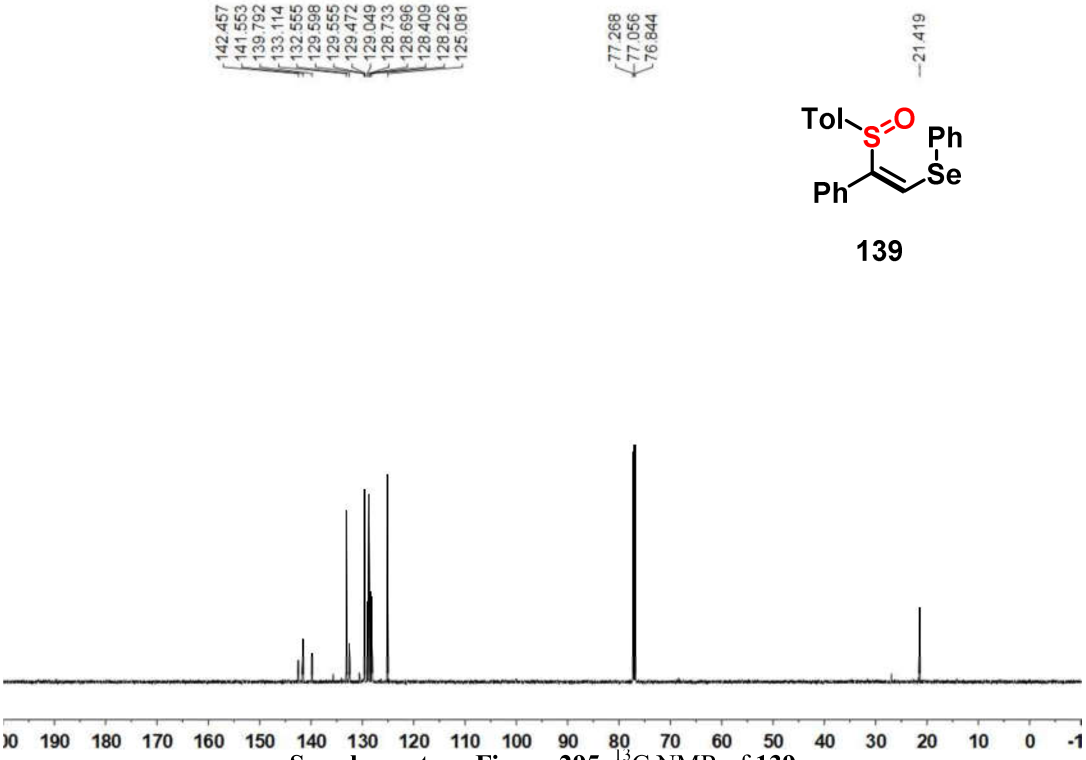

Supplementary Figure 295. ^{13}C NMR of **139**

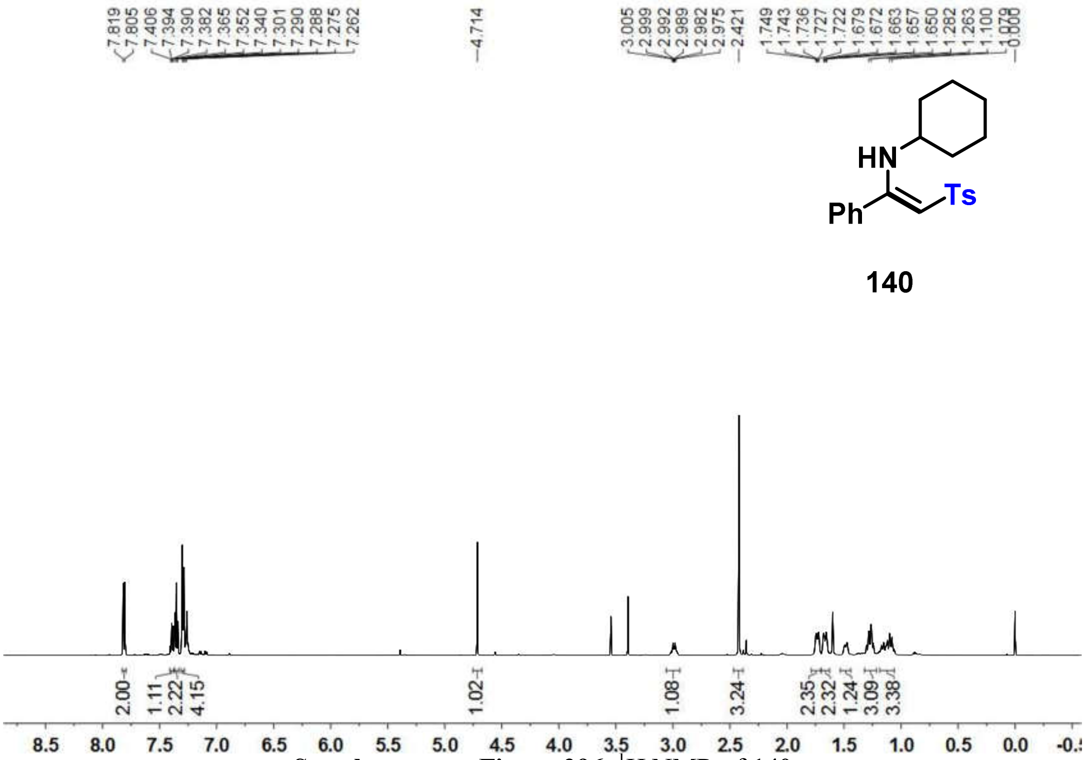

Supplementary Figure 296. ^{1}H NMR of **140**

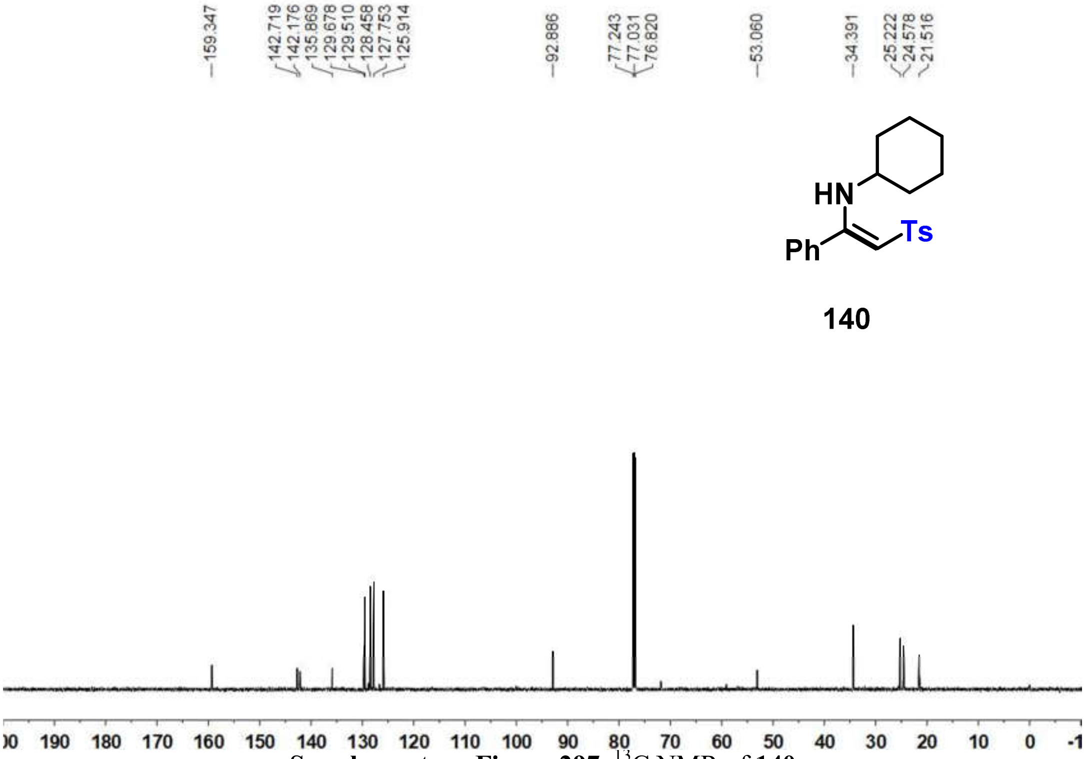

Supplementary Figure 297. ^{13}C NMR of **140**

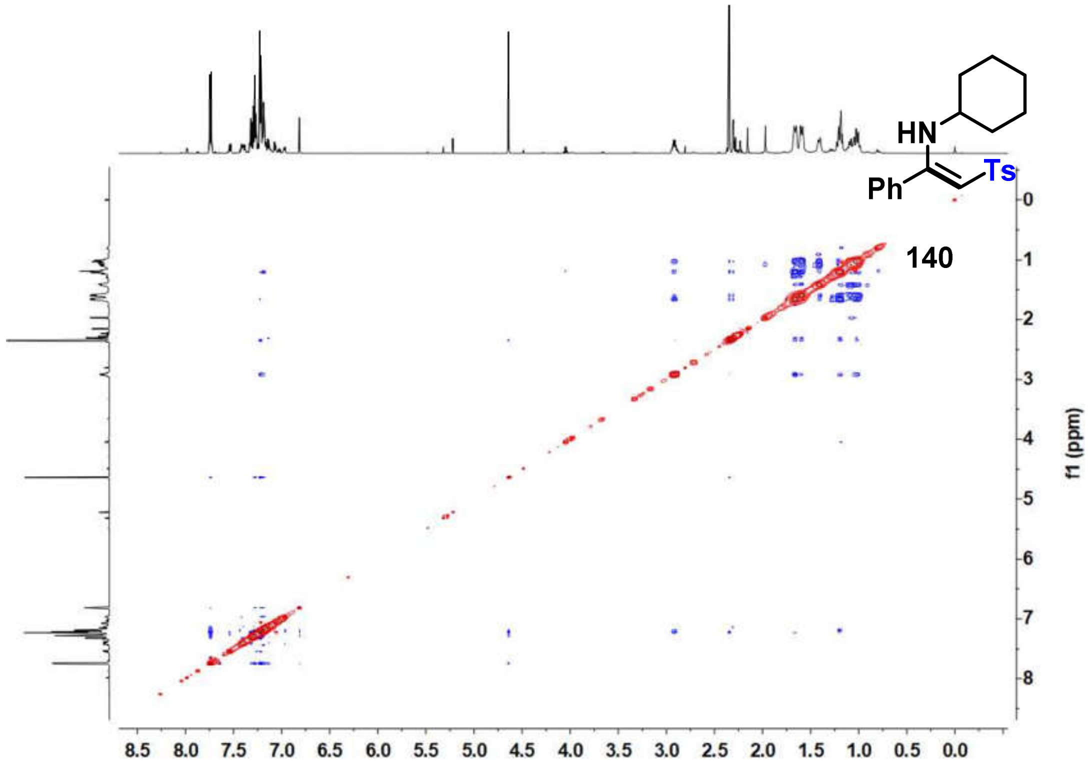

Supplementary Figure 298. NOESY Spectrum of compound **140**

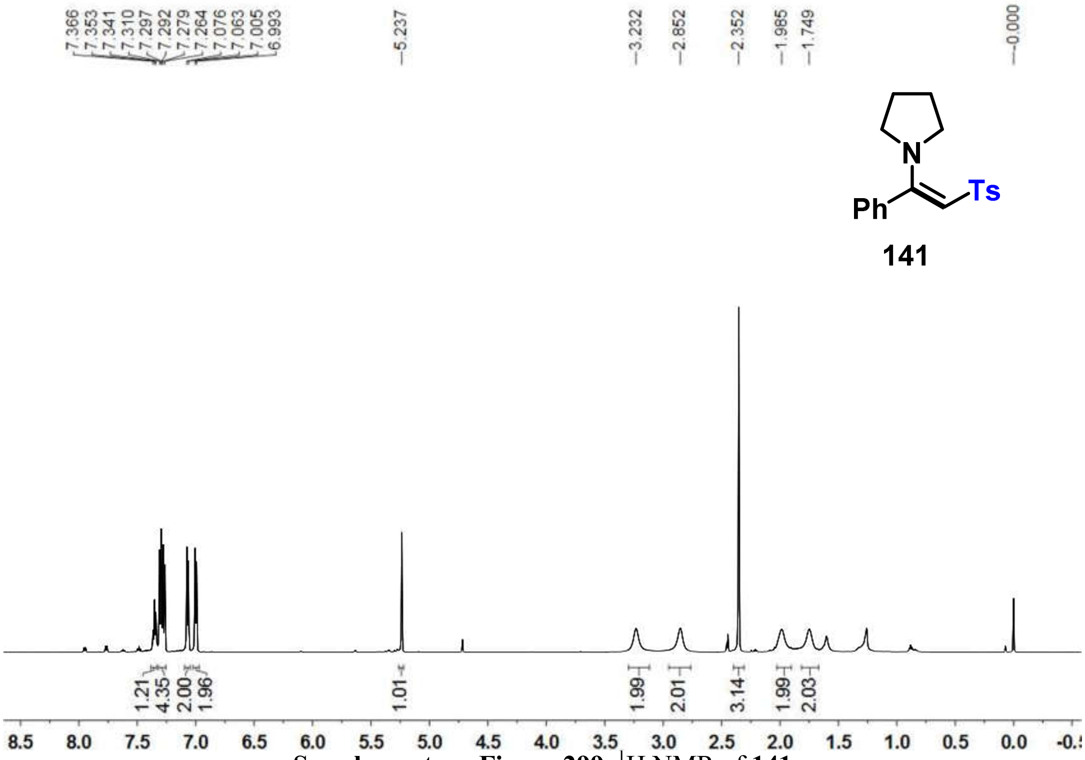

Supplementary Figure 299. ^{1}H NMR of **141**

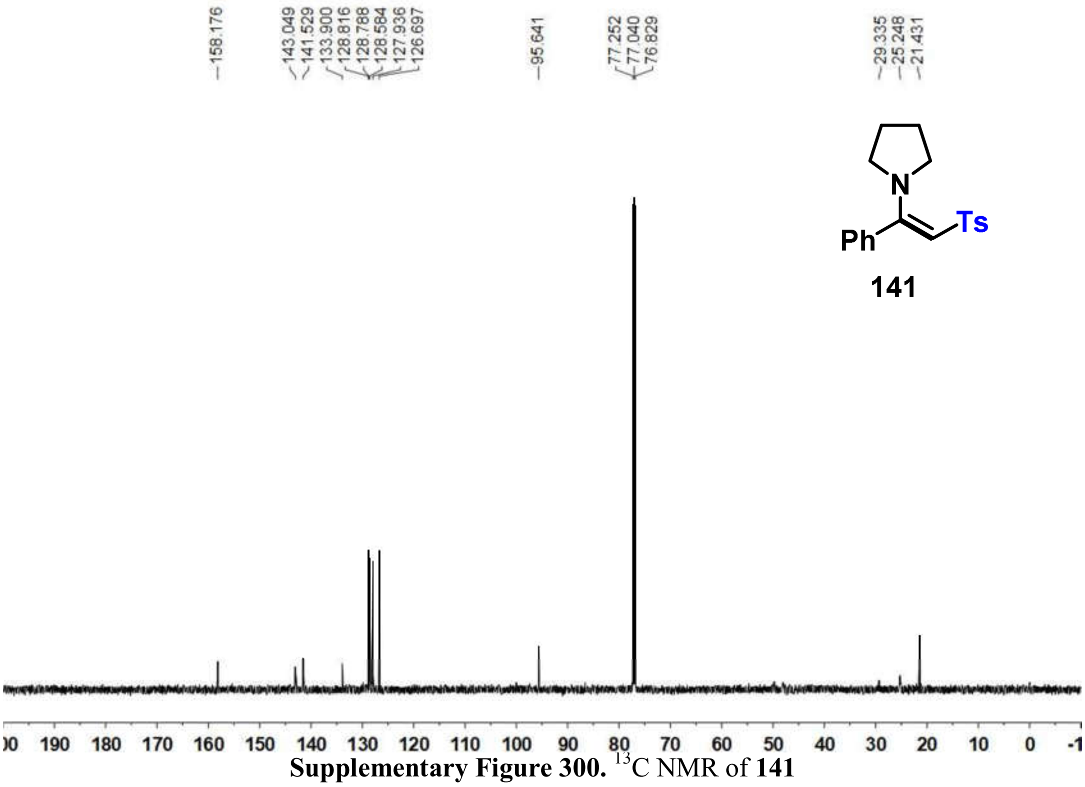

Supplementary Figure 300. ^{13}C NMR of **141**

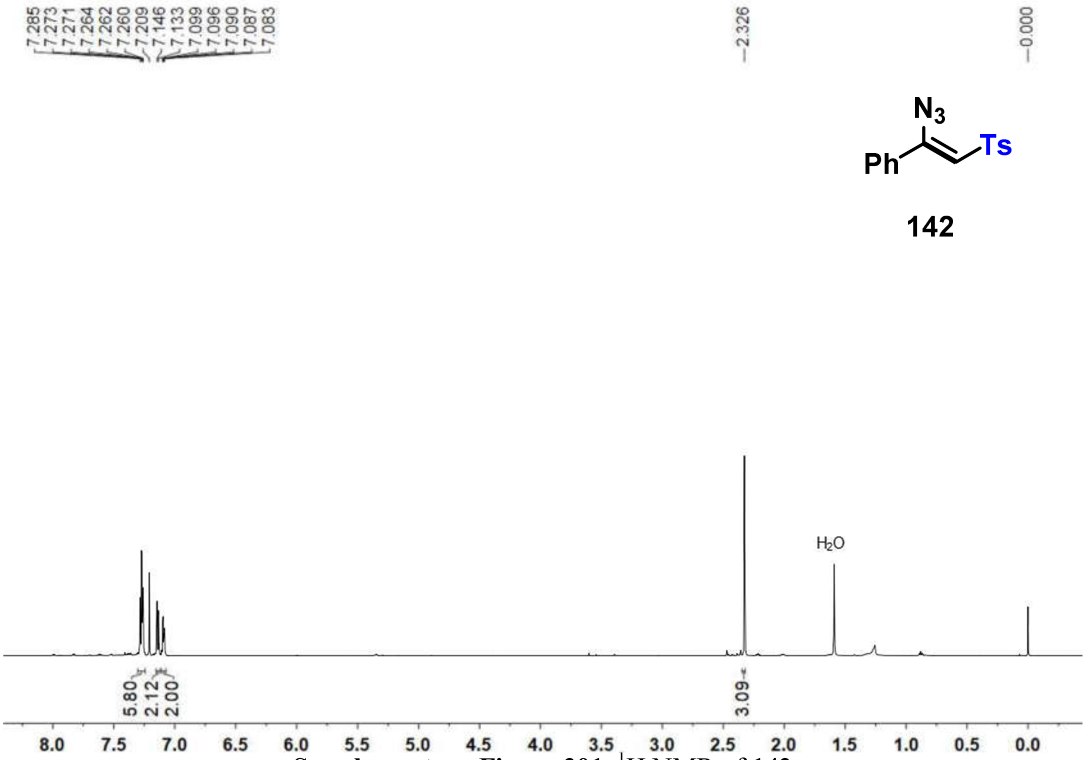

Supplementary Figure 301. ^{1}H NMR of **142**

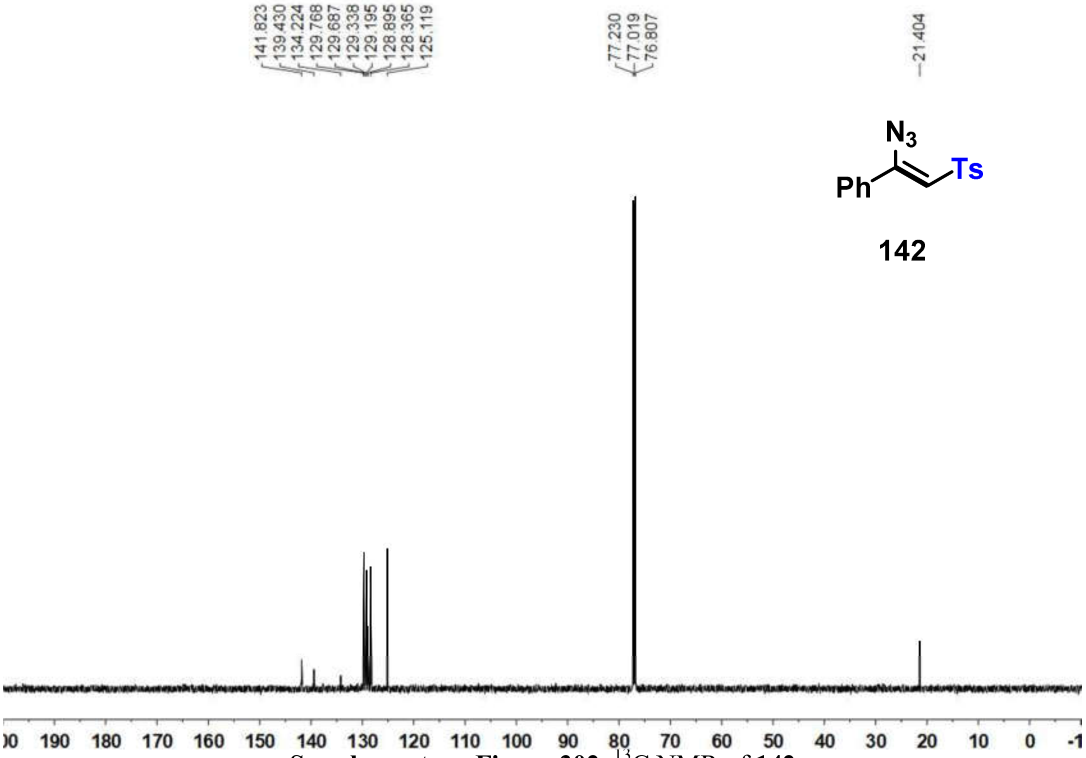

Supplementary Figure 302. ^{13}C NMR of **142**

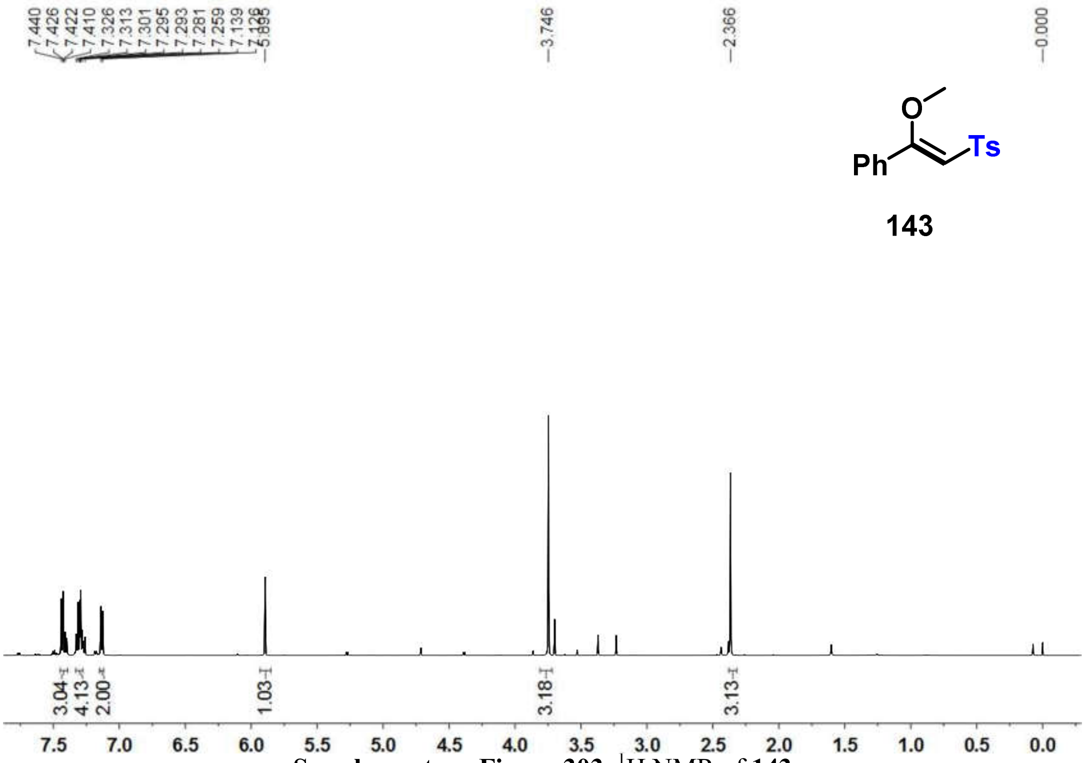

Supplementary Figure 303. ^{1}H NMR of **143**

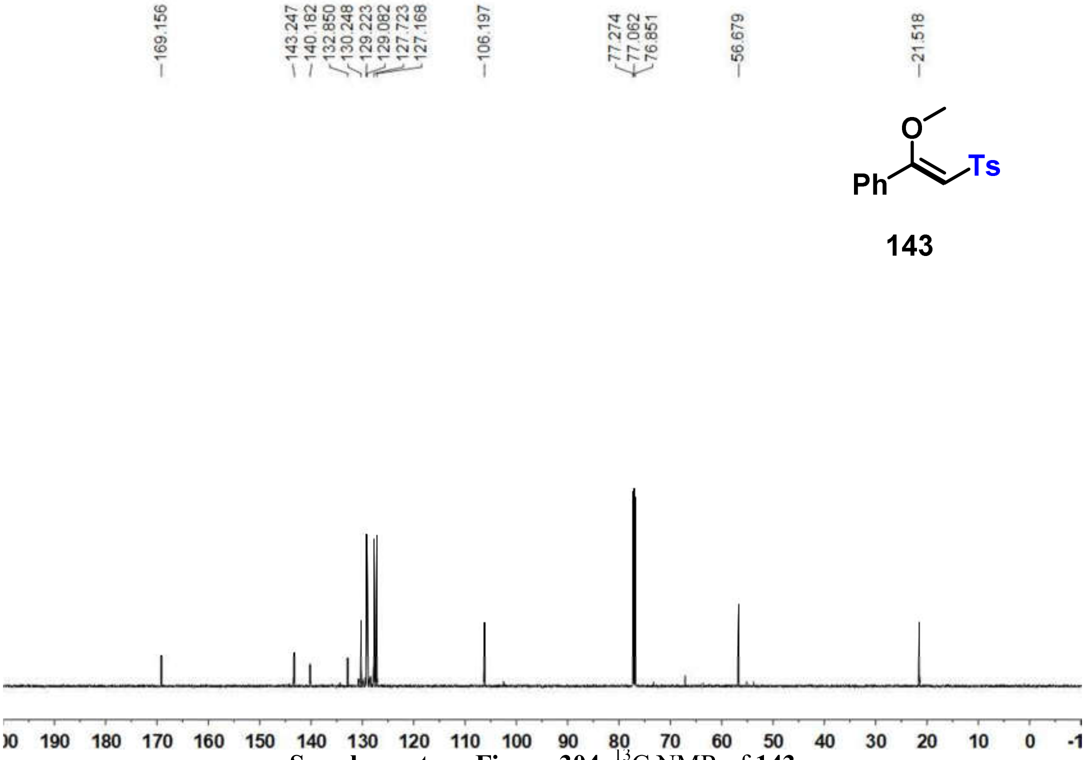

Supplementary Figure 304. ^{13}C NMR of **143**

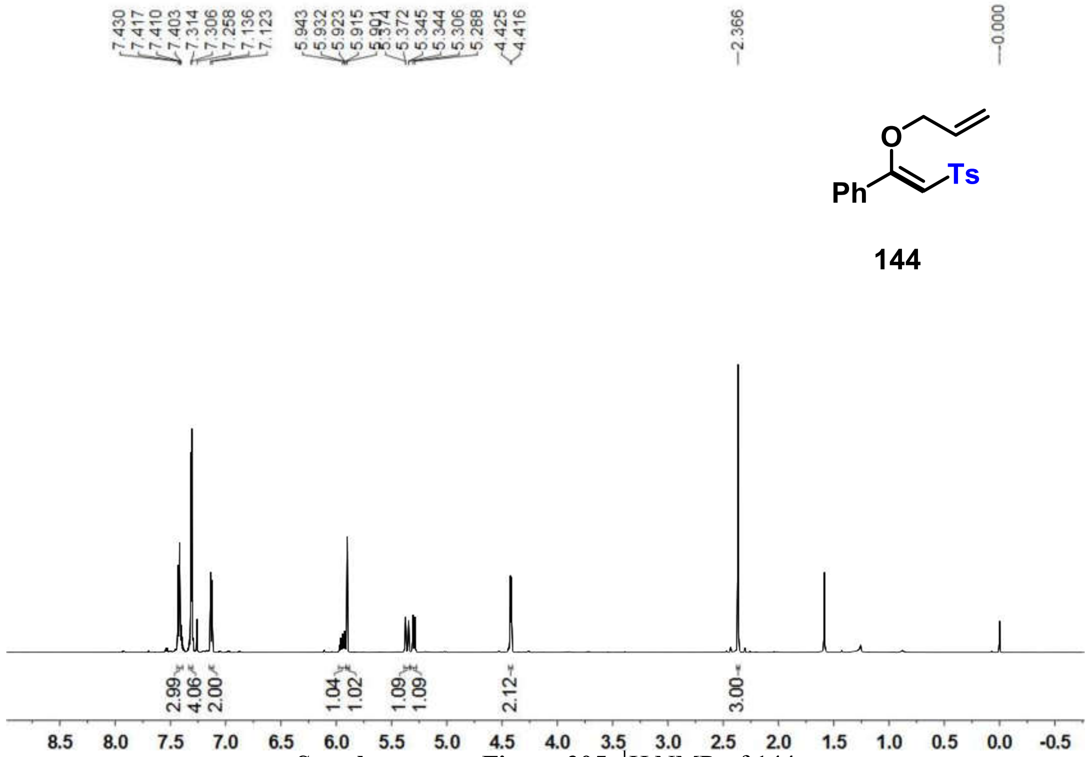

Supplementary Figure 305. ^{1}H NMR of **144**

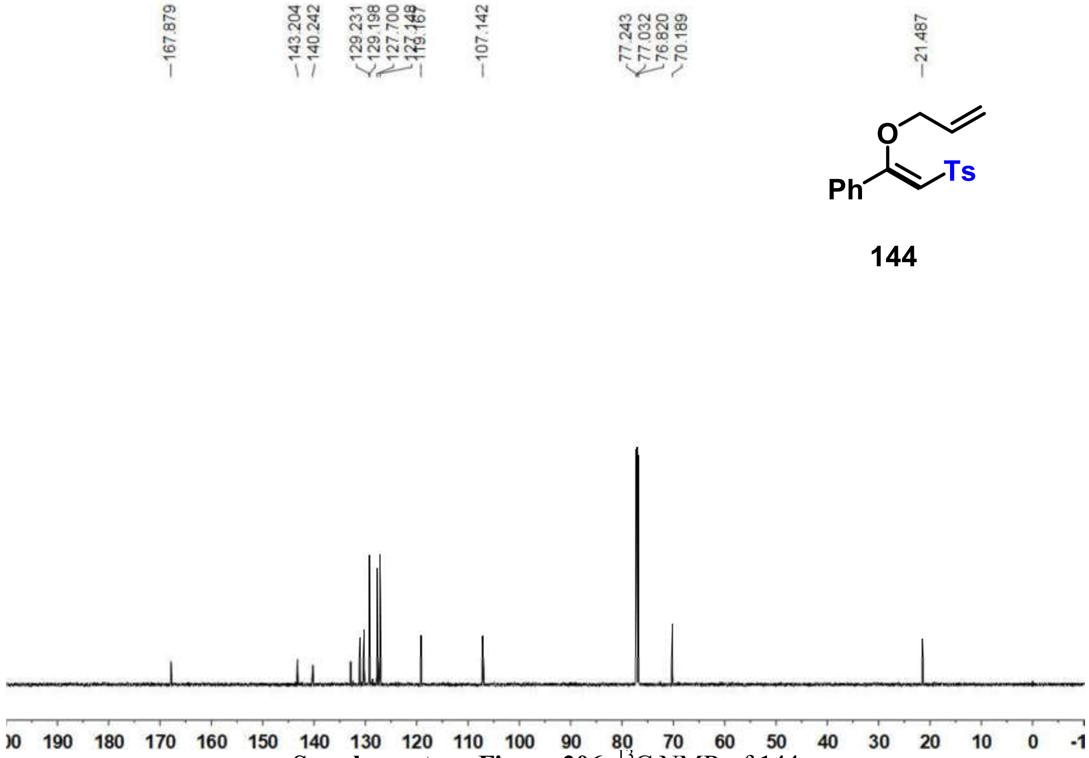

Supplementary Figure 306. ^{13}C NMR of **144**

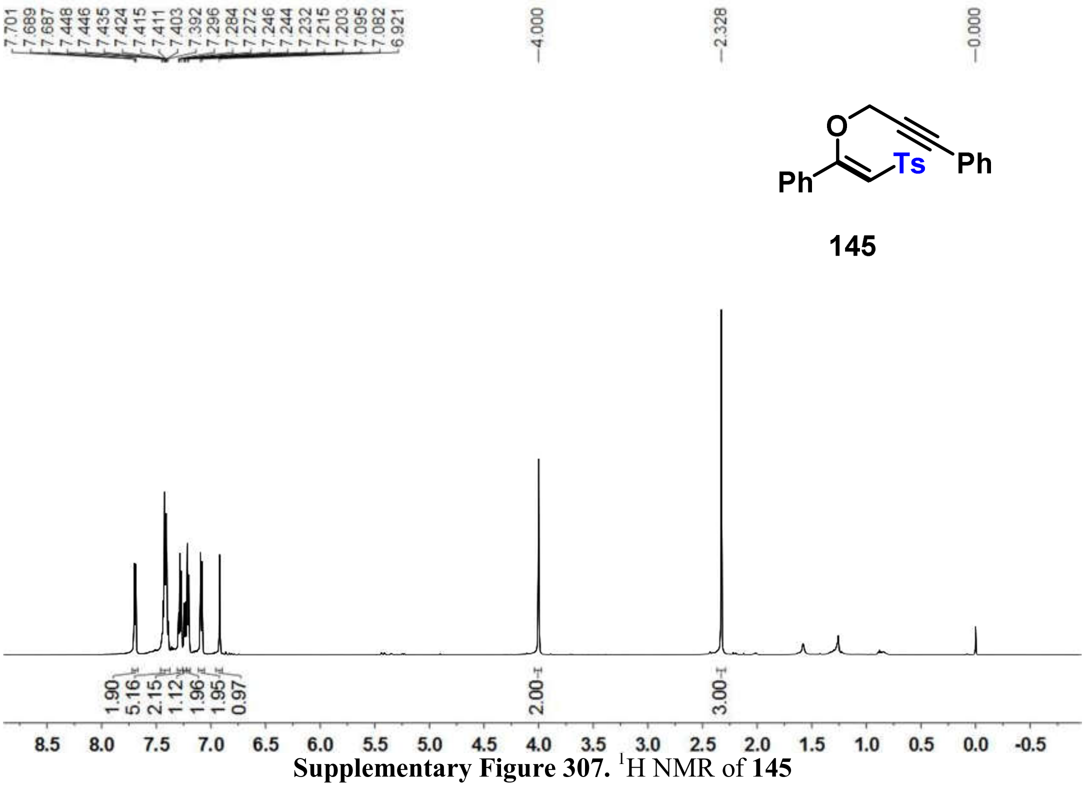

145

Supplementary Figure 307. ^{1}H NMR of **145**

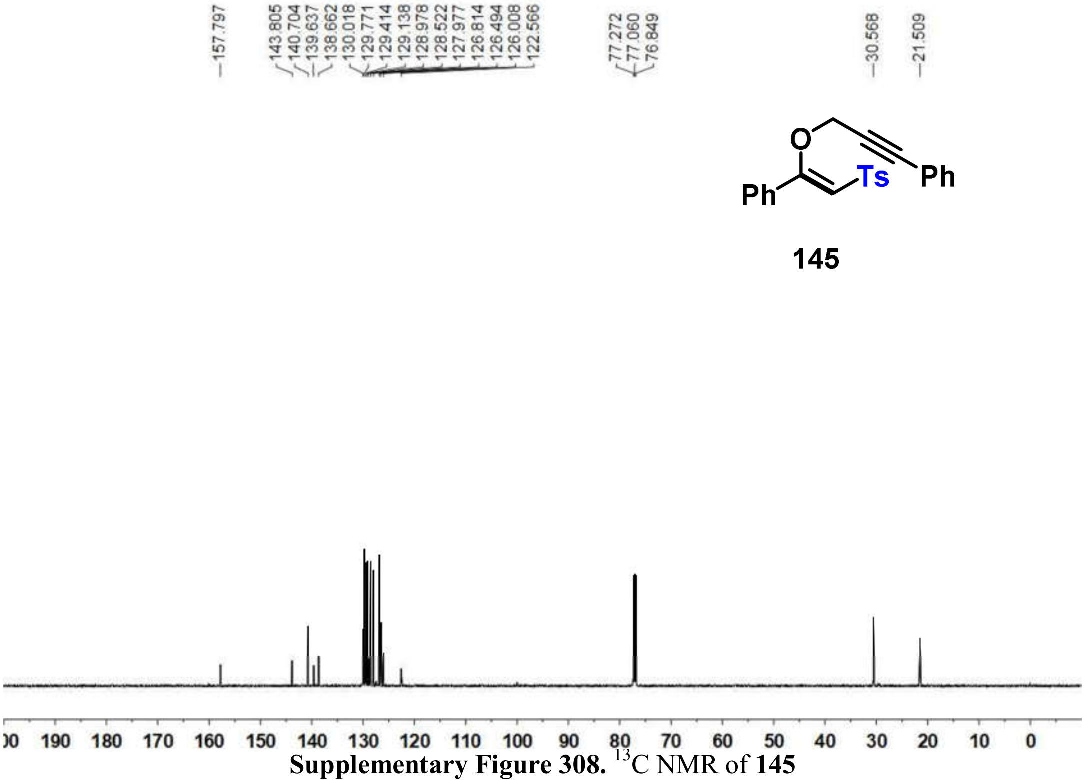

145

Supplementary Figure 308. ^{13}C NMR of **145**

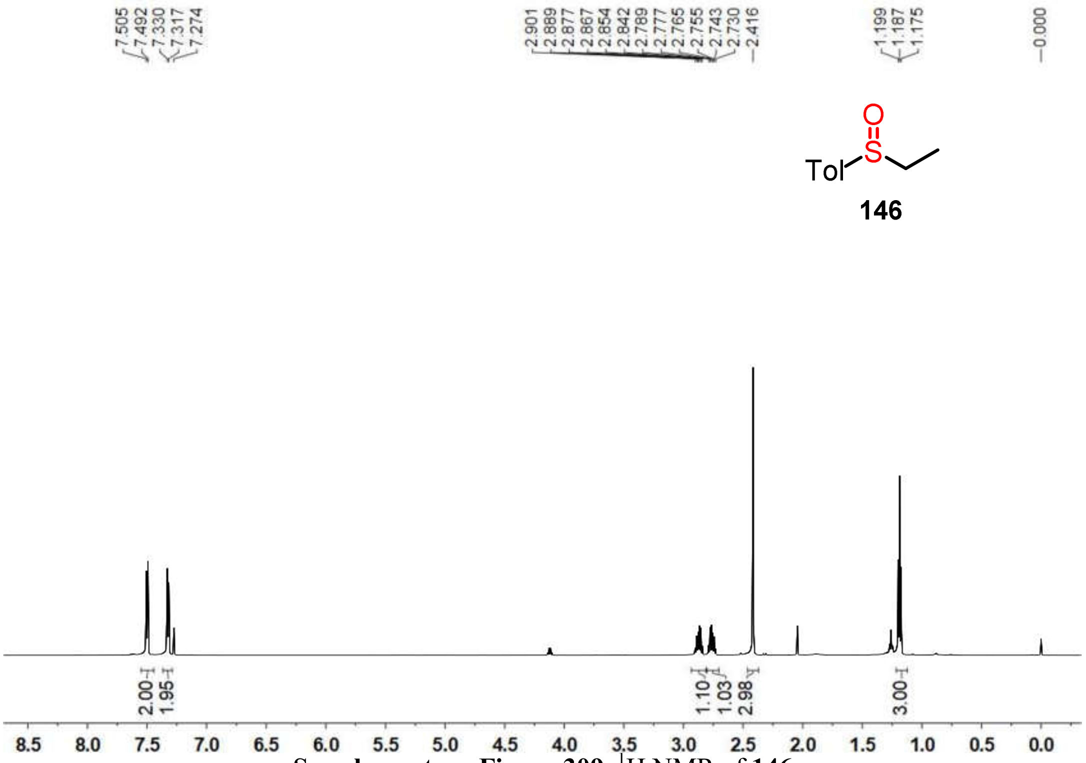

Supplementary Figure 309. ^{1}H NMR of **146**

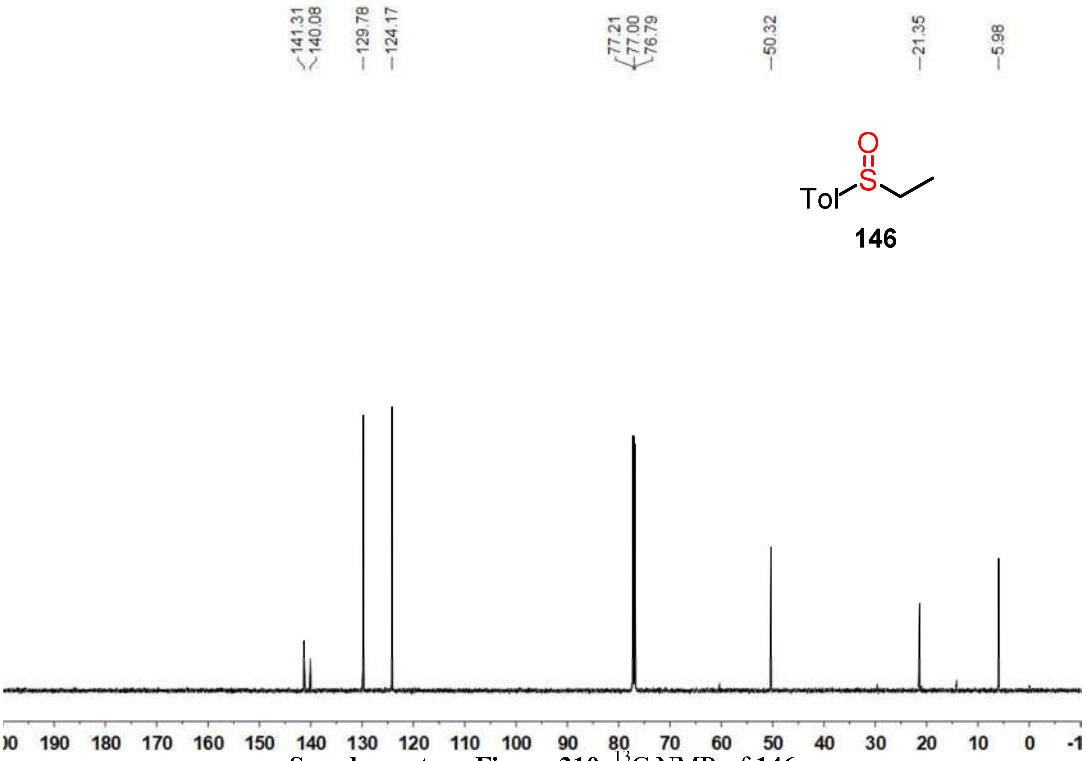

Supplementary Figure 310. ^{13}C NMR of **146**

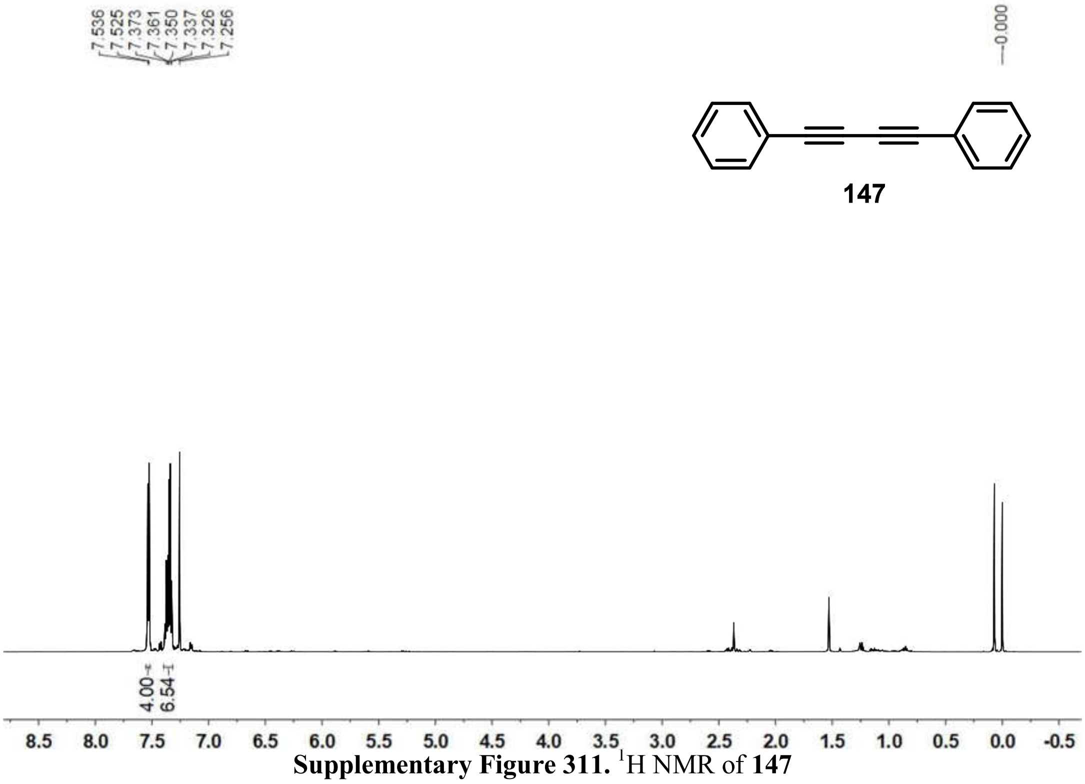

Supplementary Figure 311. ^{1}H NMR of **147**

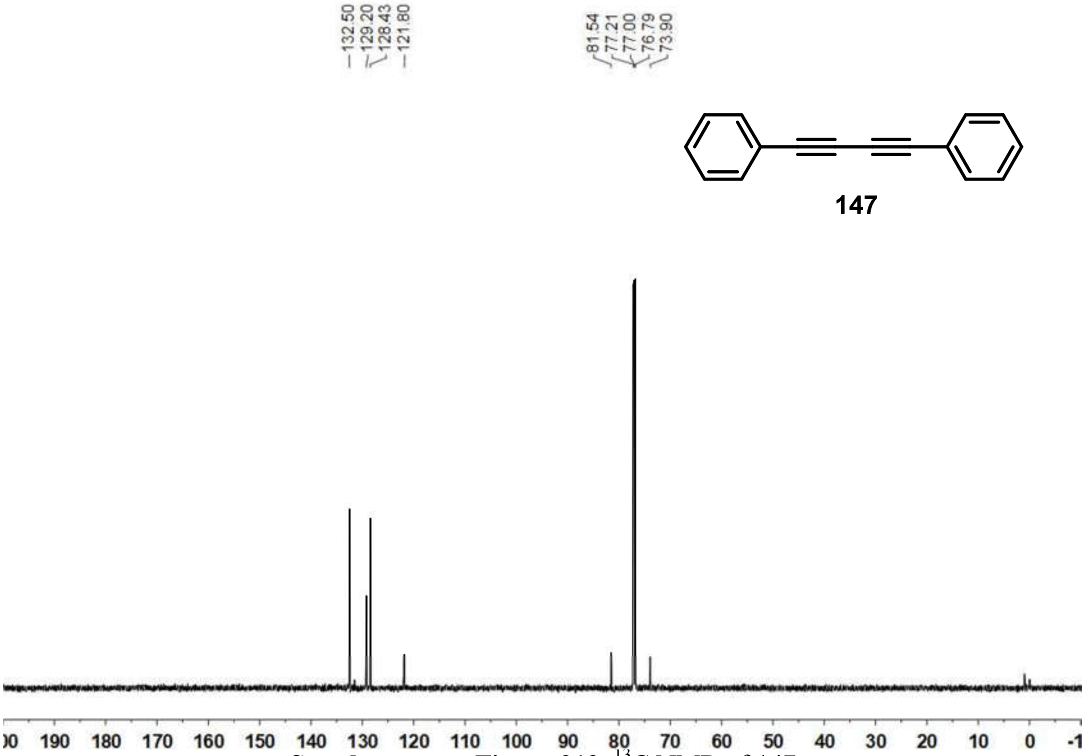

Supplementary Figure 312. ^{13}C NMR of **147**

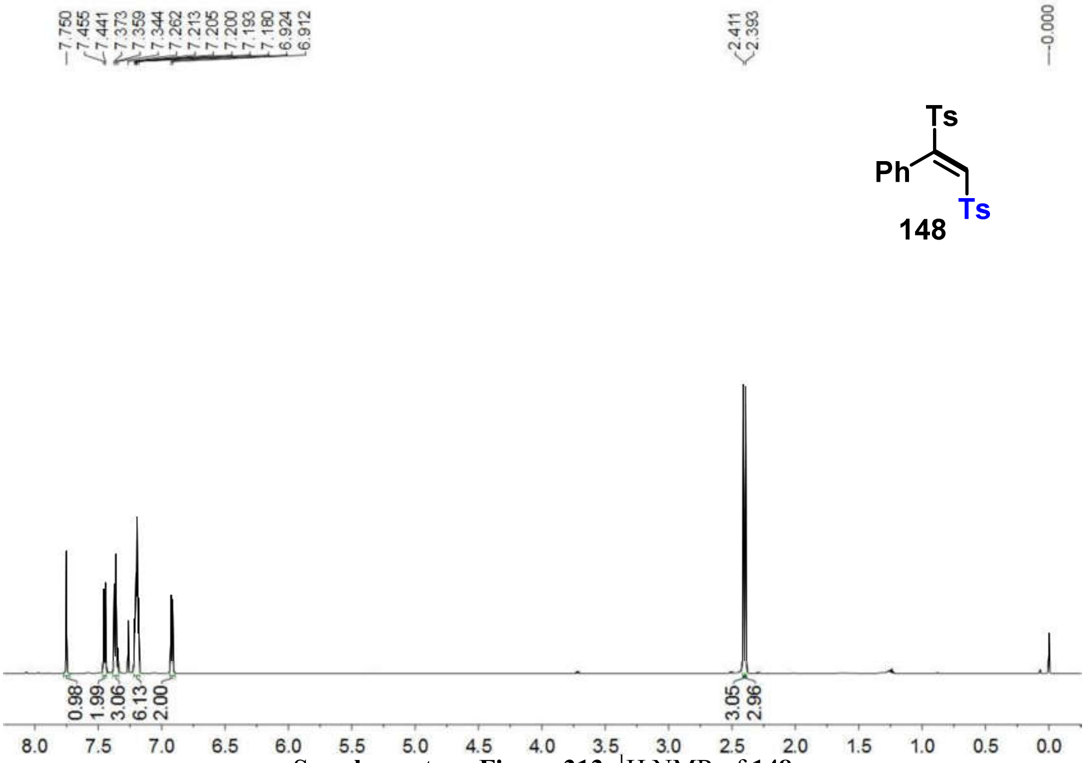

Supplementary Figure 313. ^{1}H NMR of **148**

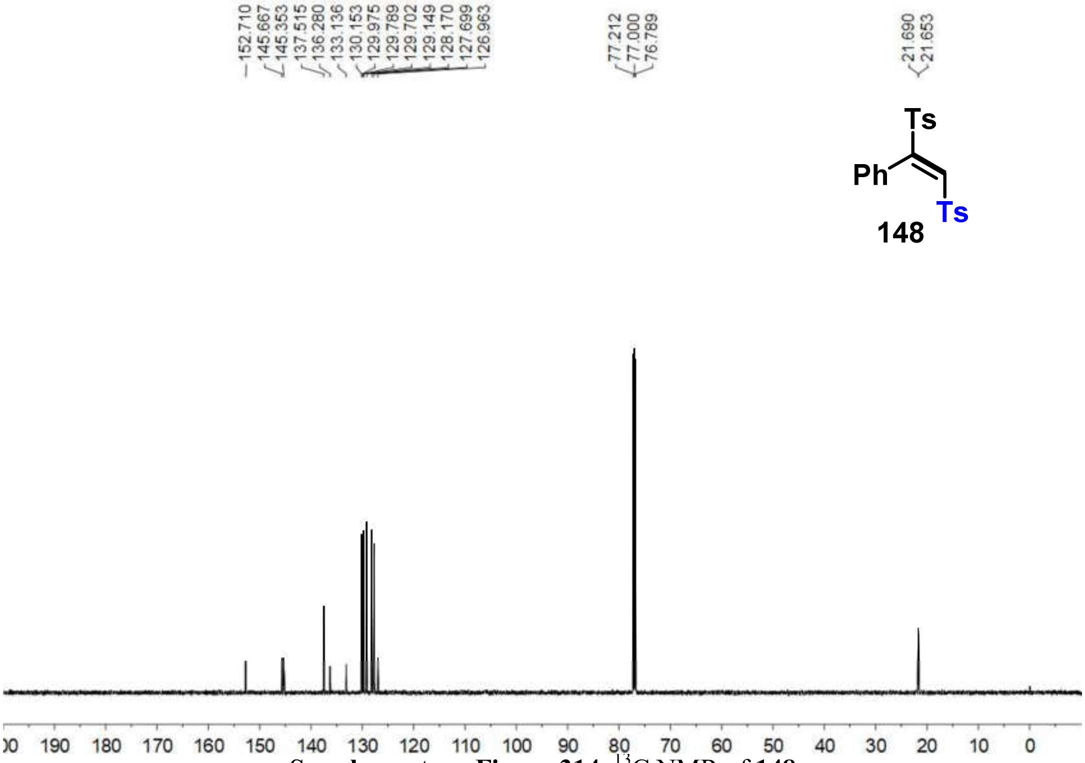

Supplementary Figure 314. ^{13}C NMR of **148**

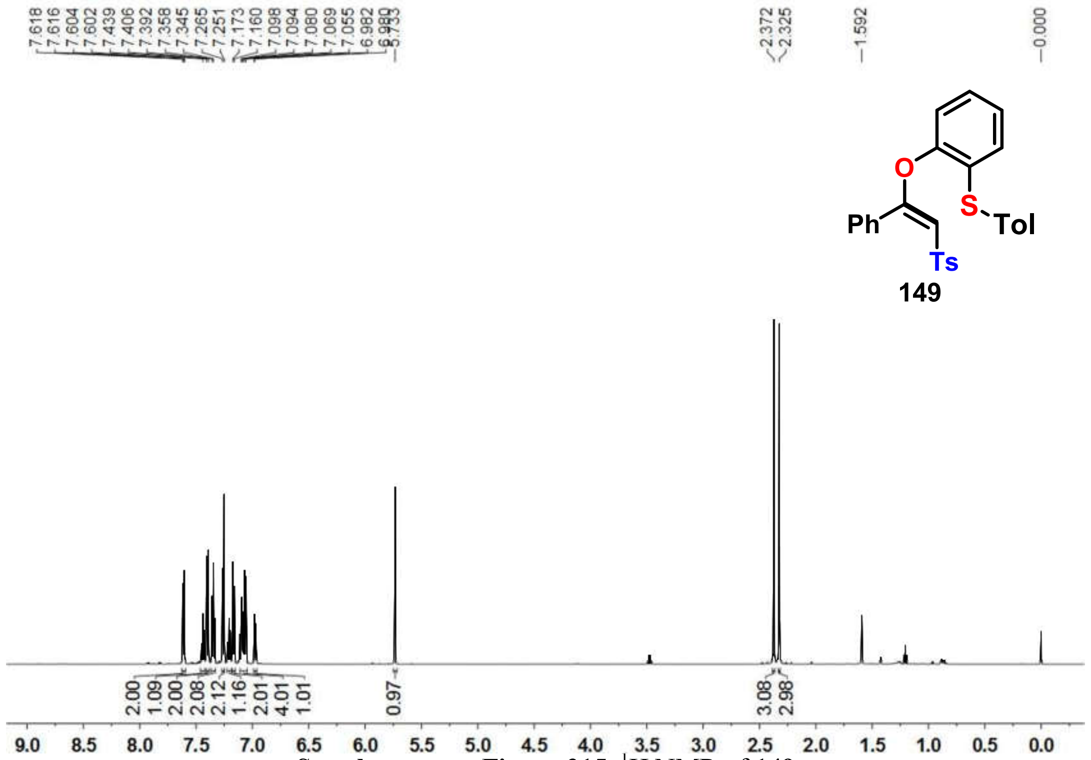

Supplementary Figure 315. ^{1}H NMR of **149**

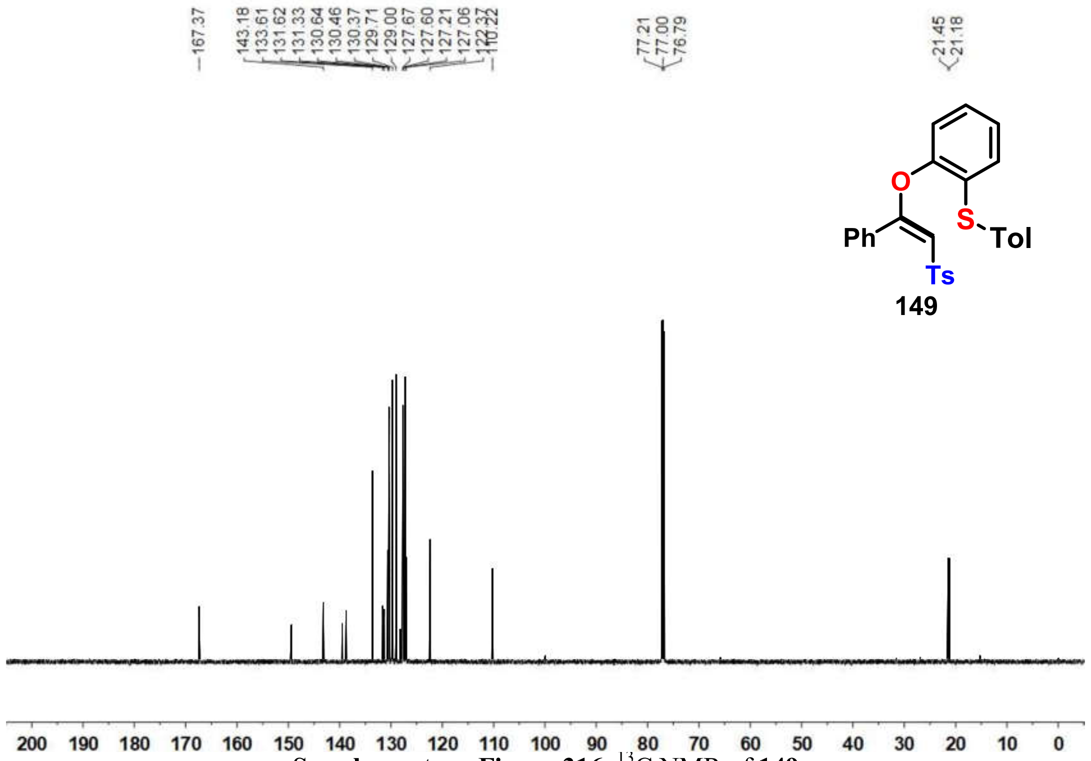

Supplementary Figure 316. ^{13}C NMR of **149**

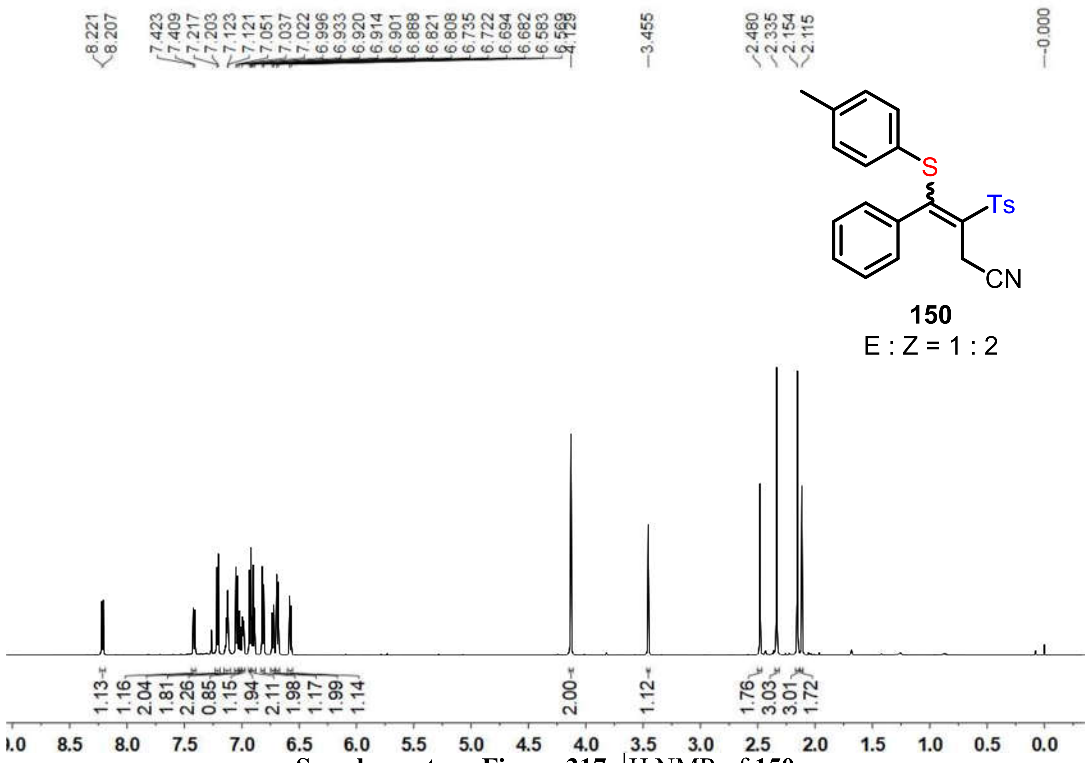

Supplementary Figure 317. ^{1}H NMR of **150**

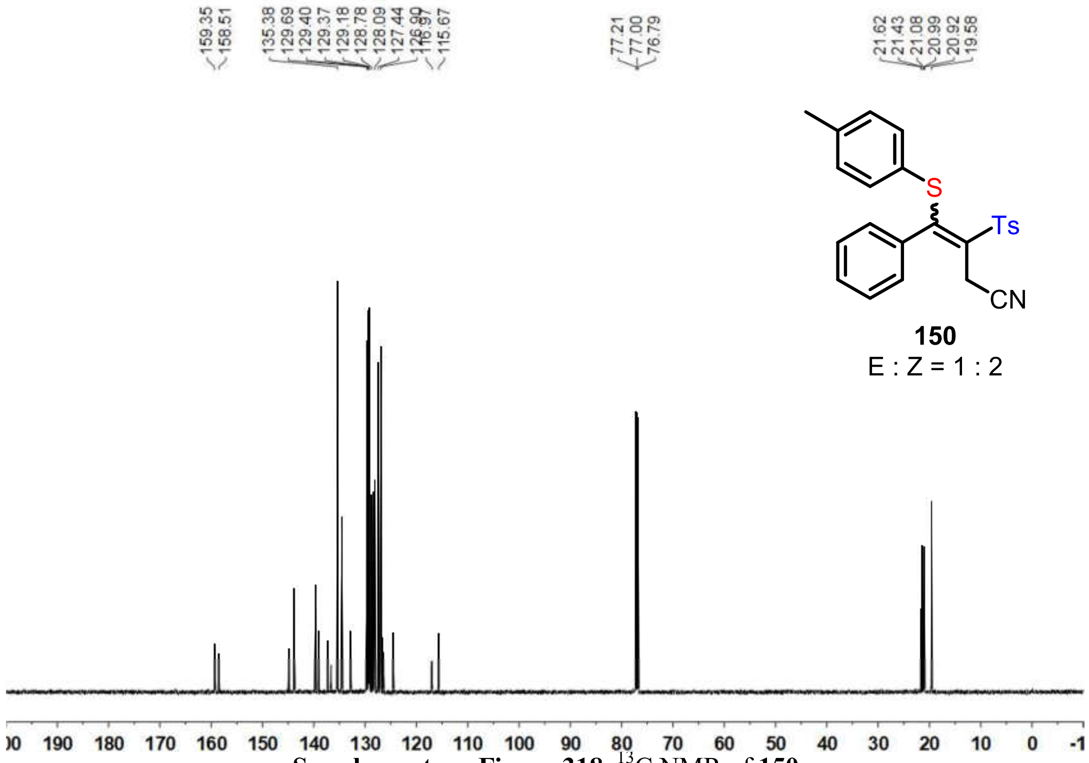

Supplementary Figure 318. ^{13}C NMR of **150**

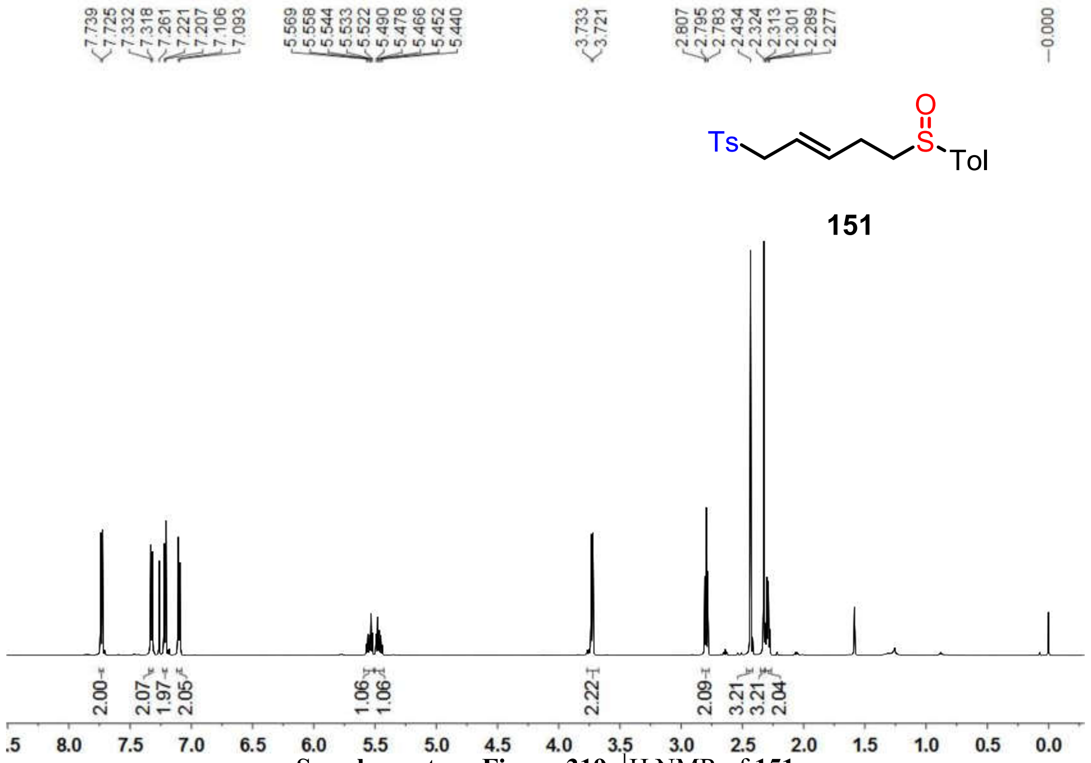

Supplementary Figure 319. ^{1}H NMR of **151**

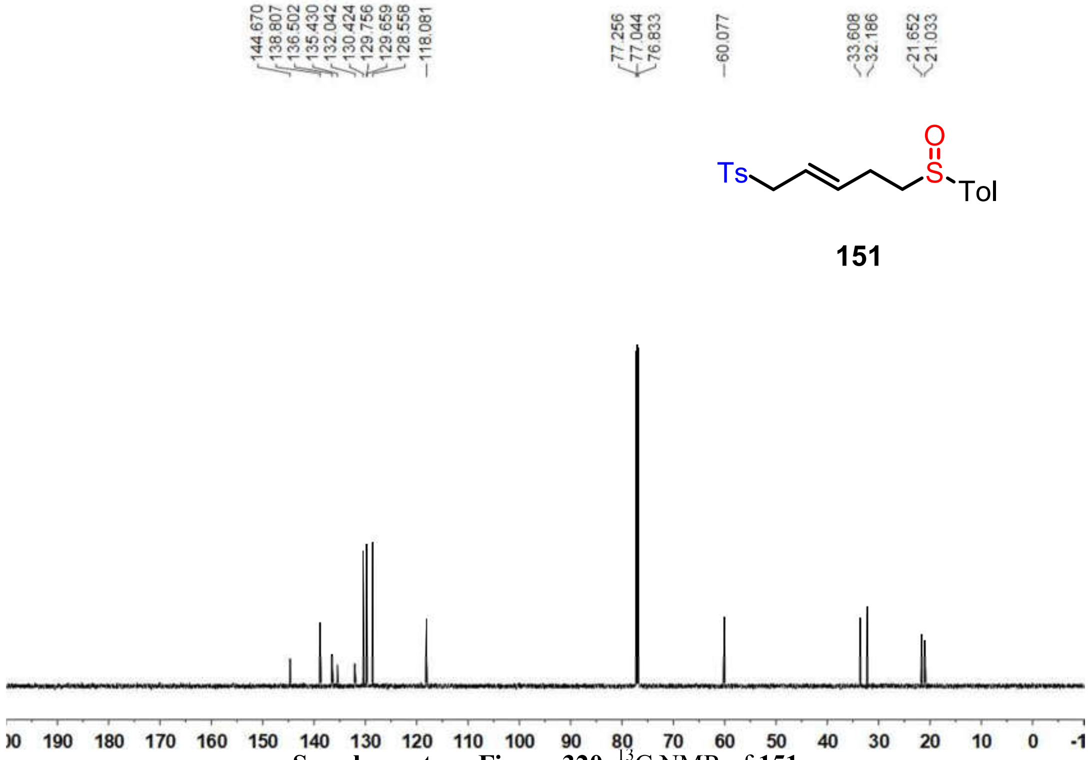

Supplementary Figure 320. ^{13}C NMR of **151**

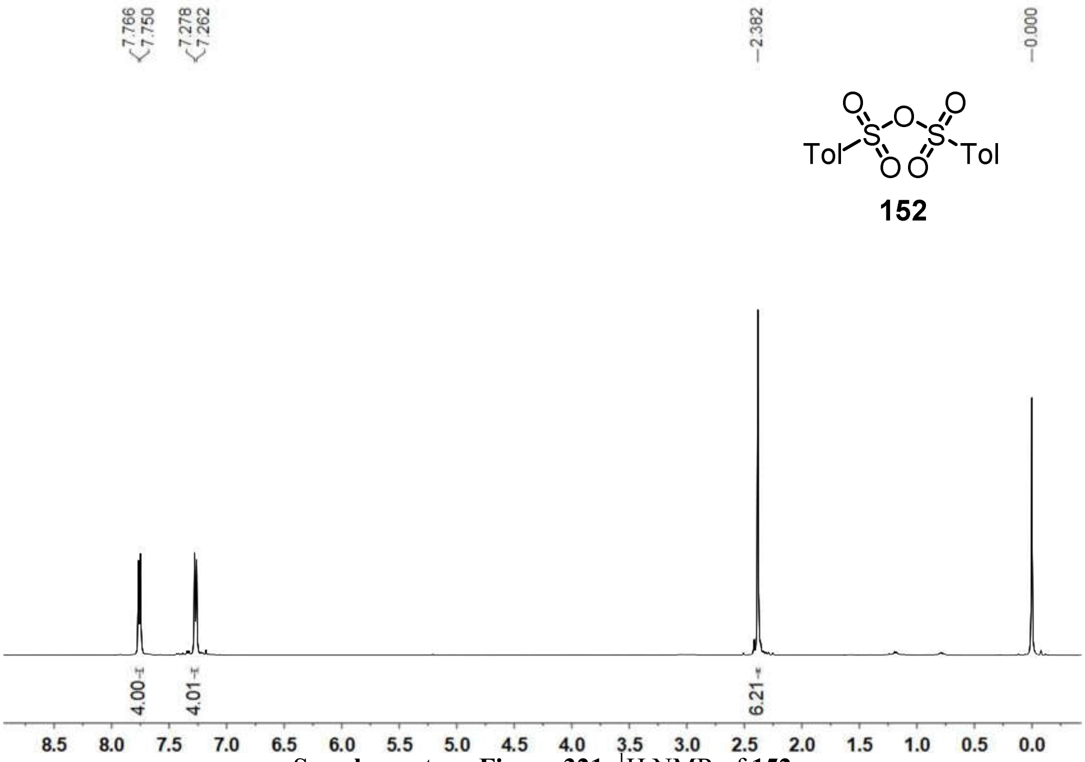

Supplementary Figure 321. ^{1}H NMR of **152**

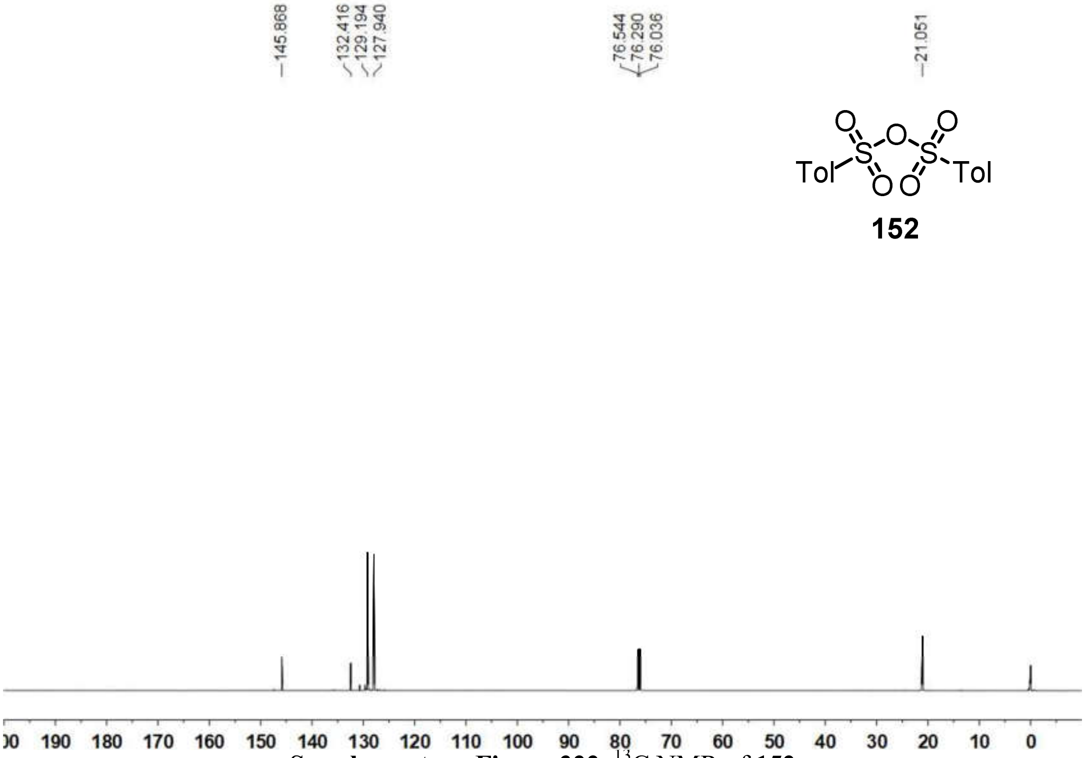

Supplementary Figure 322. ^{13}C NMR of **152**

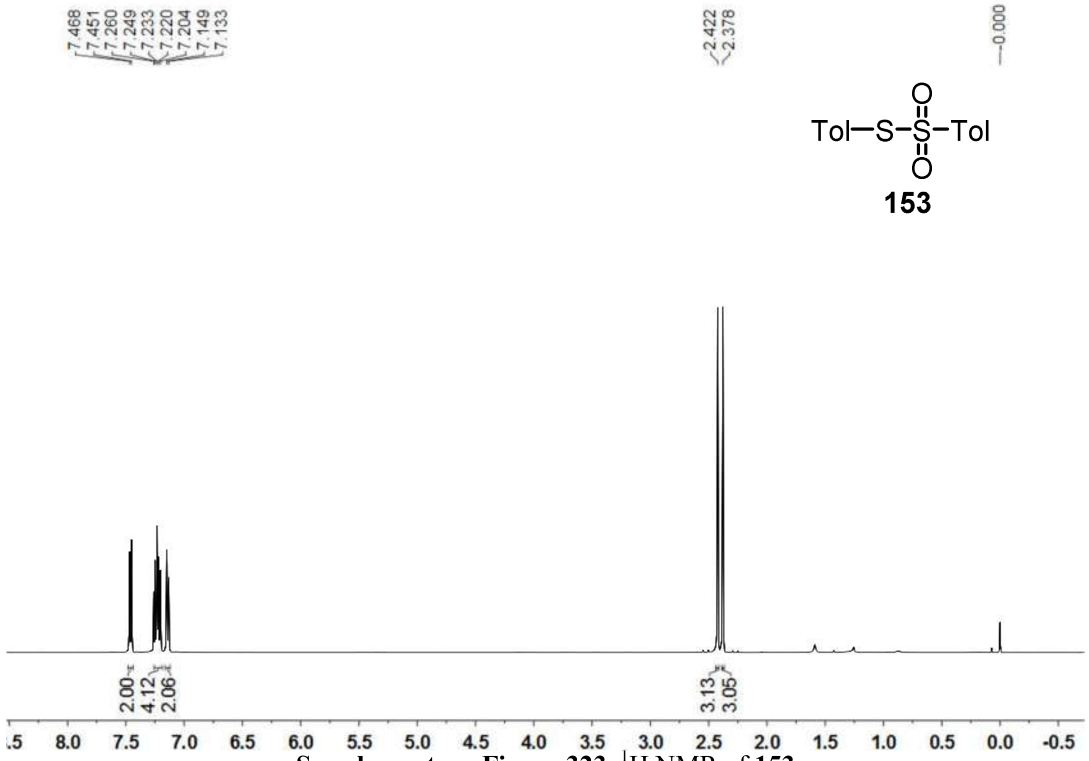

Supplementary Figure 323. ^{1}H NMR of **153**

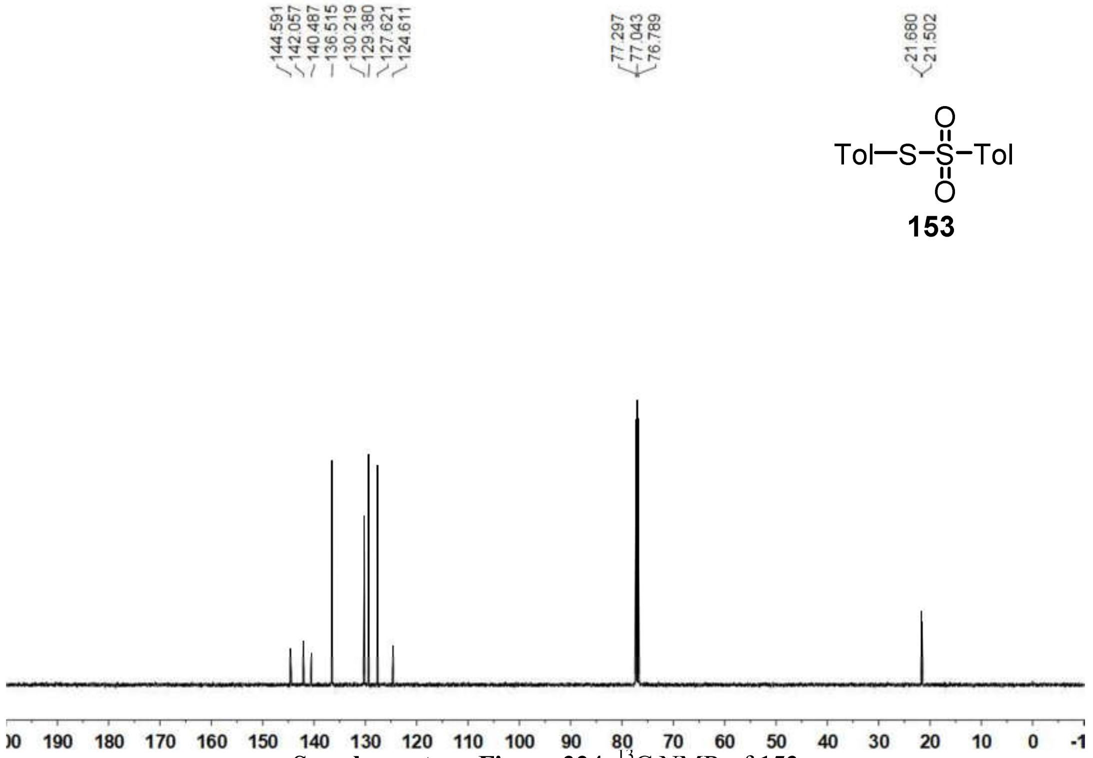

Supplementary Figure 324. ^{13}C NMR of **153**

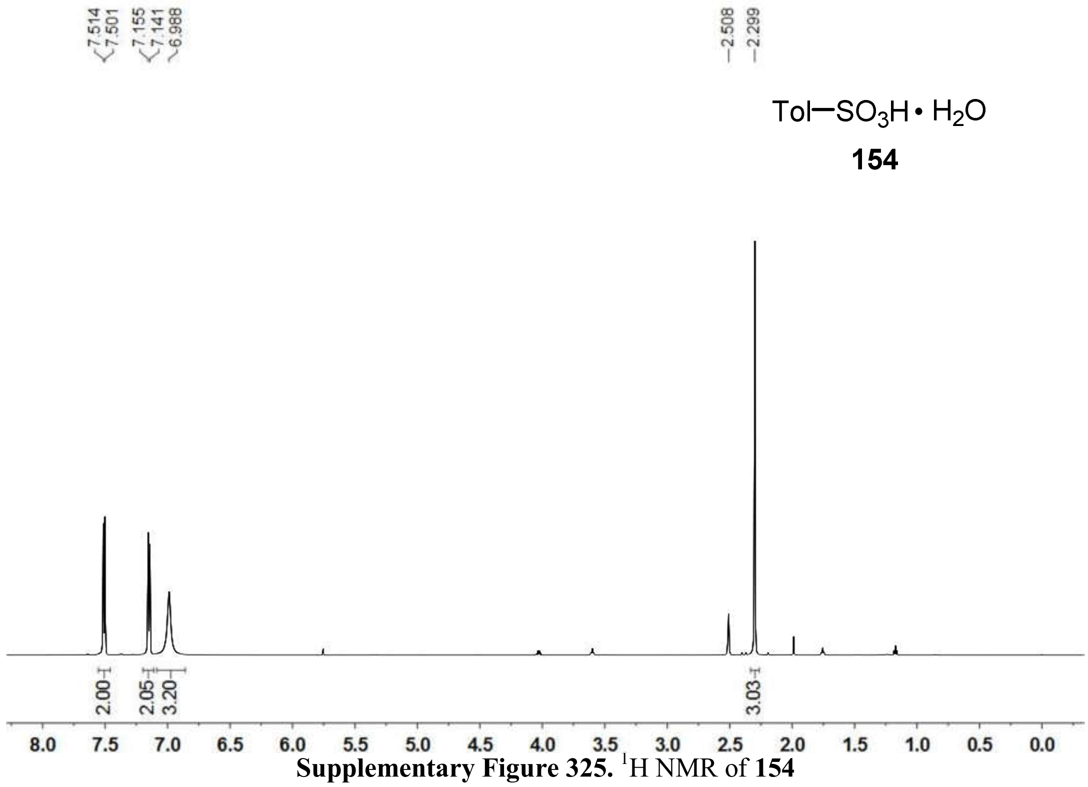

Supplementary Figure 325. ^{1}H NMR of **154**

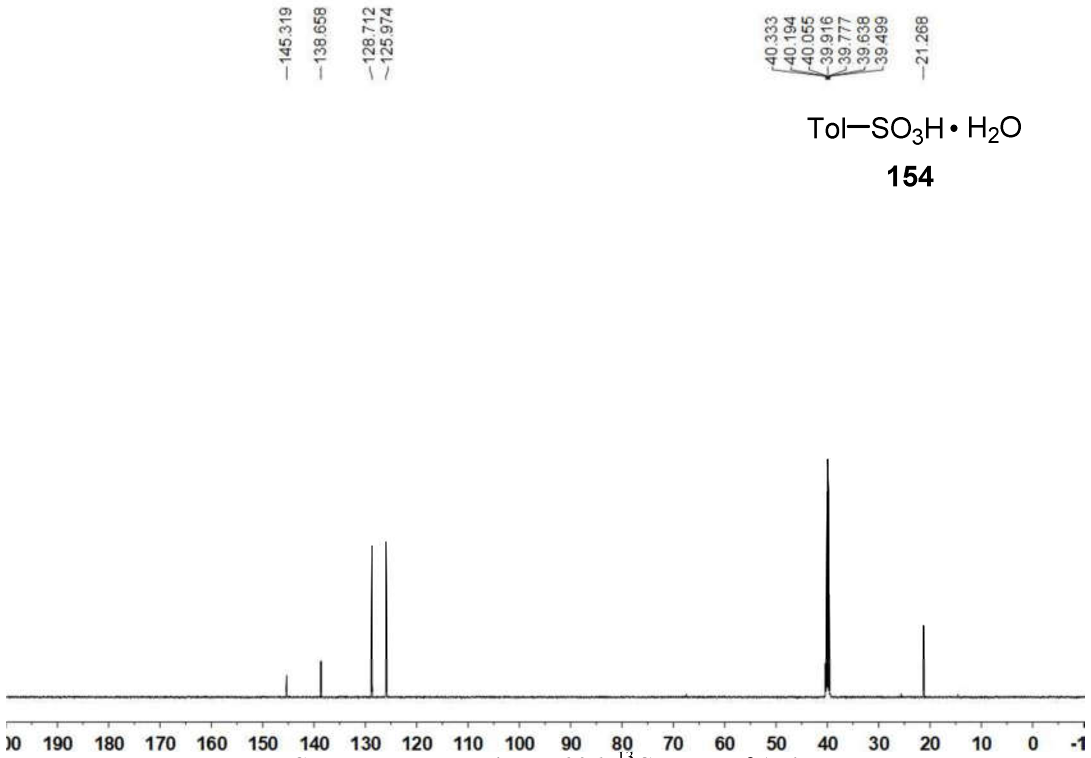

Supplementary Figure 326. ^{13}C NMR of **154**

Supplementary References

1. Mark, I. E., Murphy, F. & Dolan, S. Efficient preparation of trisubstituted alkenes using the Julia-Lythgoe olefination of ketones. On the key-role of SmI_2 in the reductive elimination step. *Tetrahedron Lett*. **37**, 2089–2092 (1996).
2. Meek, J. S. & Fowler. J. S. Nucleophilic addition-elimination reactions of 1,2-bis(p-tolylsulfonyl)ethane. *J. Org. Chem*. **33**, 985–991(1968)
3. Peng, B., Huang, X., Xie, L. -G. & Maulide, N. A brønsted acid catalyzed redox arylation. *Angew. Chem. Int. Ed*. **53**, 8718–8721 (2014).
4. Yuanming, L., Armido, S. Reaction of Arynes with Vinyl Sulfoxides: Highly Stereospecific Synthesis of *ortho*-Sulfinylaryl Vinyl Ethers. *Org. Lett.* **19**, 666–669 (2017).
5. Li, S., Yonghui, C., Fan, L., Jia-Ni, H., Xin, H., Lei, Z., Lichun, K., Kaixiao, L., Bo, P. Redox-Neutral α-Arylation of Alkyl Nitriles with Aryl Sulfoxides: A Rapid Electrophilic Rearrangement. *J. Am. Chem. Soc.* **139**, 4211−4217 (2017).
6. Frisch, M. J. et al. *Gaussian 09, Revision D.01*; Gaussian, Inc.: Wallingford, CT, 2009.
7. Lee, C. T. et al. Development of the Colle-Salvetti correlation-energy formula into a functional of the electron density. *Phys. Rev. B*: *Condens.Matter Mater. Phys*. **37**, 785–789 (1988).
8. Becke, A. D. Density-functional thermochemistry. III. The role of exact exchange. *J. Chem. Phys*. **98**, 5648–5652 (1993).
9. Fukui, K. Formulation of the reaction coordinate. *J. Phys. Chem*. **74**, 4161–4163 (1970).
10. Fukui, K. The path of chemical reactions – the IRC approach. *Acc. Chem. Res*. **14**, 363–368 (2002).
11. Grimme, S., Antony, J., Ehrlich S. & Krieg, H. A consistent and accurate ab initio parameterization of density functional dispersion correction (DFT-D) for the 94 elements H-Pu, *J. Chem. Phys*., **132**, 154104: (1-19) (2010).
12. Legault, C.Y. CYLview, version 1.0b; 2009 (http://www.cylview.org/).
